# Modular Synthetic Platform for the Elaboration of Fragments in Three Dimensions for Fragment-Based Drug Discovery

**DOI:** 10.1021/jacs.5c08786

**Published:** 2025-07-30

**Authors:** Andres R. Gomez-Angel, Hanna F. Klein, Stephen Y. Yao, James R. Donald, James D. Firth, Rebecca Appiani, Cameron J. Palmer, Joshua Lincoln, Simon C. C. Lucas, Lucia Fusani, R. Ian Storer, Peter O’Brien

**Affiliations:** † Department of Chemistry, 8748 University of York, York YO10 5DD, U.K.; ‡ Hit Discovery, Discovery Sciences, R&D, AstraZeneca, 1 Francis Crick Ave, Cambridge CB2 0AA, U.K.

## Abstract

Fragment-based drug discovery (FBDD) is a key strategy employed in the hit-to-lead phase of pharmaceutical development. The rate-limiting step of this process is often identifying and optimizing synthetic chemistry suitable for fragment elaboration, especially in three dimensions (3-D). To address this limitation, we herein present a modular platform for the systematic and programmable elaboration of two-dimensional (2-D) fragment hits into lead-like 3-D compounds, utilizing nine bifunctional building blocks that explore a range of vectors in 3-D. The building blocks comprise (i) rigid sp^3^-rich bicyclic cyclopropane-based structures to fix the vectors and (ii) two synthetic handles—a protected cyclic amine and a cyclopropyl *N-*methyliminodiacetic acid (MIDA) boronate. To validate our approach, we present (i) multigram-scale synthesis of each 3-D building block; (ii) Suzuki-Miyaura cross-coupling reactions of the cyclopropyl BMIDA functionality with aryl bromides; and (iii) *N*-functionalization (via commonplace medicinal chemistry toolkit reactions) of arylated products to deliver 3-D lead-like compounds. Each building block accesses a distinct 3-D exit vector, as shown by analysis of the lowest energy conformations of lead-like molecules using RDKit, and by X-ray crystallography of pyrimidine methanesulfonamide derivatives. Since the synthetic methodology is established in advance of fragment screening and utilizes robust chemistry, the elaboration of fragment hits in 3-D for biochemical screening can be achieved rapidly. To provide proof-of-concept, starting from the drug Ritlecitinib, the development of inhibitors of Janus kinase 3 (JAK3) around a putative pyrrolopyrimidine 2-D fragment hit was explored, streamlining the discovery of a novel and selective JAK3 inhibitor with IC_50_ = 69 nM.

## Graphical abstract

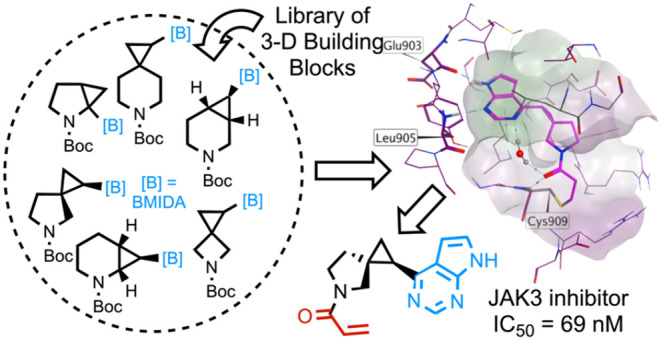

## Introduction

Fragment-based drug discovery (FBDD) has developed into a mature technology for the identification of low molecular weight hits against protein targets and subsequent progression to lead candidates.^1–5^ Indeed, eight drugs, along with over 59 additional clinical candidates, have originated from FBDD programs.^6,7^ Due to the low molecular weight (MW) of fragments (MW typically <300 Da),^8^ establishing and employing a fragment library that can effectively sample chemical space (typically a few thousand compounds) is far cheaper and more straightforward than utilizing a high-throughput screening library.^3,4,9^ This is perhaps one of the reasons for its popularity. A key part of FBDD is “growing” or “elaborating” fragment hits (often guided by X-ray crystallographic studies) to increase the potency and to obtain lead compounds. It is often desirable that this fragment growth can be performed along multiple vectors in 3-D space, potentially from sp3 carbons, allowing the synthesis of 3-D lead-like molecules with associated desirable physicochemical properties.^10,11^ However, fragment growth is often limited to commercially available structural analogs of fragment hits, and even seemingly simple fragment growth design ideas can require de novo synthetic strategies. Further limitations can arise from the focus of medicinal chemists on a small toolkit of reactions, especially the formation of sp^2^–sp^2^ (aryl–aryl) C–C bonds.^12,13^ For these reasons, synthetic organic chemistry has been highlighted as the rate-limiting step in the fragment growth/elaboration stage,^14,15^ and this led to a call from FBDD industrial practitioners to academia to develop methods to allow the synthetic “elaboration of fragments in three dimensions from many different growth points/vectors using methodology that is worked out prior to fragment screening.”^14^

One approach to achieve this goal is the development of new fragments with accompanying synthetic methodology that facilitates growth along multiple vectors.^16^ However, this approach requires significant up-front investment of synthetic chemistry resources, as highlighted in our previous work on 3-D fragment libraries.^17–20^ A second approach would be the direct growth of existing fragments along non-traditional vectors through C–H activation methods that are site-selective for different growth points on a fragment, while being robust enough to be compatible with essential polar functionality.^21–24^ In both of these contexts, the term “fragment sociability” has been identified to distinguish between fragments for which fragment elaboration is synthetically enabled (“sociable”) from those where it is not (“unsociable”).^25^ Despite some progress in both of these approaches, the extensive structural diversity presented by 3-D molecular architectures, the polar functionalities required in medicinal chemistry settings, and the speed with which drug discovery projects move forward mean that progress has been slow and such a synthetic ideal for fragment elaboration currently remains elusive.

As a result, we propose a different approach, namely, the development of a modular synthetic platform that would enable the systematic and programmable elaboration of typically 2-D fragment hits into 3-D lead-like compounds. This alternative approach should enable fragment elaboration across a range of 3-D vectors, and key features are captured in Figure 1A,1B. Rather than the development of complex elaboration methodology for each fragment class prior to screening, our approach utilizes the power and robustness of the limited, but reliable, toolkit of reactions that are commonly employed in medicinal chemistry.^12,13,26^ This, together with a newly designed collection of bifunctional 3-D building blocks **1a**-**i** (Figure 1C) equipped with distinct vectors (vide infra), will enable the rapid and systematic fragment growth of 2-D fragments hits.

**Figure 1:**
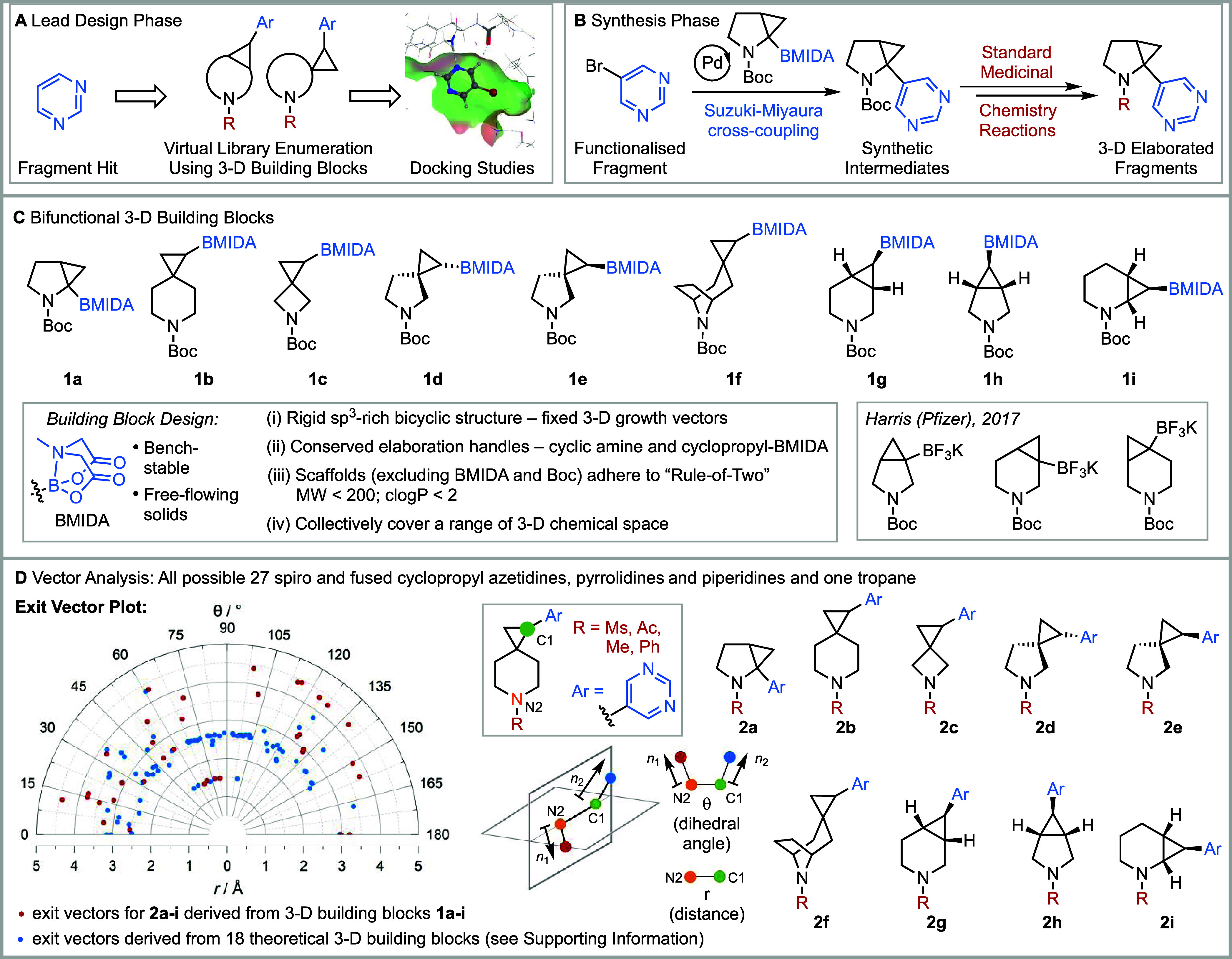
Modular synthetic platform for fragment elaboration in 3-D and the design and analysis of 3-D building blocks. (A) Lead design phase. (B) Synthesis phase. (C) Bifunctional 3-D building blocks. (D) Exit vector analysis and exit vector plot (where applicable, only one enantiomeric series for each compound is displayed on the exit vector plot).

The approach, which is best suited to working with proteins that are structurally enabled, begins with the lead design phase (Figure 1A). For example, consider that pyrimidine is a fragment hit against the protein target of interest and binding has been confirmed by X-ray crystallography. The set of 3-D building blocks **1a**-**i** (Figure 1C) can then be used to enumerate a virtual library of 3-D lead-like compounds in which the pyrimidine fragment hit (blue) has been diversified with the building blocks and typical medicinal chemistry capping groups (red) to pick up additional protein binding interactions. Computational docking studies of this virtual library will then reveal a set of potential lead-like molecules for synthesis comprising the pyrimidine fragment (blue) and 3-D building block linker scaffolds that display the additional binding groups (red) along the 3-D vector provided by each building block. Next, there are two parts to the synthesis phase (Figure 1B). First, elaboration of the pyrimidine fragment hit (blue) will be performed by cross-coupling a commercially available brominated analog, 3-bromopyrimidine in this case, with the selected 3-D building blocks, illustrated with **1a**. Second, after Boc group removal, *N*-functionalization will be carried out to place the capping groups (red) in particular 3-D vectors relative to the initial fragment hit (blue). We envisioned that both stages of the synthesis phase would utilize reactions that are robust and widely used in medicinal chemistry to minimize any barriers to uptake by FBDD practitioners; *N*-functionalization using amide/sulfonamide formation, Buchwald-Hartwig cross-coupling, S_N_Ar (nucleophilic aromatic substitution), and reductive amination have been used in this work. Since the synthetic methodology is established in advance of fragment screening and utilizes the same set of reactions, the generation of fragment hits that are elaborated in 3-D for biochemical screening can be achieved rapidly. After these initial screening results, the process can be applied iteratively to further optimize the compounds.

The utility of our approach hinges on the availability of a collection of synthetically enabled bifunctional building blocks that access a range of 3-D vectors and 3-D chemical space. With this in mind, we designed the set of 3-D building blocks **1a**-**i** shown in Figure 1C using the following criteria: (i) they have a rigid sp^3^-rich bicyclic structure (fused or spirocyclic) comprising a cyclopropane^27,28^ to fix the 3-D vectors between the fragment and the *N-*capping groups (vide infra); (ii) they are equipped with the same two synthetic handles, namely, a Boc-protected cyclic amine and a cyclopropyl *N-*methyliminodiacetic acid (MIDA) boronate;^29^ (iii) the scaffolds (i.e., excluding the BMIDA and Boc groups) have suitable physicochemical properties, achieved by adhering to AstraZeneca’s “Rule-of-Two” guidelines for medicinal chemistry building blocks^30^ (MW < 200; clogP < 2); and (iv) they cover, collectively, a range of 3-D chemical space (vide infra).

The incorporation of cyclopropyl MIDA boronates into 3-D building blocks **1a**-**i** as the fragment elaboration handle is strategic. First, since many fragment libraries contain a high proportion of aryl and heteroaryl compounds, it was envisaged that any such fragment hit could be elaborated from a halogenated analog (including FragLites which are a screening set of ∼ 30 medicinally relevant aryl bromides and iodides^31,32^ ) using well-established, reliable Suzuki-Miyaura cross-coupling (Figure 1B, synthesis phase).^33–38^ Second, MIDA boronates are known to be bench-stable, free-flowing crystalline solids that can be easily purified, making them ideal linchpin building blocks, as shown by Burke’s automated syntheses.^29^ At the outset of our project, the closest structural analogs to 3-D building blocks **1a**-**i** were fused cyclopropyl pyrrolidine and piperidine BF_3_K salts reported by Harris et al. at Pfizer^39^ (Figure 1C), which were not designed with fragment elaboration in mind, and difluorocyclopropane analogs of **1b**, **1c** and Harris’ fused piperidines, as described by Grygorenko et al.^40^ After the development of our synthetic routes to racemic 3-D building blocks **1a**-**i** (excluding **1h** which is a *meso* compound), Gutiérrez-Bonet, Popov and co-workers at Merck reported the asymmetric synthesis of cyclopropyl boronate analogs of **1c** and Harris’ cyclopropyl pyrrolidine and piperidine BF_3_K salts.^38^

A key design feature of 3-D building blocks **1a**-**i** was that they would provide a range of 3-D vectors and 3-D chemical space coverage (Figure 1D). For this, 3-D vector analysis was carried out by enumeration of relevant chemical space in tandem with Grygorenko’s exit vector plot analysis.^41,42^ To start, virtual 3-D bifunctional building blocks comprising all 26 possible combinations of a cyclic amine (derived from azetidine, pyrrolidine or piperidine) and a fused or spirocyclic cyclopropyl BMIDA group were enumerated (compounds are racemic or *meso* and included diastereomers where relevant, see Supporting Information for details). This was augmented with a synthetically accessible tropane building block **1f** to give a total of 27 theoretical 3-D building blocks that fitted within our design criteria (i)–(iii). Then, using pyrimidine as a plausible fragment hit (5-bromopyrimidine is a FragLite^31,32^ ), virtual elaboration on all 27 scaffolds was carried out in conjunction with *N*-capping with methanesulfonyl, acetyl, methyl, and phenyl groups. This gave 112 virtual lead-like compounds (effectively elaborated fragments, examples shown from the nine building blocks, **2a**-**i**) whose lowest energy conformations were determined using RDKit, an open-source cheminformatics platform. For bifunctional scaffolds, such as those present in the enumerated 112 lead-like compounds, the vectors *n*_*1*_ and *n*_*2*_ can be defined by two geometric parameters: the distance through space, *r*, between the variation points C1 (green atom) and N2 (orange atom) and the dihedral angle, θ, between the two planes defined by the vectors *n*_*1*_, C1–N2 and *n*_*2*_ (defined by the red, orange, green, and blue atoms). The parameters *r* and θ are then readily obtained from the atomic coordinates of the lowest energy conformers of the virtual compounds and allow the construction of a plot of *r* vs θ.^41^ Figure 1D shows an exit vector plot of *r* vs θ for the 112 lead-like compounds derived from the 27 theoretical 3-D building blocks. Points shown in red correspond to the exit vectors for lead-like compounds **2a**-**i** (derived from the nine 3-D building blocks **1a**-**i**) and those in blue are derived from the remaining 18 theoretical 3-D building blocks. The combined plot of red and blue points shows that a wide range of dihedral angles (θ) and spacings (*r*) is available from the 27 scaffolds. From this plot, we selected 3-D building blocks based on their anticipated ease of synthesis and the fact that they provided a range of distinct 3-D vectors. This ultimately led to the selection of nine 3-D building blocks **1a**-**i**.

In this paper, we set out the design principles underpinning our modular synthetic platform that enables the straightforward elaboration of typically 2-D fragment hits into 3-D lead-like compounds for use in FBDD. The approach is illustrated with nine bifunctional 3-D building blocks **1a**-**i** (Figure 1C), whose multigram-scale synthesis is demonstrated. In particular, we present 65 Suzuki-Miyaura cross-coupling reactions between building blocks **1a**-**i** and medicinally relevant aryl bromides, including a selection of FragLites.^31,32^ Several of these cross-coupled arylated products are, after Boc group removal, *N*-functionalized to deliver 32 3-D lead-like compounds. Finally, having validated the synthetic elements of our approach, the synthetic platform is deployed in, and showcased with, the development of a novel and selective Janus kinase 3 (JAK3) inhibitor. Herein, we present our results.

## Results and Discussion

### Synthesis of 3-D Building Blocks

The different strategies employed for the gram-scale synthesis of each of the nine fused and spirocyclic bifunctional 3-D building blocks **1a**-**i** are summarized in Figure 2. Fused 2,3-pyrrolidine cyclopropane BMIDA building block **1a** was synthesized via the lithiation–trapping of 4-chloropiperidine **3** using a bespoke synthetic approach.^43,44^ Treatment of **3** with *s*-BuLi and TMEDA at −78 °C resulted in α-lithiation, followed by intramolecular cyclopropanation to generate the azabicyclo[3.1.0]­hexane ring system (Figure 2A, insert). A second α-lithiation event and subsequent trapping with trimethyl borate gave boronic acid **4**. Finally, conversion into the MIDA boronate using *N*-methyl-iminodiacetic acid (MIDA) and triethylorthoformate in DMSO at 100 °C gave the 3-D building block **1a** (4.2 g prepared in one batch) in 43% yield from **3** (Figure 2A). Use of *N*-2-benzyloxycyclopentyl-iminodiacetic acid (BIDA), an enantiopure chiral MIDA equivalent developed by Burke,^45–47^ allowed isolation of enantiomerically pure building blocks. In this exemplar case, condensation of **4** with enantiopure BIDA gave separable diastereomeric 3-D building blocks **1a’** and **1a’’** in 26% and 20% yield, respectively (Figure 2A). The configuration of **1a’** and **1a’’** was determined by conversion of **1a’** into a cross-coupled product of known configuration (vide infra).

**Figure 2:**
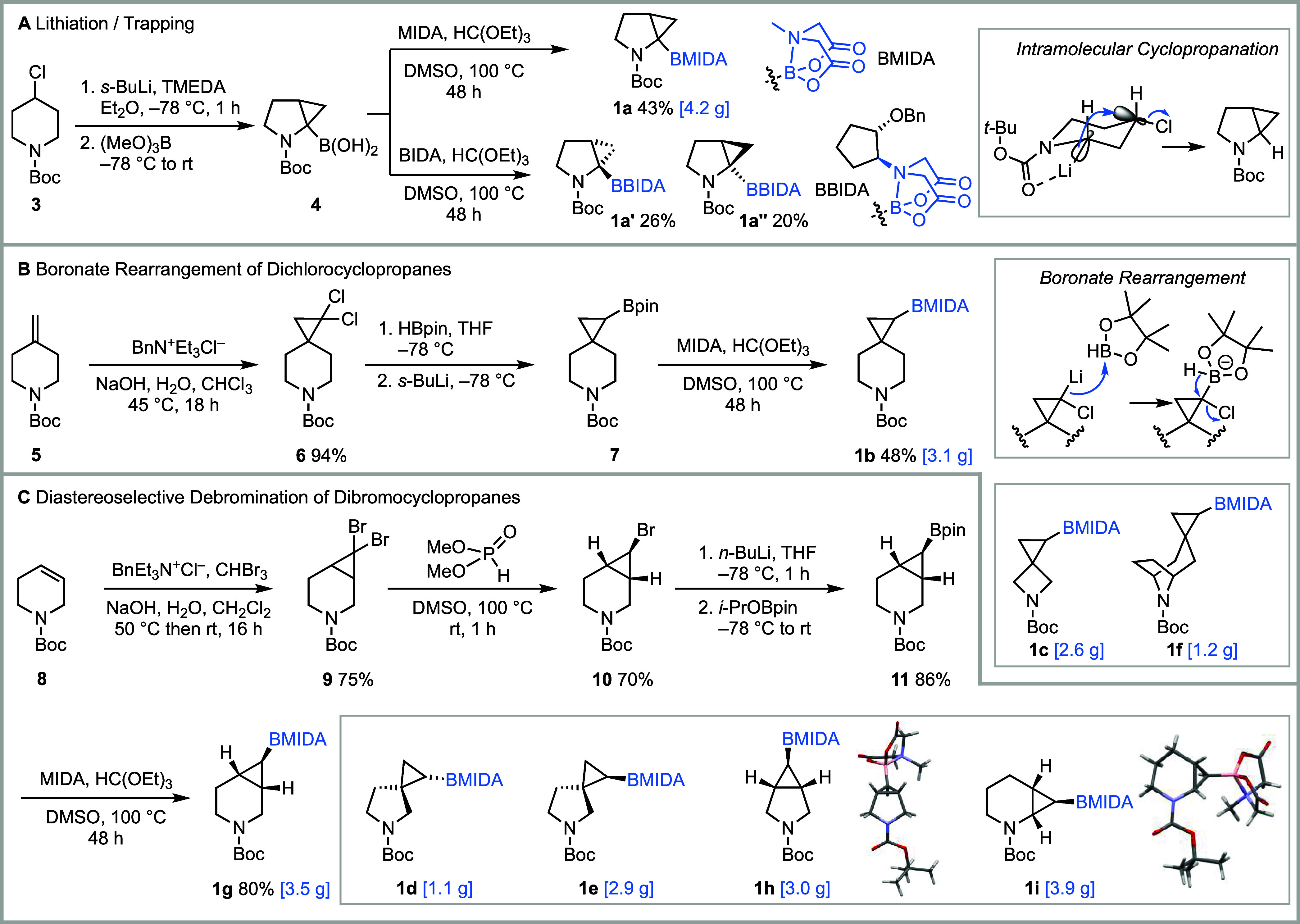
Synthesis of the *N*-Boc MIDA boronate building blocks. (A) Bespoke lithiation/trapping route. (B) Boronate rearrangement of dichlorocyclopropanes. (C) Diastereoselective debromination of dibromocyclopropanes.

For the synthesis of three spirocyclic 3-D building blocks **1b**, **1c**, and **1f**, a general strategy based on a boronate rearrangement of an intermediate derived from *gem*-dichlorocyclopropanes was developed (Figure 2B). As an example of this synthetic approach, dichlorocyclopropanation of exocyclic alkene-containing *N*-Boc piperidine **5** gave spirocyclic *gem*-dichlorocyclopropane **6** in 94% yield. Treatment of a mixture of **6** and pinacolborane with *s*-BuLi at −78 °C in THF for 30 min gave cyclopropyl pinacol boronate **7**. The reaction presumably proceeds via lithium–halogen exchange and trapping with HBpin followed by 1,2-hydride migration (Figure 2B, insert).^48,49^ Conversion of crude pinacol boronate **7** into the corresponding MIDA boronate gave the 3-D building block **1b** (3.1 g prepared in one batch) in 48% yield from **6**. This synthetic approach also enabled the preparation of 2.6 g of spiro-fused cyclopropyl azetidine **1c** and 1.2 g of cyclopropyl tropane **1f** (Figure 2B, insert). Tropane-based 3-D building block **1f** was produced as a single diastereomer as a result of a highly diastereoselective dichlorocyclopropanation reaction, with the dichlorocarbene added opposite to the tropane bridge.

A second general strategy was also developed and was used to access fused 3-D building blocks **1d**-**1e** and **1g**–**1i**. For fused 3-D building blocks **1g**, **1h**, and **1i**, an under-developed *exo*-selective diastereoselective debromination^50^ of accessible *gem*-dibromocyclopropanes followed by lithium–halogen exchange and *i*-PrOBpin trapping was utilized. The synthesis of **1g** via this approach is shown in Figure 2C. Dibromocyclopropanation of *N*-Boc tetrahydropyridine **8** with NaOH and CHBr_3_ in CH_2_Cl_2_–water in the presence of a phase transfer catalyst (BnEt_3_N^+^Cl^–^)^51^ gave *gem*-dibromocyclopropane **9** in 75% yield. Monodebromination was achieved by treating **9** with dimethylphosphite and KO*t*-Bu in DMSO at 100 °C, a procedure reported by Meijs and Doyle in 1985.^50^ This gave *exo*-monobromocyclopropane **10** in 70% yield (configuration assigned from the typical ^3^ *J* values for *cis* and *trans* couplings in cyclopropanes^52^), with the stereoselectivity proposed to arise from protonation of a monobromocyclopropane carbanion intermediate to place the bromide in the less sterically hindered *exo* position.^50^ Next, pinacol boronate **11** (86% yield) was formed via the stereospecific retentive lithium–halogen exchange of **10** using *n*-BuLi and trapping with *i*-PrOBpin. Treatment of **11** with MIDA gave 3-D building block **1g** (3.5 g prepared in one batch, configuration assigned from ^3^ *J* values) in 80% yield. In a similar way, fused 3-D building blocks **1h** (3.0 g) and **1i** (3.9 g) were prepared, and the configuration of each was assigned using X-ray crystallography.^53^ The same route was also used to synthesize diastereomeric spirocyclic pyrrolidines **1d** (1.1 g) and **1e** (2.9 g). In this case, due to similar steric hindrance on each side of the cyclopropane ring, the monodebromination reaction lacked diastereoselectivity: a 55:45 mixture of diastereomeric monobromides was formed. The configurations of **1d** and **1e** were assigned based on the X-ray crystal structure of the monobromocyclopropane that was an intermediate in the synthesis of **1e** (see Supporting Information for full details). To facilitate the use of 3-D building blocks **1a**-**i**, all nine building blocks are commercially available.

### Elaboration of 3-D Building Blocks

With the nine bifunctional 3-D building blocks **1a**-**i** in hand, the next stage involved demonstrating that both functional groups could be utilized in the planned elaboration methodology. Initial focus was on the development of general conditions for the Suzuki-Miyaura cross-coupling with aryl and heteroaryl bromides (Figure 3). Cross-coupling of 2,3-pyrrolidine cyclopropane BMIDA **1a** with 4-bromoanisole using Burke’s conditions^35^ (5 mol % Pd­(OAc)_2_/10 mol % SPhos and K_3_PO_4_ in dioxane/H_2_O) gave a 50:50 mixture of aryl cyclopropane **12** and unsubstituted cyclopropane **13** (protodeboronation product), with only an 18% isolated yield of pure **12**. Use of 7 mol % Pd­(OAc)_2_/13 mol % RuPhos and K_2_CO_3_ in toluene/H_2_O at 100 °C^36^ also gave a 50:50 mixture of **12** and **13**, but with an improved 48% yield of **12**. In contrast, use of 15 mol % Pd­(OAc)_2_/30 mol % PCy_3_ and 6 eq. Cs_2_CO_3_ in toluene/H_2_O at 100 °C (conditions A)^34^ gave only **12**, isolated in 77% yield (Figure 3A). Attempts to reduce the catalyst loading for reactions of BMIDA **1a** led to significant formation of **13**, presumably due to protodeboronation of the cyclopropyl boronic acid that is formed in situ. Using conditions A, cross-coupling of BMIDA **1a** with a range of 2-D fragment-like heteroaryl bromides, including pyrimidines, pyridines, *N-*tosyl 7-azaindole, and a *N*-tosyl indazole, gave aryl cyclopropanes **14**-**21** in 69–85% yield (Figure 3A). Access to enantiopure cross-coupled products was demonstrated using 2-bromoanisole; readily separable enantiopure BBIDA diastereomeric building blocks **1a’** and **1a’’** were converted into enantiomeric aryl cyclopropanes (*S*,*R*)-**14** (69%) (known configuration)^44^ and (*R*,*S*)-**14** (61%), respectively (Figure 3A).

**Figure 3:**
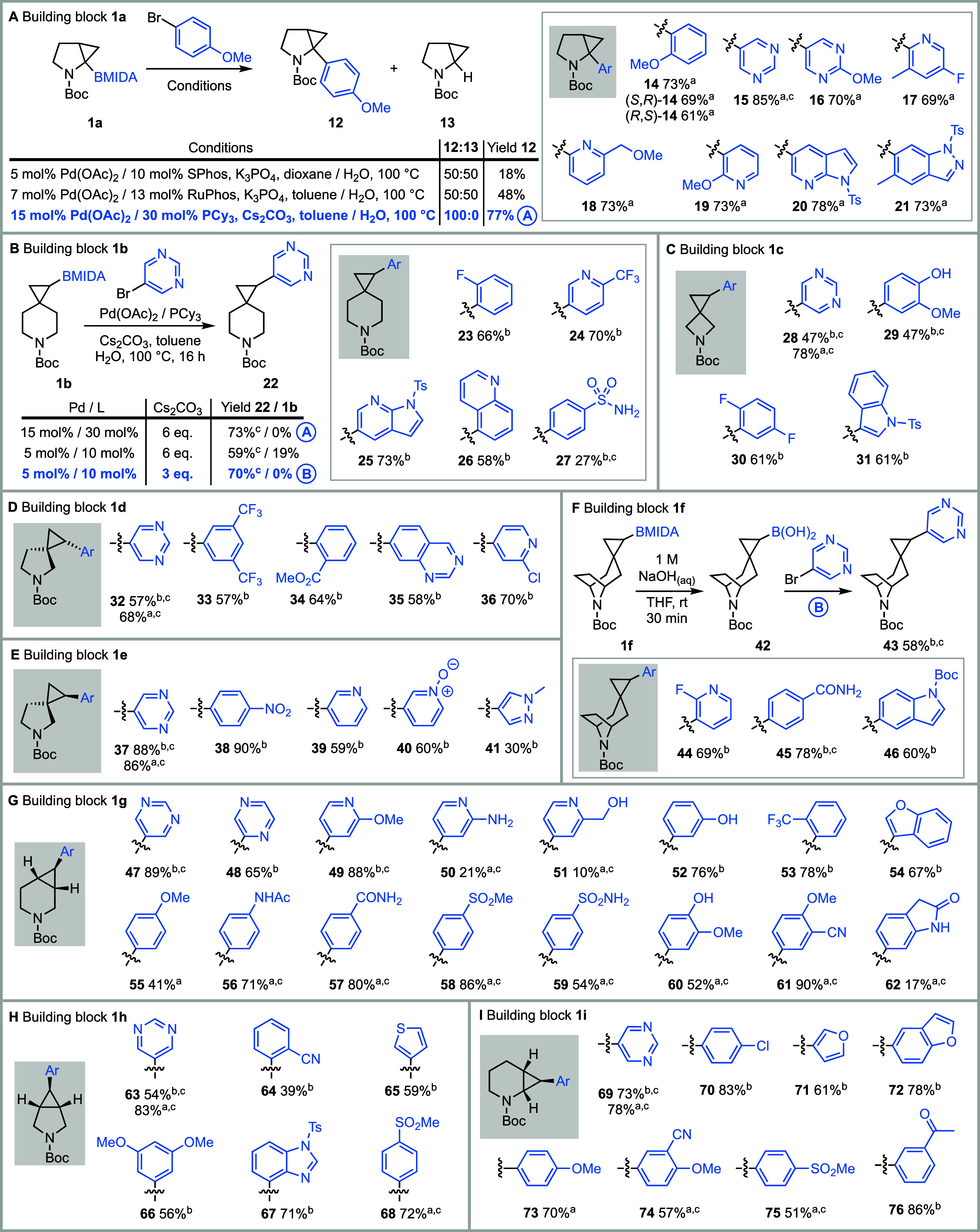
Optimization and scope of the Suzuki-Miyaura cross-coupling of *N*-Boc MIDA boronate building blocks **1a**-**i**. (A) **1a**. (B) **1b**. (C) **1c**. (D) **1d**. (E) **1e**. (F) **1f**. (G) **1g**. (H) **1h**. (I) **1i**. ^a^Using conditions A: 15 mol% Pd­(OAc)_2_, 30 mol% PCy_3_, Cs_2_CO_3_ (6 eq.), ArBr (1.4 eq.), toluene/H_2_O, 100 °C, 18 h. ^b^Using conditions B: 5 mol% Pd­(OAc)_2_,10 mol% PCy_3_, Cs_2_CO_3_ (3eq.), ArBr (1.4 eq.), toluene/H_2_O, 100 °C, 18 h. ^c^ArBr is a FragLite.

Conditions A were also applied to the cross-coupling between spiro piperidine BMIDA **1b** and 5-bromopyrimidine, which gave aryl cyclopropane **22** in 73% yield (Figure 3B). In this case, lowering the catalyst loading to 5 mol % Pd­(OAc)_2_/10 mol % PCy_3_ was accommodated, with **22** being obtained in 59% yield, together with unhydrolyzed BMIDA **1b** (19%). Reducing the amount of Cs_2_CO_3_ from 6 eq. to 3 eq. at this lower catalyst loading gave a 70% yield of aryl cyclopropane **22**. The majority of the cross-coupling examples were carried out under these conditions, namely, 5 mol % Pd­(OAc)_2_/10 mol % PCy_3_ and 3 eq. Cs_2_CO_3_ in toluene/H_2_O at 100 °C (conditions B). Issues were encountered in the attempted cross-couplings using cyclopropyl tropane BMIDA **1f**, as a low yield (29%, conditions B) of **43** was obtained. This was believed to be due to BMIDA **1f** not being fully soluble in toluene/water. Other solvents were explored without success, and use of the analogous Bpin derivative of **1f** gave **43** in 39% yield. As a result, ex situ hydrolysis of the MIDA boronate group of **1f** to the presumed boronic acid **42** was performed with NaOH_(aq)_ prior to cross-coupling.^47^ Then, cross-coupling of crude boronic acid **42** using conditions B gave aryl cyclopropane **43** in 58% yield. Similarly, using this two-step approach, **44**-**46** were obtained in 60–78% yield (Figure 3F).

Using conditions A and B, 3-D building blocks **1a-i** were cross-coupled with a wide range of aryl and heteroaryl bromides, with retention of configuration where relevant, as shown by X-ray crystallography of pyrimidine methanesulfonamides (vide infra, see Figure 4). Figure 3 illustrates 63 examples; yields ranged from 10 to 90%, and most were ≥ 60%. Electron-poor (**23**, **30**, **33**, **38**, **53**, **58**, **64**, **68**, **75**, **76**) and electron-rich (**12**, **14**, **29**, **52**, **55**, **56**, **66**, **73**) aryl bromides were well tolerated, as was a range of heteroaryl groups, including azaindole (**20**, **25**), indazole (**21**), quinoline (**26**), indole (**31**, **46**), quinazoline (**35**), pyrazole (**41**), benzofuran (**54**, **72**), thiophene (**65**), benzimidazole (**67**), and furan (**71**). Given the usefulness of the FragLite screening set in mapping out protein binding sites^31,32^ and their challenging functionality for cross-couplings, 11 distinct FragLites were included in our study of scope (examples with FragLites include **15**, **22**, **27–29**, **32**, **37**, **43**, **45**, **47**, **49–51**, **56–62**, **63**, **68**, **69**, **74–75**). There were some unsuccessful cross-coupling reactions, and these are presented in the Supporting Information. In addition, potassium trifluoroborate derivatives analogous to cyclopropyl BMIDAs **1b**, **1c**, **1f**, and **1g** were readily prepared and cross-coupled successfully (see Supporting Information for full details). In summary, all nine 3-D building blocks **1a-i** were successfully cross-coupled with 46 different aryl and heteroaryl bromides to generate 63 *N*-Boc aryl cyclopropanes (Figure 3).

**Figure 4:**
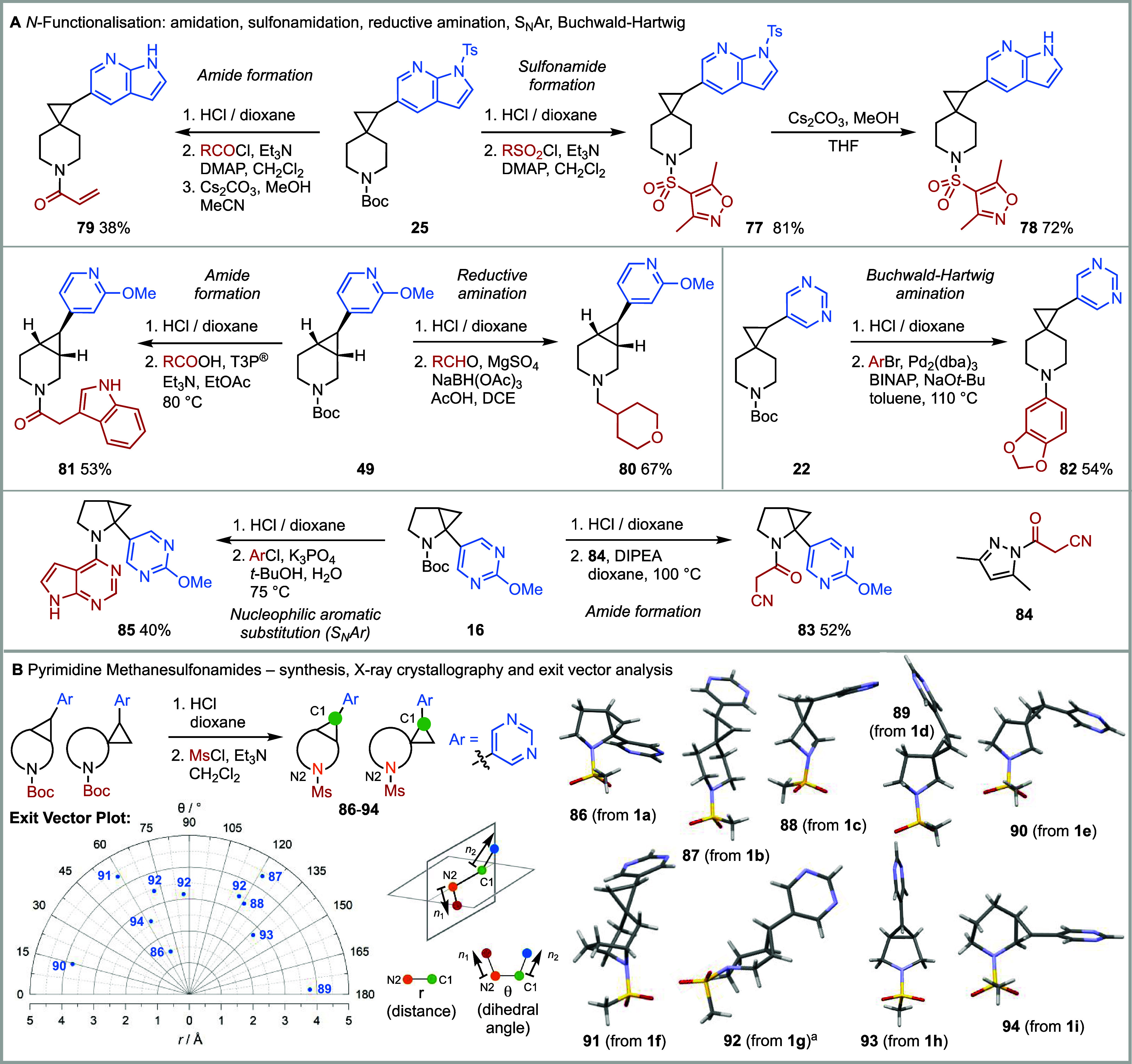
Exemplar diversification of aryl cyclopropanes with medicinally relevant *N*-capping groups and exit vector plot for pyrimidine sulfonamides **86–94**. (A) *N*-functionalization. (B) Synthesis, X-ray crystallography, and exit vector plot for **86–94** (where applicable, only one enantiomeric series for each compound is displayed on the exit vector plot). ^a^The X-ray crystal structure of **92** showed three different conformations but only one structure is shown here (Supporting Information for all three structures). The three conformations for **92** are shown on the exit vector plot.

Next, to validate that the nine 3-D building blocks **1a-i** would be suitable for fragment elaboration, we explored deprotection and diversification of the amine functionality of a selection of the *N*-Boc aryl cyclopropanes with a range of chemistries and capping groups commonly employed by medicinal chemists (Figure 4A).^26^ The Boc group was removed from aryl cyclopropanes **16**, **22**, **25**, and **49** using HCl/dioxane to give crude hydrochloride salts for subsequent reactions. Starting from azaindolyl spiro piperidine **25**, reaction with a sulfonyl chloride gave sulfonamide **77**, and subsequent treatment with Cs_2_CO_3_/MeOH^54^ removed the *N*-tosyl group to give azaindole sulfonamide **78**. In a similar way, **25** was converted into acrylamide **79** (using acryloyl chloride); acrylamides are commonly employed as covalent warheads, and this approach is gaining significant prominence in medicinal chemistry.^55^ Methoxy pyridine-containing fused piperidine **49** was derivatized in two different ways: reductive amination delivered *N*-alkyl derivative **80** and amidation using T3P and a carboxylic acid gave amide **81**. Pyrimidinyl spiro piperidine **22** was *N-*arylated using a Buchwald-Hartwig reaction to give *N-*aryl piperidine **82**. Another covalent warhead was added to methoxypyrimidinyl-fused pyrrolidine **16**, via amidation with acyl pyrazole **84**, to give amide **83**. Alternatively, starting from **22**, a S_N_Ar reaction with 4-chloro-7H-pyrrolo­[2,3-*d*]­pyrimidine led to *N*-aryl pyrrolidine **85**.

A key design feature of 3-D building blocks **1a-i** was that they would access a range of 3-D vectors and 3-D chemical space. To show this, each of the building blocks **1a-i** was cross-coupled with 5-bromopyrimidine (see Figure 3) and subsequently converted into the corresponding methanesulfonamides **86–94** (Figure 4B). As anticipated, sulfonamides **86–94** were crystalline, and each was analyzed by X-ray crystallography. The X-ray crystal structures and the exit vector plot of *r* (distance) vs θ (dihedral angle)^41^ for sulfonamides **86–94** are shown in Figure 4B. For fused piperidine **92**, three conformations (only one shown) were found in the asymmetric unit in the X-ray crystal structure, and all three conformations are presented on the exit vector plot of *r* vs θ. Of note, the *r* vs θ plot shows that sulfonamides **86–94**, derived from building blocks **1a-i**, respectively, cover a wide and varied range of chemical space, with distances between variation points (*r*) of 1.5–4.4 Å and a range of spatial orientations of the diversification groups (θ).

For the synthesis of 3-D lead-like compounds from putative 2-D fragments and 3-D building blocks **1a-i**, the 16 examples of *N*-functionalization shown in Figure 4A,B were supplemented by 16 further examples (see Supporting Information) to give 32 3-D lead-like compounds. The lead-like nature of the compounds was confirmed by the analysis of their calculated molecular properties. Under Churcher’s definition,^11^ compounds occupy lead-like chemical space if they have suitable lipophilicity (−1 < clogP < 3) and molecular weight (200–350 Da), with a low degree of aromatic character. The 32 exemplar compounds have a mean clogP of 0.46, a mean MW of 266, and a mean fraction of sp^3^ hybridized carbon atoms (Fsp^3^)^10^ of 0.56, and thus comfortably occupy lead-like space (29 of the 32 compounds satisfy these lead-like criteria; values for each lead-like compound are provided in the Supporting Information). In addition, 12 of the lead-like compounds (**78**, **81**, **82**, and **86–94**) were subjected to AstraZeneca’s drug metabolism and pharmacokinetics (DMPK) Wave1 analysis.^56^ This provides information on lipophilicity (measured logD), aqueous solubility, and metabolic stability in human liver microsomes (HLM) and rat hepatocytes (RH) (see Supporting Information for full details). For the 12 compounds, logD ranged from −0.4 to 3.6, and all exhibited suitable aqueous solubility (49.0 to >981 μM). Ten of the 12 compounds (**82** and all sulfonamides **86–94**) showed good metabolic stability in both assays; compounds **78** and **81**, which had the highest logD values, have benzylic positions and/or an electron-rich indole ring which likely accounts for their lower metabolic stability. This also shows that the metabolic profile will be dependent on the overall properties of the molecules designed using 3-D building blocks **1a**-**i** and that there are no intrinsic liabilities with the cyclopropyl building block scaffolds.

### Design and Synthesis of a Selective JAK3 Inhibitor

Having demonstrated the synthesis phase (see Figure 1B) of the fragment elaboration synthetic platform, the final element was to showcase our approach with the design and synthesis of a 3-D lead compound using the lead design phase (see Figure 1A). For this, the development of a Janus kinase (JAK) inhibitor related to Ritlecitinib was targeted. The Janus kinases are a family of nonreceptor tyrosine kinases (TYK), which control cytokine signaling, and are common targets for the modulation of autoimmune, autoinflammatory, and allergic diseases. Ritlecitinib (PF-06651600) (Figure 5A), a covalent inhibitor of JAK3 that shows selectivity over the other JAK isoforms JAK1, JAK2, and TYK2,^57^ was approved in 2023 for the treatment of alopecia areata. The X-ray crystal structure of the JAK3 covalent adduct formed from Cys909 reacting with the acrylamide of Ritlecitinib is shown in Figure 5A. In addition to this covalent bond, the pyrrolopyrimidine was hydrogen bonded with Glu903 and Leu905 in JAK3. In addition, a key conformational activation of the acrylamide was identified from water-linked hydrogen bonds from the pyrimidine to the carbonyl group of the acrylamide.

**Figure 5:**
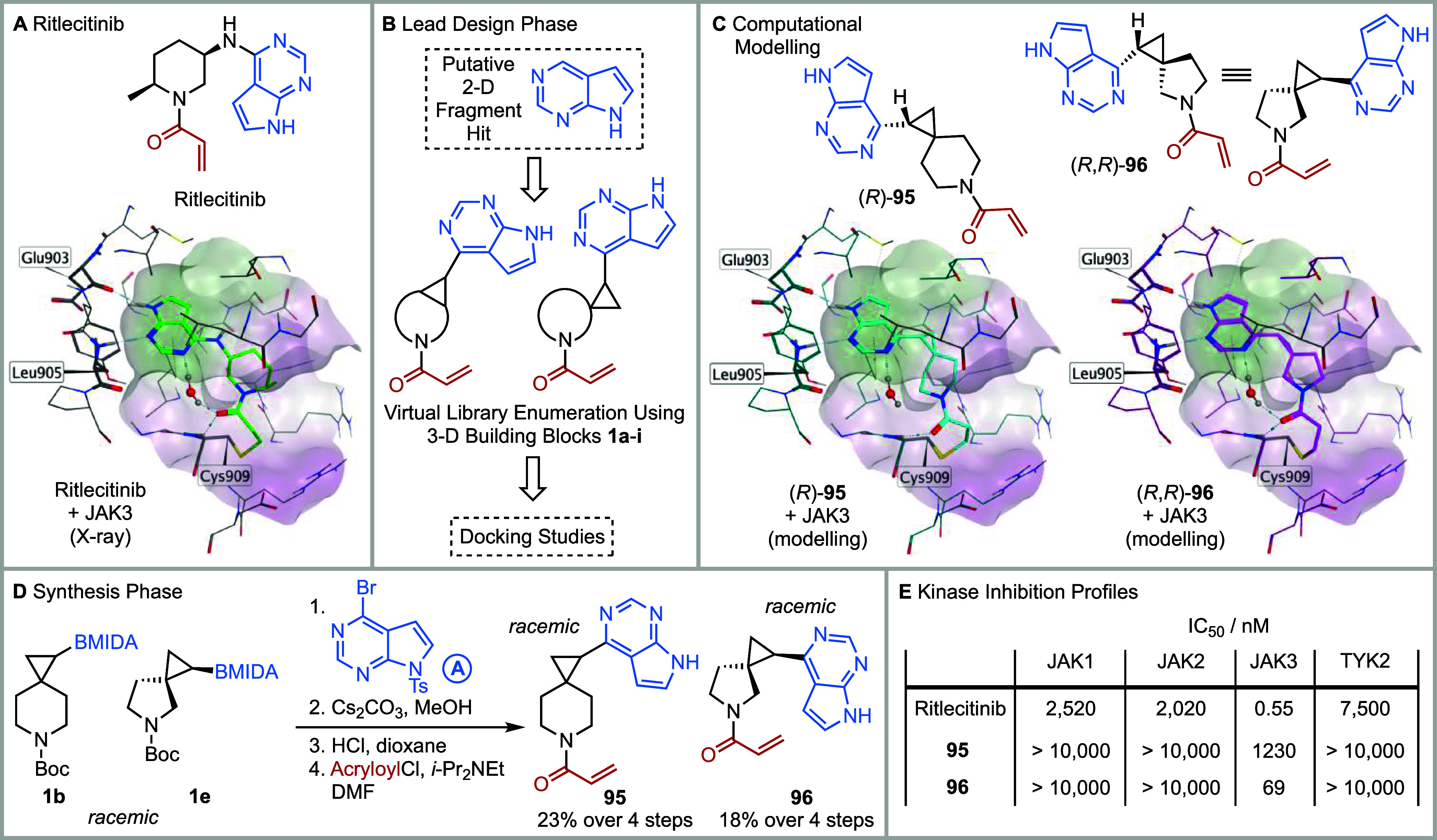
Design and synthesis of a JAK3 selective inhibitor. (A) Cocrystal X-ray structure of ritlecitinib and JAK3. (B) Lead design phase. (C) Computational modeling of (*R*)-**95** and (*R*,*R*)-**96** and JAK3. (D) Synthesis of racemic **95** and **96**. (E) Kinase inhibition profiles of ritlecitinib, **95**, and **96** against JAK1, JAK2, JAK3, and TYK2 (see Supporting Information for details on the concentrations of reagents used in the assay).

For our fragment elaboration docking studies, we first imagined that the pyrrolopyrimidine was an initial 2-D fragment hit that had bound in the kinase hinge region of JAK3 in the same pose as Ritlecitinib. Nine potential lead-like compounds were then virtually enumerated by combining the pyrrolopyrimidine (from the 4-position) with each of the nine 3-D building blocks **1a-i** which were *N*-functionalized with an acrylamide (Figure 5B). Computational modeling of each enantiomer of these lead-like compounds (except for that derived from **1h** which is meso) was carried out by docking the pyrrolopyrimidine to Glu903 and Leu905 with and without a covalent bond to the acrylamide. The poses with the lowest docking scores/energies and similar conformational features to the Ritlecitinib–JAK3 complex were identified (see Supporting Information for details). The modeled complexes for **95** and **96** bound to JAK3 are shown in Figure 5C. From this analysis, acrylamides **95** and **96** were targeted for synthesis. Of note, **95** and **96** (derived from 3-D building blocks **1b** and **1e**) were readily synthesized since the synthetic methodology to do so (Suzuki-Miyaura cross-coupling, tosyl and Boc group removal and acrylamide formation, Figure 5D) was already established, thus highlighting that building blocks **1a-i** are synthetically enabled. Acrylamides **95** and **96** were evaluated in a biochemical kinase inhibition assay, screening against JAK1/2/3 and TYK2 at a 1 h time point.^58^ Both **95** and **96** were selective for JAK3, with no inhibition (>10,000 nM) of JAK1, JAK2, or TYK2 (Figure 5E). Pleasingly, **96** was a potent inhibitor of JAK3, with IC_50_ = 69 nM, with **95** being less potent, and with IC_50_ = 1.23 μM (see Supporting Information). Although **96** was ∼10-fold less active than Ritlecitinib (IC_50_ = 0.55 nM under the same assay conditions, where the ATP concentration is close to K_m_), **96** has a better selectivity profile against JAK1, JAK2, and TYK2 compared to Ritlecitinib (Figure 5E). In addition, racemic **95** and **96** were evaluated, whereas Ritlecitinib is a single enantiomer. The better inhibition results for **96** compared to **95** may be due to the fully formed water-linked hydrogen bonds from the pyrimidine to the carbonyl group of the acrylamide in the computational modeling of the docking pose (Figure 5C). Of note, the glutathione (GSH) reactivity of both **96** and Ritlecitinib were similar: *t*_1/2_ for **96** is 1254 min and *t*_1/2_ for Ritlecitinib is 2020 min in a comparative assay^59^ (see Supporting Information). Thus, using both the lead design and synthesis phases, we have demonstrated elaboration of the putative 2-D fragment hit, pyrrolopyrimidine, using 3-D building block **1e** (identified from the computational docking studies) into 3-D lead-like compound **96** which is a selective inhibitor of JAK3, with IC_50_ = 69 nM. Our approach is complementary to that reported by Reymond et al. for the discovery of a JAK1 selective inhibitor and a JAK3 selective inhibitor, each based on a novel triquinazine scaffold.^60^

## Conclusions

In conclusion, we have developed a modular synthetic platform for the systematic and programmable elaboration of 2-D fragment hits into 3-D lead-like compounds for use in FBDD. The design and gram-scale synthesis of nine bifunctional 3-D building blocks **1a-i**, together with a wide range of Suzuki-Miyaura cross-coupling reactions (65 examples) and *N*-functionalizations (32 lead-like compounds), are presented. Crucially, each of the 3-D building blocks **1a-i** accesses a distinct 3-D exit vector, as shown both by analysis of the lowest energy conformations of potential lead-like molecules using RDKit (Figure 1D) and by X-ray crystallography of methanesulfonamides (Figure 4B). The design and synthesis of a selective inhibitor of JAK3 with IC_50_ = 69 nM showcased the synthetic platform, with the rapid generation of lead-like compounds from an initial 2-D fragment hit. In this way, our methodology is a step toward addressing the call to arms from industry by providing the synthetic “elaboration of fragments in three dimensions from many different growth points/vectors using methodology that is worked out prior to fragment screening.”^14^ Of note, 3-D building blocks **1a-i** are commercially available. Future efforts will be directed toward the development of additional bifunctional 3-D building blocks that provide entry into 3-D exit vectors that are not covered by 3-D building blocks **1a-i**. In this way, the synthetic platform will be expanded to provide a comprehensive coverage of 3-D vector and chemical space for use in FBDD.

## Supplementary Material



## Notes

Experimental procedures and characterization data; exit vector analysis; lead-like analysis; DMPK analysis; and molecular modeling and inhibition studies (PDF)

## Abbreviations

FBDD: fragment-based drug discovery
MW: molecular weight
3-D: 3-dimensional
2-D: 2-dimensional
Boc: *tert*-butoxy carbonyl
S_N_Ar: nucleophilic aromatic substitution
MIDA: *N-*methyliminodiacetic acid
BMIDA: *N-*methyliminodiacetic acid boronate
JAK3: Janus kinase 3
TMEDA: *N,N,N’,N’*-tetramethylethylene-diamine
DMSO: dimethyl sulfoxide
BIDA: *N*-2-benzyloxycyclopentyl-iminodiacetic acid
pin: piacolate
SPhos: 2-dicyclohexylphosphino-2′,6′-dimethoxybiphenyl
RuPhos: 2-dicyclohexylphosphino-2′,6′-diisopropoxybiphenyl
DMPK: drug metabolism and pharmacokinetics
TYK: nonreceptor tyrosine kinase
