# Supplementary material for: Modular Synthetic Platform for the Elaboration of Fragments in Three Dimensions for Fragment-Based Drug Discovery
Source: J Am Chem Soc. 2025 Jul 30;147(32):29292–303. doi: 10.1021/jacs.5c08786 (PMC12356528; doi:10.1021/jacs.5c08786)
Supplement: Supplementary file 1 [file ja5c08786_si_001.pdf]

Modular Synthetic Platform for the Elaboration of Fragments in Three Dimensions for Fragment-Based Drug Discovery

Andres R. Gomez-Angel,[a,†] Hanna F. Klein,[a†] Stephen Y. Yao,[a†], James R. Donald,[a] James D. Firth,[a] Rebecca Appiani,[a] Cameron J. Palmer,[a] Joshua Lincoln,[a] Simon C. C. Lucas,[b] Lucia Fusani,[b] R. Ian Storer[b] and Peter O'Brien[a,*]

[†] These authors contributed equally to this work.

[a] Department of Chemistry, University of York, Heslington, York YO10 5DD, U.K.

[b] Hit Discovery, Discovery Sciences, R&D, AstraZeneca, 1 Francis Crick Ave, Cambridge, CB2 0AA, U.K.

peter.obrien@york.ac.uk

The data supporting this paper can be found at:

https://doi.org/10.15124/91115136-fd90-43ce-93f9-ab678a706060

The nine 3-D building blocks **1a**-**1i** are commercially available from Key Organics.

Contents

1. Experimental Details S3

1.1. General S3

1.2. General Procedures S4

1.3. Experimental Procedures and Characterisation S6

1.4. Synthesis of Potassium Trifluoroborates S158

1.5. Failed Suzuki-Miyaura Cross-Coupling Reactions S158

2. Compound Analysis S159

2.1. Exit Vector Analysis S159

2.2. Lead-like Analysis S161

2.3. AstraZeneca's drug metabolism and pharmacokinetics (DMPK) Wave1 analysis S163

3. Molecular Modelling, DMPK Analysis and Inhibition Studies of JAK3 Inhibitors S164

4. ^{1}H and ^{13}C NMR Spectra S172

5. References S342

1. Experimental Details

1.1. General

All-non aqueous reactions were carried out under oxygen free Ar or N_2 using flame-dried glassware. Toluene, Et_2O, and THF were dried using Innovative Technologies solvent purification system using activated alumina. Alkyllithiums were titrated against *N*-benzylbenzamide before use. Et_3N and pyrrolidine were distilled over CaH_2 before use. Brine refers to a saturated solution. Water is distilled water.

Flash column chromatography was carried out using Supelco silica (220-440 mesh). Thin layer chromatography was carried out using commercially available Merck F_{254} aluminium backed silica plates. Proton (400 MHz) and carbon (100.6 MHz) NMR spectra were recorded on a Jeol ECX-400 instrument using an internal deuterium lock. For samples recorded in $CDCl_3$, chemical shifts are quoted in parts per million relative to $CHCl_3$ (δ_H 7.26) and $CDCl_3$ (δ_C 77.0, central line of triplet). For samples recorded in d_6-DMSO, chemical shifts are quoted in parts per million relative to DMSO (δH 2.50, central line of quintet) and d_6-DMSO (δ_C 39.5, central line of septet). For samples recorded in d_6-acetone, chemical shifts are quoted in parts per million relative to acetone (δ_H 2.05, central line of quintet) and d_6-acetone (δ_C 29.8, central line of septet). Carbon NMR spectra were recorded with broad band proton decoupling and assigned using DEPT experiments. Coupling constants (*J*) are quoted in Hertz. Melting points were carried out on a Gallenkamp melting point apparatus. Infrared spectra were recorded on a Perkin Elmer UATR Two FT-IR spectrometer. Electrospray high and low resonance mass spectra were recorded at room temperature on a Bruker Daltronics microOTOF spectrometer.

1.2. General Procedures

General Procedure A: Suzuki-Miyaura cross-coupling of cyclopropyl MIDA boronates using Conditions A

A solution of $Pd(OAc)_2$ (8 mg, 0.04 mmol, 0.15 eq) and PCy_3 (21 mg, 0.08 mmol, 0.30 eq), Cs_2CO_3 (489 mg, 1.5 mmol, 6.0 eq) and the aryl bromide (0.35 mmol, 1.4 eq.) was added to MIDA boronate (0.25 mmol, 1.0 eq) in toluene (5.0 mL) at rt in a sealed tube under Ar. H_2O (0.5 mL) was added and the suspension was degassed by sparging with Ar for 20 min. The resulting mixture was stirred and heated at 100 °C in a sealed tube for 18 h under Ar. The mixture was then allowed to cool to rt and EtOAc (15 mL) and water (15 mL). The two layers were separated and the aqueous layer was extracted with EtOAc (3 × 15 mL). The combined organic layers were washed with brine (30 mL), dried ($MgSO_4$) and evaporated under reduced pressure to give the crude product.

General Procedure B: Suzuki-Miyaura cross-coupling of cyclopropyl MIDA boronates using Conditions B

A solution of $Pd(OAc)_2$ (3 mg, 0.01 mmol, 0.05 eq) and PCy_3 (7 mg, 0.02 mmol, 0.10 eq), Cs_2CO_3 (244 mg, 0.75 mmol, 3.0 eq) and the aryl bromide (0.35 mmol, 1.4 eq.) was added to MIDA boronate (0.25 mmol, 1.0 eq) in toluene (5.0 mL) at rt in a sealed tube under Ar. H_2O (0.5 mL) was added and the suspension was degassed by sparging with Ar for 20 min. The resulting mixture was stirred and heated at 100 °C in a sealed tube for 18 h under Ar. The mixture was then allowed to cool to rt and EtOAc (15 mL) and water (15 mL). The two layers were separated and the aqueous layer was extracted with EtOAc (3 × 15 mL). The combined organic layers were washed with brine (30 mL), dried ($MgSO_4$) and evaporated under reduced pressure to give the crude product.

General Procedure C: Suzuki-Miyaura Cross-Coupling of Cyclopropyl MIDA Boronate 1f *via ex situ* hydrolysis

1 M $NaOH_{(aq)}$ (1 mL) was added to a solution of cyclopropyl MIDA boronate (0.25 mmol, 1.0 eq.) in THF (1 mL) at rt and the resulting mixture was stirred at rt for 30 min. Saturated $NH_4Cl_{(aq)}$ (1 mL) and MTBE (5 mL) were added and the two layers were separated. The aqueous layer was extracted with MTBE (3 × 5 mL). The combined organic layers were washed with brine (10 mL), dried ($MgSO_4$) and evaporated under reduced pressure to a volume of ~5 mL. Toluene (10 mL) was added and the solvent was evaporated under reduced pressure to a volume of ~2 mL. The resulting solution was transferred to a sealed tube. Then, Cs_2CO_3 (244 mg, 0.75 mmol, 3.0 eq.) and the aryl bromide (0.35 mmol, 1.4 eq.)

were added and the sealed tube was evacuated and backfilled with Ar three times. A solution of $Pd(OAc)_2$ (2.81 mg, 0.01 mmol, 0.05 eq.) and PCy_3 (7.01 mg, 0.02 mmol, 0.10 eq.) in toluene (2.8 mL) was added. Then, H_2O (0.5 mL), degassed by sparging with Ar for 30 min, was added and the resulting mixture was stirred and heated at 100 °C in a sealed tube for 18 h under Ar. The mixture was then allowed to cool to rt and EtOAc (15 mL) and water (15 mL). The two layers were separated and the aqueous layer was extracted with EtOAc (3 × 15 mL). The combined organic layers were washed with brine (30 mL), dried ($MgSO_4$) and evaporated under reduced pressure to give the crude product.

General Procedure D: Synthesis of Methanesulfonamides

HCl (4 M solution in dioxane) was added dropwise to a stirred solution of cyclopropyl-pyrimidine (1.0 eq.) at rt under Ar. The resulting solution was stirred at rt for 1 h. Then, the solvent was evaporated under reduced pressure to give the crude HCl salt. Et_3N (4.8 eq.) was added to a stirred solution the HCl salt in CH_2Cl_2 at rt under Ar. The resulting solution was stirred at rt for 10 min and MsCl (2.2 eq.) was added. The resulting solution was stirred at rt for 16 h. The mixture was poured into $NaHCO_{3(aq)}$ (20 mL) and extracted with CH_2Cl_2 (3 × 20 mL). The combined organics were dried (Na_2SO_4) and evaporated under reduced pressure to give the crude product.

1.3. Experimental Procedures and Characterisation

tert-Butyl 4-chloropiperidine-1-carboxylate 3

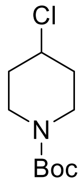

3

NCS (6.63 g, 49.7 mmol, 1.0 eq.) was added to a solution of *N*-Boc-4-hydroxypiperidine (10.0 g, 49.7 mmol, 1.0 eq.) and PPh_3 (13.0 g, 49.6 mmol, 1.0 eq.) in CH_2Cl_2 (200 mL) at 0 °C under Ar. The resulting solution was stirred at rt for 16 h. The solvent was evaporated under reduced pressure, Et_2O was added (200 mL) and the solids were removed by filtration, washing with Et_2O (100 mL). The filtrate was evaporated under reduced pressure and the Et_2O addition/filtration/washing/concentration procedure was repeated three times to give the crude product as a yellow oil. Purification by flash column chromatography on silica with 97:3 hexane-THF and then 96:4 hexane-THF as eluent gave *tert*-butyl 4-chloropiperidine-1-carboxylate **3** (7.97 g, 73%) as a very pale yellow oil, R_F (96:4 hexane-THF) 0.11; IR (ATR) 2976, 2250, 1682 (C=O), 1163, 727 cm^{-1}; ^{1}H NMR (400 MHz, $CDCl_3$) δ 4.12 (tt, *J* = 7.5, 3.5 Hz, 1H, CHCl), 3.62 (ddd, *J* = 13.5, 7.5, 7.0 Hz, 2H, NCH), 3.21 (ddd, *J* = 13.5, 7.5, 3.5 Hz, 2H, NCH), 1.94 (dddd, *J* = 13.5, 7.0, 3.5, 3.5 Hz, 2H, CH), 1.70 (dddd, *J* = 13.5, 7.5, 7.5, 3.6 Hz, 2H, CH), 1.37 (s, 9H, CMe_3); ^{13}C NMR (100.6 MHz, $CDCl_3$) δ 154.6 (C=O), 79.7 (O*C*Me_3), 56.9 (CHCl), 41.3 (NCH_2), 34.6 (CH_2), 28.4 (C*Me*$_3$); MS (ESI) *m/z* 242 [(M(^{35}Cl) + Na)$^+$]; HRMS *m/z* calcd for $C_{10}H_{18}{}^{35}ClNO_2$ (M + Na)$^+$ 242.0918, found 242.0916 (+0.8 ppm error). Spectroscopic data consistent with those reported in the literature.[1]

Lab book reference JRD_VIII_4

tert-Butyl 1-(6-methyl-4,8-dioxo-1,3,6,2-dioxazaborocan-2-yl)-2-azabicyclo[3.1.0]hexane-2-carboxylate 1a

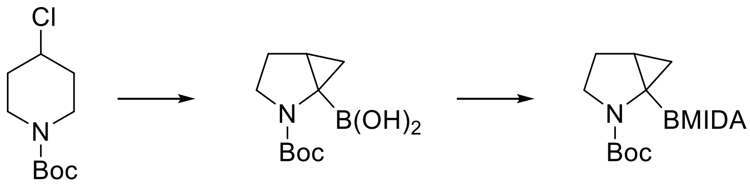

3 **4** **1a**

s-BuLi (59.7 mL of a 1.27 M solution in cyclohexane, 75.9 mmol, 2.2 eq.) was added dropwise to a stirred solution of *tert*-butyl 4-chloropiperidine-1-carboxylate **3** (7.58 g, 34.5 mmol, 1.0 eq.) and TMEDA (8.81 g, 11.4 mL, 75.9 mmol, 2.2 eq.) in Et_2O (230 mL) at –78 °C under Ar. The resulting solution was stirred at –78 °C for 1 h. Then, trimethyl borate (7.88 g, 8.46 mL, 75.9 mmol, 2.2 eq.) was added dropwise. After stirring at –78 °C for 30 min, the mixture was removed from the cooling bath and stirred at rt for 30 min. The reaction was cooled to 0 °C, 5% $HCl_{(aq)}$ (150 mL) was added, the layers were separated and the aqueous layer was extracted with EtOAc (3 × 100 mL). The combined organic extracts were dried ($MgSO_4$) and evaporated under reduced pressure to give the crude product as a pale-yellow oil. Purification by flash column chromatography on silica with 19:1 CH_2Cl_2-acetone and then 19:1 CH_2Cl_2-MeOH as eluent gave *N*-Boc boronic acid **4** (6.30 g) as a pale yellow oil possibly containing some of the methyl boronic ester, R_F (19:1 CH_2Cl_2-acetone) 0.13; ^{1}H NMR (400 MHz, $CDCl_3$) (60:40 mixture of rotamers) δ 9.57 (br s, 0.5H, OH), 3.75–3.37 (m, 4H, NCH and OCH_3), 3.29–2.66 (m, 1H, NCH), 2.29–1.84 (m, 2H, CH), 1.83–1.70 (m, 0.6H, CH), 1.64–1.30 (m, 9.4H, CMe_3, CH), 0.97–0.82 (m, 1H, CH), 0.72–0.65 (m, 0.6H, C*H*), 0.61–0.48 (m, 0.3H, CH).

Lab book reference JRD_VIII_12

Methyliminodiacetic acid (MIDA) (3.76 g, 25.6 mmol, 1.1 eq.) was added to a stirred solution of *N*-Boc boronic acid **4** (5.28 g, 23.2 mmol, 1.0 eq.) in toluene (116 mL) and DMSO (116 mL) at rt under Ar in a 500 mL RBF fitted with a Dean-Stark apparatus. The resulting suspension was stirred and heated at 140 °C (bath temp) for 16 h. The mixture was allowed to cool to rt, diluted with H_2O (500 mL) and extracted with CH_2Cl_2 (3 × 300 mL). The combined organics were washed with H_2O (2 × 200 mL), dried ($MgSO_4$) and evaporated under reduced pressure to give the crude product as an off-white solid. Recrystallisation from boiling acetone gave *N*-Boc MIDA boronate **1a** (4.17 g, 43% over two steps) as colourless needles, mp 248–250 °C; R_F (8:2 CH_2Cl_2-acetone) 0.38; IR (ATR) 2979, 1779 (C=O, ester), 1732 (C=O, ester),

1682 (C=O, Boc), 1409 cm^{-1}; ^{1}H NMR (400 MHz, acetone-*d$_6$*) δ 4.47 (d, *J* = 16.0 Hz, 1H, MeNC*H*), 4.27 (d, *J* = 18.0 Hz, 1H, MeNC*H*), 4.04 (d, *J* = 18.0 Hz, 1H, MeNC*H*), 4.01 (d, *J* = 16.0 Hz, 1H, MeNC*H*), 3.60 (ddd, *J* = 11.5, 10.0, 5.5 Hz, 1H, NCH), 3.45 (s, 3H, NMe), 3.28 (ddd, *J* = 11.5, 8.5, 6.5 Hz, 1H, 1H, NCH), 2.14–2.07 (m, 1H, CH), 1.85 (ddd, 1H, *J* = 13.0, 8.5, 5.5 Hz, CH), 1.73–1.65 (m, 1H, CH), 1.39 (s, 9H, C(C*H*$_3$)$_3$), 1.00 (dd, *J* = 8.5, 5.0 Hz, 1H, CH), 0.73 (t, *J* = 5.0 Hz, 1H, CH); ^{13}C NMR (100.6 MHz, acetone-d$_6$) 169.5 (C=O, ester), 169.1 (C=O, ester), 156.9 (C=O, Boc), 79.5 (O*C*Me$_3$), 65.4 (MeN*C*H$_2$), 64.6 (MeN*C*H$_2$), 49.9 (NCH$_2$), 47.4 (NMe), 28.6 (C*Me$_3$*), 26.4 (CH$_2$), 24.9 (CH), 19.4 (CH$_2$), (NCB resonance not resolved); ^{11}B (128.4 MHz, acetone-d$_6$) 10.8; MS (ESI) *m/z* 361 [(M(^{11}B) + Na)$^+$]; HRMS *m/z* calcd for $C_{15}H_{23}BN_2O_6$ (M+ Na)$^+$ 361.1541, found 361.1544 (0.0 ppm error).

Lab book reference JRD_VIII_6, JRD_VIII_15, JRD_VIII_16

2-{[(1*S*,2*S*)-2-(Benzyloxy)cyclopentyl](carboxymethyl)amino}acetic acid S1

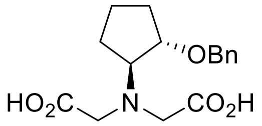

(*S*,*S*)-**S1**

A solution of *tert*-butyl bromoacetate (461 mg, 2.4 mmol, 2.4 eq.) in MeCN (1.5 mL) was added to a stirred mixture of K_2CO_3 (691 mg, 5.0 mmol, 5.0 eq.) and (1*S*,2*S*)-(+)-2-benzyloxycyclopentylamine (191 mg, 1.0 mmol, 1.0 eq.) in MeCN (10 mL) at 0 °C. The mixture was stirred and heated at 70 °C for 24 h. Then, the solids were removed by filtration through Celite® and washed with EtOAc (5 mL). The filtrate was evaporated under reduced pressure to give a viscous oil. Formic acid (1.01 mL, 26.7 mmol, 26.7 eq.) was added to the viscous oil and the resulting mixture was stirred and heated at 85 °C for 2 h. After being allowed to cool to rt, the solvent was evaporated under reduced pressure and the residue was dissolved in hot EtOH (4 mL). The solution was allowed to cool slowly to rt and then it was cooled to 0 °C. The precipitated solid was collected by filtration and dried under high vacuum to give di-acid **S1** (265 mg, 86%) as a white solid, mp 171-173 °C; IR (ATR) 1962, 1731 (C=O), 1630, 1356 cm^{-1}; ^{1}H NMR (400 MHz, DMSO-*d$_6$*) δ 7.31–7.26 (m, 4H, Ph), 7.26–7.19 (m, 1H, Ph), 4.41 (d, *J* = 11.5 Hz, 1H, OPh), 4.33 (d, *J* = 11.5 Hz, 1H, OPh), 3.79–3.66 (m, 1H, OCH), 3.48 (d, *J* = 17.5 Hz, 2H, NCH), 3.44 (d, *J* = 17.5 Hz, 2H, NCH), 3.26–3.13 (m, 1H, NCH), 1.83 (ddd, *J* = 12.5, 8.5, 5.5 Hz, 1H, CH), 1.74 (ddd, *J* = 11.5, 7.5, 5.0 Hz, 1H, CH), 1.60–1.44 (m, 3H, CH), 1.40–1.25 (m, 1H); ^{13}C NMR (100.6 MHz,

DMSO-d_6) δ 173.6 (C=O), 139.1 (*ipso*-Ph), 128.7 (Ph), 128.2 (Ph), 127.9 (Ph), 83.5 (OCH), 70.9 (OCH_2), 68.7 (NCH), 54.1 (NCH_2), 30.3 (CH_2), 29.0 (CH_2), 21.4 (CH_2); MS (ESI) *m/z* 237 [$(M + Na)^+$]; MS (ESI) *m/z* 330 [$(M + Na)^+$]; HRMS *m/z* calcd for $C_{16}H_{21}NO_5$ $(M + Na)^+$ 330.1312, found 330.1311 (+0.2 ppm error). Spectroscopic data consistent with those reported in the literature.[2]

Lab book reference RA 1-023

***tert*-Butyl (1*S*,5*R*)-1-{6-[(1*S*,2*S*)-2-(benzyloxy)cyclopentyl]-4,8-dioxo-1,3,6,2-dioxazaborocan-2-yl}-2-azabicyclo[3.1.0]hexane-2-carboxylate (*S*,*R*,*S*,*S*)-1a' and *tert*-butyl (1*R*,5*S*)-1-{6-[(1*S*,2*S*)-2-(benzyloxy)cyclopentyl]-4,8-dioxo-1,3,6,2-dioxazaborocan-2-yl}-2-azabicyclo[3.1.0]hexane-2-carboxylate (*S*,*R*,*S*,*S*)-1a''**

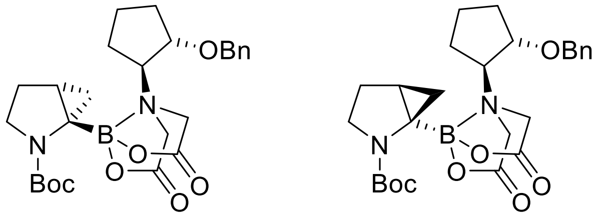

(*S*,*R*,*S*,*S*)-**1a'** (*R*,*S*,*S*,*S*)-**1a''**

N-Boc boronic acid **4** (114 mg, 0.5 mmol, 1.0 eq.) and BIDA **S1** (308 mg, 1.0 mmol, 2.0 eq.) were dissolved in DMF (5 mL) and stirred and heated at 85 °C under Ar for 16 h. The reaction mixture was allowed to cool to rt, diluted with H_2O (10 mL) and extracted with Et_2O (3 × 5 mL). The combined organics were dried ($MgSO_4$) and evaporated under reduced pressure to give the crude product as a white solid. Purification by flash column chromatography on silica with 8:2 hexane-acetone as eluent gave *N*-Boc BIDA boronate (*S,R,S,S*)-**1a'** (67 mg, 26%) as a white solid, mp 186-188 °C; R_F (7:3 hexane-acetone 7:3) 0.4; IR (ATR) 2974, 1764, 1684 (C=O), 1401 cm^{-1}; 1H NMR (400 MHz, $CDCl_3$) (60:40 mixture of rotamers) δ 7.40–7.23 (m, 5H, Ar), 5.45–5.30 (m, 1H, NCH), 5.12–5.03 (m, 1H, OCH), 4.69 (d, J = 12.0 Hz, 1H, NCH), 4.43 (d, J = 12.0 Hz, 1H, NCH), 3.71 (d, J = 17.0 Hz, 1H, OCH), 3.62 (ddd, J = 11.5, 9.5, 6.5 Hz, 1H, NCH), 3.54 (ddd, J = 11.5, 10.0, 2.5 Hz, 1H, NCH), 3.45–3.33 (m, 3H), 3.08–2.97 (m, 1H, CH), 2.35–2.18 (m, 1H, CH), 2.15–2.00 (m, 2H, CH), 1.98–1.71 (m, 3H, CH), 1.69–1.57 (m, 1H, CH), 1.44 (s, 3.6H, C*Me*3), 1.39 (s, 5.4H, C*Me*3), 1.38–1.18 (m, 1H, CH), 1.04 (dd, J = 9.0, 5.0 Hz, 0.6H, NCCH), 1.01–0.96 (m, 0.4H, NCCH), 0.90–0.72 (m, 1H, NCCH); ^{13}C NMR (100.6 MHz, $CDCl_3$) (rotamers) δ 169.2 (C=O), 168.4 (C=O), 156.5 (C=O), 137.5 (*ipso*-Ar), 128.6 (Ar), 127.9 (Ar), 127.1 (Ar), 83.6 (*C*Me3), 80.9 (NCH), 79.6 (NCH), 71.0 (NCH_2), 70.3 (OCH), 61.1 (NCH_2), 57.3 (OCH_2), 51.3

(CH_2), 46.1 (NCH_2), 30.1 (CH_2), 29.8 (CH_2), 28.5 (C*Me*$_3$), 27.0 (CH_2), 26.5 (CH_2), 26.0 (CH), 24.9 (CH_2), 24.2 (CH), 22.5 (CH_2), 18.2 (CH_2); HRMS *m/z* calcd for $C_{26}H_{35}BN_2O_7$ $(M + Na)^+$ 521.2430 found 521.2435 (–0.1 ppm error) and *N*-Boc BIDA boronate (*R,S,S,S*)-**1a''** (52 mg, 20%) as a white solid, mp 202-204°C; R_F (7:3 hexane-acetone 7:3) 0.28; IR (ATR) 2969, 1743, 1670 (C=O), 1400 cm^{-1}; ^1H NMR (400 MHz, $CDCl_3$) (60:40 mixture of rotamers) δ 7.37–7.24 (m, 5H, Ar), 4.60–4.48 (m, 2H, NCH), 4.36 (d, *J* = 11.3 Hz, 1H, NCH), 4.07–3.97 (m, 1H), 3.97–3.88 (m, 1H, OCH), 3.84 (d, *J* = 17.1 Hz, 1H, OCH), 3.59–3.41 (m, 3H), 3.20 (ddd, *J* = 11.5, 8.5, 8.5 Hz, 0.6H), 3.08–2.96 (m, 1H), 2.12–1.62 (m, 6H), 1.62–1.47 (m, 1H), 1.44 (s, 3.6H, CMe_3), 1.34 (s, 5.4H, CMe_3), 1.27–1.20 (m, 0.6H), 1.03–0.92 (m, 1H), 0.90–0.79 (m, 0.4H) 0.75 (dd, *J* = 5.5, 5.5 Hz, 0.4H, NCCH), 0.69 (dd, *J* = 5.0, 5.0 Hz, 0.6H, NCCH); ^{13}C NMR (100.6 MHz, $CDCl_3$) (rotamers) δ 168.6 (C=O), 168.4 (C=O), 156.8 (C=O), 136.7 (*ipso*-Ar), 128.7 (Ar), 128.4 (Ar), 128.2 (Ar), 80.9 (*C*Me$_3$), 80.0 (OCH), 79.9 (OCH), 72.5 (NCH), 72.0 (NCH_2), 63.0 (NCH_2), 57.6 (OCH_2), 47.5 (CH_2), 46.1 (CH_2), 30.2 (CH_2), 29.8 (CH_2), 28.51 (C*Me*$_3$), 28.47 (C*Me*$_3$), 26.6 (CH_2), 25.5 (CH_2), 24.9 (CH_2), 24.4 (NC*C*H), 24.2 (NC*C*H), 21.9 (CH_2), 18.2 (CH_2), 17.8 (CH_2); HRMS *m/z* calcd for $C_{26}H_{35}BN_2O_7$ $(M + Na)^+$ 521.2430 found 521.2435 (–0.1 ppm error). The stereochemistry was assigned by conversion of **1a'** into aryl cyclopropanes (*S,R*)-**14** and (*S,R*)-**32** of known configurations (*vide infra*).

Lab book reference RA 1-049/RA 1-077

***tert*-Butyl 1,1-dichloro-6-azaspiro[2.5]octane-6-carboxylate 6**

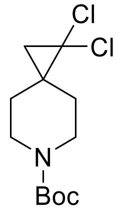

6

A solution of NaOH (40.0 g, 1.00 mol, 10 eq.) in H_2O (40 mL) was added dropwise to a solution of 1-*N*-boc-4-methylene-piperidine (19.7 g, 100 mmol, 1.0 eq.) and $BnNEt_3Cl$ (4.56 g, 20 mmol, 0.2 eq.) in $CHCl_3$ (300 mL) under Ar and the resulting mixture was stirred vigorously at 50 °C for 3 h, and then at rt for 16 h. The resulting solution was poured into H_2O (500 mL) and extracted with CH_2Cl_2 (3 × 100 mL). The combined organics were dried (Na_2SO_4) and evaporated under reduced pressure to give a yellow oil that solidified upon the addition and evaporation of hexane under reduced pressure.

Purification by recrystallisation from hexane gave dichlorocyclopropane **6** (26.5 g, 94%) as colourless needles, mp 86–88 °C; R_F (19:1 hexane-acetone) 0.29; IR (ATR) 2962, 2929, 2869, 1678 (C=O), 1423, 1364, 1244, 1167, 1127, 1045, 963, 863, 749 cm^{-1}; ^{1}H NMR (400 MHz, $CDCl_3$) δ 3.51 (ddd, *J* = 13.5, 7.5, 3.5 Hz, 2H, NCH), 3.41 (ddd, *J* = 13.5, 7.0, 4.0 Hz, 2H, NCH), 1.75 (ddd, *J* = 13.5, 7.0, 3.5 Hz, 2H, CH), 1.61 (ddd, *J* = 13.5, 7.5, 4.0 Hz, 2H, CH), 1.42 (s, 9H, CMe_3), 1.25 (s, 2H, CH_2CCl_2); ^{13}C NMR (100.6 MHz, $CDCl_3$) δ (rotamers) 154.8 (C=O), 79.7 (O*C*Me$_3$), 66.4 (CCl_2), 43.3 (NCH_2), 42.6 (NCH_2), 32.5 (CH_2), 31.5 (C), 31.3 (CH_2), 28.5 (C*Me*$_3$); HRMS (ESI) *m/z* calcd for $C_{12}H_{20}{}^{35}Cl_2NO_2$ $(M + H)^+$ 280.0866, found 280.0864 (+0.5 ppm error).

Lab book reference JRD_VIII_73, JRD_VIII_83

***tert*-Butyl 1-(6-methyl-4,8-dioxo-1,3,2,6-dioxadiazocan-2-yl)-6-azaspiro[2.5]octane-6-carboxylate 1b**

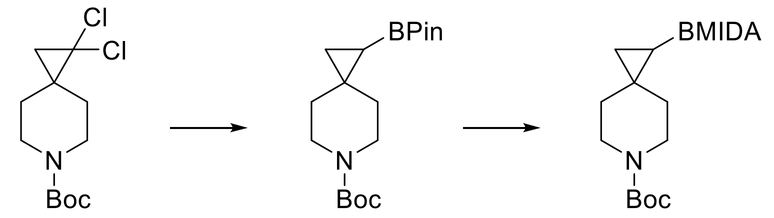

6 **7** **1b**

s-BuLi (17.7 mL, of a 1.21 M solution in cyclohexane, 21.4 mmol, 1.2 eq.) was added dropwise over 10 min to a solution of dichlorocyclopropane **6** (5.00 g, 17.8 mmol, 1.0 eq.) and HBpin (2.74 g, 3.11 mL, 21.4 mmol, 1.2 eq.) in THF (89 mL) under Ar at –78 °C and the resulting mixture was stirred at –78 °C for 30 min. The cooling bath was removed and the reaction was stirred for 1.5 h, and then cooled to 0 °C and saturated $NH_4Cl_{(aq)}$ (150 mL) was added. The mixture was extracted with EtOAc (3 × 100 mL) and the combined organics dried (Na_2SO_4) and evaporated under reduced pressure to give the crude mixture. Then, the crude mixture filtered through a short plug of silica with 19:1 hexane-acetone to give crude pinacol boronate **7** as a colourless oil 5.54 g which was used in the next step without further purification. ^{1}H NMR (400 MHz, $CDCl_3$) (rotamers) δ 3.48–3.38 (m, 4H, NCH), 1.59–1.54 (m, 2H, CH), 1.46–1.45 (m, 9H, CMe_3), 1.38–1.32 (m, 2H, CH), 1.28–1.19 (m, 12H, CMe_2), 0.69–0.64 (m, 2H, CH), -0.19 (t, *J* = 8.0 Hz, 1H, CHB); HRMS (ESI) *m/z* calcd for $C_{18}H_{33}BNO_4$ $(M + H)^+$ 338.2497, found 338.2499 (+0.3 ppm error).

Crude pinacol boronate **7** (5.54 g, 16.4 mmol based on 100% purity, 1.0 eq.) was dissolved in DMSO (82 mL) and methyliminodiacetic acid (MIDA) (15.7 g, 107 mmol, 6.5 eq.) and $HC(OEt)_3$ (11.0 g, 12.3 mL, 73.9 mmol, 4.5 eq.) were added under Ar. The resulting mixture was stirred and heated at 100 °C for 48 h. The reaction was allowed to cool to rt, saturated $NH_4Cl_{(aq)}$ (500 mL) was added and the mixture was extracted with EtOAc (4 × 200 mL). The combined organic extracts were washed with brine (200 mL), dried ($MgSO_4$) and evaporated under reduced pressure to give the crude product. Purification by flash column chromatography on silica with 19:1 to 7:3 EtOAc-acetone as eluent gave a tan solid that was recrystallised from THF-hexane to give MIDA boronate **1b** (3.12 g, 48% over two steps) as a colourless solid, mp 209–211 °C; R_F (19:1 EtOAc-acetone) 0.15; IR (ATR) 3009, 2965, 2926, 1772 (C=O, ester), 1744 (C=O, ester), 1681 (C=O, Boc), 1428, 1300, 1240, 1165, 1118, 988, 896, 849 cm^{-1}; ^{1}H NMR (400 MHz, acetone-d_6) δ 4.19 (m, 2H, MeNC*H*), 4.07 (d, 1H, *J* = 17.5 Hz, 1H, MeNC*H*), 4.01 (d, *J* = 17.0 Hz, 1H, MeNC*H*), 3.59 (ddd, *J* = 13.0, 7.0, 3.5, 1H, NCH), 3.48 (ddd, *J* = 13.0, 5.5, 4.0 Hz, 1H, NCH), 3.39–3.24 (m, 2H, NCH), 3.18 (s, 3H, NMe), 1.64 (ddd, *J* = 13.5, 8.0, 3.5 Hz, 1H, CH), 1.52–1.35 (m, 11H, CH, CMe_3), 1.24 (ddd, *J* = 9.5, 7.0, 3.5 Hz, 1H, CH), 0.55 (dd, *J* = 9.5, 3.5 Hz, 1H, CH), 1.32 (dd, *J* = 7.0, 3.5 Hz, 1H, CH), –0.24 (dd, *J* = 9.5, 7.0 Hz, 1H, CHB); ^{13}C NMR (100.6 MHz, acetone-d_6) (rotamers) δ 169.0 (C=O, ester), 168.9 (C=O, ester), 155.2 (C=O, Boc), 79.0 (O*C*Me_3), 62.44 (MeN*C*H_2), 62.40 (MeN*C*H_2), 46.8 (NMe), 45.0 (NCH_2), 44.2 (NCH_2), 39.0 (CH_2), 31.8 (CH_2), 28.6 (C*Me*$_3$), 22.8 (C), 15.1 (CH_2), 11.0 (CHB); ^{11}B (128.4 MHz, acetone-d_6) δ 11.4; HRMS (ESI) *m/z* calcd for $C_{17}H_{27}BN_2NaO_6$ $(M + Na)^+$ 389.1854, found 389.1857 (+0.1 ppm error).

Lab book reference JRD_VIII_78, JRD_VIII_85 (dichlorocyclopropane to BPin); JRD_VIII_81, JRD_VIII_82, JRD_VIII_88 (BMIDA)

Potassium {6-[(*tert*-butoxy)carbonyl]-6-azaspiro[2.5]octan-1-yl}trifluoroboranuide S2

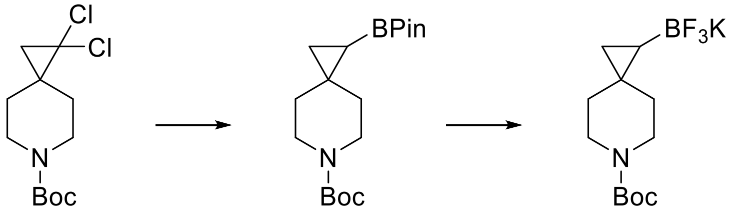

6 **7** **S2**

s-BuLi (17.7 mL, of a 1.21 M solution in cyclohexane, 21.4 mmol, 1.2 eq.) was added dropwise over 10 min to a solution of dichlorocyclopropane **6** (5.00 g, 17.8 mmol, 1.0 eq.) and HBpin (2.74 g, 3.11 mL, 21.4 mmol, 1.2 eq.) in THF (89 mL) under Ar at -78 °C and the resulting mixture was stirred at -78 °C

for 30 min. The cooling bath was removed and the reaction was stirred for 1.5 h, and then cooled to 0 °C and saturated $NH_4Cl_{(aq)}$ (150 mL) was added (slowly at first). The mixture was extracted with EtOAc (3 × 100 mL) and the combined organics dried (Na_2SO_4) and evaporated under reduced pressure to give the crude pinacol boronate. The crude pinacol boronate was filtered through a short plug of silica with 19:1 hexane-acetone to give pinacol boronate **7** as a colourless oil 5.11 g which was used in the next step without further purification.

KHF_2 (8.29 g, 106 mmol, 7.0 eq.) and H_2O (7.6 mL) were added to a solution of crude pinacol boronate **7** (5.11 g, 15.2 mmol based on 100% purity, 1.0 eq.) in MeOH (38 mL) and the resulting mixture was stirred at rt for 4 h. The reaction was evaporated under reduced pressure, then MeCN (230 mL) was added, heated to reflux and the mixture was filtered, and the filtrate evaporated under reduced pressure to give a colourless solid. The solid was washed with Et_2O (3 × 80 mL) and dried under vacuum to give the crude potassium trifluoroborate as a colourless solid. Purification by recrystallisation from boiling acetone gave potassium trifluoroborate **S2** as a colourless solid (1.44 g, 24% over two steps) mp 245–247 °C; IR 2974, 2925, 1680 (C=O), 1432, 1361, 1278, 1243, 1179, 1123, 1071, 1003, 971, 938, 892, 761 cm^{-1}; ^1H NMR (400 MHz, DMSO-d_6) δ 3.44–3.15 (m, 4H, NCH), 1.46–1.35 (m, 10H, CH, CMe_3), 1.29 (ddd, J = 13.5, 7.5, 3.5 Hz, 1H, CH), 1.18–1.05 (m, 1H, CH), 0.00–-0.06 (m, 2H, CH), -0.75–-0.85 (m, 1H, CHB); ^{13}C NMR (100.6 MHz, DMSO-d_6) (rotamers) δ 154.2 (C=O), 78.0 (O*C*Me_3), 44.5 (NCH_2), 43.7 (NCH_2), 38.6 (CH_2), 31.5 (CH_2), 28.2 (C*Me*$_3$), 19.7 (C), 15.3 (CHB), 14.4 (CH_2); ^{19}F (376.5 MHz, DMSO-d_6) δ –133.7; ^{11}B (128.4 MHz, DMSO-d_6) δ 3.0; HRMS (ESI) *m/z* calcd for $C_{12}H_{20}BF_3NO_2$ (M^-) 278.1545, found 278.1547 (+0.7 ppm error).

Lab book reference JRD_VIII_85, JRD_VIII_87

tert-Butyl 3-oxoazetidine-1-carboxylate S3

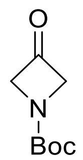

S3

Et_3N (35.1 g, 48.3 mL, 346 mmol, 3.0 eq.) was added to a solution of sulfur trioxide pyridine complex (55.1 g, 346 mmol, 3.0 eq.) in DMSO (180 mL) at 10 °C under Ar, followed by the addition of a solution of 1-Boc-3-hydroxyazetidine (20.0 g, 115 mmol, 1.0 eq.) in DMSO (100 mL) dropwise. The resulting mixture was stirred at rt for 16 h, then poured into brine (200 mL) and the resulting slurry was filtered.

The filtrate was extracted with Et_2O (3 × 200 mL) and the combined organics were washed with saturated $NaHCO_{3(aq)}$ (300 mL) and brine (300 mL) and then dried ($MgSO_4$) and carefully evaporated under reduced pressure to give ketone **S3** as a yellow liquid that was used in the next step without further purification (18.1 g, 1:0.45:0.44 ratio with pyridine and Et_2O, estimated mass of product ≈ 13.0 g, 66%). ^{1}H NMR (400 MHz, $CDCl_3$) δ 4.69 (s, 4H, CH_2), 1.48 (s, 9H, CMe_3). Spectroscopic data consistent with those reported in the literature.[3]

Lab book reference JRD_VIII_55, JRD_VIII_89

***tert*-Butyl 3-methyleneazetidine-1-carboxylate S4**

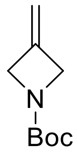

S4

KO*t*-Bu (20.1 g, 179 mmol, 2.36 eq.) was added to a suspension of methyltriphenylphosphonium bromide (64.0 g, 179 mmol, 2.36 eq.) in Et_2O (400 mL) at rt under Ar and the resulting mixture was stirred for 1 h. Ketone **S3** (13.0 g, 75.9 mmol, 1.0 eq.) was added as a solution in Et_2O (130 mL) and the reaction was stirred at 35 °C for 2 h. The mixture was allowed to cool to rt, filtered through Celite® and the filtrate was washed with H_2O (3 × 200 mL), brine (200 mL), dried (Na_2SO_4) and evaporated under reduced pressure. Purification by flash column chromatography on silica with 9:1 pentane-Et_2O as eluent gave alkene **S4** (9.24 g, 72%) as a colourless oil, R_F (9:1 pentane-Et_2O) 0.36; ^{1}H NMR (400 MHz, $CDCl_3$) δ 4.99 (quintet, J = 2.5 Hz, 2H, C=CH_2), 4.48 (t, J = 2.5 Hz, 4H, NCH_2), 1.45 (s, 9H, CMe_3). Spectroscopic data consistent with those reported in the literature.[4] Careful evaporation is required due to the volatility of the product.

Lab book reference JRD_VIII_57, JRD_VIII_90

tert-Butyl 1,1-dichloro-5-azaspiro[2.3]hexane-5-carboxylate S5

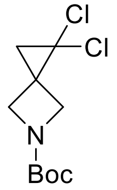

S5

A solution of NaOH (21.8 g, 546 mmol, 10 eq.) in H_2O (21.8 mL) was added dropwise to a solution of alkene **S4** (9.24 g, 54.6 mmol, 1.0 eq.) and $BnNEt_3Cl$ (2.49 g, 10.9 mmol, 0.2 eq.) in $CHCl_3$ (164 mL) under Ar and the resulting mixture was stirred vigorously at 50 °C for 3 h, and then at rt for 16 h. The resulting solution was poured into H_2O (50 mL) and extracted with CH_2Cl_2 (4 × 100 mL). The combined organics were dried (Na_2SO_4) and evaporated under reduced pressure to give a dark brown oil. Purification by flash column chromatography on silica with 8:2 hexane-EtOAc as eluent gave dichlorocyclopropane **S5** (6.01 g, 44%) as a yellow solid, mp 95–97 °C; R_F (8:2 hexane-EtOAc) 0.49; IR 2977, 2883, 1705 (C=O), 1400, 1358, 1130, 1048, 765 cm^{-1}; ^1H NMR (400 MHz, $CDCl_3$) δ 4.18 (d, J = 9.5 Hz, 2H, NCH), 3.93 (d, J = 9.5 Hz, 2H, NCH), 1.59 (s, 2H, CH_2), 1.47 (s, 9H, CMe_3); ^{13}C NMR (100.6 MHz, $CDCl_3$) δ 156.1 (C=O), 80.2 (CMe_3), 60.7 (CCl_2), 53.7 (NCH_2), 29.9 (CH_2), 29.2 (C), 28.5 (CMe_3); HRMS (ESI) *m/z* calcd for $C_{10}H_{16}{}^{35}Cl_2NO_2$ $(M + H)^+$ 252.0553, found 252.0550 (+1.2 ppm error).

Lab book reference JRD_VIII_68, JRD_VIII_70, JRD_VIII_91, JRD_VIII_92

tert-Butyl 1-(4,4,5,5-tetramethyl-1,3,2-dioxaborolan-2-yl)-5-azaspiro[2.3]hexane-5-carboxylate S6

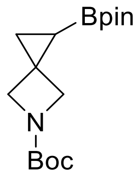

S6

s-BuLi (24.4 mL, of a 1.17 M solution in cyclohexane, 28.6 mmol, 1.2 eq.) was added dropwise over 10 min to a solution of dichlorocyclopropane **S5** (6.01 g, 23.8 mmol, 1.0 eq.) and HBpin (3.66 g, 4.15 mL, 28.6 mmol, 1.2 eq.) in THF (119 mL) under Ar at –78 °C and the resulting mixture was stirred at –78 °C for 30 min. The cooling bath was removed and the reaction was stirred for 1.5 h, and then cooled to 0 °C and saturated $NH_4Cl_{(aq)}$ (200 mL) was added (slowly at first). The mixture was extracted with EtOAc (3

× 150 mL) and the combined organics dried (Na_2SO_4) and evaporated under reduced pressure to give the crude mixture. Purification by flash column chromatography on silica with 9:1 hexane-EtOAc as eluent gave pinacol boronate **S6** (4.17 g, 57%) as a colourless oil that solidified upon standing, mp 76–78 °C; R_F (9:1 hexane-EtOAc) 0.09; IR 2977, 2878, 1701 (C=O), 1400, 1344, 1322, 1229, 1142, 1118, 983, 852 cm^{-1}; ^{1}H NMR (400 MHz, $CDCl_3$) δ 4.06–3.93 (m, 4H, NCH_2), 1.45 (s, 9H, CMe_3), 1.22 (s, 6H, CMe_2), 1.21 (s, 6H, CMe_2), 0.89 (dd, *J* = 10.0, 4.0 Hz, 1H, CH), 0.84 (dd, *J* = 7.0, 4.0 Hz, 1H, CH), 0.10 (dd, *J* = 10.0, 7.0 Hz, 1H, CHB); ^{13}C NMR (100.6 MHz, $CDCl_3$) δ (rotamers) 156.2 (C=O), 83.4 (O*C*Me$_2$), 79.3 ($OCMe_3$), 58.2 (NCH_2), 57.2 (NCH_2), 56.0 (NCH_2), 28.6 (C*Me*$_3$), 25.2 (C*Me*), 24.8 (C*Me*), 21.3 (C), 15.7 (CH), 4.2 (CHB); HRMS (ESI) *m/z* calcd for $C_{16}H_{29}BNO_4$ $(M + H)^+$ 310.2184, found 310.2191 (–1.3 ppm error).

Lab book reference JRD_VIII_93, JRD_VIII_94

***tert*-Butyl 1-(6-methyl-4,8-dioxo-1,3,6,2-dioxazaborocan-2-yl)-5-azaspiro[2.3]hexane-5-carboxylate 1c**

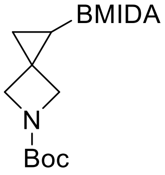

1c

Methyliminodiacetic acid (MIDA) (8.65 g, 58.8 mmol, 6.5 eq.) and $HC(OEt)_3$ (6.03 g, 6.77 mL, 40.7 mmol, 4.5 eq.) were added to a solution of pinacol boronate **S6** (2.80 g, 9.05 mmol, 1.0 eq.) in DMSO (45 mL) under Ar. The resulting mixture was stirred and heated at 100 °C for 48 h. The reaction was allowed to cool to rt, saturated $NH_4Cl_{(aq)}$ (280 mL) was added and the mixture was extracted with EtOAc (4 × 100 mL). The combined organic extracts were washed with brine (100 mL), dried ($MgSO_4$) and evaporated under reduced pressure to give the crude product. Purification by flash column chromatography on silica with 8:2 EtOAc-acetone as eluent gave MIDA boronate **1c** as a colourless solid (2.56 g, 84%) mp 235–237 °C; R_F (8:2 EtOAc-acetone) 0.20; IR 2974, 2877, 1760 (C=O, ester), 1692 (C=O, amide), 1457, 1343, 1408, 1313, 1290, 1115, 962, 887, 770, 597 cm^{-1}; ^{1}H NMR (400 MHz, acetone-d_6) δ 4.26–4.18 (m, 2H, MeNC*H*), 4.12 (d, *J* = 17.0 Hz, 1H, MeNC*H*), 4.08–3.77 (m, 5H, MeNC*H*, NCH_2), 3.23 (s, 3H, NMe), 1.41 (s, 9H, CMe_3), 0.80 (dd, *J* = 10.0, 4.0 Hz, 1H, CH), 0.49 (dd, *J* = 7.5, 4.0 Hz, 1H, CH), 0.11 (dd, *J* = 10.0, 7.5 Hz, 1H, C*H*B); ^{13}C NMR (100.6 MHz, acetone-d_6)

(rotamers) δ 169.0 (C=O, ester), 168.8 (C=O, ester), 156.4 (C=O, Boc), 79.0 ($OCMe_3$), 62.8 ($MeNCH_2$), 62.7 ($MeNCH_2$), 59.0 (NCH_2), 58.0 (NCH_2), 56.7 (NCH_2), 47.1 (NMe), 28.6 ($C\mathit{Me}_3$), 19.6 (C), 13.6 (CH_2), 8.2 (CHB); ^{11}B (128.4 MHz, acetone-d_6) δ 11.1; HRMS (ESI) *m/z* calcd for $C_{15}H_{24}BN_2O_6$ (M + H)$^+$ 339.1722, found 339.1728 (–1.0 ppm error).

Lab book reference JRD_VIII_95

Potassium {5-[(*tert*-butoxy)carbonyl]-5-azaspiro[2.3]hexan-1-yl}trifluoroboranuide S7

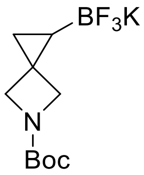

S7

KHF_2 (5.07 g, 65 mmol, 7.0 eq.) and H_2O (4.6 mL) were added to a solution of pinacol boronate **S6** (2.87 g, 9.28 mmol, 1.0 eq.) in MeOH (23 mL) and the resulting mixture was stirred at rt for 5 h. The reaction was evaporated under reduced pressure, then MeCN (140 mL) was added, heated to reflux and the mixture was filtered, and the filtrate evaporated under reduced pressure to give a colourless solid. The solid was washed with Et_2O (3 × 50 mL) and dried under vacuum to give potassium trifluoroborate **S7** as a colourless solid (2.48 g, 92%), mp 249–250 °C; IR 2977, 2964, 2875, 1687 (C=O), 1477, 1420, 1385, 1365, 1307, 1270, 1181, 1120, 1092, 958, 940, 876, 770 cm^{-1}; ^{1}H NMR (400 MHz, DMSO-*d*$_6$) δ 4.00–3.86 (m, 1H, NCH), 3.83–3.69 (m, 2H, NCH), 3.67–3.52 (m, 1H, NCH), 1.37 (s, 9H, CMe_3), 0.24 (dd, J = 10.5, 3.0 Hz, 1H, CH), 0.17 (dd, J = 7.5, 3.0 Hz, 1H, CH), -0.47–-0.59 (m, 1H, C*H*B); ^{13}C NMR (100.6 MHz, DMSO-d_6) (rotamers) δ 155.4 (C=O), 77.9 (CMe_3), 59.1 (NCH_2), 57.8 (NCH_2), 56.5 (NCH_2), 28.2 ($C\mathit{Me}_3$), 17.1 (C), 13.1 (CH_2), 13.0 (CH_2), 12.0 (*C*HB); ^{19}F (376.5 MHz, DMSO-d_6) δ -138.5; ^{11}B (128.4 MHz, DMSO-*d*$_6$) δ 2.5; HRMS (ESI) *m/z* calcd for $C_{10}H_{16}BF_3NO_2$ (M + H)$^+$ 250.1232, found 250.1240 (-2.4 ppm error).

Lab book reference JRD_VIII_96

***tert*-Butyl 3-methylidene-8-azabicyclo[3.2.1]octane-8-carboxylate S8**

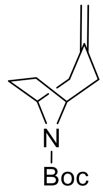

S8

KO*t*-Bu (31.1 mL of a 1.0 M solution in THF, 31.1 mmol, 1.40 eq.) was added to a suspension of methyltriphenylphosphonium bromide (11.6 g, 32.4 mmol, 1.46 eq.) in THF (100 mL) at 0 °C under Ar and the resulting mixture was stirred for 15 min and then heated at 70 °C for 45 min. The reaction was cooled to 15 °C (cold water bath), *N*-Boc nortropinone (5.00 g, 22.2 mmol, 1.0 eq.) was added as a solution in THF (25 mL) and the mixture was stirred at 15 °C for 2 h and then at rt for 16 h. The mixture was diluted with acetone (22 mL), filtered through Celite® and the filtrate evaporated under reduced pressure. The residue was partitioned between H_2O (100 mL), brine (50 mL) and EtOAc (3 × 150 mL) and the combined organic extracts were dried (Na_2SO_4) and evaporated under reduced pressure. Purification by flash column chromatography on silica with 1:0 to 9:1 hexane-EtOAc as eluent gave alkene **S8** (4.83 g, 97%) as a colourless oil, R_F (9:1 hexane-EtOAc) 0.38; ^{1}H NMR (400 MHz, $CDCl_3$) (50:50 mixture of rotamers) δ 4.84 (t, J = 2.0 Hz, 2H, C=CH_2), 4.35–4.11 (m, 2H, NCH), 2.59–2.37 (m, 2H, CH), 2.11–2.02 (m, 2H, CH), 1.94–1.78 (m, 2H, CH), 1.66–1.53 (m, 2H, CH), 1.48 (s, 9H, CMe_3). Spectroscopic data consistent with those reported in the literature.[5]

Lab book reference JRD_IX_36

***tert*-Butyl 3',3'-dichloro-8-azaspiro[bicyclo[3.2.1]octane-3,1'-cyclopropane]-8-carboxylate S9**

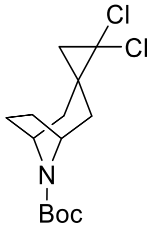

S9

A solution of NaOH (8.23 g, 206 mmol, 10 eq.) in H_2O (8.23 mL) was added dropwise to a solution of alkene **S8** (4.59 g, 20.6 mmol, 1.0 eq.) and $BnNEt_3Cl$ (937 mg, 4.11 mmol, 0.2 eq.) in $CHCl_3$ (62 mL) under Ar and the resulting mixture was stirred vigorously at 50 °C for 2 h, and then at rt for 16 h. The resulting solution was poured into H_2O (100 mL) and extracted with CH_2Cl_2 (3 × 100 mL). The combined organics were dried (Na_2SO_4) and evaporated under reduced pressure to give a colourless solid. Purification by flash column chromatography on silica with 9:1 to 8:2 hexane-EtOAc as eluent gave dichlorocyclopropane **S9** (6.19 g, 98%) as a colourless solid, mp 162–164 °C; R_F (9:1 hexane-EtOAc) 0.32; IR 2977, 2931, 1681 (C=O), 1402, 1365, 1322, 1168, 1101, 1054, 750 cm^{-1}; ^1H NMR (400 MHz, $CDCl_3$) (50:50 mixture of rotamers) δ 4.49–4.14 (m, 2H, NCH), 2.41–2.09 (m, 2H, CH), 2.04–1.86 (m, 2H, CH), 1.81–1.66 (m, 2H, CH), 1.54–1.40 (m, 11H, CMe_3 and CH), 1.27–1.15 (m, 2H, CH); ^{13}C NMR (100.6 MHz, $CDCl_3$) (rotamers) δ 153.2 (C=O), 79.7 ($OCMe_3$), 64.2 (CCl_2), 53.7 (NCH), 53.0 (NCH), 38.7 (CH_2), 37.9 (CH_2), 35.2 (CH_2), 28.6 ($C\mathit{Me}_3$), 27.9 (CH_2), 27.2 (CH_2), 26.8 (C); HRMS (ESI) *m/z* calcd for $C_{14}H_{21}{}^{35}Cl_2NNaO_2$ $(M + Na)^+$ 328.0842, found 328.0834 (–2.2 ppm error).

Lab book reference JRD_IX_38, JRD_IX_43

***tert*-Butyl (1*R**,3'*S**,5*S**)-3'-(4,4,5,5-tetramethyl-1,3,2-dioxaborolan-2-yl)-8-azaspiro[bicyclo[3.2.1]octane-3,1'-cyclopropane]-8-carboxylate S10**

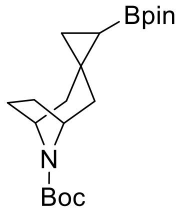

S10

s-BuLi (14.3 mL, of a 1.37 M solution in cyclohexane, 19.6 mmol, 1.2 eq.) was added dropwise over 10 min to a solution of dichlorocyclopropane **S9** (5.00 g, 16.3 mmol, 1.0 eq.) and HBpin (2.51 g, 2.84 mL, 19.6 mmol, 1.2 eq.) in THF (163 mL) under Ar at –40 °C and the resulting mixture was stirred at –40 °C for 30 min. The cooling bath was removed and the reaction was stirred for 1.5 h, and then cooled to 0 °C and saturated $NH_4Cl_{(aq)}$ (150 mL) was added (slowly at first). The mixture was extracted with EtOAc (3 × 150 mL) and the combined organics dried (Na_2SO_4) and evaporated under reduced pressure to give the crude pinacol boronate. Purification by flash column chromatography on silica with 8:2 to 7:3 hexane-Et_2O as eluent gave pinacol boronate **S10** (2.12 g of pure material, 36%; and 2.03 g of product and starting dichlorocyclopropane **S9** in a 1.0:0.41 ratio containing 1.51 g product, 25%; total product 3.63 g, 61%) as a colourless solid, mp 110-111 °C; R_F (8:2 hexane-Et_2O) 0.15; IR (ATR) 2978, 2927, 1685 (C=O), 1403, 1362, 1320, 1165, 1146, 1098, cm^{-1}; ^1H NMR (400 MHz, $CDCl_3$) δ 4.30–4.02 (m, 2H, NCH), 2.28–2.04 (m, 1H, CH), 2.03–1.73 (m, 5 H, CH), 1.40 (s, 9H, CMe_3), 1.30–1.23 (m, 1H, CH), 1.23–1.08 (m, 12H, CMe_2), 0.88–0.74 (m, 2H, CH_2), 0.74–0.64 (m, 1H, CH), –0.46 (dd, *J* = 9.0, 7.0 Hz, 1H, CHB); ^{13}C NMR (100.6 MHz, $CDCl_3$) (rotamers) δ 153.3 (C=O), 153.1 (C=O), 84.8 (*C*Me_2), 83.1 (*C*Me_2), 79.3 (O*C*Me_3), 78.9 (O*C*Me_3), 54.8 (NCH), 54.1 (NCH), 53.8 (NCH), 43.0 (CH_2), 42.3 (CH_2), 38.8 (CH_2), 38.1 (CH_2), 37.7 (CH_2), 28.6 (CMe_3), 28.1 (CH_2), 27.3 (CH_2), 25.3 (C*Me*), 24.5 (C*Me*), 23.0 (CH_2), 19.3 (C), 2.6 (CHB); ^{11}B (128.4 MHz, $CDCl_3$) δ 31.8; HRMS (ESI) *m/z* calcd for $C_{20}H_{34}BNNaO_4$ $(M + Na)^+$ 386.2473, found 386.2479 (–0.6 ppm error).

Lab book reference JRD_IX_49, JRD_IX_56

***tert*-Butyl (1*R**,3'*S**,5*S**)-3'-(6-methyl-4,8-dioxo-1,3,6,2-dioxazaborocan-2-yl)-8-azaspiro[bicyclo[3.2.1]octane-3,1'-cyclopropane]-8-carboxylate 1f**

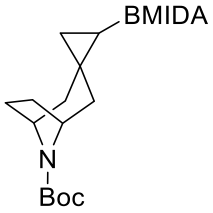

1f

Methyliminodiacetic acid (MIDA) (3.98 g, 27.1 mmol, 6.5 eq.) and $HC(OEt)_3$ (2.78 g, 3.11 mL, 18.7 mmol, 4.5 eq.) were added to a solution of pinacol boronate **S10** (1.51 g, 4.16 mmol, 1.0 eq.) in DMSO (20 mL) under Ar. The resulting mixture was stirred and heated at 100 °C for 31 h. The reaction was allowed to cool to rt, saturated $NH_4Cl_{(aq)}$ (130 mL) was added and the mixture was extracted with EtOAc (4 × 50 mL). The combined organic extracts were washed with brine (60 mL), dried ($MgSO_4$) and evaporated under reduced pressure to give the crude product. Purification by flash column chromatography on silica with 9:1 to 8:2 EtOAc-acetone as eluent gave MIDA boronate **1f** as a colourless solid (1.15 g, 71%) mp 255 °C (dec.); R_F (9:1 EtOAc-acetone) 0.16; IR (ATR) 2983, 1778 (C=O, ester), 1743 (C=O, ester), 1684 (C=O, amide), 1406, 1303, 1170, 1094, 985, 872 cm^{-1}; ^{1}H NMR (400 MHz, DMSO-d$_6$) δ 4.19–4.12 (m, 2H, MeNCH), 4.11–4.03 (m, 2H, NCH), 4.03–3.93 (m, 2H, MeNCH), 2.89 (s, 3H, NMe), 2.09–1.96 (m, 1H, CH) 1.96–1.72 (m, 5H, CH), 1.40 (s, 9H, CMe_3), 1.26–1.09 (m, 1H, CH), 0.81–0.65 (m, 2H, CH), 0.49–0.37 (m, 1H, CH), –0.63 (dd, *J* = 9.5, 7.5 Hz, 1H, C*H*B); ^{13}C NMR (100.6 MHz, DMSO-d$_6$) (rotamers) δ 169.1 (C=O, ester), 169.0 (C=O, ester), 152.6 (C=O, Boc), 78.1 (O*C*Me$_3$), 61.4 (MeN*C*H$_2$), 61.3 (MeN*C*H$_2$), 54.6 (NCH_2), 54.1 (NCH_2), 53.6 (NCH_2), 46.2 (NMe), 46.0 (NMe), 42.9 (CH_2), 42.1 (CH_2), 37.3 (CH_2), 36.3 (CH_2), 28.4 (C*Me*$_3$), 28.3 (C*Me*$_3$), 28.2 (C*Me*$_3$), 28.0 (C*Me*$_3$), 27.6 (CH_2), 26.9 (CH_2), 19.8 (CH_2), 19.7 (CH_2), 15.6 (C), 5.5 (CHB); ^{11}B (128.4 MHz, DMSO-d$_6$) δ 11.7; HRMS (ESI) *m/z* calcd for $C_{19}H_{29}BN_2NaO_6$ $(M + H)^+$ 415.2011, found 415.2015 (–0.1 ppm error).

Lab book reference JRD_IX_61

Potassium {(1*R,3'*S**,5*S**)-8-[(*tert*-butoxy)carbonyl]-8-azaspiro[bicyclo[3.2.1]octane-3,1'-cyclopropan]-3'-yl}trifluoroboranuide S11**

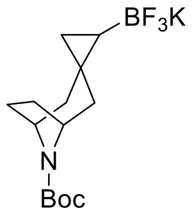

S11

KHF_2 (752 mg, 9.63 mmol, 7.0 eq.) and H_2O (690 μL) were added to a solution of pinacol boronate **S10** (500 mg, 1.38 mmol, 1.0 eq.) in MeOH (3.45 mL) and the resulting mixture was stirred at rt for 24 h. The reaction was evaporated under reduced pressure, then MeCN (21 mL) was added, heated to reflux and the mixture was filtered, and the filtrate evaporated under reduced pressure. Et_2O (5 mL) was added to the residue and the mixture evaporated under reduced pressure (3 ×) to give a colourless solid. The solid was washed with hexane (3 × 8 mL) and dried under vacuum to give the crude potassium trifluoroborate as a colourless solid (382 mg) contaminated with pinacol in a 1:1 ratio (determined by ^1H NMR in DMSO-d_6). The crude potassium trifluoroborate was stirred in ice-cold 1:1 hexane-Et_2O (10 mL) for 20 mins and then filtered, washed with ice-cold 1:1 hexane-Et_2O and dried under vacuum to give potassium trifluoroborate **S11** as a colourless solid (268 mg, 57%) mp 195–198 °C; IR 2975, 1677 (C=O), 1406, 1366, 1331, 1251, 1158, 1104, 1088, 997, 929 cm^{-1}; ^1H NMR (400 MHz, DMSO-d_6) δ 4.15–3.88 (m, 2H, NCH), 2.00–1.65 (m, 6 H, CH), 1.39 (s, 9H, CMe_3), 1.12–1.00 (m, 1H, CH), 0.62–0.45 (CH), 0.30–0.13 (m, 2H, CH), -1.05–-1.22 (m, CHB); ^{13}C NMR (100.6 MHz, DMSO-d_6) (rotamers) δ 152.4 (C=O), 78.0 (OC*Me*$_3$), 77.7 (OC*Me*$_3$), 54.9 (NCH_2), 54.6 (NCH_2), 54.0 (NCH_2), 53.7 (NCH_2), 43.8 (CH_2), 42.8 (CH_2), 38.1 (CH_2), 37.1 (CH_2), 28.3 (C*Me*$_3$), 27.8 (CH_2), 27.7 (CH_2), 27.2 (CH_2), 27.0 (CH_2), 19.9 (CH_2), 13.5 (C), 9.5 (CHB); HRMS (ESI) *m/z* calcd for $C_{14}H_{22}BF_3NO_2$ $(M)^-$ 304.1701, found 304.1712 (–2.7 ppm error).

Lab book reference JRD_IX_65

***tert*-Butyl 7,7-dibromo-3-azabicyclo[4.1.0]heptane-3-carboxylate 9**

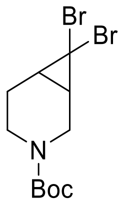

9

$CHBr_3$ (6.56 mL, 75.0 mmol, 3 eq.) and benzyltriethylammonium chloride (1.03 g, 4.50 mmol, 0.2 eq.) were added to a stirred solution of *tert*-butyl 1,2,3,6-tetrahydropyridine-1-carboxylate **8** (4.58 g, 25.0 mmol, 1.0 eq.) in CH_2Cl_2 (75 mL) at rt. Then, a solution of NaOH (39.0 g, 975 mmol, 39 eq.) in H_2O (39 mL) was added. The resulting solution was stirred and heated at 45 °C for 18 h. The mixture was then allowed to cool to rt. Then, H_2O (40 mL) was added, and the mixture was extracted with CH_2Cl_2 (3 × 40 mL). The combined organic extracts were dried (Na_2SO_4), decanted from the black sediment formed and evaporated under reduced pressure to give the crude product. Purification by flash chromatography on silica with 95:5 to 90:10 to 70:30 hexane-Et_2O as eluent gave dibromocyclopropane **9** (6.66 g, 75%) as a yellow oil, R_F (1:1 hexane-Et_2O) 0.44; IR (ATR) 1689 (C=O), 1423, 1249, 1167 cm^{-1}; ^{1}H NMR (400 MHz, $CDCl_3$) (60:40 mixture of rotamers) δ 4.11–3.75 (m, 1.6H, NCH), 3.67–3.38 (m, 1.4H, NCH), 2.72 (m, 0.4H, NCH), 2.63–2.50 (m, 0.6H, NCH), 2.14–2.00 (m, 2H, CH), 1.91–1.74 (m, 1H, CH), 1.63 (m, 1H, CH), 1.42 (s, 9H, CMe_3); ^{13}C NMR (100.6 MHz, $CDCl_3$) (rotamers) δ 154.5 (C=O), 79.9 (O*C*Me_3), 40.3 (NCH_2), 39.8 (NCH_2), 39.5 (NCH_2), 38.6 (NCH_2), 36.6 (CBr_2), 36.5 (CBr_2), 28.6 (C*Me_3*), 26.7 (CH), 26.6 (CH), 25.9 (CH), 25.7 (CH), 20.9 (CH_2), 20.7 (CH_2); MS (EI) *m/z* 376 ($^{79;79}$M + Na)$^+$; HRMS (ESI) *m/z* calcd for $C_{11}H_{17}{}^{79}Br_2NO_2$ ($^{79;79}$M + Na)$^+$ 375.9518, found 375.9507 (+3.0 ppm error).

Lab book reference HFK7-014

tert-Butyl (1*R,6*R**,7*S**)-7-bromo-3-azabicyclo[4.1.0]heptane-3-carboxylate 10**

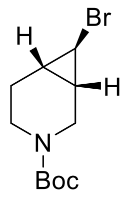

10

Dimethyl phosphite (18.2 mL, 198 mmol, 6.0 eq.) was added to a stirred solution of dibromocyclopropane **9** (11.6 g, 33.0 mmol, 1.0 eq.) in anhydrous DMSO (200 mL) at rt under Ar. Then, KO*t*-Bu (14.8 g, 132 mmol, 4.0 eq.) was added portionwise and the resulting solution was stirred at rt for 1 h. Saturated $Na_2CO_{3(aq)}$ (300 mL) and Et_2O (400 mL) were added. The two layers were separated, and the aqueous layer was extracted with Et_2O (3 × 300 mL). The combined organics were dried (Na_2SO_4) and evaporated under reduced pressure to give the crude product. Purification by flash column chromatography on silica with 95:5 hexane-EtOAc as eluent gave bromocyclopropane **10 (**6.68 g, 70%) as a colourless oil, R_F (1:1 hexane-Et_2O) 0.52; IR (ATR) 1694 (C=O), 1366, 1168, 1129 cm^{-1}; ^1H NMR (400 MHz, $CDCl_3$) δ 3.97 (br d, *J* = 14.0 Hz, 1H, NCH), 3.37 (dd, *J* = 14.0, 5.0 Hz, 1H, NCH), 3.40–3.35 (m, 1H, CH), 2.87–2.75 (m, 1H, NCH), 2.58 (dd, *J* = 3.5, 3.5 Hz, 1H, CHBr), 1.97–1.89 (m, 1H, CH), 1.82–1.71 (m, 1H, CH), 1.56–1.50 (m, 1H, CH), 1.45–1.42 (m, 1H, CH), 1.43 (br s, 9H, CMe_3); ^{13}C NMR (100.6 MHz, $CDCl_3$) δ 154.9 (C=O), 79.9 (O*C*Me_3), 41.0 (br, NCH), 28.5 (C*Me*$_3$), 23.6 (CHBr), 22.1 (CH2), 21.1 (CH), 19.9 (CH) (NCH_2 resonance not resolved); MS (EI) *m/z* 298 (^{79}M + Na)$^+$; HRMS (ESI) *m/z* calcd for $C_{11}H_{18}{}^{79}BrNO_2$ (^{79}M + Na)$^+$ 298.0413, found 298.0407 (+1.9 ppm error).

Lab book reference HFK7-095

tert-Butyl (1R*,6S*,7S*)-7-(4,4,5,5-tetramethyl-1,3,2-dioxaborolan-2-yl)-3-azabicyclo[4.1.0]heptane-3-carboxylate 11

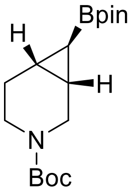

11

n-BuLi (12.0 mL of a 2.44 M solution in hexanes, 29.5 mmol, 1.5 eq.) was added dropwise to a stirred solution of bromocyclopropane **10** (5.71 g, 19.6 mmol, 1.0 eq.) in THF (200 mL) at –78 °C under Ar. The resulting solution was stirred at –78 °C for 1 h. Then, *i*-PrOBpin (6.00 mL, 29.5 mmol, 1.5 eq.) was added and the solution was allowed to warm to rt. Saturated $NH_4Cl_{(aq)}$ (200 mL) was added, and the mixture was extracted with Et_2O (3 × 100 mL). The combined organic extracts were washed with brine (100 mL), dried (Na_2SO_4) and evaporated under reduced pressure to give the crude product. Purification by flash column chromatography on silica with 90:10 to 70:30 to 60:40 hexane-Et_2O as eluent gave pinacol boronate **11** (5.45 g, 86%) as a pale yellow oil, R_F (7:3 hexane-Et2O) 0.25; IR (ATR) 1692 (C=O), 1318, 1144, 1167 cm^{-1}; ^1H NMR (400 MHz, $CDCl_3$) δ 3.81–3.71 (m, 1H, NCH), 3.53 (dd, *J* = 13.5, 3.5 Hz, 1H, NCH), 3.38–3.20 (m, 1H, NCH), 3.10–2.90 (ddd, *J* = 13.5, 8.5, 5.5 Hz, 1H, NCH), 2.02-1.84 (m, 1H, CH), 1.76–1.65 (m, 1H, CH), 1.41 (s, 9H, CMe_3), 1.20 (s, 14H, Me and CH), –0.25 (dd, *J* = 5.5, 5.5 Hz, 1H, CHB); ^{13}C NMR (100.6 MHz, $CDCl_3$) (rotamers) δ 155.2 (C=O), 83.1 (O*C*Me_3), 79.3 (O*C*Me_3), 42.7 (NCH_2), 41.8 (NCH_2), 40.5 (NCH_2), 39.4 (NCH_2), 28.6 (C*Me_3*), 25.0 (Me) 24.9 (Me), 24.8 (Me), 23.1 (CH_2), 16.7 (CH), 15.1 (CH), 4.9 (CHB); MS (EI) *m/z* 346 $(M + Na)^+$; HRMS (ESI) *m/z* calcd for $C_{17}H_{30}BNO_4$ $(M + Na)^+$ 346.2160, found 346.2162 (+0.3 ppm error).

Lab book reference HFK7-090

tert-Butyl (1*R,6*S**,7*S**)-7-(6-methyl-4,8-dioxo-1,3,6,2-dioxazaborocan-2-yl)-3-azabicyclo[4.1.0]heptane-3-carboxylate 1g**

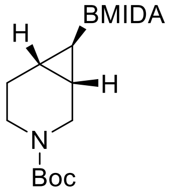

1g

$HC(OEt)_3$ (9.20 mL, 55.8 mmol, 4.5 eq.) and MIDA (11.9 mg, 80.6 mmol, 6.5 eq.) were added to a stirred solution of pinacol boronate **11** (4.00 g, 12.4 mmol, 1.0 eq.) in anhydrous DMSO (60 mL) at rt. The resulting suspension was stirred and heated at 100 °C for 24 h. The reaction was then allowed to cool to rt and saturated $NH_4Cl_{(aq)}$ (30 mL) was added. The mixture was extracted with EtOAc (3 × 30 mL). The combined organic extracts were washed with brine (30 mL), dried ($MgSO_4$) and evaporated under reduced pressure to give the crude product. Purification by recrystallisation from acetone gave MIDA boronate **1g** (3.52 g, 80%) as a beige solid, mp 189–192 °C; R_F (4:1 EtOAc-acetone) 0.23; IR (ATR) 1746 (C=O, ester), 1674 (C=O, Boc), 1243, 1283, 1004 cm^{-1}; ^{1}H NMR (400 MHz, *d*$_6$-acetone) δ 4.21 (d, 1H, *J* = 17.0 Hz, 1H, MeNC*H*), 4.17 (d, 1H, *J* = 17.0 Hz, 1H, MeNC*H*), 4.09 (d, *J* = 17.0 Hz, 1H, MeNC*H*), 4.04 (d, *J* = 17.0 Hz, 1H, MeNC*H*), 3.80 (br d, *J* = 13.3 Hz, 1H, NCH), 3.60–3.46 (m, 1H, NCH), 3.38 (ddd, *J* = 13.0, 11.5, 5.5 Hz, 1H, NCH), 3.20 (s, 3H, NMe), 3.08–2.86 (m, 1H, NCH), 2.00–1.84 (m, 1H, CH), 1.77–1.63 (m, 1H, CH), 1.41 (s, 9H, CMe_3), 1.03–0.80 (m, 2H, CH), –0.39 (dd, *J* = 6.0, 6.0 Hz, 1H, CHB); ^{13}C NMR (100.6 MHz, *d*$_6$-acetone) (rotamers) δ 169.0 (C=O, ester), 168.9 (C=O, ester), 155.4 (C=O, Boc), 79.1 (O*C*Me_3), 62.9 (MeN*C*H_2), 62.8 (MeN*C*H_2), 47.1 (NMe), 43.5 (NCH_2), 42.7 (NCH_2), 41.5 (NCH_2), 40.3 (NCH_2), 28.6 (C*Me*$_3$), 23.8 (CH_2), 23.3 (CH_2), 14.3 (CH), 12.3 (CH), 9.35 (CHB); MS (EI) *m/z* 375 (M + Na)$^+$; HRMS (ESI) *m/z* calcd for $C_{16}H_{25}BN_2O_6$ (M + Na)$^+$ 375.1698, found 375.1703 (–0.5 ppm error).

Lab book reference HFK7-091

Potassium [(1*S,6*R**,7*R**)-3-[(*tert*-butoxy)carbonyl]-3-azabicyclo[4.1.0]heptan-7-yl]trifluoroboranuide S12**

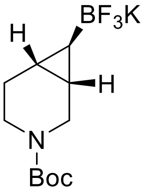

S12

KHF_2 (3.9 g, 50 mmol, 7.0 eq.) was added to a stirred solution of pinacol boronate **11** (2.30 g, 7.12 mmol, 1.0 eq.) in 5:1 MeOH/H_2O (50 mL) at rt under Ar. The resulting solution was stirred at rt for 1.5 h. Then, the solvent was evaporated under reduced pressure. Then, CH_3CN (3 × 100 mL) was added and the solvent evaporated under reduced pressure. Then, CH_3CN (100 mL) was added and the resulting suspension was stirred and heated at 80 °C for 20 min. The solids were removed by filtration and evaporated under reduced pressure to give crude BF_3K salt. The solid was washed with Et_2O (2 × 50 mL) to give BF_3K **S12** (2.05 g, 95%) as a white solid, mp 200–203 °C; R_F (7:3 CH_2Cl_2-acetone) 0.32; IR (ATR) 1678 (C=O), 1451, 1282, 882 cm^{-1}; ^1H NMR (400 MHz, DMSO-d_6) δ 3.48–3.41 (m, 2H, NCH), 3.16–3.10 (m, 1H, NCH), 2.94–2.82 (m, 1H, NCH), 1.78 (dddd, J = 12.0, 6.0, 6.0, 6.0 Hz, 1H, CH), 1.48–1.42 (m, 1H, CH), 1.36 (s, 9H, CMe_3), 0.56–0.50 (m, 2H, C*H*CHB), –0.88– -0.97 (m, 1H, CHB); ^{13}C NMR (100.6 MHz, DMSO-d_6) δ 154.2 (C=O, Boc), 77.9 (O*C*Me_3), 43.7 (NCH_2), 42.9 (NCH_2), 40.9 (NCH_2), 39.9 (NCH_2), 28.2 (C*Me_3*), 25.0 (CHB), 24.0 (CH_2), 23.6 (CH_2), 12.4 (*C*HCHB), 10.5 (*C*HCHB); ^{11}B (128.4 MHz, $CDCl_3$) δ 2.7; MS (EI) *m/z* 264 $(M)^-$; HRMS (ESI) *m/z* calcd for $C_{11}H_{18}BF_3NO_2$ $(M)^-$ 264.1388, found 264.1386 (+1.6 ppm error).

Lab book reference HFK7-092

tert-Butyl 3-methylenepyrrolidine-1-carboxylate S13

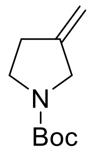

S13

KO*t*-Bu (15.8 g, 130 mmol, 1.2 eq.) was added to a stirred suspension of methyltriphenylphosphonium bromide (45.4 g, 130 mmol, 1.2 eq.) in THF (150 mL) at 0 °C under Ar. The resulting mixture was stirred at 0 °C for 1 h. Then, a solution of *tert*-butyl 3-oxopyrrolidine-1-carboxylate (20.0 g, 108 mmol, 1.0 eq.) in THF (100 mL) was added at 0 °C. After being allowed to warm to rt, the mixture was stirred at rt for 16 h. The mixture was poured into water (150 mL) and the two layers were separated. The aqueous layer was extracted with Et_2O (3 × 150 mL). The combined organic layers were dried ($MgSO_4$) and evaporated under reduced pressure to give the crude product. Purification by flash column chromatography on silica using 10:90 EtOAc-hexane as eluent gave alkene **S13** (11.1 g, 56%) as a pale-yellow oil, R_F (20:80 EtOAc-hexane) 0.48; IR (ATR) 2978, 2934, 2897, 1687 (C=O), 1391, 1367, 1253, 1159, 890, 775, 730 cm^{-1}; ^1H NMR (400 MHz, $CDCl_3$) δ 5.00–4.91 (m, 2H, C=CH_2), 3.96–3.88 (m, 2H, NCH_2), 3.50–3.41 (m, 2H, NCH_2), 2.58–2.52 (m, 2H, CH_2), 1.46 (s, 9H, CMe_3); ^{13}C NMR (100.6 MHz, $CDCl_3$) *δ* 154.5 (C=O), 146.1 (*C*=CH_2), 145.2 (*C*=CH_2), 106.7 (C=*C*H_2), 106.6 (C=*C*H_2), 79.3 (O*C*Me_3), 50.4 (NCH), 50.1 (NCH), 45.9 (NCH), 45.4 (NCH), 32.2 (CH), 31.5 (CH), 28.5 (C*Me*3); MS (ESI) *m/z* 206 [$(M + Na)^+$, 100], HRMS ESI *m/z* calcd for $C_{10}H_{17}NO_2$ [$(M + Na)^+$, 100] 206.1151, found 206.1155 (–1.8 ppm error). Spectroscopic data consistent with those reported in the literature.[6]

Lab Book Reference: SY-2-117

***tert*-Butyl 1,1-dibromo-5-azaspiro[2.4]heptane-5-carboxylate S14**

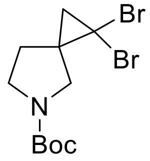

S14

A solution of NaOH (93.6 g, 2.34 mol, 39.0 eq.) in H_2O (94 mL) was added to a stirred solution of alkene **S13** (11.0 g, 60.0 mmol, 1.0 eq.) and $BnEt_3N^+Cl^-$ (2.46 g, 10.8 mmol, 0.18 eq.) in CH_2Cl_2 (200 mL) at rt under Ar. Then, bromoform (7.4 mL, 84.0 mmol, 1.4 eq.) was added dropwise. The resulting mixture was stirred vigorously and heated at 50 °C for 16 h. After being allowed to cool to rt, the mixture was poured into water (200 mL) and extracted with CH_2Cl_2 (3 × 200 mL). The combined organics were dried ($MgSO_4$) and evaporated under reduced pressure to give the crude product. Purification by flash column chromatography on silica using 10:90 EtOAc-hexane as eluent gave dibromocyclopropane **S14** (14.7 g, 69%) as an off-white solid, R_F (10:90 EtOAc-hexane) 0.26; IR (ATR) 2976, 2930, 2873, 1691, 1397, 1365, 1246, 1167, 1112, 1036, 877, 771, 687 cm^{-1}; ^1H NMR (400 MHz, $CDCl_3$) (50:50 mixture of rotamers) δ 3.85–3.46 (m, 3H, NCH), 3.32 (d, *J* = 11.5 Hz, 0.5H, NCH), 3.24 (d, *J* = 11.5 Hz, 0.5H, NCH), 2.49–2.35 (m, 1H, CH), 1.86–1.70 (m, 3H, CH), 1.47 (s, 9H, CMe_3); ^{13}C NMR (100.6 MHz, $CDCl_3$) (rotamers) δ 154.3 (C=O), 79.9 (O*C*Me_3), 53.8 (NCH_2), 53.6 (NCH_2), 46.4 (NCH_2), 46.0 (NCH_2), 36.3 (C), 35.6 (C), 35.0 (CBr_2), 34.4 (CH_2), 34.3 (CBr_2), 31.5 (CH_2), 28.6 (C*Me*$_3$); HRMS (ESI) *m/z* calcd for $C_{11}H_{18}{}^{79}Br_2NO_2$ ($^{79,79}M + Na)^+$ 375.9518, found 375.9515 (+0.8 ppm error).

Lab Book Reference: SY-2-120

***tert*-butyl (1*R**,3*S**)-1-bromo-5-azaspiro[2.4]heptane-5-carboxylate S15 and *tert*-butyl (1*R**,3*R**)-1-bromo-5-azaspiro[2.4]heptane-5-carboxylate S16**

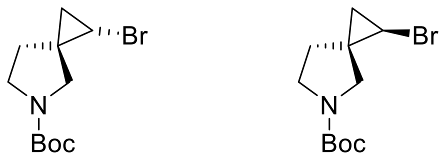

S15 **S16**

KO*t*-Bu (29.3 g, 0.261 mol, 6.0 eq.) was added portion wise to a stirred solution of dibromocyclopropane **S14** (15.45 g, 43.5 mmol, 1.0 eq.) and dimethyl phosphite (23.9 mL, 0.261 mol, 6.0 eq.) in anhydrous DMSO (200 mL) at 0 °C under Ar. The resulting solution was stirred at rt for 1.5 h. Then, Et_2O (100 mL, chilled to 0 °C) and saturated $Na_2CO_{3(aq)}$ (100 mL) were added. A white precipitate formed and additional H_2O (~100 mL) was added to re-dissolve the solids. The two layers were separated and the aqueous layer was extracted with Et_2O (3 × 150 mL). The combined organic layers were dried ($MgSO_4$) and evaporated under reduced pressure to give the crude product which contained a 55:45 mixture (by ^1H NMR spectroscopy) of monobromocyclopropanes **S15** and **S16**. Purification by flash column chromatography on silica using 10:90 EtOAc-hexane as eluent (× 3) gave monobromocyclopropane **S15** (5.82 g, 48%) as a pale yellow oil, R_F (10:90 EtOAc-hexane) 0.38; IR (ATR) 2977, 2934, 2871, 1696, 1403, 1366, 1171, 1112, 882, 772 cm^{-1}; ^1H NMR (400 MHz, $CDCl_3$) δ 3.60–3.38 (m, 2H, NCH), 3.34–3.18 (m, 2H, NCH), 3.07–3.02 (m, 1H, BrCH), 2.13 (ddd, *J* = 13.0, 7.5, 5.0 Hz, 1H, CH), 1.97–1.84 (m, 1H, CH), 1.46 (s, 9H, CMe_3), 1.39–1.29 (m, 1H, CH), 0.93 (dd, *J* = 7.0, 4.5 Hz, 1H, CHBr); ^{13}C NMR (100.6 MHz, $CDCl_3$) (rotamers) δ 154.4 (C=O), 154.2 (C=O), 79.4 (O*C*Me$_3$), 53.0 (NCH_2), 52.5 (NCH_2), 45.8 (NCH_2), 45.4 (NCH_2), 31.9 (CH_2), 31.2 (C), 28.5 (C*Me*$_3$), 26.9 (CH_2), 26.6 (CH_2), 19.8 (CH), 19.5 (CH); MS (ESI) *m/z* 298 (M + Na)$^+$; HRMS (ESI) *m/z* calcd for $C_{11}H_{18}{}^{79}BrNO_2$ (^{79}M + Na)$^+$ 298.0413, found 298.0412 (+0.3 ppm error) and monobromocyclopropane **S16** (4.24 g, 35%) as an off-white crystalline solid, R_F (10:90 EtOAc-hexane) 0.29; IR (ATR) 2975, 2934, 2873, 1693, 1402, 1366, 1170, 1111, 884, 771 cm^{-1}; ^1H NMR (400 MHz, $CDCl_3$) δ 3.67–3.45 (m, 3H, NCH), 3.41–3.27 (m, 1H, NCH), 3.00 (dd, *J* = 8.0, 4.5 Hz, 1H, BrCH), 1.87–1.79 (m, 2H, CH), 1.47 (s, 9H, CMe_3), 1.37–1.29 (m, 1H, CH), 1.01–0.97 (m, 1H, CH); ^{13}C NMR (100.6 MHz, $CDCl_3$) (rotamers) δ 154.5 (C=O), 79.5 (O*C*Me$_3$), 51.8 (NCH_2), 51.6 (NCH_2), 46.2 (NCH_2), 45.8 (NCH_2), 34.0 (CH_2), 33.3 (C), 28.6 (C*Me*$_3$), 26.0 (CH), 25.7 (CH), 20.7 (CH_2), 20.5 (CH_2); MS (ESI) *m/z* 298 [(^{79}M + Na)$^+$; HRMS (ESI) *m/z* calcd for $C_{11}H_{18}{}^{79}BrNO_2$ (M + Na)$^+$ 298.0413, found 298.0412 (+ 0.3 ppm error).

The structure of **S16** was confirmed by X-Ray crystallography (CCDC 2219557):

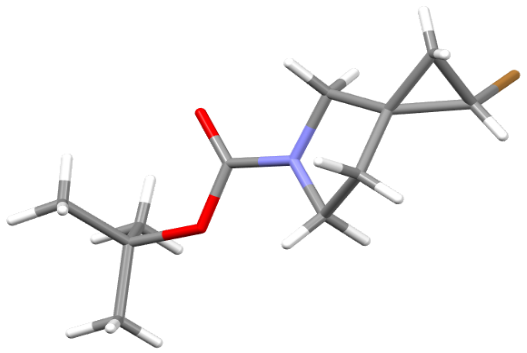

Lab Book Reference: SY-2-127

tert-Butyl (1_R_*,3_S_*)-1-(4,4,5,5-tetramethyl-1,3,2-dioxaborolan-2-yl)-5-azaspiro[2.4]heptane-5-carboxylate S17

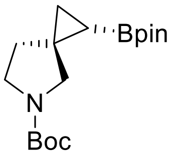

S17

n-BuLi (20.8 mL of a 1.52 M solution in hexane, 31.6 mmol, 1.5 eq.) was added dropwise to a stirred solution of monobromocyclopropane **S15** (5.82 g, 21.1 mmol, 1.0 eq.) in THF (100 mL) at –78 °C under Ar. The resulting solution was stirred at –78 °C for 1 h. Then, *i*-PrO-Bpin (6.5 mL, 31.6 mmol, 1.5 eq.) was added and the solution was stirred at –78 °C for 10 min. After being allowed to warm to rt, saturated $NH_4Cl_{(aq)}$ (100 mL) was added, and the two layers were separated. The aqueous layer was extracted with Et_2O (3 × 100 mL). The combined organic layers were dried ($MgSO_4$) and evaporated under reduced pressure to give the crude product. Purification by flash column chromatography on silica using 10:90 Et_2O-hexane as eluent gave boronic pinacol ester **S17** (4.01 g, 59%) as a white solid, mp 73–75 °C; R_F (10:90 Et_2O-hexane) 0.16; IR (ATR) 2977, 2933, 2870, 1696 (C=O), 1402, 1361, 1322, 1169, 1145, 1108, 858 cm^{-1}; ^{1}H NMR (400 MHz, $CDCl_3$) (50:50 mixture of rotamers) δ 3.54–3.37 (m, 2H, NCH), 3.28 (d, *J* = 10.5 Hz, 0.5H NCH), 3.24 (d, *J* = 10.5 Hz, 0.5H, NCH), 3.17 (d, *J* = 10.5 Hz, 0.5H, NCH), 3.11 (d, *J* = 10.5 Hz, 0.5H, NCH), 2.02–1.91 (m, 1H, CH), 1.88–1.77 (m, 1H, CH), 1.45 (s, 9H, CMe_3), 1.22 (s, 12H, CMe_2), 0.99–0.77 (m, 2H, CH), 0.15–0.03 (m, 1H, BCH); ^{13}C NMR (100.6 MHz, $CDCl_3$) (rotamers) δ 154.6 (C=O), 83.3 (O*C*Me_2), 79.1 (O*C*Me_3), 55.0 (NCH_2), 54.6 (NCH_2), 46.5 (NCH_2), 46.1 (NCH_2), 31.8 (CH_2), 31.1 (CH_2), 28.7 (C*Me*$_3$), 28.6 (C), 28.0 (C), 25.2 (C*Me*), 24.7 (C*Me*), 24.6 (C*Me*),

17.2 (CH_2), (BCH not resolved); ^{11}B (128.4 MHz, $CDCl_3$) δ 32.6; MS (ESI) *m/z* 346 [$(M + Na)^+$, 100]; HRMS (ESI) *m/z* calcd for $C_{17}H_{30}BNO_4$ $(M + Na)^+$ 346.2160, found 346.2161 (+ 0.2 ppm error)

Lab Book Reference: SY-2-132

tert-butyl (1*R,3*S**)-1-(6-methyl-4,8-dioxo-1,3,6,2-dioxazaborocan-2-yl)-5-azaspiro[2.4]heptane-5-carboxylate 1d**

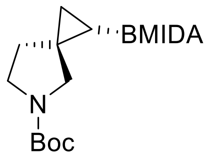

1d

MIDA (4.31 g, 29.3 mmol, 6.5 eq.) and $HC(OEt)_3$ (3.4 mL, 20.3 mmol, 4.5 eq.) were added to a stirred solution of pinacol boronate **S17** (1.46 g, 4.5 mmol, 1.0 eq.) in anhydrous DMSO (80 mL) at rt under Ar. The resulting mixture was stirred and heated at 100 °C for 48 h. After being allowed to cool to rt, saturated $NH_4Cl_{(aq)}$ (50 mL) was added and the mixture was extracted with EtOAc (3 × 50 mL). The combined organic extracts were washed with brine (50 mL), dried ($MgSO_4$) and evaporated under reduced pressure to give the crude product. Purification by flash column chromatography on silica with 60:40 to 50:50 CH_2Cl_2-acetone as eluent gave MIDA boronate **1d** (1.13 g, 71%) as an off-white solid, mp 222–224 °C; R_F (7:3 CH_2Cl_2-acetone) 0.23; IR (ATR) 2975, 2873, 1759 (C=O, ester), 1682 (C=O, Boc), 1411, 1345, 1298, 1162, 1130, 1024, 996, 893 cm^{-1}; ^1H NMR (400 MHz, $CDCl_3$) (60:40 mixture of rotamers) δ 3.92–3.84 (m, 2H, N*C*HC=O), 3.79–3.66 (m, 2H, N*C*HC=O), 3.63–3.47 (m, 1H, NCH), 3.43–3.33 (m, 2H, NCH), 3.16–3.05 (m, 1H, NCH), 3.03 (s, 3H, NMe), 2.11–1.99 (m, 1H, CH), 1.85–1.75 (m, 1H, CH), 1.46 (s, 5.4H, CMe_3), 1.44 (s, 3.6H, CMe_3), 0.81–0.74 (m, 1H, CH), 0.52–0.47 (m, 1H, CH), -0.03–-0.15 (m, 1H, BCH); ^{13}C NMR (100.6 MHz, $CDCl_3$) (rotamers) δ 167.2 (C=O, ester), 167.1 (C=O, ester), 154.7 (C=O, Boc), 154.5 (C=O, Boc), 79.2 (O*C*Me_3), 62.0 ($NCH_2C(O)$), 61.9 ($NCH_2C(O)$), 55.6 (NCH_2), 55.2 (NCH_2), 46.7 (NCH_2), 46.5 (NMe), 31.0 (CH_2), 30.4 (CH_2), 28.7 (C*Me*$_3$), 26.2 (C), 25.5 (C), 16.0 (CH_2), 15.6 (CH_2) (BCH not resolved); ^{11}B (128.4 MHz, $CDCl_3$) δ 12.5; MS (ESI) *m/z* 375 $(M + Na)^+$; HRMS (ESI) m/z calcd for $C_{16}H_{25}BN_2O_6$ $(M + Na)^+$ 375.1698, found 375.1699 (+0.4 ppm error).

Lab Book Reference: SY-2-136

tert-Butyl (1R*,3R*)-1-(4,4,5,5-tetramethyl-1,3,2-dioxaborolan-2-yl)-5-azaspiro[2.4]heptane-5-carboxylate S18

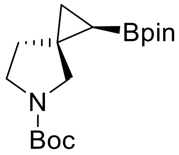

S18

n-BuLi (13.1 mL of a 1.50 M solution in hexane, 19.7 mmol, 1.5 eq.) was added dropwise to a stirred solution of monobromocyclopropane **S16** (3.62 g, 13.1 mmol, 1.0 eq.) in THF (200 mL) at –78 °C under Ar. The resulting solution was stirred at –78 °C for 1 h. Then, *i*-PrOBpin (4.0 mL, 19.7 mmol, 1.5 eq.) was added and the solution was stirred at –78 °C for 10 min. After being allowed to warm to rt, saturated $NH_4Cl_{(aq)}$ (100 mL) was added and the two layers were separated. The aqueous layer was extracted with Et_2O (3 × 100 mL). The combined organic layers were dried ($MgSO_4$) and evaporated under reduced pressure to give the crude product. Purification by flash column chromatography on silica using 10:90 Et_2O-hexane as eluent gave boronic pinacol ester **S18** (3.01 g, 71%) as a white solid, mp 48–50 °C; R_F (10:90 Et_2O-hexane) 0.10; IR (ATR) 2976, 2933, 2869, 1694 (C=O), 1402, 1363, 1322, 1259, 1168, 1144, 1109, 859 cm^{-1}; ^1H NMR (400 MHz, $CDCl_3$) δ 3.52–3.44 (m, 1H, NCH), 3.43–3.35 (m, 2H, NCH), 3.25 (d, *J* = 11.0 Hz, 1H, NCH), 2.02–1.88 (m, 1H, CH), 1.65–1.55 (m, 1H, CH), 1.45 (s, 9H, CMe_3), 1.21 (s, 6H, CMe), 1.21 (s, 6H, CMe), 0.91–0.74 (m, 2H, CH), 0.09 (dd, *J* = 9.5, 7.0 Hz, 1H, CH); ^{13}C NMR (100.6 MHz, $CDCl_3$) δ 154.7 (C=O), 83.4 (O*C*Me_2), 79.0 (O*C*Me_3), 51.5 (NCH), 45.6 (NCH_2), 35.6 (CH_2), 28.7 (C*Me*$_3$), 25.3 (C*Me*), 25.0 (C), 24.9 (C), 24.6 (C*Me*) (BCH not resolved); ^{11}B (128.4 MHz, $CDCl_3$) δ 32.5; MS (ESI) *m/z* 346 $(M + Na)^+$; HRMS (ESI) *m/z* calcd for $C_{17}H_{30}BNO_4$ $(M + Na)^+$ 346.2160, found 346.2165 (–0.6 ppm error).

Lab Book Reference: SY-2-77

***tert*-Butyl (1*R**,3*R**)-1-(6-methyl-4,8-dioxo-1,3,6,2-dioxazaborocan-2-yl)-5-azaspiro[2.4]heptane-5-carboxylate 1e**

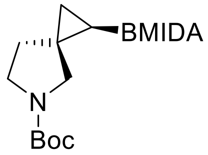

1e

MIDA (8.49 g, 57.7 mmol, 6.5 eq.) and $HC(OEt)_3$ (5.9 mL, 40.0 mmol, 4.5 eq.) were added to a stirred solution of pinacol boronate **S18** (2.87 g, 8.9 mmol, 1.0 eq.) in anhydrous DMSO (100 mL) at rt under Ar. The resulting mixture was stirred and heated at 100 °C for 48 h. After being allowed to cool to rt, saturated $NH_4Cl_{(aq)}$ (100 mL) was added and the mixture was extracted with EtOAc (3 × 100 mL). The combined organic extracts were washed with brine (100 mL), dried ($MgSO_4$) and evaporated under reduced pressure to give the crude product. Purification by flash column chromatography on silica with 80:20 CH_2Cl_2-acetone as eluent gave MIDA boronate **1e** (2.87 g, 92%) as an off-white solid, mp 200–203 °C; R_F (7:3 CH_2Cl_2-acetone) 0.26; IR (ATR) 2974, 2938, 2874, 1758 (C=O, ester), 1680 (C=O, Boc), 1410, 1342, 1296, 1167, 1116, 1026, 1002, 885 cm^{-1}; ^1H NMR (400 MHz, $CDCl_3$) (50:50 mixture of rotamers) δ 4.03 (d, J = 16.5 Hz, 1H, MeNC*H*), 3.95 (d, J = 16.5 Hz, 0.5H, MeNC*H*), 3.93 (d, J = 16.5 Hz, 0.5H, MeNC*H*), 3.84 (d, J = 16.5 Hz, 0.5H, MeNC*H*), 3.80 (d, J = 16.5 Hz, 0.5H, MeNC*H*), 3.76 (d, J = 16.5 Hz, 1H, MeNC*H*), 3.56–3.43 (m, 1H, NCH), 3.41 (d, J = 11.0 Hz, 1H, NCH), 3.39–3.29 (m, 1H NCH), 3.21 (d, J = 11.0, 0.5H, NCH), 3.20 (d, J = 11.0, 0.5H, NCH), 3.05 (s, 3H, NMe), 2.15–1.99 (m, 1H, CH), 1.53–1.47 (m, 1H, CH), 1.44 (s, 9H, CMe_3), 0.72 (dd, J = 9.5, 3.5 Hz, 1H, CH), 0.53–0.46 (m, 1H, CH), -0.01–-0.10 (m, 1H, BCH); ^{13}C NMR (100.6 MHz, $CDCl_3$) (rotamers) δ 168.3 (C=O, ester), 167.8 (C=O, ester), 154.7 (C=O, Boc), 79.1 (O*C*Me_3), 62.1 ($NCH_2C(O)$), 51.2 (NCH_2), 50.9 (NCH_2), 46.5 (NMe), 45.5 (NCH_2), 36.4 (CH_2), 36.0 (CH_2), 28.7 (C*Me*$_3$), 26.1 (C), 25.3 (C), 16.7 (CH_2), 16.3 (CH_2) (BCH not resolved); ^{11}B (128.4 MHz, $CDCl_3$) δ 11.6; HRMS (ESI) m/z calcd for $C_{16}H_{25}BN_2O_6$ [$(M + Na)^+$, 100] 375.1698, found 375.1701 (+0.1 ppm error).

Lab Book Reference: SY-2-137

***tert*-Butyl 6,6-dibromo-3-azabicyclo[3.1.0]hexane-3-carboxylate S19 and *tert*-butyl (3*R**,4*R**)-3-bromo-4-hydroxypyrrolidine-1-carboxylate S20**

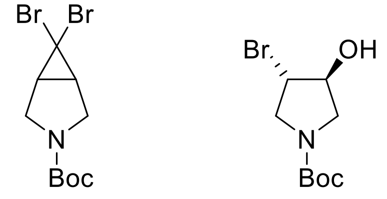

S19 **S20**

$CHBr_3$ (7.8 mL, 88.7 mmol, 3.0 eq.) was added dropwise over 1 h to a stirred solution of *tert*-butyl 2,5-dihydro-1H-pyrrole-1-carboxylate (5.00 g, 29.6 mmol, 1.0 eq.) and NaO*t*-pent (22.8 g, 0.21 mol, 7.0 eq.) in *n*-heptane (100 mL) at –10 °C under Ar. The resulting solution was stirred vigorously at –10 °C for 5 h. After being allowed to warm to rt, the solids were removed by filtration and washed with Et_2O (50 mL). The filtrate was poured into water (200 mL) and extracted with Et_2O (3 × 150 mL). The combined organics were dried ($MgSO_4$) and evaporated under reduced pressure to give the crude product (9.80 g) as a black viscous oil which contained a 60:40 mixture (by ^{1}H NMR spectroscopy) of dibromocyclopropane **19** and *N*-Boc pyrroline. The crude product was dissolved in DMSO (80 mL) and water (6 mL) and cooled to 0 °C. Then, NBS (5.80 g, 32.8 mmol, 2.4 eq.) was added portionwise at 0 °C. The resulting mixture was stirred at rt for 16 h. Then, water (50 mL) was added and the mixture was extracted with EtOAc (3 × 100 mL). The combined organics were washed with brine (100 mL), dried ($MgSO_4$) and evaporated under reduced pressure to give the crude product as a brown oil. Purification by flash column chromatography on silica with 10:90 EtOAc-hexane as eluent gave dibromocyclopropane **S19** (5.00 g, 47%) as a pale-yellow solid, mp 74–76 °C; R_F (20:80 EtOAc-hexane) 0.32; IR (ATR) 2975, 2932, 2881, 1694 (C=O), 1392, 1366, 1171, 1117, 881, 861, 769, 724, 552 cm^{-1}; ^{1}H NMR (400 MHz, $CDCl_3$) δ 3.66–3.51 (m, 4H, NCH), 2.44–2.34 (m, 2H, CH), 1.43 (s, 9H, CMe_3); ^{13}C NMR (100.6 MHz, $CDCl_3$) δ 153.7 (C=O), 80.0 (O*C*Me), 48.9 (NCH_2), 48.8 (NCH_2), 35.6 (CH), 34.9 (CBr_2), 34.8 (CH), 28.5 (C*Me*$_3$); MS (ESI) *m/z* 361 [(^{79,79}M + Na)$^+$, 100], HRMS ESI *m/z* calcd for $C_{10}H_{15}{}^{79}Br_2NO_2$ [(^{79,79}M + Na)$^+$, 100] 361.9362, found 361.9363 (–0.3 ppm error) and bromohydrin **S20** (2.3 g, 30%) as a brown solid, mp 42–44 °C; R_F (20:80 EtOAc-hexane) 0.10; IR (ATR) 3380 (br, OH), 2977, 2934, 2886, 1670, 1420, 1367, 1162, 1118, 960, 867, 770, 643, 553 cm^{-1}; ^{1}H NMR (400 MHz, $CDCl_3$) δ 4.50–4.45 (m, 1H, HOC*H*), 4.18–4.13 (m, 1H, BrCH), 4.07–3.98 (m, 1H, NCH), 3.93–3.69 (m, 2H, NCH), 3.40 (dd, *J* = 12.0, 6.0 Hz, 1H, NCH), 2.37 (br s, 1H, OH), 1.47 (s, 9H, CMe_3); ^{13}C NMR (100.6 MHz, $CDCl_3$) (rotamers) δ 154.8 (C=O), 80.4 (O*C*Me$_3$), 80.3 (O*C*Me$_3$), 77.1 (HOCH), 76.2 (HOCH), 53.1 (NCH_2), 52.5 (NCH_2), 51.6 (NCH_2), 51.3 (NCH_2), 50.7 (CHBr), 50.2 (CHBr), 28.6

(C*Me*$_3$); MS (ESI) *m/z* 288 [(^{79}M + Na)$^+$, 100], HRMS ESI *m/z* calcd for $C_9H_{16}{}^{79}BrNO_3$ [(^{79}M + Na)$^+$, 100] 288.0206, found 288.0206 (–0.1 ppm error) Spectroscopic data of dibromocyclopropane **S19**[7] and bromohydrin **S20**[8] consistent with those reported in the literature.

Lab Book Reference: SY-1-40

***tert*-Butyl (1*R**,5*S**,6*R**)-6-bromo-3-azabicyclo[3.1.0]hexane-3-carboxylate S21 and *tert*-Butyl (1*R**,5*S**,6*S**)-6-bromo-3-azabicyclo[3.1.0]hexane-3-carboxylate S22**

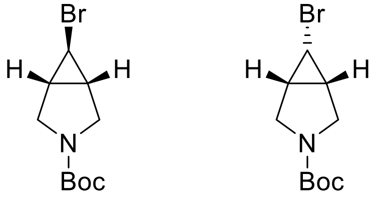

S21 **S22**

KO*t*-Bu (13.6 g, 0.12 mol, 6.0 eq.) was added portionwise to a stirred solution of dibromocyclopropane **S19** (6.9 g, 20.3 mmol, 1.0 eq.) and dimethyl phosphite (13.4 mL, 0.12 mol, 6.0 eq.) in anhydrous DMSO (120 mL) at 0 °C under Ar. The resulting solution was stirred at rt for 1.5 h. Then, Et_2O (50 mL, chilled to 0 °C) was added. Saturated $Na_2CO_{3(aq)}$ (50 mL) and water (200 mL) were added and the two layers were separated. The aqueous layer was extracted with Et_2O (3 × 150 mL). The combined organic layers were dried ($MgSO_4$) and evaporated under reduced pressure to give the crude product which contained a 70:30 mixture (by ^{1}H NMR spectroscopy) of monobromocyclopropanes **S21** and **S22**. Purification by flash column chromatography on silica using 10:90 EtOAc-hexane as eluent gave monobromocyclopropane **S21** (3.1 g, 58%) as a yellow oil, R_F (20:80 EtOAc-hexane) 0.40; IR (ATR) 2975, 2874, 1694 (C=O), 1478, 1376, 1365, 1333, 1223, 1169, 1047, 996, 885, 862, 773, 675, 547 cm^{-1}; ^{1}H NMR (400 MHz, $CDCl_3$) (50:50 mixture of rotamers) δ 3.71 (d, *J* = 11.5 Hz, 1H, NCH), 3.63 (d, *J* = 11.5 Hz, 1H, NCH), 3.39–3.30 (m, 2H, NCH), 2.55 (t, *J* = 2.5 Hz, 1H, BrCH), 1.97–1.89 (m, 2H, CH), 1.43 (s, 9H, CMe$_3$); ^{13}C NMR (100.6 MHz, $CDCl_3$) (rotamers) δ 154.7 (C=O), 79.9 (O*C*Me$_3$), 48.4 (NCH$_2$), 48.2 (NCH$_2$), 28.5 (C*Me*$_3$), 27.4 (CH), 26.6 (CH), 21.7 (BrCH); HRMS ESI *m/z* calcd for $C_{10}H_{16}{}^{79}BrNO_2$ (M + Na)$^+$ 284.0257, found 284.0258 (+0.4 ppm error) and monobromocyclopropane **S22** (1.2 g, 23%) as an off-white crystalline solid, mp 71–73 °C; R_F (20:80 EtOAc-hexane) 0.19; IR (ATR) 2976, 1676 (C=O), 1474, 1405, 1364, 1346, 1247, 1174, 1115, 933, 871, 778, 640, 543 cm^{-1}; ^{1}H NMR (400 MHz, $CDCl_3$) (50:50 mixture of rotamers) δ 3.66–3.53 (m, 3H, NCH), 3.48 (d, *J* = 11.0 Hz,

1H, NCH), 3.39 (t, J = 7.0 Hz, 1H, BrCH), 1.92–1.81 (m, 2H, CH), 1.44 (s, 9H, CMe$_3$); ^{13}C NMR (100.6 MHz, CDCl$_3$) (rotamers) δ 153.8 (C=O), 79.6 (O*C*Me$_3$), 47.3 (NCH$_2$), 47.1 (NCH$_2$), 31.5 (BrCH), 28.6 (C*Me*$_3$), 22.0 (CH), 21.3 (CH); HRMS ESI *m/z* calcd for $C_{10}H_{16}{}^{79}BrNO_2$ (M + Na)$^+$ 284.0257, found 284.0257 (+0.0 ppm error).

The structure of **S22** was confirmed by X-Ray crystallography (CCDC 2219555):

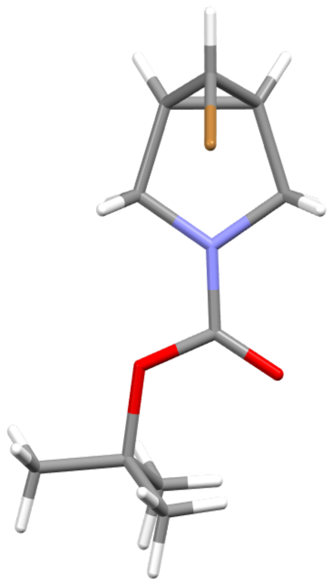

Lab Book Reference: SY-1-48

tert-Butyl (1_R_*,5_S_*,6_R_*)-6-(4,4,5,5-tetramethyl-1,3,2-dioxaborolan-2-yl)-3-azabicyclo[3.1.0]hexane-3-carboxylate S23

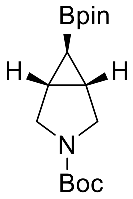

S23

n-BuLi (10.2 mL of a 2.30 M solution in hexane, 23.5 mmol, 1.5 eq.) was added dropwise to a stirred solution of monobromocyclopropane **S21** (4.10 g, 15.6 mmol, 1.0 eq.) in THF (150 mL) at –78 °C under Ar. The resulting solution was stirred at –78 °C for 1 h. Then, *i*-PrOBpin (4.8 mL, 23.5 mmol, 1.5 eq.) was added and the solution was stirred at –78 °C for 10 min. After being allowed to warm to rt, saturated $NH_4Cl_{(aq)}$ (100 mL) was added and the two layers were separated. The aqueous layer was extracted with Et_2O (3 × 100 mL). The combined organic layers were dried ($MgSO_4$) and evaporated under reduced pressure to give the crude product. Purification by flash column chromatography on silica using 40:60 to 60:40 Et_2O-hexane as eluent gave boronic pinacol ester **S23** (3.54 g, 73%) as an off-white solid, mp 106–109 °C; R_F (40:60 Et_2O-hexane) 0.36; IR (ATR) 2977, 2931, 2868, 1699 (C=O), 1394, 1320, 1171, 1144, 1112, 858 cm^{-1}; ^1H NMR (400 MHz, $CDCl_3$) (50:50 mixture of rotamers) δ 3.61 (d, *J* = 11.0 Hz, 1H, NCH), 3.53 (d, *J* = 11.0 Hz, 1H, NCH), 3.37–3.27 (m, 2H, NCH), 1.68–1.59 (m, 2H, CH), 1.41 (s, 9H, CMe_3), 1.22 (s, 12H, CMe_2), –0.28 (t, *J* = 4.5 Hz, 1H, BCH); ^{13}C NMR (100.6 MHz, $CDCl_3$) (rotamers) δ 155.4 (C=O), 83.3 (O*C*Me_2), 79.3 (O*C*Me_3), 48.7 (NCH_2), 48.4 (NCH_2), 28.6 (C*Me*$_3$), 24.8 (C*Me*$_2$), 22.3 (CH), 21.5 (CH) (BCH resonance not resolved); ^{11}B (128.4 MHz, $CDCl_3$) δ 30.6; HRMS ESI *m/z* calcd for $C_{16}H_{29}BNO_4$ $(M + Na)^+$ 332.2004, found 332.2004 (+0.7 ppm error).

Lab Book Reference: SY-1-49

***tert*-Butyl (1*R**,5*S**,6*R**)-6-(6-methyl-4,8-dioxo-1,3,6,2-dioxazaborocan-2-yl)-3-azabicyclo[3.1.0]hexane-3-carboxylate 1h**

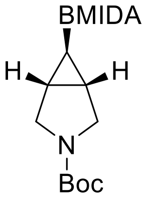

1h

MIDA (10.6 g, 72.0 mmol, 6.5 eq.) and $HC(OEt)_3$ (8.3 mL, 49.8 mmol, 4.5 eq.) were added to a stirred solution of boronic pinacol ester **S23** (3.4 g, 11.1 mmol, 1.0 eq.) in anhydrous DMSO (80 mL) at rt under Ar. The resulting solution was stirred and heated at 100 °C for 48 h. After being allowed to cool to rt, saturated $NH_4Cl_{(aq)}$ (200 mL) was added and the mixture was extracted with EtOAc (3 × 300 mL). The combined organic extracts were washed with brine (200 mL), dried ($MgSO_4$) and evaporated under reduced pressure to give the crude product. Purification by flash column chromatography on silica with 20:80 to 60:40 acetone-CH_2Cl_2 as eluent gave MIDA boronate **1h** (2.97 g, 79%) as a white solid, mp 146–150 °C; R_F (20:80 acetone-CH_2Cl_2) 0.36; IR (ATR) 2974, 2934, 2871, 1753 (C=O, ester), 1680 (C=O, Boc), 1391, 1295, 1174, 1112, 1028, 988, 963, 868 cm^{-1}; ^1H NMR (400 MHz, $CDCl_3$) (50:50 mixture of rotamers) δ 3.85 (d, *J* = 16.5 Hz, 2H, NCHC=O), 3.74 (d, *J* = 16.5 Hz, 1H, NCHC=O), 3.70 (d, *J* = 16.5 Hz, 1H, NCHC=O), 3.58 (d, *J* = 11.0 Hz, 1H, NCH), 3.54 (d, *J* = 11.0 Hz, 1H, NCH), 3.40–3.31 (m, 2H, NCH), 3.03 (s, 3H, NMe), 1.59–1.53 (m, 1H, CH), 1.43 (s, 9H, CMe_3), 1.41–1.35 (m, 1H, CH), –0.45 (t, *J* = 5.0 Hz, 1H, BCH); ^{13}C NMR (100.6 MHz, $CDCl_3$) (rotamers) δ 166.7 (C=O, ester), 155.6 (C=O, Boc), 79.6 (O*C*Me_3), 62.2 ($NCH_2C(O)$), 62.1 ($NCH_2C(O)$), 48.6 (NCH_2), 48.2 (NCH_2), 46.6 (NMe), 28.6 (C*Me*$_3$), 20.3 (CH), 19.0 (CH) (BCH resonance not resolved); ^{11}B (128.4 MHz, $CDCl_3$) δ 10.9; MS (ESI) *m/z* 361 [$(M + Na)^+$, 100], HRMS ESI *m/z* calcd for $C_{15}H_{23}BN_2O_6$ [$(M + Na)^+$, 100] 361.1541, found 338.1545 (–0.4 ppm error).

The structure was confirmed by X-Ray crystallography (CCDC 2219556):

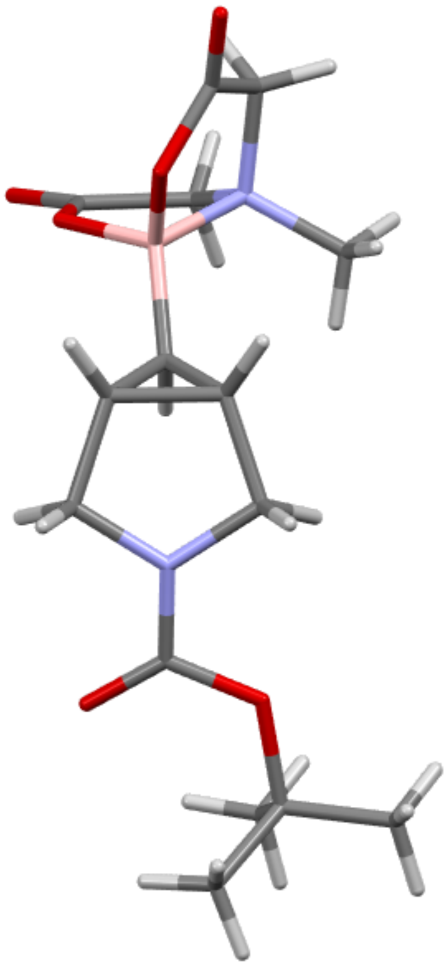

Lab Book Reference: SY-1-50

tert-Butyl 7,7-dibromo-2-azabicyclo[4.1.0]heptane-2-carboxylate S26

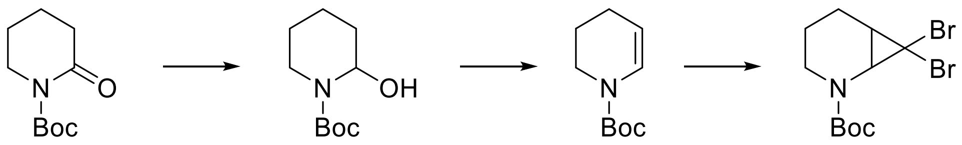

S24 **S25** **S26**

DIBAL-H (107.5 mL of a 1.0 M solution in hexane, 107.5 mmol, 1.5 eq.) was added dropwise to a stirred solution of 1-*N*-Boc-2-piperidone (14.28 g, 71.7 mmol, 1.0 eq.) in THF (200 mL) at –78 °C under Ar. The resulting solution was stirred at –78 °C for 2 h. Then, saturated potassium sodium tartrate$_{(aq)}$ (100 mL) was added. The mixture was allowed to warm to rt and stirred at rt for 16 h. H_2O (100 mL) was added, and the mixture was extracted with EtOAc (3 × 150 mL). The combined organic extracts were dried ($MgSO_4$) and evaporated under reduced pressure to give the crude hydroxy piperidine **S24** as a colourless oil. Then, *p*-TsOH•H_2O (177 mg, 1.05 mmol, 1.3 mol%) was added to a stirred solution of crude hydroxy piperidine **S24** in toluene (120 mL). The solution was connected to a Dean-Stark apparatus and stirred and heated at reflux for 30 min. After being allowed to cool to rt, the solvent was evaporated under reduced pressure to give crude *N*-Boc enamide **S25**. Then, $CHBr_3$ (18.4 mL, 210.0 mmol, 3.0 eq.) and $BnEt_3N^+Cl^-$ (2.87 g, 12.6 mmol, 0.2 eq.) were added to a stirred solution of *N*-Boc enamide **S25** (12.83 g, 70.0 mmol, 1.0 eq.) in CH_2Cl_2 (200 mL) at rt under Ar. A solution of NaOH (109.2 g, 2.7 mol, 39.0 eq.) in H_2O (110 mL) was added and the resulting mixture was stirred vigorously and heated at 50 °C for 16 h. After being allowed to cool to rt, the mixture was evaporated under reduced pressure to remove CH_2Cl_2. Then, H_2O (100 mL) was added, and the mixture was extracted with Et_2O (3 × 150 mL). The combined organic extracts were dried ($MgSO_4$) and evaporated under reduced pressure to give the crude product. Purification by flash chromatography on silica with 90:10 hexane-Et_2O as eluent gave dibromocyclopropane **S26** (20.18 g, 81%) as a yellow solid, mp 48–50 °C; R_F (9:1 hexane-Et_2O) 0.19; IR (ATR) 2973, 2938, 2874, 1701 (C=O), 1366, 1352, 1164, 1135, 758 cm^{-1}; ^{1}H NMR (400 MHz, $CDCl_3$) (85:15 mixture of rotamers) δ 3.43 (ddd, *J* = 12.5, 8.0, 4.0 Hz, 0.85H, NCH), 3.37 (d, *J* = 9.5 Hz, 0.15H, NCH), 3.29 (d, *J* = 9.0 Hz, 0.85H, NCH), 3.27–3.18 (m, 0.15H, NCH), 3.08 (ddd, *J* = 12.5, 6.5, 4.0 Hz, 0.15H, NCH), 2.90 (ddd, *J* = 12.5 8.0, 4.0 Hz, 0.85H, NCH), 2.15–2.01 (m, 2H, CH), 1.79–1.67 (m, 1H, CH), 1.61–1.57 (m, 1H, CH), 1.56 (s, 7.65H, CMe_3), 1.50 (s, 1.35H, CMe_3), 1.49–1.36 (m, 1H, CH); ^{13}C NMR (100.6 MHz, $CDCl_3$) δ 156.0 (C=O), 80.6 (O*C*Me_3), 40.8 (NCH), 40.4 (NCH_2), 36.9 (CBr_2), 29.6 (CH), 28.5 (C*Me*$_3$), 21.3 (CH_2), 19.2 (CH_2); MS (ESI) *m/z* 376 [(^{79,79}M + Na)$^+$; HRMS (ESI) *m/z* calcd for $C_{11}H_{17}{}^{79}Br_2NO_2$ [(^{79,79}M + Na)$^+$ 375.9518, found 375.9518 (0.0 ppm error).

Lab Book Reference: SY-3-71

tert-Butyl (1_R_*,6_S_*,7_R_*)-7-bromo-2-azabicyclo[4.1.0]heptane-2-carboxylate S27

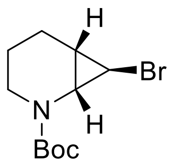

S27

KO*t*-Bu (37.93 g, 0.338 mol, 6.0 eq.) was added portionwise to a stirred solution of dibromocyclopropane **S26** (20.00 g, 0.563 mol, 1.0 eq.) and dimethyl phosphite (31.0 mL, 0.338 mol, 6.0 eq.) in anhydrous DMSO (250 mL) at 0 °C under Ar. The resulting solution was stirred at rt for 1.5 h. Then, Et_2O (120 mL) and saturated $NaHCO_{3(aq)}$ (120 mL) were added. A white precipitate formed, and additional H_2O (~100 mL) was added to redissolve the solids. The two layers separated, and the aqueous layer was extracted with Et_2O (3 × 150 mL). The combined organic layers were dried ($MgSO_4$) and evaporated under reduced pressure to give the crude product. Purification by flash column chromatography on silica with 90:10 hexane-Et_2O as eluent gave monobromocyclopropane **S27** (13.41 g, 86%) as a colourless oil, R_F (9:1 hexane-EtOAc) 0.38; IR (ATR) 2979, 2934, 2867, 1696 (C=O), 1365, 1159, 1130, 1032, 894, 894, 859, 772 cm^{-1}; ^{1}H NMR (400 MHz, $CDCl_3$) (80:20 mixture of rotamers) δ 3.77 (br d, *J* = 12.5 Hz, 0.8H, NCH), 3.59 (br d, *J* = 12.5 Hz, 0.2H, NCH), 3.21 (br d, *J* = 9.0 Hz, 0.2H, NCH), 3.08 (d, *J* = 9.0 Hz, 0.8H, NCH), 2.61–2.57 (m, 0z.4H, NCH), 2.56–2.52 (m, 0.8H, NCH), 2.49–2.38 (m, 0.8H, CHBr), 2.03–1.93 (m, 1H, CH), 1.76–1.66 (m, 1H, CH), 1.66–1.53 (m, 2H, CH), 1.50 (s, 7.2H, CMe_3), 1.46 (s, 1.8H, CMe_3), 1.21–1.06 (m, 1H, CH); ^{13}C NMR (100.6 MHz, $CDCl_3$) (rotamers) δ 156.2 (C=O), 80.1 (O*C*Me$_3$), 41.5 (NCH), 39.8 (NCH), 37.6 (NCH_2), 37.4 (NCH_2), 28.6 (C*Me*$_3$), 23.4 (CH_2), 22.9 (CHBr), 22.3 (CH), 19.6 (CH_2); MS (ESI) *m/z* 298 [(^{79}M + Na)$^+$; HRMS (ESI) *m/z* calcd for $C_{11}H_{18}{}^{79}BrNO_2$ [(^{79}M + Na)$^+$ 298.0413, found 298.0410 (+0.9 ppm error).

Lab Book Reference: SY-3-73

***tert*-Butyl (1*R**,6*S**,7*S**)-7-(4,4,5,5-tetramethyl-1,3,2-dioxaborolan-2-yl)-2-azabicyclo[4.1.0]heptane-2-carboxylate S28**

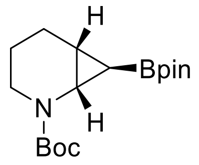

S28

n-BuLi (47.9 mL of a 1.50 M solution in hexanes, 71.9 mmol, 1.5 eq.) was added dropwise to a stirred solution of monobromocyclopropane **S27** (13.23 g, 47.9 mmol, 1.0 eq.) in THF (250 mL) at –78 °C under Ar. The resulting solution was stirred at –78 °C for 1 h. Then, *i*-PrO-Bpin (14.7 mL, 71.9 mmol, 1.5 eq.) was added and the solution was stirred at –78 °C for 10 min. After being allowed to warm to rt, saturated $NH_4Cl_{(aq)}$ (200 mL) was added, and the two layers were separated. The aqueous layer was extracted with Et_2O (3 × 120 mL). The combined organic layers were washed with brine (120 mL), dried ($MgSO_4$) and evaporated under reduced pressure to give the crude product. Purification by flash column chromatography on silica with 90:10 to 80:20 to 70:30 hexane-Et_2O as eluent gave cyclopropyl−Bpin **S28** (9.32 g, 60%) as a white solid, R_F (9:1 hexane-EtOAc) 0.41; IR (ATR) 2978, 2934, 2867, 1697 (C=O), 1398, 1366, 1319, 1167, 1143, 962, 893, 857, 778 cm^{-1}; ^1H NMR (400 MHz, $CDCl_3$) (90:10 mixture of rotamers) δ 3.77 (ddd, *J* = 12.5, 3.5, 3.5 Hz, 0.9H, NCH), 3.63–3.54 (m, 0.1H, NCH), 3.13–3.06 (m, 0.1H, NCH), 2.93 (dd, *J* = 8.0, 3.5 Hz, 0.9H, NCH), 2.72–2.61 (m, 0.1H, NCH), 2.55 (ddd, *J* = 12.5, 12.5, 2.5 Hz, 0.9H, NCH), 1.91–1.76 (m, 2H, CH), 1.59–1.52 (m, 1H, CH), 1.46 (s, 9H, CMe_3), 1.43–1.35 (m, 1H, CH), 1.35–1.26 (m, 1H, CH), 1.212 (s, 6H, CMe), 1.206 (s, 6H, CMe), –0.17 (dd, *J* = 7.5, 3.5 Hz, 1H, BCH); ^{13}C NMR (100.6 MHz, $CDCl_3$) δ 156.6 (C=O), 83.1 (O*C*Me_2), 79.3 (O*C*Me_3), 40.8 (NCH_2), 35.0 (NCH), 28.6 (C*Me*$_3$), 25.0 (C*Me*), 24.6 (C*Me*), 21.5 (CH_2), 21.3 (CH_2), 18.0 (CH), (BCH not resolved); ^{11}B (128.4 MHz, $CDCl_3$) δ 32.2; MS (ESI) *m/z* 346 $(M + Na)^+$; HRMS (ESI) *m/z* calcd for $C_{17}H_{30}BNO_4$ $(M + Na)^+$ 346.2160, found 346.2164 (–0.5 ppm error).

Lab Book Reference: SY-3-75

***tert*-Butyl (1*R**,6*S**,7*S**)-7-(6-methyl-4,8-dioxo-1,3,6,2-dioxazaborocan-2-yl)-2-azabicyclo [4.1.0]heptane-2-carboxylate 1i**

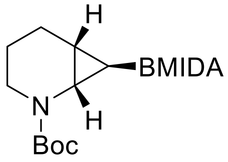

1i

MIDA (11.82 g, 80.3 mmol, 6.5 eq.) and $HC(OEt)_3$ (9.3 mL, 55.6 mmol, 4.5 eq.) were added to a stirred solution of pinacol boronate **S28** (4.00 g, 12.4 mmol, 1.0 eq.) in anhydrous DMSO (80 mL) at rt under Ar. The resulting suspension was stirred and heated at 100 °C for 48 h. After being allowed to cool to rt, saturated $NH_4Cl_{(aq)}$ (50 mL) was added, and the mixture was extracted with EtOAc (3 × 50 mL). The combined organic extracts were washed with brine (50 mL), dried ($MgSO_4$) and evaporated under reduced pressure to give the crude product. Purification by flash column chromatography on silica with 95:5 to 80:20 CH_2Cl_2-acetone as eluent gave MIDA boronate **1i** (3.92 g, 90%) as an off-white solid, mp 189-192 °C; R_F (9:1 CH_2Cl_2-acetone) 0.21; IR (ATR) 1758 (C=O, ester), 1675 (C=O, Boc), 1399, 1283, 1168, 1122, 995, 965, 915, 732 cm^{-1}; ^1H NMR (400 MHz, $CDCl_3$) δ 4.62 (d, *J* = 16.0 Hz, 1H, NCHC=O), 3.78 (d, *J* = 16.0 Hz, 1H, NCHC=O), 3.74–3.70 (m, 2H, MeNC*H*), 3.60 (ddd, *J* = 12.5, 4.0, 4.0 Hz, 1H, NCH), 3.15 (s, 3H, NMe), 2.63 (ddd, *J* = 12.5, 12.5, 2.5 Hz, 1H, NCH), 2.46 (dd, *J* = 8.0, 4.0 Hz, 1H, NCH), 1.93–1.77 (m, 2H, CH), 1.60–1.54 (m, 1H, CH), 1.42 (s, 9H, CMe_3), 1.40–1.35 (m, 1H, CH), 1.34–1.25 (m, 1H, CH), –0.38 (dd, *J* = 7.5, 4.0 Hz, 1H, BCH); ^{13}C NMR (100.6 MHz, $CDCl_3$) δ 168.7 (C=O, ester), 167.6 (C=O, ester), 156.6 (C=O, Boc), 79.6 (O*C*Me_3), 61.9 (MeN*C*H_2), 61.4 (MeN*C*H_2), 45.1 (NMe), 42.1 (NCH_2), 32.0 (NCH), 28.5 (C*Me*$_3$), 21.7 (CH_2), 21.1 (CH_2), 14.3 (CH) (BCH not resolved); ^{11}B (128.4 MHz, $CDCl_3$) δ 10.9; MS (ESI) *m/z* 375 $(M + Na)^+$; HRMS (ESI) *m/z* calcd for $C_{16}H_{25}BN_2O_6$ $(M + Na)^+$ 375.1698, found 375.1703 (−0.5 ppm error).

The structure was confirmed by X-Ray crystallography (CCDC 2219554):

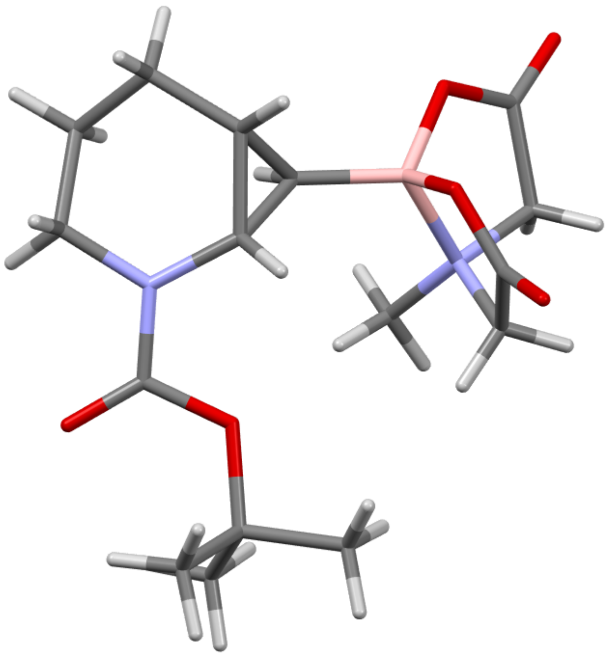

Lab Book Reference: SY-3-79

***tert*-Butyl 1-(4-methoxyphenyl)-2-azabicyclo[3.1.0]hexane-2-carboxylate 12**

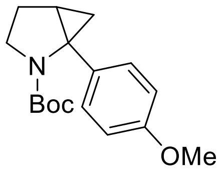

12

Using general procedure A, *N*-Boc MIDA boronate **1a** (400 mg, 1.18 mmol, 1.0 eq.), Cs_2CO_3 (2.31 g, 7.10 mmol, 6.0 eq.), PCy_3 (100 mg, 0.355 mmol, 0.3 eq.), $Pd(OAc)_2$, (40 mg, 0.18 mmol, 0.15 eq.) and 4-bromoanisole (208 µL, 1.66 mmol, 1.4 eq.) in toluene (13 mL) and H_2O (0.7 mL) gave the crude product. Purification by flash column chromatography on silica with 80:20 hexane-Et_2O as eluent gave arylated pyrrolidine **12** (264 mg, 77%) as a white solid, mp 69–71 °C; R_F (1:1 hexane-Et_2O) 0.48; IR (ATR) 1688 (C=O), 1516, 1363, 1244 cm^{-1}; ^1H NMR (400 MHz, $CDCl_3$) δ 7.19 (d, *J* = 9.0 Hz, 2H, Ar), 6.81 (d, *J* = 9.0 Hz, 2H, Ar), 3.86 (ddd, *J* = 11.5, 9.5, 6.0 Hz, 1H, NCH), 3.77 (s, 3H, OMe), 3.60–3.48 (m, 1H, NCH), 2.29 (dddd, *J* = 13.0, 9.5, 6.0, 6.0 Hz, 1H, CH), 1.93 (dddd, *J* = 13.0, 9.0, 6.0, 5.5 Hz, 1H, CH), 1.69 (dd, *J* = 9.0, 5.5 Hz, 1H, CH), 1.53–1.47 (m, 1H, CH), 1.23 (br s, 9H, CMe_3), 0.95 (dd, *J* = 5.5, 5.5 Hz, 1H, CH); ^{13}C NMR (100.6 MHz, $CDCl_3$) δ 158.1 (*ipso*-Ar), 156.1 (C=O), 133.6 (*ipso*-Ar), 127.7 (Ar), 113.4 (Ar), 79.4 (O*C*Me_3), 55.3 (OMe), 50.1 (NCH_2), 49.3 (N*C*Ar), 29.5 (CH), 28.3 (C*Me*$_3$), 26.7 (CH_2), 22.0 (CH_2); MS (EI) *m/z* 312 $(M + Na)^+$; HRMS (ESI) *m/z* calcd for $C_{17}H_{23}NO_3$ $(M + Na)^+$ 312.1570, found 312.1571 (–0.2 ppm error).

Lab book reference HFK5-041

N-Boc MIDA boronate **1a** (59 mg, 0.17 mmol, 1.0 eq.), $Pd(OAc)_2$, (2 mg, 0.0087 mmol, 0.5 eq.), SPhos (7 mg, 0.017 mmol, 0.1 eq.) and 4-bromoanisole (33 µL, 0.26 mmol, 1.5 eq.) were combined in dioxane (2.2 mL) at rt under Ar. The resulting mixture was stirred at rt for 10 min (with purging of the head-space with Ar). Then, 2.0 M $K_3PO_{4(aq)}$ (0.64 mL, 1.28 mmol, 7.5 eq.) (which had been degassed by sparging with Ar for 30 min) was added. The resulting mixture was stirred and heated in a sealed tube at 100 °C for 18 h under Ar. The mixture was then allowed to cool to rt and EtOAc (10 mL) was added. The organic layer was washed with saturated $NaHCO_{3(aq)}$ (10 mL), dried (Na_2SO_4) and evaporated under reduced pressure to give the crude product which contained a 50:50 mixture (by ^1H NMR spectroscopy) of **12** and **13**. Purification by flash column chromatography on silica with 95:5 to 90:10 to 80:20 hexane-Et_2O as eluent gave cyclopropane **13** (4 mg, 13%) as a colourless oil, R_F (4:1

hexane-Et_2O) 0.30; IR (ATR) 1692 (C=O), 1406, 1168, 1105 cm^{-1}; ^1H NMR (400 MHz, $CDCl_3$) δ 3.69-3.55 (m, 1H, NCH), 3.47-3.18 (m, 1H, NCH), 2.94-2.86 (m, 1H, NCH), 2.12-1.97 (m, 1H, CH), 1.92-1.86 (m, 1H, CH), 1.51-1.39 (m, 1H, CH), 1.45 (s, 9H, CMe_3), 0.69-0.59 (m, 1H, CH), 0.52-0.49 (m, 1H, CH); ^{13}C NMR (100.6 MHz, $CDCl_3$) (rotamers) δ 155.4 (C=O), 79.4 (O*C*Me_3), 43.8 (NCH_2), 35.6 (NCH), 28.6 (C*Me*$_3$), 26.5 (CH_2), 25.7 (CH_2), 15.9 (CH), 15.1 (CH), 11.0 (CH_2); MS (EI) *m/z* 206 $(M + Na)^+$; HRMS (ESI) *m/z* calcd for $C_{10}H_{17}NO_2$ $(M + Na)^+$ 206.1151, found 206.1157 (–2.9 ppm error) and arylated pyrrolidine **12** (9 mg, 18%) as a white solid.

Lab book reference HFK5-019

N-Boc MIDA boronate **1a** (90 mg, 0.27 mmol, 1.0 eq.), K_2CO_3 (99 mg, 0.71 mmol, 2.7 eq.), RuPhos (16 mg, 0.035 mmol, 0.13 eq.), $Pd(OAc)_2$, (4 mg, 0.017 mmol, 0.066 eq.) and 4-bromoanisole (50 μL, 0.40 mmol, 1.5 eq.) were combined in toluene (1.7 mL) and H_2O (0.3 mL) at rt under Ar. The resulting mixture was degassed with Ar for 15 min and then stirred and heated in a sealed tube at 100 °C for 18 h under Ar. The mixture was then allowed to cool to rt and the solids were removed by filtration through Celite. EtOAc (10 mL) was added and the mixture was washed with H_2O (10 mL). The organic layer was dried (Na_2SO_4) and evaporated under reduced pressure to give the crude product which contained a 50:50 mixture (by ^1H NMR spectroscopy) of **12** and **13**. Purification by flash column chromatography on silica with 95:5 to 90:10 hexane-Et_2O as eluent gave cyclopropane **13** (10 mg, 20%) as a colourless oil and arylated pyrrolidine **12** (37 mg, 48%) as a white solid.

Lab book reference HFK5-030

***tert*-Butyl 1-(2-methoxyphenyl)-2-azabicyclo[3.1.0]hexane-2-carboxylate 14**

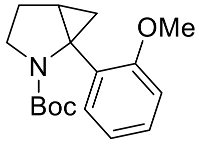

14

Using general procedure A, *N*-Boc MIDA boronate **1A** (90 mg, 0.27 mmol, 1.0 eq.), Cs_2CO_3 (520 mg, 1.60 mmol, 6.0 eq.), PCy_3 (22 mg, 0.080 mmol, 0.3 eq.), $Pd(OAc)_2$, (9 mg, 0.040 mmol, 0.15 eq.) and 2-bromoanisole (46 µL, 0.37 mmol, 1.4 eq.) in toluene (3 mL) and H_2O (0.2 mL) gave the crude product. Purification by flash column chromatography on silica with 80:20 hexane-Et_2O as eluent gave arylated pyrrolidine **14** (56 mg, 73%) as a white solid, mp 78–80 °C; R_F (1:1 hexane-Et_2O) 0.48; IR (ATR) 1685 (C=O), 1390, 1363, 751 cm^{-1}; ^1H NMR (400 MHz, $CDCl_3$) δ 7.32–7.26 (m, 1H, Ar), 7.21 (ddd, J = 8.0, 8.0, 2.0 Hz, 1H, Ar), 6.87 (dd, J = 8.0, 8.0 Hz, 1H, Ar), 6.82 (dd, J = 8.0, 1.0 Hz, 1H, Ar), 4.04–3.91 (m, 1H, NCH), 3.82 (s, 3H, OMe), 3.59–3.53 (m, 1H, NCH), 2.40–2.31 (m, 1H, CH), 1.96–1.88 (m, 1H, CH), 1.59–1.52 (m, 2H, CH), 1.21 (s, 9H, CMe_3), 0.90–0.88 (m, 1H, CH); ^{13}C NMR (100.6 MHz, $CDCl_3$) δ 158.9 (*ipso*-Ar), 155.6 (C=O), 131.7 (Ar), 128.8 (*ipso*-Ar), 128.3 (Ar), 119.8 (Ar), 110.2 (Ar), 79.1 ($OCMe_3$), 55.6 (OMe), 50.6 (NCH_2), 46.9 (N*C*Ar), 28.4 (CMe_3), 27.4 (CH), 27.0 (CH_2), 22.9 (CH_2); MS (EI) m/z 312 $(M + Na)^+$; HRMS (ESI) m/z calcd for $C_{17}H_{23}NO_3$ $(M + Na)^+$ 312.1570, found 312.1570 (–0.1 ppm error).

Lab book reference HFK5-039

***tert*-Butyl (1*S*,5*R*)-1-(2-methoxyphenyl)-2-azabicyclo[3.1.0]hexane-2-carboxylate (*S*,*R*)-14**

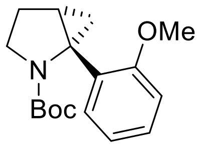

(*S*,*R*)-**14**

Using general procedure A, *N*-Boc BIDA boronate **1a'** (90 mg, 0.18 mmol, 1.0 eq.), Cs_2CO_3 (352 mg, 1.08 mmol, 6.0 eq.), PCy_3 (15 mg, 0.054 mmol, 0.3 eq.), 2-bromoanisole (32 μL, 0.252 mmol, 1.4 eq.) and $Pd(OAc)_2$ (6 mg, 0.027 mmol, 0.15 eq.) in toluene (3.03 mL) and H_2O (0.16 mL) gave the crude product as a yellow oil. Purification by flash column chromatography on silica with 95:5-90:10 hexane-EtOAc as eluent gave aryl pyrrolidine (*S,R*)-**14** (36 mg, 69%) as a yellow oil. $[\alpha]_D$ –6.5 (*c* 0.65 in $CHCl_3$), [lit.,[7] $[\alpha]_D$ –6.9 (c 0.65 in $CHCl_3$), for (*S,R*)-**14**, 99:1 er].

Lab book reference RA 1-092

***tert*-Butyl (1*R*,5*S*)-1-(2-methoxyphenyl)-2-azabicyclo[3.1.0]hexane-2-carboxylate (*R*,*S*)-14**

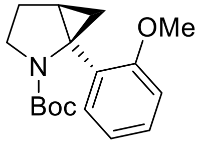

(*R*,*S*)-**14**

Using general procedure A, *N*-Boc BIDA boronate **1a''** (90 mg, 0.18 mmol, 1.0 eq.), Cs_2CO_3 (352 mg, 1.08 mmol, 6.0 eq.), PCy_3 (15 mg, 0.054 mmol, 0.3 eq.), 2-bromoanisole (32 μL, 0.252 mmol, 1.4 eq.) and $Pd(OAc)_2$ (6 mg, 0.027 mmol, 0.15 eq.) in toluene (3.03 mL) and H_2O (0.16 mL) gave the crude product as a yellow oil. Purification by flash column chromatography on silica with 95:5-90:10 hexane-EtOAc as eluent gave aryl pyrrolidine (*R*,*S*)-**14** (36 mg, 69%) as a yellow oil; $[\alpha]_D$ +7.3 (*c* 0.65 in $CHCl_3$), [lit.,[7] $[\alpha]_D$ –6.9 (c 0.65 in $CHCl_3$), for (*S,R*)-**14**, 99:1 er].

Lab book reference RA 1-087

***tert*-Butyl 1-(pyrimidin-5-yl)-2-azabicyclo[3.1.0]hexane-2-carboxylate 15**

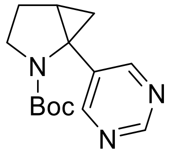

15

Using general procedure A, MIDA boronate **1a** (90 mg, 0.27 mmol, 1.0 eq.), 5-bromopyrimidine (59 mg, 0.37 mmol, 1.4 eq.), $Pd(OAc)_2$ (9.0 mg, 0.04 mmol, 0.15 eq.), PCy_3 (22 mg, 0.08 mmol, 0.30 eq.) and Cs_2CO_3 (520 mg, 1.60 mmol, 6.0 eq.) were reacted in toluene (3.03 mL) and H_2O (0.16 mL) to give the crude product. Purification by flash column chromatography on silica with Et_2O as eluent gave pyrimidine **15** (59 mg, 85%) as a colourless solid, mp 93–94 °C; R_F (Et_2O) 0.13; IR (ATR) 2974, 1693 (C=O), 1559, 1417, 1386, 1363, 1249, 1164, 1142, 1082, 1007, 863, 772, 726, 631 cm^{-1}; ^1H NMR (400 MHz, $CDCl_3$) δ 9.00 (s, 1H, Ar), 8.57 (s, 2H, Ar), 3.82 (ddd, *J* = 11.0, 10.5, 5.0 Hz, 1H, NCH), 3.60–3.41 (m, 1H, NCH), 2.38–2.26 (m, 1H, CH), 2.00 (ddd, *J* = 14.0, 9.0, 5.0 Hz, 1H, CH), 1.79–1.71 (m, 1H, CH), 1.71–1.60 (m, 1H CH), 1.36–1.06 (m, 10H, CMe_3, CH); ^{13}C NMR (100.6 MHz, $CDCl_3$) δ 156.5 (Ar), 155.9 (C=O), 154.8 (Ar), 135.1 (*ipso*-Ar), 80.5 (O*C*Me₃), 49.4 (NCH_2), 45.8 (NC), 30.4 (CH), 28.3 (C*Me*₃), 26.3 (CH_2), 21.1 (CH_2); HRMS (ESI) *m/z* calcd for $C_{14}H_{20}N_3O_2$ $(M + H)^+$ 262.1550, found 262.1549 (+0.6 ppm error).

Lab book reference JRD_VIII_97, JRD_VIII_98

***tert*-Butyl 1-(2-methoxypyrimidin-5-yl)-2-azabicyclo[3.1.0]hexane-2-carboxylate 16**

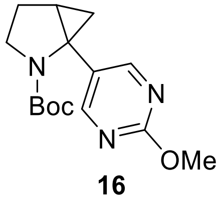

16

Using general procedure A, *N*-Boc MIDA boronate **1a** (90 mg, 0.27 mmol, 1.0 eq.), Cs_2CO_3 (520 mg, 1.6 mmol, 6.0 eq.), PCy_3 (22.4 mg, 0.0798 mmol, 0.3 eq.), 5-bromo-2-methoxypyrimidine (70.5 mg, 0.373 mmol, 1.4 eq.) and $Pd(OAc)_2$ (8.96 mg, 0.04 mmol, 0.15 eq.) in toluene (3.03 mL) and H_2O (0.16 mL) gave the crude product as a yellow oil. Purification by flash column chromatography on silica with 9:1 CH_2Cl_2-EtOAc as eluent gave aryl pyrrolidine **16** (55 mg, 70%) as a white solid, mp 100–102 °C; R_F

(7:3 hexane-EtOAc) 0.21; IR (ATR) 2974, 1692 (C=O), 1472, 1162 cm^{-1}; ^{1}H NMR (400 MHz, $CDCl_3$) δ 8.40 (s, 2H, Ar), 3.97 (s, 3H, OMe), 3.83 (ddd, *J* = 11.5, 9.5, 5.5 Hz, 1H, NCH_2), 3.56–3.42 (m, 1H, NCH), 2.41–2.26 (m, 1H, NCH_2C*H*), 1.99 (dddd, *J* = 13.0, 9.0, 5.5, 1.0 Hz, 1H, NCH_2C*H*), 1.65–1.54 (m, 2H, $NCCH_2$), 1.27 (s, 9H, CMe_3), 1.09–1.01 (m, 1H, NCCH); ^{13}C NMR (100.6 MHz, $CDCl_3$) δ 164.5 (C=O), 158.2 (Ar), 156.0 (*ipso*-Ar), 128.1 (*ipso*-Ar), 80.3 (*C*Me_3), 55.0 (OMe), 49.5 (NCH_2), 45.3 (NC), 28.4 (C*Me*$_3$), 26.3 (NC*C*H), 20.8 (NC*C*H_2); MS (ESI) *m/z* 314 (M + Na)$^+$]; HRMS *m/z* calcd for $C_{15}H_{21}N_3O_3$ (M + Na)$^+$ 314.1475, found 314.1473 (+0.8 ppm error).

Lab book reference RA 1-008

tert-Butyl 1-(5-fluoro-3-methylpyridin-2-yl)-2-azabicyclo[3.1.0]hexane-2-carboxylate 17

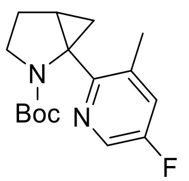

17

Using general procedure A, *N*-Boc MIDA boronate **1a** (90 mg, 0.27 mmol, 1.0 eq.), Cs_2CO_3 (520 mg, 1.6 mmol, 6.0 eq.), PCy_3 (22.4 mg, 0.0798 mmol, 0.3 eq.), 2-bromo-5-fluoro-3-methylpyridine (70.9 mg, 0.373 mmol, 1.4 eq.) and $Pd(OAc)_2$ (8.96 mg, 0.04 mmol, 0.15 eq.) in toluene (3.03 mL) and H_2O (0.16 mL) gave the crude product as a yellow oil. Purification by flash column chromatography on silica with 95:5-9:1 CH_2Cl_2-EtOAc as eluent gave aryl pyrrolidine **17** (54 mg, 69%) as a yellow oil, R_F (8:2 hexane-EtOAc) 0.18; IR (ATR) 2975, 1687 (C=O), 1389, 1159 cm^{-1}; ^{1}H NMR (400 MHz, DMSO-d_6) δ 8.17 (d, *J* = 3.0 Hz, 1H, Ar), 7.14 (dd, *J* = 9.0, 3.0 Hz, 1H, Ar), 3.88 (ddd, *J* = 12.9, 3.5, 3.5 Hz, 1H, NCH), 3.32 (ddd, *J* = 12.0, 9.0, 9.0 Hz, 1H, NCH), 2.42–2.27 (m, 4H, CMe, NCH_2C*H*), 2.08 (ddd, *J* = 12.5, 9.0, 3.5 Hz, 1H, NCH_2C*H*), 1.91 (dd, *J* = 9.0, 5.5, Hz, 1H, NCCH), 1.68–1.58 (m, 1H, NCCH), 1.08 (s, 9H, CMe_3), 1.06 (dd, *J* = 5.5, 5.5 Hz, 1H, NCCH); ^{13}C NMR (100.6 MHz, $CDCl_3$) δ 159.8 (C=O), 157.3 (Ar), 154.5 (d, *J* = 242.4 Hz, CF), 134.7 (d, *J* = 3.0 Hz, *C*CH_3), 133.5 (d, *J* = 22.2 Hz, N=CH), 124.6 (d, *J* = 18.2 Hz, C=*C*H), 79.5 (*C*Me_3), 50.2 (NC), 47.1 (NCH_2), 28.1 (C*Me*$_3$), 27.1 (NC*C*H), 24.8 (NCH_2*C*H_2), 18.9 (C*C*H_3), 17.7 (NC*C*H_2); MS (ESI) *m/z* 315 (M + Na)$^+$]; HRMS *m/z* calcd for $C_{16}H_{21}FN_2O_2$ (M + Na)$^+$ 315.1479, found 315.1483 (–1.1 ppm error).

Lab book reference RA 1-010

2-Bromo-6-(methoxymethyl)pyridine S29

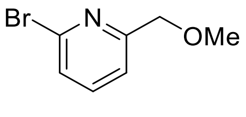

S29

A solution of 2-bromo-6-(hydroxymethyl)pyridine (500 mg, 2.66 mmol, 1.0 eq.) in THF (5 mL) was added to dropwise to a stirred solution of NaH (138 mg of a 60% dispersion in mineral oil, 3.12 mmol, 1.2 eq.) in THF (10 mL) at 0 °C under Ar. After gas evolution ceased, methyl iodide (0.25 mL, 3.99 mmol, 1.5 eq.) was added dropwise. After being allowed to warm to rt, the resulting mixture was stirred at rt for 1 h. Saturated $NH_4Cl_{(aq)}$ (5 mL) and 35% $NH_{3(aq)}$ (5 mL) were added sequentially and the mixture was extracted with EtOAc (3 × 7 mL). The combined organic extracts were dried (Na_2SO_4) and evaporated under reduced pressure to give methyl ether **S29** (439 mg, 81%) as a yellow oil, R_F (7:3 hexane-EtOAc) 0.76; IR (ATR) 2924, 1557, 1406, 1121 cm^{-1}; ^{1}H NMR (400 MHz, $CDCl_3$) δ 7.54 (dd, *J* = 8.0, 8.0 Hz, 1H, Ar), 7.38 (dd, *J* = 8.0 Hz, 1H, Ar), 7.36 (dd, *J* = 8.0 Hz, 1H, Ar), 4.53 (s, 2H, OCH_2), 3.45 (s, 3H, OMe); ^{13}C NMR (100.6 MHz, $CDCl_3$) δ 160.3 (*ipso*-Ar), 141.4 (*ipso*-Ar), 139.1 (Ar), 126.7 (Ar), 119.9 (Ar), 74.7 (OCH_2), 59.0 (OMe); MS (ESI) *m/z* 223 [(M(^{79}Br) + Na)$^+$]; HRMS *m/z* calcd for $C_7H_8{}^{79}BrNO$ (M + Na)$^+$ 223.9681, found 223.9679 (+1.0 ppm error). Spectroscopic data consistent with those reported in the literature.[9]

Lab book reference RA 1-015

***tert*-Butyl 1-(6-(methoxymethyl)pyridin-2-yl)-2-azabicyclo[3.1.0]hexane-2-carboxylate 18**

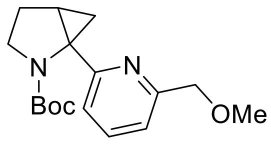

18

Using general procedure A, *N*-Boc MIDA boronate **1a** (90 mg, 0.27 mmol, 1.0 eq.), Cs_2CO_3 (520 mg, 1.6 mmol, 6.0 eq.), PCy_3 (22.4 mg, 0.0798 mmol, 0.3 eq.), 6-bromo-2-(methoxymethyl)pyridine **S29** (75.2 mg, 0.373 mmol, 1.4 eq.) and $Pd(OAc)_2$ (8.96 mg, 0.04 mmol, 0.15 eq.) in toluene (3.03 mL) and H_2O (0.16 mL) gave the crude product as a yellow oil. Purification by flash column chromatography on silica with 9:1 hexane-EtOAc as eluent gave aryl pyrrolidine **18** (59 mg, 73%) as a clear oil, R_F (8:2 hexane-EtOAc) 0.23; IR (ATR) 2975, 1695 (C=O), 1363, 1160 cm^{-1}; ^{1}H NMR (400 MHz, $CDCl_3$) δ 7.56

(dd, J = 8.0, 7.5 Hz, 1H, Ar), 7.28 (d, J = 8.0 Hz, 1H, Ar), 7.13 (d, J = 7.5 Hz, 1H, Ar), 4.45 (d, J = 12.0 Hz, 1H, OCH), 4.44 (d, J = 12.0 Hz, 1H, OCH), 3.80 (ddd, J = 11.5, 9.5, 6.5 Hz, 1H, NCH), 3.72–3.61 (m, 1H, NCH), 3.40 (s, 3H, OMe), 2.31–2.24 (m, 1H, NCCH), 2.24–2.17 (m, 1H, NCH_2CH), 1.91 (dddd, J = 12.5, 8.0, 6.5, 1.5 Hz, 1H, NCH_2CH), 1.86–1.78 (m, 1H, NCCH), 1.23 (s, 9H, CMe_3), 1.02 (dd, J = 5.5, 5.5 Hz, 1H, NCCH); ^{13}C NMR (100.6 MHz, $CDCl_3$) δ 157.3 (C=O), 156.4 (*ipso*-Ar), 137.5 (*ipso*-Ar), 136.1 (Ar), 118.9 (Ar), 117.8 (Ar), 79.7 (CMe_3), 75.8 (OCH_2), 58.7 (OMe), 51.7 (NC), 51.0 (NCH_2), 32.1 (NC*C*H), 28.3 (CMe_3), 27.0 (NCH_2CH_2), 25.2 ($NCCH_2$); MS (ESI) *m/z* 327 $(M + Na)^+$]; HRMS *m/z* calcd for $C_{17}H_{24}N_2O_3$ $(M + Na)^+$ 327.1679, found 327.1675 (+1.2 ppm error).

Lab book reference RA 1-017

***tert*-Butyl 1-(2-methoxypyridin-3-yl)-2-azabicyclo[3.1.0]hexane-2-carboxylate 19**

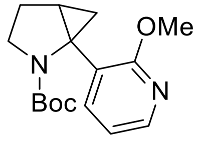

19

Using general procedure A, *N*-Boc MIDA boronate **1a** (90 mg, 0.27 mmol, 1.0 eq.), Cs_2CO_3 (520 mg, 1.6 mmol, 6.0 eq.), PCy_3 (22.4 mg, 0.0798 mmol, 0.3 eq.), 3-bromo-2-methoxypyridine (44.5 µL, 0.373 mmol, 1.4 eq.) and $Pd(OAc)_2$ (8.96 mg, 0.04 mmol, 0.15 eq.) in toluene (3.03 mL) and H_2O (0.16 mL) gave the crude product as a yellow oil. Purification by flash column chromatography on silica with CH_2Cl_2 and then 95:5 CH_2Cl_2-EtOAc as eluent gave aryl pyrrolidine **19** (56 mg, 73%) as a yellow oil, R_F (8:2 hexane-EtOAc) 0.29; IR (ATR) 2975, 1688 (C=O), 1462, 1391 cm^{-1}; ^1H NMR (400 MHz, $CDCl_3$) δ 8.02 (dd, J = 5.0, 2.0 Hz, 1H, Ar), 7.59–7.43 (m, 1H, Ar), 6.79 (dd, J = 7.0, 5.0 Hz, 1H, Ar), 4.00–3.86 (m, 4H, OMe, NCH), 3.61–3.47 (m, 1H, NCH), 2.45–2.26 (m, 1H, NCH_2CH), 1.90 (dddd, J = 13.0, 8.5, 7.0, 1.5 Hz, 1H, NCH_2CH), 1.63–1.45 (m, 2H, NCCH), 1.20 (s, 9H, CMe_3), 0.92 (dd, J = 5.0, 5.0 Hz, 1H, $NCCH_2$); ^{13}C NMR (100.6 MHz, $CDCl_3$) δ 162.8 (C=O), 155.5 (*ipso*-Ar), 145.2 (Ar), 139.4 (Ar), 123.1 (Ar), 116.2 (Ar), 79.3 (CMe_3), 53.6 (OMe), 50.5 (NCH_2), 46.3 (NC), 28.4 (CMe_3), 27.3 (NCH_2CH_2), 26.9 (NC*C*H), 22.8 ($NCCH_2$); MS (ESI) *m/z* 313 $(M + Na)^+$]; HRMS *m/z* calcd for $C_{16}H_{22}N_2O_3$ $(M + Na)^+$ 313.1523, found 313.1519 (+1.1 ppm error).

Lab book reference RA 1-009

5-Bromo-1-(4-methylbenzenesulfonyl)-1H-pyrrolo[2,3-b]pyridine S30

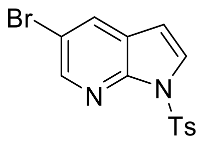

S30

NaH (60% dispersion in mineral oil, 72 mg, 1.3 mmol, 1.3 eq.) was added to a stirred solution of 5-bromo-7-azaindole (197 mg, 1.0 mmol, 1.0 eq.) in THF (7 mL) at 0 °C under Ar. The resulting mixture was allowed to warm to rt and stirred at rt for 30 min. Then, the mixture was cooled to 0 °C and TsCl (229 mg, 1.2 mmol, 1.2 eq.) was added. The mixture was allowed to warm to rt and stirred at rt for 16 h. Then, H_2O (10 mL) was added dropwise and the mixture was extracted with EtOAc (3 × 5 mL). The combined organics were washed with brine (5 mL), dried (Na_2SO_4) and evaporated under reduced pressure to give the crude product as a white solid. Purification by flash column chromatography on silica with 9:1 hexane-EtOAc as eluent gave *N*-Ts azaindole **S30** (188 mg, 54%) as a white solid, mp 139–141 °C (lit.,[10] 140–141 °C); R_F (8:2 hexane-EtOAc) 0.44; IR (ATR) 1441, 1374, 1153, 581 cm^{-1}; ^{1}H NMR (400 MHz, $CDCl_3$) δ 8.43 (d, *J* = 2.0 Hz, 1H, Ar), 8.03 (d, *J* = 8.5 Hz, 2H, Ar), 7.95 (d, *J* = 2.0 Hz, 1H, Ar), 7.72 (d, *J* = 4.0 Hz, 1H, Ar), 7.26 (d, *J* = 8.5 Hz, 2H, Ar), 6.52 (d, *J* = 4.0 Hz, 1H, Ar), 2.36 (s, 3H, CH_3); ^{13}C NMR (100.6 MHz, $CDCl_3$) δ 145.61 (*ipso*-Ar), 145.56 (Ar), 145.53 (*ipso*-Ar), 135.1 (Ar), 131.8 (Ar), 129.8 (Ar), 128.2 (Ar) 128.0 (Ar), 124.5 (*ipso*-Ar), 115.3 (*ipso*-Ar), 104.6 (Ar), 21.8 (CH_3); MS (ESI) *m/z* 372 [(M(^{79}Br) + Na)$^+$]; HRMS *m/z* calcd for $C_{14}H_{11}{}^{79}BrN_2O_2S$ $(M + Na)^+$ 372.9617, found 372.9611 (+1.5 ppm error). Spectroscopic data consistent with those reported in the literature.[10]

Lab book reference RA 1-020

***tert*-Butyl 1-[1-(4-methylbenzenesulfonyl)-1H-pyrrolo[2,3-b]pyridin-5-yl]-2-azabicyclo[3.1.0]hexane-2-carboxylate 20**

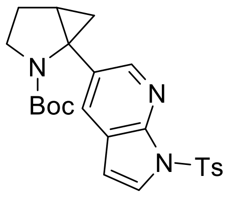

20

Using general procedure A, *N*-Boc MIDA boronate **1a** (90 mg, 0.27 mmol, 1.0 eq.), Cs_2CO_3 (520 mg, 1.6 mmol, 6.0 eq.), PCy_3 (22.4 mg, 0.0798 mmol, 0.3 eq.), tosyl protected azaindole **S30** (131 mg, 0.373 mmol, 1.4 eq.) and $Pd(OAc)_2$ (8.96 mg, 0.04 mmol, 0.15 eq.) in toluene (3.03 mL) and H_2O (0.16 mL) gave the crude product as a yellow oil. Purification by flash column chromatography on silica with 9:1 to 8:2 hexane-EtOAc as eluent gave aryl pyrrolidine **20** (95 mg, 78%) as a clear oil, R_F (8:2 hexane-EtOAc) 0.18; IR (ATR) 2931, 1688 (C=O), 1365, 1154 cm^{-1}; ^1H NMR (400 MHz, $CDCl_3$) δ 8.31 (d, J = 2.0 Hz, 1H, Ar), 7.98 (d, J = 8.5 Hz, 2H, Ar), 7.72 (d, J = 2.0 Hz, 1H, Ar), 7.64 (d, J = 4.0 Hz, 1H, Ar), 7.19 (d, J = 8.5 Hz, 2H, Ar), 6.49 (d, J = 4.0 Hz, 1H, Ar), 3.84 (ddd, J = 11.5, 9.5, 5.5 Hz, 1H, NCH), 3.50 (ddd, J = 11.5, 9.0, 6.0 Hz, 1H, NCH), 2.36–2.24 (m, 4H, NCH_2CH, CMe), 1.95 (dddd, J = 13.0, 9.0, 5.5, 1.5 Hz, 1H, NCH_2CH_2), 1.71 (dd, J = 9.0, 5.5 Hz, 1H, NCCH), 1.57–1.50 (m, 1H, NCCH), 1.06 (s, 9H, CMe_3), 1.02 (dd, J = 5.5, 5.5 Hz, 1H, NCCH); ^{13}C NMR (100.6 MHz, $CDCl_3$) δ 156.0 (C=O), 146.1 (*ipso*-Ar), 145.1 (*ipso*-Ar), 144.5 (*ipso*-Ar), 135.49 (*ipso*-Ar), 132.5 (Ar), 129.7 (Ar), 128.01 (Ar), 127.99 (Ar), 126.9 (Ar), 122.4 (*ipso*-Ar), 105.4 (Ar), 79.8 ($OCMe_3$), 49.8 (NCH_2), 48.0 (NC), 29.5 (NC*C*H), 28.2 (CMe_3), 26.5 (NCH_2CH_2), 21.7 (CH_3), 21.4 ($NCCH_2$); MS (ESI) *m/z* 476 $(M + Na)^+$]; HRMS *m/z* calcd for $C_{24}H_{27}N_3O_4S$ $(M + Na)^+$ 476.1614, found 476.1616 (–0.2 ppm error).

Lab book reference RA 1-022

6-Bromo-5-methyl-1-(4-methylbenzenesulfonyl)-1H-indazole S31

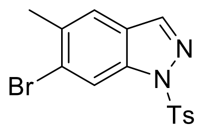

S31

To a solution of 6-bromo-5-methyl-1*H*-indazole (106 mg, 0.5 mmol, 1.0 eq.) in THF (5 mL) was added KOtBu (62 mg, 0.55 mmol, 1.1 eq.) at 0 °C under Ar and the resulting mixture was stirred for 30 min. TsCl (105 mg, 0.55 mmol, 1.1 eq.) was added and left to stir at rt for 40 min. The reaction was quenched with $NH_4Cl_{(aq)}$ (5 mL) and extracted with EtOAc (3 × 5 mL). The combined organics were washed with brine (5 mL), dried (Na_2SO_4) and evaporated under reduced pressure to give the crude product. Purification by flash chromatography on silica with hexane-EtOAc 9:1 as eluent gave tosyl protected indazole **S31** (61 mg, 34%) as white solid; mp 176–178 °C; R_F (hexane/EtOAc 7:3) 0.62; IR (ATR) 3141, 1631, 1379, 1174 cm^{-1}; ^1H NMR (400 MHz, $CDCl_3$) δ 8.49 (s, 1H, Ar), 7.95 (d, J = 8.0 Hz, 2H, Ar), 7.90 (s, 1H, Ar), 7.44 (s, 1H, Ar), 7.30 (d, J = 8.0 Hz, 2H, Ar), 2.39 (s, 3H, Me), 2.38 (s, 3H, Me); ^{13}C NMR (100.6 MHz, $CDCl_3$) 151.4 (*ipso*-Ar), 146.7 (*ipso*-Ar), 133.9 (Ar), 133.5 (Ar), 130.3 (Ph), 128.7 (Ph), 127.9 (*ipso*-Ar), 123.9 (Ar), 121.9 (Ar), 121.1 (Ar), 120.4 (Ar), 23.7 (Me), 21.9 (Me); MS (ESI) *m/z* 386 $(M + Na)^+$]; HRMS *m/z* calcd for $C_{15}H_{13}BrN_2O_2S$ $(M + Na)^+$ 386.9773, found 386.9771 (+0.6 ppm error).

Lab book reference RA 1-045

***tert*-Butyl 1-[5-methyl-1-(4-methylbenzenesulfonyl)-1H-indazol-6-yl]-2-azabicyclo[3.1.0]hexane-2-carboxylate 21**

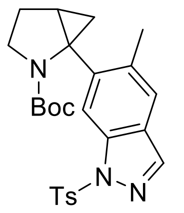

21

Using general procedure A, *N*-Boc MIDA boronate **1a** (37 mg, 0.11 mmol, 1.0 eq.), Cs_2CO_3 (215 mg, 0.66 mmol, 6 eq.), PCy_3 (9.25 mg, 0.033 mmol, 0.3 eq.), tosyl protected indazole **S31** (55 mg, 0.15 mmol, 1.4 eq.) and $Pd(OAc)_2$ (3.69 mg, 0.017 mmol, 0.15 eq.) in toluene (3.03 mL) and H_2O (0.16 mL) gave

the crude product as a yellow oil. Purification by flash column chromatography on silica with 9:1 hexane-Et_2O as eluent gave aryl pyrrolidine **21** (37 mg, 73%) as a yellow oil, R_F (9:1 hexane-Et_2O) 0.26; IR (ATR) 2975, 2244, 1681 (C=O), 1174 cm^{-1}; ^1H NMR (400 MHz, $CDCl_3$) δ 8.38 (s, 1H, Ar), 7.93–7.83 (m, 2H, Ar), 7.49 (s, 1H, Ar), 7.35–7.18 (m, 3H, Ar), 3.81 (ddd, *J* = 11.5, 10.5, 4.0 Hz, 1H, NCH), 3.30 (ddd, *J* = 11.5, 9.0, 8.5 Hz, 1H, NCH), 2.38–2.22 (m, 7H, Me, CH), 2.02 (ddd, *J* = 13.0, 9.0, 4.0 Hz, 1H, CH), 1.58–1.45 (m, 2H, CH), 1.09–0.82 (m, 10H, CH, CMe_3); ^{13}C NMR (100.6 MHz, $CDCl_3$) δ 155.9 (C=O), 151.5 (*ipso*-Ar), 146.2 (*ipso*-Ar), 141.8 (*ipso*-Ar), 135.0 (*ipso*-Ar), 134.0 (*ipso*-Ar), 130.1 (Ph), 128.4 (Ph), 123.3 (*ipso*-Ar), 121.3 (Ar), 112.0 (Ar), 119.5 (Ar), 79.6 (CMe_3), 49.92 (NC), 47.8 (NCH_2), 28.1 (CMe_3), 27.2 (NCCH), 25.7 (NCH_2CH_2), 21.8 (Me), 20.5 (Me), 18.9 ($NCCH_2$); MS (ESI) *m/z* 467 $(M + Na)^+$]; HRMS *m/z* calcd for $C_{25}H_{29}N_3O_4S$ $(M + Na)^+$ 490.1771, found 490.1768 (+0.6 ppm error).

Lab book reference RA 1-054

tert-Butyl (1_S_,5_R_)-1-[2-(methoxycarbonyl)phenyl]-2-azabicyclo[3.1.0]hexane-2-carboxylate (_S_,_R_)-S32

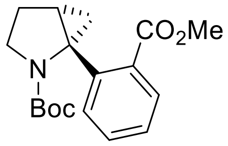

(*S*,*R*)-**S32**

Using general procedure A, *N*-Boc BIDA boronate **1a’** (90 mg, 0.18 mmol, 1.0 eq.), Cs_2CO_3 (352 mg, 1.08 mmol, 6.0 eq.), PCy_3 (15 mg, 0.054 mmol, 0.3 eq.), methyl 2-bromobenzoate (35.4 μL, 0.252 mmol, 1.4 eq.) and $Pd(OAc)_2$ (6 mg, 0.027 mmol, 0.15 eq.) in toluene (3.03 mL) and H_2O (0.16 mL) gave the crude product as a yellow oil. Purification by flash column chromatography on silica with 95:5-90:10 hexane-EtOAc as eluent gave aryl pyrrolidine (*S,R*)-**S32** (24 mg, 42%) as a yellow oil; R_F (9:1 hexane-EtOAc) 0.12; IR (ATR) 2975, 1729, 1687 (C=O), 1364 cm^{-1}; ^1H NMR (400 MHz, $CDCl_3$) δ 7.54 (dd, *J* = 7.5, 1.0 Hz, 1H, Ar), 7.35 (dd, *J* = 7.5, 7.5 Hz, 1H, Ar), 7.31–7.21 (m, 2H, Ar), 3.85 (s, 1H, OMe), 3.73 (ddd, *J* = 11.0, 11.0, 4.0 Hz, 1H, NCH), 3.39–3.24 (m, 1H, NCH), 2.58–2.43 (m, 1H, NCH_2CH), 1.98 (ddd, *J* = 13.0, 9.5, 4.0 Hz, 1H, NCH_2CH), 1.80–1.63 (m, 2H, NCCH), 1.05 (s, 9H, CMe_3), 0.87 (dd, *J* = 5.5, 5.5 Hz, 1H, NCCH); ^{13}C NMR (100.6 MHz, $CDCl_3$) δ 169.4 (C=O), 155.9 (C=O), 140.1 (*ipso*-Ar), 132.1 (*ipso*-Ar), 130.5 (Ar), 129.0 (Ar), 128.8 (Ar), 126.6 (Ar), 79.4 (CMe_3), 52.1 (OMe), 48.84 (NCH_2), 48.76 (NC), 30.0 (NC*C*H), 28.0 (CMe_3), 25.6 (NCH_2CH), 18.6 ($NCCH_2$); HRMS *m/z* calcd for

$C_{18}H_{23}NO_4$ (M + Na)$^+$ 340.1519, found 340.1513 (+1.9 ppm error); [α]D –46.8 (*c* 1.00 in $CHCl_3$), [lit.,[7] [α]D –46.4 (c 1.00 in $CHCl_3$), for (*S,R*)-**S32**, 99:1 er].

Lab book reference RA 1-093

tert-Butyl (1*R*,5*S*)-1-[2-(methoxycarbonyl)phenyl]-2-azabicyclo[3.1.0]hexane-2-carboxylate (*R*,*S*)-S32

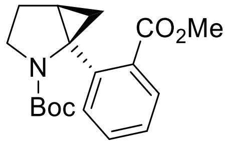

(*R*,*S*)-**S32**

Using general procedure A, *N*-Boc BIDA boronate **1a''** (90 mg, 0.18 mmol, 1.0 eq.), Cs_2CO_3 (352 mg, 1.08 mmol, 6.0 eq.), PCy_3 (15 mg, 0.054 mmol, 0.3 eq.), methyl 2-bromobenzoate (35.4 µL, 0.252 mmol, 1.4 eq.) and $Pd(OAc)_2$ (6 mg, 0.027 mmol, 0.15 eq.) in toluene (3.03 mL) and H_2O (0.16 mL) gave the crude product as a yellow oil. Purification by flash column chromatography on silica with 95:5-90:10 hexane-EtOAc as eluent gave aryl pyrrolidine (*R,S*)-**S32** (20 mg, 35%) as a yellow oil; [α]D +45.0 (*c* 1.00 in $CHCl_3$), [lit.,[7] [α]D –46.4 (c 1.00 in $CHCl_3$), for (*S,R*)-**S32**, 99:1 er].

Lab book reference RA 1-088

***tert*-Butyl 1-(pyrimidin-5-yl)-6-azaspiro[2.5]octane-6-carboxylate 22**

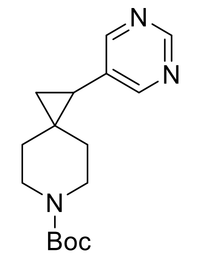

22

Using general procedure B, MIDA boronate **1b** (92 mg, 0.25 mmol, 1.0 eq.), Cs_2CO_3 (244 mg, 0.75 mmol, 3.0 eq.), 5-bromopyrimidine (56 mg, 0.35 mmol, 1.4 eq.), $Pd(OAc)_2$ (2.8 mg, 0.01 mmol, 0.05 eq.) and PCy_3 (7.0 mg, 0.02 mmol, 0.10 eq.) in toluene (5 mL) and H_2O (0.5 mL) gave the crude product. Purification by flash column chromatography on silica with 7:3 hexane-EtOAc as eluent gave arylated piperidine **22** (51 mg, 70%) as a white solid, mp 92–94 °C; R_F (7:3 hexane-EtOAc) 0.18; IR (ATR) 2931, 1687 (C=O), 1415, 1238, 1167, 1124, 1008, 730 cm^{-1}; ^{1}H NMR (400 MHz, $CDCl_3$) δ 9.03 (s, 1H, Ar), 8.56 (s, 2H, Ar), 3.62 (ddd, *J* = 13.0, 6.5, 4.0 Hz, 1H, NCH), 3.43–3.28 (m, 2H, NCH), 3.16 (ddd, *J* = 13.0, 8.0, 4.0 Hz, 1H, NCH), 1.90 (dd, *J* = 8.0, 6.5 Hz, 1H, C*H*Ar), 1.65 (ddd, *J* = 12.5, 8.3, 3.9 Hz, 1H, CH), 1.47–1.37 (m, 10H, CMe_3, CH), 1.25–1.16 (m, 1H, CH), 1.07 (ddd, *J* = 13.5, 6.5, 4.0 Hz, 1H, CH), 1.02–0.97 (m, 2H, CH); ^{13}C NMR (100.6 MHz, $CDCl_3$) δ 157.1 (Ar), 156.7 (Ar), 155.0 (C=O), 132.6 (*ipso*-Ar), 79.7 (O*C*Me_3), 42.9 (br, NCH_2), 36.5 (CH_2), 30.1 (CH_2), 28.5 (C*Me_3*), 25.6 (CHAr), 23.6 (C), 15.9 (CH*CH_2*); HRMS (ESI) *m/z* calcd for $C_{16}H_{23}N_3O_2$ $(M + Na)^+$ 312.1682, found 312.1683 (–0.3 ppm error).

Lab book reference: ARG-2-132

Using general procedure A, *N*-Boc MIDA boronate **1a** (93 mg, 0.25 mmol, 1.0 eq.), Cs_2CO_3 (497 mg, 1.52 mmol, 6 eq.), PCy_3 (9.25 mg, 0.033 mmol, 0.3 eq.), 5-bromopyrimidine (57 mg, 0.36 mmol, 1.4 eq.) and $Pd(OAc)_2$ (8.6 mg, 0.038 mmol, 0.15 eq.) in toluene (3.5 mL) and H_2O (0.3 mL) gave the crude product. Purification by flash column chromatography on silica with EtOAc as eluent gave aryl pyrrolidine **22** (54 mg, 73%) as a cream solid.

Lab book reference: HFK8-036

Using general procedure B, MIDA boronate **1b** (92 mg, 0.25 mmol, 1.0 eq.), Cs_2CO_3 (244 mg, 0.75 mmol, 3.0 eq.), 5-bromopyrimidine (56 mg, 0.35 mmol, 1.4 eq.), $Pd(OAc)_2$ (2.8 mg, 0.01 mmol, 0.05 eq.) and PCy_3 (7.0 mg, 0.02 mmol, 0.10 eq.) in toluene (5 mL) and H_2O (0.5 mL) gave the crude product. Purification by flash column chromatography on silica with 7:3 hexane-EtOAc as eluent gave arylated piperidine **22** (51 mg, 70%) as a white solid.

Using modified general procedure A, MIDA boronate **XX** (92 mg, 0.25 mmol, 1.0 eq), Cs_2CO_3 (488 mg, 1.50 mmol, 6.0 eq), 5-bromopyrimidine (56 mg, 0.35 mmol, 1.4 eq), $Pd(OAc)_2$ (2.8 mg, 0.01 mmol, 0.05 eq) and PCy_3 (7.0 mg, 0.02 mmol, 0.10 eq) in toluene (5 mL) and H_2O (0.5 mL) gave the crude product. Purification by flash column chromatography on silica with 7:3 hexane-EtOAc as eluent gave arylated piperidine **XX** (42 mg, 59%) as a white solid. Spectroscopic data identical to those reported above.

Lab book reference: ARG-2-128

A mixture of potassium trifluoroborate **S2** (159 mg, 0.5 mmol, 1.0 eq.), 5-bromopyrimidine (95 mg, 0.6 mmol, 1.2 eq.), $Pd(dppf)Cl_2$ (22 mg, 0.03 mmol, 0.06 eq.) and Cs_2CO_3 (375 mg, 1.15 mmol, 2.3 eq.) in THF (2.1 mL) and water (0.7 mL) was stirred at rt in a sealed tube and the suspension was degassed by sparging with Ar for 20 min. The resulting mixture was stirred and heated at 100 °C for 18 h. The mixture was allowed to cool to rt, water (5 mL) and CH_2Cl_2 (5 mL) were added and the two layers were separated. The aqueous layer was extracted with CH_2Cl_2 (3 × 5 mL). The combined organic layers were dried (Na_2SO_4) and evaporated under reduced pressure to give the crude product. Purification by flash column chromatography on silica with EtOAc as eluent gave pyrimidine cyclopropane **22** (60 mg, 42%) as a white solid.

Lab Book Reference: JNL 47

***tert*-Butyl 1-(2-fluorophenyl)-6-azaspiro[2.5]octane-6-carboxylate 23**

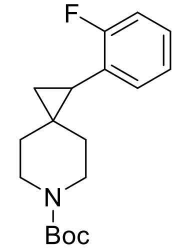

23

Using general procedure B, MIDA boronate **1b** (92 mg, 0.25 mmol, 1.0 eq.), Cs_2CO_3 (244 mg, 0.75 mmol, 3.0 eq.), 1-bromo-2-fluorobenzene (61 mg, 0.35 mmol, 1.4 eq.), $Pd(OAc)_2$ (2.8 mg, 0.01 mmol, 0.05 eq.) and PCy_3 (7.0 mg, 0.02 mmol, 0.10 eq.) in toluene (5 mL) and H_2O (0.5 mL) gave the crude product. Purification by flash column chromatography on silica with 96:4 hexane-EtOAc as eluent gave arylated piperidine **23** (50 mg, 66%) as a colourless oil which solidified upon standing to a white solid, mp 60–62 °C; R_F (96:4 hexane-EtOAc) 0.18; IR (ATR) 2925, 1687 (C=O), 1418, 1235, 1167, 1121, 751 cm^{-1}; ^{1}H NMR (400 MHz, $CDCl_3$) δ 7.20–7.12 (m, 1H, Ar), 7.07–6.95 (m, 3H, Ar), 3.56–3.49 (m, 2H, NCH), 3.27 (ddd, *J* = 13.0, 7.0, 4.0 Hz, 1H, NCH), 3.11 (ddd, *J* = 13.0, 7.5, 4.0 Hz, 1H, NCH), 1.99 (dd, *J* = 8.5, 5.5 Hz, 1H, C*H*Ar), 1.59–1.50 (m, 2H, CH), 1.43 (s, 9H, CMe_3), 1.22–1.06 (m, 2H, CH), 0.94 (dd, *J* = 5.5, 5.0 Hz, 1H, CH), 0.86 (dd, *J* = 8.5, 5.0 Hz, 1H, CH); ^{13}C NMR (100.6 MHz, $CDCl_3$) δ 162.8 (d, *J* = 245.5 Hz, *ipso*-Ar), 155.1 (C=O), 129.6 (d, *J* = 4.0 Hz, Ar), 127.6 (d, *J* = 8.0 Hz, Ar), 126.4 (d, *J* = 15.0 Hz, *ipso*-Ar), 123.7 (d, *J* = 3.5 Hz, Ar), 115.0 (d, *J* = 22.0 Hz, Ar), 79.3 (O*C*Me_3), 43.3 (br, NCH_2), 36.5 (CH_2), 30.0 (CH_2), 28.6 (C*Me*$_3$), 24.6 (C), 22.7 (d, *J* = 4.5 Hz, CHAr), 15.3 (CCH_2); HRMS (ESI) *m/z* calcd for $C_{18}H_{24}FNO_2$ $(M + Na)^+$ 328.1683, found 328.1682 (+0.5 ppm error).

Lab book reference: ARG-2-139

***tert*-Butyl 1-(6-(trifluoromethyl)pyridin-3-yl)-6-azaspiro[2.5]octane-6-carboxylate 24**

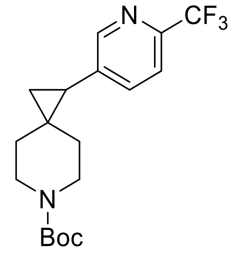

24

Using general procedure B, MIDA boronate **1b** (92 mg, 0.25 mmol, 1.0 eq.), Cs_2CO_3 (244 mg, 0.75 mmol, 3.0 eq.), 5-bromo-2-trifluoromethylpyridine (79 mg, 0.35 mmol, 1.4 eq.), $Pd(OAc)_2$ (2.8 mg, 0.01 mmol, 0.05 eq.) and PCy_3 (7.0 mg, 0.02 mmol, 0.10 eq.) in toluene (5 mL) and H_2O (0.5 mL) gave the crude product. Purification by flash column chromatography on silica with 4:1 hexane-EtOAc as eluent gave arylated piperidine **24** (63 mg, 70%) as a colourless oil which solidified upon standing to a white solid, mp 82–84 °C; R_F (4:1 hexane-EtOAc) 0.25; IR (ATR) 2928, 1684 (C=O), 1336, 1239, 1123, 1088, 732 cm^{-1}; ^{1}H NMR (400 MHz, $CDCl_3$) δ 8.54 (s, 1H, Ar), 7.63–7.42 (m, 2H, Ar), 3.72–3.52 (m, 1H, NCH), 3.43–3.34 (m, 1H, NCH), 3.27–3.19 (m, 1H, NCH), 3.19–3.11 (m, 1H, NCH), 1.99 (dd, *J* = 7.0, 7.0 Hz, 1H, C*H*Ar), 1.61 (ddd, *J* = 12.5, 8.0, 3.5 Hz, 1H, CH), 1.46–1.32 (m, 10H, CH, CMe_3), 1.23–1.13 (m, 1H, CH), 1.13–1.04 (m, 1H, CH), 1.04–0.96 (m, 2H, CH); ^{13}C NMR (100.6 MHz, $CDCl_3$) (rotamers and conformers) δ 154.9 (C=O), 150.8 (Ar), 150.6 (Ar), 145.7 (q, *J* = 35.0 Hz, *ipso*-Ar), 138.4 (*ipso*-Ar), 137.0 (br, Ar), 136.8 (Ar), 121.8 (q, *J* = 274.0 Hz, CF_3), 119.9 (q, *J* = 2.5 Hz, Ar), 119.6 (q, *J* = 2.5 Hz, Ar), 79.6 (O*C*Me_3), 43.3 (br, NCH_2), 36.5 (CH_2), 29.8 (CH_2), 28.7 (C*Me₃*→CMe_3), 28.5 (CMe_3), 28.3 (CMe_3), 28.1 (CMe_3), 26.2 (CHAr), 26.0 (C), 25.8 (C), 16.5 (CH*C*H_2), 16.4 (C*C*H_2), 16.2 (C*C*H_2); HRMS (ESI) *m/z* calcd for $C_{18}H_{23}F_3N_2O_2$ $(M + Na)^+$ 379.1604, found 379.1600 (–1.0 ppm error).

Lab book reference: ARG-2-134

***tert*-Butyl 1-[1-(4-methylbenzenesulfonyl)-1H-pyrrolo[2,3-b]pyridin-5-yl]-6-azaspiro[2.5]octane-6-carboxylate 25**

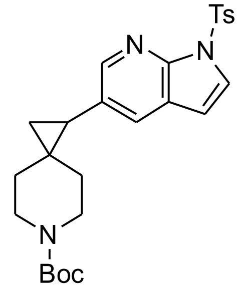

25

Using general procedure B, MIDA boronate **1b** (183 mg, 0.50 mmol, 1.0 eq.), Cs_2CO_3 (488 mg, 1.5 mmol, 3.0 eq.), 5-bromo-*N*-tosyl-azaindole **S30** (245 mg, 0.70 mmol, 1.4 eq.), $Pd(OAc)_2$ (5.6 mg, 0.025 mmol, 0.05 eq.) and PCy_3 (7.0 mg, 0.05 mmol, 0.10 eq.) in toluene (10 mL) and H_2O (1.0 mL) gave the crude product. Purification by flash column chromatography on silica with 9:1 hexane-EtOAc to 7:3 hexane-EtOAc as eluent gave arylated piperidine **25** (176 mg, 73%) as a white solid, mp 74-76 °C; R_F (4:1 hexane-EtOAc) 0.26; IR (ATR) 2924, 1597 (C=O), 1498, 1443, 993, 768 cm^{-1}; ^{1}H NMR (400 MHz, $CDCl_3$) δ 8.31 (d, *J* = 2.0 Hz, 1H, Ar), 8.07–8.02 (m, 2H, Ar), 7.66 (d, *J* = 4.0 Hz, 1H, Ar), 7.57 (d, *J* = 2.0 Hz, 1H, Ar), 7.26–7.22 (m, 2H, Ar), 6.50 (d, *J* = 4.0 Hz, 1H, Ar), 3.58 (ddd, *J* = 13.0, 6.5, 3.5 Hz, 1H, NCH), 3.41 (ddd, *J* = 13.0, 8.0, 4.0 Hz, 1H, NCH), 3.28–3.18 (m, 1H, NCH), 3.13 (ddd, *J* = 12.0, 8.0, 4.0 Hz, 1H, NCH), 2.35 (s, 3H, Me), 2.00 (dd, *J* = 8.5, 6.0 Hz, 1H, C*H*Ar), 1.58 (ddd, *J* = 12.0, 7.5, 3.5 Hz, 1H, CH), 1.48–1.35 (m, 10H, CMe_3, CH), 1.14 (ddd, *J* = 13.0, 8.0, 4.5 Hz, 1H, CH), 1.02 (ddd, *J* = 13.0, 6.0, 4.0 Hz, 1H, CH), 0.95–0.87 (m, 2H, CH); ^{13}C NMR (100.6 MHz, $CDCl_3$) δ 155.0 (C=O), 146.4 (Ar) , 145.9 (*ipso*-Ar), 145.2 (*ipso*-Ar), 135.5 (*ipso*-Ar), 129.7 (Ar), 129.2 (Ar), 128.1 (Ar), 126.8 (Ar), 122.5 (*ipso*-Ar), 105.0 (Ar), 79.5 (O*C*Me_3), 42.6 (br, NCH_2), 36.6 (CH_2), 30.0 (CH_2), 28.5 (CMe_3), 26.0 (CHAr), 24.9 (C), 21.7 (Me), 16.0 (CH*C*H_2) (1 × *ipso*-Ar resonance not resolved); HRMS (ESI) *m/z* calcd for $C_{26}H_{32}N_3O_4S$ (M + Na)$^+$ 507.1927, found 504.1929 (–0.4 ppm error).

Lab book reference: ARG-2-032

***tert*-Butyl 1-(quinolin-5-yl)-6-azaspiro[2.5]octane-6-carboxylate 26**

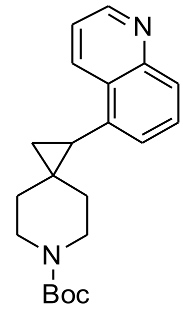

26

Using general procedure A, MIDA boronate **1b** (92 mg, 0.25 mmol, 1.0 eq.), Cs_2CO_3 (244 mg, 0.75 mmol, 3.0 eq.), 5-bromoquinoline (72 mg, 0.35 mmol, 1.4 eq.), $Pd(OAc)_2$ (2.8 mg, 0.01 mmol, 0.05 eq.) and PCy_3 (7.0 mg, 0.02 mmol, 0.10 eq.) in toluene (5 mL) and H_2O (0.5 mL) gave the crude product. Purification by flash column chromatography on silica with 4:1 hexane-EtOAc as eluent gave arylated piperidine **26** (48 mg, 58%) as a colourless oil which solidified upon standing to a white solid, mp 100–102 °C; R_F (96:4 hexane-EtOAc) 0.18; IR (ATR) 2924, 1681 (C=O), 1420, 1355, 1238, 1166, 1119, 728 cm^{-1}; ^1H NMR (400 MHz, $CDCl_3$) δ 8.83 (dd, J = 4.0, 1.5 Hz, 1H, Ar), 8.06 (dd, J = 8.5, 1.5 Hz, 1H, Ar), 7.99 (d, J = 9.0 Hz, 1H, Ar), 7.60 (dd, J = 9.0, 2.0 Hz, 1H, Ar), 7.49 (d, J = 2.0 Hz, 1H, Ar), 7.35 (dd, J = 8.5, 4.0 Hz, 1H, Ar), 3.60–3.44 (m, 2H, NCH), 3.26–3.11 (m, 2H, NCH), 2.14 (dd, J = 8.5, 6.0 Hz, 1H, C*H*Ar), 1.62–1.54 (m, 2H, CH), 1.41 (s, 9H, CMe_3), 1.18–1.14 (m, 2H, CH), 1.09 (dd, J = 6.0, 5.5 Hz, 1H, CH), 0.94 (dd, J = 8.5, 5.5 Hz, 1H, CH); ^{13}C NMR (100.6 MHz, $CDCl_3$) δ 155.0 (C=O), 149.8 (Ar), 147.1 (*ipso*-Ar), 137.7 (*ipso*-Ar), 135.7 (Ar), 131.8 (Ar), 128.9 (Ar), 128.2 (*ipso*-Ar), 126.1 (Ar), 121.3 (Ar), 79.4 (O*C*Me_3), 43.5 (br, NCH_2), 36.8 (CH_2), 29.8 (CH_2), 28.8 (CHAr), 28.5 (C*Me*$_3$), 25.7 (C), 16.0 (CH*C*H_2); HRMS (ESI) *m/z* calcd for $C_{21}H_{26}N_2O_2$ $(M + H)^+$ 339.2067, found 339.2069 (–0.5 ppm error).

Lab book reference: ARG-2-135

***tert*-Butyl 1-(4-sulfamoylphenyl)-6-azaspiro[2.5]octane-6-carboxylate 27**

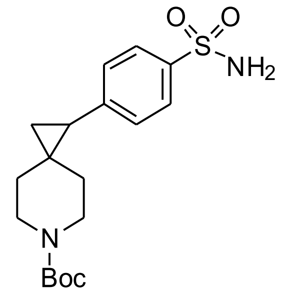

27

Using general procedure B, MIDA boronate **1b** (92 mg, 0.25 mmol, 1.0 eq.), Cs_2CO_3 (244 mg, 0.75 mmol, 3.0 eq.), 4-bromobenzenesulfonamide (82 mg, 0.35 mmol, 1.4 eq.), $Pd(OAc)_2$ (2.8 mg, 0.01 mmol, 0.05 eq.) and PCy_3 (7.0 mg, 0.02 mmol, 0.10 eq.) in toluene (5 mL) and H_2O (0.5 mL) gave the crude product. Purification by flash column chromatography on silica with 3:2 hexane-EtOAc as eluent gave arylated piperidine **27** (24 mg, 27%) as a pale-yellow oil, R_F (3:2 hexane-EtOAc) 0.13; IR (ATR) 3255 (NH_2) 2928, 1666 (C=O), 1427, 1331, 1241, 1158, 908, 728 cm^{-1}; ^1H NMR (400 MHz, $CDCl_3$) δ 7.95–7.76 (m, 2H, Ar), 7.37–7.08 (m, 2H, Ar), 5.16 (s, 2H, NH_2), 3.53 (ddd, J = 13.0, 7.0, 4.0 Hz, 1H, NCH), 3.46 (ddd, J = 13.0, 7.0, 4.0 Hz, 1H, NCH), 3.32–3.08 (m, NCH), 2.03 (dd, J = 8.0, 6.0 Hz, 1H, C*H*Ar), 1.60–1.47 (m, 2H, CH), 1.42 (s, 9H, CMe_3), 1.19–1.11 (m, 2H, CH), 1.02 (dd, J = 6.0, 5.5 Hz, 1H, CH), 0.94 (dd, J = 8.0, 5.5 Hz, 1H, CH); ^{13}C NMR (100.6 MHz, $CDCl_3$) δ 155.1 (C=O), 144.8 (*ipso*-Ar), 139.5 (*ipso*-Ar), 129.4 (Ar), 126.3 (Ar), 79.6 (O*C*Me_3), 43.5 (br, NCH_2), 36.8 (CH_2), 29.7 (CH_2), 28.7 (CHAr), 28.5 (CMe_3) 26.2 (C), 16.7 (CH*C*H_2); HRMS (ESI) *m/z* calcd for $C_{18}H_{26}N_2O_4S$ $(M + Na)^+$ 389.1505, found 389.1508 (–0.6 ppm error).

Lab book reference: ARG-2-136

***tert*-Butyl 1-(pyrimidin-5-yl)-5-azaspiro[2.3]hexane-5-carboxylate 28**

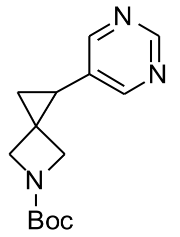

28

Using general procedure B, MIDA boronate **1c** (85 mg, 0.25 mmol, 1.0 eq.), Cs_2CO_3 (244 mg, 0.75 mmol, 3.0 eq.), 5-bromopyrimidine (56 mg, 0.35 mmol, 1.4 eq.), $Pd(OAc)_2$ (2.8 mg, 0.01 mmol, 0.05 eq.) and PCy_3 (7.0 mg, 0.02 mmol, 0.10 eq.) in toluene (5 mL) and H_2O (0.5 mL) gave the crude product. Purification by flash column chromatography on silica with 1:4 hexane-EtOAc as eluent gave arylated azetidine **28** (31 mg, 47%) as a white solid, mp 111-113 °C; R_F (3:7 hexane-EtOAc) 0.22; IR (ATR) 2875, 1696 (C=O), 1413, 1379, 1365, 1122, 771, 728 cm^{-1}; ^{1}H NMR (400 MHz, $CDCl_3$) δ 9.02 (s, 1H, Ar), 8.32 (s, 2H, Ar), 4.06 (d, *J* = 8.0 Hz, 1H, NCH), 4.03 (d, *J* = 8.0 Hz, 1H, NCH), 3.94 (d, *J* = 8.5 Hz, 1H, NCH), 3.68 (d, *J* = 8.5 Hz, 1H, NCH), 2.01 (dd, *J* = 9.0, 6.5 Hz, 1H, C*H*Ar), 1.40–1.33 (m, 10H, CMe_3, CH), 1.13 (dd, *J* = 6.5, 6.5 Hz, 1H, CH); ^{13}C NMR (100.6 MHz, $CDCl_3$) δ 156.6 (Ar), 155.9 (C=O), 155.5 (Ar), 132.8 (*ipso*-Ar), 79.8 (O*C*Me_3), 56.5 (br, NCH_2), 53.4 (br, NCH_2), 28.4 (C*Me*$_3$), 23.9 (C), 20.9 (CHAr), 16.9 (CH*C*H_2); HRMS (ESI) *m/z* calcd for $C_{14}H_{19}N_3O_2$ $(M + Na)^+$ 284.1369, found 284.1369 (+0.2 ppm error).

Lab book reference: ARG-2-161

Using general procedure A, MIDA boronate **1c** (90 mg, 0.27 mmol, 1.0 eq.), 5-bromopyrimidine (59 mg, 0.37 mmol, 1.4 eq.), $Pd(OAc)_2$ (9.0 mg, 0.040 mmol, 0.15 eq.), PCy_3 (22 mg, 0.080 mmol, 0.30 eq.) and Cs_2CO_3 (520 mg, 1.60 mmol, 6.0 eq.) were reacted in toluene (3.0 mL) and H_2O (0.16 mL) to give the crude product. Purification by flash column chromatography on silica with EtOAc as eluent gave pyrimidyl cyclopropane **28** as a colourless gum that solidified on standing (54 mg, 78%).

Lab book reference JRD_IX_1

A mixture of potassium trifluoroborate **S7** (77 mg, 0.27 mmol, 1.0 eq.), 5-bromopyrimidine (63 mg, 0.40 mmol, 1.5 eq.), $Pd(dppf)Cl_2$ (20 mg, 0.027 mmol, 0.1 eq.) and Cs_2CO_3 (303 mg, 0.93 mmol, 3.5 eq.) in THF (0.90 mL) and H_2O (0.32 mL) was stirred at rt in a sealed tube and the suspension was degassed by sparging with Ar for 20 min. The resulting mixture was stirred and heated at 100 °C for 18 h. The mixture

was allowed to cool to rt, water (5 mL) and CH_2Cl_2 (5 mL) were added and the two layers were separated. The aqueous layer was extracted with CH_2Cl_2 (3 × 5 mL). The combined organic layers were dried (Na_2SO_4) and evaporated under reduced pressure to give the crude product. Purification by flash column chromatography on silica with EtOAc as eluent gave cyclopropane **28** as a colourless gum (26 mg, 37%).

Lab book reference JRD_IX_1

***tert*-Butyl 1-(4-hydroxy-3-methoxyphenyl)-5-azaspiro[2.3]hexane-5-carboxylate 29**

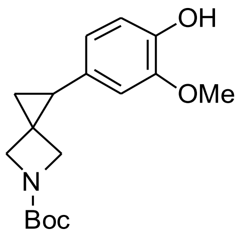

29

Using general procedure B, MIDA boronate **1c** (85 mg, 0.25 mmol, 1.0 eq.), Cs_2CO_3 (244 mg, 0.75 mmol, 3.0 eq.), 4-bromo-2-methoxyphenol (71 mg, 0.35 mmol, 1.4 eq.), $Pd(OAc)_2$ (2.8 mg, 0.01 mmol, 0.05 eq.) and PCy_3 (7.0 mg, 0.02 mmol, 0.10 eq.) in toluene (5 mL) and H_2O (0.5 mL) gave the crude product. Purification by flash column chromatography on silica with 8:2 hexane-EtOAc as eluent gave arylated azetidine **29** (36 mg, 47%) as an off-white gum, R_F (4:1 hexane-EtOAc) 0.11; IR (ATR) 3331 (OH), 2934, 2251, 1681 (C=O), 1517, 1417, 1365, 1157, 1121, 729 cm^{-1}; ^{1}H NMR (400 MHz, $CDCl_3$) δ 6.83 (d, J = 8.0 Hz, 1H, Ar), 6.51 (d, J = 2.0 Hz, 1H, Ar), 6.40 (dd, J = 8.0, 2.0 Hz, 1H, Ar), 5.63 (br s, 1H, OH), 4.05 (d, J = 8.0 Hz, 1H, NCH), 4.00 (d, J = 8.0 Hz, 1H, NCH), 3.89–3.85 (m, 1H, NCH), 3.87 (s, 3H, OMe), 3.71 (d, J = 8.5 Hz, 1H, NCH), 2.01 (dd, J = 9.5, 6.0 Hz, 1H, C*H*Ar), 1.42 (s, 9H, CMe_3), 1.17 (dd, J = 9.5, 6.0 Hz, 1H, CH), 0.99 (dd, J = 6.0 Hz, 1H, CH); ^{13}C NMR (100.6 MHz, $CDCl_3$) (rotamers) δ 156.2 (C=O), 146.6 (*ipso*-Ar), 144.1 (*ipso*-Ar), 130.8 (*ipso*-Ar), 119.1 (Ar) , 118.9 (Ar), 114.6 (Ar), 114.4 (Ar), 110.0 (Ar), 109.8 (Ar), 79.6 (*C*Me_3), 57.5 (br, NCH_2), 56.1 (OMe), 55.9 (OMe), 53.1 (br, NCH_2), 28.5 (C*Me_3*), 28.4 (C*Me_3*), 25.6 (CHAr), 25.4 (CHAr), 22.6 (C), 16.4 (CH*CH_2*); HRMS (ESI) *m/z* calcd for $C_{17}H_{23}NO_4$ $(M + Na)^+$ 328.1519, found 329.1518 (+0.3 ppm error).

Lab book reference: ARG-2-153

***tert*-Butyl 1-(2,5-difluorophenyl)-5-azaspiro[2.3]hexane-5-carboxylate 30**

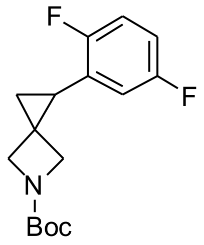

30

Using general procedure B, MIDA boronate **1c** (85 mg, 0.25 mmol, 1.0 eq.), Cs_2CO_3 (244 mg, 0.75 mmol, 3.0 eq.), 2,5-difluorobromobenzene (68 mg, 0.35 mmol, 1.4 eq.), $Pd(OAc)_2$ (2.8 mg, 0.01 mmol, 0.05 eq.) and PCy_3 (7.0 mg, 0.02 mmol, 0.10 eq.) in toluene (5 mL) and H_2O (0.5 mL) gave the crude product. Purification by flash column chromatography on silica with 9:1 hexane-EtOAc as eluent gave arylated azetidine **30** (45 mg, 61%) as a white solid, mp 80-82 °C; R_F (2:3 hexane-EtOAc) 0.52; IR (ATR) 2976, 1695 (C=O), 1497, 1365, 1179, 1120, 772 cm^{-1}; ^{1}H NMR (400 MHz, $CDCl_3$) δ 7.06–6.95 (m, 1H, Ar), 6.88–6.80 (m, 1H, Ar), 6.43–6.35 (m, 1H, Ar), 4.11 (d, J = 8.0 Hz, 1H, NCH), 4.04 (d, J = 8.0 Hz, 1H, NCH), 3.89 (d, J = 8.5 Hz, 1H, NCH), 3.66 (d, J = 8.5 Hz, 1H, NCH), 2.24 (dd, J = 9.0, 6.5 Hz, 1H, C*H*Ar), 1.43 (s, 9H CMe_3), 1.28 (dd, J = 9.0, 6.0 Hz, 1H, CH), 1.10 (dd, J = 6.5, 6.0 Hz, 1H, CH); ^{13}C NMR (100.6 MHz, $CDCl_3$) δ 158.8 (d, J = 239.5 Hz, *ipso*-Ar), 158.0 (d, J = 241.5 Hz, *ipso*-Ar), 156.1 (C=O), 116.4 (dd, J = 24.5, 8.5 Hz, Ar), 113.8 (dd, J = 24.5, 8.5 Hz, Ar), 113.5 (dd, J = 25.0, 5.0 Hz, Ar), 79.6 (O*C*Me_3), 56.1 (NCH_2, only resolved in HSQC), 54.8 (NCH_2, only resolved in HSQC), 28.4 (C*Me₃*), 23.2 (C), 19.6 (d, J = 4.0 Hz, CHAr), 16.0 (*C*HCH_2) (1 × *ipso*-Ar resonance not resolved); HRMS (ESI) *m/z* calcd for $C_{16}H_{19}F_2NO_2$ (M + Na)$^+$ 318.1276, found 318.1276 (+0.1 ppm error).

Lab book reference: ARG-2-151

***tert*-Butyl 1-[1-(4-methylbenzenesulfonyl)-1H-indol-3-yl]-5-azaspiro[2.3]hexane-5-carboxylate 31**

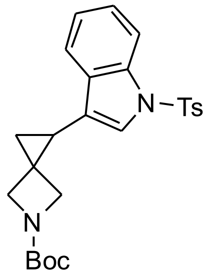

31

Using general procedure B, MIDA boronate **1c** (85 mg, 0.25 mmol, 1.0 eq.), Cs_2CO_3 (244 mg, 0.75 mmol, 3.0 eq.), *N*-Ts-3-bromoindole (123 mg, 0.35 mmol, 1.4 eq.), $Pd(OAc)_2$ (2.8 mg, 0.01 mmol, 0.05 eq.) and PCy_3 (7.0 mg, 0.02 mmol, 0.10 eq.) in toluene (5 mL) and H_2O (0.5 mL) gave the crude product. Purification by flash column chromatography on silica with 8:2 hexane-EtOAc as eluent gave arylated azetidine **31** (69 mg, 61%) as a pale-yellow oil, R_F (7:3 hexane-EtOAc) 0.38; IR (ATR) 2976, 1694 (C=O), 1365, 1172, 1118, 737, 669, 571, 537 cm^{-1}; ^1H NMR (400 MHz, $CDCl_3$) δ 7.97 (d, *J* = 8.0 Hz, 1H, Ar), 7.71–7.67 (m, 2H, Ar), 7.57 (d, *J* = 7.5 Hz, 1H, Ar), 7.33 (dd, *J* = 8.0, 8.0 Hz, 1H, Ar), 7.26 (dd, *J* = 8.0, 7.5 Hz, 1H, Ar), 7.21–7.17 (m, 2H, Ar), 7.08 (s, 1H, Ar), 4.11 (d, *J* = 8.0 Hz, 1H, NCH), 4.07 (d, *J* = 8.0 Hz, 1H, NCH), 3.77 (d, *J* = 8.5 Hz, 1H, NCH), 3.44 (d, *J* = 8.5 Hz, 1H, NCH), 2.31 (s, 3H, Me), 2.04 (dd, *J* = 9.0, 6.0 Hz, 1H, C*H*Ar), 1.41 (s, 9H, CMe_3), 1.26 (dd, *J* = 9.0, 6.0 Hz, 1H, CH), 1.07 (dd, *J* = 6.0, 6.0 Hz, 1H, CH); ^{13}C NMR (100.6 MHz, $CDCl_3$) δ 156.1 (C=O), 145.0 (*ipso*-Ar), 135.4 (*ipso*-Ar), 135.0 (*ipso*-Ar), 131.5 (*ipso*-Ar), 129.9 (Ar), 126.7 (Ar), 125.1 (Ar), 123.5 (Ar), 121.9 (Ar), 121.8 (*ipso*-Ar), 119.5 (Ar), 113.9 (Ar), 79.5 (O*C*Me_3), 57.4 (br, NCH_2), 54.9 (br, NCH_2), 28.4 (CMe_3), 22.3 (C), 21.6 (Me), 16.2 (CHAr), 15.2 (CHCH_2); HRMS (ESI) *m/z* calcd for $C_{25}H_{29}N_2O_4S$ $(M + Na)^+$ 475.1662, found 475.1673 (–2.3 ppm error).

Lab book reference: ARG-2-152

***tert*-Butyl (1*R**,3*S**)-1-(pyrimidin-5-yl)-5-azaspiro[2.4]heptane-5-carboxylate 32**

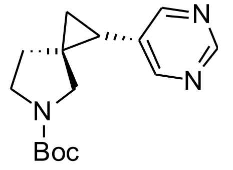

32

Using general procedure B, BMIDA building block **1d** (88 mg, 0.25 mmol, 1.0 eq.), 5-bromopyrimidine (56 mg, 0.35 mmol, 1.4 eq.), PCy_3 (7 mg, 25 μmol, 0.10 eq.), Cs_2CO_3 (244 mg, 0.75 mmol, 3.0 eq.) and $Pd(OAc)_2$ (3 mg, 13 μmol, 0.05 eq.) in toluene (5.0 mL) and water (0.5 mL) gave the crude product. Purification by flash column chromatography on silica with EtOAc as eluent gave cyclopropyl pyrimidine **32** (39 mg, 57%) as a pale-yellow oil, R_F (EtOAc) 0.24; IR (ATR) 2974, 2931, 2869, 1688 (C=O), 1554, 1398, 1166, 1107, 882, 771, 729, 631 cm^{-1}; ^1H NMR (400 MHz, $CDCl_3$) δ 9.05 (s, 1H, Ar), 8.52 (s, 2H, Ar), 3.51–3.35 (m, 3H, NCH), 3.34–3.21 (m, 1H, NCH), 2.13–2.04 (m, 1H, CH), 1.72–1.61 (m, 1H, CH), 1.50–1.40 (m, 1H, CH), 1.44 (s, 9H, CMe_3), 1.32–1.25 (m, 1H, CH), 1.20 (dd, J = 6.0, 6.0 Hz, 1H, CH); ^{13}C NMR (100.6 MHz, $CDCl_3$) (rotamers) δ 156.8 (Ar), 156.6 (Ar), 154.5 (C=O), 132.6 (*ipso*-Ar), 79.6 (O*C*Me3), 54.5 (NCH_2), 54.0 (NCH_2), 45.9 (NCH_2), 45.5 (NCH_2), 30.1 (C), 29.5 (C), 28.6 (C*Me*3), 22.4 (CH), 22.3 (CH), 15.4 (CH_2), 15.1 (CH_2); MS (ESI) *m/z* 298 $(M + Na)^+$; HRMS (ESI) *m/z* calcd for $C_{15}H_{21}N_3O_2$ $(M + Na)^+$ 298.1526, found 298.1524 (+0.8 ppm error).

Lab Book Reference: SY-2-134

Using general procedure A, BMIDA building block **1d** (176 mg, 0.50 mmol, 1.0 eq.), 5-bromopyrimidine (111 mg, 0.70 mmol, 1.4 eq.), PCy_3 (42 mg, 0.15 mmol, 0.30 eq.), Cs_2CO_3 (977 mg, 3. mmol, 6.0 eq.) and $Pd(OAc)_2$ (17 mg, 75 μmol, 0.15 eq.) in toluene (5.0 mL) and water (0.5 mL) gave the crude product. Purification by flash column chromatography on silica with EtOAc as eluent gave cyclopropyl pyrimidine **32** (94 mg, 68%) as a pale-yellow oil.

Lab Book Reference: SY-2-89

***tert*-Butyl (1*R**,3*S**)-1-(3,5-bis(trifluoromethyl)phenyl)-5-azaspiro[2.4]heptane-5-carboxylate 33**

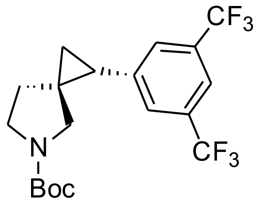

33

Using general procedure B, BMIDA building block **1d** (88 mg, 0.25 mmol, 1.0 eq.), 3,5-bis(trifluoromethyl)bromobenzene (60 μL, 0.35 mmol, 1.4 eq.), PCy_3 (7 mg, 25 μmol, 0.10 eq.), Cs_2CO_3 (244 mg, 0.75 mmol, 3.0 eq.) and $Pd(OAc)_2$ (3 mg, 13 μmol, 0.05 eq.) in toluene (5.0 mL) and water (0.5 mL) gave the crude product. Purification by flash column chromatography on silica with 90:10 hexane-EtOAc as eluent gave aryl cyclopropane **33** (58 mg, 57%) as a colourless oil, R_F (8:2 hexane-EtOAc) 0.57; IR (ATR) 2978, 2934, 2872, 1692 (C=O), 1478, 1402, 1368, 1276, 1168, 1127, 894, 844, 772, 706, 682 cm^{-1}; ^{1}H NMR (400 MHz, $CDCl_3$) δ 7.71 (s, 1H, Ar), 7.56 (s, 2H, Ar), 3.52–3.36 (m, 3H, NCH), 3.36–3.19 (m, 1H, NCH), 2.34–2.23 (m, 1H, CH), 1.69–1.59 (m, 1H, CH), 1.46 (s, 9H, CMe_3), 1.44–1.35 (m, 1H, CH), 1.35–1.26 (m, 1H, CH), 1.26–1.19 (m, 1H, CH); ^{13}C NMR (100.6 MHz, $CDCl_3$) (rotamers) δ 154.5 (C=O), 141.9 (*ipso*-Ar), 141.7 (*ipso*-Ar), 131.7 (q, *J* = 33.0 Hz, *ipso*-Ar), 128.3 (q, *J* = 4.0 Hz, Ar), 123.4 (q, *J* = 273.0 Hz, CF_3), 120.2 (Ar), 79.6 (O*C*Me_3), 54.7 (NCH_2), 54.2 (NCH_2), 46.0 (NCH_2), 45.6 (NCH_2), 30.6 (C), 30.0 (C), 29.1 (CH), 28.6 (C*Me*$_3$), 28.5 (CH), 27.1 (CH_2), 16.1 (CH_2); MS (ESI) *m/z* 432 (M + Na)$^+$; HRMS (ESI) *m/z* calcd for $C_{19}H_{21}F_6NO_2$ (M + Na)$^+$ 432.1369, found 432.1373 (–1.1 ppm error).

Lab Book Reference: SY-2-125

tert-Butyl (1*R,3*S**)-1-(2-(methoxycarbonyl)phenyl)-5-azaspiro[2.4]heptane-5-carboxylate 34**

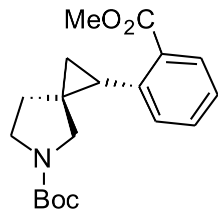

34

Using general procedure B, BMIDA building block **1d** (88 mg, 0.25 mmol, 1.0 eq.), methyl 2-bromobenzoate (49 μL, 0.35 mmol, 1.4 eq.), PCy_3 (7 mg, 25 μmol, 0.10 eq.), Cs_2CO_3 (244 mg, 0.75 mmol, 3.0 eq.) and $Pd(OAc)_2$ (3 mg, 13 μmol, 0.05 eq.) in toluene (5.0 mL) and water (0.5 mL) gave the crude product. Purification by flash column chromatography on silica with 80:20 hexane-EtOAc as eluent gave cyclopropyl benzoate **34** (53 mg, 64%) as a pale yellow oil, R_F (8:2 hexane-EtOAc) 0.25; IR (ATR) 2974, 2867, 1722 (C=O, ester), 1689 (C=O, Boc), 1398, 1365, 1293, 1253, 1169, 1131, 1106, 1078, 881, 771, 736, 715 cm^{-1}; ^{1}H NMR (400 MHz, $CDCl_3$) (55:45 mixture of rotamers) δ 7.90 (dd, J = 7.5, 1.5 Hz, 1H, Ar), 7.43 (ddd, J = 7.5, 7.5, 1.5 Hz, 1H, Ar), 7.30–7.24 (m, 1H, Ar), 7.16 (d, J = 7.5 Hz, 1H, Ar), 3.86 (s, 3H, OMe), 3.53 (d, J = 10.5 Hz, 1H, NCH), 3.42–3.27 (m, 2H, NCH), 3.25–3.11 (m, 1H, NCH), 2.73–2.62 (m, 1H, CH), 1.47 z(s, 4.95H, CMe_3), 1.45 (s, 4.05H, CMe_3), 1.44–1.34 (m, 1H, CH), 1.31–1.19 (m, 1H, CH), 1.16–1.07 (m, 2H, CH); ^{13}C NMR (100.6 MHz, $CDCl_3$) (rotamers) δ 168.4 (C=O, ester), 168.1 (C=O, ester), 154.6 (C=O, Boc), 154.5 (C=O, Boc), 140.1 (*ipso*-Ar), 139.8 (*ipso*-Ar), 132.1 (Ar), 131.8 (*ipso*-Ar), 131.5 (*ipso*-Ar), 130.9 (Ar), 130.8 (Ar), 128.8 (Ar), 126.4 (Ar), 79.1 (O*C*Me$_3$), 54.7 (NCH_2), 54.2 (NCH_2), 52.1 (OMe), 46.0 (NCH_2), 45.6 (NCH_2), 29.6 (CH), 29.0 (C), 28.9 (C), 28.7 (C*Me*$_3$), 27.6 (CH_2), 27.1 (CH_2), 15.5 (CH_2), 15.2 (CH_2); MS (ESI) *m/z* 354 (M + Na)$^+$; HRMS (ESI) *m/z* calcd for $C_{19}H_{25}NO_4$ (M + Na)$^+$ 354.1676, found 354.1676 (0.0 ppm error).

Lab Book Reference: SY-2-128

***tert*-Butyl (1*R**,3*S**)-1-(quinazolin-7-yl)-5-azaspiro[2.4]heptane-5-carboxylate 35**

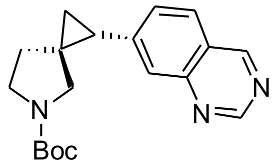

35

Using general procedure B, BMIDA building block **1d** (88 mg, 0.25 mmol, 1.0 eq.), 6-bromoquinazoline (73 mg, 0.35 mmol, 1.4 eq.), PCy_3 (7 mg, 25 μmol, 0.10 eq.), Cs_2CO_3 (244 mg, 0.75 mmol, 3.0 eq.) and $Pd(OAc)_2$ (3 mg, 13 μmol, 0.05 eq.) in toluene (5.0 mL) and water (0.5 mL) gave the crude product. Purification by flash column chromatography on silica with 70:30 EtOAc-hexane as eluent gave cyclopropyl quinazoline **35** (47 mg, 58%) as a colourless oil, R_F (8:2 EtOAc-hexane) 0.33; IR (ATR) 2972, 2927, 2866, 1689 (C=O), 1621, 1400, 1364, 1167, 1109, 927, 889, 771, 730, 631 cm^{-1}; ^{1}H NMR (400 MHz, $CDCl_3$) (55:45 mixture of rotamers) δ 9.33 (s, 1H, Ar), 9.26 (s, 1H, Ar), 7.83 (d, *J* = 8.5 Hz, 1H, Ar), 7.67 (s, 1H, Ar), 7.51 (dd, *J* = 8.5, 1.5 Hz, 1H, Ar), 3.50–3.31 (m, 3H, NCH), 3.28–3.12 (m, 1H, NCH), 2.41–2.31 (m, 1H, CH), 1.76–1.64 (m, 1H, CH), 1.45 (s, 4H, CMe_3), 1.43 (s, 5H, CMe_3), 1.42–1.39 (m, 1H, CH), 1.39–1.26 (m, 2H, CH); ^{13}C NMR (100.6 MHz, $CDCl_3$) (rotamers) δ 159.7 (Ar), 155.5 (Ar), 154.5 (C=O), 154.5 (C=O) 150.2 (*ipso*-Ar), 146.9 (*ipso*-Ar), 146.7 (*ipso*-Ar), 129.8 (Ar), 127.0 (Ar), 127.0 (Ar), 125.6 (Ar), 125.6 (Ar), 123.8 (Ar), 79.4 (O*C*Me$_3$), 54.9 (NCH_2), 54.4 (NCH_2), 46.0 (NCH_2), 45.6 (NCH_2), 31.6 (C), 30.9 (C), 28.8 (CH) 28.6 (C*Me*$_3$), 28.1 (CH_2), 15.9 (CH_2); MS (ESI) *m/z* 348 (M + Na)$^+$; HRMS (ESI) *m/z* calcd for $C_{19}H_{23}N_3O_2$ (M + Na)$^+$ 348.1682, found 348.1683 (–0.1 ppm error).

Lab Book Reference: SY-2-147

***tert*-Butyl (1*R**,3*S**)-1-(2-chloropyridin-4-yl)-5-azaspiro[2.4]heptane-5-carboxylate 36**

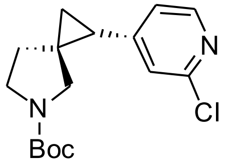

36

Using general procedure B, BMIDA building block **1d** (88 mg, 0.25 mmol, 1.0 eq.), 2-chloro-4-bromopyridine (67 mg, 0.35 mmol, 1.4 eq.), PCy_3 (7 mg, 25 μmol, 0.10 eq.), Cs_2CO_3 (244 mg, 0.75 mmol, 3.0 eq.) and $Pd(OAc)_2$ (3 mg, 13 μmol, 0.05 eq.) in toluene (5.0 mL) and water (0.5 mL) gave the crude product. Purification by flash column chromatography on silica with 80:20 hexane-EtOAc as eluent gave cyclopropyl chloropyridine **36** (54 mg, 70%) as a pale yellow oil, R_F (8:2 hexane-EtOAc) 0.20; IR (ATR) 2975, 2925, 2869, 1687 (C=O), 1591, 1400, 1365, 1168, 1109, 1087, 990, 869, 772 cm^{-1}; ^1H NMR (400 MHz, $CDCl_3$) δ 8.24 (d, J = 5.0 Hz, 1H, Ar), 7.05 (d, J = 1.5 Hz, 1H, Ar), 6.95 (dd, J = 5.0, 1.5 Hz, 1H, Ar), 3.50–3.32 (m, 3H, NCH), 3.31–3.15 (m, 1H, NCH), 2.16–2.05 (m, 1H, CH), 1.77–1.64 (m, 1H, CH), 1.51–1.35 (m, 10H, CH, CMe_3), 1.32–1.17 (m, 2H, CH); ^{13}C NMR (100.6 MHz, $CDCl_3$) (rotamers) δ 154.4 (C=O), 152.1 (*ipso*-Ar), 151.9 (*ipso*-Ar), 151.8 (Ar), 149.4 (Ar), 123.4 (Ar), 122.1 (Ar), 79.5 (O*C*Me_3), 54.7 (NCH_2), 54.3 (NCH_2), 46.0 (NCH_2), 45.9 (NCH_2), 31.5 (C), 30.9 (C), 28.7 (CH), 28.6 (C*Me*$_3$), 28.1 (CH_2), 26.9 (CH_2), 16.1 (CH_2); MS (ESI) *m/z* 331 [(^{35}M + Na)$^+$; HRMS (ESI) *m/z* calcd for $C_{16}H_{21}{}^{35}ClN_2O_2$ (^{35}M + Na)$^+$ 331.1184, found 331.1187 (–1.0 ppm error).

Lab Book Reference: SY-2-115

***tert*-Butyl (1*R**,3*R**)-1-(pyrimidin-5-yl)-5-azaspiro[2.4]heptane-5-carboxylate 37**

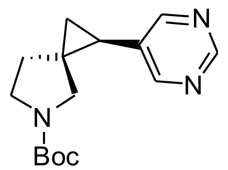

37

Using general procedure B, BMIDA building block **1e** (88 mg, 0.25 mmol, 1.0 eq.), 5-bromopyrimidine (56 mg, 0.35 mmol, 1.4 eq.), PCy_3 (7 mg, 25 μmol, 0.10 eq.), Cs_2CO_3 (244 mg, 0.75 mmol, 3.0 eq.) and $Pd(OAc)_2$ (3 mg, 13 μmol, 0.05 eq.) in toluene (5.0 mL) and water (0.5 mL) gave the crude product. Purification by flash column chromatography on silica with EtOAc as eluent gave cyclopropyl

pyrimidine **37** (61 mg, 88%) as a colourless oil, R_F (EtOAc) 0.27; IR (ATR) 2974, 2932, 2870, 1686 (C=O), 1554, 1399, 1166, 1109, 884, 771, 728, 631 cm^{-1}; ^{1}H NMR (400 MHz, $CDCl_3$) (60:40 mixture of rotamers) δ 9.07 (s, 0.6H, Ar), 9.04 (s, 0.4H, Ar), 8.51–8.46 (m, 2H, Ar), 3.58–3.45 (m, 2H, NCH), 3.13 (d, J = 11.0 Hz, 0.4H, NCH), 3.07 (d, J = 11.0 Hz, 0.6H, NCH), 2.92 (d, J = 11.0 Hz, 0.4H, NCH), 2.85 (d, J = 11.0 Hz, 0.6H, NCH), 2.05 (dd, J = 9.0, 6.0 Hz, 1H, CH), 2.01–1.90 (m, 2H, CH), 1.42 (s, 3.6H, CMe_3), 1.35 (s, 5.4H, CMe_3), 1.32–1.24 (m, 1H, CH), 1.23–1.15 (m, 1H, CH); ^{13}C NMR (100.6 MHz, $CDCl_3$) (rotamers) δ 156.9 (Ar), 156.7 (Ar), 156.5 (Ar), 154.4 (C=O), 132.6 (*ipso*-Ar), 79.6 (O*C*Me$_3$), 48.8 (NCH_2), 46.0 (NCH_2), 45.5 (NCH_2), 35.4 (CH), 34.7 (CH), 30.2 (C), 29.6 (C), 28.5 (C*Me*$_3$), 22.6 (CH_2), 22.4 (CH_2), 15.9 (CH_2), 15.3 (CH_2); MS (ESI) *m/z* 298 (M + Na)$^+$; HRMS (ESI) *m/z* calcd for $C_{15}H_{21}N_3O_2$ (M + Na)$^+$ 298.1526, found 298.1527 (–0.4 ppm error).

Lab Book Reference: SY-2-135

Using general procedure A, BMIDA building block **1e** (176 mg, 0.50 mmol, 1.0 eq.), 5-bromopyrimidine (111 mg, 0.70 mmol, 1.4 eq.), PCy_3 (42 mg, 0.15 mmol, 0.30 eq.), Cs_2CO_3 (977 mg, 3.00 mmol, 6.0 eq.) and $Pd(OAc)_2$ (17 mg, 75 μmol, 0.15 eq.) in toluene (5.0 mL) and water (0.5 mL) gave the crude product. Purification by flash column chromatography on silica with EtOAc as eluent gave cyclopropyl pyrimidine **37** (119 mg, 86%) as a colourless oil

Lab Book Reference: SY-2-92

***tert*-Butyl (1*R**,3*R**)-1-(4-nitrophenyl)-5-azaspiro[2.4]heptane-5-carboxylate 38**

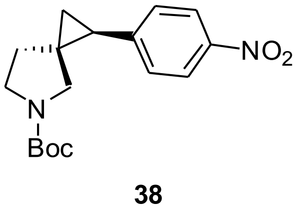

38

Using general procedure B, BMIDA building block **1e** (88 mg, 0.25 mmol, 1.0 eq.), 1-bromo-4-nitrobenzene (71 mg, 0.35 mmol, 1.4 eq.), PCy_3 (7 mg, 25 μmol, 0.10 eq.), Cs_2CO_3 (244 mg, 0.75 mmol, 3.0 eq.) and $Pd(OAc)_2$ (3 mg, 13 μmol, 0.05 eq.) in toluene (5.0 mL) and water (0.5 mL) gave the crude product. Purification by flash column chromatography on silica with 80:20 hexane-EtOAc as eluent gave cyclopropyl nitrobenzene **38** (72 mg, 90%) as an off-white solid, mp 88–89 °C; R_F (8:2 hexane-EtOAc)

0.24; IR (ATR) 2974, 2934, 2869, 1687 (C=O), 1597, 1516 (NO_2), 1400, 1342 (NO_2), 1167, 1108, 856, 771, 739, 700 cm^{-1}; ^{1}H NMR (400 MHz, $CDCl_3$) (50:50 mixture of rotamers) δ 8.14 (d, *J* = 8.0 Hz, 1H, Ar), 8.10 (d, *J* = 8.0 Hz, 1H, Ar), 7.23 (d, *J* = 8.0 Hz, 1H, Ar), 7.20 (d, *J* = 8.0 Hz, 1H, Ar), 3.58–3.42 (m, 2H, NCH), 3.15 (d, *J* = 11.0 Hz, 0.5H, NCH), 3.09 (d, *J* = 11.0 Hz, 0.5H, NCH), 2.91 (d, *J* = 11.0 Hz, 0.5H, NCH), 2.80 (d, *J* = 11.0 Hz, 0.5H, NCH), 2.20 (dd, *J* = 8.5, 6.5 Hz, 1H, C*H*Ar), 2.05–1.87 (m, 2H, CH), 1.41 (s, 4.5H, CMe_3), 1.32 (s, 4.5H, CMe_3), 1.30–1.24 (m, 2H, CH); ^{13}C NMR (100.6 MHz, $CDCl_3$) (rotamers) δ 154.5 (C=O), 147.2 (*ipso*-Ar), 146.4 (*ipso*-Ar), 128.8 (Ar), 128.5 (Ar), 123.7 (Ar), 79.5 (O*C*Me_3), 48.7 (NCH_2), 46.0 (NCH_2), 45.5 (NCH_2), 35.7 (*C*HAr), 35.0 (*C*HAr), 31.3 (C), 30.7 (C), 28.5 (C*Me*$_3$), 28.1 (CH_2), 27.7 (CH_2), 17.6 (CH_2), 16.5 (CH_2); MS (ESI) *m/z* 341 (M + Na)$^+$; HRMS (ESI) *m/z* calcd for $C_{17}H_{22}N_2O_4$ (M + Na)$^+$ 341.1472, found 341.1472 (–0.1 ppm error).

Lab Book Reference: SY-2-116

***tert*-Butyl (1*R**,3*R**)-1-(pyridin-3-yl)-5-azaspiro[2.4]heptane-5-carboxylate 39**

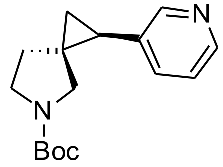

39

Using general procedure B, BMIDA building block **1e** (88 mg, 0.25 mmol, 1.0 eq.), 3-bromopyridine (55 mg, 0.35 mmol, 1.4 eq.), PCy_3 (7 mg, 25 μmol, 0.10 eq.), Cs_2CO_3 (244 mg, 0.75 mmol, 3.0 eq.) and $Pd(OAc)_2$ (3 mg, 13 μmol, 0.05 eq.) in toluene (5.0 mL) and water (0.5 mL) gave the crude product. Purification by flash column chromatography on silica with 90:10 EtOAc-hexane as eluent gave cyclopropyl pyridine **39** (41 mg, 59%) as a colourless oil, R_F (EtOAc) 0.40; IR (ATR) 2975, 2932, 2869, 1687 (C=O), 1400, 1365, 1167, 1109, 884, 771, 730, 714 cm^{-1}; ^{1}H NMR (400 MHz, $CDCl_3$) (50:50 mixture of rotamers) δ 8.46–8.36 (m, 2H, Ar), 7.36 (d, *J* = 8.0 Hz, 0.5H, Ar), 7.28 (d, *J* = 8.0 Hz, 0.5H, Ar), 7.23–7.11 (m, 1H, Ar), 3.55–3.39 (m, 2H, NCH), 3.08 (d, *J* = 11.0 Hz, 0.5H, NCH), 3.02 (d, *J* = 11.0 Hz, 0.5H, NCH), 2.91 (d, *J* = 11.0 Hz, 0.5H, NCH), 2.80 (d, *J* = 11.0 Hz, 0.5H, NCH), 2.12–2.03 (m, 1H, CH), 1.99–1.82 (m, 2H, CH), 1.40 (s, 4.5H, CMe_3), 1.32 (s, 4.5H, CMe_3), 1.24–1.08 (m, 1H, CH); ^{13}C NMR (100.6 MHz, $CDCl_3$) (rotamers) δ 154.5 (C=O), 150.3 (Ar), 150.0 (Ar), 147.6 (Ar), 147.6 (Ar), 135.7 (Ar), 134.5 (*ipso*-Ar), 134.5 (Ar), 123.2 (Ar), 79.3 (O*C*Me_3), 48.8 (NCH_2), 46.0 (NCH_2), 45.5 (NCH_2), 35.3 (CH), 34.8 (CH), 29.8 (C), 29.2 (C), 28.53 (C*Me*$_3$), 28.50 (C*Me*$_3$), 25.2 (CH_2), 24.8

(CH_2), 16.1 (CH_2), 15.0 (CH_2); MS (ESI) *m/z* 297 (M + Na)$^+$; HRMS (ESI) *m/z* calcd for $C_{16}H_{22}N_2O_2$ (M + Na)$^+$ 297.1573, found 297.1575 (–0.4 ppm error).

Lab Book Reference: SY-2-124

3-[(1*R,3*R**)-5-[(*tert*-Butoxy)carbonyl]-5-azaspiro[2.4]heptan-1-yl]pyridin-1-ium-1-olate 40**

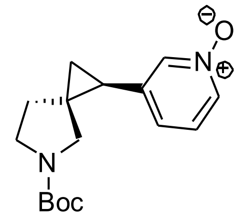

40

Using general procedure B, BMIDA building block **1e** (88 mg, 0.25 mmol, 1.0 eq.), 3-bromopyridine *N*-oxide (64 mg, 0.35 mmol, 1.4 eq.), PCy_3 (7 mg, 25 μmol, 0.10 eq.), Cs_2CO_3 (244 mg, 0.75 mmol, 3.0 eq.) and $Pd(OAc)_2$ (3 mg, 13 μmol, 0.05 eq.) in toluene (5.0 mL) and water (0.5 mL) gave the crude product. Purification by flash column chromatography on silica with 90:10 acetone-MeOH as eluent gave cyclopropyl *N*-oxide **40** (44 mg, 60%) as a colourless oil, R_F (9:1 acetone-MeOH) 0.30; IR (ATR) 2974, 2931, 2870, 2229, 1683 (C=O), 1401, 1365, 1270, 1164, 1111, 1011, 881, 729, 682 cm^{-1}; ^{1}H NMR (400 MHz, $CDCl_3$) (50:50 mixture of rotamers) δ 8.09–8.02 (m, 1H, Ar), 8.02–7.97 (m, 1H, Ar), 7.22–7.10 (m, 1H, Ar), 7.02 (d, *J* = 7.5 Hz, 0.5H, Ar), 6.89 (d, *J* = 7.5 Hz, 0.5H, Ar), 3.57–3.37 (m, 2H, NCH), 3.12 (d, *J* = 11.0 Hz, 0.5H, NCH), 3.04 (d, *J* = 11.0 Hz, 0.5H, NCH), 2.90 (d, *J* = 11.0 Hz, 0.5H, NCH), 2.80 (d, *J* = 11.0 Hz, 0.5H, NCH), 2.06–1.97 (m, 1H, CH), 1.96–1.82 (m, 2H, CH), 1.40 (s, 4.5H, CMe_3), 1.34 (s, 4.5H, CMe_3), 1.26–1.18 (m, 1H, CH), 1.15–1.06 (m, 1H, CH); ^{13}C NMR (100.6 MHz, $CDCl_3$) (rotamers) δ 154.4 (C=O), 139.4 (*ipso*-Ar), 138.9 (*ipso*-Ar), 138.8 (Ar), 137.2 (Ar), 137.1 (Ar), 126.7 (Ar), 125.6 (Ar), 125.2 (Ar), 79.6 (O*C*Me_3), 48.7 (NCH_2), 48.5 (NCH_2), 45.9 (NCH_2), 45.5 (NCH_2), 35.4 (CH), 34.6 (CH), 30.5 (C), 29.9 (C), 28.5 (C*Me*$_3$), 24.7 (CH_2), 16.0 (CH_2), 15.4 (CH_2); MS (ESI) *m/z* 313 (M + Na)$^+$; HRMS (ESI) *m/z* calcd for $C_{16}H_{22}N_2O_3$ (M + Na)$^+$ 313.1523, found 313.1527 (–1.5 ppm error).

Lab Book Reference: SY-2-121

***tert*-Butyl (1*R**,3*R**)-1-(1-methyl-1*H*-pyrazol-4-yl)-5-azaspiro[2.4]heptane-5-carboxylate 41**

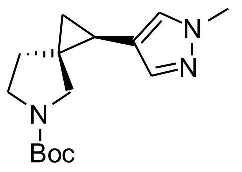

41

Using general procedure B, BMIDA building block **1e** (88 mg, 0.25 mmol, 1.0 eq.), 4-bromo-1-methyl-1*H*-pyrazole (36 μL, 0.35 mmol, 1.4 eq.), PCy_3 (7 mg, 25 μmol, 0.10 eq.), Cs_2CO_3 (244 mg, 0.75 mmol, 3.0 eq.) and $Pd(OAc)_2$ (3 mg, 13 μmol, 0.05 eq.) in toluene (5.0 mL) and water (0.5 mL) gave the crude product. Purification by flash column chromatography on silica with 80:20 hexane-EtOAc as eluent gave cyclopropyl pyrazole **41** (21 mg, 30%) as a colourless oil, R_F (8:2 hexane-EtOAc) 0.25; IR (ATR) 2974, 2930, 2869, 1687 (C=O), 1398, 1364, 1167, 1364, 1167, 1109, 987, 882, 771, 680 cm^{-1}; ^{1}H NMR (400 MHz, $CDCl_3$) (50:50 mixture of rotamers) δ 7.24 (s, 0.5H, Ar), 7.21 (s, 0.5H, Ar), 7.11 (s, 0.5H, Ar), 7.01 (s, 0.5H, Ar), 3.85 (s, 1.5H, Me), 3.81 (s, 1.5H, Me), 3.56–3.39 (m, 2H, NCH), 3.14–2.92 (m, 2H, NCH), 1.93–1.74 (m, 3H, CH), 1.44 (s, 4.5H, CMe_3), 1.39 (s, 4.5H, CMe_3), 1.11–1.04 (m, 1H, CH), 0.79 (dd, *J* = 5.5, 5.5 Hz, 1H, CH); ^{13}C NMR (100.6 MHz, $CDCl_3$) (rotamers) δ 154.7 (C=O), 139.1 (Ar), 138.9 (Ar), 129.0 (Ar), 128.1 (Ar), 120.0 (*ipso*-Ar), 79.2 (O*C*Me_3), 49.4 (NCH_2), 46.2 (NCH_2), 45.7 (NCH_2), 39.1 (Me), 39.0 (Me), 35.1 (*C*HAr), 34.5 (*C*HAr), 28.6 (C*Me*$_3$), 28.0 (C), 17.44 (CH_2) 17.35 (CH_2), 17.3 (CH_2), 16.3 (CH_2) (C not resolved); MS (ESI) *m/z* 300 (M + Na)$^+$; HRMS (ESI) *m/z* calcd for $C_{15}H_{23}N_3O_2$ (M + Na)$^+$ 300.1682, found 300.1681 (+0.5 ppm error).

Lab Book Reference: SY-2-129

***tert*-Butyl (1*R**,3'*S**,5*S**)-3'-(pyrimidin-5-yl)-8-azaspiro[bicyclo[3.2.1]octane-3,1'-cyclopropane]-8-carboxylate 43**

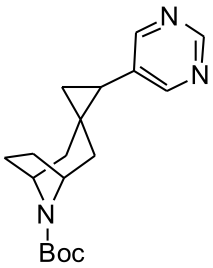

43

Using general procedure C, MIDA boronate **1f** (98 mg, 0.25 mmol, 1.0 eq.), 1 M $NaOH_{(aq)}$ (1 mL) and THF (1 mL) and then Cs_2CO_3 (244 mg, 0.75 mmol, 3.0 eq.), 5-bromopyrimidine (56 mg, 0.35 mmol, 1.4 eq.), $Pd(OAc)_2$ (2.8 mg, 0.01 mmol, 0.05 eq.) and PCy_3 (7.0 mg, 0.02 mmol, 0.10 eq.) in toluene (5 mL) and H_2O (0.5 mL) gave the crude product. Purification by flash column chromatography on silica with 80:20 hexane-EtOAc as eluent gave arylated tropane **43** (44 mg, 58%) as a white gum, R_F (7:3 hexane-EtOAc) 0.36; IR (ATR) 2976, 1683 (C=O), 1413, 1158, 1099, 727 cm^{-1}; ^{1}H NMR (400 MHz, $CDCl_3$) δ 9.01 (s, 1H, Ar), 8.49 (s, 2H, Ar), 4.33–4.10 (m, 2H, NCH), 2.38–2.22 (m, 1H, CH), 1.98–1.81 (m, 4H, CH), 1.72–1.52 (m, 2H, CH, C*H*Ar), 1.41 (s, 9H, CMe_3), 1.26–1.14 (m, 2H, CH), 1.06–0.96 (m, 1H, CH), 0.61–0.53 (m, 1H, CH); ^{13}C NMR (100.6 MHz, $CDCl_3$) (rotamers) δ 156.9 (Ar), 156.5 (Ar), 153.3 (C=O), 132.5 (*ipso*-Ar), 79.4 (O*C*Me_3), 54.5 (NCH), 54.0 (NCH), 53.7 (NCH), 53.3 (NCH), 42.0 (CH_2), 41.3 (CH_2), 37.4 (CH_2), 36.6 (CH_2), 28.6 (CMe_3), 28.0 (CH_2), 27.4 (CH_2), 21.4 (CH*C*H_2), 19.9 (C), 19.5 (CHAr); HRMS (ESI) *m/z* calcd for $C_{18}H_{25}N_3O_2$ $(M + Na)^+$ 338.1839, found 338.1839 (–0.7 ppm error).

Lab book reference: ARG-2-167

Using general procedure B, MIDA boronate **1f** (98 mg, 0.25 mmol, 1.0 eq.), Cs_2CO_3 (244 mg, 0.75 mmol, 3.0 eq.), 5-bromopyrimidine (56 mg, 0.35 mmol, 1.4 eq.), $Pd(OAc)_2$ (2.8 mg, 0.01 mmol, 0.05 eq.) and PCy_3 (7.0 mg, 0.02 mmol, 0.10 eq.) in toluene (5 mL) and H_2O (0.5 mL) gave the crude product. Purification by flash column chromatography on silica with 80:20 hexane-EtOAc as eluent gave arylated tropane **43** (22 mg, 29%) as a white gum and BMIDA building block **1e** (43 mg, 50%).

Lab book reference: ARG-2-162

Using general procedure B, pinacol boronate **S10** (91 mg, 0.25 mmol, 1.0 eq.), Cs_2CO_3 (244 mg, 0.75 mmol, 3.0 eq.), 5-bromopyrimidine (56 mg, 0.35 mmol, 1.4 eq.), $Pd(OAc)_2$ (2.8 mg, 0.01 mmol, 0.05 eq.) and PCy_3 (7.0 mg, 0.02 mmol, 0.10 eq.) in toluene (5 mL) and H_2O (0.5 mL) gave the crude product. Purification by flash column chromatography on silica with 80:20 hexane-EtOAc as eluent gave arylated tropane **43** (30 mg, 39%) as a white gum and pinacol boronate **S10** (47 mg, 52%).

Lab book reference: ARG-2-165

H_2O (0.5 mL), degassed by sparging with Ar for 30 min was added to a solution of cataCXium A Pd G3 (9 mg, 0.01 mmol, 0.05 eq.), cyclopropyl BF_3K **S11** (86 mg, 0.25 mmol, 1.0 eq.), Cs_2CO_3 (244 mg, 0.75 mmol, 3.0 eq.) and 5-bromopyrimidine (56 mg, 0.35 mmol, 1.4 eq.) in toluene (5.0 mL) at rt in a sealed tube under Ar. The resulting mixture was stirred and heated at 100 °C for 18 h under Ar. The solids were removed by filtration through Celite® and washed with EtOAc (5 mL) and water (5 mL). The two layers were separated and the aqueous layer was extracted with EtOAc (3 × 5 mL). The combined organic layers were washed with brine (10 mL), dried (Na_2SO_4) and evaporated under reduced pressure to give the crude product. Purification by flash column chromatography on silica with 4:1 hexane-EtOAc as eluent gave arylated piperidine **43** (4 mg, 6%) as a white gum.

Note: cyclopropyl BF_3K **S11** was very insoluble in the reaction solvent.

Lab book reference: ARG-3-165

***tert*-Butyl (1*R**,3'*S**,5*S**)-3'-(2-fluoropyridin-3-yl)-8-azaspiro[bicyclo[3.2.1]octane-3,1'-cyclopropane]-8-carboxylate 44**

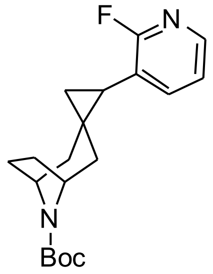

44

Using general procedure C, MIDA boronate **1f** (98 mg, 0.25 mmol, 1.0 eq.), 1 M $NaOH_{(aq)}$ (1 mL) and THF (1 mL) and then Cs_2CO_3 (244 mg, 0.75 mmol, 3.0 eq.), 3-bromo-2-fluoropyridine (62 mg, 0.35 mmol, 1.4 eq.), $Pd(OAc)_2$ (2.8 mg, 0.01 mmol, 0.05 eq.) and PCy_3 (7.0 mg, 0.02 mmol, 0.10 eq.) in toluene (5 mL) and H_2O (0.5 mL) gave the crude product. Purification by flash column chromatography on silica with 9:1 hexane-EtOAc as eluent gave arylated tropane **44** (57 mg, 69%) as an off-white solid, mp 136-138 °C; R_F (7:3 hexane-EtOAc) 0.42; IR (ATR) 2976, 1683 (C=O), 1389, 1157, 1100, 773, 729 cm^{-1}; ^1H NMR (400 MHz, $CDCl_3$) δ 8.11–7.94 (m, 1H, Ar), 7.47–7.37 (m, 1H, Ar), 7.11–7.00 (m, 1H, Ar), 4.36–3.96 (m, 2H, NCH), 2.47–2.29 (m, 1H, CH), 1.95–1.83 (m, 4H, CH), 1.73–1.58 (m, 2H, CH, C*H*Ar), 1.48–1.39 (m, 9H, CMe_3), 1.18–1.09 (m, 2H, CH), 1.04–0.95 (m, 1H, CH), 0.53–0.45 (m, 1H, CH); ^{13}C NMR (100.6 MHz, $CDCl_3$) (rotamers) δ 163.7 (d, *J* = 239.5 Hz, *ipso*-Ar), 153.5 (C=O), 144.9 (d, *J* = 14.5 Hz, Ar), 139.85 (d, *J* = 48.5 Hz, Ar), 121.6 (br, *ipso*-Ar), 121.0 (Ar), 79.4 (O*C*Me_3), 54.6 (NCH), 54.1 (NCH), 53.9 (NCH), 53.5 (NCH), 41.6 (CH_2), 40.9 (CH_2), 36.8 (CH_2), 36.1 (CH_2), 28.6 (C*Me*$_3$), 28.1 (br, *C*H_2), 27.4 (br, CH_2), 21.0 (C), 19.3 (CH*C*H_2), 18.5 (CHAr); HRMS (ESI) *m/z* calcd for $C_{19}H_{25}FN_2O_2$ $(M + Na)^+$ 355.1792, found 355.1797 (–1.2 ppm error).

Lab book reference: ARG-2-169

***tert*-Butyl (1*R**,3'*S**,5*S**)-3'-(4-carbamoylphenyl)-8-azaspiro[bicyclo[3.2.1]octane-3,1'-cyclopropane]-8-carboxylate 45**

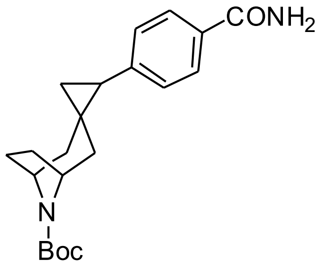

45

Using general procedure C, MIDA boronate **1f** (98 mg, 0.25 mmol, 1.0 eq.), 1 M $NaOH_{(aq)}$ (1 mL) and THF (1 mL) and then Cs_2CO_3 (244 mg, 0.75 mmol, 3.0 eq.), 4-bromobenzamide (70 mg, 0.35 mmol, 1.4 eq.), $Pd(OAc)_2$ (2.8 mg, 0.01 mmol, 0.05 eq.) and PCy_3 (7.0 mg, 0.02 mmol, 0.10 eq.) in toluene (5 mL) and H_2O (0.5 mL) gave the crude product. Purification by flash column chromatography on silica with 3:2 hexane-EtOAc as eluent gave arylated tropane **45** (69 mg, 78%) as an off-white solid, mp 144–146 °C; R_F (7:3 hexane-EtOAc) 0.42; IR (ATR) 3347 (NH), 3196 (NH), 2977, 2243, 1661 (C=O, Boc), 1611 (C=O, amide), 1390, 1159, 1102, 728 cm^{-1}; ^1H NMR (400 MHz, $CDCl_3$) δ 7.75–7.64 (m, 2H, Ar), 7.18–7.05 (m, 2H, Ar), 6.55–6.22 (m, 2H, NH_2), 4.32–4.09 (m, 2H, NCH), 2.38–2.20 (m, 1H, CH), 1.94–1.85 (m, 4H, CH), 1.69 (dd, *J* = 8.5, 6.0 Hz, 1H, C*H*Ar), 1.55–1.34 (m, 10H, CMe_3, CH), 1.17 (dd, *J* = 6.0, 5.0 Hz, 1H, CH), 1.10 (dd, *J* = 8.5, 5.0 Hz, 1H, CH), 0.98 (d, *J* = 13.5 Hz, 1H, CH), 0.63–0.50 (m, 1H, CH); ^{13}C NMR (100.6 MHz, $CDCl_3$) (rotamers) δ 169.8 (C=O, amide), 153.4 (C=O, Boc), 143.4 (*ipso*-Ar), 131.0 (*ipso*-Ar), 128.7 (Ar), 127.2 (Ar), 79.2 (O*C*Me_3), 55.2 (br, NCH), 54.4 (br, NCH), 53.9 (br, NCH), 52.5 (br, NCH), 42.8 (br, CH_2), 41.6 (br, CH_2), 37.0 (br, CH_2), 36.5 (br, CH_2), 29.7 (CH_2), 29.3 (CH_2), 28.5 (CMe_3), 28.0 (br, CH_2), 27.4 (br, CH_2), 24.4 (CHAr), 21.8 (C), 20.0 (br, $CHCH_2$); HRMS (ESI) *m/z* calcd for $C_{21}H_{28}N_2O_3$ $(M + Na)^+$ 379.1992, found 379.1986 (+1.6 ppm error).

Lab book reference: ARG-2-170

***tert*-Butyl (1*R**,3'*S**,5*S**)-3'-(1-(*tert*-butoxycarbonyl)-1*H*-indol-5-yl)-8-azaspiro[bicyclo[3.2.1]octane-3,1'-cyclopropane]-8-carboxylate 46**

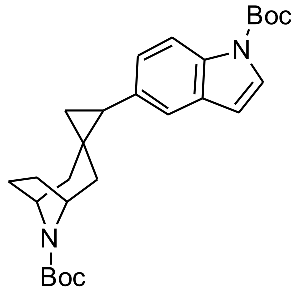

46

Using general procedure C, MIDA boronate **1f** (98 mg, 0.25 mmol, 1.0 eq.), 1 M $NaOH_{(aq)}$ (1 mL) and THF (1 mL) and then Cs_2CO_3 (244 mg, 0.75 mmol, 3.0 eq.), *N*-Boc-5-bromoindole (103 mg, 0.35 mmol, 1.4 eq.), $Pd(OAc)_2$ (2.8 mg, 0.01 mmol, 0.05 eq.) and PCy_3 (7.0 mg, 0.02 mmol, 0.10 eq.) in toluene (5 mL) and H_2O (0.5 mL) gave the crude product. Purification by flash column chromatography on silica with 98:2 hexane-EtOAc as eluent gave arylated tropane **46** (68 mg, 60%) as a colourless oil, R_F (95:5 hexane-EtOAc) 0.20; IR (ATR) 2977, 1732 (C=O), 1687 (C=O), 1367, 1325, 1158, 1023, 728 cm^{-1}; ^1H NMR (400 MHz, $CDCl_3$) δ 8.06–7.95 (m, 1H, Ar), 7.55 (d, *J* = 4.0 Hz, 1H, Ar), 7.30–7.28 (m, 1H, Ar), 7.11 (dd, *J* = 8.5, 2.0 Hz, 1H, Ar), 6.50 (d, *J* = 4.0 Hz, 1H, Ar), 4.31–4.26 (m, 1H, NCH), 4.20–4.13 (m, 1H, NCH), 2.37–2.30 (m, 1H, CH), 1.97–1.88 (m, 4H, CH), 1.79 (dd, *J* = 8.0, 6.0 Hz, 1H, C*H*Ar), 1.68–1.63 (m, 10H, CMe_3, CH), 1.44 (s, 9H, CMe_3), 1.18 (dd, *J* = 6.0, 5.0 Hz, 1H, CH), 1.09 (dd, *J* = 8.0, 5.0 Hz, 1H, CH), 1.01 (d, *J* = 13.5 Hz, 1H, CH), 0.57 (d, *J* = 13.5 Hz, 1H, CH); ^{13}C NMR (100.6 MHz, $CDCl_3$) (rotamers) δ 153.4 (C=O), 149.9 (C=O), 135.9 (*ipso*-Ar), 133.1 (*ipso*-Ar), 130.6 (*ipso*-Ar), 126.2 (Ar), 126.1 (Ar), 125.7 (Ar), 120.6 (Ar), 114.7 (Ar), 114.6 (Ar), 107.3 (Ar), 107.2 (Ar), 83.6 (O*C*Me_3), 79.1 (O*C*Me_3), 54.4 (NCH), 54.1 (NCH), 42.0 (CH_2), 37.2 (CH_2), 28.7 (C*Me*$_3$), 28.6 (C*Me*$_3$), 28.3 (C*Me*$_3$), 28.2 (C*Me*$_3$), 27.8 (br, CH_2), 24.1 (CHAr), 24.0 (CHAr), 21.5 (br, C), 18.9 (CH*C*H_2) (1 × CH_2 resonance not resolved); HRMS (ESI) *m/z* calcd for $C_{27}H_{36}N_2O_4$ $(M + Na)^+$ 475.2567, found 475.2577 (–2.0 ppm error).

Lab book reference: ARG-2-184

***tert*-Butyl (1*R**,6*R**,7*R**)-7-(pyrimidin-5-yl)-3-azabicyclo[4.1.0]heptane-3-carboxylate 47**

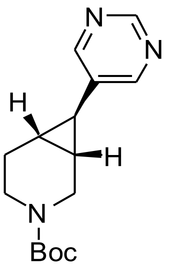

47

Using general procedure B, MIDA boronate **1g** (88 mg, 0.25 mmol, 1.0 eq.), Cs_2CO_3 (244 mg, 0.75 mmol, 3.0 eq.), 5-bromopyrimidine (56 mg, 0.35 mmol, 1.4 eq.), $Pd(OAc)_2$ (2.8 mg, 0.01 mmol, 0.05 eq.) and PCy_3 (7.0 mg, 0.02 mmol, 0.10 eq.) in toluene (5 mL) and H_2O (0.5 mL) gave the crude product. Purification by flash column chromatography on silica with 4:1 hexane-EtOAc as eluent gave arylated piperidine **47** (61 mg, 89%) as a white solid, mp 68–70 °C; R_F (7:3 hexane-EtOAc) 0.24; IR (ATR) 2975, 1684 (C=O), 1418, 1365, 1245, 1164, 727, 630 cm^{-1}; ^1H NMR (400 MHz, $CDCl_3$) δ 8.98 (s, 1H, Ar), 8.38 (s, 2H, Ar), 4.11–3.90 (m, 1H, NCH), 3.69–3.42 (m, 2H, NCH), 2.98 (ddd, J = 13.5, 9.5, 5.5 Hz, 1H, NCH), 2.05 (dddd, J = 14.0, 7.0, 5.5, 5.0 Hz, 1H, CH), 1.95–1.81 (m, 1H, CH), 1.59 (dd, J = 4.5, 4.5 Hz, 1H, C*H*Ar), 1.51–1.38 (m, 11H, CMe_3, CH); ^{13}C NMR (100.6 MHz, $CDCl_3$) δ 156.2 (Ar), 155.1 (C=O), 154.2 (Ar), 136.2 (*ipso*-Ar), 79.9 (O*C*Me_3), 41.8 (br, NCH_2), 39.8 (br, NCH_2), 28.5 (C*Me*$_3$), 22.5 (br, CH_2, CH), 22.0 (CHAr), 20.5 (CH); HRMS (ESI) *m/z* calcd for $C_{15}H_{21}N_3O_2$ $(M + H)^+$ 276.1707, found 276.1713 (–2.5 ppm error).

Lab book reference: ARG-2-160

tert-Butyl (1_R_*,6_R_*,7_R_*)-7-(pyrazin-2-yl)-3-azabicyclo[4.1.0]heptane-3-carboxylate 48

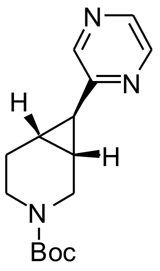

48

Using general procedure B, MIDA boronate **1g** (88 mg, 0.25 mmol, 1.0 eq.), Cs_2CO_3 (244 mg, 0.75 mmol, 3.0 eq.), 2-bromopyrazine (32 μL, 0.35 mmol, 1.4 eq.), $Pd(OAc)_2$ (2.8 mg, 0.01 mmol, 0.05 eq.) and PCy_3 (7.0 mg, 0.02 mmol, 0.10 eq.) in toluene (5 mL) and H_2O (0.5 mL) gave the crude product. Purification by flash column chromatography on silica with 1:1 hexane-EtOAc as eluent gave arylated piperidine **48** (45 mg, 65%) as a colourless oil, R_F (1:1 hexane-EtOAc) 0.22; IR (ATR) 2975, 2928, 1686 (C=O), 1416, 1364, 1244, 1165, 1131, 769 cm^{-1}; ^{1}H NMR (400 MHz, $CDCl_3$) δ 8.42 (d, J = 1.5 Hz, 1H, Ar), 8.32 (dd, J = 2.5, 1.5 Hz, 1H, Ar), 8.25 (d, J = 2.5 Hz, 1H, Ar), 4.03–3.87 (m, 1H, NCH), 3.65–3.39 (m, 1H, NCH), 3.52 (dd, J = 13.5, 4.0 Hz, 1H, NCH), 2.98 (ddd, J = 13.5, 9.5, 5.5 Hz, 1H, NCH), 2.08–1.96 (m, 1H, C*H*Ar), 1.92–1.81 (m, 1H, CH), 1.79 (dd, J = 4.5, 4.0 Hz, 1H, CH), 1.75–1.70 (m, 2H, CH), 1.45 (s, 9H, CMe_3); ^{13}C NMR (100.6 MHz, $CDCl_3$) (rotamers) δ 157.7 (*ipso*-Ar), 155.2 (C=O), 144.1 (Ar), 143.7 (Ar), 141.3 (Ar), 79.7 (O*C*Me_3), 42.2 (NCH_2), 41.5 (NCH_2), 40.9 (NCH_2), 39.7 (NCH_2), 28.5 (CMe_3), 26.3 (br, CH), 23.2 (CHAr), 22.8 (CH_2), 22.4 (CH_2), 21.7 (br, CH); HRMS (ESI) *m/z* calcd for $C_{15}H_{21}N_3O_2$ $(M + Na)^+$ 298.1526, found 298.1527 (–0.3 ppm error).

Lab book reference: ARG-2-146

***tert*-Butyl (1*R**,6*R**,7*R**)-7-(2-methoxypyridin-4-yl)-3-azabicyclo[4.1.0]heptane-3-carboxylate 49**

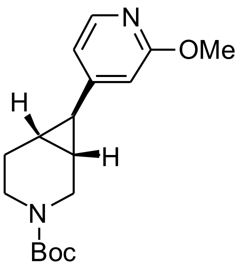

49

Using general procedure B, MIDA boronate **1g** (88 mg, 0.25 mmol, 1.0 eq.), Cs_2CO_3 (244 mg, 0.75 mmol, 3.0 eq.), 4-bromo-2-methoxypyridine (66 mg, 0.35 mmol, 1.4 eq.), $Pd(OAc)_2$ (2.8 mg, 0.01 mmol, 0.05 eq.) and PCy_3 (7.0 mg, 0.02 mmol, 0.10 eq.) in toluene (5 mL) and H_2O (0.5 mL) gave the crude product. Purification by flash column chromatography on silica with 4:1 hexane-EtOAc as eluent gave arylated piperidine **49** (67 mg, 88%) as a colourless oil, R_F (3:2 hexane-EtOAc) 0.38; IR (ATR) 2976, 1687 (C=O), 1611, 1417, 1390, 1245, 1162, 1129, 1045, 731 cm^{-1}; ^{1}H NMR (400 MHz, $CDCl_3$) δ 7.94 (d, J = 5.5 Hz, 1H, Ar), 6.45 (dd, J = 5.5, 1.5 Hz, 1H, Ar), 6.31 (d, J = 1.5 Hz, 1H, Ar), 3.92 (d, J = 14.0 Hz, 1H, NCH), 3.86 (s, 3H, OMe), 3.51 (dd, J = 14.0, 4.5 Hz, 1H, NCH), 3.58–3.38 (m, 1H, NCH), 3.01–2.89 (m, 1H, NCH), 2.03–1.93 (m, 1H, CHAr), 1.88–1.74 (br m, 1H, CH), 1.54 (dd, J = 4.5, 4.5 Hz, 1H, CH), 1.44 (s, 9H, CMe_3), 1.45–1.31 (m, 2H, CH); ^{13}C NMR (100.6 MHz, $CDCl_3$) (rotamers) δ 164.5 (*ipso*-Ar), 155.4 (*ipso*-Ar), 155.1 (C=O), 146.6 (Ar), 114.5 (Ar), 106.8 (Ar), 79.7 (O*C*Me_3), 53.4 (OMe), 42.4 (NCH_2), 41.6 (NCH_2), 40.4 (NCH_2), 39.9 (NCH_2), 28.5 (C*Me_3*), 26.9 (br, CH), 23.0 (CHAr), 22.5 (br, CH_2), 21.4 (CH); HRMS (ESI) *m/z* calcd for $C_{17}H_{24}N_2O_3$ $(M + H)^+$ 305.1860, found 305.1859 (+0.3 ppm error).

Lab book reference: ARG-2-147

H_2O (0.5 mL), degassed by sparging with Ar for 30 min was added to a solution of cataCXium A Pd G3 (9 mg, 0.01 mmol, 0.05 eq.), cyclopropyl BF_3K **S12** (76 mg, 0.25 mmol, 1.0 eq.), Cs_2CO_3 (244 mg, 0.75 mmol, 3.0 eq.) and 4-bromo-2-methoxypyridine (66 mg, 0.35 mmol, 1.4 eq.) in toluene (5.0 mL) at rt in a sealed tube under Ar. The resulting mixture was stirred and heated at 100 °C for 18 h under Ar. The solids were removed by filtration through Celite® and washed with EtOAc (5 mL) and water (5 mL). The two layers were separated and the aqueous layer was extracted with EtOAc (3 × 5 mL). The combined organic layers were washed with brine (10 mL), dried (Na_2SO_4) and evaporated under reduced pressure

to give the crude product. Purification by flash column chromatography on silica with 4:1 hexane-EtOAc as eluent gave arylated piperidine **49** (64 mg, 85%) as a colourless oil identical (by NMR spectroscopy) to that described above.

Lab book reference: ARG-3-019

***tert*-Butyl (1*R**,6*R**,7*R**)-7-(2-aminopyridin-4-yl)-3-azabicyclo[4.1.0]heptane-3-carboxylate 50**

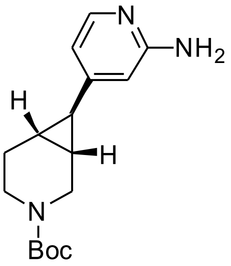

50

Using general procedure A, MIDA boronate **1g** (176 mg, 0.500 mmol, 1.0 eq.), Cs_2CO_3 (978 mg, 3.00 mmol, 6.0 eq.), PCy_3 (84 mg, 0.15 mmol, 0.3 eq.), $Pd(OAc)_2$, (17 mg, 0.076 mmol, 0.15 eq.) and 2-amino-4-bromopyridine (121 mg, 0.700 mmol, 1.4 eq.) in toluene (7 mL) and H_2O (0.6 mL) gave the crude product. Purification by flash column chromatography on silica with 99:1 to 97:3 to 90:10 CH_2Cl_2-methanol gave impure product. Further purification by flash column chromatography on silica with hexane to 99:1 EtOAc-Et_3N gave arylated piperidine **50** (31 mg, 21%) as a yellow oil, R_F (99:1 EtOAc-Et_3N) 0.12; IR (ATR) 3320 (NH), 3306 (NH), 1678 (C=O), 1616, 1366, 1131, 728 cm^{-1}; ^1H NMR (400 MHz, $CDCl_3$) δ 7.86 (br d, *J* = 5.5 Hz, 1H, Ar), 6.22 (dd, *J* = 5.5, 1.5 Hz, 1H, Ar), 6.13 (br s, 1H, Ar), 4.53-4.23 (brs, 2H, NH_2), 3.91 (br d, *J* = 13.5 Hz, 1H, NCH), 3.52 (dd, *J* = 13.5, 4.5 Hz, 1H, NCH), 3.49–3.37 (m, 1H, NCH), 3.02–2.90 (m, 1H, NCH), 2.03–1.95 (m, 1H, CH), 1.89–1.72 (m, 1H, CH), 1.49–1.35 (m, 3H, CH), 1.45 (s, 9H, CMe_3); ^{13}C NMR (100.6 MHz, $CDCl_3$) (rotamers) δ 158.5 (*ipso*-Ar), 155.1 (C=O), 154.3 (*ipso*-Ar), 147.9 (Ar), 111.3 (Ar), 105.2 (Ar), 79.7 (O*C*Me_3), 42.3 (NCH_2), 41.6 (NCH_2), 40.9 (NCH_2), 39.9 (NCH_2), 28.5 (C*Me_3*), 27.0 (*C*HAr), 22.8 (CH_2), 22.6 (CH), 21.0 (CH); MS (EI) *m/z* 290 (M + H)$^+$; HRMS (ESI) *m/z* calcd for $C_{15}H_{24}N_3O_2$ (M + H)$^+$ 290.1863, found 290.1864 (–0.4 ppm error).

Lab book reference HFK8-023

***tert*-Butyl (1*R**,6*R**,7*R**)-7-[2-(hydroxymethyl)pyridin-4-yl]-3-azabicyclo[4.1.0]heptane-3-carboxylate 51**

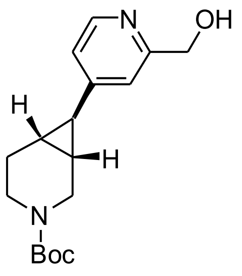

51

Using general procedure A, MIDA boronate **1g** (176 mg, 0.50 mmol, 1.0 eq.), Cs_2CO_3 (978 mg, 3.0 mmol, 6.0 eq.), PCy_3 (84 mg, 0.15 mmol, 0.3 eq.), $Pd(OAc)_2$, (17 mg, 0.076 mmol, 0.15 eq.) and 4-bromo-2-pyridinemethanol (132 mg, 0.700 mmol, 1.4 eq.) in toluene (7 mL) and H_2O (0.6 mL) gave the crude product. Purification by flash column chromatography on silica with 70:30 to 50:50 CH_2Cl_2-acetone as eluent gave impure product. Purification by flash column chromatography on silica with EtOAc to 80:20 to 70:30 to 60:40 CH_2Cl_2-acetone as eluent gave arylated piperidine **51** (15 mg, 10%) as a yellow oil, R_F (1:1 CH_2Cl_2-acetone) 0.18; IR (ATR) 2927 (OH), 1684 (C=O), 1605, 1420, 1365, 1247 cm^{-1}; ^1H NMR (400 MHz, $CDCl_3$) δ 8.35 (br s, 1H, Ar), 6.88 (br s, 1H, Ar), 6.81 (br s, 1H, Ar), 4.70 (br s, 2H, OCH), 3.98 (br d, *J* = 13.5 Hz, 1H, NCH), 3.54 (dd, *J* = 13.5, 4.5 Hz, 2H, NCH), 3.02–2.95 (m, 1H, NCH), 2.08–2.00 (m, 1H, CH), 1.92–1.7z9 (m, 1H, CH), 1.63 (dd, *J* = 4.5, 4.5 Hz, 1H, CHAr), 1.55–1.48 (m, 2H, CH), 1.47 (s, 9H, CMe_3); ^{13}C NMR (100.6 MHz, $CDCl_3$) (rotamers) δ 158.8 (*ipso*-Ar), 155.2 (C=O), 153.9 (*ipso*-Ar), 148.1 (Ar), 119.6 (Ar), 117.4 (Ar), 79.9 (O*C*Me_3), 64.2 ($HOCH_2$), 42.3 (NCH_2), 41.5 (NCH_2), 40.9 (NCH_2), 39.9 (NCH_2), 28.6 (C*Me₃*), 27.2 (CHAr), 23.6 (CH_2), 22.8 (CH), 22.0 (CH); MS (EI) *m/z* 327 $(M + Na)^+$; HRMS (ESI) *m/z* calcd for $C_{17}H_{24}N_2O_3$ $(M + Na)^+$ 327.1679, found 327.1682 (–1.0 ppm error).

Lab book reference HFK8-028

tert-Butyl (1_R_*,6_R_*,7_R_*)-7-(3-hydroxyphenyl)-3-azabicyclo[4.1.0]heptane-3-carboxylate 52

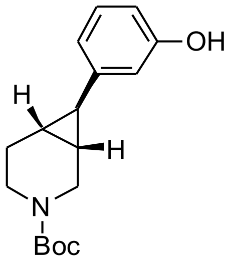

52

Using general procedure B, MIDA boronate **1g** (88 mg, 0.25 mmol, 1.0 eq.), Cs_2CO_3 (244 mg, 0.75 mmol, 3.0 eq.), 3-bromophenol (61 mg, 0.35 mmol, 1.4 eq.), $Pd(OAc)_2$ (2.8 mg, 0.01 mmol, 0.05 eq.) and PCy_3 (7.0 mg, 0.02 mmol, 0.10 eq.) in toluene (5 mL) and H_2O (0.5 mL) gave the crude product. Purification by flash column chromatography on silica with 4:1 hexane-EtOAc as eluent gave arylated piperidine **52** (55 mg, 76%) as a white solid, mp 112-114 °C; R_F (3:2 hexane-EtOAc) 0.35; IR (ATR) 3315 (OH), 2929, 1657 (C=O), 1430, 1366, 1248, 1159, 730 cm^{-1}; ^{1}H NMR (400 MHz, $CDCl_3$) δ 7.07 (dd, J = 8.0, 7.5 Hz, 1H, Ar), 6.82 (br s, 1H, OH), 6.64 (d, J = 8.0 Hz, 1H, Ar), 6.53 (d, J = 7.5 Hz, 1H, Ar), 6.50 (s, 1H, Ar), 3.90 (br d, J = 13.5 Hz, 1H, NCH), 3.67–3.36 (m, 2H, NCH), 3.16–2.83 (m, 1H, NCH), 2.05–1.93 (m, 1H, C*H*Ar), 1.87–1.74 (m, 1H, CH), 1.58–1.54 (m, 1H, CH), 1.48 (s, 9H, CMe_3), 1.40–1.27 (m, 2H, CH); ^{13}C NMR (100.6 MHz, $CDCl_3$) (rotamers) δ 156.6 (C=O), 155.6 (*ipso*-Ar), 144.7 (*ipso*-Ar), 129.5 (Ar), 117.4 (br, Ar), 112.7 (Ar), 112.4 (Ar), 80.2 (O*C*Me_3), 42.6 (NCH_2), 41.9 (NCH_2), 41.1 (NCH_2), 40.3 (NCH_2), 28.6 (C*Me₃*), 27.5 (br, CHAr), 23.0 (CH_2), 22.6 (CH_2), 21.7 (br, CH), 20.2 (br, CH); HRMS (ESI) *m/z* calcd for $C_{17}H_{23}NO_3$ (M + Na)$^+$ 312.1570, found 312.1574 (–1.2 ppm error).

Lab book reference: ARG-2-144

***tert*-Butyl (1*R**,6*R**,7*R**)-7-(2-(trifluoromethyl)phenyl)-3-azabicyclo[4.1.0]heptane-3-carboxylate 53**

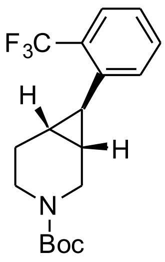

53

Using general procedure B, MIDA boronate **1g** (88 mg, 0.25 mmol, 1.0 eq.), Cs_2CO_3 (244 mg, 0.75 mmol, 3.0 eq.), 2-bromobenzotrifluoride (79 mg, 0.35 mmol, 1.4 eq.), $Pd(OAc)_2$ (2.8 mg, 0.01 mmol, 0.05 eq.) and PCy_3 (7.0 mg, 0.02 mmol, 0.10 eq.) in toluene (5 mL) and H_2O (0.5 mL) gave the crude product. Purification by flash column chromatography on silica with 9:1 hexane-Et_2O as eluent gave arylated piperidine **53** (66 mg, 78%) as a white solid, mp 98–100 °C; R_F (7:3 hexane-Et_2O) 0.48; IR (ATR) 2930, 1688 (C=O), 1419, 1313, 1259, 1113, 764 cm^{-1}; ^1H NMR (400 MHz, $CDCl_3$) (55:45 mixture of rotamers) δ 7.59 (d, J = 8.0 Hz, 1H, Ar), 7.41 (dd, J = 8.0, 7.5 Hz, 1H, Ar), 7.23 (dd, J = 8.0, 7.5 Hz, 1H, Ar), 7.03 (d, J = 8.0 Hz, 1H, Ar), 4.13–4.03 (m, 0.55H, NCH), 4.02–3.93 (m, 0.45H, NCH), 3.73–3.59 (m, 0.9H, NCH), 3.54–3.37 (m, 1.1H, NCH), 3.12–3.00 (m, 0.45H, NCH), 2.93–2.84 (m, 0.55H, NCH), 2.06–1.93 (m, 2H, CH, C*H*Ar), 1.93–1.82 (m, 1H, CH), 1.47 (s, 9H, CMe_3), 1.53–1.39 (m, 1H, CH), 1.35–1.20 (m, 1H, CH); ^{13}C NMR (100.6 MHz, $CDCl_3$) (rotamers and conformers) δ 155.1 (C=O), 140.9 (br, *ipso*-Ar), 132.1 (Ar), 131.8 (Ar), 129.5 (Ar), 129.2 (Ar), 126.3 (Ar), 126.0 (Ar), 125.9 (Ar), 125.8 (Ar), 125.4 (Ar), 124.8 (q, J =272.0 Hz, CF_3), 79.6 (O*C*Me_3), 42.3 (br, NCH_2), 28.8 (C*Me₃*), 28.5 (C*Me₃*), 28.3 (C*Me₃*), 28.1 (C*Me₃*), 24.1 (*C*HAr), 23.9 (*C*HAr), 22.4 (br, CH_2), 21.5 (br, CH or CH_2), 19.8 (br, CH or CH_2), 18.4 (br, CH or CH_2); HRMS (ESI) *m/z* calcd for $C_{18}H_{22}F_3NO_2$ $(M + H)^+$ 364.1485, found 364.1485 (+2.7 ppm error).

Lab book reference: ARG-2-137

***tert*-Butyl (1*R**,6*R**,7*R**)-7-(benzofuran-3-yl)-3-azabicyclo[4.1.0]heptane-3-carboxylate 54**

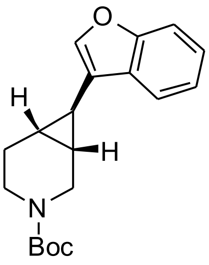

54

Using general procedure B, MIDA boronate **1g** (88 mg, 0.25 mmol, 1.0 eq.), Cs_2CO_3 (244 mg, 0.75 mmol, 3.0 eq.), 3-bromobenzofuran (69 mg, 0.35 mmol, 1.4 eq.), $Pd(OAc)_2$ (2.8 mg, 0.01 mmol, 0.05 eq.) and PCy_3 (7.0 mg, 0.02 mmol, 0.10 eq.) in toluene (5 mL) and H_2O (0.5 mL) gave the crude product. Purification by flash column chromatography on silica with 9:1 hexane-EtOAc as eluent gave arylated piperidine **54** (52 mg, 67%) as a colourless oil, R_F (4:1 hexane-EtOAc) 0.30; IR (ATR) 2975, 2928, 1685 (C=O), 1452, 1418, 1364, 1244, 1166, 743 cm^{-1}; ^{1}H NMR (400 MHz, $CDCl_3$) δ 7.58 (d, *J* = 7.5 Hz, 1H, Ar), 7.44 (d, *J* = 7.5 Hz, 1H, Ar), 7.31 (d, *J* = 1.0 Hz, 1H, Ar), 7.29–7.21 (m, 2H, Ar), 4.05 (d, *J* = 13.5 Hz, 1H, NCH), 3.61 (dd, *J* = 13.5, 4.5 Hz, 1H, NCH), 3.57–3.43 (m, 1H, NCH), 3.10–2.97 (m, 1H, NCH), 2.07 (dddd, *J* = 14.0, 7.0, 5.5, 5.0 Hz, 1H, CH), 2.01–1.89 (m, 1H, C*H*Ar), 1.65 (dd, *J* = 5.0, 5.0 Hz, 1H, CH), 1.49 (s, 9H, CMe_3), 1.47–1.31 (m, 2H, CH); ^{13}C NMR (100.6 MHz, $CDCl_3$) (rotamers) δ 155.4 (C=O), 155.3 (*ipso*-Ar), 140.3 (Ar), 128.3 (*ipso*-Ar), 124.4 (Ar), 122.4 (Ar), 122.1 (*ipso*-Ar), 119.7 (Ar), 111.5 (Ar), 79.6 (O*C*Me_3), 42.6 (NCH_2), 41.7 (NCH_2), 41.0 (NCH_2), 40.0 (NCH_2), 28.6 (C*Me*$_3$), 23.0 (br, CH_2), 22.4 (br, CH_2), 18.6 (CHAr), 17.0 (br, CH), 16.6 (br, CH); HRMS (ESI) *m/z* calcd for $C_{19}H_{23}NO_3$ (M + Na)$^+$ 336.1570, found 336.1564 (+1.9 ppm error).

Lab book reference: ARG-2-149

tert-Butyl (1R*,6R*,7R*)-7-(4-methoxyphenyl)-3-azabicyclo[4.1.0]heptane-3-carboxylate 55

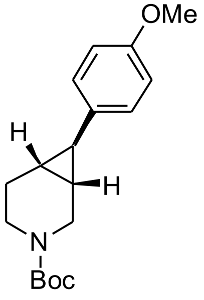

55

Using general procedure A, MIDA boronate **1g** (183 mg, 0.5 mmol, 1.0 eq.), Cs_2CO_3 (978 mg, 3.00 mmol, 6.0 eq.), PCy_3 (84 mg, 0.30 mmol, 0.3 eq.), $Pd(OAc)_2$, (17 mg, 0.076 mmol, 0.15 eq.) and 4-bromoanisole (88 µL, 0.70 mmol, 1.4 eq.) in toluene (11 mL) and H_2O (0.6 mL) gave the crude product. Purification by flash column chromatography on silica with 90:10 hexane-EtOAc as eluent gave arylated piperidine **55** (65 mg, 41%) as an amber oil, R_F (1:1 hexane-Et_2O) 0.43; IR (ATR) 1687 (C=O), 1515, 1243, 1166 cm^{-1}; ^1H NMR (400 MHz, $CDCl_3$) δ 6.94 (d, J = 8.5 Hz, 2H, Ar), 6.80 (d, J = 8.5 Hz, 2H, Ar), 3.92 (br d, J = 13.5 Hz, 1H, NCH), 3.77 (s, 3H, OMe), 3.64–3.36 (m, 2H, NCH), 3.09–2.94 (m, 1H, NCH), 2.06–1.98 (m, 1H, CH), 1.92–1.77 (m, 1H, CH), 1.61 (dd, J = 5.0, 5.0 Hz, 1H, CHAr), 1.48 (s, 9H, CMe_3), 1.38–1.22 (m, 2H, CH); ^{13}C NMR (100.6 MHz, $CDCl_3$) (rotamers) δ 157.7 (*ipso*-Ar), 155.3 (C=O), 134.9 (*ipso*-Ar), 126.5 (Ar), 113.9 (Ar), 79.5 (O*C*Me3), 55.4 (OMe), 42.6 (NCH_2), 41.8 (NCH_2), 41.0 (NCH_2), 40.1 (NCH_2), 28.6 (C*Me₃*), 26.7 (CHAr), 23.0 (CH_2), 22.6 (CH_2), 21.2 (CH), 19.7 (CH); MS (EI) *m/z* 326 (M + Na)$^+$; HRMS (ESI) *m/z* calcd for $C_{18}H_{25}NO_3$ (M + Na)$^+$ 326.1727, found 326.1713 (+4.2 ppm error).

Lab book reference HFK8-010

A mixture of BF_3K **S12** (152 mg, 0.5 mmol, 1.0 eq.), 4-bromoanisole (75 µL, 0.60 mmol, 1.2 eq.), $Pd(dppf)Cl_2$ (22 mg, 0.03 mmol, 0.06 eq.) and Cs_2CO_3 (375 mg, 1.15 mmol, 2.3 eq.) in THF (2.1 mL) and water (0.7 mL) was stirred at rt in a sealed tube and the suspension was degassed by sparging with Ar for 20 min. The resulting mixture was stirred and heated at 100 °C for 18 h. The mixture was allowed to cool to rt, water (5 mL) and CH_2Cl_2 (5 mL) were added and the two layers were separated. The aqueous layer was extracted with CH_2Cl_2 (3 × 5 mL). The combined organic layers were dried (Na_2SO_4) and evaporated under reduced pressure to give the crude product. Purification by flash column

chromatography on silica with EtOAc as eluent gave pyrimidine cyclopropane **55** (114 mg, 75%) as an amber oil.

Lab book reference HFK7-096

***tert*-Butyl (1*R**,6*R**,7*R**)-7-(4-acetamidophenyl)-3-azabicyclo[4.1.0]heptane-3-carboxylate 56**

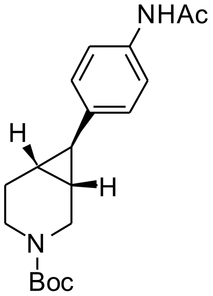

56

Using general procedure A, MIDA boronate **1g** (176 mg, 0.500 mmol, 1.0 eq.), Cs_2CO_3 (978 mg, 3.00 mmol, 6.0 eq.), PCy_3 (84 mg, 0.15 mmol, 0.3 eq.), $Pd(OAc)_2$, (17 mg, 0.076 mmol, 0.15 eq.) and 6-bromoacetanilide (150 mg, 0.700 mmol, 1.4 eq.) in toluene (7 mL) and H_2O (0.6 mL) gave the crude product. Purification by flash column chromatography on silica with 95:5 to 80:20 CH_2Cl_2-acetone as eluent gave arylated piperidine **56** (117 mg, 71%) as a cream solid, mp 132–134 °C; R_F (4:1 CH_2Cl_2-acetone) 0.47; IR (ATR) 3306 (NH), 1662 (C=O), 1516, 1655, 730 cm^{-1}; ^1H NMR (400 MHz, $CDCl_3$) δ 7.61 (s, 1H, NH), 7.37 (d, J = 8.5 Hz, 2H, Ar), 6.93 (d, J = 8.5 Hz, 2H, Ar), 3.91 (br d, J = 13.5 Hz, 1H, NCH), 3.56 (dd, J = 13.5, 4.5 Hz, 1H, NCH), 3.52-3.38 (m, 1H, NCH), 3.05–2.95 (m, 1H, NCH), 2.14 (s, 3H, C(O)Me), 2.05–1.97 (m, 1H, CH), 1.89–1.74 (m, 1H, CH), 1.60 (dd, J = 4.5, 4.5 Hz, 1H, C*H*Ar), 1.47 (s, 9H, CMe_3), 1.37–1.27 (m, 2H, CH); ^{13}C NMR (100.6 MHz, $CDCl_3$) (rotamers) δ 168.5 (C=O, C(O)Me), 155.3 (C=O, Boc), 138.9 (*ipso*-Ar), 135.7 (*ipso*-Ar), 126.0 (Ar), 120.2 (Ar), 79.7 (O*C*Me_3), 42.6 (NCH_2), 41.8 (NCH_2), 41.1 (NCH_2), 40.1 (NCH_2), 28.6 (C*Me₃*), 27.0 (CHAr), 24.6 (C(O)*Me*), 23.1 (CH_2), 22.6 (CH_2), 21.6 (CH), 20.1 (CH); MS (EI) *m/z* 353 (M + Na)$^+$; HRMS (ESI) *m/z* calcd for $C_{19}H_{26}N_2O_3$ (M + Na)$^+$ 353.1836, found 353.1831 (+1.2 ppm error).

Lab book reference HFK8-020

***tert*-Butyl (1*R**,6*R**,7*R**)-7-(4-carbamoylphenyl)-3-azabicyclo[4.1.0]heptane-3-carboxylate 57**

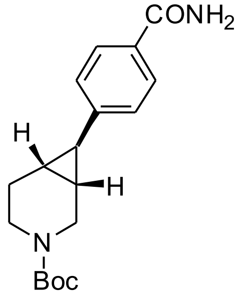

57

Using general procedure A, MIDA boronate **1g** (176 mg, 0.500 mmol, 1.0 eq.), Cs_2CO_3 (978 mg, 3.00 mmol, 6.0 eq.), PCy_3 (84 mg, 0.15 mmol, 0.3 eq.), $Pd(OAc)_2$, (17 mg, 0.076 mmol, 0.15 eq.) and 4-bromobenzamide (140 mg, 0.700 mmol, 1.4 eq.) in toluene (7 mL) and H_2O (0.6 mL) gave the crude product. Purification by flash column chromatography on silica with 90:10 to 75:15 to 80:20 to 60:40 CH_2Cl_2-acetone as eluent gave arylated piperidine **57** (126 mg, 80%) as a yellow solid, mp 166–168 °C; R_F (3:2 CH_2Cl_2-acetone) 0.38; IR (ATR) 3191 (NH), 1664 (C=O), 1611, 1390, 1168, 730 cm^{-1}; ^1H NMR (400 MHz, $CDCl_3$) δ 7.70 (d, *J* = 8.0 Hz, 2H, Ar), 7.02 (d, *J* = 8.0 Hz, 2H, Ar), 6.40–6.19 (m, 2H, NH_2), 3.94 (br d, *J* = 13.5 Hz, 1H, NCH), 3.55 (dd, *J* = 13.5, 4.5 Hz, 1H, NCH), 3.52–3.41 (m, 1H, NCH), 3.05–2.94 (m, 1H, NCH), 2.06–1.98 (m, 1H, CH), 1.91–1.77 (m, 1H, CH), 1.67 (dd, *J* = 4.5, 4.5 Hz, 1H, CHAr), 1.48–1.37 (m, 2H, CH), 1.46 (s, 9H, CMe_3); ^{13}C NMR (100.6 MHz, $CDCl_3$) (rotamers) δ 169.6 (C=O, $CONH_2$), 155.2 (C=O, Boc), 147.7 (*ipso*-Ar), 130.4 (*ipso*-Ar), 127.6 (Ar), 125.4 (Ar), 79.7 (O*C*Me_3), 42.4 (NCH_2), 41.7 (NCH_2), 41.0 (NCH_2), 40.0 (NCH_2), 28.6 (C*Me_3*), 27.6 (CH), 22.9 (CH), 21.3 (CH) (one CH_2 resonance not resolved); MS (EI) *m/z* 339 $(M + Na)^+$; HRMS (ESI) *m/z* calcd for $C_{18}H_{24}N_2O_3$ $(M + Na)^+$ 339.1679, found 339.1683 (–0.4 ppm error).

Lab book reference HFK8-032

***tert*-Butyl (1*R**,6*R**,7*R**)-7-(4-methanesulfonylphenyl)-3-azabicyclo[4.1.0]heptane-3-carboxylate 58**

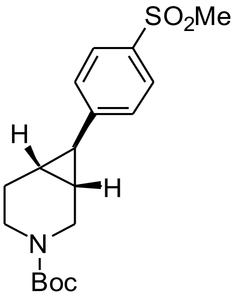

58

Using general procedure A, MIDA boronate **1g** (176 mg, 0.500 mmol, 1.0 eq.), Cs_2CO_3 (978 mg, 3.00 mmol, 6.0 eq.), PCy_3 (84 mg, 0.15 mmol, 0.3 eq.), $Pd(OAc)_2$, (17 mg, 0.076 mmol, 0.15 eq.) and 4-bromophenyl methyl sulfone (165 mg, 0.700 mmol, 1.4 eq.) in toluene (7 mL) and H_2O (0.6 mL) gave the crude product. Purification by flash column chromatography on silica with 60:40 hexane-EtOAc gave arylated piperidine **58** (151 mg, 86%) as a yellow oil, R_F (7:3 hexane-EtOAc) 0.21; IR (ATR) 1680 (C=O), 1304, 1147, 726 cm^{-1}; ^{1}H NMR (400 MHz, $CDCl_3$) δ 7.74 (d, *J* = 8.0 Hz, 2H, Ar), 7.11 (d, *J* = 8.0 Hz, 2H, Ar), 3.94 (br d, *J* = 14.0 Hz, 1H, NCH), 3.52 (dd, *J* = 14.0, 4.5 Hz, 1H, NCH), 2.98–2.92 (m, 1H, NCH), 2.97 (s, 3H, SO_2Me), 2.04–1.96 (m, 1H, CH), 1.90–1.75 (m, 1H, CH), 1.68 (dd, *J* = 4.5, 4.5 Hz, CHAr), 1.50–1.45 (m, 1H, CH), 1.44–1.38 (m, 1H, CH), 1.43 (s, 9H, CMe_3); ^{13}C NMR (100.6 MHz, $CDCl_3$) (rotamers) δ 155.0 (C=O), 150.0 (*ipso*-Ar), 137.2 (*ipso*-Ar), 127.4 (Ar), 126.0 (Ar), 79.7 (O*C*Me$_3$), 44.6 (SO2Me), 42.2 (NCH_2), 41.5 (NCH_2), 40.8 (NCH_2), 39.8 (NCH_2), 28.5 (C*Me$_3$*), 27.5 (CHAr), 23.5 (CH), 22.6 (CH_2), 21.9 (CH); MS (EI) *m/z* 374 (M + Na)$^+$; HRMS (ESI) *m/z* calcd for $C_{18}H_{25}NO_4S$ (M + Na)$^+$ 374.1397, found 374.1397 (–0.3 ppm error).

Lab book reference HFK8-025

tert-Butyl (1_R_*,6_R_*,7_R_*)-7-(4-sulfamoylphenyl)-3-azabicyclo[4.1.0]heptane-3-carboxylate 59

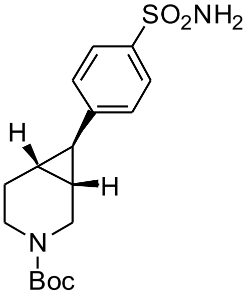

59

Using general procedure A, MIDA boronate **1g** (176 mg, 0.500 mmol, 1.0 eq.), Cs_2CO_3 (978 mg, 3.00 mmol, 6.0 eq.), PCy_3 (84 mg, 0.15 mmol, 0.3 eq.), $Pd(OAc)_2$, (17 mg, 0.076 mmol, 0.15 eq.) and 4-bromobenzenesulfonamide (165 mg, 0.700 mmol, 1.4 eq.) in toluene (7 mL) and H_2O (0.6 mL) gave the crude product. Purification by flash column chromatography on silica with 95:5 to 90:10 CH_2Cl_2-acetone as eluent gave arylated piperidine **59** (47 mg, 54%) as a yellow oil, R_F (9:1 CH_2Cl_2-acetone) 0.44; IR (ATR) 3254 (NH), 1669 (C=O), 1426, 1161, 731 cm^{-1}; ^{1}H NMR (400 MHz, $CDCl_3$) δ 7.78 (d, J = 8.5 Hz, 2H, Ar), 7.08 (d, J = 8.5 Hz, 2H, Ar), 5.17 (s, 2H, NH_2), 3.95 (br d, J = 13.5 Hz, 1H, NCH), 3.55 (dd, J = 13.5, 4.5 Hz, 1H, NCH), 3.52–3.44 (m, 1H, NCH), 3.03–2.95 (m, 1H, NCH), 2.07–1.99 (m, 1H, CH), 1.94–1.78 (m, 1H, CH), 1.69 (dd, J = 4.5, 4.5 Hz, 1H, CHAr), 1.50–1.38 (m, 2H, CH), i1.46 (s, 9H, CMe_3); ^{13}C NMR (100.6 MHz, $CDCl_3$) (rotamers) δ 155.2 (C=O), 148.9 (*ipso*-Ar), 139.0 (*ipso*-Ar), 126.6 (Ar), 125.9 (Ar), 79.9 (O*C*Me_3), 42.3 (NCH_2), 41.7 (NCH_2), 41.0 (NCH_2), 39.9 (NCH_2), 28.6 (C*Me*$_3$), 27.5 (CHAr), 23.3 (CH), 22.8 (CH_2), 21.7 (CH); MS (EI) *m/z* 375 (M + Na)$^+$; HRMS (ESI) *m/z* calcd for $C_{17}H_{24}N_2O_4S$ (M + Na)$^+$ 375.1349, found 375.1352 (–0.8 ppm error).

Lab book reference HFK8-022

***tert*-Butyl (1*R**,6*R**,7*R**)-7-(4-hydroxy-3-methoxyphenyl)-3-azabicyclo[4.1.0]heptane-3-carboxylate 60**

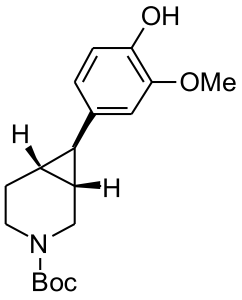

60

Using general procedure A, MIDA boronate **1g** (176 mg, 0.500 mmol, 1.0 eq.), Cs_2CO_3 (978 mg, 3.00 mmol, 6.0 eq.), PCy_3 (84 mg, 0.15 mmol, 0.3 eq.), $Pd(OAc)_2$, (17 mg, 0.076 mmol, 0.15 eq.) and 4-bromo-2-methoxyphenol (142 mg, 0.700 mmol, 1.4 eq.) in toluene (7 mL) and H_2O (0.6 mL) gave the crude product. Purification by flash column chromatography on silica with 95:5 to 90:10 to 80:20 hexane-EtOAc gave arylated piperidine **60** (83 mg, 52%) as a colourless oil, R_F (7:3 hexane-EtOAc) 0.17; IR (ATR) 3306 (OH), 1674 (C=O), 1516, 1162, 728 cm^{-1}; ^1H NMR (400 MHz, $CDCl_3$) δ 6.80 (d, J = 8.0 Hz, 1H, Ar), 6.57–6.54 (m, 1H, Ar), 6.49 (dd, J = 8.0, 2.0 Hz, 1H, Ar), 5.65 (s, 1H, OH), 3.92 (br d, J = 13.5 Hz, 1H, NCH), 3.86 (s, 3H, OMe), 3.58 (dd, J = 13.5, 5.0 Hz, 1H, NCH), 3.52–3.40 (m, 1H, NCH), 3.01 (ddd, J = 13.5, 9.0, 5.5 Hz, 1H, NCH), 2.06–1.98 (m, 1H, CH), 1.91–1.77 (m, 1H, CH), 1.60 (dd, J = 5.0, 5.0 Hz, 1H, CHAr), 1.47 (s, 9H, CMe_3), 1.39–1.20 (m, 2H, CH); ^{13}C NMR (100.6 MHz, $CDCl_3$) (rotamers) δ 155.3 (C=O), 146.6 (*ipso*-Ar), 143.7 (*ipso*-Ar), 134.7 (*ipso*-Ar), 117.8 (Ar), 114.4 (Ar), 108.9 (Ar), 79.6 (O*C*Me_3), 55.9 (OMe), 42.5 (NCH_2), 42.0 (NCH_2), 41.1 (NCH_2), 40.2 (NCH_2), 28.6 (C*Me*$_3$), 27.2 (CHAr), 22.8 (CH_2), 21.2 (CH), 19.6 (CH); MS (EI) *m/z* 342 $(M + Na)^+$; HRMS (ESI) *m/z* calcd for $C_{18}H_{25}NO_4$ $(M + Na)^+$ 342.1676, found 342.1676 (–0.1 ppm error).

Lab book reference HFK8-024

***tert*-Butyl (1*R**,6*R**,7*R**)-7-(3-cyano-4-methoxyphenyl)-3-azabicyclo[4.1.0]heptane-3-carboxylate 61**

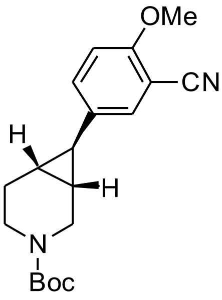

61

Using general procedure A, MIDA boronate **1g** (176 mg, 0.500 mmol, 1.0 eq.), Cs_2CO_3 (978 mg, 3.00 mmol, 6.0 eq.), PCy_3 (84 mg, 0.15 mmol, 0.3 eq.), $Pd(OAc)_2$ (17 mg, 0.076 mmol, 0.15 eq.) and 4-bromo-2-methoxybenzonitrile (149 mg, 0.700 mmol, 1.4 eq.) in toluene (7 mL) and H_2O (0.6 mL) gave the crude product. Purification by flash column chromatography on silica with 80:20 to 70:30 hexane-EtOAc gave arylated piperidine **61** (148 mg, 90%) as a yellow oil, R_F (7:3 hexane-EtOAc) 0.25; IR (ATR) 2975, 2226 (C≡N), 1683 (C=O), 1504, 1246, 1165 cm^{-1}; ^1H NMR (400 MHz, $CDCl_3$) δ 7.18 (dd, *J* = 8.5, 2.5 Hz, 1H, Ar), 7.15 (d, *J* = 2.5 Hz, 1H, Ar), 6.84 (d, *J* = 8.5 Hz, 1H, Ar), 3.98–3.90 (m, 1H, NCH), 3.87 (s, 3H, OMe), 3.53 (dd, *J* = 13.5, 4.5 Hz, 1H, NCH), 3.50–3.40 (m, 1H, NCH), 2.97 (ddd, *J* = 13.5, 9.5, 5.5 Hz, 1H, NCH), 2.05–1.97 (m, 1H, CH), 1.89–1.74 (m, 1H, CH), 1.58 (dd, *J* = 4.5, 4.5 Hz, 1H, CHAr), 1.45 (s, 9H, CMe_3), 1.34–1.23 (m, 2H, CH); ^{13}C NMR (100.6 MHz, $CDCl_3$) (rotamers) δ 159.4 (*ipso*-Ar), 155.2 (C=O), 135.6 (*ipso*-Ar), 131.9 (Ar), 130.4 (Ar), 116.7 (*ipso*-Ar), 111.4 (Ar), 101.6 (CN), 79.7 (O*C*Me_3), 56.2 (OMe), 42.3 (NCH_2), 41.5 (NCH_2), 41.0 (NCH_2), 39.9 (NCH_2), 28.5 (C*Me₃*), 26.1 (CH), 22.6 (CH_2), 21.5 (CH), 19.9 (CH); MS (EI) *m/z* 351 $(M + Na)^+$; HRMS (ESI) *m/z* calcd for $C_{19}H_{24}N_2O_3$ $(M + Na)^+$ 351.1679, found 351.1686 (–1.8 ppm error).

Lab book reference HFK8-029

***tert*-Butyl (1*R**,6*R**,7*R**)-7-(2-oxo-2,3-dihydro-1H-indol-6-yl)-3-azabicyclo[4.1.0]heptane-3-carboxylate 62**

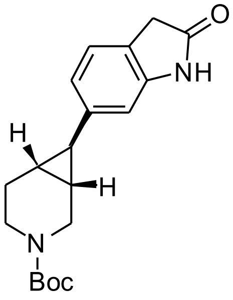

62

Using general procedure A, MIDA boronate **1g** (176 mg, 0.500 mmol, 1.0 eq.), Cs_2CO_3 (978 mg, 3.00 mmol, 6.0 eq.), PCy_3 (84 mg, 0.15 mmol, 0.3 eq.), $Pd(OAc)_2$ (17 mg, 0.076 mmol, 0.15 eq.) and 6-bromooxindole (148 mg, 0.700 mmol, 1.4 eq.) in toluene (7 mL) and H_2O (0.6 mL) gave the crude product. Purification by flash column chromatography on silica with 98:2 to 95:5 to 80:20 CH_2Cl_2-acetone as eluent gave the impure product. Further purification by flash column chromatography on silica with 60:40 hexane-EtOAc as eluent gave arylated piperidine **62** (28 mg, 17%) as a yellow oil, R_F (7:3 hexane-EtOAc) 0.27; IR (ATR) 3240 (NH), 1688 (C=O), 1681 (C=O), 1246, 1167, 730 cm^{-1}; ^1H NMR (400 MHz, $CDCl_3$) δ 9.03 (s, 1H, NH), 7.07 (br d, J = 7.5 Hz, 1H, Ar), 6.68 (dd, J = 7.5, 1.5 Hz, 1H, Ar), 6.53 (br d, J = 1.5 Hz, 1H, Ar), 3.93 (br d, J = 13.5 Hz, 1H, NCH), 3.59–3.52 (m, 2H, NCH), 3.48 (s, 2H, C(O)CH), 3.06–2.94 (m, 1H, NCH), 2.05–1.97 (m, 1H, CH), 1.94–1.77 (m, 1H, CH), 1.62 (dd, J = 4.5, 4.5 Hz, 1H, CHAr), 1.47 (s, 9H, CMe_3), 1.41–1.28 (m, 2H, CH); ^{13}C NMR (100.6 MHz, $CDCl_3$) (rotamers) δ 178.5 (C=O, lactam), 155.3 (C=O, Boc), 143.4 (*ipso*-Ar), 143.0 (*ipso*-Ar), 124.4 (Ar), 122.4 (*ipso*-Ar), 119.8 (Ar), 107.0 (Ar), 79.7 (O*C*Me_3), 42.5 (NCH_2), 41.7 (NCH_2), 41.1 (NCH_2), 40.0 (NCH_2), 36.2 ($CH_2C(O)$), 28.6 (C*Me_3*), 27.6 (CHAr), 23.0 (CH), 22.6 (CH), 22.0 (CH_2), 20.4 (CH_2); MS (EI) *m/z* 351 $(M + Na)^+$; HRMS (ESI) *m/z* calcd for $C_{19}H_{24}N_2O_3$ $(M + Na)^+$ 351.1679, found 351.1679 (+0.1 ppm error).

Lab book reference HFK8-019

***tert*-Butyl (1*R**,5*S**,6*R**)-6-(pyrimidin-5-yl)-3-azabicyclo[3.1.0]hexane-3-carboxylate 63**

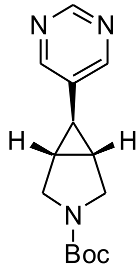

63

Using general procedure A, BMIDA building block **1h** (100 mg, 0.3 mmol, 1.0 eq.), 5-bromopyrimidine (66 mg, 0.41 mmol, 1.4 eq.), PCy_3 (25 mg, 0.09 mmol, 0.3 eq.), Cs_2CO_3 (579 mg, 1.8 mmol, 6 eq.) and $Pd(OAc)_2$ (10 mg, 0.04 mmol, 0.15 eq.) in toluene (3.1 mL) and water (0.2 mL) gave crude the product. Purification by flash column chromatography on silica with EtOAc as eluent gave cyclopropyl pyrimidine **63** (64 mg, 83%) as a colourless oil, R_F (EtOAc) 0.20; IR (ATR) 2975, 2931, 2872, 1690 (C=O), 1555, 1403, 1387, 1169, 1113, 1051, 727, 630, 552 cm^{-1}; ^1H NMR (400 MHz, $CDCl_3$) (50:50 mixture of rotamers) δ 9.00 (s, 1H, Ar), 8.42 (s, 2H, Ar), 3.78 (d, J = 11.0 Hz, 1H, NCH), 3.70 (d, J = 11.0 Hz, 1H, NCH), 3.52–3.43 (m, 2H, NCH), 1.92–1.83 (m, 2H, CH), 1.68 (t, J = 3.5 Hz, 1H, CH), 1.43 (s, 9H, CMe_3); ^{13}C NMR (100.6 MHz, $CDCl_3$) (rotamers) δ 156.5 (Ar), 154.9 (C=O), 154.4 (Ar), 134.7 (*ipso*-Ar), 79.9 (O*C*Me_3), 48.5 (NCH_2), 48.3 (NCH_2), 28.5 (C*Me*$_3$), 27.2 (CH), 26.5 (CH), 22.1 (*C*HAr); MS (ESI) *m/z* 284 (M + Na)$^+$; HRMS (ESI) *m/z* calcd for $C_{14}H_{19}N_3O_2$ (M + Na)$^+$ 284.1369, found 284.1375 (+1.9 ppm error).

Lab Book Reference: SY-1-51

Using general procedure B, BMIDA building block **1h** (169 mg, 0.50 mmol, 1.0 eq.), 5-bromopyrimidine (111 mg, 0.70 mmol, 1.4 eq.), PCy_3 (14 mg, 50 μmol, 0.10 eq.), Cs_2CO_3 (490 mg, 1.50 mmol, 3.0 eq.) and $Pd(OAc)_2$ (6 mg, 25 μmol, 0.05 eq.) in toluene (5.0 mL) and water (0.5 mL) gave the crude product. Purification by flash column chromatography on silica with 90:10 EtOAc-hexane as eluent gave cyclopropyl pyrimidine **63** (71 mg, 54%) as a colourless oil.

Lab Book Reference: SY-1-110

***tert*-Butyl (1*R**,5*S**,6*R**)-6-(2-cyanophenyl)-3-azabicyclo[3.1.0]hexane-3-carboxylate 64**

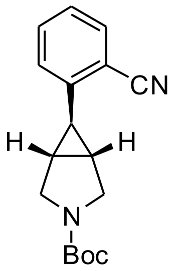

64

Using general procedure B, BMIDA building block **1h** (85 mg, 0.25 mmol, 1.0 eq.), 2-bromobenzonitrile (64 mg, 0.35 mmol, 1.4 eq.), PCy_3 (7 mg, 25 μmol, 0.10 eq.), Cs_2CO_3 (244 mg, 0.75 mmol, 3.0 eq.) and $Pd(OAc)_2$ (10 mg, 13 μmol, 0.05 eq.) in toluene (5.0 mL) and water (0.5 mL) gave the crude product. Purification by flash column chromatography on silica with EtOAc as eluent gave cyclopropyl benzonitrile **64** (28 mg, 39%) as a colourless oil, R_F (8:2 hexane-EtOAc) 0.17; IR (ATR) 2975, 2931, 2873, 2224 (C≡N), 1691 (C=O), 1418, 1388, 1366, 1171, 1115, 860, 762, 505 cm^{-1}; ^{1}H NMR (400 MHz, $CDCl_3$) (50:50 mixture of rotamers) δ 7.59 (dd, *J* = 7.5, 1.5 Hz, 1H, Ar), 7.48 (ddd, *J* = 7.5, 7.5, 1.5 Hz, 1H, Ar), 7.26 (ddd, *J* = 7.5, 7.5, 1.0 Hz, 1H, Ar), 6.98 (dd, *J* = 7.5, 1.0 Hz, 1H, Ar), 3.81 (d, *J* = 11.0 Hz, 1H, NCH), 3.75 (d, *J* = 11.0 Hz, 1H, NCH), 3.53 (dd, *J* = 11.0, 4.0 Hz, 1H, NCH), 3.49 (dd, *J* = 11.0, 4.0 Hz, 1H, NCH), 2.09 (t, *J* = 4.0 Hz, 1H, CH), 1.98–1.92 (m, 1H, CH), 1.90–1.83 (m, 1H, CH), 1.46 (s, 9H, CMe_3); ^{13}C NMR (100.6 MHz, $CDCl_3$) (rotamers) δ 155.0 (C=O), 145.1 (*ipso*-Ar), 133.0 (Ar), 132.9 (Ar), 126.4 (Ar), 125.4 (Ar), 118.3 (C≡N), 112.8 (*ipso*-Ar), 79.8 (O*C*Me_3), 48.8 (NCH_2), 48.5 (NCH_2), 28.6 (C*Me*$_3$), 28.0 (CH), 26.4 (CH), 25.6 (*C*HAr); MS (ESI) *m/z* 307 (M + Na)$^+$; HRMS (ESI) *m/z* calcd for $C_{17}H_{20}N_2O_2$ (M + Na)$^+$ 307.1417, found 307.1411 (+2.0 ppm error).

Lab Book Reference: SY-1-119

tert-Butyl (1_R_*,5_S_*,6_R_*)-6-(thiophen-3-yl)-3-azabicyclo[3.1.0]hexane-3-carboxylate 65

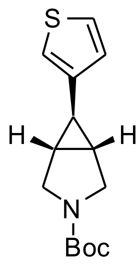

65

Using general procedure B, BMIDA building block **1h** (85 mg, 0.25 mmol, 1.0 eq.), 3-bromothiophene (33 μL, 0.35 mmol, 1.4 eq.), PCy_3 (7 mg, 25 μmol, 0.10 eq.), Cs_2CO_3 (244 mg, 0.75 mmol, 3.0 eq.) and $Pd(OAc)_2$ (3 mg, 13 μmol, 0.05 eq.) in toluene (5.0 mL) and water (0.5 mL) gave the crude product. Purification by flash column chromatography on silica with 90:10 hexane-EtOAc as eluent gave cyclopropyl thiophene **65** (39 mg, 59%) as a pale yellow oil, R_F (9:1 hexane-EtOAc) 0.22; IR (ATR) 2974, 2929, 2868, 1690 (C=O), 1392, 1365, 1169, 1111, 861, 767, 631, 546 cm^{-1}; ^{1}H NMR (400 MHz, $CDCl_3$) (50:50 mixture of rotamers) δ 7.23 (dd, J = 5.0, 3.0 Hz, 1H, Ar), 6.85–6.83 (m, 1H, Ar), 6.82–6.79 (m, 1H, Ar), 3.74 (d, J = 11.0 Hz, 1H, NCH), 3.65 (d, J = 11.0 Hz, 1H, NCH), 3.49–3.39 (m, 2H, NCH), 1.79–1.76 (m, 1H, CH), 1.76–1.72 (m, 2H, CH), 1.46 (s, 9H, CMe_3); ^{13}C NMR (100.6 MHz, $CDCl_3$) δ 155.1 (C=O), 142.0 (*ipso*-Ar), 126.1 (Ar), 125.8 (Ar), 118.1 (Ar), 79.6 (O*C*Me_3), 48.7 (NCH_2), 48.4 (NCH_2), 28.6 (C*Me*$_3$), 26.8 (CH), 26.1 (CH), 23.1 (CH); MS (ESI) *m/z* 288 $(M + Na)^+$; HRMS (ESI) *m/z* calcd for $C_{14}H_{19}NO_2S$ $(M + Na)^+$ 288.1029, found 288.1035 (–2.1 ppm error).

Lab Book Reference: SY-1-112

***tert*-Butyl (1*R**,5*S**,6*R**)-6-(3,5-dimethoxyphenyl)-3-azabicyclo[3.1.0]hexane-3-carboxylate 66**

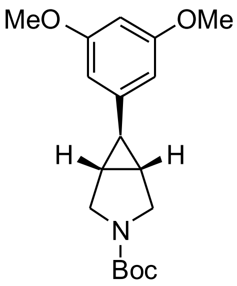

66

Using general procedure B, BMIDA building block **1h** (85 mg, 0.25 mmol, 1.0 eq.), 1-bromo-3,5-dimethoxybenzene (76 mg, 0.35 mmol, 1.4 eq.), PCy_3 (7 mg, 25 μmol, 0.10 eq.), Cs_2CO_3 (244 mg, 0.75 mmol, 3.0 eq.) and $Pd(OAc)_2$ (3 mg, 13 μmol, 0.05 eq.) in toluene (5.0 mL) and water (0.5 mL) gave the crude product. Purification by flash column chromatography on silica with 90:10 hexane-Et_2O as eluent gave cyclopropyl dimethoxybenzene **66** (45 mg, 56%) as a white solid, mp 76–78 °C; R_F (8:2 hexane-EtOAc) 0.29; IR (ATR) 2972, 2931, 2869, 1692 (C=O), 1594, 1459, 1407, 1389, 1204, 1151, 1113, 1061, 867, 825, 772, 692, 544 cm^{-1}; ^1H NMR (400 MHz, $CDCl_3$) (50:50 mixture of rotamers) δ 6.27 (t, *J* = 2.5 Hz, 1H, Ar), 6.18 (d, *J* = 2.5 Hz, 2H, Ar), 3.76 (s, 6H, OMe), 3.75–3.71 (m, 1H, NCH), 3.66 (d, *J* = 11.0 Hz, 1H, NCH), 3.50–3.41 (m, 2H, NCH), 1.80–1.76 (m, 2H, CH), 1.64 (t, *J* = 3.5 Hz, 1H, CH), 1.46 (s, 9H, CMe_3); ^{13}C NMR (100.6 MHz, $CDCl_3$) δ 160.9 (*ipso*-Ar), 155.1 (C=O), 143.9 (*ipso*-Ar), 103.9 (Ar), 97.7 (Ar), 79.6 ($OCMe_3$), 55.4 (OMe), 48.9 (NCH_2), 48.6 (NCH_2), 28.6 (CMe_3), 27.5 (*C*HAr), 27.4, (CH) 26.8 (CH); MS (ESI) *m/z* 342 $(M + Na)^+$; HRMS (ESI) *m/z* calcd for $C_{18}H_{25}NO_4$ $(M + Na)^+$ 342.1676, found 342.1677 (–0.4 ppm error).

Lab Book Reference: SY-1-118

***tert*-Butyl (1*R**,5*S**,6*R**)-6-[1-(4-methylbenzenesulfonyl)-1H-1,3-benzodiazol-4-yl]-3-azabicyclo[3.1.0]hexane-3-carboxylate 67**

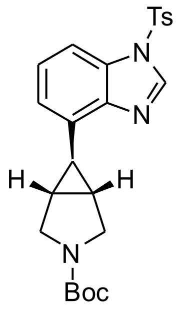

67

Using general procedure B, BMIDA building block **1h** (85 mg, 0.25 mmol, 1.0 eq.), *N*-Ts, 4-bromo-1*H*-benzo[d]imidazole (123 mg, 0.35 mmol, 1.4 eq.), PCy_3 (7 mg, 25 μmol, 0.10 eq.), Cs_2CO_3 (244 mg, 0.75 mmol, 3.0 eq.) and $Pd(OAc)_2$ (10 mg, 13 μmol, 0.05 eq.) in toluene (5.0 mL) and water (0.5 mL) gave the crude product. Purification by flash column chromatography on silica with 60:40 hexane-EtOAc as eluent gave cyclopropyl benzimidazole **67** (80 mg, 71%) as an off-white solid, mp 175–176 °C; R_F (6:4 hexane-EtOAc) 0.27; IR (ATR) 2975, 2930, 2871, 1688 (C=O), 1597, 1493, 1409, 1382, 1179, 1162, 1145, 1099, 910, 729, 676, 598, 573 cm^{-1}; ^1H NMR (400 MHz, $CDCl_3$) (50:50 mixture of rotamers) δ 8.36 (s, 1H, Ar), 7.90–7.85 (m, 2H, Ar), 7.65 (dd, *J* = 8.5, 1.0 Hz, 1H, Ar), 7.33–7.27 (m, 3H, Ar), 6.85 (dd, *J* = 7.5, 1.0 Hz, 1H, Ar), 3.78 (d, *J* = 11.0 Hz, 1H, NCH), 3.73 (d, *J* = 11.0 Hz, 1H, NCH), 3.53 (dd, *J* = 11.0, 4.0 Hz, 1H, NCH), 3.48 (dd, *J* = 11.0, 4.0 Hz, 1H, NCH), 2.38 (s, 3H, Me), 2.34 (t, *J* = 4.0 Hz, 1H, CH), 2.15–2.06 (m, 2H, CH), 1.47 (s, 9H, CMe_3); ^{13}C NMR (100.6 MHz, $CDCl_3$) δ 155.0 (C=O), 146.3 (*ipso*-Ar), 142.3 (*ipso*-Ar), 140.5 (Ar), 134.8 (*ipso*-Ar), 134.5 (*ipso*-Ar), 130.7 (*ipso*-Ar), 130.4 (Ar), 127.3 (Ar), 125.7 (Ar), 120.1 (Ar), 109.6 (Ar), 79.5 (O*C*Me$_3$), 48.8 (NCH_2), 48.6 (NCH_2), 28.6 (C*Me*$_3$), 28.0 (CH), 27.0 (CH), 23.4 (*C*HAr), 21.8 (Me); MS (ESI) *m/z* 476 (M + Na)$^+$; HRMS (ESI) *m/z* calcd for $C_{24}H_{27}N_3O_4S$ (M + Na)$^+$ 476.1614, found 476.1620 (–1.1 ppm error).

Lab Book Reference: SY-1-131

***tert*-Butyl (1*R**,5*S**,6*R**)-6-(4-(methylsulfonyl)phenyl)-3-azabicyclo[3.1.0]hexane-3-carboxylate 68**

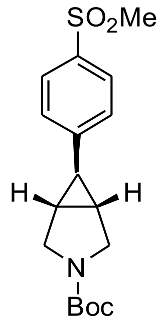

68

Using general procedure A, BMIDA building block **1h** (100 mg, 0.3 mmol, 1.0 eq.), 4-bromophenylmethyl sulfone (97 mg, 0.41 mmol, 1.4 eq.), PCy_3 (25 mg, 0.09 mmol, 0.3 eq.), Cs_2CO_3 (579 mg, 1.8 mmol, 6 eq.) and $Pd(OAc)_2$ (10 mg, 0.04 mmol, 0.15 eq.) in toluene (3.1 mL) and water (0.2 mL) gave crude the product. Purification by flash column chromatography on silica with 60:40 EtOAc-hexane as eluent gave cyclopropyl sulfone **68** (72 mg, 72%) as a white solid, mp 158–160 °C; R_F (60:40 EtOAc-hexane) 0.40; IR (ATR) 2976, 2929, 2873, 1689 (C=O), 1402 (SO_2), 1300, 1147, 1116, 957, 865, 762, 535 cm^{-1}; ^1H NMR (400 MHz, $CDCl_3$) (50:50 mixture of rotamers) δ 7.82 (d, J = 8.5 Hz, 2H, Ar), 7.19 (d, J = 8.5 Hz, 2H, Ar), 3.80 (d, J = 11.0 Hz, 1H, NCH), 3.71 (d, J = 11.0 Hz, 1H, NCH), 3.55–3.45 (m, 2H, NCH), 3.03 (s, 3H, SO_2Me), 1.91–1.86 (m, 2H, CH), 1.80 (t, J = 3.5 Hz, 1H, CH), 1.47 (s, 9H, CMe_3); ^{13}C NMR (100.6 MHz, $CDCl_3$) δ 155.0 (C=O), 148.4 (*ipso*-Ar), 137.8 (*ipso*-Ar), 127.7 (Ar), 126.4 (Ar), 79.9 (O*C*Me$_3$), 48.8 (NCH_2), 48.5 (NCH_2), 44.8 (SO_2Me), 28.6 (C*Me*$_3$), 28.0 (CH), 27.2 (*C*HAr); MS (ESI) *m/z* 360 $(M + Na)^+$; HRMS (ESI) *m/z* calcd for $C_{17}H_{23}NO_4S$ $(M + Na)^+$ 360.1240, found 360.1249 (–2.6 ppm error).

Lab Book Reference: SY-1-53

***tert*-Butyl (1*R**,6*R**,7*S**)-7-(pyrimidin-5-yl)-2-azabicyclo[4.1.0]heptane-2-carboxylate 69**

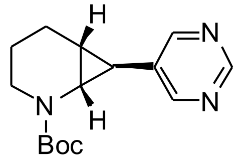

69

Using general procedure B, BMIDA building block **1i** (88 mg, 0.25 mmol, 1.0 eq.), 5-bromopyrimidine (56 mg, 0.35 mmol, 1.4 eq.), PCy_3 (7 mg, 25 μmol, 0.10 eq.), Cs_2CO_3 (244 mg, 0.8 mmol, 3.0 eq.) and $Pd(OAc)_2$ (3 mg, 13 μmol, 0.05 eq.) in toluene (5.0 mL) and water (0.5 mL) gave the crude product. Purification by flash column chromatography on silica with EtOAc as eluent gave cyclopropyl pyrimidine **69** (50 mg, 73%) as an off-white solid, mp 89–91 °C; R_F (EtOAc) 0.20; IR (ATR) 2975, 2933, 2863, 1690 (C=O), 1557, 1410, 1386, 1364, 1159, 1132, 1040, 727, 631 cm^{-1}; ^1H NMR (400 MHz, $CDCl_3$) (70:30 mixture of rotamers) δ 9.00 (s, 0.7H, Ar), 8.98 (s, 0.3H, Ar), 8.49 (s, 0.6H, Ar), 8.47 (s, 1.4H, Ar), 3.86 (ddd, J = 12.5, 4.0, 4.0 Hz, 0.7H, NCH), 3.69 (ddd, J = 12.5, 4.0, 4.0 Hz, 0.3H, NCH), 3.17 (dd, J = 8.5, 3.0 Hz, 0.3H, NCH), 3.01 (dd, J = 8.5, 3.0 Hz, 0.7H, NCH), 2.71 (ddd, J = 12.5, 12.5, 2.5 Hz, 0.3H, NCH), 2.59 (ddd, J = 12.5, 12.5 , 2.5 Hz, 0.8H, NCH), 2.06–1.98 (m, 1H, CH), 1.97–1.84 (m, 1H, CH), 1.75–1.62 (m, 3H, CH), 1.44 (s, 2.7H, CMe_3), 1.43–1.37 (m, 1H, CH), 1.35 (s, 6.3H, CMe_3); ^{13}C NMR (100.6 MHz, $CDCl_3$) (rotamers) δ 156.5 (C=O), 156.3 (C=O), 156.1 (Ar), 155.8 (Ar), 154.6 (Ar), 154.5 (Ar), 134.8 (*ipso*-Ar), 134.7 (*ipso*-Ar), 80.1 (O*C*Me_3), 40.40 (NCH_2), 40.36 (NCH_2), 38.9 (NCH), 38.8 (NCH), 28.59 (C*Me*$_3$), 28.55 (C*Me*$_3$), 24.8 (CH), 24.1 (CH), 22.2 (CH_2), 22.0 (CH_2), 21.8 (CH), 20.4 (CH_2); MS (ESI) *m/z* 298 $(M + Na)^+$; HRMS (ESI) *m/z* calcd for $C_{15}H_{21}N_3O_2$ $(M + Na)^+$ 298.1526, found 298.1529 (–1.0 ppm error).

Lab Book Reference: SY-3-107

Using general procedure A, BMIDA building block **1i** (176 mg, 0.5 mmol, 1.0 eq.), 5-bromopyrimidine (111 mg, 0.7 mmol, 1.4 eq.), PCy_3 (42 mg, 0.15 mmol, 0.3 eq.), Cs_2CO_3 (977 mg, 3.0 mmol, 6 eq.) and $Pd(OAc)_2$ (17 mg, 0.075 mmol, 0.15 eq.) in toluene (6.0 mL) and water (0.5 mL) gave crude the product. Purification by flash column chromatography on silica with EtOAc as eluent gave cyclopropyl pyrimidine **69** (108 mg, 78%) as an off-white solid.

Lab Book Reference: SY-3-86

***tert*-Butyl (1*R**,6*R**,7*S**)-7-(4-chlorophenyl)-2-azabicyclo[4.1.0]heptane-2-carboxylate 70**

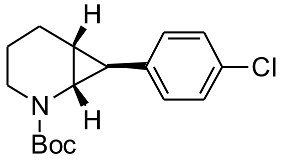

70

Using general procedure B, BMIDA building block **1i** (88 mg, 0.25 mmol, 1.0 eq.), 1-bromo-4-chlorobenzene (67 mg, 0.35 mmol, 1.4 eq.), PCy_3 (7 mg, 25 μmol, 0.10 eq.), Cs_2CO_3 (244 mg, 0.75 mmol, 3.0 eq.) and $Pd(OAc)_2$ (3 mg, 13 μmol, 0.05 eq.) in toluene (5.0 mL) and water (0.5 mL) gave the crude product. Purification by flash column chromatography on silica with 90:10 hexane-EtOAc as eluent gave cyclopropyl chlorobenzene **70** (64 mg, 83%) as an off-white solid, mp 86–88 °C; R_F (8:2 hexane-EtOAc) 0.43; IR (ATR) 2976, 2933, 2861, 1691 (C=O), 1494, 1385, 1364, 1161, 1131, 1090, 894, 849, 731, 511 cm^{-1}; ^1H NMR (400 MHz, $CDCl_3$) (80:20 mixture of rotamers) δ 7.24–7.16 (m, 2H, Ar), 7.11–7.05 (m, 0.4H, Ar), 7.03–6.93 (m, 1.6H, Ar), 3.86 (ddd, J = 12.5, 3.5, 3.5 Hz, 0.8H, NCH), 3.68 (ddd, J = 12.5, 3.5, 3.5 Hz, 0.2H, NCH), 3.06 (dd, J = 8.5, 3.0 Hz, 0.2H, NCH), 2.91 (dd, J = 8.5, 3.0 Hz, 0.8H, NCH), 2.69 (ddd, J = 12.5, 12.5, 3.5 Hz, 0.2H, NCH), 2.58 (ddd, J = 12.5, 12.5, 2.5 Hz, 0.8H, NCH), 2.03–1.95 (m, 1H, CH), 1.93–1.80 (m, 1H, CH), 1.78–1.72 (m, 0.4H, CH), 1.70 (dd, J = 6.5, 3.0 Hz, 1H, CH), 1.68–1.61 (m, 0.6H, CH), 1.59–1.50 (m, 1H, CH), 1.46 (s, 1.8H, CMe_3), 1.45–1.38 (m, 1H, CH), 1.36 (s, 7.2H, CMe_3); ^{13}C NMR (100.6 MHz, $CDCl_3$) (rotamers) δ 156.5 (C=O), 140.0 (Ar), 131.3 (*ipso*-Ar), 131.2 (*ipso*-Ar), 128.4 (Ar), 128.4 (Ar), 128.0 (Ar), 127.3 (Ar), 79.7 (O*C*Me$_3$), 42.0 (NCH_2), 40.4 (NCH_2), 38.8 (NCH), 38.0 (NCH), 29.0 (CH), 28.6 (C*Me*$_3$), 28.2 (CH), 22.2 (CH_2), 21.9 (CH), 21.9 (CH), 20.6 (CH_2), 20.6 (CH_2); MS (ESI) *m/z* 330 [(^{35}M + Na)$^+$; HRMS (ESI) *m/z* calcd for $C_{17}H_{22}{}^{35}ClNO_2$ (^{35}M + Na)$^+$ 330.1231, found 330.1228 (+1.0 ppm error).

Lab Book Reference: SY-3-122

tert-Butyl (1_R_*,6_R_*,7_S_*)-7-(furan-3-yl)-2-azabicyclo[4.1.0]heptane-2-carboxylate 71

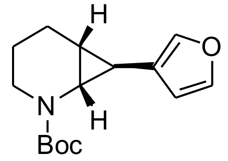

71

Using general procedure B, B(MIDA) building block **1i** (88 mg, 0.25 mmol, 1.0 eq.), 3-bromofuran (51 mg, 0.35 mmol, 1.4 eq.), PCy_3 (7 mg, 25 μmol, 0.10 eq.), Cs_2CO_3 (244 mg, 0.75 mmol, 3.0 eq.) and $Pd(OAc)_2$ (3 mg, 13 μmol, 0.05 eq.) in toluene (5.0 mL) and water (0.5 mL) gave the crude product. Purification by flash column chromatography on silica with 90:10 hexane-EtOAc as eluent gave cyclopropyl furan **71** (40 mg, 61%) as a yellow oil, R_F (9:1 hexane-EtOAc) 0.23; IR (ATR) 2972, 2933, 2865, 1691 (C=O), 1391, 1365, 1254, 1153, 1031, 874, 770, 734, 599 cm^{-1}; ^1H NMR (400 MHz, $CDCl_3$) (80:20 mixture of rotamers) δ 7.31–7.22 (m, 2H, Ar), 6.22–6.17 (m, 1H, Ar), 3.83 (ddd, J = 12.5, 4.0, 4.0 Hz, 0.8H, NCH), 3.65 (ddd, J = 12.5, 4.0, 4.0 Hz, 0.2H, NCH), 2.96 (dd, J = 8.5, 3.0 Hz, 0.2H, NCH), 2.83 (dd, J = 8.5, 3.0 Hz, 0.8H, NCH), 2.66 (ddd, J = 12.5, 12.5, 2.5 Hz, 0.2H, NCH), 2.55 (ddd, J = 12.5, 12.5, 2.5 Hz, 0.8H, NCH), 2.01–1.92 (m, 1H, CH), 1.89–1.75 (m, 1H, CH), 1.70–1.58 (m, 1H, CH), 1.50 (dd, J = 6.0, 3.0 Hz, 1H, CH), 1.46 (s, 1.8H, CMe_3), 1.43 (s, 7.2H, CMe_3), 1.37–1.32 (m, 1H, CH), 1.31–1.22 (m, 1H, CH); ^{13}C NMR (100.6 MHz, $CDCl_3$) (rotamers) δ 156.7 (C=O), 142.9 (Ar), 142.8 (Ar), 138.6 (Ar), 138.4 (Ar), 125.5 (_ipso_-Ar), 109.9 (Ar), 109.8 (Ar), 79.6 (OCMe$_3$), 42.0 (NCH_2), 40.5 (NCH_2), 37.3 (NCH), 37.0 (NCH), 28.7 (CMe_3), 28.7 (CMe_3), 22.3 (CH_2), 20.7 (CH), 20.5 (CH_2), 20.5 (CH), 20.1 (CH), 19.5 (CH); MS (ESI) m/z 286 $(M + Na)^+$; HRMS (ESI) m/z calcd for $C_{15}H_{21}NO_3$ $(M + Na)^+$ 286.1414, found 286.1415 (–0.6 ppm error).

Lab Book Reference: SY-3-113

tert-Butyl (1_R_*,6_R_*,7_S_*)-7-(benzofuran-5-yl)-2-azabicyclo[4.1.0]heptane-2-carboxylate 72

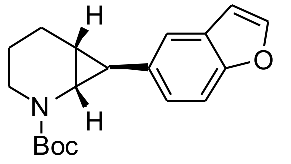

72

Using general procedure B, BMIDA building block **1i** (88 mg, 0.25 mmol, 1.0 eq.), 5-bromobenzofuran (69 mg, 0.35 mmol, 1.4 eq.), PCy_3 (7 mg, 25 μmol, 0.10 eq.), Cs_2CO_3 (244 mg, 0.75 mmol, 3.0 eq.) and $Pd(OAc)_2$ (3 mg, 13 μmol, 0.05 eq.) in toluene (5.0 mL) and water (0.5 mL) gave the crude product. Purification by flash column chromatography on silica with 90:10 hexane-Et_2O as eluent gave cyclopropyl benzofuran **72** (61 mg, 78%) as a pale yellow oil, R_F (9:1 hexane-Et_2O) 0.37; IR (ATR) 2979, 2931, 2860, 1691 (C=O), 1470, 1389, 1364, 1164, 1130, 1032, 765, 735 cm^{-1}; ^1H NMR (400 MHz, $CDCl_3$) (80:20 mixture of rotamers) δ 7.59 (d, J = 2.0 Hz, 0.8H, Ar), 7.55 (d, J = 2.0 Hz, 0.2H, Ar), 7.46–7.44 (m, 0.2H, Ar), 7.39 (d, J = 8.5 Hz, 0.8H, Ar), 7.37–7.36 (m, 0.2H, Ar), 7.33 (d, J = 2.0 Hz, 0.8H, Ar), 7.18 (dd, J = 8.5, 2.0 Hz, 0.2H, Ar), 7.10 (dd, J = 8.5, 2.0 Hz, 0.8H, Ar), 6.72–6.67 (m, 1H, Ar), 3.89 (ddd, J = 12.5, 2.5, 2.5 Hz, 0.8H, NCH), 3.71 (ddd, J = 12.5, 2.5, 2.5 Hz, 0.2H, NCH), 3.13 (dd, J = 8.5, 3.0 Hz, 0.2H, NCH), 2.97 (dd, J = 8.5, 3.0 Hz, 0.8H, NCH), 2.71 (ddd, J = 12.5, 12.5, 2.5 Hz, 0.2H, NCH), 2.60 (ddd, J = 12.5, 12.5, 2.5 Hz, 0.8H, NCH), 2.08–2.01 (m, 1H, CH), 1.95–1.87 (m, 1H, CH), 1.86 (dd, J = 6.5, 3.0 Hz, 1H, CH), 1.74–1.65 (m, 1H, CH), 1.65–1.54 (m, 2H, CH), 1.49 (s, 1.8H, CMe_3), 1.40 (s, 7.2H, CMe_3); ^{13}C NMR (100.6 MHz, $CDCl_3$) (rotamers) δ 156.7 (C=O), 153.7 (*ipso*-Ar), 145.32 (Ar), 145.30 (Ar), 135.8 (*ipso*-Ar), 127.5 (*ipso*-Ar), 123.6 (Ar), 118.3 (Ar), 111.0 (Ar), 106.5 (Ar), 79.7 ($OCMe_3$), 42.1 (NCH_2), 40.6 (NCH_2), 38.4 (NCH), 37.6 (NCH), 29.3 (CH), 28.7 (CMe_3), 22.4 (CH_2), 21.5 (CH), 21.3 (CH), 20.9 (CH_2), 20.8 (CH_2); MS (ESI) m/z 336 $(M + Na)^+$; HRMS (ESI) m/z calcd for $C_{19}H_{23}NO_3$ $(M + Na)^+$ 336.1570, found 336.1573 (–0.7 ppm error).

Lab Book Reference: SY-3-111

tert-Butyl (1_R_*,6_R_*,7_S_*)-7-(4-methoxyphenyl)-2-azabicyclo[4.1.0]heptane-2-carboxylate 73

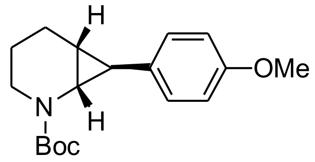

73

Using general procedure A, MIDA boronate **1i** (100 mg, 0.28 mmol, 1.0 eq.), 4-bromoanisole (73 mg, 0.39 mmol, 1.4 eq.), PCy_3 (24 mg, 0.084 mmol, 0.3 eq.), Cs_2CO_3 (547 mg, 1.68 mmol, 6.0 eq.) and $Pd(OAc)_2$ (9.4 mg, 0.042 mmol, 0.15 eq.) in toluene (3.5 mL) and water (0.3 mL) gave the crude product. Purification by flash column chromatography on silica with 95:5 hexane-Et_2O as eluent gave gave aryl cyclopropane **73** (59 mg, 70%) as a colourless oil, R_F (75:25 hexane-Et2O) 0.20; IR (ATR) 2933, 1691 (C=O), 1612, 1514, 1449, 1387, 1369, 1245, 1161, 1129, 1037, 895, 793, 755, 530 cm^{-1}; ^{1}H NMR (400 MHz, $CDCl_3$) (80:20 mixture of rotamers) δ 7.12 (d, J = 8.5 Hz, 0.4H, Ar), 7.03 (d, J = 8.5 Hz, 1.6H, Ar), 6.79 (d, J = 8.5 Hz, 2H, Ar), 3.85 (ddd, J = 11.0, 3.5, 3.5 Hz, 0.8H, NCH), 3.76 (s, 2.4H, OMe), 3.74 (s, 0.6H, OMe), 3.71–3.63 (ddd, J = 11.0, 3.5, 3.5 Hz, 0.2H, NCH), 3.02 (dd, J = 8.5, 3.0 Hz, 0.2H, NCH), 2.87 (dd, J = 8.5, 3.0 Hz, 0.8H, NCH), 2.67 (ddd, J = 12.5, 12.5, 2.5 Hz, 0.2H, NCH), 2.56 (ddd, J = 12.5, 12.5, 2.5 Hz, 0.8H, NCH), 2.03–1.94 (m, 1H, CH), 1.90–1.79 (m, 1H, CH), 1.75–1.61 (m, 2H, CH), 1.55–1.48 (m, 1H, CH), 1.46 (s, 1.8H, CMe_3), 1.39 (s, 7.2H, CMe_3), 1.37–1.22 (m, 1H, CH); ^{13}C NMR (100.6 MHz, $CDCl_3$) (rotamers) δ 157.6 (C=O or _ipso_-Ar), 156.5 (C=O or _ipso_-Ar), 133.3 (_ipso_-Ar), 133.2 (_ipso_-Ar), 127.8 (Ar), 127.2 (Ar), 113.7 (Ar), 113.5 (Ar), 79.4 (_C_Me_3), 55.2 (OMe), 41.9 (NCH_2), 40.3 (NCH_2), 37.9 (NCH), 37.3 (NCH), 28.6 (_C_Me_3), 22.1 (CH_2), 20.9 (CH), 20.8 (CH), 20.6 (CH_2), 20.5 (CH_2); MS (ESI) _m/z_ 326 (M + Na)$^+$; HRMS ESI _m/z_ calcd for $C_{18}H_{25}NO_3$ (M + Na)$^+$ 326.1727, found 326.1725 (+0.5 ppm error).

Lab book reference: CP1-50

***tert*-Butyl (1*R**,6*R**,7*S**)-7-(3-cyano-4-methoxy-phenyl)-2-azabicyclo[4.1.0]heptane-2-carboxylate 74**

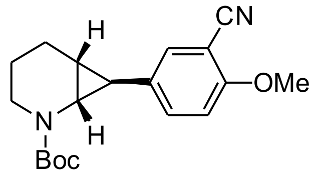

74

Using general procedure A, MIDA boronate **1i** (111 mg, 0.32 mmol, 1.0 eq.), 5-bromo-2-methoxybenzonitrile (83 mg, 0.39 mmol, 1.2 eq.), PCy_3 (24 mg, 0.084 mmol, 0.3 eq.), Cs_2CO_3 (547 mg, 1.68 mmol, 5.25 eq.) and $Pd(OAc)_2$ (9.4 mg, 0.042 mmol, 0.13 eq.) in toluene (3.5 mL) and water (0.3 mL) gave the crude product. Purification by flash column chromatography on silica with 75:25 hexane-EtOAc as eluent gave gave aryl cyclopropane **74** (60 mg, 57%) as a yellow oil, R_F (75:25 hexane-EtOAc) 0.30; IR (ATR) 2935, 2226 (C≡N), 1688 (C=O), 1504, 1451, 1389, 1365, 1280, 1265, 1163, 1127, 1024, 910, 811, 760, 730, 647, 499 cm^{-1}; ^{1}H NMR (400 MHz, $CDCl_3$) (75:25 mixture of rotamers) δ 7.39–7.22 (m, 2H, Ar), 6.84 (d, *J* = 8.5 Hz, 1H, Ar), 3.90–3.83 (s, 3H, OMe), 3.83–3.79 (m, 0.75H, NCH), 3.65 (ddd, *J* = 12.5, 4.0, 4.0 Hz, 0.25H, NCH), 2.96 (dd, *J* = 8.5, 3.0 Hz, 0.25H, NCH), 2.85 (dd, *J* = 8.5, 3.0 Hz, 0.75H, NCH), 2.65 (ddd, *J* = 12.5, 12.5 2.5 Hz, 0.25H, NCH), 2.54 (ddd, *J* = 12.5, 12.5, 2.5 Hz, 0.75H, NCH), 1.99–1.92 (m, 1H, CH), 1.90–1.78 (m, 1H, CH), 1.72–1.59 (m, 2H, CH), 1.54–1.46 (m, 1H, CH), 1.43 (s, 2.25H, CMe_3), 1.37 (s, 6.75H, CMe_3); ^{13}C NMR (100.6 MHz, $CDCl_3$) (roatmers) δ 159.5 (C≡N or C=O), 156.2 (C≡N or C=O), 133.9 (*ipso*-Ar), 133.1 (Ar), 132.5 (Ar), 131.5 (Ar), 131.2 (Ar), 116.6 (*ipso*-Ar), 116.5 (*ipso*-Ar), 111.2 (Ar), 111.1 (Ar), 101.3 (*ipso*-Ar), 79.6 (*C*Me_3), 56.0 (OMe), 41.7 (NCH_2), 40.2 (NCH_2), 38.0 (NCH), 37.6 (NCH), 28.4 (CMe_3), 28.4 (CMe_3), 27.9 (CH), 27.6 (CH_2), 27.3 (CH_2), 22.0 (CH), 21.1 (CH_2); MS (ESI) *m/z* 351 (M + Na)$^+$; HRMS ESI *m/z* calcd for $C_{19}H_{24}N_2O_3$ (M + Na)$^+$ 351.1679, found 351.1679 (0.0 ppm error).

Lab book reference: CP1-51

***tert*-Butyl (1*R**,6*R**,7*S**)-7-(4-methylsulfonylphenyl)-2-azabicyclo[4.1.0]heptane-2-carboxylate 75**

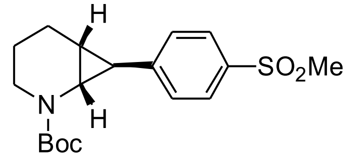

75

Using general procedure A, MIDA boronate **1i** (100 mg, 0.28 mmol, 1.0 eq.), 4-bromophenyl methyl sulfoxide (92 mg, 0.39 mmol, 1.4 eq.), PCy_3 (24 mg, 0.084 mmol, 0.3 eq.), Cs_2CO_3 (547 mg, 1.68 mmol, 6.0 eq.) and $Pd(OAc)_2$ (9.4 mg, 0.042 mmol, 0.15 eq.) in toluene (3.5 mL) and water (0.3 mL) gave the crude product. Purification by flash column chromatography on silica using 70:30 hexane-EtOAc as eluent gave cyclopropyl sulfone **75** (71 mg, 51%) as an off-white solid, R_F (50:50 hexane-EtOAc) 0.64; IR (ATR) 2931, 1689 (C=O), 1596, 1476, 1449, 1388, 1365, 1303, 1081, 957, 915, 893, 790, 585, 548, 533, 492 cm^{-1}; ^{1}H NMR (400 MHz, $CDCl_3$) (70:30 mixture of rotamers) δ 7.79 (d, J = 8.0 Hz, 1.4H, Ar), 7.75 (d, J = 8.0 Hz, 0.6H, Ar), 7.22 (d, J = 8.0 Hz, 1.4H, Ar), 7.18 (d, J = 8.0 Hz, 0.6H, Ar), 3.84 (ddd, J = 13.0, 4.0, 4.0 Hz, 0.7H, NCH), 3.68 (ddd, J = 13.0, 4.0, 4.0 Hz, 0.3H, NCH), 3.15 (dd, J = 8.5, 3.0 Hz, 0.3H, NCH), 3.02 (dd, J = 8.5, 3.0 Hz, 0.7H, NCH), 2.99 (s, 2.1H, SMe), 2.96 (s, 0.9H, SMe), 2.70 (ddd, J = 12.5, 12.5, 2.5 Hz, 0.3H, NCH), 2.58 (ddd, J = 12.5, 12.5, 2.5 Hz, 0.7H, NCH), 2.02–1.95 (m, 1H, CH), 1.94–1.76 (m, 2H, CH), 1.71–1.65 (m, 2H, CH), 1.43 (s, 2.7H, CMe_3), 1.41–0.312 (m, 1H, CH), 1.29 (s, 6.3H, CMe_3); ^{13}C NMR (100.6 MHz, $CDCl_3$) (rotamers) δ 156.2 (C=O), 155.8 (C=O), 148.5 (*ipso*-Ar), 137.4 (*ipso*-Ar), 137.3 (*ipso*-Ar), 127.5 (Ar), 127.5 (Ar), 126.7 (Ar), 126.4 (Ar), 78.0 (*C*Me$_3$), 79.8 (*C*Me$_3$), 44.8 (SMe), 44.7 (SMe), 41.9 (NCH_2), 40.4 (NCH_2), 40.2 (NCH), 39.7 (NCH), 29.9 (CH), 29.0 (CH), 28.5 (C*Me*$_3$), 23.5 (CH), 23.5 (CH), 22.1 (CH_2), 22.0 (CH_2), 20.5 (CH_2); MS (ESI) *m/z* 374 (M + Na)$^+$; HRMS ESI *m/z* calcd for $C_{18}H_{25}NO_4S$ (M + Na)$^+$ 374.1397, found 374.1395 (+0.9 ppm error).

Lab book reference: CP1-49

***tert*-Butyl (1*R**,6*R**,7*S**)-7-(3-acetylphenyl)-2-azabicyclo[4.1.0]heptane-2-carboxylate 76**

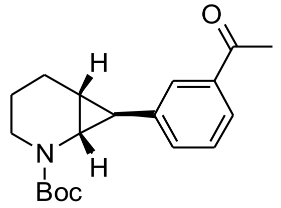

76

Using general procedure B, BMIDA building block **1i** (88 mg, 0.25 mmol, 1.0 eq.), 3-bromoacetophenone (70 mg, 0.35 mmol, 1.4 eq.), PCy_3 (7 mg, 25 μmol, 0.10 eq.), Cs_2CO_3 (244 mg, 0.75 mmol, 3.0 eq.) and $Pd(OAc)_2$ (3 mg, 13 μmol, 0.05 eq.) in toluene (5.0 mL) and water (0.5 mL) gave the crude product. Purification by flash column chromatography on silica with 90:10 hexane-EtOAc as eluent gave cyclopropyl acetophenone **76** (68 mg, 86%) as an off-white solid, mp 90–93 °C; R_F (8:2 hexane-EtOAc) 0.38; IR (ATR) 2975, 2933, 2861, 1683 (C=O), 1387, 1363, 1291, 1252, 1161, 1131, 775, 694, 588 cm^{-1}; ^{1}H NMR (400 MHz, $CDCl_3$) (75:25 mixture of rotamers) δ 7.74–7.62 (m, 2H, Ar), 7.37–7.27 (m, 2H, Ar), 3.86 (ddd, *J* = 13.0, 4.0, 4.0 Hz, 0.75H, NCH), 3.69 (ddd, *J* = 13.0, 3.5, 3.5 Hz, 0.25H), 3.13 (dd, *J* = 8.5, 3.0 Hz, 0.25H, NCH), 2.98 (dd, *J* = 8.5, 3.0 Hz, 0.75H, NCH), 2.75–2.60 (m, 1H, NCH), 2.57 (s, 2.3H, Me), 2.55 (s, 0.7H, Me), 2.04–1.96 (m, 1H, CH), 1.94–1.84 (m, 1H, CH), 1.82 (dd, *J* = 6.5, 3.5, 0.25H, CH), 1.79 (dd, *J* = 6.5, 3.0 Hz, 0.75H, CH), 1.73–1.58 (m, 2H, CH), 1.45 (s, 2.25H, CMe_3), 1.33 (s, 6.75H, CMe_3); ^{13}C NMR (100.6 MHz, $CDCl_3$) (rotamers) δ 198.5 (C=O, ketone), 198.3 (C=O, ketone), 156.4 (C=O, Boc), 155.9 (C=O, Boc), 142.1 (*ipso*-Ar), 137.1 (*ipso*-Ar), 131.2 (Ar), 131.0 (Ar), 128.6 (Ar), 128.5 (Ar), 126.0 (Ar), 125.8 (Ar), 125.3 (Ar), 79.7 (O*C*Me_3), 79.6 (O*C*Me_3), 42.0 (NCH_2), 40.4 (NCH_2), 38.9 (NCH), 38.5 (NCH), 29.4 (CH), 28.5 (C*Me_3*), 26.8 (Me), 22.3 (CH_2), 22.2 (CH_2), 22.1 (CH), 22.0 (CH), 20.6 (CH_2), 20.6 (CH_2); MS (ESI) *m/z* 338 $(M + Na)^+$; HRMS (ESI) *m/z* calcd for $C_{19}H_{25}NO_3$ $(M + Na)^+$ 338.1727, found 338.1726 (+0.1 ppm error).

Lab Book Reference: SY-3-126

6-[(3,5-Dimethyl-1,2-oxazol-4-yl)sulfonyl]-1-[1-(4-methylbenzenesulfonyl)-1H-pyrrolo[2,3-b]pyridin-5-yl]-6-azaspiro[2.5]octane 77

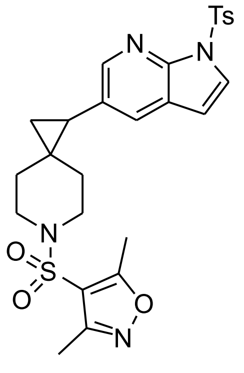

77

4 N HCl in dioxane (3 mL) was added to a stirred solution of arylated piperidine **25** (76 mg, 0.16 mmol, 1.0 eq.) in dioxane (1 mL) at rt under Ar. The resulting mixture was stirred at rt for 1 h and the solvent was concentrated under reduced pressure to give the crude HCl salt. The crude HCl salt was suspended in CH_2Cl_2 and DMAP (2 mg, 0.02 mmol, 0.1 eq.) and Et_3N (120 µL, 0.8 mmol, 5.0 eq.) were added under Ar. The resulting solution was cooled to 0 °C and 3,5-dimethylisoxazole-4-sulfonyl chloride (40 mg, 0.20 mmol, 1.25 eq.) was added and the solution was stirred at rt for 4 h. 1 M $HCl_{(aq)}$ (10 mL) was added and the two layers were separated. The aqueous layer was extracted with CH_2Cl_2 (3 × 10 mL). The combined organic extracts were washed with brine (15 mL), dried ($MgSO_4$) and concentrated under reduced pressure to give the crude product. Purification by flash column chromatography on silica with 7:3 hexane-EtOAc as eluent gave sulfonamide **77** (70 mg, 81%) as a white solid, mp 90–92 °C; R_F (7:3 hexane-EtOAc) 0.24; IR (ATR) 2923, 1371, 1173, 908, 723, 674, 579 cm^{-1}; ^{1}H NMR (400 MHz, $CDCl_3$) δ 8.25 (d, *J* = 2.0 Hz, 1H, Ar), 8.08–8.01 (m, 2H, Ar), 7.67 (d, *J* = 4.0 Hz, 1H, Ar), 7.55 (d, *J* = 2.0 Hz, 1H, Ar), 7.29–7.23 (m, 2H, Ar), 6.51 (d, *J* = 4.0 Hz, 1H, Ar), 3.40 (ddd, *J* = 11.5, 8.5, 3.5 Hz, 1H, NCH), 3.13–3.02 (m, 2H, NCH), 2.81 (ddd, *J* = 11.5, 8.5, 3.5 Hz, 1H, NCH), 2.58 (s, 3H, Me), 2.39–2.32 (m, 6H, Me), 2.06–2.01 (m, 1H, CHAr), 1.83 (ddd, *J* = 13.5, 8.5, 3.5 Hz, 1H, CH), 1.51 (ddd, *J* = 13.5, 6.0, 3.5 Hz, 1H, CH), 1.36 (ddd, *J* = 14.0, 8.5, 4.0 Hz, 1H, CH), 1.08 (ddd, *J* = 14.0, 6.5, 3.5 Hz, 1H, CH), 0.93–0.86 (m, 2H, CH); ^{13}C NMR (100.6 MHz, $CDCl_3$) (conformers) δ 173.6 (*ipso*-Ar), 158.0 (*ipso*-Ar), 146.1 (Ar), 146.0 (Ar), 145.3 (*ipso*-Ar), 135.4 (*ipso*-Ar), 129.7 (Ar), 129.4 (*ipso*-Ar), 129.3 (Ar) 129.0 (*ipso*-Ar), 128.1 (Ar), 128.0 (Ar), 127.0 (Ar), 126.9 (Ar), 122.6 (*ipso*-Ar), 114.0 (*ipso*-Ar), 105.0 (Ar), 104.9 (Ar), 45.3 (NCH_2), 45.0 (NCH_2), 35.9 (CH_2), 25.9 (CHAr), 25.8 (CHAr), 23.9 (C), 21.73 (Me), 21.69 (Me), 15.9 (CH*C*H$_2$), 13.0 (Me), 11.4 (Me), 11.37 (Me); HRMS *m/z* calcd for $C_{26}H_{28}N_4O_5S_2$ (M + Na)$^+$ 563.1393, found 563.1401 (–1.4 ppm error). Lab book reference: ARG-3-035

6-[(3,5-Dimethyl-1,2-oxazol-4-yl)sulfonyl]-1-{1H-pyrrolo[2,3-b]pyridin-5-yl}-6-azaspiro[2.5]octane 78

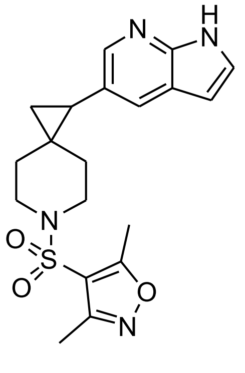

78

Cs_2CO_3 (166 mg, 0.51 mmol, 3.0 eq.) was added to a stirred solution of sulfonamide **77** (65 mg, 0.12 mmol, 1.0 eq.) in THF (1.1 mL) and MeOH (0.6 mL) and the resulting mixture was stirred at rt for 16 h. H_2O (10 mL) and EtOAc (10 mL) were added, and the two layers were separated. The aqueous layer was extracted with EtOAc (3 × 10 mL). The combined organic extracts were washed with brine (15 mL), dried ($MgSO_4$) and concentrated under reduced pressure to give the crude product. Purification by flash column chromatography on silica with 4:1 CH_2Cl_2-EtOAc as eluent gave *N*'-H sulfonamide **78** (41 mg, 72%) as a white solid, mp 96–98 °C; R_F (4:1 CH_2Cl_2-EtOAc) 0.14; IR (ATR) 3133 (NH), 2923, 2852, 1585, 1405, 1336, 1177, 905, 724, 624, 571 cm^{-1}; ^1H NMR (400 MHz, $CDCl_3$) δ 10.77 (s, 1H, NH), 8.22–8.12 (m, 1H, Ar), 7.78–7.61 (m, 1H, Ar), 7.46–7.34 (m, 1H, Ar), 6.61–6.37 (m, 1H, Ar), 3.42 (ddd, *J* = 11.0, 6.0, 4.0 Hz, 1H, NCH), 3.17–3.07 (m, 2H, NCH), 2.86 (ddd, *J* = 11.5, 8.5, 3.5 Hz, 1H, NCH), 2.61 (s, 3H, Me), 2.39 (s, 3H, Me), 2.13 (dd, *J* = 8.5, 6.0 Hz, 1H, CHAr), 1.85 (ddd, *J* = 13.5, 9.0, 3.5 Hz, 1H, CH), 1.58 (ddd, *J* = 13.5, 6.0, 3.5 Hz, 1H, CH), 1.42 (ddd, *J* = 13.0, 8.5, 4.0 Hz, 1H, CH), 1.21–1.14 (m, 1H, CH), 0.97 (dd, *J* = 6.0, 5.5 Hz, 1H, CH), 0.90 (dd, *J* = 8.5, 5.5 Hz, 1H, CH); ^{13}C NMR (100.6 MHz, $CDCl_3$) δ 173.6 (*ipso*-Ar), 158.1 (*ipso*-Ar), 147.8 (Ar), 144.0 (Ar), 128.9 (*ipso*-Ar), 125.9 (Ar), 125.4 (*ipso*-Ar), 120.2 (*ipso*-Ar), 114.0 (*ipso*-Ar), 100.4 (Ar), 45.5 (NCH_2), 45.1 (NCH_2), 36.0 (CH_2), 29.8 (CH_2), 26.2 (CHAr), 23.5 (C), 15.8 (CH*C*H_2), 13.1 (Me), 11.5 (Me); HRMS *m/z* calcd for $C_{19}H_{22}N_4O_3S$ $(M + H)^+$ 387.1485, found 387.1489 (–1.0 ppm error).

Lab book reference: ARG-3-038

1-{1-[1-(4-Methylbenzenesulfonyl)-1H-pyrrolo[2,3-b]pyridin-5-yl]-6-azaspiro[2.5]octan-6-yl}prop-2-en-1-one S33

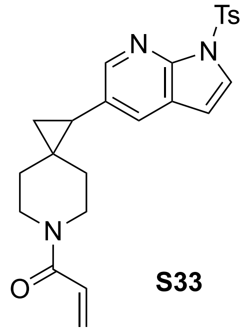

S33

4 N HCl in dioxane (5 mL) was added to a stirred solution of arylated piperidine **25** (150 mg, 0.31 mmol, 1.0 eq.) in dioxane (1 mL) at rt under Ar. The resulting mixture was stirred at rt for 1 h and the solvent was concentrated under reduced pressure to give the crude HCl salt. The crude HCl salt was suspended in CH_2Cl_2 (4 mL) and DMAP (4 mg, 0.03 mmol, 0.1 eq.) and Et_3N (0.2 mL, 1.4 mmol, 4.5 eq.) were added under Ar. The resulting solution was cooled to 0 °C and acryloyl chloride (88 μL, 1.09 mmol, 3.5 eq.) was added and the solution was stirred at rt for 4 h. 1 M $HCl_{(aq)}$ (15 mL) was added and the two layers were separated. The aqueous layer was extracted with CH_2Cl_2 (3 × 10 mL). The combined organic extracts were washed with brine (15 mL), dried ($MgSO_4$) and concentrated under reduced pressure to give the crude product. Purification by flash column chromatography on silica with 9:1 CH_2Cl_2-EtOAc as eluent gave acrylamide **S33** (82 mg, 61%) as a white semi-solid, R_F (9:1 CH_2Cl_2-EtOAc) 0.10; IR (ATR) 2920, 2239, 1641 (C=O), 1442, 1372, 1163, 907, 724, 673, 579 cm^{-1}; ^{1}H NMR (400 MHz, $CDCl_3$) (55:45 mixture of rotamers) δ 8.30 (d, J = 2.0 Hz, 1H, Ar), 8.09–7.96 (m, 2H, Ar), 7.66 (d, J = 4.0 Hz, 1H, Ar), 7.60–7.57 (m, 1H, Ar), 7.28–7.21 (m, 2H, Ar), 6.58 (dd, J = 16.5, 10.5 Hz, 0.45H, =CH), 6.50 (d, J = 4.0 Hz, 1H, Ar), 6.46 (dd, J = 16.5, 10.5 Hz, 0.55H, =CH), 6.27–6.17 (m, 1H, =CH), 5.69–5.55 (m, 1H, =CH), 3.91–3.81 (m, 0.55H, NCH), 3.72–3.60 (m, 1H, NCH), 3.59–3.50 (m, 0.9H, NCH), 3.39–3.21 (m, 1.55H, NCH), 2.34 (s, 3H, Me), 2.07–2.02 (m, 1H, CHAr), 1.70–1.61 (m, 1H, CH), 1.56–1.44 (m, 1H, CH), 1.24–1.15 (m, 1.1H, CH), 1.13–1.03 (m, 0.9H, CH), 0.99–0.91 (m, 2H, CH); ^{13}C NMR (100.6 MHz, $CDCl_3$) (rotamers) δ 165.6 (C=O), 146.3 (Ar), 146.2 (Ar), 146.0 (*ipso*-Ar), 145.3 (*ipso*-Ar), 135.5 (*ipso*-Ar), 129.8 (Ar), 129.5 (*ipso*-Ar), 129.3 (br, Ar), 128.1 (Ar), 128.0 (=CH), 127.5 (=CH_2), 126.9 (Ar), 122.6 (*ipso*-Ar), 105.0 (Ar), 45.9 (NCH_2), 45.4 (NCH_2), 42.7 (NCH_2), 41.6 (NCH_2), 37.4 (CH_2), 36.2 (CH_2), 31.0 (CH_2), 29.8 (CH_2), 26.1 (CHAr), 25.0 (C), 21.7 (Me), 16.1 (CHCH$_2$); HRMS *m/z* calcd for $C_{24}H_{25}N_3O_3S$ (M + Na)$^+$ 458.1509, found 458.1513 (–0.9 ppm error).

Lab book reference: ARG-3-053

1-(1-{1H-Pyrrolo[2,3-b]pyridin-5-yl}-6-azaspiro[2.5]octan-6-yl)prop-2-en-1-one 79

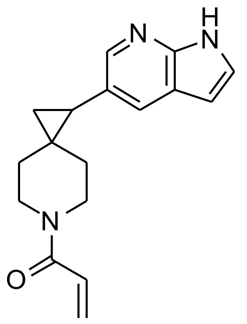

79

Cs_2CO_3 (110 mg, 0.34 mmol, 3.0 eq.) was added to a stirred solution of sulfonamide **S33** (50 mg, 0.11 mmol, 1.0 eq.) in THF (1.1 mL) and MeOH (0.35 mL) and the resulting mixture was stirred at rt for 8 h. H_2O (10 mL) and EtOAc (10 mL) were added and the two layers were separated. The aqueous layer was extracted with EtOAc (3 × 10 mL). The combined organic extracts were washed with brine (15 mL), dried ($MgSO_4$) and concentrated under reduced pressure to give the crude product. Purification by flash column chromatography on silica with 1:9 CH_2Cl_2-EtOAc as eluent gave *N*'-H sulfonamide **79** (19 mg, 61%) as a clear oil, R_F (1:8 CH_2Cl_2-EtOAc) 0.20; IR (ATR) 3203 (NH), 2923, 2853, 1640 (C=O), 1443, 1222, 729 cm^{-1}; ^1H NMR (400 MHz, $CDCl_3$) (55:45 mixture of rotamers) δ 9.91–9.76 (m, 1H, NH), 8.23 (s, 1H, Ar), 7.72 (s, 1H, Ar), 7.36–7.29 (m, 1H, Ar), 6.62 (dd, *J* = 17.0, 10.5 Hz, 0.45H, =CH), 6.55–6.41 (m, 1.55H, =CH, Ar), 6.31–6.18 (m, 1H, =CH), 5.72–5.57 (m, 1H, =CH), 3.95–3.85 (m, 0.55H, NCH), 3.76–3.68 (m, 1H, NCH), 3.66–3.54 (m, 0.9H, NCH), 3.45–3.27 (m, 1.55H, NCH), 2.20–2.09 (m, 1H, CHAr), 1.76–1.65 (m, 1H, CH), 1.62–1.52 (m, 1H, CH), 1.34–1.22 (m, 1H, CH), 1.22–1.11 (m, 1H, CH), 1.08–0.99 (m, 1H, CH), 1.02–0.92 (m, 1H, CH); ^{13}C NMR (100.6 MHz, $CDCl_3$) (rotamers) δ 165.7 (C=O), 147.6 (*ipso*-Ar), 144.5 (Ar), 128.8 (Ar), 128.2 (Ar), 128.1 (Ar), 127.4 (=CH_2), 125.5 (Ar), 120.0 (*ipso*-Ar), 100.7 (=CH), 46.1 (NCH_2), 45.6 (NCH_2), 42.3 (NCH_2), 41.8 (NCH_2), 37.6 (CH_2), 36.4 (CH_2), 31.2 (CH_2), 30.0 (CH_2), 29.8 (CH_2), 26.4 (CHAr), 24.6 (C), 16.0 ($CH\mathit{C}H_2$) (1 × *ipso*-Ar resonance not resolved); HRMS *m/z* calcd for $C_{17}H_{20}N_3O$ $(M + H)^+$ 282.1601, found 282.1605 (–1.5 ppm error).

Lab book reference: ARG-3-064

(1*R,6*R**,7*R**)-7-(2-Methoxypyridin-4-yl)-3-[(oxan-4-yl)methyl]-3-azabicyclo[4.1.0]heptane 80**

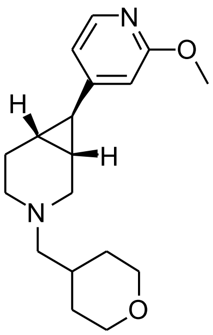

80

4 N HCl in dioxane (4 mL) was added stirred solution of arylated piperidine **49** (80 mg, 0.26 mmol, 1.0 eq.) in dioxane (1 mL) at rt under Ar. The resulting mixture was stirred at rt for 1 h under Ar and the solvent was concentrated under reduced pressure to give the crude HCl salt. The crude HCl salt was suspended in DCE (2.6 mL) and tetrahydro-2H-pyran-4-carbaldehyde (45 mg, 0.39 mmol, 1.5 eq.) and AcOH (59 µL, 1.0 mmol, 4.0 eq.) were added under Ar. The resulting solution was cooled to 0 °C and $NaBH(OAc)_3$ (220 mg, 1.0 mmol, 4.0 eq.) was added and the solution stirred at rt for 4 h. Saturated $NaHCO_{3(aq)}$ (20 mL) was added, and the two layers separated. The aqueous layer was extracted with EtOAc (3 × 15 mL). The combined organic extracts were washed with brine (15 mL), dried ($MgSO_4$) and concentrated under reduced pressure to give the crude product. Purification by flash column chromatography on silica with 4:1 EtOAc-hexane as eluent gave amine **80** (54 mg, 67%) as a yellow oil; R_F (4:1 EtOAc-hexane) 0.17; IR (ATR) 2926, 2839, 1611, 1454, 1390, 1093, 851 cm^{-1}; ^1H NMR (400 MHz, $CDCl_3$) δ 7.95 (d, *J* = 5.5 Hz, 1H, Ar), 6.49 (d, *J* = 5.5 Hz, 1H, Ar), 6.33 (s, 1H, Ar), 4.00–3.93 (m, 3H, OCH), 3.89 (s, 3H,OCH_3), 3.41–3.33 (m, 2H, OCH, NCH), 2.87 (d, *J* = 11.5 Hz, 1H, NCH), 2.56–2.51 (m, 1H, NCH), 2.38–2.32(m, 1H, NCH), 2.15–1.99 (m, 4H, CHAr, NCH), 1.94–1.81 (m, 2H, CH), 1.76–1.58 (m, 4H, CH), 1.48–1.41 (m, 1H, CH), 1.40–1.33 (m, 1H, CH); ^{13}C NMR (100.6 MHz, CD_2Cl_2) δ 165.0 (*ipso*-Ar), 157.2 (*ipso*-Ar), 146.8 (Ar), 114.8 (Ar), 106.8 (Ar), 68.4 (OCH_2), 65.3 (NCH_2), 53.6 (OMe), 53.4 (NCH_2), 51.2 (NCH_2), 33.2 (CH), 32.4 (CH_2), 28.0 (CHAr), 25.9 (CH), 24.9 (CH2), 22.3 (CH); HRMS *m/z* calcd for $C_{18}H_{26}N_2O_2$ $(M + H)^+$ 303.2067, found 303.2068 (–0.5 ppm error).

Lab book reference: ARG-3-022

2-(1H-Indol-3-yl)-1-[(1*R,6*R**,7*R**)-7-(2-methoxypyridin-4-yl)-3-azabicyclo[4.1.0]heptan-3-yl]ethan-1-one 81**

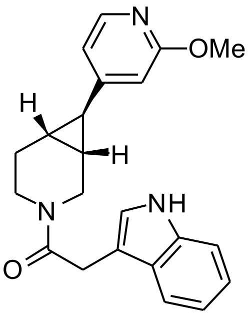

81

4 N HCl in dioxane (4 mL) was added to a stirred solution of arylated piperidine **49** (103 mg, 0.34 mmol, 1.0 eq.) in dioxane (1 mL) at rt under Ar. The resulting mixture was stirred at rt for 1 h and the solvent was concentrated under reduced pressure to give the crude HCl salt. The crude HCl salt was suspended in EtOAc (3 mL) and 3-indoleacetic acid (66 mg, 0.37 mmol, 1.1 eq.) and Et_3N (0.19 mL, 1.4 mmol, 4.0 eq.) were added under Ar. The resulting solution was cooled to 0 °C and a 50% solution of T3P® in toluene (0.35 mL, 0.68 mmol, 2.0 eq.) was added and the resulting solution was stirred and heated at 80 °C for 18 h. After being allowed to cool to rt, EtOAc (20 mL) was added and the solution was washed with saturated $NaHCO_{3(aq)}$ (10 mL), 0.5 M $HCl_{(aq)}$ (10 mL) and brine (15 mL), dried ($MgSO_4$) and concentrated under reduced pressure to give the crude product. Purification by flash column chromatography on silica with 99:1 to 9:1 EtOAc-MeOH as eluent gave amide **81** (62 mg, 53%) as a red solid, mp 200-202 °C (decomposition); R_F (19:1 EtOAc-MeOH) 0.21; IR (ATR) 3255 (NH), 2925, 1612 (C=O), 1453, 1165, 745 cm^{-1}; ^1H NMR (400 MHz, $CDCl_3$) (50:50 mixture of rotamers) δ 8.25 (s, 1H, NH), 7.92 (d, *J* = 5.5 Hz, 0.5H, Ar), 7.87 (d, *J* = 5.5 Hz, 0.5H, Ar), 7.72–7.66 (m, 1H, Ar), 7.37 (dd, *J* = 7.5, 7.5 Hz, 1H, Ar), 7.26–7.05 (m, 3H, Ar), 6.33 (dd, *J* = 5.5, 1.5 Hz, 0.5H, Ar), 6.18 (d, *J* = 1.5 Hz, 0.5H, Ar), 6.10 (dd, *J* = 5.5, 1.5 Hz, 0.5H, Ar), 5.99 (d, *J* = 1.5 Hz, 0.5H, Ar), 4.42 (d, *J* = 14.0 Hz, 0.5H, NCH), 4.08–4.00 (m, 1H, NCH), 3.91–3.78 (m, 5H, OMe, C(O)CH), 3.64 (dd, *J* = 14.0, 4.5 Hz, 0.5H, NCH), 3.54 (ddd, *J* = 14.0, 5.0, 5.0 Hz, 0.5H, NCH), 3.45 (dd, *J* = 14.0, 4.5 Hz, 0.5H), 3.07 (ddd, *J* = 13.0, 10.5, 5.5 Hz, 0.5H, NCH), 2.83 (ddd, *J* = 13.0, 10.5, 5.5 Hz, 0.5H, NCH), 2.06– 1.95 (m, 0.5H, CHAr), 1.89–1.68 (m, 1.5H, CHAr, CH), 1.45–1.26 (m, 2.5H, CH), 1.12–1.03 (m, 0.5H, CH); ^{13}C NMR (100.6 MHz, $CDCl_3$) (rotamers) δ 170.8 (C=O), 170.4 (*ipso*-Ar), 154.9 (*ipso*-Ar), 146.4 (*ipso*-Ar), 122.6 (Ar), 122.5 (*ipso*-Ar) 120.0 (Ar), 119.9 (Ar), 119.2 (Ar), 119.1 (Ar), 118.3 (*ipso*-Ar) 114.5 (Ar), 114.3 (Ar), 111.4 (Ar), 109.5 (*ipso*-Ar), 109.4 (*ipso*-Ar) 106.7 (Ar), 53.5 (OMe), 44.3 (NCH_2), 43.1 (NCH_2),

40.3 (NCH2), 39.2 (CH2), 32.2 (CH2Ar), 32.1 (CH2), 29.8 (CH2), 26.9 (CH2), 26.5 (CH2), 23.3 (CHAr), 23.0 (CH), 22.9 (CH), 22.5 (CH), 21.1 (CH), 20.9 (CH); HRMS *m/z* calcd for $C_{22}H_{23}N_3O_2$ $(M + H)^+$ 362.1863, found 362.1867 (–1.2 ppm error).

Lab book reference: ARG-3-029

6-(2H-1,3-Benzodioxol-5-yl)-1-(pyrimidin-5-yl)-6-azaspiro[2.5]octane 82

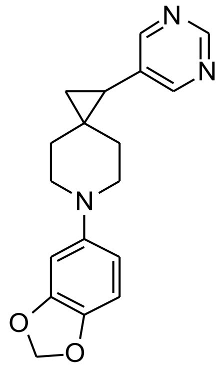

82

4 N HCl in dioxane (4 mL) was added to a stirred solution of arylated piperidine **22** (115 mg, 0.40 mmol, 1.0 eq.) in dioxane (1 mL) at rt under Ar. The resulting mixture was stirred at rt for 1 h and the solvent was concentrated under reduced pressure to give the crude HCl salt. The crude HCl salt was suspended in toluene (1.5 mL) and 3,4-methylenedioxybromobenzene (88 mg, 0.44 mmol, 1.1 eq.), $Pd_2(dba)_3$ (37 mg, 0.04 mmol, 0.1 eq.), (±)-BINAP (37 mg, 0.06 mmol, 0.15 eq.) and NaO*t*-Bu (96 mg, 1.0 mmol, 2.5 eq.) were added under Ar and the resulting mixture was stirred and heated at 110 °C for 48 h. After being allowed to cool to rt, EtOAc (5 mL) was added and the solids were removed by filtration through celite, eluting with EtOAc (25 mL). The filtrate was concentrated under reduced pressure to give the crude product. Purification by flash column chromatography on silica with 4:1 hexane-EtOAc to 1:1 hexane-EtOAc as eluent gave aniline **82** (66 mg, 54%) as a clear oil, R_F (1:1 hexane-EtOAc) 0.20; IR (ATR) 2923, 1503, 1488, 1197, 1036, 729 cm^{-1}; ^1H NMR (400 MHz, $CDCl_3$) δ 9.05 (s, 1H, Ar), 8.59 (s, 2H, Ar), 6.69 (d, J = 8.5 Hz, 1H, Ar), 6.54 (d, J = 2.5 Hz, 1H, Ar), 6.35 (dd, J = 8.5, 2.5 Hz, 1H, Ar), 5.88 (s, 2H, OCH2), 3.23–3.16 (m, 1H, NCH), 3.10 (ddd, J = 11.5, 8.0, 3.5 Hz, 1H, NCH), 2.90–2.86 (m, 2H, NCH), 1.93 (dd, J = 8.0, 6.0 Hz, 1H, CHAr), 1.84 (ddd, J = 12.5, 8.0, 3.5 Hz, 1H, CH), 1.64 (ddd, J = 13.5, 6.5, 3.5 Hz, 1H, CH), 1.45–1.37 (m, 1H, CH), 1.32–1.23 (m, 1H, CH), 1.06–0.99 (m, 2H, CH); ^{13}C NMR (100.6 MHz, $CDCl_3$) δ 157.2 (Ar), 156.6 (Ar), 148.2 (*ipso*-Ar), 147.9 (*ipso*-Ar), 141.7 (*ipso*-Ar),

132.9 (*ipso*-Ar), 110.0 (Ar), 108.2 (Ar), 101.0 (OCH_2), 100.9 (Ar), 51.4 (NCH_2), 50.9 (NCH_2), 36.7 (CH_2), 30.1 (CH_2), 25.5 (C), 23.5 (CHAr), 16.0 (CH*C*H_2); HRMS *m/z* calcd for $C_{18}H_{19}N_3O_2$ $(M + H)^+$ 310.1550, found 310.1555 (–1.8 ppm error).

Lab book reference: ARG-3-049

3-[1-(2-Methoxypyrimidin-5-yl)-2-azabicyclo[3.1.0]hexan-2-yl]-3-oxopropanenitrile 83

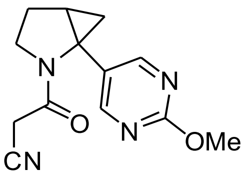

83

4 N HCl in dioxane (3 mL) was added to a stirred solution of arylated pyrrolidine **16** (51 mg, 0.18 mmol, 1.0 eq.) in dioxane (0.5 mL) at rt under Ar. The resulting mixture was stirred at rt for 1 h and the solvent was concentrated under reduced pressure to give the crude HCl salt. The crude HCl salt was suspended in dioxane (1.7 mL) and 3-(3,5-dimethyl-1H-pyrazol-1-yl)-3-oxopropanenitrile **84** (72 mg, 0.44 mmol, 2.5 eq.) and DIPEA (0.14 mL, 0.79 mmol, 4.5 eq.) were added under Ar. The resulting solution was stirred and heated at 100 °C for 18 h. After being allowed to cool to rt, 1 M $HCl_{(aq)}$ (10 mL) was added and the two layers were separated. The aqueous layer was extracted with EtOAc (3 × 10 mL). The combined organic extracts were washed with brine (15 mL), dried ($MgSO_4$) and concentrated under reduced pressure to give the crude product. Purification by flash column chromatography on silica with 4:1 EtOAc-hexane as eluent gave amide **83** (23 mg, 52%) as a pale-yellow semi-solid, R_F (4:1 EtOAc-hexane) 0.11; IR (ATR) 2937, 2258 (C≡N), 1661 (C=O), 1474, 1415, 1327, 1033, 801 cm^{-1}; ^1H NMR (400 MHz, $CDCl_3$) (50:50 mixture of rotamers) δ 8.51–8.40 (m, 2H, Ar), 4.19–4.06 (m, 1H, NCH), 4.03–3.95 (m, 3.5H, OMe, NCH), 3.58–3.50 (m, 0.5H, NCH), 3.41 (s, 1H, C(O)CH), 3.34 (d, *J* = 18.0 Hz, 0.5H, C(O)CH), 3.13 (d, *J* = 18.0 Hz, 0.5H, C(O)CH), 2.61–2.51 (m, 0.5H, CH), 2.51–2.40 (m, 0.5H, CH), 2.20–2.07 (m, 1H, CH), 2.04–1.93 (m, 0.5H, CH), 1.88–1.81 (m, 0.5H, CH), 1.75–1.62 (m, 1H, CH), 1.26 (dd, *J* = 5.0, 5.0 Hz, 0.5H, CH), 1.14 (dd, *J* = 5.0, 5.0 Hz, 0.5H, CH); ^{13}C NMR (100.6 MHz, $CDCl_3$) (rotamers) δ 165.4 (*ipso*-Ar), 164.9 (*ipso*-Ar), 162.4 (C=O), 161.6 (C=O), 159.6 (Ar), 157.7 (Ar), 126.2 (*ipso*-Ar), 125.8 (*ipso*-Ar), 113.6 (C≡N), 113.5 (C≡N), 55.4 (OMe), 55.1 (OMe), 53.4 (NCH_2), 50.8 (NCH_2), 47.0 (NC), 45.7 (NC), 32.6 (CH), 27.1 (CH_2), 26.8 (C(O)*C*H_2), 26.7 (C(O)*C*H_2), 26.0 (CH),

25.5 (CH_2), 21.8 (CH_2); HRMS *m/z* calcd for $C_{13}H_{15}N_4O_2$ $(M + Na)^+$ 281.1009, found 281.1003 (2.3 ppm error).

Lab book reference: ARG-3-057

1-(2-Methoxypyrimidin-5-yl)-2-{7H-pyrrolo[2,3-d]pyrimidin-4-yl}-2-azabicyclo[3.1.0]hexane 85

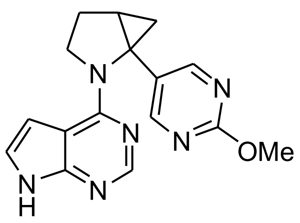

85

4 N HCl in dioxane (4 mL) was added to a stirred solution of arylated pyrrolidine **16** (55 mg, 0.19 mmol, 1.0 eq.) in dioxane (0.5 mL) at rt under Ar. The resulting mixture was stirred at rt for 1 h and the solvent was concentrated under reduced pressure to give the crude HCl salt. The crude HCl salt was suspended in *t*-BuOH (1 mL) and H_2O (0.8 mL) and 4-chloro-7H-pyrrolo[2,3-d]pyrimidine (31 mg, 0.20 mmol, 1.1 eq.) and K_3PO_4 (242 mg, 1.14 mmol, 6.0 eq.) were added under Ar. The resulting solution was stirred and heated at 75 °C for 18 h. After being allowed to cool to rt, 1 M $HCl_{(aq)}$ (10 mL) and EtOAc (10 mL) were added and the two layers were separated. The aqueous layer was extracted with EtOAc (3 × 10 mL). The combined organic extracts were washed with brine (15 mL), dried ($MgSO_4$) and concentrated under reduced pressure to give the crude product. Purification by flash column chromatography on silica with EtOAc to 95:5 EtOAc-MeOH as eluent gave diarylated pyrrolidine **85** (23 mg, 40%) as a white semi-solid, R_F (95:5 EtOAc-MeOH) 0.32; IR (ATR) 3193 (NH), 2927, 1456, 1430, 1276, 1003, 726 cm^{-1}; ^1H NMR (400 MHz, $CDCl_3$) δ 10.39 (s, 1H, NH), 8.39 (s, 2H, Ar), 8.28 (s, 1H, Ar), 6.97 (d, *J* = 3.5 Hz, 1H, Ar), 6.33 (d, *J* = 3.5 Hz, 1H, Ar), 4.39 (ddd, *J* = 11.0, 9.0, 7.0 Hz, 1H, NCH), 4.28–4.17 (m, 1H, NCH), 3.94 (s, 3H, OMe), 2.64–2.52 (m, 1H, CH), 2.16–2.06 (m, 1H, CH), 2.04 (dd, *J* = 9.0, 6.0 Hz, 1H, CH), 1.90 (ddd, *J* = 9.0, 7.0, 6.0 Hz, 1H, CH), 1.29 (ddd, *J* = 6.0, 6.0 Hz, 1H, CH); ^{13}C NMR (100.6 MHz, Acetone-d_6) δ 157.1 (Ar), 151.1 (*ipso*-Ar), 143.9 (*ipso*-Ar), 129.1 (*ipso*-Ar), 120.9 (Ar), 120.1 (Ar), 108.1 (Ar), 101.5 (Ar), 54.8 (OMe), 54.0 (NCH_2), 53.2 (NCH), 47.7 (CAr), 42.0 (CH or CH_2), 30.5 (CH or CH_2), 27.9 (CH or CH_2); HRMS *m/z* calcd for $C_{16}H_{16}N_6O$ $(M + Na)^+$ 331.1283, found 331.1286 (–0.9 ppm error).

Lab book reference: ARG-3-042

2-Methanesulfonyl-1-(pyrimidin-5-yl)-2-azabicyclo[3.1.0]hexane 86

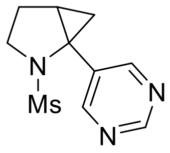

86

Using general procedure D, *N*-Boc aryl cyclopropane **15** (76 mg, 0.29 mmol, 1.0 eq.) was reacted with HCl in dioxane (6 mL of 4 M solution) then, Et_3N (141 mg, 195 µL, 1.40 mmol, 4.8 eq.) and MsCl (73 mg, 50 µL, 0.64 mmol, 2.2 eq.) in CH_2Cl_2 (5 mL) to give the crude product. Purification by flash column chromatography on silica with 8:2 EtOAc-acetone as eluent gave sulfonamide **86** (60 mg, 86%) as a colourless solid, mp 136–137 °C; R_F (8:2 EtOAc-acetone) 0.24; IR (ATR) 3047, 2994, 2920, 1563, 1420, 1321, 1239, 1156, 1145, 1086, 1048, 1029, 1010, 976, 955, 906, 804, 752, 726, 630 cm^{-1}; ^{1}H NMR (400 MHz, $CDCl_3$) δ 9.04 (s, 1H, Ar), 8.73 (s, 2H, Ar), 3.73 (ddd, *J* = 10.0, 10.0, 2.5 Hz, 1H, NCH), 3.20 (ddd, *J* = 10.0, 10.0, 8.0 Hz, 1H, NCH), 2.80 (s, 3H, CH_3), 2.46–2.34 (m, 1H, CH), 2.15 (ddd, *J* = 13.0, 8.0, 2.5 Hz, 1H, CH), 1.69–1.60 (m, 2H, CH), 1.49–1.41 (m, 1H, CH); ^{13}C NMR (100.6 MHz, $CDCl_3$) δ 157.2 (Ar), 156.1 (Ar), 133.5 (*ipso*-Ar), 48.3 (NCH_2), 46.5 (NC), 37.5 (SO_2Me), 28.5 (CH), 25.9 (CH_2), 13.9 (CH_2); HRMS (ESI) *m/z* calcd for $C_{10}H_{14}N_3O_2S$ $(M + H)^+$ 240.0801, found 240.0793 (+3.3 ppm error).

The structure was confirmed by X-Ray crystallography (CCDC 2219566):

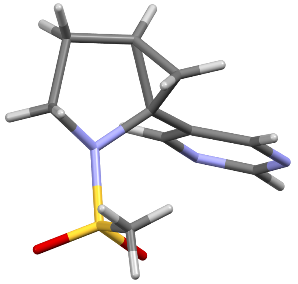

Lab book reference JRD_IX_3

6-Methanesulfonyl-1-(pyrimidin-5-yl)-6-azaspiro[2.5]octane 87

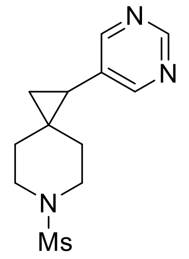

87

Using general procedure D, *N*-Boc aryl cyclopropane **22** (97 mg, 0.34 mmol, 1.0 eq.) was reacted with HCl in dioxane (7.26 mL of 4 M solution) then, Et_3N (176 mg, 243 µL, 1.74 mmol, 4.8 eq.) and MsCl (91 mg, 62 µL, 0.80 mmol, 2.2 eq.) in CH_2Cl_2 (6 mL) to give the crude product. Purification by flash column chromatography on silica with 9:1 EtOAc-acetone as eluent gave sulfonamide **87** (84 mg, 94%) as a colourless gum that solidified on standing, mp 141–142 °C; R_F (9:1 EtOAc-acetone) 0.27; IR (ATR) 2922, 2848, 1556, 1415, 1321, 1236, 1151, 1047, 934, 950, 774, 728, 632, 515 cm^{-1}; ^{1}H NMR (400 MHz, $CDCl_3$) δ 9.02 (s, 1H, Ar), 8.54 (s, 2H, Ar), 3.46–3.36 (m, 1H, NCH), 3.23–3.07 (m, 2H, NCH), 2.98 (ddd, *J* = 12.0, 8.5, 3.5 Hz, 1H, NCH), 2.74 (s, 3H, SO_2Me), 1.94 (dd, *J* = 7.0, 7.0 Hz, 1H, CHAr), 1.83 (ddd, *J* = 13.0, 8.5, 3.5 Hz, 1H, CH), 1.54 (ddd, *J* = 13.0, 6.0, 3.5 Hz, 1H, CH), 1.39 (ddd, *J* = 13.0, 8.5, 3.5 Hz, 1H, CH), 1.23–1.13 (ddd, *J* = 13.0, 6.0, 3.5 Hz, 1H, CH), 1.04–0.95 (m, 2H, CH); ^{13}C NMR (100.6 MHz, $CDCl_3$) δ (rotamers) 157.0 (Ar), 156.7 (Ar), 132.0 (*ipso*-Ar), 45.6 (NCH_2), 45.2 (NCH_2), 35.9 (CH_2), 35.03 (SO_2Me), 35.01 (SO_2Me), 29.7 (CH_2), 24.6 (C), 23.4 (*C*HAr), 15.7 (CH_2); HRMS (ESI) *m/z* calcd for $C_{12}H_{17}N_3NaO_2S$ $(M + Na)^+$ 290.0934, found 290.0935 (–0.3 ppm error).

The structure was confirmed by X-Ray crystallography (CCDC 2219563):

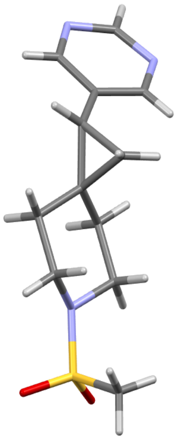

Lab book reference JRD_IX_55

5-Methanesulfonyl-1-(pyrimidin-5-yl)-5-azaspiro[2.3]hexane 88

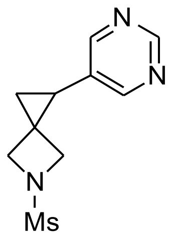

88

Using general procedure D, *N*-Boc aryl cyclopropane **28** (98 mg, 0.38 mmol, 1.0 eq.) was reacted with HCl in dioxane (7.50 mL of 4 M solution) then, Et_3N (182 mg, 251 µL, 1.80 mmol, 4.8 eq.) and MsCl (95 mg, 64 µL, 0.83 mmol, 2.2 eq.) in CH_2Cl_2 (6.3 mL) to give the crude product. Purification by flash column chromatography on silica with 8:2 EtOAc-acetone as eluent gave sulfonamide **88** (39 mg, 43%) as a colourless solid, mp 125–126 °C; R_F (8:2 EtOAc-acetone) 0.17; IR (ATR) 3014, 2931, 2880, 1560, 1417, 1327, 1149, 1040, 968, 765, 738, 521 cm^{-1}; ^{1}H NMR (400 MHz, $CDCl_3$) δ 9.08 (s, 1H, Ar), 8.40 (s, 2H, Ar), 4.18–4.09 (m, 2H, NCH), 4.01 (d, *J* = 8.0 Hz, 1H, NCH), 3.73 (d, *J* = 8.0 Hz, 1H, NCH), 2.87 (s, 3H, SO_2Me), 2.11 (dd, *J* = 9.5, 6.5 Hz, 1H, CHAr) 1.44 (dd, *J* = 9.5, 6.5 Hz, 1H, CH), 1.22 (dd, *J* = 6.5, 6.5 Hz, 1H, CH); ^{13}C NMR (100.6 MHz, $CDCl_3$) (rotamers) δ 157.1 (Ar), 155.4 (Ar), 132.0 (*ipso*-Ar), 57.36 (NCH_2), 57.33 (NCH_2), 57.30 (NCH_2), 54.09 (NCH_2), 54.05 (NCH_2), 54.01 (NCH_2), 37.1 (SO_2Me), 22.8 (C), 21.1 (*C*HAr), 16.4 (CH_2); HRMS (ESI) *m/z* calcd for $C_{10}H_{14}N_3O_2S$ $(M + H)^+$ 240.0801, found 240.0801 (+0.3 ppm error).

The structure was confirmed by X-Ray crystallography (CCDC 2219564):

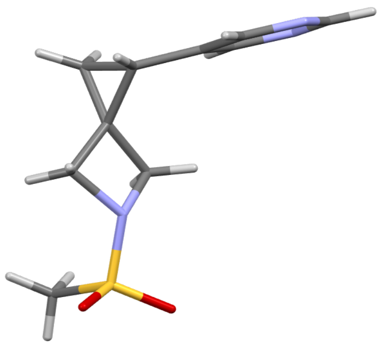

Lab book reference JRD_IX_51

(1*R,3*S**)-5-Methanesulfonyl-1-(pyrimidin-5-yl)-5-azaspiro[2.4]heptane 89**

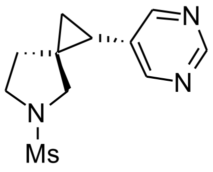

89

Using general procedure D, cyclopropyl-pyrimidine **32** (149 mg, 0.54 mmol, 1.0 eq.) and HCl (4.0 M in dioxane) (8 mL) then Et_3N (0.4 mL, 2.59 mmol, 4.8 eq.) and MsCl (92 μL, 1.19 mmol, 2.2 eq.) in CH_2Cl_2 (7 mL) gave the crude product. Purification by flash column chromatography on silica with 20:80 to 60:40 acetone-CH_2Cl_2 as eluent gave mesyl cyclopropyl-pyrimidine **89** (107 mg, 78%) as a crystalline white solid, mp 139–140 °C, R_F (2:8 acetone-CH_2Cl_2) 0.12; IR (ATR) 2984, 2931, 2869, 1556, 1416, 1325 (S=O), 1151 (S=O), 1052, 730, 563, 519 cm^{-1}; ^{1}H NMR (400 MHz, $CDCl_3$) δ 9.08 (s, 1H, Ar), 8.54 (s, 2H, Ar), 3.48–3.40 (m, 2H, NCH), 3.40–3.33 (m, 2H, NCH), 2.87 (s, 3H, SO_2Me), 2.18 (dd, *J* = 9.0, 6.0 Hz, 1H, CH), 1.75 (ddd, *J* = 13.0, 7.5, 6.0 Hz, 1H, CH), 1.59 (ddd, *J* = 13.0, 7.5, 6.0 Hz, 1H, CH), 1.35 (dd, *J* = 9.0, 6.0 Hz, 1H), 1.26 (dd, *J* = 6.0, 6.0 Hz, 1H, CH); ^{13}C NMR (100.6 MHz, $CDCl_3$) 157.1 (Ar), 156.6 (Ar), 132.0 (*ipso*-Ar), 55.9 (NCH_2), 47.8 (NCH_2), 35.4 (SO_2Me), 30.0 (C), 29.5 (CH_2), 22.5 (CH), 15.7 (CH_2); MS (ESI) *m/z* 276 (M + Na)$^+$; HRMS (ESI) *m/z* calcd for $C_{11}H_{15}N_3O_2S$ (M + Na)$^+$ 276.0777, found 276.0776 (+0.4 ppm error).

The structure was confirmed by X-Ray crystallography (CCDC 2219561):

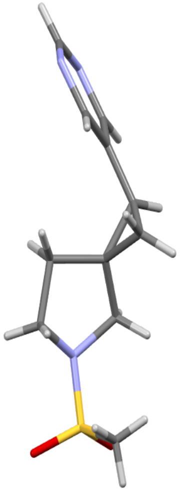

Lab Book Reference: SY-2-93

(1*R,3*R**)-5-Methanesulfonyl-1-(pyrimidin-5-yl)-5-azaspiro[2.4]heptane 90**

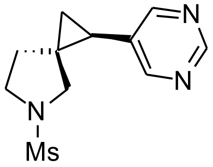

90

Using general procedure D, cyclopropyl-pyrimidine **37** (109 mg, 0.40 mmol, 1.0 eq.) and HCl (4.0 M in dioxane) (7 mL) then Et_3N (0.3 mL, 1.90 mmol, 4.8 eq.) and MsCl (69 μL, 0.6 mmol, 2.2 eq.) in CH_2Cl_2 (6 mL) gave the crude product. Purification by flash column chromatography on silica with 20:80 acetone-CH_2Cl_2 as eluent gave mesyl cyclopropyl-pyrimidine **90** (86 mg, 86%) as a crystalline off-white solid, mp 120–121 °C, R_F (2:8 acetone-CH_2Cl_2) 0.19; IR (ATR) 2988, 2930, 2875, 1556, 1417, 1327 (S=O), 1151 (S=O), 1055, 754, 729, 518 cm^{-1}; ^{1}H NMR (400 MHz, $CDCl_3$) δ 9.09 (s, 1H, Ar), 8.52 (s, 2H, Ar), 3.63–3.43 (m, 2H, NCH), 3.14 (d, J = 10.5 Hz, 1H, NCH), 2.89 (d, J = 10.5 Hz, 1H, NCH), 2.73 (s, 3H, SO_2Me), 2.15 (dd, J = 9.0, 6.0 Hz, 1H, CH), 2.11–2.06 (m, 2H, CH), 1.36 (dd, J = 9.0, 6.0 Hz, 1H, CH), 1.28 (dd, J = 6.0, 6.0 Hz, 1H, CH); ^{13}C NMR (100.6 MHz, $CDCl_3$) δ 157.2 (Ar), 156.6 (Ar), 131.9 (*ipso*-Ar), 50.6 (NCH_2), 47.9 (NCH_2), 35.5 (CH_2), 35.3 (SO_2Me), 30.2 (C), 22.8 (CH), 15.7 (CH_2); MS (ESI) *m/z* 276 (M + Na)$^+$; HRMS (ESI) *m/z* calcd for $C_{11}H_{15}N_3O_2S$ (M + Na)$^+$ 276.0777, found 276.0780 (–0.9 ppm error). The structure was confirmed by X-Ray crystallography (CCDC 2219562):

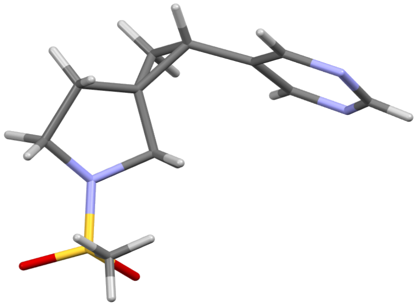

Lab Book Reference: SY-2-94

(1*R,3'*S**,5*S**)-8-Methanesulfonyl-3'-(pyrimidin-5-yl)-8-azaspiro[bicyclo[3.2.1]octane-3,1'-cyclopropane 91**

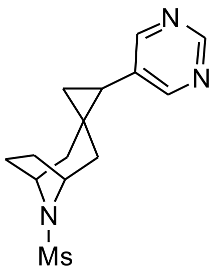

91

Using general procedure D, *N*-Boc aryl cyclopropane **43** (51 mg, 0.16 mmol, 1.0 eq.) was reacted with HCl in dioxane (3.24 mL of 4 M solution) then, Et_3N (79 mg, 108 µL, 0.78 mmol, 4.8 eq.) and MsCl (41 mg, 28 µL, 0.36 mmol, 2.2 eq.) in CH_2Cl_2 (2.7 mL) to give the crude product. Purification by flash column chromatography on silica with 9:1 EtOAc-acetone as eluent gave sulfonamide **91** (45 mg, 95%) as a colourless solid, mp 167–168 °C; R_F (9:1 EtOAc-acetone) 0.27; IR 2926, 1552, 1416, 1318, 1138, 1018, 980, 780, 766, 727, 609, 539, 500 cm^{-1}; ^{1}H NMR (400 MHz, DMSO-d_6) δ 8.99 (s, 1H, Ar), 8.66 (s, 2H, Ar), 4.22–4.16 (m, 1H, NCH), 4.16–4.10 (m, 1H, NCH), 2.92 (s, 3H, SO_2Me), 2.23 (dd, *J* = 13.0, 2.5 Hz, 1H, CH), 2.02–1.87 (m, 4H, CH), 1.72 (dd, *J* = 8.5, 6.0 Hz, 1H, CH), 1.49 (dd, *J* = 6.0, 6.0 Hz, 1H, CH), 1.41 (dd, *J* = 13.0, 2.5 Hz, 1H, CH), 1.24–1.11 (m, 2H, CH), 0.86 (dd, *J* = 13.0, 2.5 Hz, 1H, CH); ^{13}C NMR (100.6 MHz, DMSO-d_6) (rotamers) δ 156.3 (Ar), 156.0 (Ar), 132.6 (*ipso*-Ar), 56.9 (NCH), 56.8 (NCH), 56.5 (NCH), 56.4 (NCH), 42.5 (CH_2), 37.5 (CH_2), 27.6 (CH_2), 27.4 (CH_2), 20.1 (CH_2), 19.5 (C), 18.5 (*C*HAr); HRMS (ESI) *m/z* calcd for $C_{14}H_{19}N_3NaO_2S$ $(M + Na)^+$ 316.1090, found 316.1089 (+0.5 ppm error).

The structure was confirmed by X-Ray crystallography (CCDC 2219565):

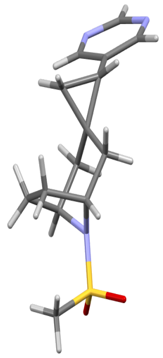

Lab book reference JRD_IX_66

(1*R,6*R**,7*R**)-3-Methanesulfonyl-7-(pyrimidin-5-yl)-3-azabicyclo[4.1.0] 92**

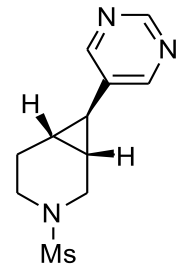

92

Using general procedure D, *N*-Boc aryl cyclopropane **47** (123 mg, 0.45 mmol, 1.0 eq.) was reacted with HCl in dioxane (8.90 mL of 4 M solution) then, Et_3N (217 mg, 299 µL, 2.14 mmol, 4.8 eq.) and MsCl (113 mg, 76 µL, 0.98 mmol, 2.2 eq.) in CH_2Cl_2 (7.4 mL) to give the crude product. Purification by flash column chromatography on silica with 8:2 EtOAc-acetone as eluent gave sulfonamide **92** (103 mg, 91%) as a colourless solid, mp 112–113 °C; R_F (8:2 EtOAc-acetone) 0.20; ^{1}H NMR (400 MHz, acetone-d_6) δ 8.90 (s, 1H, Ar), 8.51 (s, 2H, Ar), 3.77–3.68 (m, 1H, NCH), 3.44 (dd, J = 12.0, 5.0 Hz, 1H, NCH), 3.30 (dddd, J = 12.0, 6.0, 4.5, 1.5 Hz, 1H, NCH), 2.89–2.78 (m, 4H, CH, SO_2Me), 2.24–2.13 (m, 1H, CH), 2.00–1.89 (m, 2H, CH, CHAr), 1.74 (dddd, J = 9.5, 4.5, 4.5, 9.5 Hz, CH), 1.63–1.54 (m, 1H); ^{13}C NMR (100.6 MHz, acetone-d_6) δ 156.8 (Ar), 154.9 (Ar), 137.0 (*ipso*-Ar), 44.7 (NCH_2), 43.1 (NCH_2), 34.7 (SO_2Me), 24.0 (CH_2), 23.2 (*C*HAr), 22.4 (CH), 20.3 (CH); HRMS (ESI) *m/z* calcd for $C_{11}H_{15}N_3NaO_2S$ $(M + Na)^+$ 276.0777, found 276.0777 (+0.2 ppm error).

The structure was confirmed by X-Ray crystallography (CCDC 2219558) and contained three conformers in the unit cell:

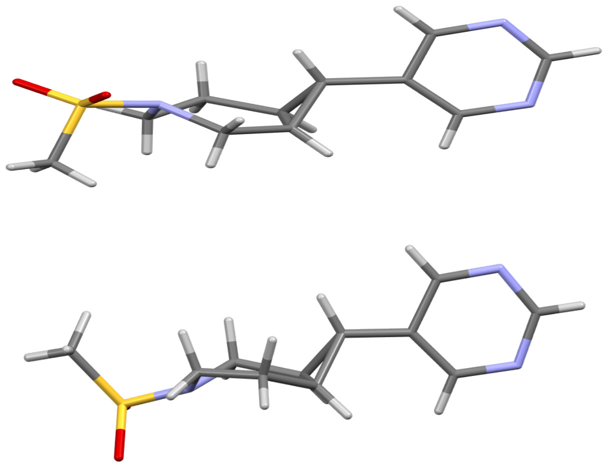

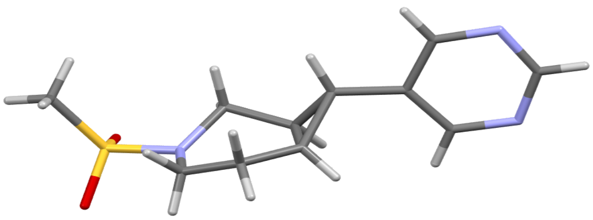

Lab book reference JRD_IX_10

(1*R,5*S**,6*R**)-3-Methanesulfonyl-6-(pyrimidin-5-yl)-3-azabicyclo[3.1.0]hexane 93**

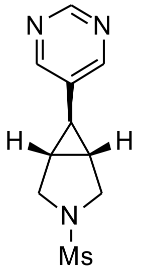

93

Using general procedure D, cyclopropyl-pyrimidine **63** (69 mg, 0.26 mmol, 1.0 eq.) and HCl (4.0 M in dioxane) (5 mL) then Et_3N (0.2 mL, 1.3 mmol, 4.8 eq.) and MsCl (45 μL, 0.6 mmol, 2.2 eq.) in CH_2Cl_2 (5 mL) gave the crude product. Purification by flash column chromatography on silica with 20:80 acetone-CH_2Cl_2 as eluent gave mesyl cyclopropyl-pyrimidine **93** (60 mg, 95%) as a crystalline off-white solid, mp 148–150 °C; R_F (2:8 acetone-CH_2Cl_2) 0.21; IR (ATR) 3044, 3009, 2974, 2936, 2875, 1557, 1445, 1424, 1401, 1324 (S=O), 1289, 1150 (S=O), 1129, 1082, 1017, 968, 915, 817, 750, 728 cm^{-1}; ^{1}H NMR (400 MHz, $CDCl_3$) δ 9.02 (s, 1H, Ar), 8.45 (s, 2H, Ar), 3.77 (d, *J* = 9.5 Hz, 2H, NCH), 3.47–3.42 (m, 2H, NCH), 2.87 (s, 3H, SO_2Me), 2.03 (t, *J* = 3.5 Hz, 1H, CH), 1.99–1.93 (m, 2H, CH); ^{13}C NMR (100.6 MHz, $CDCl_3$) 156.8 (Ar), 154.6 (Ar), 133.8 (*ipso*-Ar), 49.9 (NCH_2), 36.1 (SO_2Me), 26.3 (CH), 20.2 (*C*HAr); MS (ESI) *m/z* 262 (M + Na)$^+$; HRMS (ESI) *m/z* calcd for $C_{10}H_{13}N_3O_2S$ (M + Na)$^+$ 262.0621, found 262.0624 (–0.9 ppm error).

The structure was confirmed by X-Ray crystallography (CCDC 2219560):

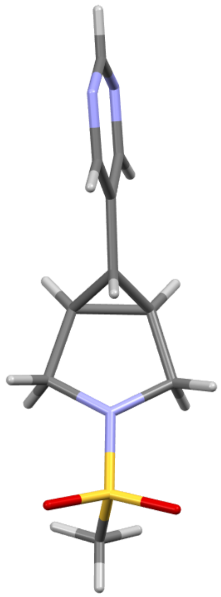

Lab Book Reference: SY-1-114

(1*R,6*R**,7*S**)-2-Methanesulfonyl-7-(pyrimidin-5-yl)-2-azabicyclo[4.1.0]heptane 94**

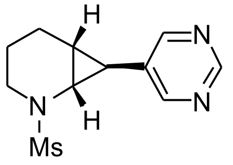

94

Using general procedure D, cyclopropyl-pyrimidine **69** (108 mg, 0.39 mmol, 1.0 eq.) and HCl (4.0 M in dioxane) (5 mL) then Et_3N (0.3 mL, 1.9 mmol, 4.8 eq.) and MsCl (67 μL, 0.9 mmol, 2.2 eq.) in CH_2Cl_2 (6 mL) gave the crude product. Purification by flash column chromatography on silica with 20:80 acetone-CH_2Cl_2 as eluent gave mesyl cyclopropyl-pyrimidine **94** (99 mg, 72%) as a crystalline white solid, mp 191–193 °C, R_F (2:8 acetone-CH_2Cl_2) 0.24; IR (ATR) 3006, 2928, 2872, 1558, 1424, 1333 (S=O), 1323, 1142 (S=O), 1042, 972, 941, 773, 524, 504 cm^{-1}; ^{1}H NMR (400 MHz, $CDCl_3$) δ 9.05 (s, 1H, Ar), 8.52 (s, 2H, Ar), 3.54 (ddd, *J* = 12.5, 4.0, 4.0 Hz, 1H, NCH), 3.09 (dd, *J* = 9.0, 3.0 Hz, 1H, NCH), 2.89 (s, 3H, SO_2Me), 2.89–2.82 (m, 1H, NCH), 2.12–2.03 (m, 1H, CH), 2.03–1.92 (m, 2H, CH), 1.86–1.74 (m, 2H, CH), 1.62–1.46 (m, 1H, CH); ^{13}C NMR (100.6 MHz, $CDCl_3$) δ 156.9 (Ar), 155.1 (Ar), 133.5 (*ipso*-Ar), 42.8 (NCH_2), 38.1 (NCH), 37.9 (SO_2Me), 24.5 (CH), 22.3 (CH_2), 20.7 (CH), 19.9 (CH_2);

MS (ESI) *m/z* 276 (M + Na)$^+$; HRMS (ESI) *m/z* calcd for $C_{11}H_{15}N_3O_2S$ (M + Na)$^+$ 276.0777, found 276.0779 (–0.6 ppm error).

The structure was confirmed by X-Ray crystallography (CCDC 2219559):

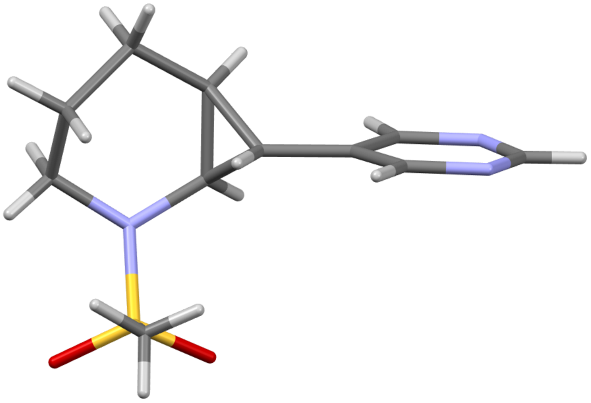

Lab Book Reference: SY-3-87

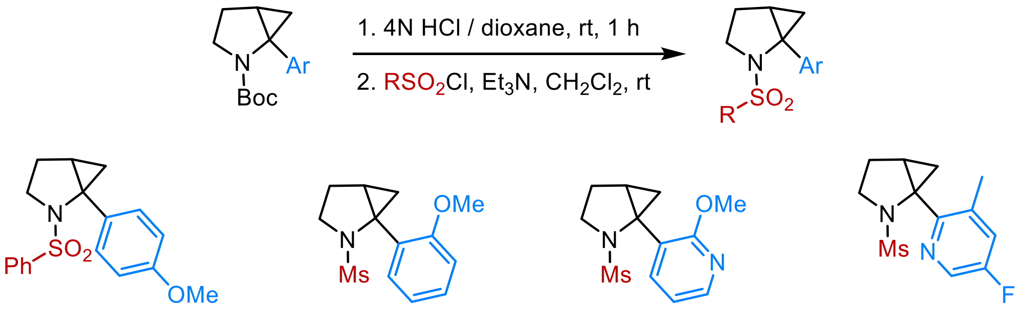

S34 93% **S35** 80% **S36** 55% **S37** 57%

Scheme S1. Additional examples of *N*-sulfonylations of arylated building blocks

2-(Benzenesulfonyl)-1-(4-methoxyphenyl)-2-azabicyclo[3.1.0]hexane S34

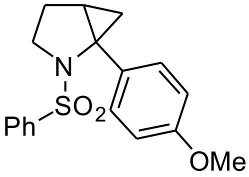

S34

HCl (3.0 mL of a 4 N solution in dioxane) was added dropwise to a stirred solution of arylated cyclopropane **12** (115 mg, 0.398 mmol, 1.0 eq.) at rt under Ar. The resulting solution was stirred at rt for 1 h. Then, the solvent was evaporated under reduced pressure to give the crude HCl salt. Et_3N (277 µL, 1.99 mmol, 5.0 eq.) was added dropwise to a stirred solution the HCl salt in CH_2Cl_2 (6 mL) at rt under Ar. The resulting solution was stirred at rt for 10 min and $PhSO_2Cl$ (112 µL, 0.876 mmol, 2.2 eq.) was added dropwise. The resulting solution was stirred at rt for 18 h. The mixture was poured into water (10 mL) and extracted with CH_2Cl_2 (3 × 10 mL). The combined organics were dried ($MgSO_4$) and evaporated under reduced pressure to give the crude product. Purification by flash column chromatography on silica with 80:20 hexane-EtOAc as eluent gave phenylsulfonamide **S34** (122 mg, 93%) as a yellow oil, R_F (1:1 hexane-EtOAc) 0.52; IR (ATR) 1516, 1349, 1245, 1165 cm^{-1}; ^{1}H NMR (400 MHz, $CDCl_3$) δ 7.75 (d, *J* = 8.0 Hz, 2H, Ar), 7.63–7.55 (m, 1H, Ar), 7.49 (dd, *J* = 8.0, 8.0 Hz, 2H, Ar), 7.36 (d, *J* = 9.0 Hz, 2H, Ar), 6.85 (d, *J* = 9.0 Hz, 2H, Ar), 3.80 (s, 4H, OMe and NCH), 2.87 (ddd, *J* = 10.5, 9.5, 8.0 Hz, 1H, NCH), 2.34–2.25 (m, 1H, CH), 1.92–1.87 (m, 1H, CH), 1.38–1.33 (m, 1H, CH), 1.21 (dd, *J* = 9.0, 6.0 Hz, 1H, CH), 0.40 (dd, *J* = 6.0 Hz, 1H, CH); ^{13}C NMR (100.6 MHz, $CDCl_3$) δ 158.8 (*ipso*-Ar), 136.7 (*ipso*-Ar), 132.8 (Ar), 131.4 (*ipso*-Ar), 129.5 (Ar), 128.7 (Ar), 128.5 (Ar), 113.6 (Ar), 55.4 (OMe), 49.7 (N*C*Ar), 47.9 (NCH_2), 27.5 (CH), 25.2 (CH_2), 11.7 (CH_2); HRMS (ESI) *m/z* calcd for $C_{18}H_{19}NO_3S$ [(M + Na)+, 100] 352.0978, found 352.0980 (–0.7 ppm error).

Lab Book Reference: HFK5-043

2-Methanesulfonyl-1-(2-methoxyphenyl)-2-azabicyclo[3.1.0]hexane S35

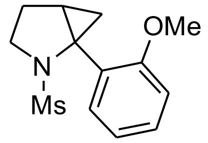

S35

HCl (3.0 mL of a 4 N solution in dioxane) was added dropwise to a stirred solution of arylated cyclopropane **14** (53 mg, 0.18 mmol, 1.0 eq.) at rt under Ar. The resulting solution was stirred at rt for 1 h. Then, the solvent was evaporated under reduced pressure to give the crude HCl salt. Et_3N (128 µL, 0.916 mmol, 5.0 eq.) was added dropwise to a stirred solution the HCl salt in CH_2Cl_2 (3 mL) at rt under Ar. The resulting solution was stirred at rt for 10 min and MsCl (31 µL, 0.40 mmol, 2.2 eq.) as added dropwise. The resulting solution was stirred at rt for 18 h. The mixture was poured into water (10 mL) and extracted with CH_2Cl_2 (3 × 10 mL). The combined organics were dried (MgSO4) and evaporated under reduced pressure to give the crude product. Purification by flash column chromatography on silica with 70:30 hexane-EtOAc as eluent gave methanesulfonamide **S35** (39 mg, 80%) as a white solid, mp 86–88 °C; R_F (1:1 hexane-EtOAc 0.31); IR (ATR) 1331, 1149, 752, 517 cm^{-1}; ^1H NMR (400 MHz, $CDCl_3$) δ 7.42 (dd, J = 7.5, 2.0 Hz, 1H, Ar), 7.30 (ddd, J = 8.0, 7.5, 2.0 Hz, 1H, Ar), 6.93 (ddd, J = 7.5, 7.5, 1.0 Hz, 1H, Ar), 6.88 (dd, J = 8.0, 1.0 Hz, 1H, Ar), 3.88 (s, 3H, OMe), 3.75 (ddd, J = 9.0, 9.0, 3.0 Hz, 1H, NCH), 3.31–3.25 (m, 1H, NCH), 2.53 (s, 3H, SO_2Me), 2.46–2.37 (m, 1H, CH), 2.11–2.05 (m, 1H, CH), 1.66–1.61 (m, 1H, CH), 1.35–1.34 (m, 1H, CH), 1.33–1.32 (m, 1H, CH); ^{13}C NMR (100.6 MHz, $CDCl_3$) δ 159.3 (*ipso*-Ar), 132.6 (Ar), 129.7 (Ar), 126.2 (*ipso*-Ar), 120.4 (Ar), 110.7 (Ar), 55.7 (OMe), 48.5 (NCH_2), 47.0 (N*C*Ar), 38.8 (SO_2Me), 26.3 (CH), 26.2 (CH_2), 16.4 (CH_2); HRMS (ESI) *m/z* calcd for $C_{13}H_{17}NO_3S$ [$(M + Na)^+$, 100] 290.0821, found 290.0820 (+0.5 ppm error).

Lab Book Reference: HFK5-045

2-Methanesulfonyl-1-(2-methoxypyridin-3-yl)-2-azabicyclo[3.1.0]hexane S36

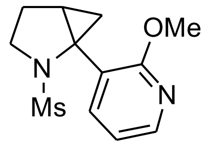

S36

HCl (6 mL of a 4 N solution in dioxane) was added to *N*-Boc aryl cyclopropane **19** (91 mg, 0.31 mmol, 1.0 eq.) at rt under Ar. The resulting solution was stirred at rt for 1 h. Then, the solvent was evaporated under reduced pressure to give the crude HCl salt as a white solid. Et_3N (0.21 mL, 1.5 mmol, 4.8 eq.) was added dropwise to a stirred solution of HCl salt in CH_2Cl_2 (5 mL) at rt under Ar. The resulting solution was stirred at rt for 10 min and MsCl (52 µL, 0.68 mmol, 2.2 eq.) was added dropwise. The resulting solution was stirred at rt for 18 h. The mixture was poured into H_2O (10 mL) and extracted with CH_2Cl_2 (3 × 7 mL). The combined organics were dried ($MgSO_4$) and evaporated under reduced pressure to give the crude product as a yellow oil. Purification by flash column chromatography on silica with 99:1 CH_2Cl_2-Et_3N as eluent gave methanesulfonamide **S36** (46 mg, 55%) as a white solid, mp 121–123 °C; R_F (99:1 CH_2Cl_2-Et_3N) 0.35; IR (ATR) 2951, 1582, 1464, 1332 cm^{-1}; ^1H NMR (400 MHz, $CDCl_3$) δ 8.10 (dd, *J* = 5.0, 2.0 Hz, 1H, Ar), 7.66 (d, *J* = 7.5, 2.0 Hz, 1H, Ar), 6.86 (d, *J* = 7.5, 5.0 Hz, 1H, Ar), 3.98 (s, 1H, SO_2Me), 3.76 (ddd, *J* = 10.0, 9.5, 3.0 Hz, 1H, NCH), 3.26 (ddd, *J* = 10.0, 9.0, 8.0 Hz, 1H, NCH), 2.62 (s, 3H, Me), 2.42 (dddd, *J* = 13.0, 9.0, 9.0, 6.0 Hz, 1H, NCH_2CH), 2.08 (ddd, *J* = 13.0, 8.0, 3.0 Hz, 1H, NCH_2CH), 1.66–1.60 (m, 1H, NCCH), 1.38–1.29 (m, 2H, NCCH); ^{13}C NMR (100.6 MHz, $CDCl_3$) δ 163.1 (*ipso*-Ar), 146.6 (Ar), 142.8 (Ar), 140.8 (Ar), 120.9 (*ipso*-Ar), 116.7 (Ar), 53.7 (OMe), 48.8 (NCH_2), 46.6 (NC), 38.5 (Me), 26.3 (NC*C*H), 26.2 (NCH_2CH_2), 16.7 ($NCCH_2$); HRMS *m/z* calcd for $C_{12}H_{16}N_2O_3S$ $(M + Na)^+$ 291.0774, found 291.0767 (+2.4 ppm error).

Lab book reference RA 1-037

1-(5-Fluoro-3-methylpyridin-2-yl)-2-methanesulfonyl-2-azabicyclo[3.1.0]hexane S37

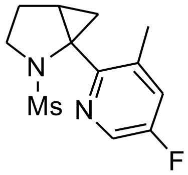

S37

HCl (6 mL of a 4 N solution in dioxane) was added to *N*-Boc aryl cyclopropane **17** (84 mg, 0.29 mmol, 1.0 eq.) at rt under Ar. The resulting solution was stirred at rt for 1 h. Then, the solvent was evaporated under reduced pressure to give the crude HCl salt as a white solid. Et_3N (0.19 mL, 1.38 mmol, 4.8 eq.) was added dropwise to a stirred solution of HCl salt in CH_2Cl_2 (5 mL) at rt under Ar. The resulting solution was stirred at rt for 10 min and MsCl (50 µL, 0.64 mmol, 2.2 eq.) was added dropwise. The resulting solution was stirred at rt for 18 h. The mixture was poured into H_2O (10 mL) and extracted with CH_2Cl_2 (3 × 7 mL). The combined organics were dried ($MgSO_4$) and evaporated under reduced pressure to give the crude product as a yellow oil. Purification by flash column chromatography on silica with 89:10:1 CH_2Cl_2-Et_2O-Et_3N as eluent methanesulfonamide **S37** (45 mg, 57%) as white solid, mp 120–122 °C; R_F (69:30:1 CH_2Cl_2-Et_2O-Et_3N) 0.40; IR (ATR) 1473, 1332, 1206, 1155 cm^{-1}; ^1H NMR (400 MHz, $CDCl_3$) δ 8.16 (d, *J* = 3.0 Hz, 1H, Ar), 7.18 (dd, *J* = 9.0, 3.0 Hz, 1H, Ar), 3.79 (dd, *J* = 10.0, 10.0 Hz, 1H, NCH), 3.00 (ddd, *J* = 10.0, 10.0, 8.0 Hz, 1H, NCH), 2.84 (d, *J* = 5.5 Hz, 3H, SO_2Me), 2.47 (s, 3H, CMe), 2.45–2.34 (m, 1H, NCH_2C*H*), 2.15 (dd, *J* = 13.0, 8.0 Hz, 1H, NCH_2C*H*), 1.84–1.72 (m, 1H, NCCH), 1.75–1.64 (m, 1H, NCCH), 1.31 (dd, *J* = 5.5, 5.5 Hz, 1H, NCCH); ^{13}C NMR (100.6 MHz, $CDCl_3$) δ 159.1 (d, *J* = 256.5 Hz, CF), 150.6 (d, *J* = 4.0 Hz, C=N), 136.6 (d, *J* = 5.9 Hz, *C*Me), 133.9 (d, *J* = 23.1 Hz, C=CF), 125.0 (d, *J* = 18.1 Hz, C=CF), 50.8 (NC), 47.2 (NCH_2), 34.8 (SMe), 25.9 (NC*C*H), 25.7 (NCH_2CH_2), 18.9 (C*Me*), 12.3 ($NCCH_2$); HRMS *m/z* calcd for $C_{12}H_{15}FN_2O_2S$ $(M + Na)^+$ 293.0730, found 293.0726 (+1.6 ppm error).

Lab book reference RA 1-038

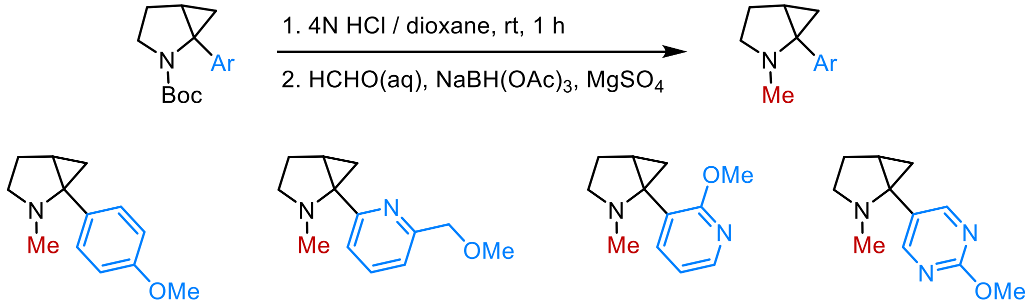

S38 71% **S39** 44% **S40** 45% **S41** 39%

Scheme S2 *N*-Methylations of arylated building blocks

1-(4-Methoxyphenyl)-2-methyl-2-azabicyclo[3.1.0]hexane S38

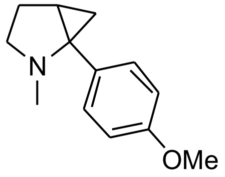

S38

HCl (5 mL of a 4 N solution in dioxane) was added to *N*-Boc aryl cyclopropane **12** (50 mg, 0.17 mmol, 1.0 eq.) at rt under Ar. The resulting solution was stirred at rt for 1 h. Then, the solvent was evaporated under reduced pressure to give the crude HCl salt as a white solid. 37% aqueous formaldehyde (48.3 µL, 1.7 mmol, 10.0 eq.) was added dropwise to a stirred suspension of the crude HCl salt, $NaBH(OAc)_3$ (106 mg, 0.51 mmol, 3.0 eq.) and $MgSO_4$ (49 mg, 0.41 mmol, 2.4 eq.) in CH_2Cl_2 (4 mL) and AcOH (1 mL) at rt under Ar and stirred at rt for 1 h. Then, CH_2Cl_2 (5 mL) and $NaHCO_{3(aq)}$ (15 mL) were added. The two layers were separated, and the aqueous layer was extracted with CH_2Cl_2 (3 × 5 mL). The combined organics were washed with brine (5 mL), dried ($MgSO_4$) and evaporated under reduced pressure to give *N*-methyl cyclopropane **S38** (24 mg, 71%) as a yellow oil, R_F (7:3 hexane-Et_2O) 0.23; IR (ATR) 2937, 2556, 1516, 1247 cm^{-1}; ^1H NMR (400 MHz, $CDCl_3$) δ 7.35 (d, *J* = 8.5 Hz, 2H, Ar), 6.84 (d, *J* = 8.5 Hz, 2H, Ar), 3.77 (s, 3H, OMe), 3.28 (ddd, *J* = 10.5, 8.0, 8.0 Hz, 1H, NCH_2CH_2), 2.30–2.21 (m, 4H, NMe, CH), 2.20–2.09 (m, 1H, NCH), 1.89 (dd, *J* = 12.0, 7.0 Hz, 1H, CH), 1.56–1.46 (m, 1H, NCCH), 1.11 (dd, *J* = 6.0, 4.5 Hz, 1H, NCCH), 0.83 (dd, *J* = 8.5, 6.0 Hz, 1H, NCCH); ^{13}C NMR (100.6 MHz, $CDCl_3$) δ 175.8 (*ipso*-Ar), 159.0 (*ipso*-Ar), 130.3 (Ar), 113.8 (Ar), 55.4 (OMe), 53.9 (NC), 51.6 (NCH_2), 37.7 (NMe), 26.53 (NCH_2CH_2), 25.4 (NC*C*H), 6.8 (NCCH_2); HRMS *m/z* calcd for $C_{13}H_{17}NO$ $(M + H)^+$ 204.1383, found 204.1381 (+1.1 ppm error).

Lab book reference RA 1-028

1-[6-(Methoxymethyl)pyridin-2-yl]-2-methyl-2-azabicyclo[3.1.0]hexane S39

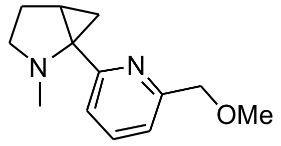

S39

HCl (7 mL of a 4 N solution in dioxane) was added to *N*-Boc aryl cyclopropane **18** (85 mg, 0.28 mmol, 1.0 eq.) at rt under Ar. The resulting solution was stirred at rt for 1 h. Then, the solvent was evaporated under reduced pressure to give the crude HCl salt as white solid. 37% aqueous formaldehyde (80 µL, 2.8 mmol, 10.0 eq.) was added dropwise to a stirred solution of the crude HCl salt, $NaBH(OAc)_3$ (178 mg, 0.84 mmol, 3.0 eq.) and $MgSO_4$ (81 mg, 0.67 mmol, 2.4 eq.) in CH_2Cl_2 (1.8 mL) and AcOH (0.45 mL) at rt under Ar and stirred at rt for 1 h. Then, CH_2Cl_2 (5 mL) and $NaHCO_{3(aq)}$ (15 mL) were added. The two layers were separated, and the aqueous layer was extracted with CH_2Cl_2 (3 × 7 mL). The combined organic layers were washed with brine (5 mL), dried ($MgSO_4$) and evaporated under reduced pressure giving the crude product as a yellow oil. Purification by flash column chromatography on silica with 69:30:1 CH_2Cl_2-Et_2O-Et_3N as eluent gave *N*-methyl cyclopropane **S39** (27 mg, 44%) as a yellow oil, R_F (99:1 CH_2Cl_2-Et_3N) 0.34; IR (ATR) 2932, 2866, 1586, 1455, 1113 cm^{-1}; ^1H NMR (400 MHz, $CDCl_3$) δ 7.61 (dd, *J* = 7.5, 7.5 Hz, 1H, Ar), 7.50 (dd, *J* = 7.5, 1.0 Hz, 1H, Ar), 7.16 (dd, *J* = 7.5, 1.0 Hz, 1H, Ar), 4.49 (d, *J* = 12.0 Hz, 1H, OCH), 4.48 (d, *J* = 12.0 Hz, 1H, OCH), 3.43 (s, 3H, OMe), 3.24–3.11 (m, 1H, NCH), 2.31 (s, 3H, NMe), 2.14 (ddd, *J* = 11.0, 9.0, 7.0 Hz, 1H, NCH_2CH), 2.10–2.01 (m, 1H, NCH), 1.92–1.81 (m, 1H, NCH_2CH), 1.66 (ddd, *J* = 9.0, 8.5, 5.0 Hz, 1H, NCCH), 1.55 (dd, *J* = 8.5, 5.0 Hz, 1H, NCCH), 1.19 (dd, *J* = 5.0, 5.0 Hz, 1H, NCCH); ^{13}C NMR (100.6 MHz, $CDCl_3$) δ 160.9 (*ipso*-Ar), 157.7 (*ipso*-Ar), 136.5 (Ar), 119.1 (Ar), 117.8 (Ar), 76.0 (OCH_2), 58.8 (OMe), 55.4 (NC), 53.2 (NCH_2), 39.3 (NCH_3), 30.4 (NC*C*H), 26.6 (NCH_2CH_2), 8.8 ($NCCH_2$); HRMS *m/z* calcd for $C_{13}H_{18}N_2O$ $(M + Na)^+$ 241.1311, found 241.1312 (–0.2 ppm error).

Lab book reference RA 1-030

1-(2-Methoxypyridin-3-yl)-2-methyl-2-azabicyclo[3.1.0]hexane S40

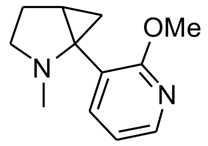

S40

HCl (7 mL of a 4 N solution in dioxane) was added to *N*-Boc aryl cyclopropane **19** (80 mg, 0.28 mmol, 1.0 eq.) at rt under Ar. The resulting solution was stirred at rt for 1 h. Then, the solvent was evaporated under reduced pressure to give the crude HCl salt as white solid. 37% aqueous formaldehyde (80 µL, 2.8 mmol, 10.0 eq.) was added dropwise to a stirred solution of the crude HCl salt, $NaBH(OAc)_3$ (178 mg, 0.84 mmol, 3.0 eq.) and $MgSO_4$ (81 mg, 0.67 mmol, 2.4 eq.) in CH_2Cl_2 (1.8 mL) and AcOH (0.45 mL) at rt under Ar and stirred at rt for 1 h. Then, CH_2Cl_2 (5 mL) and $NaHCO_{3(aq)}$ (15 mL) were added. The two layers were separated, and the aqueous layer was extracted with CH_2Cl_2 (3 × 7 mL). The combined organic layers were washed with brine (5 mL), dried ($MgSO_4$) and evaporated under reduced pressure giving the crude product as a yellow oil. Purification by flash column chromatography on silica with 89:10:1 CH_2Cl_2-Et_2O-Et_3N as eluent gave *N*-methyl cyclopropane **S40** (35 mg, 45%) as a yellow oil, R_F (89:10:1 CH_2Cl_2-Et_2O-Et_3N) 0.29; IR (ATR) 2937, 1578, 1460, 1403 cm^{-1}; ^1H NMR (400 MHz, $CDCl_3$) δ 8.05 (dd, *J* = 5.0, 2.0 Hz, 1H, Ar), 7.52 (dd, *J* = 7.0, 2.0 Hz, 1H, Ar), 6.80 (dd, *J* = 7.0, 5.0 Hz, 1H, Ar), 3.93 (s, 3H, Me), 3.09 (ddd, *J* = 10.0, 8.0, 8.0 Hz, 1H, NCH), 2.31–2.18 (m, 1H, NCH), 2.16 (s, 3H, Me), 2.00–1.92 (m, 1H, NCH_2CH), 1.89–1.81 (m, 1H, NCH_2CH), 1.52 (ddd, *J* = 8.5, 4.5, 4.5 Hz, 1H, NCCH), 1.07 (dd, *J* = 5.5, 4.5 Hz, 1H, $NCCH_2$), 0.52 (dd, *J* = 8.5, 5.5 Hz, 1H, $NCCH_2$); ^{13}C NMR (100.6 MHz, $CDCl_3$) δ 163.4 (*ipso*-Ar), 145.6 (Ar), 140.8 (Ar), 123.0 (*ipso*-Ar), 116.4 (Ar), 53.8 (Me), 51.9 (NCH_2), 50.5 (NC), 38.4 (Me), 26.8 (NCH_2CH_2), 23.7 (NCCH), 6.2 ($NCCH_2$); HRMS *m/z* calcd for $C_{12}H_{16}N_2O$ $(M + H)^+$ 205.1335, found 205.1335 (+0.3 ppm error).

Lab book reference RA 1-031/RA 1-063

1-(2-Methoxypyrimidin-5-yl)-2-methyl-2-azabicyclo[3.1.0]hexane S41

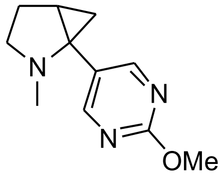

S41

HCl (6 mL of a 4 N solution in dioxane) was added to *N*-Boc aryl cyclopropane **16** (65 mg, 0.22 mmol, 1.0 eq.) at rt under Ar. The resulting solution was stirred at rt for 1 h. Then, the solvent was evaporated under reduced pressure to give the crude HCl salt as a white solid. 37% aqueous formaldehyde (64 µL, 2.2 mmol, 10 eq.) was added dropwise to a stirred solution of the crude HCl salt, $NaBH(OAc)_3$ (140 mg, 0.66 mmol, 3.0 eq.) and $MgSO_4$ (64 mg, 0.54 mmol, 2.4 eq.) in CH_2Cl_2 (1.8 mL) and AcOH (0.45 mL) at rt under Ar and stirred at rt for 1 h. Then, CH_2Cl_2 (5 mL) and $NaHCO_{3(aq)}$ (15 mL) were added. The two layers were separated, and the aqueous layer was extracted with CH_2Cl_2 (3 × 7 mL). The combined organic layers were washed with brine (5 mL), dried ($MgSO_4$) and evaporated under reduced pressure giving the crude product as a yellow oil. Purification by flash column chromatography on silica with 89:10:1 CH_2Cl_2-Et_2O-Et_3N as eluent gave *N*-methyl cyclopropane **S41** (29 mg, 39%) as a yellow oil, R_F (89:10:1 CH_2Cl_2-Et_2O-Et_3N) 0.27; IR (ATR) 2937, 1597, 1471, 1410 cm^{-1}; ^{1}H NMR (400 MHz, $CDCl_3$) δ 8.46 (d, *J* = 1.0 Hz, 2H, Ar), 3.96 (d, *J* = 1.0 Hz, 3H, OMe), 3.09 (ddd, *J* = 9.5, 8.0, 8.0 Hz, 1H, NCH), 2.21–2.07 (m, 1H, NCH), 2.14 (s, 3H, NMe) 2.02 (dd, *J* = 11.0, 10.0, 7.0, 1.0 Hz, 1H, NCH_2CH), 1.87 (dd, *J* = 12.0, 7.0 Hz, 1H, NCH_2CH), 1.43–1.36 (m, 1H, NCCH), 1.17–1.04 (m, 1H, NCCH), 0.80 (dd, *J* = 8.5, 6.0 Hz, 1H, NCCH); ^{13}C NMR (100.6 MHz, $CDCl_3$) δ 164.8 (*ipso*-Ar), 159.1 (Ar), 128.0 (*ipso*-Ar), 54.9 (OMe), 51.7 (NCH_2), 49.8 (NC), 38.0 (NMe), 26.7 (NCH_2CH_2), 26.0 (NCCH), 5.1 ($NCCH_2$); HRMS *m/z* calcd for $C_{11}H_{15}N_3O$ $(M + Na)^+$ 228.1107, found 228.1107 (+0.2 ppm error).

Lab book reference RA 1-032/RA 1-066

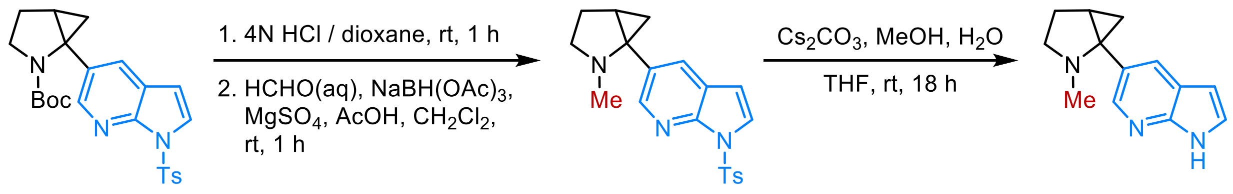

Scheme S3

2-Methyl-1-[1-(4-methylbenzenesulfonyl)-1H-pyrrolo[2,3-b]pyridin-5-yl]-2-azabicyclo[3.1.0]hexane S42

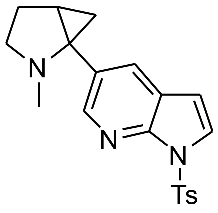

S42

HCl (5 mL of a 4 N solution in dioxane) was added to *N*-Boc aryl cyclopropane **20** (140 mg, 0.31 mmol, 1.0 eq.) at rt under Ar. The resulting solution was stirred at rt for 1 h. Then, the solvent was evaporated under reduced pressure to give the crude HCl salt as a white solid. 37% aqueous formaldehyde (0.1 mL, 3.1 mmol, 10 eq.) was added dropwise to a stirred solution of the crude HCl salt, $NaBH(OAc)_3$ (197 mg, 0.93 mmol, 3.0 eq.) and $MgSO_4$ (90 mg, 0.74 mmol, 2.4 eq.) in CH_2Cl_2 (1.8 mL) and AcOH (0.45 mL) at rt under Ar and stirred at rt for 1 h. Then, CH_2Cl_2 (5 mL) and $NaHCO_{3(aq)}$ (15 mL) was added. The two layers were separated, and the aqueous layer was extracted with CH_2Cl_2 (3 × 7 mL). The combined organic layers were washed with brine (5 mL), dried (MgSO4) and evaporated under reduced pressure giving the crude product as a yellow oil. Purification by flash column chromatography on silica with 89:10:1 CH_2Cl_2-Et_2O-Et_3N as eluent gave *N*-methyl cyclopropane **S42** (90 mg, 79%) as a yellow oil, R_F (89:10:1 CH_2Cl_2-Et_2O-Et_3N) 0.18; IR (ATR) 2935, 1370, 1152, 668 cm^{-1}; ^{1}H NMR (400 MHz, $CDCl_3$) δ 8.39 (d, *J* = 2.0 Hz, 1H, Ar), 8.04 (d, *J* = 8.5 Hz, 2H, Ar), 7.86 (dd, *J* = 2.0, 0.5 Hz, 1H, Ar), 7.65 (d, *J* = 4.0 Hz, 1H, Ar), 7.22 (d, *J* = 8.5 Hz, 1H, Ar), 6.50 (dd, *J* = 4.0, 0.5 Hz, 1H, Ar), 3.10 (dd, *J* = 9.5, 7.5 Hz, 1H, NCH), 2.32 (s, 3H, OMe), 2.19–2.09 (m, 1H, NCH), 2.12 (s, 3H, Me) 2.04 (dddd, *J* = 12.0, 9.5, 6.5, 1.0 Hz, 1H, NCH_2CH), 1.87 (dd, *J* = 12.0, 2.0 Hz, 1H, NCH_2CH), 1.45–1.33 (m, 1H, NCCH), 1.13 (dd, *J* = 5.5, 5.5 Hz, 1H, NCCH), 0.86 (dd, *J* = 8.5, 5.5 Hz, 1H, NCCH); ^{13}C NMR (100.6 MHz, $CDCl_3$) δ 146.3 (Ar), 145.5 (Ar), 145.1 (Ar), 135.6 (*ipso*-Ar), 132.5 (*ipso*-Ar), 129.7 (Ar), 129.4 (Ar), 128.1 (Ar), 126.7 (Ar), 122.6 (*ipso*-Ar), 105.2 (Ar), 52.3 (NCH_2), 52.0 (NC), 38.1 (Me), 26.8 (NCH_2CH_2), 26.5

(NC*C*H), 21.7 (OMe), 5.8 (NC*C*H_2); HRMS *m/z* calcd for $C_{20}H_{21}N_3O_2S$ $(M + Na)^+$ 390.1247, found 390.1239 (+2.0 ppm error).

Lab book reference RA 1-069

2-Methyl-1-{1H-pyrrolo[2,3-b]pyridin-5-yl}-2-azabicyclo[3.1.0]hexane S43

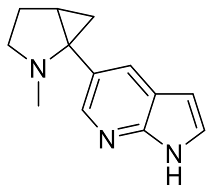

S43

Tosyl protected *N*-methyl cyclopropane **S41** (90 mg, 0.25 mmol, 1.0 eq.) was dissolved in a mixture of THF (6 mL), MeOH (2 mL) and H_2O (4.5 µL, 0.25 mmol, 1.0 eq.) at rt under Ar. Cs_2CO_3 (245 mg, 0.75 mmol, 3.0 eq.) was added to the solution. The resulting mixture was stirred at rt for 18 h. The solvent was evaporated under reduced pressure. To the residue was added water (5 mL) and the mixture was stirred at rt for 10 min. The mixture was extracted with EtOAc (3 × 7 mL). The combined organics were dried ($MgSO_4$) and evaporated under reduced pressure to give the crude product. Purification by flash chromatography on silica with 89:10:1 CH_2Cl_2-Et_2O-Et_3N as eluent gave *N*-methyl cyclopropane **S43** (25 mg, 60%) as a yellow oil; R_F (89:10:1 CH_2Cl_2-Et_2O-Et_3N) 0.26; IR (ATR) 2935, 2865, 1397, 1341 cm^{-1}; ^1H NMR (400 MHz, $CDCl_3$) δ 10.89 (s, 1H, NH), 8.43 (d, *J* = 2.0 Hz, 1H, Ar), 8.04 (d, *J* = 2.0 Hz, 1H, Ar), 7.35 (d, *J* = 3.5 Hz, 1H, Ar), 6.45 (d, *J* = 3.5 Hz, 1H, Ar), 3.33–3.23 (m, 1H, NCH), 2.38–2.29 (m, 1H, NCH), 2.27 (s, 3H, Me), 2.22–2.12 (m, 1H, NCH_2CH), 1.96 (dd, *J* = 12.0, 7.0 Hz, 1H, NCH_2CH), 1.55 (ddd, *J* = 8.5, 4.5, 4.5 Hz, 1H, NCCH), 1.20 (dd, *J* = 6.0, 4.5 Hz, 1H, NCCH), 0.94 (dd, *J* = 8.5, 6.0 Hz, 1H, NCCH); ^{13}C NMR (100.6 MHz, $CDCl_3$) δ 148.2 (*ipso*-Ar), 144.2 (Ar), 129.9 (Ar), 127.4 (*ipso*-Ar), 125.8 (Ar), 120.2 (*ipso*-Ar), 100.7 (Ar), 53.1 (NC), 51.9 (NCH_2), 37.9 (Me), 26.7 (NCH_2CH_2), 25.2 (NC*C*H), 8.7 (NC*CH2*); HRMS *m/z* calcd for $C_{13}H_{15}N_3$ $(M + H)^+$ 214.1339, found 214.1338 (+0.5 ppm error).

Lab book reference RA 1-080

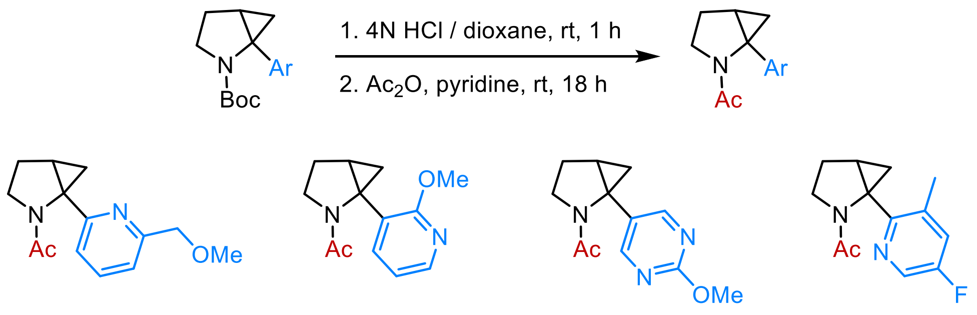

S44 58% **S45** 46% **S46** 45% **S47** 60%

Scheme S4

1-{1-[6-(Methoxymethyl)pyridin-2-yl]-2-azabicyclo[3.1.0]hexan-2-yl}ethan-1-one S44

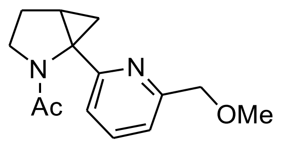

S44

HCl (6 mL of 4 N solution in dioxane) was added to *N*-Boc aryl cyclopropane **18** (86 mg, 0.28 mmol, 1.0 eq.) at rt under Ar. The resulting solution was stirred at rt for 1 h. Then, the solvent was evaporated under reduced pressure to give the crude HCl salt as a white solid. Ac_2O (0.16 mL, 1.7 mmol, 6.0 eq.) was added dropwise to a stirred solution of the crude HCl salt in pyridine (3 mL) at rt under Ar. The resulting solution was stirred at rt for 18 h. Then, the solvent was evaporated under reduced pressure to give a clear oil. CH_2Cl_2 (10 mL) and H_2O (15 mL) were added, and the two layers were separated. The aqueous layer was extracted with CH_2Cl_2 (3 × 10 mL) and the combined organics were dried ($MgSO_4$) and evaporated under reduced pressure to give the crude product as a yellow oil. Purification by flash column chromatography on silica with 79:20:1 CH_2Cl_2-Et_2O-Et_3N as eluent gave *N*-aryl cyclopropane **S44** (40 mg, 58%) as white solid, mp 54–56 °C; R_F (79:20:1 CH_2Cl_2-Et_2O-Et_3N) 0.41; IR (ATR) 2928, 2878, 1655 (C=O), 1457 cm^{-1}; ^{1}H NMR (400 MHz, $CDCl_3$) (90:10 mixture of rotamers) δ 7.62 (dd, *J* = 8.0, 8.0 Hz, 0.9H, Ar), 7.55 (dd, *J* = 8.0, 8.0 Hz, 0.1H, Ar), 7.28 (dd, *J* = 8.0, 1.0 Hz, 0.9H, Ar), 7.20 (dd, *J* = 8.0, 1.0 Hz, 0.9H, Ar), 7.13 (dd, *J* = 8.0, 1.0 Hz, 0.2H, Ar), 4.47 (d, *J* = 12.0 Hz, 1H, OCH), 4.46 (d, *J* = 12.0 Hz, 1H, OCH), 4.26 (ddd, *J* = 11.5, 8.5, 2.5, Hz, 0.9H, NCH), 4.02 (ddd, *J* = 10.5, 9.0, 7.5 Hz, 0.1H, NCH), 3.79 (ddd, *J* = 11.5, 9.0, 9.0 Hz, 0.9H, NCH), 3.73–3.61 (m, 0.1H, NCH), 3.44 (s, 2.7H, CMe), 3.41 (s, 0.3H, CMe), 2.59 (dd, *J* = 9.0, 4.5 Hz, 0.9H, NCCH), 2.44–2.34 (m, 0.1H, NCH_2CH), 2.36–2.22 (m, 0.9H, NCH_2CH), 1.98 (dddd, *J* = 9.0, 7.0, 5.5, 2.5 Hz, 1H, NCCH), 1.92 (s, 3H, OMe), 1.88–1.78 (m, 1H, NCH_2CH), 1.14 (dd, *J* = 5.5, 4.5 Hz, 0.9H, NCCH), 1.08 (dd, *J* = 5.5, 5.5 Hz, 0.1H, NCCH); ^{13}C NMR (100.6 MHz, $CDCl_3$) (rotamers) δ 172.5 (C=O), 170.8 (C=O), 159.1 (*ipso*-Ar), 158.5

(*ipso*-Ar), 158.3 (*ipso*-Ar), 157.6 (*ipso*-Ar), 137.1 (Ar), 136.4 (Ar), 118.6 (ArC), 118.5 (Ar), 117.9 (Ar), 75.8 (OCH_2), 75.7 (OCH_2), 58.9 (C*Me*), 58.7 (C*Me*), 53.1 (NCH_2), 53.0 (NCH_2), 52.4 (NC), 52.0 (NC), 35.0 (NC*C*H), 31.0 (NC*C*H_2), 30.0 (NC*C*H_2), 28.0 (NCH_2*C*H_2), 27.8 (NCH_2*C*H_2), 23.6 (OMe), 23.2 (OMe); HRMS *m/z* calcd for $C_{14}H_{18}N_2O_2$ $(M + Na)^+$ 269.1260, found 269.1261 (0.0 ppm error).

Lab book reference RA 1-035

1-[1-(2-Methoxypyridin-3-yl)-2-azabicyclo[3.1.0]hexan-2-yl]ethan-1-one S45

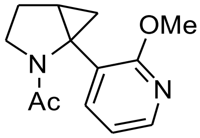

S45

HCl (6 mL of 4 N solution in dioxane) was added to *N*-Boc aryl cyclopropane **19** (80 mg, 0.26 mmol, 1.0 eq.) at rt under Ar. The resulting solution was stirred at rt for 1 h. Then, the solvent was evaporated under reduced pressure to give the crude HCl salt as a white solid. Ac_2O (0.15 mL, 1.56 mmol, 6.0 eq.) was added dropwise to a stirred solution of the crude HCl salt in pyridine (3 mL) at rt under Ar. The resulting solution was stirred at rt for 18 h. Then, the solvent was evaporated under reduced pressure to give a clear oil. CH_2Cl_2 (10 mL) and H_2O (15 mL) were added, and the two layers were separated. The aqueous layer was extracted with CH_2Cl_2 (3 × 10 mL) and the combined organics were dried ($MgSO_4$) and evaporated under reduced pressure to give the crude product as a yellow oil. Purification by flash column chromatography on silica with 89:10:1 CH_2Cl_2-Et_2O-Et_3N as eluent *N*-aryl cyclopropane **S45** (28 mg, 46%) as white solid, mp 108–110 °C; R_F (89:10:1 CH_2Cl_2-Et_2O-Et_3N) 0.31; IR (ATR) 2950, 2891, 1634 (C=O), 1402 cm^{-1}; ^1H NMR (400 MHz, $CDCl_3$) (85:15 mixture of rotamers) δ 8.06 (dd, *J* = 5.0, 2.0 Hz, 0.85H, Ar), 7.99 (dd, *J* = 5.0, 2.0 Hz, 0.15H, Ar), 7.70 (dd, *J* = 7.5, 2.0 Hz, 0.15H, Ar), 7.44 (dd, *J* = 7.5, 2.0 Hz, 0.85H, Ar), 6.85–6.78 (m, 1H, Ar), 4.15–4.01 (m, 0.15H, NCH), 4.01–3.96 (m, 1.7H, NCH), 3.93 (s, 2.55H, OMe), 3.92 (s, 0.45H, OMe), 3.49 (ddd, *J* = 10.0, 8.5, 4.0 Hz, 0.15H, NCH), 2.51–2.42 (m, 0.15H, NCH_2C*H*), 2.37 (dddd, *J* = 13.0, 7.0, 5.5, 2.0 Hz, 0.85H, NCH_2C*H*), 2.02–1.95 (m, 0.15H, NCH_2C*H*), 1.91 (s, 0.45H, CMe), 1.90–1.83 (m, 0.85H), 1.83–1.77 (m, 3.55H), 1.72 (dddd, *J* = 9.0, 7.0, 5.0, 1.0 Hz, 0.85H, NCCH), 1.59–1.54 (m, 0.15H, NCCH), 0.99 (dd, *J* = 5.0, 5.0 Hz, 0.85H, NCCH), 0.98–0.88 (m, 0.15H, NCCH); ^{13}C NMR (100.6 MHz, $CDCl_3$) (rotamers) δ 170.9 (C=O), 169.7 (C=O), 162.8 (*ipso*-Ar), 162.5 (*ipso*-Ar), 146.1 (Ar), 145.4 (Ar), 140.3 (Ar), 138.2 (Ar), 122.13 (*ipso*-Ar), 122.10 (*ipso*-Ar), 116.6 (Ar), 116.4 (Ar), 53.8 (OMe), 53.5 (OMe), 52.2 (NCH_2), 51.8 (NCH_2), 47.2

(NC), 47.0 (NC), 29.6 (NC*C*H), 27.8 (NCH_2*C*H_2), 27.0 (NCH_2*C*H_2), 25.8 (NC*C*H_2), 25.5 (NC*C*H), 23.8 (NC*C*H_2), 23.5 (C*Me*), 23.4 (C*Me*); HRMS *m/z* calcd for $C_{13}H_{16}N_2O_2$ (M + Na)$^+$ 255.1104, found 255.1102 (+0.8 ppm error)

Lab book reference RA 1-034

1-[1-(2-Methoxypyrimidin-5-yl)-2-azabicyclo[3.1.0]hexan-2-yl]ethan-1-one S46

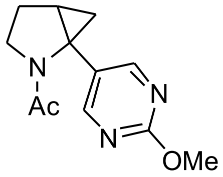

S46

HCl (5 mL of 4 N solution in dioxane) was added to *N*-Boc aryl cyclopropane **16** (50 mg, 0.17 mmol, 1.0 eq.) at rt under Ar. The resulting solution was stirred at rt for 1h. Then, the solvent was evaporated under reduced pressure to give crude HCl salt as a white solid. Ac_2O (95 µL, 1.02 mmol, 6.0 eq.) was added dropwise to a stirred solution of the crude HCl salt in pyridine (2 mL) at rt under Ar. The resulting solution was stirred at rt for 18 h. Then, the solvent was evaporated under reduced pressure to give a clear oil. CH_2Cl_2 (7 mL) and H_2O (10 mL) were added, and the two layers were separated. The aqueous layer was extracted with CH_2Cl_2 (3 × 7 mL), and the combined organics were dried ($MgSO_4$) and evaporated under reduced pressure to give the crude product as a yellow oil. Purification by flash column chromatography on silica with 99:1 CH_2Cl_2-MeOH as eluent gave *N*-acyl cyclopropane **S46** (18 mg, 45%) as white solid, mp 126–128 °C; R_F (98:2 CH_2Cl_2-MeOH) 0.24; IR (ATR) 2934, 1646 (C=O), 1473, cm^{-1}; ^{1}H NMR (400 MHz, $CDCl_3$) (60:40 mixture of rotamers) δ 8.43 (s, 0.8H, Ar), 8.38 (s, 1.2H, Ar), 4.13 (ddd, *J* = 12.0, 9.0, 3.5 Hz, 0.6H, NCH), 4.06–3.93 (m, 0.4H, NCH), 3.98 (s, 1.8H, OMe), 3.95 (s, 1.2H, OMe), 3.86 (ddd, *J* = 12.0, 9.0, 9.0 Hz, 0.6H, NCH), 3.50 (ddd, *J* = 10.5, 9.0, 5.5 Hz, 0.4H, NCH), 2.50–2.43 (m, 0.4H, NCH_2C*H*), 2.43–2.33 (m, 0.6H, NCH_2C*H*), 2.11–2.03 (m, 0.4H, NCCH), 1.99 (s, 1.2H, C*Me*), 1.96–1.83 (m, 1.4H, CH), 1.87 (s, 1.8H, CMe), 1.82–1.72 (m, 0.6H, NCCH), 1.68–1.56 (m, 0.6H, NCCH), 1.13 (dd, *J* = 5.5, 5.5 Hz, 0.6H, NCCH), 1.07 (dd, *J* = 4.5, 4.5 Hz, 0.4H, NCCH); ^{13}C NMR (100.6 MHz, $CDCl_3$) (rotamers) δ 171.8 (C=O), 170.9 (C=O), 164.9 (Ar), 164.5 (Ar), 158.8 (Ar), 157.2 (Ar), 127.5 (Ar), 126.8 (Ar), 55.2 (OMe), 55.0 (OMe), 52.3 (NCH_2), 51.0 (NCH_2), 46.0 (NC), 46.1 (NC), 32.4 (NC*C*H), 27.2 (NCH_2*C*H_2), 27.1 (NCH_2*C*H_2), 26.6 (NC*C*H), 25.8 (NC*C*H_2), 23.6 (C*Me*), 23.5

(C*Me*), 22.1 (NC*C*H_2); HRMS *m/z* calcd for $C_{12}H_{15}N_3O_2$ (M + Na)$^+$ 256.1056, found 256.1056 (+0.2 ppm error).

Lab book reference RA 1-012/RA 1-033

1-[1-(5-Fluoro-3-methylpyridin-2-yl)-2-azabicyclo[3.1.0]hexan-2-yl]ethan-1-one S47

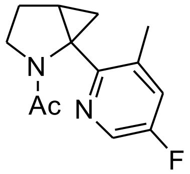

S47

HCl (6 mL of 4 N solution in dioxane) was added to *N*-Boc aryl cyclopropane **17** (83 mg, 0.28 mmol, 1.0 eq.) at rt under Ar. The resulting solution was stirred at rt for 1 h. Then, the solvent was evaporated under reduced pressure to give the crude HCl salt as a white solid. Ac_2O (0.16 mL, 1.7 mmol, 6.0 eq.) was added dropwise to a stirred solution of the crude HCl salt in pyridine (3 mL) at rt under Ar. The resulting solution was stirred at rt for 18 h. Then, the solvent was evaporated under reduced pressure to give a clear oil. CH_2Cl_2 (10 mL) and H_2O (15 mL) were added, and the two layers were separated. The aqueous layer was extracted with CH_2Cl_2 (3 × 10 mL), and the combined organics were dried ($MgSO_4$) and evaporated under reduced pressure to give the crude product as a yellow oil. Purification by flash column chromatography on silica with 197:2:1 CH_2Cl_2-MeOH-Et_3N as eluent gave *N*-aryl cyclopropane **S47** (41 mg, 60%) as white solid, mp 107–109 °C; R_F (98:1:1 CH_2Cl_2-MeOH-Et_3N) 0.34; IR (ATR) 2957, 2879, 1646 (C=O), 1470 cm^{-1}; ^{1}H NMR (400 MHz, $CDCl_3$) (85:15 mixture of rotamers) δ 8.17 (d, *J* = 2.5 Hz, 1H, Ar), 7.19 (dd, *J* = 9.0, 2.5 Hz, 0.85H, Ar), 7.07 (dd, *J* = 9.0, 2.5 Hz, 0.15H, Ar), 4.00–3.90 (m, 1H, NCH), 3.77 (ddd, *J* = 12.5, 9.5, 6.0 Hz, 0.85H, NCH), 3.49–3.3 (m, 0.15H, NCH), 2.55–2.46 (m, 0.15H, NCH_2C*H*), 2.46–2.35 (m, 0.85H, NCH_2C*H*), 2.32 (s, 3H, Me), 2.18–2.11 (m, 1H), 2.11–2.02 (m, 1H), 1.94 (s, 0.45H, Me), 1.88 (dd, *J* = 9.0, 6.0 Hz, 0.15H), 1.77 (ddd, *J* = 9.5, 7.0, 6.0 Hz, 0.85H), 1.65 (s, 2.55H, Me), 1.07 (dd, *J* = 5.5, 5.5 Hz, 0.15H, $NCCH_2$), 1.00 (dd, *J* = 5.5, 5.5 Hz, 0.85H, $NCCH_2$); ^{13}C NMR (100.6 MHz, $CDCl_3$) (rotamers) δ 172.32 (C=O), 169.98 (C=O), 158.7 (*J* = 257.54 Hz, Ar), 152.3 (*J* = 3.0 Hz, Ar), 135.5 (*J* = 5.03 Hz, Ar), 134.2 (*J* = 23.1 Hz, Ar), 133.9 (Ar), 125.4 (*J* = 18.1 Hz, Ar), 124.6 (*J* = 18.1 Hz), 51.3 (NC), 50.8 (NC), 49.9 (NCH_2), 49.0 (NCH_2), 29.7 (NC*C*H), 26.9 (NC*C*H), 26.2 (NCH_2*C*H_2), 25.9 (NCH_2*C*H_2), 23.5 (Me), 23.1 (Me), 22.8 (NC*C*H2), 20.1 (NC*C*H2), 19.0

(Me), 18.9 (Me); HRMS *m/z* calcd for $C_{13}H_{15}FN_2O$ (M + Na)$^+$ 257.1061, found 257.1056 (+1.8 ppm error).

Lab book reference RA 1-036

tert-Butyl 1-(1_H_-pyrrolo[2,3-_b_]pyridin-5-yl)-6-azaspiro[2.5]octane-6-carboxylate S48

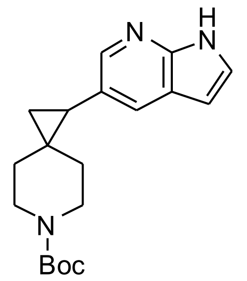

S48

Cs_2CO_3 (568 mg, 1.75 mmol, 3.0 eq.) was added to a stirred solution of arylated piperidine **25** (281 mg, 0.58 mmol, 1.0 eq.) and pyrrolidine (0.14 mL, 1.75 mmol, 3.0 eq.) in THF (5.8 mL) and MeOH (1.9 mL) and the resulting mixture was stirred at rt for 16 h. H_2O (15 mL) and EtOAc (15 mL) were added and the two layers were separated. The aqueous layer was extracted with EtOAc (3 × 15 mL). The combined organic extracts were washed with brine (20 mL), dried ($MgSO_4$) and evaporated under reduced pressure to give the crude product. Purification by flash column chromatography on silica with 2:3 hexane-EtOAc as eluent gave *N*'-H arylated piperidine **S48** (137 mg, 73%) as a clear oil, R_F (2:3 hexane-EtOAc) 0.20; ^{1}H NMR (400 MHz, $CDCl_3$) δ 11.55 (s, 1H, NH), 8.26 (s, 1H, Ar), 7.73 (d, *J* = 2.5 Hz, 1H, Ar), 7.38 (dd, *J* = 3.5, 2.5 Hz, 1H, Ar), 6.44 (d, *J* = 3.5 Hz, 1H, Ar), 3.68–3.60 (m, 1H, NCH), 3.52–3.43 (m, 1H, NCH), 3.34–3.27 (m, 1H, NCH), 3.22–3.10 (m, 1H, NCH), 2.10 (dd, *J* = 8.5, 5.5 Hz, 1H, CHAr), 1.63 (ddd, *J* = 12.5, 7.5, 4.0 Hz, 1H, CH), 1.52–1.40 (m, 10H, CH, CMe_3), 1.27–1.20 (m, 1H, CH), 1.15–1.06 (m, 1H, CH), 0.99 (dd, *J* = 5.5, 5.0 Hz, 1H, CH), 0.91 (dd, *J* = 8.5, 5.0 Hz, 1H, CH); ^{13}C NMR (100.6 MHz, $CDCl_3$) δ 155.1 (C=O), 147.8 (*ipso*-Ar), 144.0 (Ar), 128.8 (Ar), 126.0 (*ipso*-Ar), 125.8 (Ar), 120.2 (*ipso*-Ar), 100.2 (Ar), 79.4 (*C*Me_3), 43.5 (br, NCH_2), 36.7 (CH_2), 30.2 (CH_2), 28.5 (C*Me₃*), 26.3 (CHAr), 24.5 (C), 15.8 (*C*H_2CH); HRMS *m/z* calcd for $C_{19}H_{25}N_3O_2$ (M + H)$^+$ 327.1947, found 327.1948 (–0.3 ppm error).

Lab book reference: ARG-3-103

3-Oxo-3-(1-{1H-pyrrolo[2,3-b]pyridin-5-yl}-6-azaspiro[2.5]octan-6-yl)propanenitrile S49

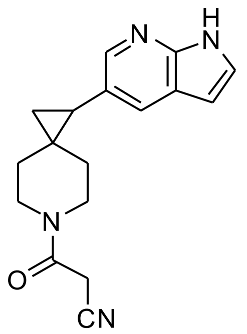

S49

4 N HCl in dioxane (4 mL) was added to a stirred solution of arylated piperidine **S47** (76 mg, 0.23 mmol, 1.0 eq.) in dioxane (1 mL) at rt under Ar. The resulting mixture was stirred at rt for 1 h and the solvent was evaporated under reduced pressure to give the crude HCl salt. The crude HCl salt was dissolved in DMF (4.6 mL) and cyanoacetic acid (25 mg, 0.26 mmol, 1.1 eq.) and Et_3N (0.13 mL, 0.92 mmol, 4.0 eq.) were added under Ar. The resulting solution was cooled to 0 °C and BOP (150 mg, 0.34 mmol, 1.5 eq.) was added and the solution was stirred at rt for 18 h. 1 M $HCl_{(aq)}$ (15 mL) was added and the two layers were separated. The aqueous layer was extracted with EtOAc (3 × 10 mL). The combined organic extracts were washed with brine (6 × 15 mL), dried ($MgSO_4$) and evaporated under reduced pressure to give the crude product. Purification by flash column chromatography on silica with 8:2 CH_2Cl_2-acetone as eluent gave amide **S49** (58 mg, 77%) as a white solid, mp 210–212 °C; R_F (6:4 CH_2Cl_2-acetone) 0.34; IR (ATR) 3212 (NH), 2921, 2258 (CN), 1650 (C=O), 1453, 740 cm^{-1}; ^{1}H NMR (400 MHz, $CDCl_3$) (55:45 mixture of rotamers) δ 11.37 (s, 0.55H, NH), 11.32 (s, 0.45H NH), 8.24–8.10 (m, 1H, Ar), 7.73–7.60 (m, 1H, Ar), 7.43–7.29 (m, 1H, Ar), 6.48–6.18 (m, 1H, Ar), 3.74 (ddd, *J* = 13.0, 7.0, 4.0 Hz, 0.55H, NCH), 3.62–3.37 (m, 2.9H, NCH, CNCH), 3.36–3.33 (m, 1H, CNCH), 3.25 (ddd, *J* = 12.5, 8.0, 4.0 Hz, 0.54H, NCH), 3.17 – 3.02 (m, 1H, NCH), 2.07 (dd, *J* = 8.0, 6.0 Hz, 1H, CH), 1.69 (ddd, *J* = 12.0, 8.0, 3.5 Hz, 0.45H, CH), 1.62–1.41 (m, 1.55H, CH), 1.27–1.01 (m, 2H, CH), 0.96 (dd, *J* = 6.0, 5.5 Hz, 1H, CH), 0.92–0.85 (m, 1H, CH); ^{13}C NMR (100.6 MHz, $CDCl_3$) (rotamers) δ 160.2 (C=O), 160.1 (C=O), 147.8 (*ipso*-Ar), 143.85 (Ar), 143.81 (Ar), 137.9 (Ar), 129.1 (Ar), 128.8 (Ar), 128.7 (Ar), 128.3 (Ar), 126.0 (Ar), 125.9 (Ar), 125.5 (*ipso*-Ar), 125.3 (Ar), 120.2 (*ipso*-Ar), 114.3 (CN), 114.2 (CN), 100.3 (Ar), 46.6 (NCH_2), 46.1 (NCH_2), 42.8 (NCH_2), 42.3 (NCH_2), 36.8 (CH_2), 35.9 (CH_2), 30.5 (CH_2), 29.6 (CH_2), 26.3 (CHAr), 26.2 (CHAr), 25.2 (CN*C*H_2), 25.1 (CN*C*H_2), 24.1 (C), 24.0 (C), 15.8 (CH_2), 15.7 (CH_2); HRMS *m/z* calcd for $C_{17}H_{18}N_4O$ $(M + Na)^+$ 317.1373, found 317.1770 (0.8 ppm error).

Lab book reference ARG-3-112

4-Bromo-7-(4-methylbenzenesulfonyl)-7H-pyrrolo[2,3-d]pyrimidine S50

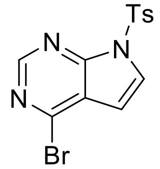

S50

A solution of 4-bromo-7*H*-pyrrolo[2,3-d]pyrimidine (1.0 g, 5.05 mmol, 1.0 eq.) in THF (20 mL) was added dropwise to a stirred mixture of NaH (60% dispersion in paraffin liquid) (263 mg, 6.57 mmol, 1.3 eq.) in THF (10 mL) at 0 °C under Ar. Then, a solution of TsCl (1.25 g, 6.57 mmol, 1.3 eq.) in THF (20 mL) was added dropwise to the stirred mixture at 0 °C under Ar. The reaction was warmed to rt and stirred for 16 h. H_2O (30 mL) was added, and the product was extracted with EtOAc (3 × 50 mL). The combined organic layers were dried (Na_2SO_4) and evaporated under reduced pressure to give the crude product. Purification by flash column chromatography on silica with 70:30 to 50:50 hexane–EtOAc as eluent gave 4-bromo-tosyl-pyrrolopyrimidine **S50** (1.41 g, 79%) as a white solid, mp 148–151 °C, R_F (1:1 EtOAc–hexane) 0.60; IR (ATR) 1595, 1578, 1541, 1508, 1429, 1373, 1349, 1190, 1175, 1152, 1100, 1188, 1008, 905, 830, 813, 728, 702, 682, 658, 627, 534, 516 cm^{-1}; ^{1}H NMR (400 MHz, $CDCl_3$) δ 8.70 (s, 1H, Ar), 8.11–8.03 (m, 2H, Ts–Ar), 7.78 (d, J = 4.0 Hz, 1H, Ar), 7.35–7.28 (m, 2H, Ts–Ar), 6.63 (d, J = 4.0 Hz, 1H, Ar), 2.39 (s, 3H, Me); ^{13}C NMR (100.6 MHz, $CDCl_3$) 152.5 (Ar), 149.9 (*ipso*–Ar), 146.5 (*ipso*–Ar), 145.0 (*ipso*–Ar), 134.4 (*ipso*–Ar), 130.1 (Ts–Ar), 128.7 (Ts–Ar), 128.2 (Ar), 123.1 (*ipso*–Ar), 104.4 (Ar), 21.8 (Me); HRMS (ESI) *m/z* calcd for $C_{13}H_{10}BrN_3O_2S$ (M + Na)$^+$ 373.9569, found 373.9575 (–1.6 ppm error).

Lab Book Reference: SY-X-240

tert-Butyl 1-[7-(4-methylbenzenesulfonyl)-7H-pyrrolo[2,3-d]pyrimidin-4-yl]-6-azaspiro[2.5]octane-6-carboxylate S51

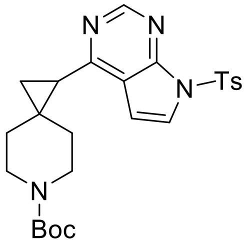

S51

Using general procedure A, MIDA boronate **1b** (366 mg, 1.0 mmol, 1.0 eq.), Cs_2CO_3 (1.95 g, 6.0 mmol, 6.0 eq.), 4-bromo-*N*-tosyl-diazaindole **S50** (456 mg, 1.3 mmol, 1.3 eq.), $Pd(OAc)_2$ (34 mg, 0.15 mmol, 0.15 eq.) and PCy_3 (84 mg, 0.3 mmol, 0.30 eq.) in toluene (20 mL) and H_2O (2 mL) gave the crude product. Purification by flash column chromatography on silica with 7:3 hexane-EtOAc as eluent gave arylated piperidine **S51** (338 mg, 70%) as a white solid, mp 94–96 °C; R_F (7:3 hexane-EtOAc) 0.24; IR (ATR) 2926, 1686 (C=O), 1572, 1176, 1130, 680, 579 cm^{-1}; ^{1}H NMR (400 MHz, $CDCl_3$) δ 8.76 (s, 1H, Ar), 8.03 (d, *J* = 8.5 Hz, 2H, Ar), 7.62 (d, *J* = 4.0 Hz, 1H, Ar), 7.25 (d, *J* = 8.5 Hz, 2H, Ar), 6.66 (d, *J* = 4.0 Hz, 1H, Ar), 3.55–3.44 (m, 1H, NCH), 3.38 (ddd, *J* = 12.5, 7.0, 4.0 Hz, 1H, NCH), 3.19 (ddd, *J* = 13.0, 7.0, 4.0 Hz, 1H, NCH), 2.81 (ddd, *J* = 13.0, 8.0, 3.5 Hz, 1H, NCH), 2.33 (s, 3H, Me), 2.16 (dd, *J* = 8.0, 5.5 Hz, 1H, CHAr), 1.72 (dd, *J* = 5.5, 4.5 Hz, 1H, CH), 1.60–1.46 (m, 3H, CH), 1.41–1.30 (m, 10H, CMe_3, CH), 1.04 (dd, *J* = 8.0, 4.5 Hz, 1H, CH); ^{13}C NMR (100.6 MHz, $CDCl_3$) δ 155.0 (C=O), 152.6 (Ar), 150.2 (*ipso*-Ar), 145.9 (*ipso*-Ar), 135.0 (*ipso*-Ar), 130.0 (Ar), 128.3 (Ar), 125.8 (Ar), 120.6 (*ipso*-Ar), 103.0 (Ar), 79.5 (*C*Me$_3$), 37.15 (NCH_2, only resolved in DEPT-135), 34.8 (CH_2), 31.0 (C), 28.5 (C*Me$_3$*), 28.0 (CH_2), 27.2 (CHAr), 21.8 (Me), 19.1 (CH*C*H$_2$) (1 x *ipso*-Ar resonance not resolved); HRMS (ESI) *m/z* calcd for $C_{25}H_{30}N_4O_4S$ $(M + Na)^+$ 505.1880, found 505.1889 (–1.9 ppm error).

Lab book reference: ARG-3-121

***tert*-Butyl 1-(7*H*-pyrrolo[2,3-*d*]pyrimidin-4-yl)-6-azaspiro[2.5]octane-6-carboxylate S52**

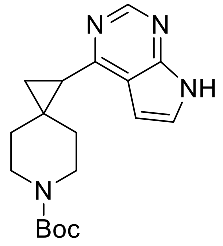

S52

Cs_2CO_3 (470 mg, 1.44 mmol, 3.0 eq.) was added to a stirred solution of arylated piperidine **S51** (231 mg, 0.48 mmol, 1.0 eq.) and pyrrolidine (0.16 mL, 1.91 mmol, 4.0 eq.) in THF (5 mL) and MeOH (1.6 mL) and the resulting mixture was stirred at rt for 16 h. H_2O (15 mL) and EtOAc (15 mL) were added and the two layers were separated. The aqueous layer was extracted with EtOAc (3 × 15 mL). The combined organic extracts were washed with 1 M $HCl_{(aq)}$ (15 mL) and brine (15 mL), dried ($MgSO_4$) and evaporated under reduced pressure to give the crude product. Purification by flash column chromatography on silica with 2:3 hexane-EtOAc as eluent gave *N*'-H arylated piperidine **S52** (121 mg, 77%) as a white solid, mp 156–158 °C; R_F (3:2 hexane-EtOAc) 0.11; IR (ATR) 3135 (NH), 2928, 1678, 1597 (C=O), 1423, 1239, 731 cm^{-1}; ^1H NMR (400 MHz, $CDCl_3$) δ 11.56 (s, 1H, NH), 8.75 (s, 1H, Ar), 7.34 (dd, *J* = 3.5, 2.0 Hz, 1H, Ar), 6.70–6.55 (m, 1H, Ar), 3.68–3.58 (m, 1H, NCH), 3.46 (ddd, *J* = 12.5, 8.0, 4.0 Hz, 1H, NCH), 3.30 (ddd, *J* = 13.0, 6.5, 4.0 Hz, 1H, NCH), 2.91–2.77 (m, 1H, NCH), 2.37 (dd, *J* = 8.0, 5.5 Hz, 1H, CHAr), 1.83 (dd, *J* = 5.5, 4.5 Hz, 1H, CH), 1.73 – 1.31 (m, 13H, CH, CMe_3), 1.10 (dd, *J* = 8.0, 4.5 Hz, 1H, CH); ^{13}C NMR (100.6 MHz, $CDCl_3$) δ 161.0 (*ipso*-Ar), 155.0 (C=O), 150.8 (*ipso*-Ar), 150.5 (Ar), 124.9 (Ar), 118.9 (*ipso*-Ar), 99.8 (Ar), 79.5 (*C*Me_3), 43.6 (br, NCH_2), 37.2 (CH_2), 30.0 (C), 28.5 (C*Me*$_3$), 28.3 (CH_2), 27.5 (CHAr), 18.3 (CH_2); HRMS *m/z* calcd for $C_{18}H_{24}N_4O_2$ $(M + H)^+$ 329.1972, found 329.1971 (+0.3 ppm error).

Lab book reference: ARG-3-125

3-Oxo-3-(1-{7H-pyrrolo[2,3-d]pyrimidin-4-yl}-6-azaspiro[2.5]octan-6-yl)propanenitrile S53

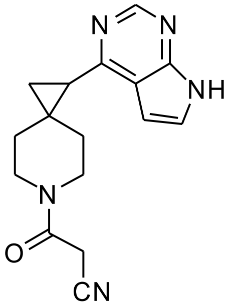

S53

4 N HCl in dioxane (4 mL) was added to a stirred solution of arylated piperidine **S52** (80 mg, 0.24 mmol, 1.0 eq.) in dioxane (1 mL) at rt under Ar. The resulting mixture was stirred at rt for 1 h and the solvent was evaporated under reduced pressure to give the crude HCl salt. The crude HCl salt was dissolved in DMF (4.8 mL) and cyanoacetic acid (23 mg, 0.27 mmol, 1.1 eq.) and Et_3N (0.13 mL, 0.96 mmol, 4.0 eq.) were added under Ar. The resulting solution was cooled to 0 °C and BOP (150 mg, 0.34 mmol, 1.5 eq.) was added and the solution was stirred at rt for 18 h. 1 M $HCl_{(aq)}$ (15 mL) was added and the two layers were separated. The aqueous layer was extracted with EtOAc (3 × 10 mL). The combined organic extracts were washed with brine (6 × 15 mL), dried ($MgSO_4$) and evaporated under reduced pressure to give the crude product. Purification by flash column chromatography on silica with 6:4 CH_2Cl_2-acetone as eluent gave amide **S53** (46 mg, 66%) as a white solid, mp 210–212 °C; R_F (6:4 CH_2Cl_2-acetone) 0.20; IR (ATR) 3229 (NH), 2854, 2270 (CN), 1652 (C=O), 1454, 744 cm^{-1}; ^{1}H NMR (400 MHz, DMSO-d_6) (60:40 mixture of rotamers) δ 11.97 (s, 1H, NH), 8.55 (s, 1H, Ar), 7.48–7.32 (m, 1H, Ar), 7.06–6.68 (m, 1H, Ar), 4.13–3.66 (m, 2.6H, CHCN, NCH), 3.51–3.37 (m, 1.8H, NCH), 3.18–2.97 (m, 0.6H, NCH), 2.80–2.32 (m, 2H, NCH, CHAr), 1.92–1.29 (m, 5H, CH), 1.06 (dd, J = 8.0, 4.0 Hz, 1H, CH); ^{13}C NMR (100.6 MHz, DMSO-d_6) (rotamers) δ 161.78 (*ipso*-Ar), 159.8 (C=O), 150.9 (*ipso*-Ar), 150.8 (Ar), 150.7 (Ar), 126.4 (Ar), 122.6 (*ipso*-Ar), 118.5 (*ipso*-Ar), 116.8 (CN), 116.7 (CN), 99.5 (Ar), 46.2 (NCH_2), 45.9 (NCH_2), 42.4 (NCH_2), 42.1 (NCH_2), 36.9 (CH_2), 36.3 (CH_2), 29.6 (C), 28.6 (CH_2), 27.9 (CH_2), 26.7 (CHAr), 25.4 ($CNCH_2$), 25.3 ($CNCH_2$), 18.4 (CH_2), 18.3 (CH_2); HRMS *m/z* calcd for $C_{16}H_{17}N_5O$ (M + H)$^+$ 296.1506, found 296.0506 (–0.1 ppm error).

Lab book reference ARG-3-135

1-(1-{7H-Pyrrolo[2,3-d]pyrimidin-4-yl}-6-azaspiro[2.5]octan-6-yl)prop-2-en-1-one 95

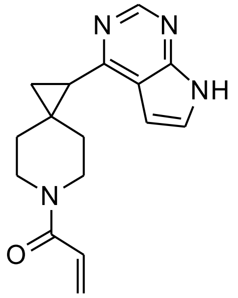

95

4 N HCl in dioxane (5 mL) was added to a stirred solution of arylated piperidine **S52** (101 mg, 0.31 mmol, 1.0 eq.) in dioxane (1 mL) at rt under Ar. The resulting mixture was stirred at rt for 1 h and the solvent was evaporated under reduced pressure to give the crude HCl salt. The crude HCl salt was suspended in DMF (2 mL) and Et_3N (0.21 mL, 1.55 mmol, 5.0 eq.) were added under Ar. The resulting solution was cooled to 0 °C and acryloyl chloride (28 μL, 0.33 mmol, 1.1 eq.) was added and the solution was stirred at rt for 4 h. 1 M $HCl_{(aq)}$ (15 mL) was added and the two layers were separated. The aqueous layer was extracted with EtOAc (3 × 15 mL). The combined organic extracts were washed with brine (6 × 15 mL), dried ($MgSO_4$) and evaporated under reduced pressure to give the crude product. Purification by flash column chromatography on silica with 6:4 CH_2Cl_2-acetone as eluent gave acrylamide **95** (36 mg, 42%) as a white solid, mp 164–166 °C; R_F (6:4 CH_2Cl_2-acetone) 0.2; IR (ATR) 3134 (NH), 2852, 1563 (C=O), 1444, 722 cm^{-1}; ^1H NMR (400 MHz, $CDCl_3$) (60:40 mixture of rotamers) δ 11.89–11.58 (m, 1H, NH), 9.10–8.55 (m, 1H, Ar), 7.37–7.31 (m, 1H, Ar), 6.70–6.38 (m, 2H, =C*H*H'), 6.28–6.13 (m, 1H, =CH*H'*), 5.71–5.50 (m, 1H, =CH), 3.97–3.88 (m, 0.6H, NCH), 3.76–3.53 (m, 1.8H, NCH), 3.45–3.34 (m, 0.6H, NCH), 3.15–3.08 (m, 0.4H, NCH), 3.05–2.96 (m, 0.6H, NCH), 2.46–2.37 (m, 1H, CHAr), 1.88–1.83 (m, 1H, CH), 1.76–1.50 (m, 4H, CH), 1.17–1.10 (m, 1H, CH); ^{13}C NMR (100.6 MHz, $CDCl_3$) (rotamers) δ 211.0 (C=O), 165.7 (*ipso*-Ar), 160.5 (*ipso*-Ar), 160.3 (*ipso*-Ar), 150.8 (*ipso*-Ar), 150.4 (Ar), 127.9 (=CH), 127.5(=CH_2), 125.2 (Ar), 118.9 (*ipso*-Ar), 99.6 (Ar), 99.4 (Ar), 45.9 (NCH_2), 42.3 (NCH_2), 42.1 (NCH_2), 37.9 (CH_2), 36.8 (CH_2) 29.8 (C), 29.6 (C), 29.1 (CH_2), 28.0 (CH_2), 27.5 (CHAr), 18.4 (CH*C*H_2), 18.2 (CH*C*H_2); HRMS *m/z* calcd for $C_{16}H_{18}N_4O$ $(M + H)^+$ 283.1553, found 283.1557 (–1.2 ppm error).

Lab book reference ARG-3-120

***tert*-Butyl (1*R**,3*R**)-1-[7-(4-methylbenzenesulfonyl)-7H-pyrrolo[2,3-d]pyrimidin-4-yl]-5-azaspiro[2.4]heptane-5-carboxylate S54**

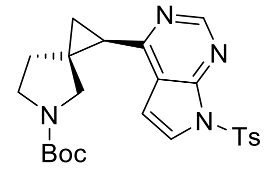

S54

Using general procedure A, MIDA boronate **1e** (247 mg, 0.70 mmol, 1.0 eq.), 4-bromo-*N*-tosyl-diazaindole **S50** (344 mg, 0.98 mmol, 1.4 eq.), PCy_3 (59 mg, 0.21 mmol, 0.30 eq.), Cs_2CO_3 (1.37 g, 4.19 mmol, 6.0 eq.) and $Pd(OAc)_2$ (24 mg, 0.11 mmol, 0.15 eq.) in toluene (10 mL) and water (1 mL) gave the crude product. Purification by flash column chromatography on silica with 98:2 CH_2Cl_2–Acetone as eluent gave cyclopropyl-tosyl-pyrrolo pyrimidine **S54** (276 mg, 84%) as an off-white solid, mp 86–88 °C, R_F (9:1 CH_2Cl_2–Acetone) 0.52; IR (ATR) 2982, 2930, 2874, 1692 (C=O), 1576, 1449, 1403 (S=O), 1382, 1364, 1179, 1159, 1129, 1092, 1043, 683, 580 cm^{-1}; ^1H NMR (400 MHz, $CDCl_3$) (55:45 mixture of rotamers) δ 8.87–8.75 (m, 1H, Ar), 8.14–8.03 (m, 2H, Ts–Ar), 7.70–7.63 (m, 1H, Ar), 7.34–7.27 (m, 2H, Ts–Ar), 6.71–6.66 (m, 1H, Ar), 3.57–3.34 (m, 3H, NCH), 3.14–3.05 (m, 1H, NCH), 2.43–2.39 (m, 1H, C*H*Ar), 2.38 (s, 3H, Me), 2.09–1.81 (m, 3H, CH), 1.79–1.73 (m, 0.45H, CH), 1.38 (s, 4H, CMe_3), 1.35–1.33 (m, 0.55H, CH), 1.29 (s, 5H, CMe_3); ^{13}C NMR (100.6 MHz, $CDCl_3$) (rotamers) δ 162.0 (*ipso*-Ar), 154.5 (C=O), 153.0 (Ar), 152.8 (Ar), 150.4 (*ipso*-Ar), 145.9 (*ipso*-Ar), 135.0 (*ipso*-Ar), 130.0 (Ts–Ar), 128.3 (Ts–Ar), 125.9 (Ar), 120.4 (*ipso*-Ar), 102.9 (Ar), 79.3 (O*C*Me_3), 48.4 (NCH_2), 45.7 (NCH_2), 45.1 (NCH_2), 36.0 (CH_2), 35.3 (CH_2), 34.6 (CH_2), 33.4 (C), 28.5 (C*Me*$_3$), 26.2 (*C*HAr), 26.0 (*C*HAr), 21.8 (Me), 20.2 (CH_2), 19.4 (CH_2); HRMS (ESI) *m/z* calcd for $C_{24}H_{28}N_4O_4S$ $(M + Na)^+$ 491.1723, found 491.1723 (+0.1 ppm error).

Lab Book Reference: SY-3-238

tert-Butyl (1*R,3*R**)-1-{7H-pyrrolo[2,3-d]pyrimidin-4-yl}-5-azaspiro[2.4]heptane-5-carboxylate S55**

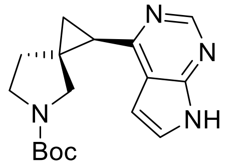

S55

Pyrrolidine (0.27 mL, 3.21 mmol, 5 eq.) and Cs_2CO_3 (627 mg, 1.93 mmol, 3.0 eq.) was added to a stirred solution of cyclopropyl-tosyl-pyrrolopyrimidine **S54** (301 mg, 0.64 mmol, 1.0 eq.) in MeOH (2.2 mL, 0.3 M) and THF (6.5 mL, 0.1 M). The resulting mixture was stirred at rt for 16 h. H_2O (10 mL) was added and the product was extracted with EtOAc (3 × 10 mL). The combined organic layers were dried (Na_2SO_4) and evaporated under reduced pressure to give the crude product. Purification by flash column chromatography on silica with 80:20 to 60:40 CH_2Cl_2–acetone as eluent gave cyclopropyl-pyrrolopyrimidine **S55** (166 mg, 82%) as a white solid, , mp 120–122 °C; R_F (6:4 CH_2Cl_2–acetone) 0.36; IR (ATR) 2975, 2934, 2870, 1684 (C=O), 1576, 1401, 1365, 1241, 1167, 1107, 911, 884, 771, 727, 579 cm^{-1}; ^1H NMR (400 MHz, $CDCl_3$) δ 8.76–8.64 (m, 1H, Ar), 7.16–7.03 (m, 1H, Ar), 6.60–6.48 (m, 1H, Ar), 3.82 (s, 3H, Me), 3.54–3.32 (m, 3H, NCH), 3.13–3.05 (m, 1H, NCH), 2.46 (dd, J = 8.5, 5.5 Hz, 1H, C*H*Ar), 2.11–1.82 (m, 3H, CH), 1.37 (s, 3.5H, CMe_3), 1.34–1.30 (m, 1H, CH), 1.28 (s, 5.5H, CMe_3); ^{13}C NMR (100.6 MHz, $CDCl_3$) δ 160.3 (*ipso*-Ar), 154.6 (C=O), 154.4 (C=O), 151.2 (Ar), 151.0 (Ar), 150.1 (*ipso*-Ar), 128.8 (Ar), 128.6 (Ar), 118.6 (*ipso*-Ar), 118.4 (*ipso*-Ar), 98.6 (Ar), 79.1 (O*C*Me_3), 48.5 (NCH_2), 45.7 (NCH_2), 45.2 (NCH_2), 36.0 (CH_2), 35.3 (CH_2), 33.5 (C), 32.3 (C), 31.2 (Me), 28.6 (C*Me*$_3$), 28.4 (C*Me*$_3$), 26.3 (*C*HAr), 26.2 (*C*HAr), 18.8 (CH_2), 17.9 (CH_2); MS (ESI) *m/z* 351 [$(M + Na)^+$, 100]; HRMS (ESI) *m/z* calcd for $C_{18}H_{24}N_4O_2$ $(M + Na)^+$ 351.1791, found 351.1801 (–2.7 ppm error).

Lab Book Reference: SY-2-249

3-Oxo-3-[(1*R,3*R**)-1-{7H-pyrrolo[2,3-d]pyrimidin-4-yl}-5-azaspiro[2.4]heptan-5-yl]propanenitrile S56**

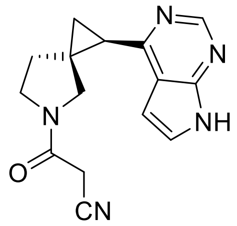

S56

HCl (5 mL of 4 N solution in dioxane) was added to cyclopropyl pyrrolopyrimidine **S55** (70 mg, 0.22 mmol, 1.0 eq.) at rt under Ar. The resulting solution was stirred at rt for 1 h. Then, the solvent was evaporated under reduced pressure to give the crude HCl salt. Then, Et_3N (0.1 mL, 0.89 mmol, 4.0 eq.), BOP (148 mg, 0.34 mmol, 1.5 eq.) and 2-cyanoacetic acid (21 mg, 0.25 mmol, 1.1 eq.) was added to a stirred solution of the crude HCl salt in DMF (5 mL) at rt under Ar. The resulting solution was stirred at rt for 16 h. H_2O (10 mL) was added the product was extracted with EtOAc (3 × 10 mL). The combined organics were washed with brine (3 × 10 mL), dried (Na_2SO_4) and evaporated under reduced pressure to give the crude product. Purification by flash column chromatography on silica with 50:50 acetone–CH_2Cl_2 as eluent gave cyano-cyclopropyl-pyrrolopyrimidine **S56** (37 mg, 59%) as a white solid, mp 188–190 °C, R_F (1:1 acetone–CH_2Cl_2) 0.18; IR (ATR) 3200 (NH), 3139 (NH), 2921, 2874, 2268 (CN), 1645 (C=O), 1579, 1443, 1359, 1299, 1255, 1157, 1025, 1006, 900, 850, 744, 607 cm^{-1}; ^1H NMR (400 MHz, DMSO-d_6) δ 12.03 (s, 1H, NH), 8.64–8.55 (m, 1H, Ar), 7.51–7.45 (m, 1H, Ar), 6.80–6.74 (m, 1H, Ar), 3.97–3.88 (m, 1H, CH), 3.87–3.63 (m, 1H, CH), 3.62–3.53 (m, 1H, NCH), 3.53–3.40 (m, 2H, NCH), 3.30–3.16 (m, 1H, NCH), 2.78–2.70 (m, 1H, C*H*Ar), 2.20–1.99 (m, 1.6H, CH), 1.97–1.87 (m, 0.4H, CH), 1.73–1.65 (m, 1H, CH), 1.42–1.34 (m, 1H, CH); ^{13}C NMR (100.6 MHz, DMSO-d_6) (rotamers) δ 161.0 (*ipso*-Ar), 160.9 (*ipso*-Ar), 159.6 (C=O), 159.4 (C=O), 150.6 (Ar), 150.5 (*ipso*-Ar), 125.9 (Ar), 117.8 (CN), 116.0 (*ipso*-Ar), 98.9 (Ar), 48.7 (NCH_2), 48.4 (NCH_2), 45.8 (NCH_2), 45.3 (NCH_2), 34.8 (CH_2), 33.6 (CH_2), 32.9 (C), 31.8 (C), 25.3 (*C*HAr), 25.2 (*C*HAr), 25.2 (*C*H_2CN), 24.9 (*C*H_2CN), 18.8 (CH_2), 18.3 (CH_2); HRMS (ESI) *m/z* calcd for $C_{15}H_{15}N_5O$ $(M + Na)^+$ 304.1169, found 304.1171 (–0.6 ppm error).

Lab Book Reference: SY-2-241

1-[(1*R,3*R**)-1-{7H-Pyrrolo[2,3-d]pyrimidin-4-yl}-5-azaspiro[2.4]heptan-5-yl]prop-2-en-1-one 96**

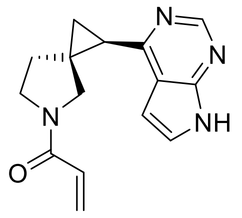

96

HCl (5 mL of 4 N solution in dioxane) was added to cyclopropyl pyrrolopyrimidine **S55** (165 mg, 0.53 mmol, 1.0 eq.) at rt under Ar. The resulting solution was stirred at rt for 1 h. Then, the solvent was evaporated under reduced pressure to give the crude HCl salt. Then, DIPEA (0.4 mL, 2.10 mmol, 4.0 eq.) and acryloyl chloride (52 mg, 0.58 mmol, 1.1 eq.) was added to a stirred solution of the crude HCl salt in DMF (5 mL) at rt under Ar. The resulting solution was stirred at rt for 16 h. H_2O (10 mL) was added the product was extracted with EtOAc (3 × 10 mL). The combined organics were dried (Na_2SO_4) and evaporated under reduced pressure to give the crude product. Purification by flash column chromatography on silica with 50:50 acetone–CH_2Cl_2 as eluent gave acyloyl-cyclopropyl-pyrrolopyrimidine **96** (36 mg, 26%) as a white solid, mp 142–145 °C, R_F (1:1 acetone–CH_2Cl_2) 0.16; ^{1}H NMR (400 MHz, DMSO-d_6) δ 12.02 (s, 1H, NH), 8.64–8.56 (m, 1H, Ar), 7.50–7.42 (m, 1H, Ar), 6.81–6.72 (m, 1H, Ar), 6.54 (dd, J = 16.5, 10.0 Hz, 0.55H, C*H*=CH$_2$), 6.27 (dd, J = 16.5, 10.0 Hz, 0.45H, C*H*=CH$_2$), 6.04 (dd, J = 16.5, 2.5 Hz, 0.55H, CH=C*H*$_2$), 5.98 (dd, J = 16.5, 2.5 Hz, 0.45H, CH=C*H*$_2$), 5.60 (dd, J = 10.0, 2.5 Hz, 0.55H, CH=C*H*$_2$), 5.54 (dd, J = 10.0, 2.5 Hz, 0.45H, CH=C*H*$_2$), 3.80–3.38 (m, 3.6H, NCH), 3.24–3.16 (m, 0.4H, NCH), 2.78–2.69 (m, 1H, C*H*Ar), 2.21–2.08 (m, 1H, CH), 2.08–1.95 (m, 1H, CH), 1.78–1.67 (m, 1H, CH), 1.41 (dd, J = 8.0, 4.0 Hz, 0.45H, CH), 1.37 (dd, J = 8.0, 4.0 Hz, 0.55H, CH); ^{13}C NMR (100.6 MHz, DMSO-d_6) (rotamers) δ 163.1 (*ipso*-Ar), 159.6 (C=O), 159.5 (C=O), 150.5 (Ar), 150.4 (*ipso*-Ar), 129.3 (*C*H=CH$_2$), 129.2 (*C*H=CH$_2$), 126.7 (CH=*C*H$_2$), 125.9 (Ar), 125.8 (Ar), 117.8 (*ipso*-Ar), 98.9 (Ar), 48.4 (NCH$_2$), 48.1 (NCH$_2$), 45.6 (NCH$_2$), 44.9 (NCH$_2$), 35.0 (CH$_2$), 33.4 (CH$_2$), 31.7 (C), 25.3 (*C*HAr), 25.2 (*C*HAr), 17.9 (CH$_2$); MS (ESI) *m/z* 291 [(M + Na)$^+$, 100]; HRMS (ESI) *m/z* calcd for $C_{15}H_{16}N_4O$ (M + Na)$^+$ 291.1216, found 291.1218 (–0.6 ppm error).

Lab Book Reference: SY-2-251

1.4. Synthesis of Potassium Trifluoroborates

The following potassium trifluoroborates were synthesised (see section 1.3 for details)

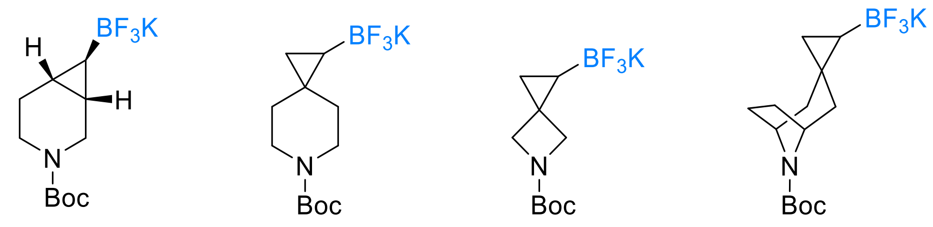

S2, 2.0 g **S7**, 1.4 g **S11**, 2.5 g **S12**, 0.27 g

Figure S1: Potassium trifluoroborate building blocks

1.5. Failed Suzuki-Miyaura Cross-Coupling Reactions

Synthesis of the products below failed, with either no product or only trace amounts observed.

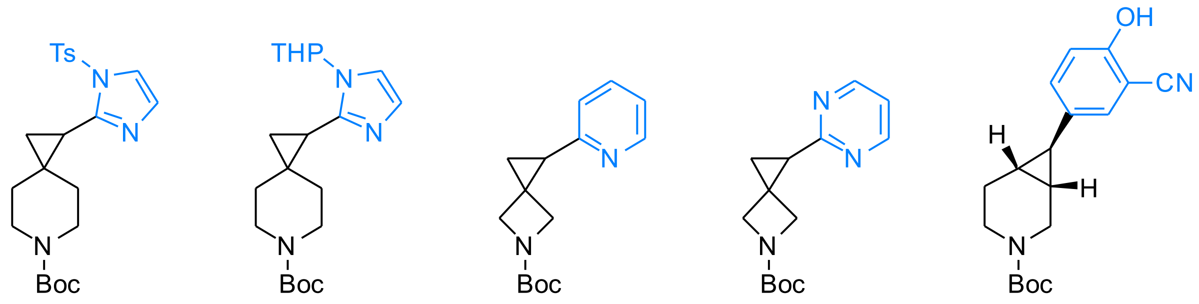

Conditions A Conditions A Conditions A Conditions A Conditions B

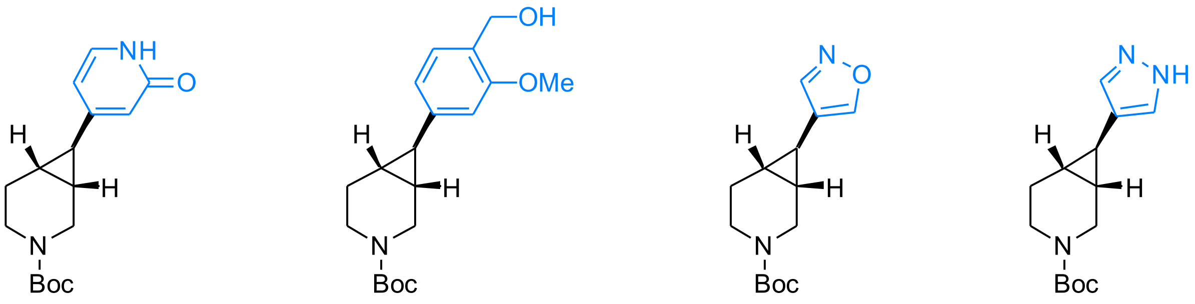

Conditions B Conditions B Conditions B Conditions B

Figure S2. Failed Suzuki-Miyaura cross-coupling reactions.

2. Compound Analysis

2.1. Exit Vector Analysis

We calculated the 3-D vectors for all 27 possible building blocks consisting of a fused- or spiro-cyclopropyl azetidine, pyrrolidine or piperidine. Using pyrimidine as an exemplar fragment hit, and four simple capping groups (R = Ms, Ac, Me and Ph) gave a total of 108 virtual elaborated fragments.

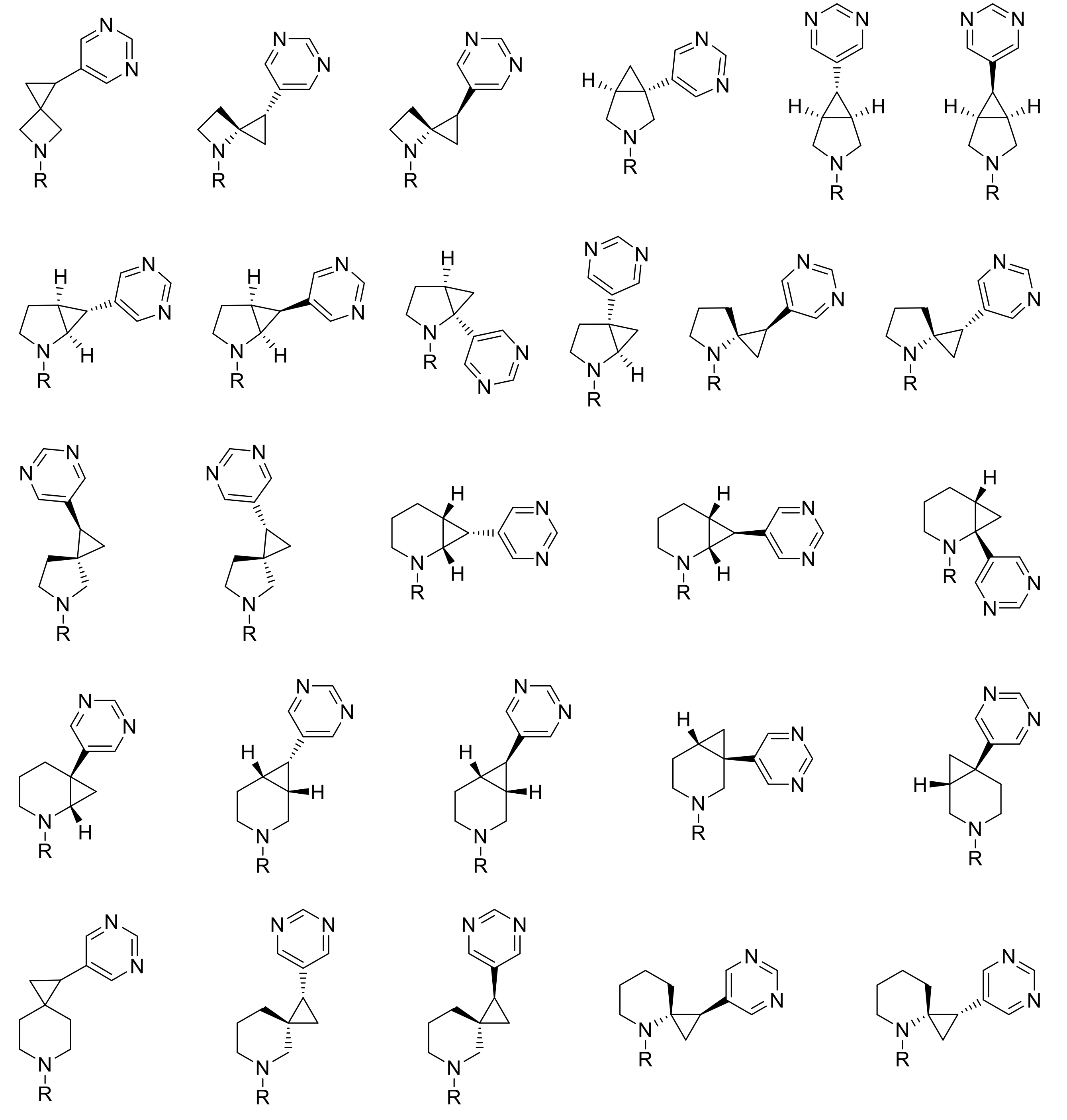

Figure S3. Virtual elaborated fragments used for Exit Vector analysis.

3-Dimensional structures of potential compounds were generated using Vernalis KNIME nodes v1.34.1 and RDKit v4.5.0 in KNIME v4.4.1, based upon the Vernalis example workflow (https://hub.knime.com/vernalis/spaces/Public/latest/PMI%20Plotting%20Example~zuyv2AxB_dFVs0Ym, accessed November 2022). A maximum of 50 conformers were generated for each molecule, using experimental torsions and 'basic knowledge'. The geometry of each was optimised using MMFF94 force field with 1000 iterations. Prior to calculation, explicit hydrogens were added. Post calculation, explicit hydrogens were kept and the lowest energy conformer of each molecule was selected. Exit Vectors were calculated according to the procedure described by Grygorenko and co-workers.[11]

The results are shown in the exit vector plot in Figure 1D.

X-ray crystallographic structures were used, together with calculation of exit vectors according to the procedure described by Grygorenko and co-workers.[11] to produce the exit vector plot in Figure 4B.

2.2. Lead-like Analysis

Properties of Synthesised Elaborated Building Blocks. Figure S4 shows all 32 lead-like compounds that were synthesised.

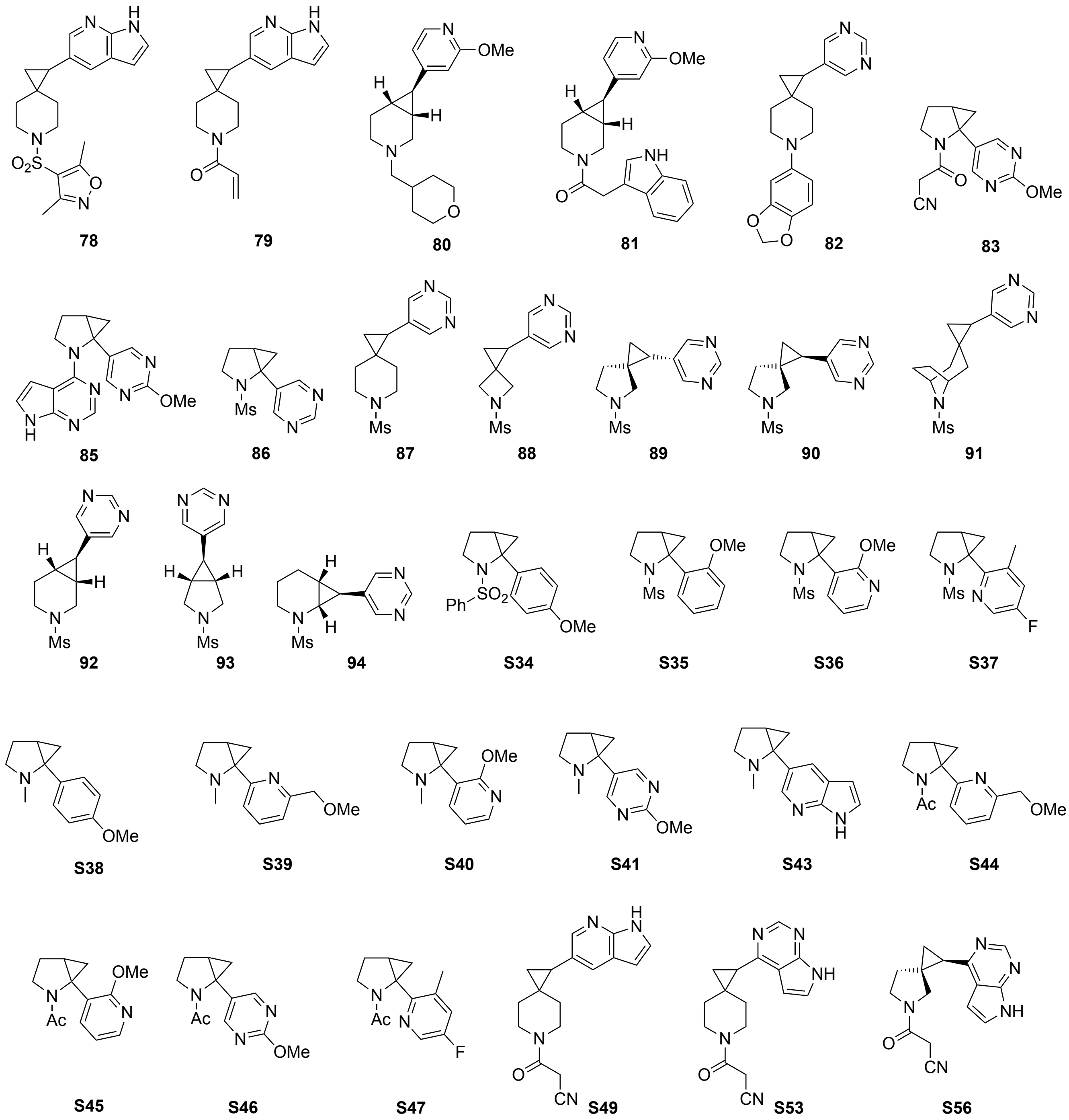

Figure S4. Compounds used for lead-like analysis.

Molecular weight and fraction of sp^3 carbons (Fsp^3) were calculated using RDKit v4.5 in KNIME v4.4.1. Prior to calculation, salts were stripped. ClogP values were calculated using Daylight/BioByte ClogP v4.3. The 32 exemplar compounds have a mean ClogP of 0.46, a mean MW of 266 and a mean fraction of sp^3 hybridised carbon atoms (Fsp^3)[12] of 0.56, and thus comfortably occupy lead-like space. ClogP, MW and Fsp^3 values for each compound in Figure S4 are provided in Table S1.

Table S1

Cpd No.	Smiles	ClogP	MW	Fsp^3
**78**	Cc1noc(C)c1S(=O)(=O)N1CCC2(CC1)CC2c1cnc2[nH]ccc2c1	3.13	386.5	0.47
**79**	C=CC(=O)N1CCC2(CC1)CC2c1cnc2[nH]ccc2c1	2.85	281.4	0.41
**80**	COc1cc([C@@H]2[C@H]3CCN(CC4CCOCC4)C[C@H]32)ccn1	2.55	302.4	0.72
**81**	COc1cc([C@@H]2[C@H]3CCN(C(=O)Cc4c[nH]c5ccccc45)C[C@H]32)ccn1	3.38	361.4	0.36
**82**	c1ncc(C2CC23CCN(c2ccc4c(c2)OCO4)CC3)cn1	2.98	309.4	0.44
**83**	COc1ncc(C23CC2CCN3C(=O)CC#N)cn1	0.846	258.3	0.54
**85**	COC(N=C1)=NC=C1C23N(C4=C(C=CN5)C5=NC=N4)CCC2C3	1.74	308.3	0.38
**86**	CS(=O)(=O)N1CCC2CC21c1cncnc1	0.357	239.3	0.6
**87**	CS(=O)(=O)N1CCC2(CC1)CC2c1cncnc1	1.01	267.4	0.6
**88**	CS(=O)(=O)N1CC2(CC2c2cncnc2)C1	0.226	239.3	0.6
**89**	CS(=O)(=O)N1CC[C@@]2(CC2c2cncnc2)C1	0.616	253.3	0.64
**90**	CS(=O)(=O)N1CC[C@@]2(CC2c2cncnc2)C1	0.616	253.3	0.64
**91**	CS(=O)(=O)N1C2CCC1CC1(C2)CC1c1cncnc1	1.94	293.3	0.71
**92**	CS(=O)(=O)N1CC[C@H]2[C@@H](C1)[C@@H]2c1cncnc1	0.472	253.3	0.64
**93**	CS(=O)(=O)N1C[C@H]2[C@H](c3cncnc3)[C@H]2C1	0.081	239.3	0.6
**94**	CS(=O)(=O)N1CCC[C@@H]2[C@@H](c3cncnc3)[C@@H]21	0.614	253.3	0.64
**S34**	O=S(N1C(C2)(C3=CC=C(OC)C=C3)C2CC1)(C4=CC=CC=C4)=O	3.51	345.5	0.37
**S35**	COc1ccccc1C12CC1CCN2S(C)(=O)=O	1.58	267.4	0.58
**S36**	COc1ncccc1C12CC1CCN2S(C)(=O)=O	0.971	268.3	0.58
**S37**	Cc1cc(F)cnc1C12CC1CCN2S(C)(=O)=O	1.41	270.3	0.58
**S38**	COc1ccc(C23CC2CCN3C)cc1	2.25	203.3	0.54
**S39**	COCc1cccc(C23CC2CCN3C)n1	1.78	218.3	0.62
**S40**	COc1ncccc1C12CC1CCN2C	1.64	204.3	0.58
**S41**	COc1ncc(C23CC2CCN3C)cn1	1.04	205.3	0.64
**S43**	CN1CCC2CC21c1cnc2[nH]ccc2c1	2.11	213.3	0.46
**S44**	COCc1cccc(C23CC2CCN3C(C)=O)n1	1.70	246.3	0.57
**S45**	COc1ncccc1C12CC1CCN2C(C)=O	1.56	232.3	0.54
**S46**	COc1ncc(C23CC2CCN3C(C)=O)cn1	0.952	233.3	0.58
**S47**	CC(=O)N1CCC2CC21c1ncc(F)cc1C	2.00	234.3	0.54
**S49**	N#CCC(=O)N1CCC2(CC1)CC2c1cnc2[nH]ccc2c1	2.57	294.4	0.47
**S53**	N#CCC(=O)N1CCC2(CC1)CC2c1ncnc2[nH]ccc12	1.97	295.3	0.5
**S56**	N#CCC(=O)N1CC[C@@]2(C[C@H]2c2ncnc3[nH]ccc23)C1	1.58	281.3	0.47

2.3. AstraZeneca's drug metabolism and pharmacokinetics (DMPK) Wave1 analysis

DMPK Analysis. Twelve lead-like compounds, **78**, **81**, **82** and **86-94**, were subjected to AstraZeneca's drug metabolism and pharmacokinetics (DMPK) Wave1 analysis.[13] This provides information on lipophilicity (measured logD), aqueous solubility and metabolic stability in human liver microsomes (HLM) and rat hepatocytes (RH). The results are shown in Table S2.

Table S2

**Compound**	**logD**	**Solubility** μM	**Rat Hepatocytes (RH) CL_{int}** μL min^{-1} mg^{-1}	**Human liver microsomes (HLM) CL_{int}** μL min^{-1} 1 x 10^{6} cells^{-1}
**78**	3.6	49.0	296	194
**81**	3.1	100	>300	251
**82**	0.1	887	90.9	93.8
**86**	–0.4	881	1.3	<3.0
**87**	0.2	931	1.83	<3.0
**88**	–0.6	913	<1.0	<3.0
**89**	–0.3	873	<1.0	<3.0
**90**	–0.3	861	1.67	<3.0
**91**	0.5	>981	5.38	<3.0
**92**	0.1	860	<1.0	<3.0
**93**	–0.3	822	<1.0	<3.0
**94**	0.1	823	1.38	<3.0

3. Molecular Modelling, DMPK Analysis and Inhibition Studies of JAK3 Inhibitors

Molecular Modelling.

Molecular modelling experiments were carried out using Schrodinger's Maestro v.13.0 (Schrodinger, Inc.: Maestro v13.0, 2021-4).

Protein and ligand preparation

The crystal structure of JAK3 with a covalent inhibitor (PDB: 5TOZ) was downloaded from RCSB PDB website. The protein was processed with the Protein Preparation Wizard[14] which corrected side chains, added missing atoms, eliminated partial occupant rotamers, fixed the orientation of Asn, Gln and His, removed water molecules far (>5 Å) from the ligand binding site. Finally, a restrained minimisation using the Impref module of Impact with the OPLS4[15] force field was performed until the average root mean square deviation of the nonhydrogen atoms reached 0.3 Å. Conformations and ionisation states of ligands were prepared at pH 7 ± 2 using the LigPrep[16] module.

Molecular Docking

Glide XP[17] (extra precision) was used for the noncovalent dockings. The following parameters were selected from Settings tab; bias sampling for torsion for amide only to penalise nonplanar conformation, reward of intramolecular hydrogen bonds and enhancement of planarity of conjugated pi groups. For the conformer generation and selection of initial poses the use of enhanced sampling was selected. From the output tab, the application of strain corrections term was included. The chosen docking settings were verified to successfully predict the known crystallographic ligand binding mode of PDB 5TOZ. The RMSD from the co-crystallised ligand (PF-06651600, Ritlecitinib) was used to guide the selection of the best poses. Ligands were covalently docked against JAK protein via a conjugate addition using Schrodinger's CovDock[18] package. The reactive residue undergoing nucleophilic addition to a double bond was set to be Cys909. The thoroughness was set to "pose prediction" for increased accuracy. The criteria used to classify poses were superposition of the adenine ring to the crystallised ligand (PF-06651600), linker position and ultimately the covalent bond position. In both docking procedures the grid box was centered on the inhibitor present in the crystal structure keeping default parameters. In all molecular modelling calculations (grid generation, noncovalent and covalent docking), the OPLS4[15] force field was used.

The calculated docking energies and modelling poses are shown in Table S3.

Table S3

Building block / scaffold	Structure / energy in kcal mol^{-1}	Pose
**1a**	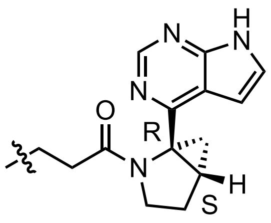<br>–9.328	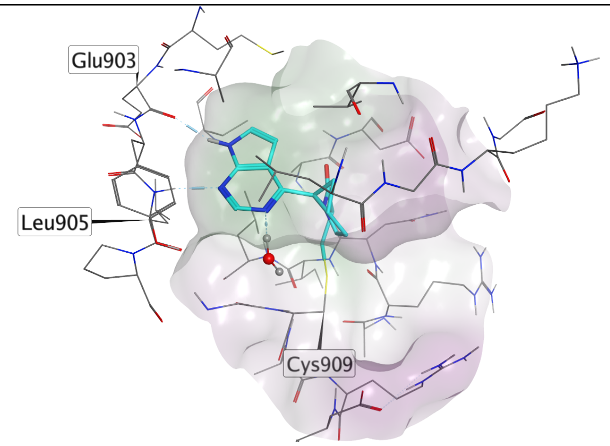
**1a**	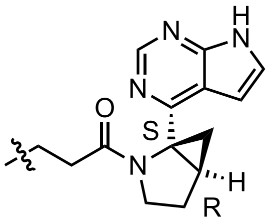<br>–8.626	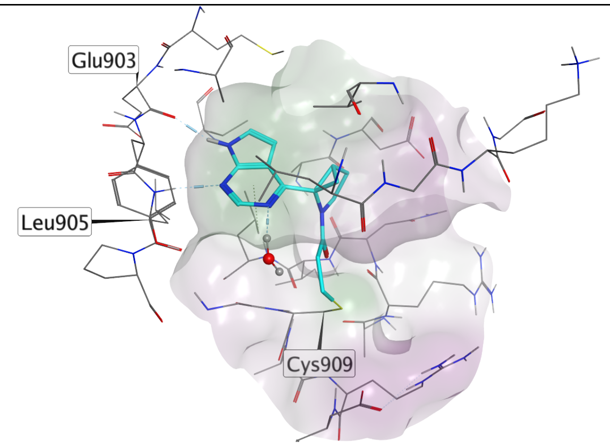
**1b**	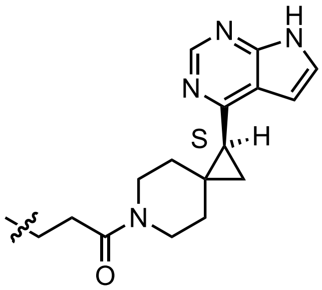<br>–6.838	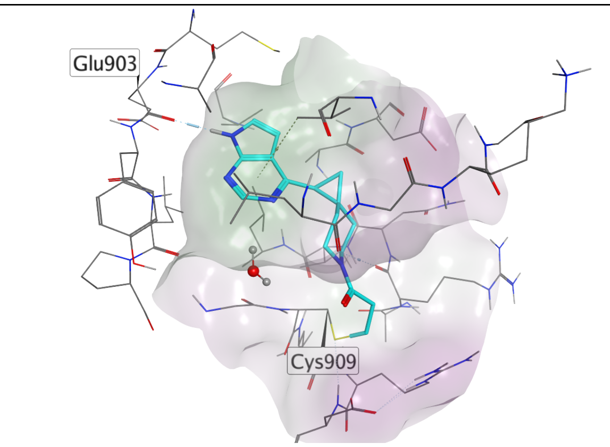

**1b**	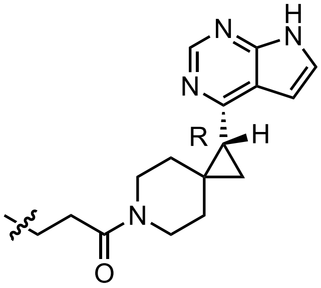 –8.274	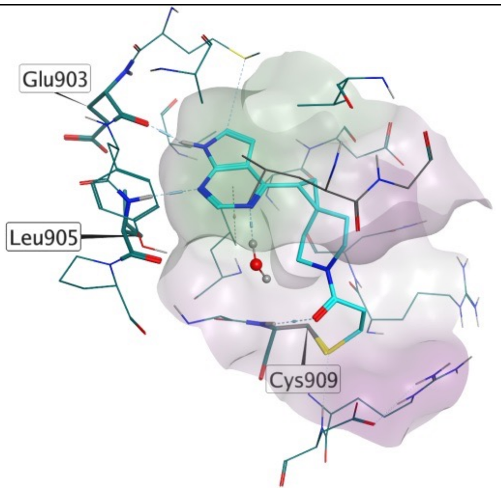
**1c**	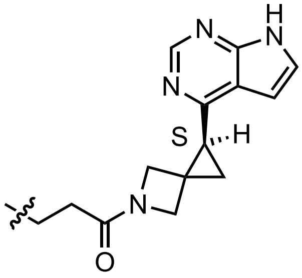 –8.521	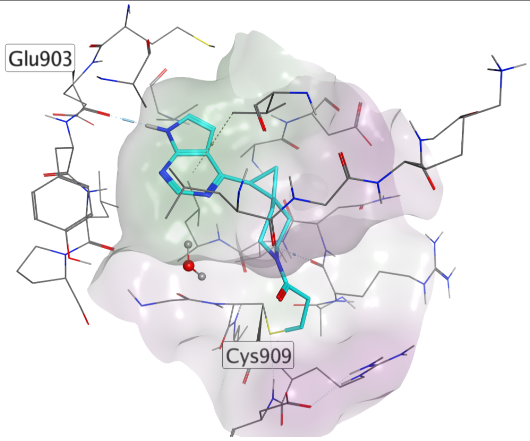
**1c**	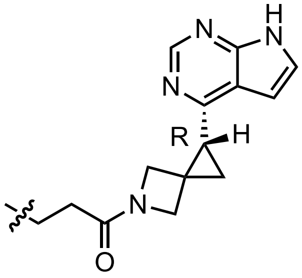 –7.846	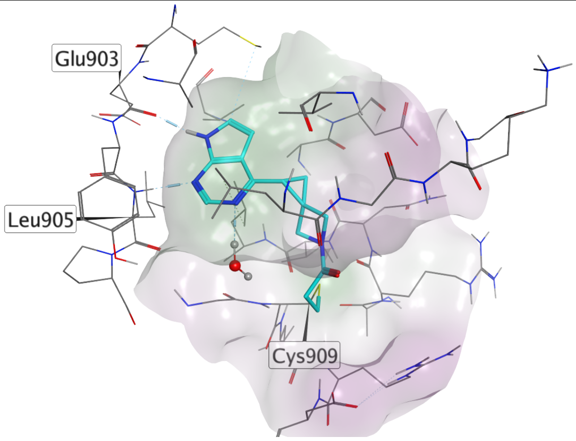

**1d**	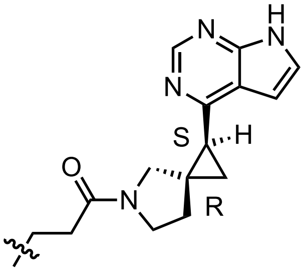 –9.133	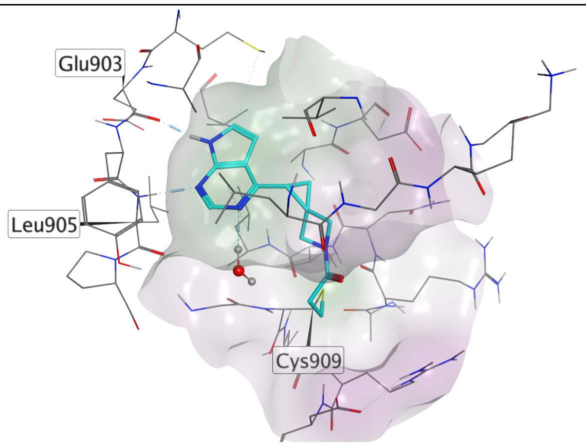
**1d**	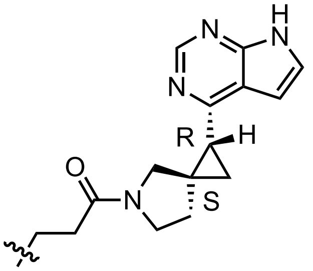 –9.017	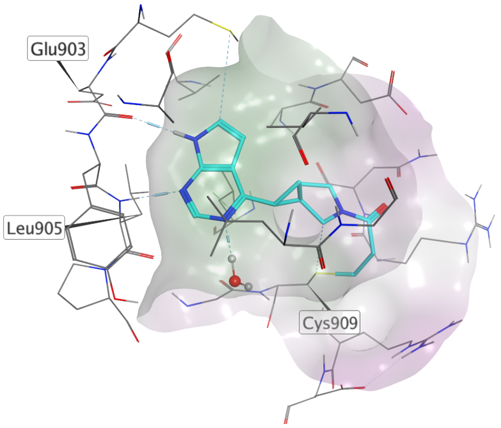
**1e**	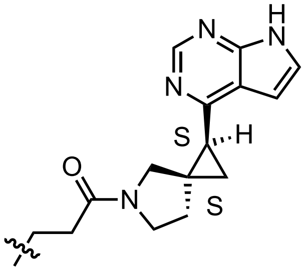 –9.135	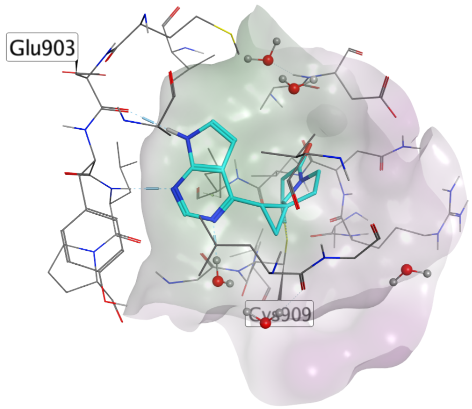

**1e**	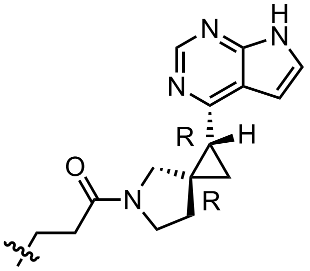 –10.21	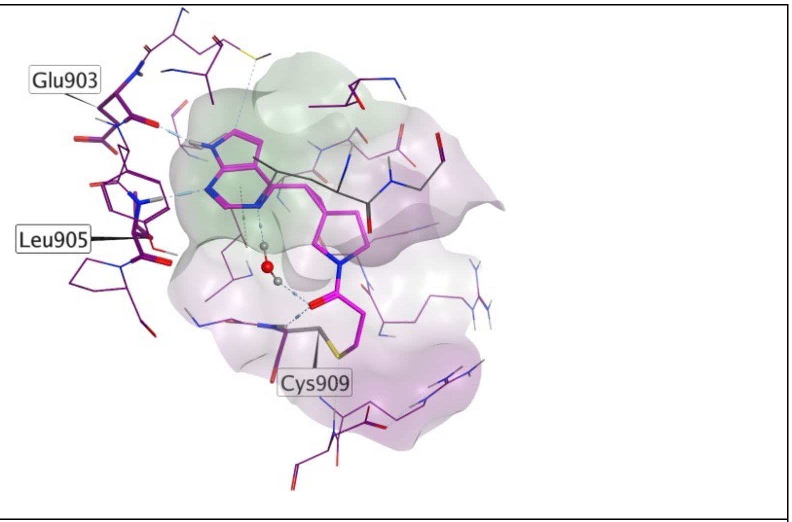
**1f**	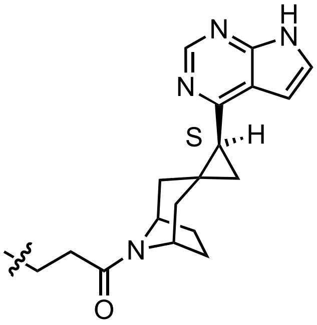 –7.107	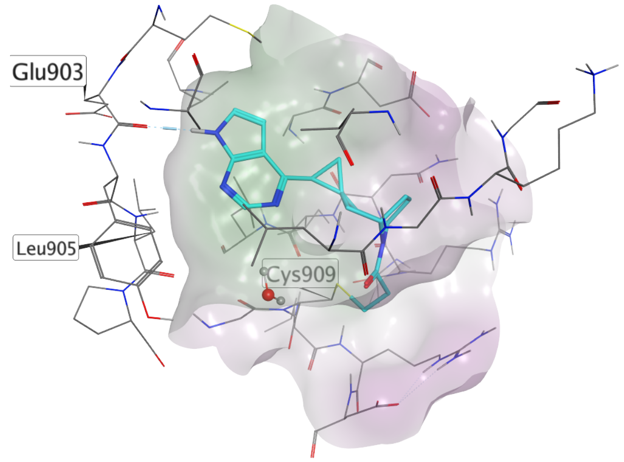
**1f**	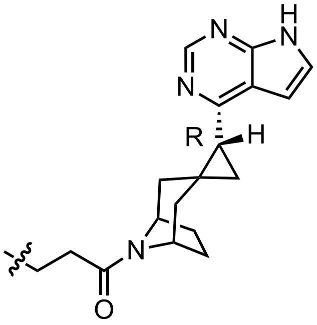 –8.470	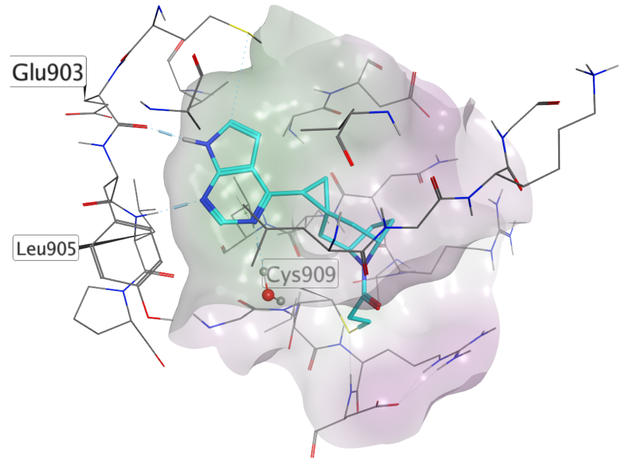

**1g**	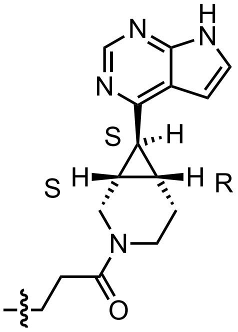 –8.91	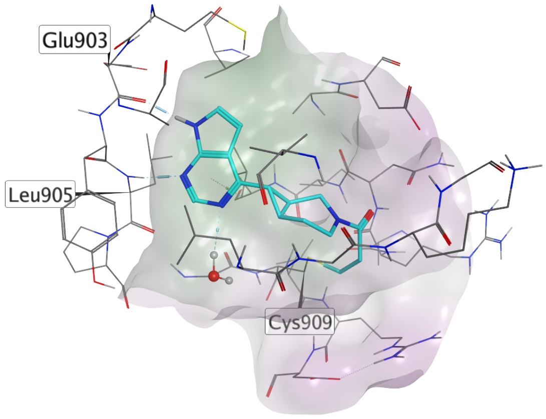
**1g**	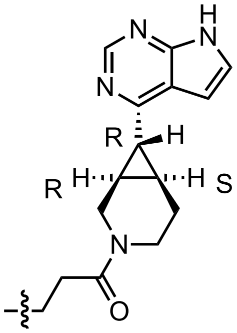 –9.134	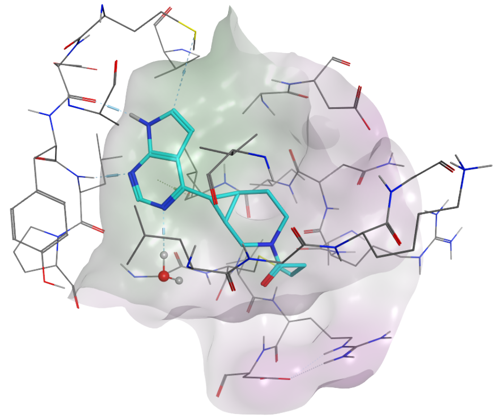
**1h**	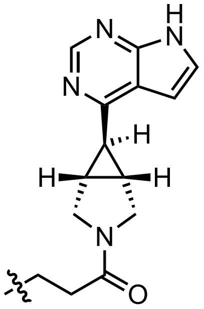 –9.49	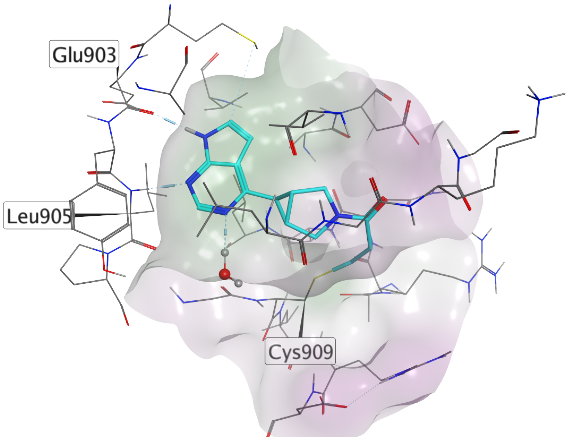

**1i**	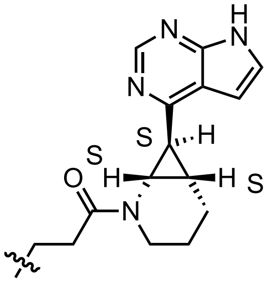 –10.188	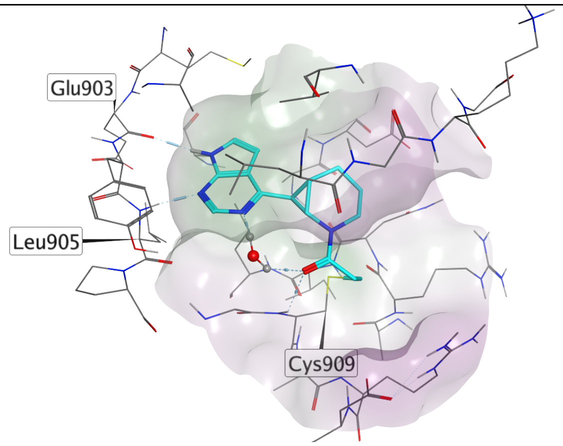
**1i**	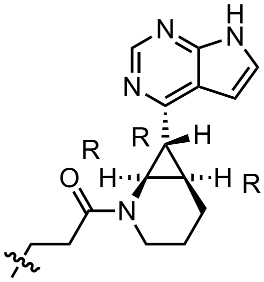 –10.143	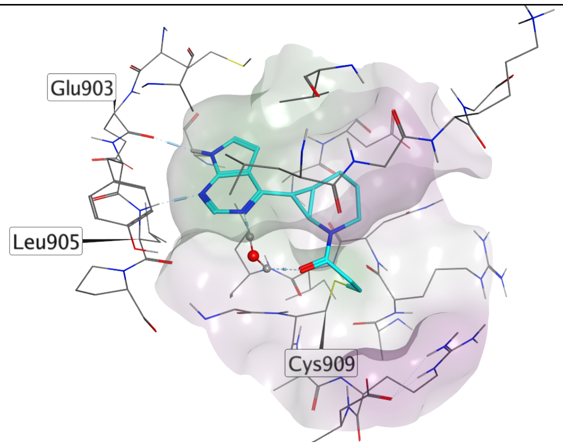

AstraZeneca's drug metabolism and pharmacokinetics (DMPK) Wave1 analysis.

DMPK Analysis. Compounds **95** and **96**, together with Ritlecitinib, were subjected to AstraZeneca's drug metabolism and pharmacokinetics (DMPK) Wave1 analysis.[13] This provides information on lipophilicity (measured logD), aqueous solubility and metabolic stability in human liver microsomes (HLM) and rat hepatocytes (RH). The results are shown in Table S4.

Glutathione (GSH) Reactivity. The data were collected using previously reported procedures.[19] The results are shown in Table S3.

Table S4

**Compound**	**logD**	**Solubility** μM	**Rat Hepatocytes (RH) CL$_{int}$** μL min^{-1} mg^{-1}	**Human liver microsomes (HLM) CL$_{int}$** μL min^{-1} 1 x 10^{6} cells^{-1}	**GSH Reactivity t$_{1/2}$** min
**95**	1.6	>1000.0	4.68	3.550	2005
**96**	1.2	334.0	5.65	14.5	1254
**Ritlecitinib**	1.6	996.0	2.90	<3.0	2020

Inhibition Studies. Kinase binding data was generated by Thermofischer using their Z-lyte technology. The ATP concentrations used in the activity assays were within 2-fold of the experimentally determined apparent Michaelis constant (Kmapp) value for each kinase. Details regarding the kinase proteins used and the assay protocols are available online.[20] The results are shown in Table S5.

Table S5

**Compound**	**JAK1 IC$_{50}$** nM IT25056 (JAK1 FRET-Z-Lyte CR) ATP Conc: 75.000 μM	**JAK2 IC$_{50}$** nM IT25058 (JAK2 FRET-Z-Lyte CR) ATP Conc: 25.000 μM	**JAK3 IC$_{50}$** nM IT25060 (JAK3 FRET-Z-Lyte CR) ATP Conc: 10.000 μM	**TYK2 IC$_{50}$** nM IT25308 (TYK2 FRET-Z-Lyte CR) ATP Conc: 25.000 μM
**Ritlecitinib**	2,520	2,020	0.55	7,500
**95**	>10,000	>10,000	1,230	>10.000
**96**	>10,000	>10,000	69	>10.000

JAK3 Assay

The 2X JAK3 / Tyr 06 mixture is prepared in 50 mM HEPES pH 7.5, 0.01% BRIJ-35, 10 mM $MgCl_2$, 1 mM EGTA. The final 10 uL Kinase Reaction consists of 0.5 - 3.58 ng JAK3 and 2 uM Tyr 06 in 50 mM HEPES pH 7.5, 0.01% BRIJ-35, 10 mM MgCl2, 1 mM EGTA. After the 1 hour Kinase Reaction incubation, 5 uL of a 1:128 dilution of Development Reagent A is added.

4. ^{1}H and ^{13}C NMR Spectra

400 MHz ^{1}H NMR spectrum; 100.6 MHz ^{13}C NMR spectrum; $CDCl_3$ of **3**

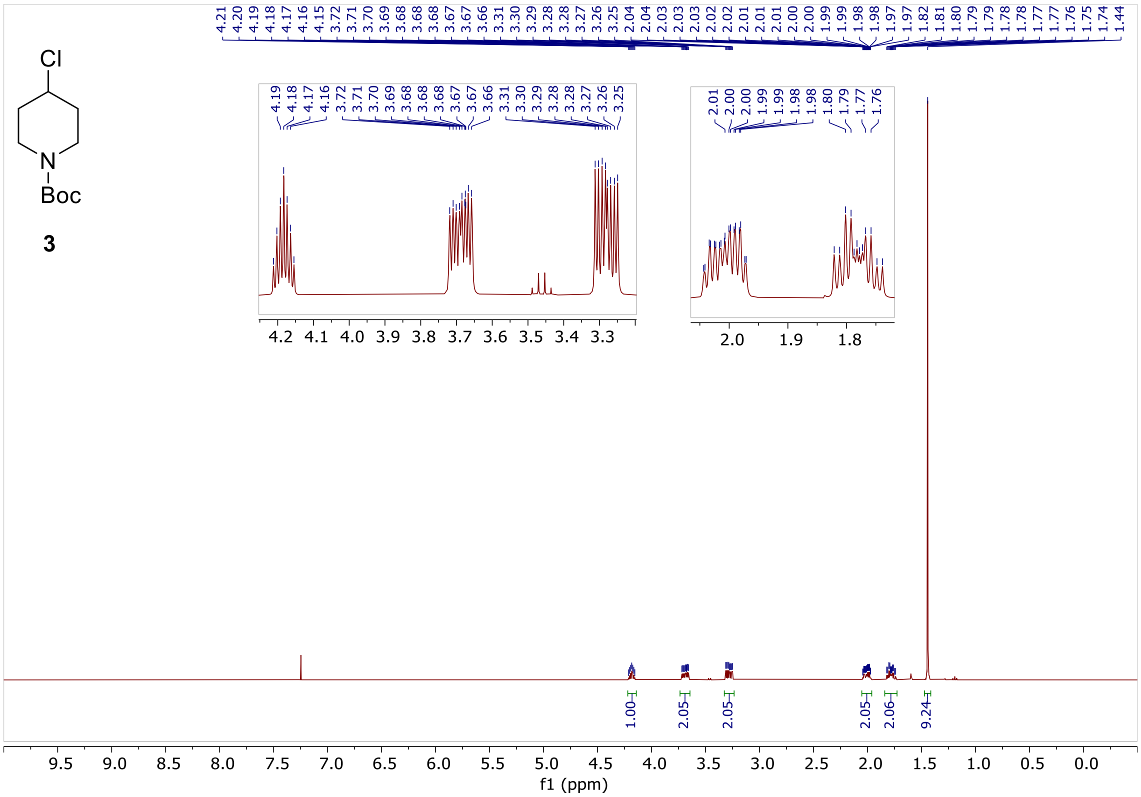

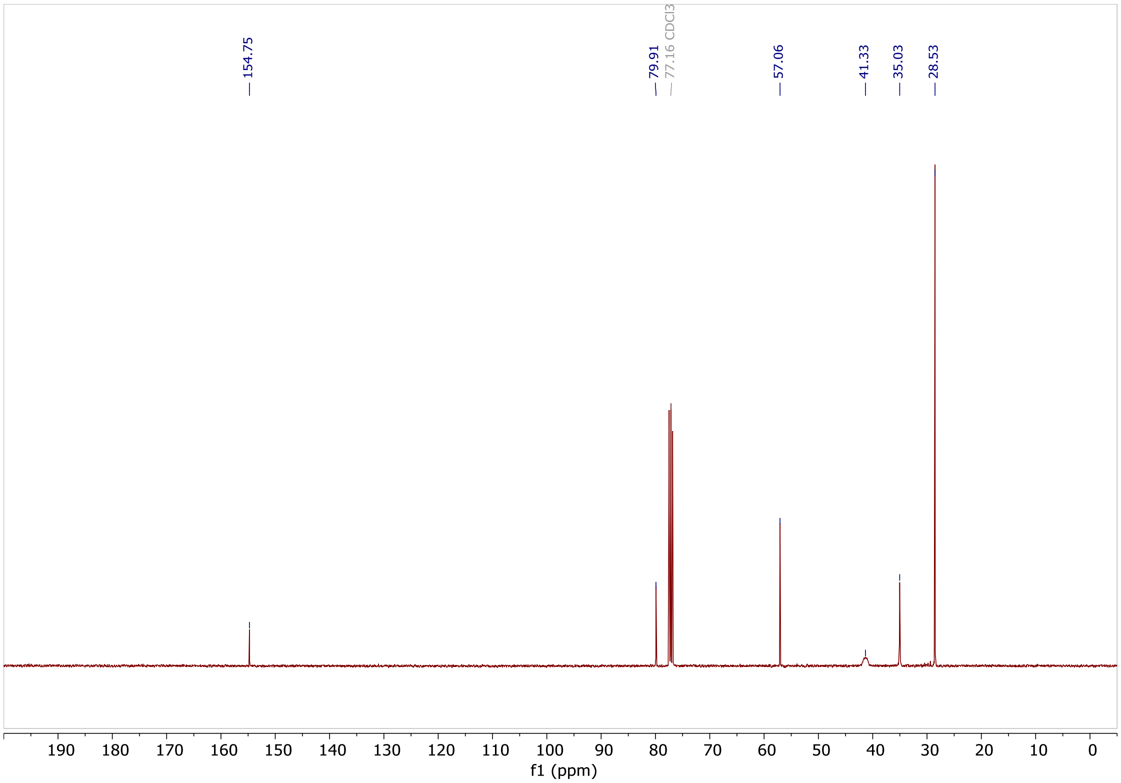

400 MHz ^{1}H NMR spectrum; $CDCl_3$ of **4**

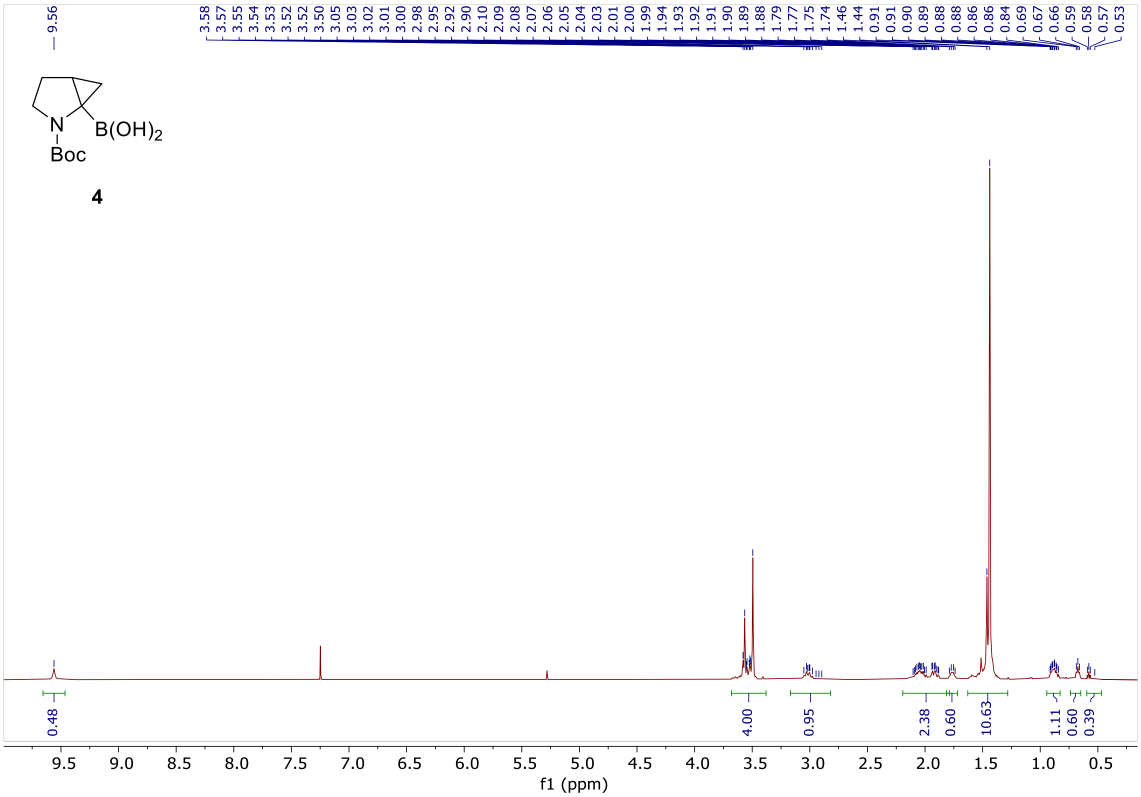

400 MHz ^{1}H NMR spectrum; 100.6 MHz ^{13}C NMR spectrum; 128.4 MHz ^{11}B NMR spectrum; acetone-d_6 of **1a**

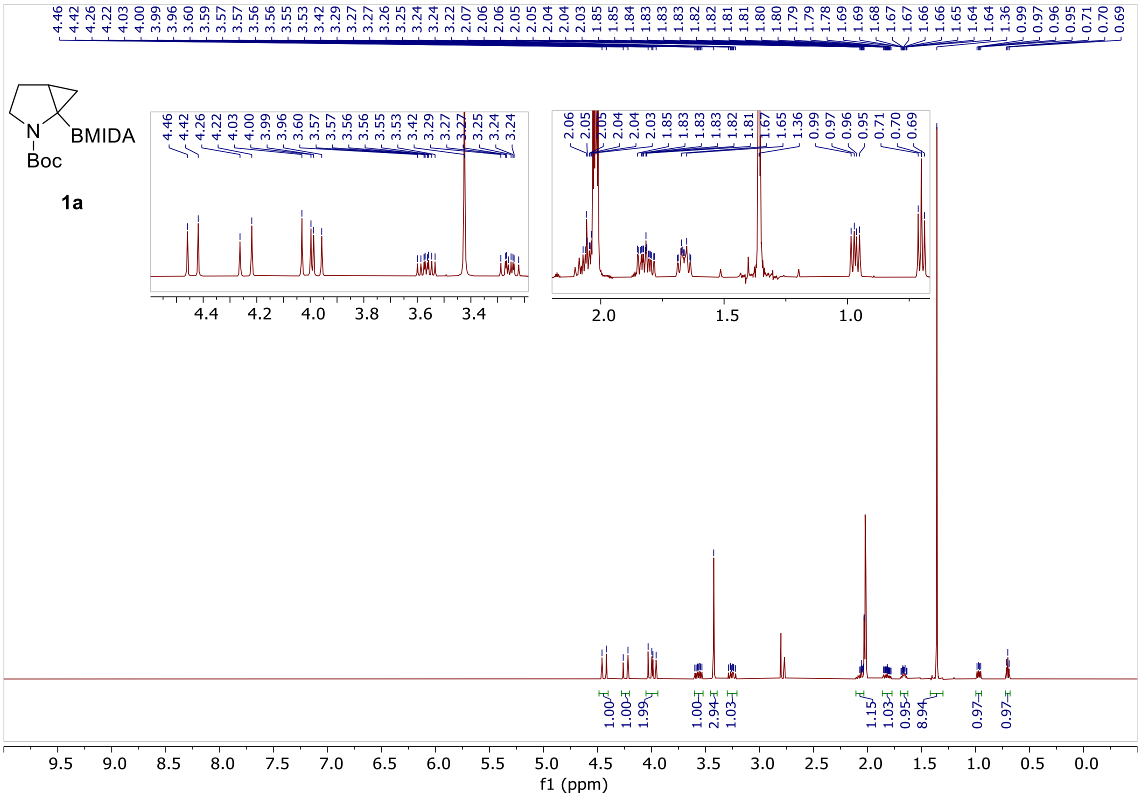

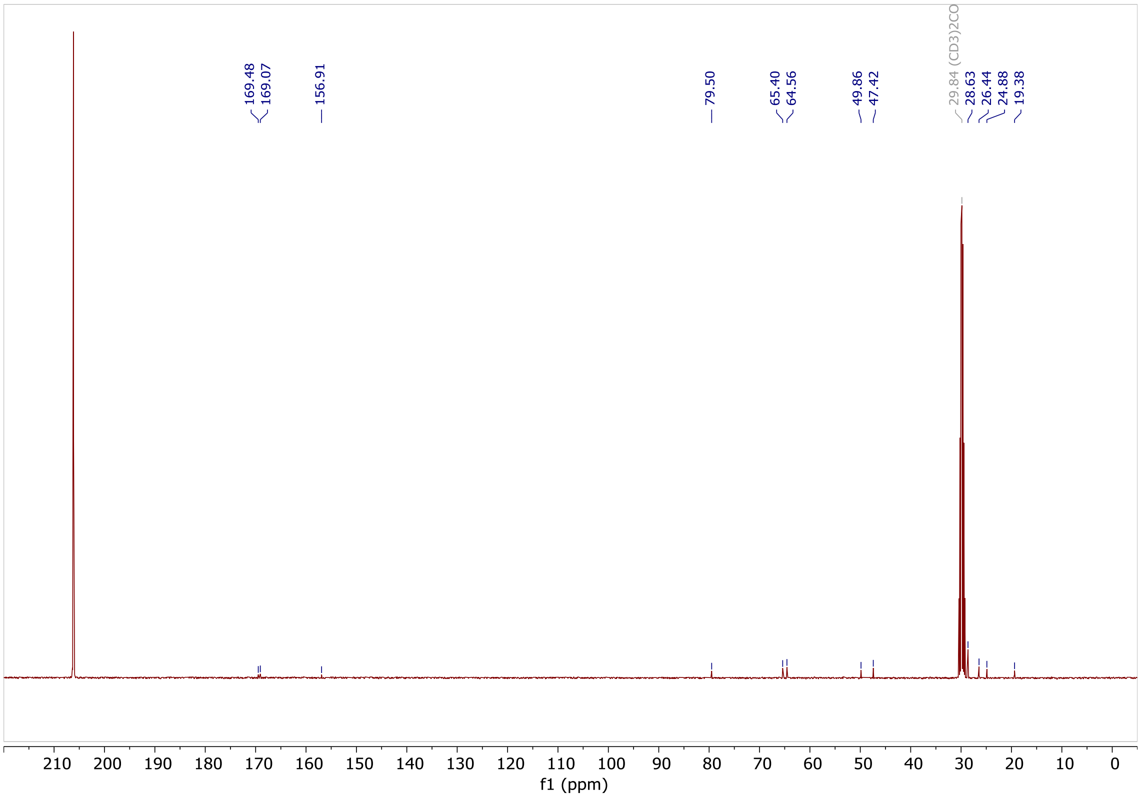

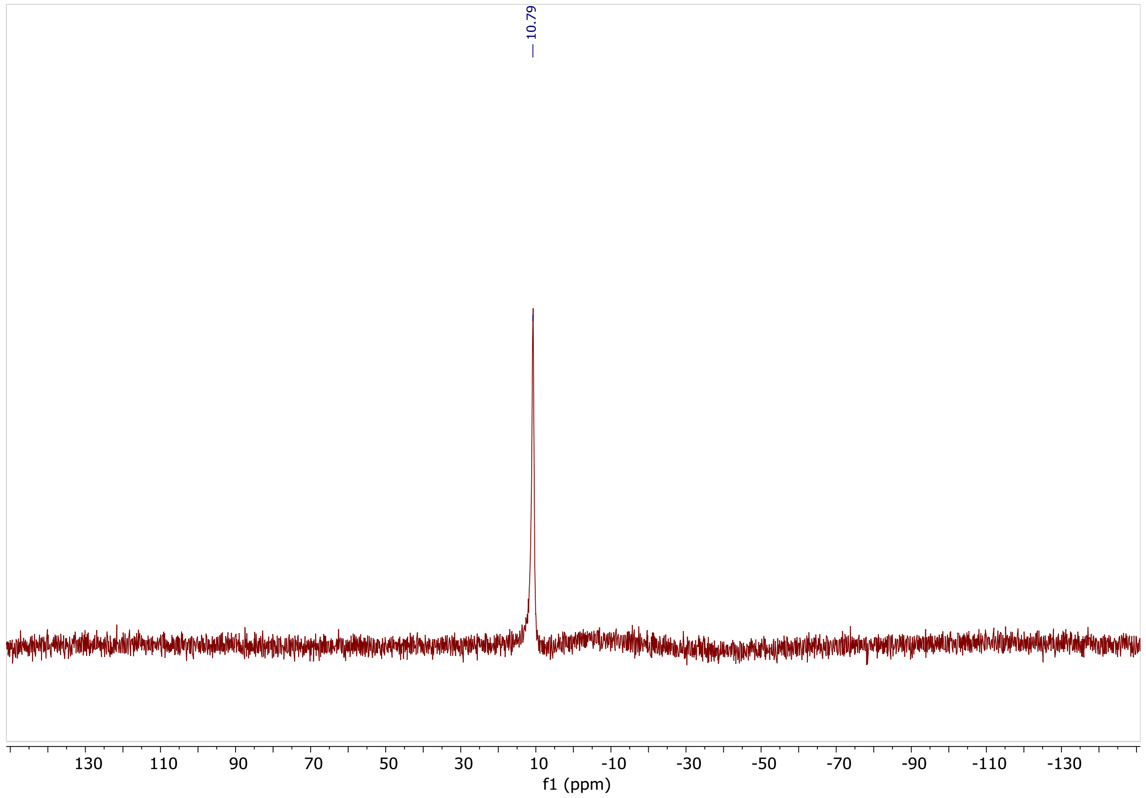
10.79
130
110
90
70
50
30
10
-10
-30
-50
-70
-90
-110
-130
f1 (ppm)

400 MHz ^{1}H NMR spectrum; 100.6 MHz ^{13}C NMR spectrum; DMSO-d_6 of (*S*,*S*)-**S1**

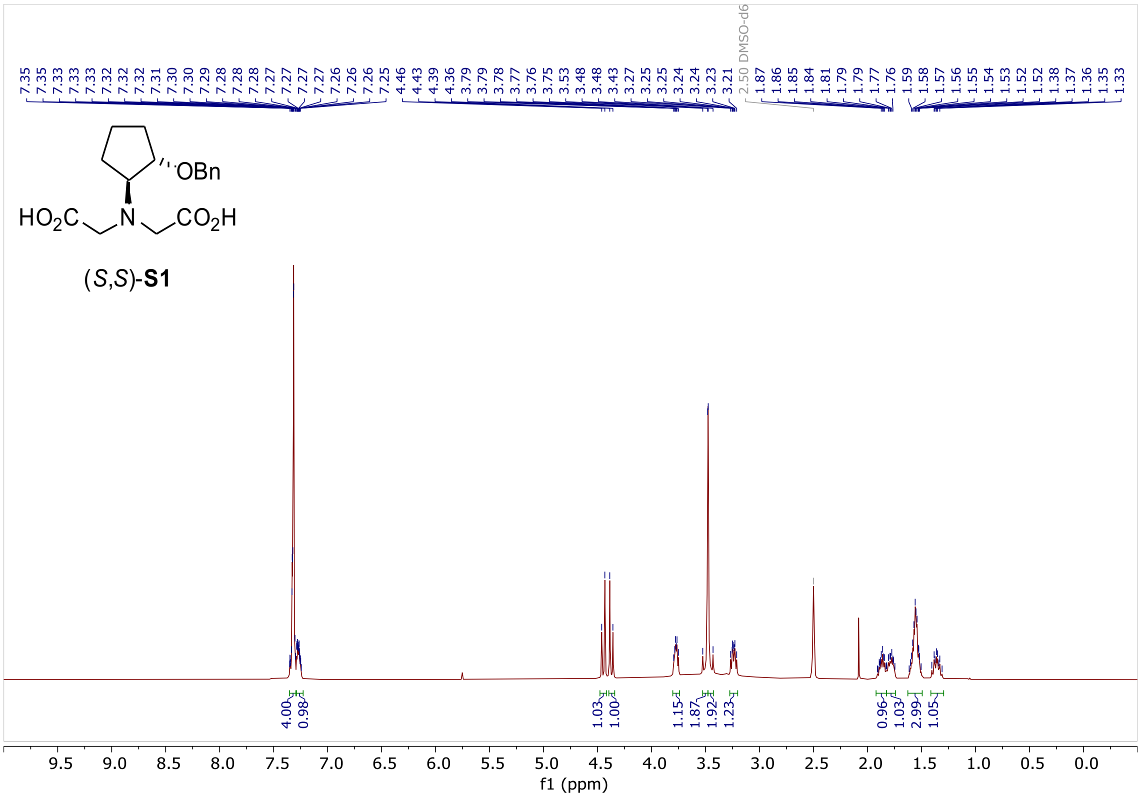

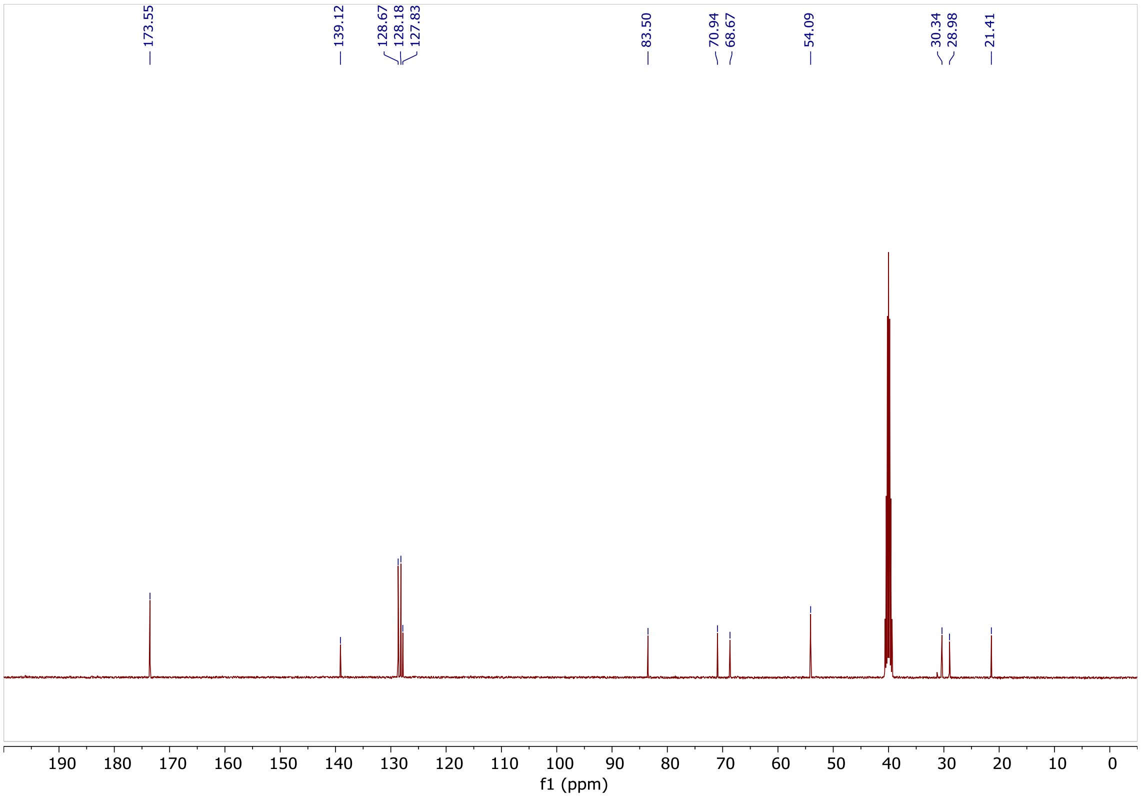

400 MHz ^{1}H NMR spectrum; 100.6 MHz ^{13}C NMR spectrum; $CDCl_3$ of (*S*,*R*,*S*,*S*)-**1a'**

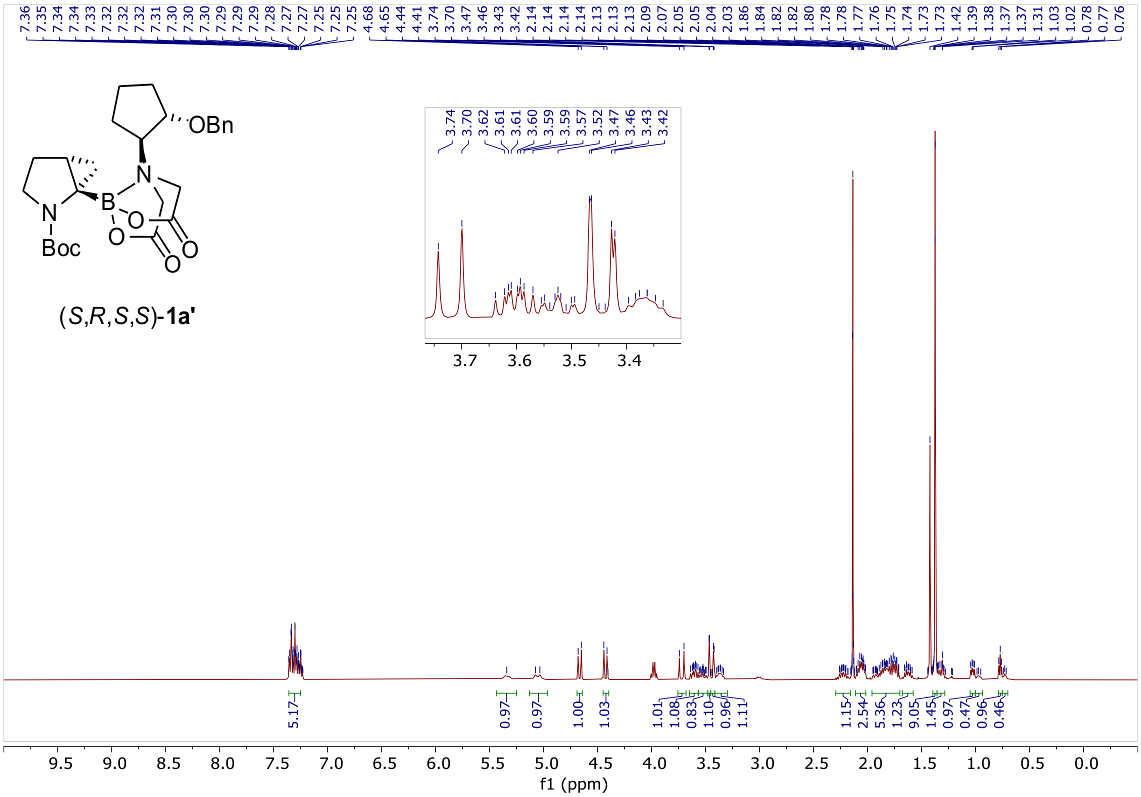

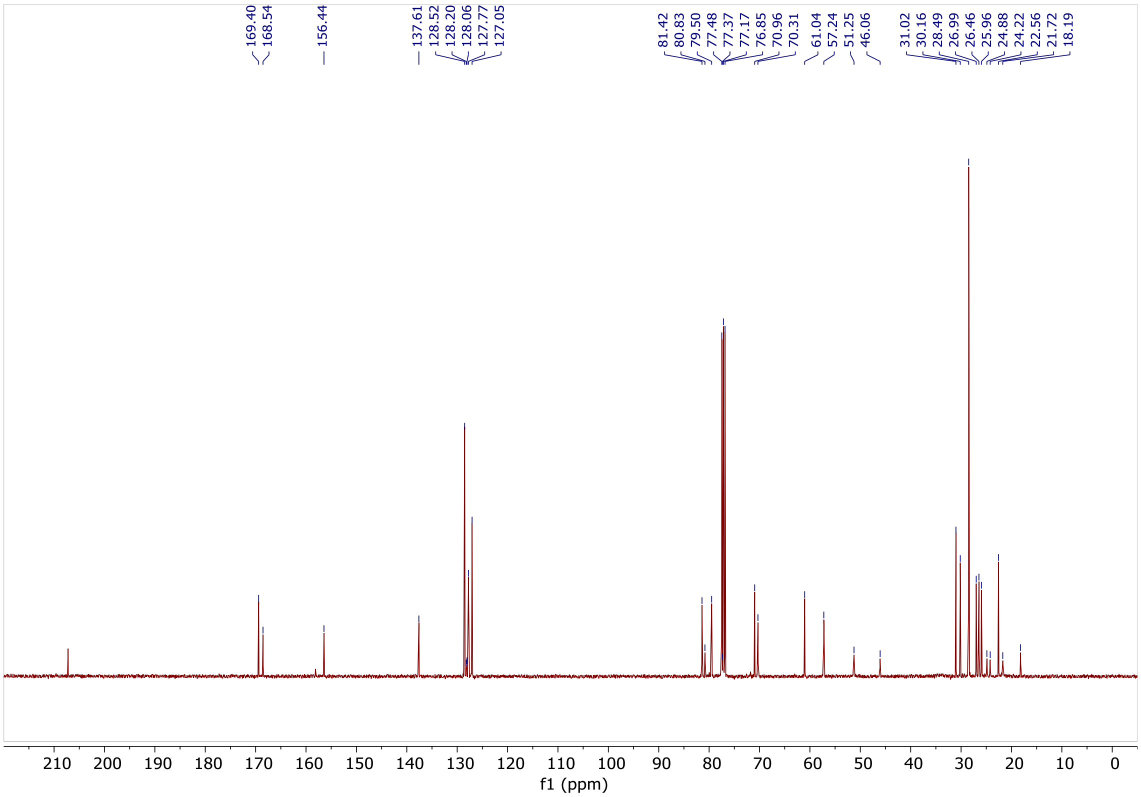

400 MHz ^{1}H NMR spectrum; 100.6 MHz ^{13}C NMR spectrum; $CDCl_3$ of (*R*,*S*,*S*,*S*)-**1a''**

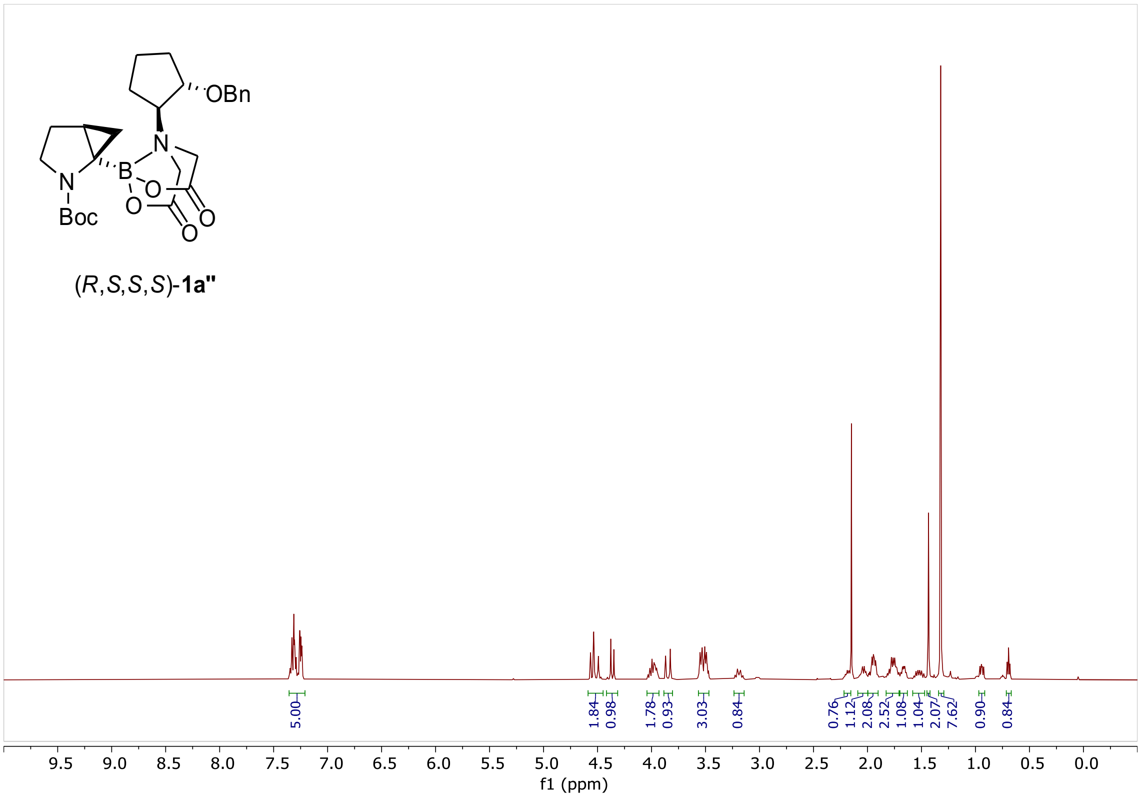

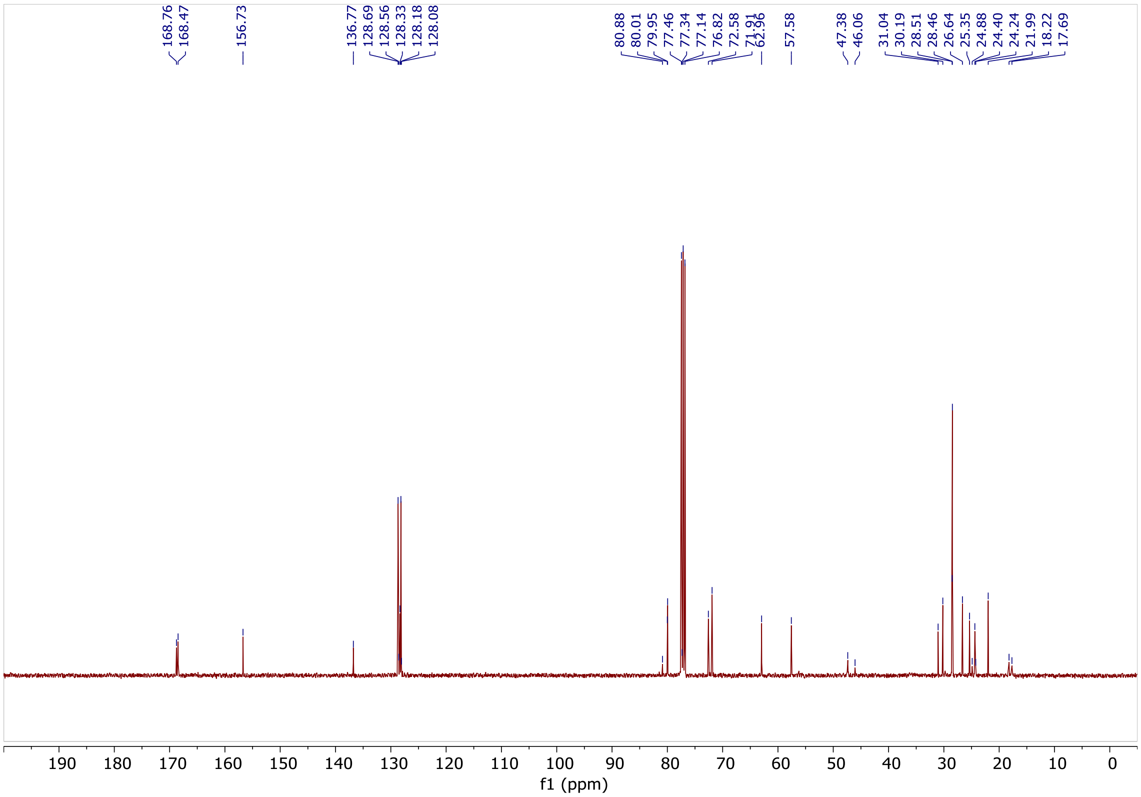

400 MHz ^{1}H NMR spectrum; 100.6 MHz ^{13}C NMR spectrum; $CDCl_3$ of **6**

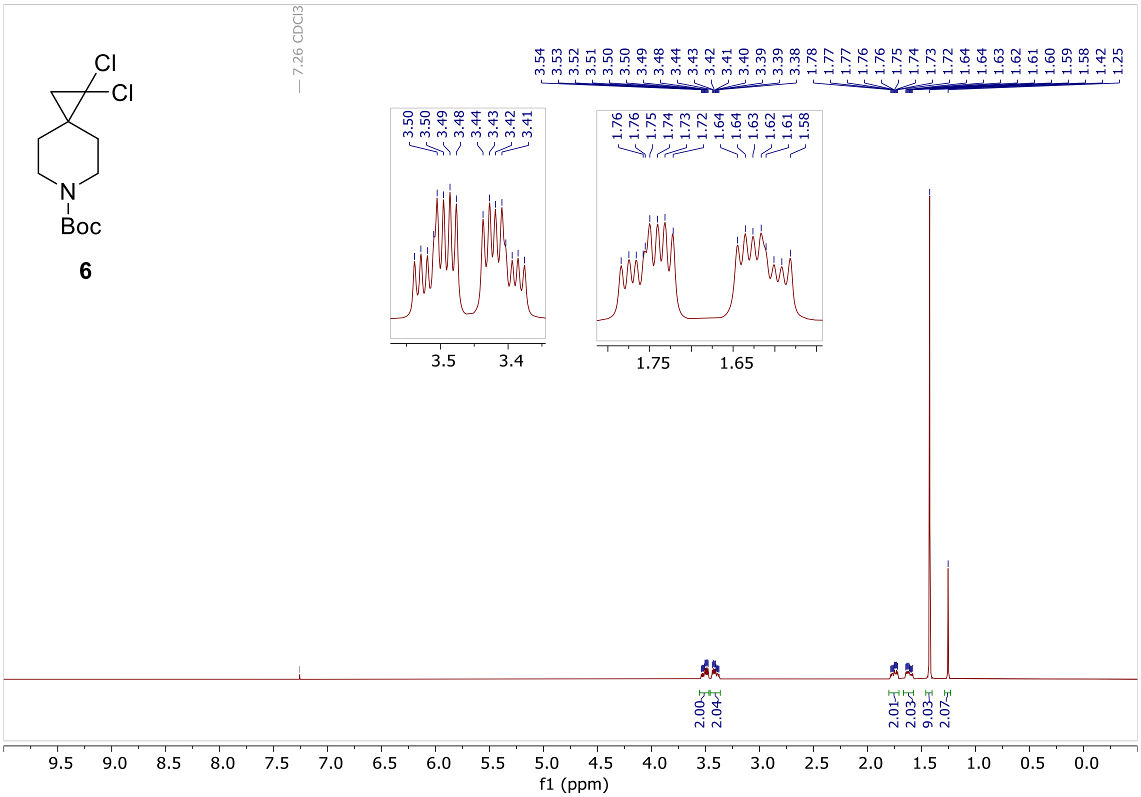

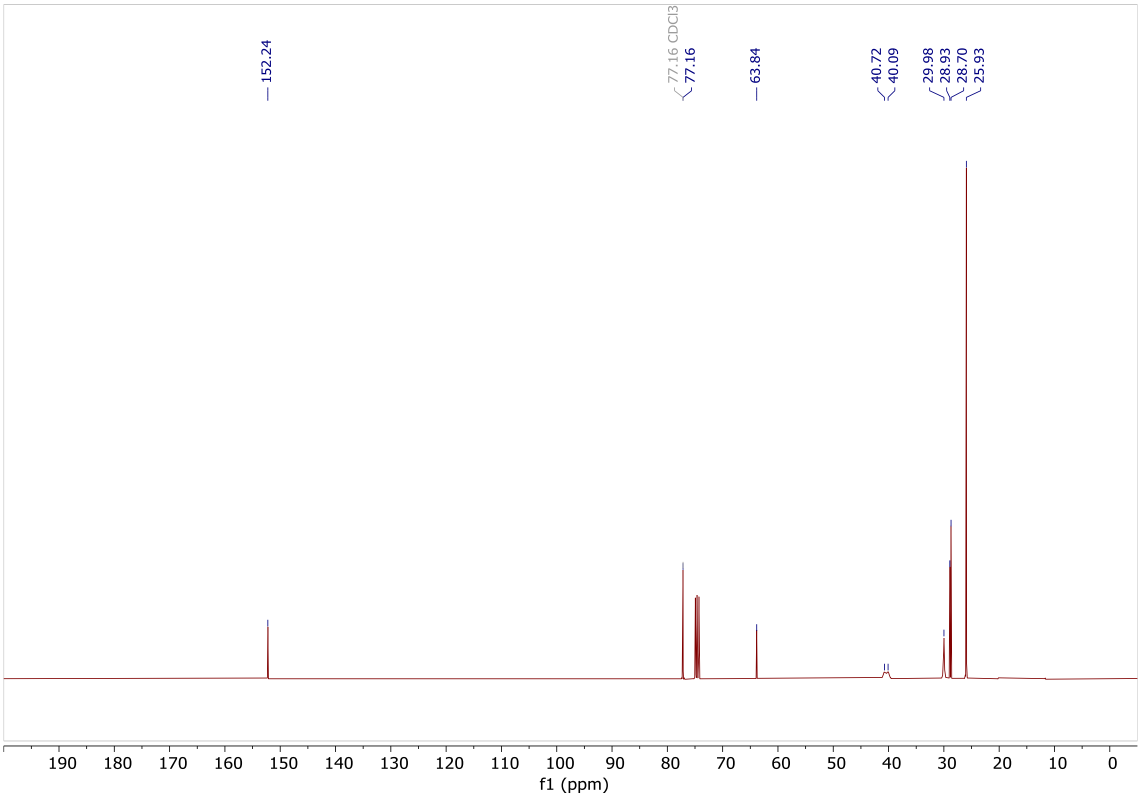

400 MHz ^{1}H NMR spectrum; $CDCl_3$ of **7**

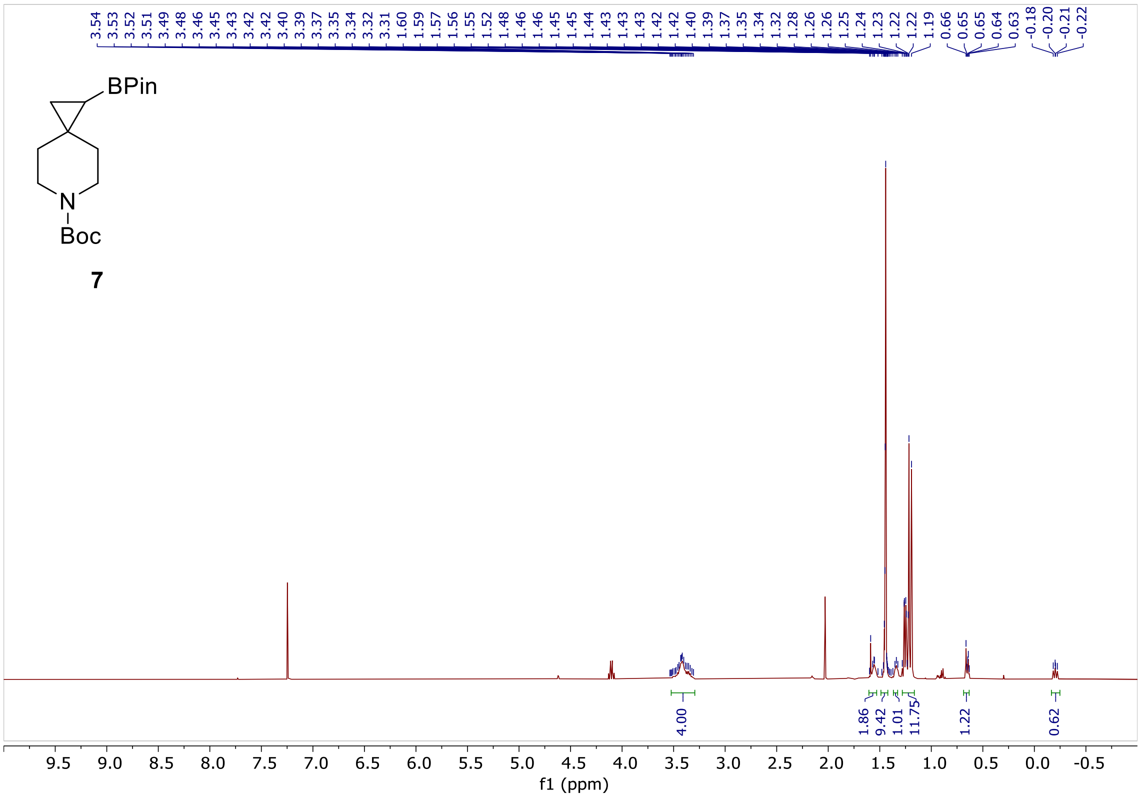

400 MHz ^{1}H NMR spectrum; 100.6 MHz ^{13}C NMR spectrum; 128.4 MHz ^{11}B NMR spectrum; acetone-*d*$_6$ of **1b**

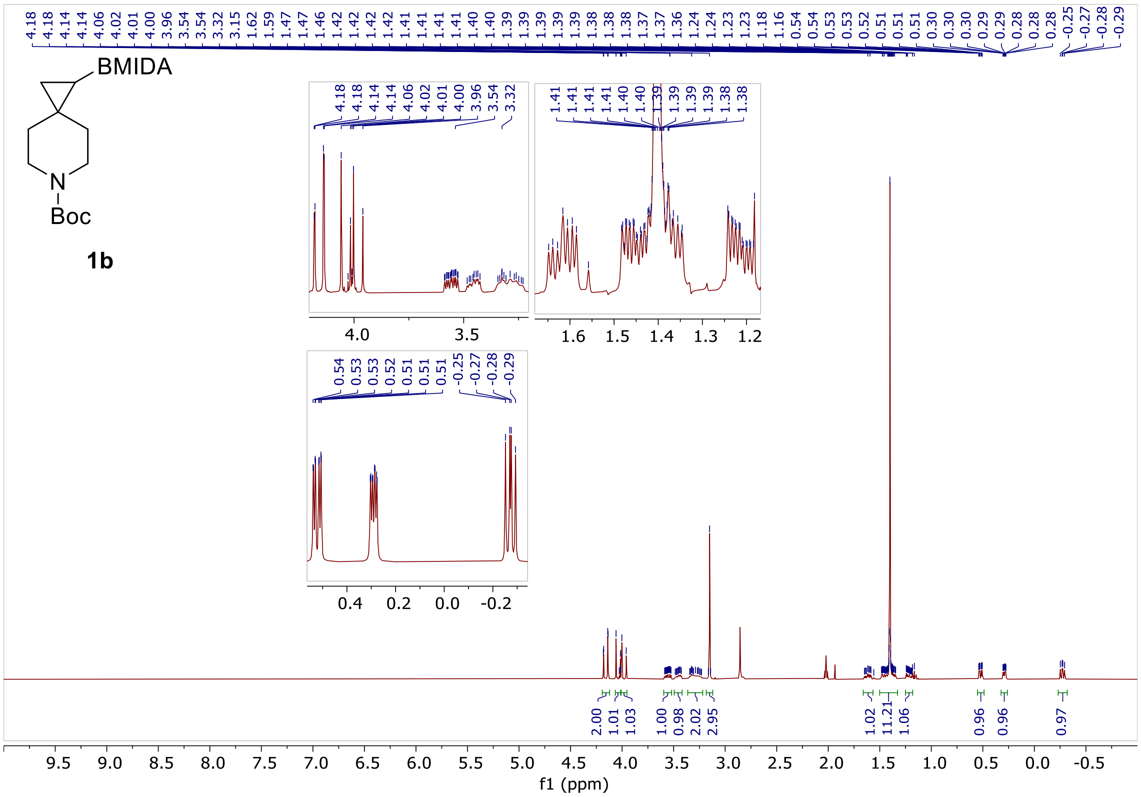

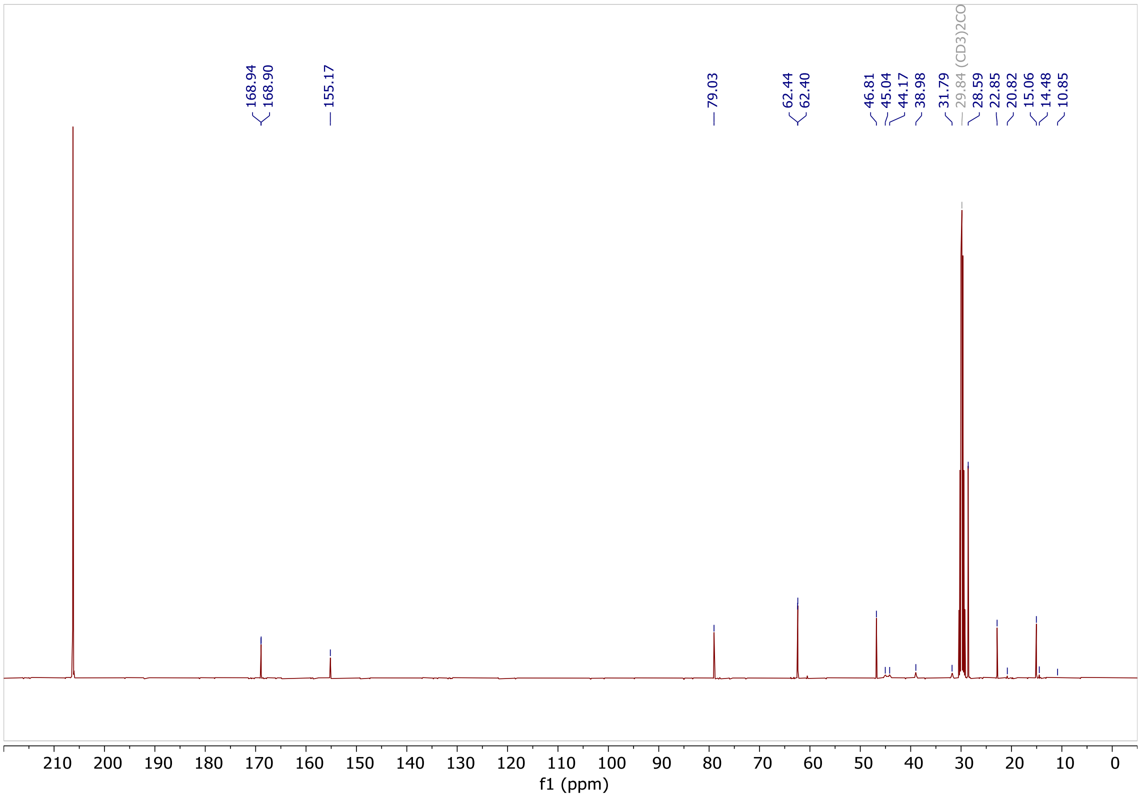

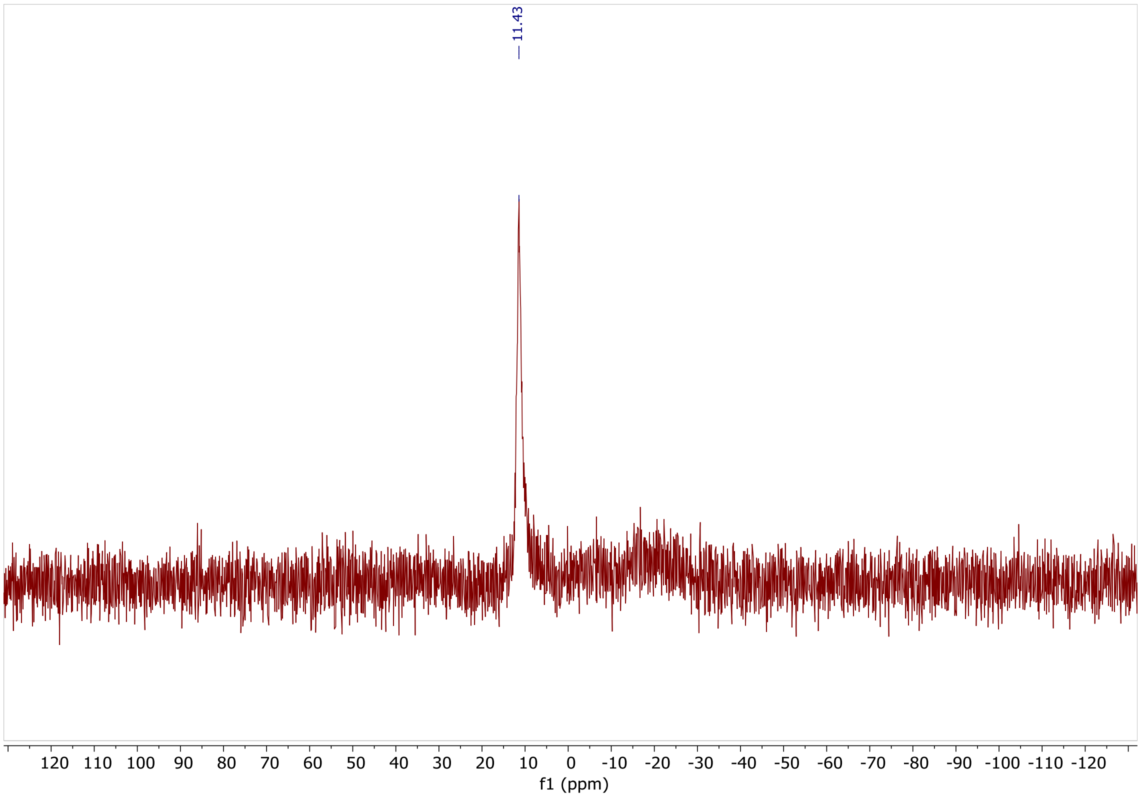
11.43
120
110
100
90
80
70
60
50
40
30
20
10
0
-10
-20
-30
-40
-50
-60
-70
-80
-90
-100
-110
-120
f1 (ppm)

400 MHz ^{1}H NMR spectrum; 100.6 MHz ^{13}C NMR spectrum; 128.4 MHz ^{11}B NMR spectrum; DMSO-d_6 of **S2**

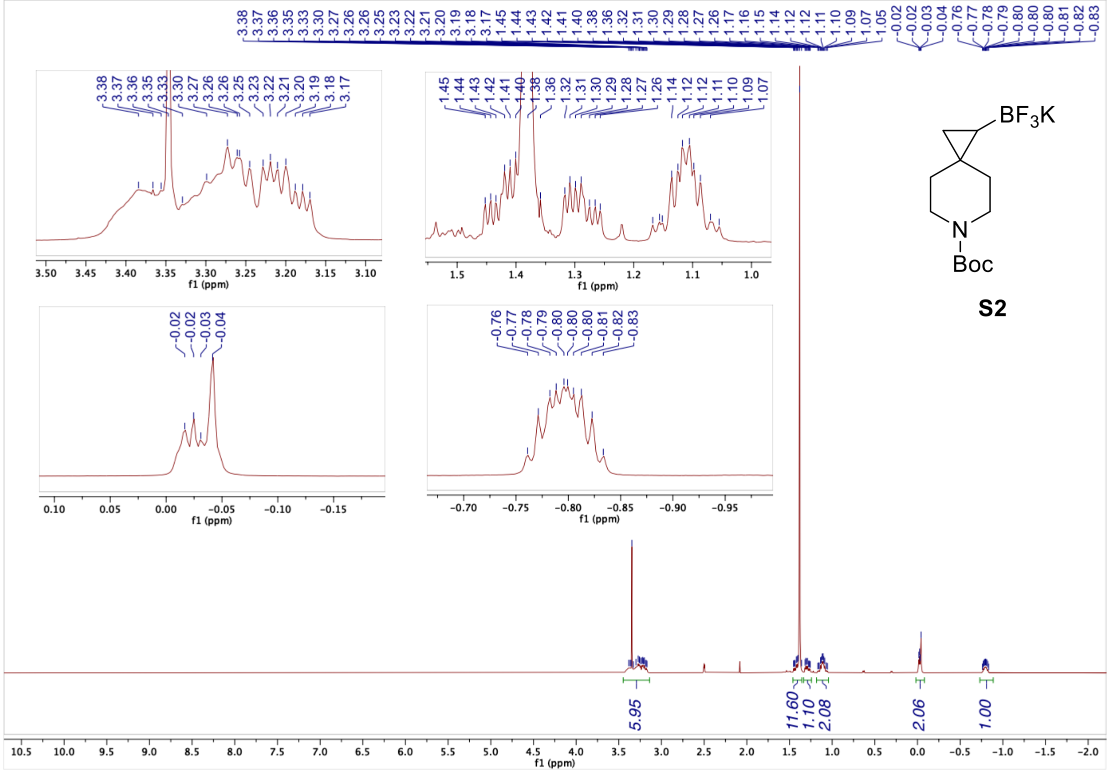

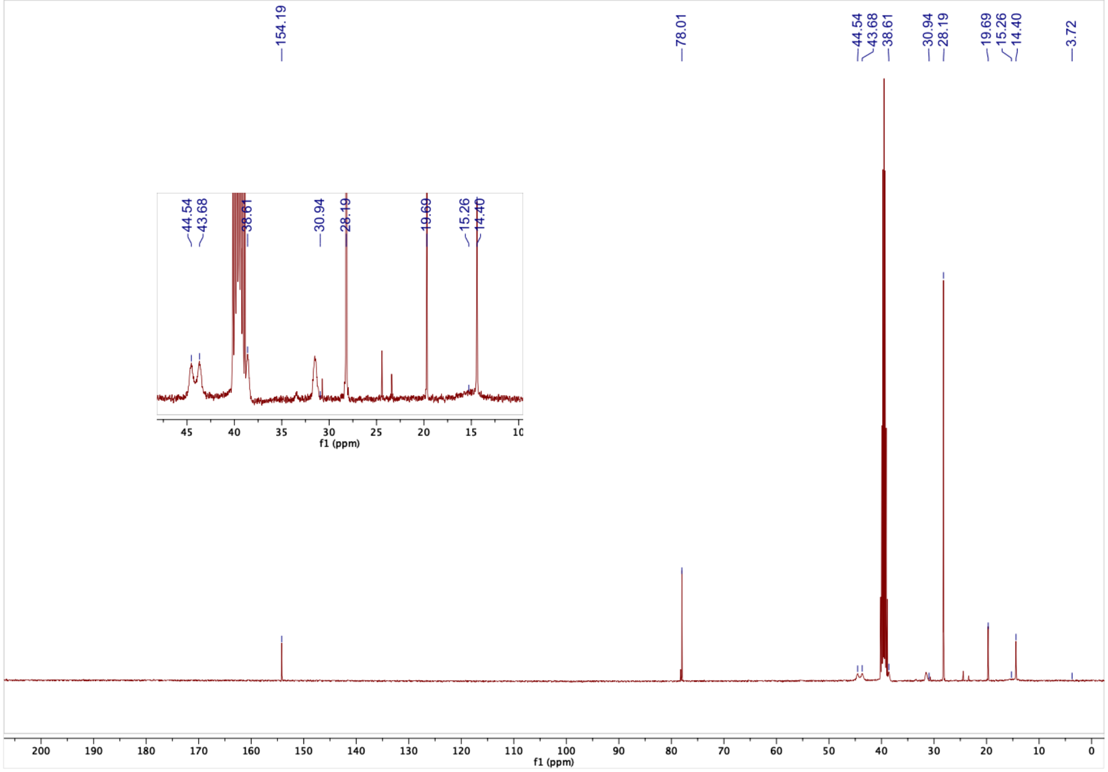

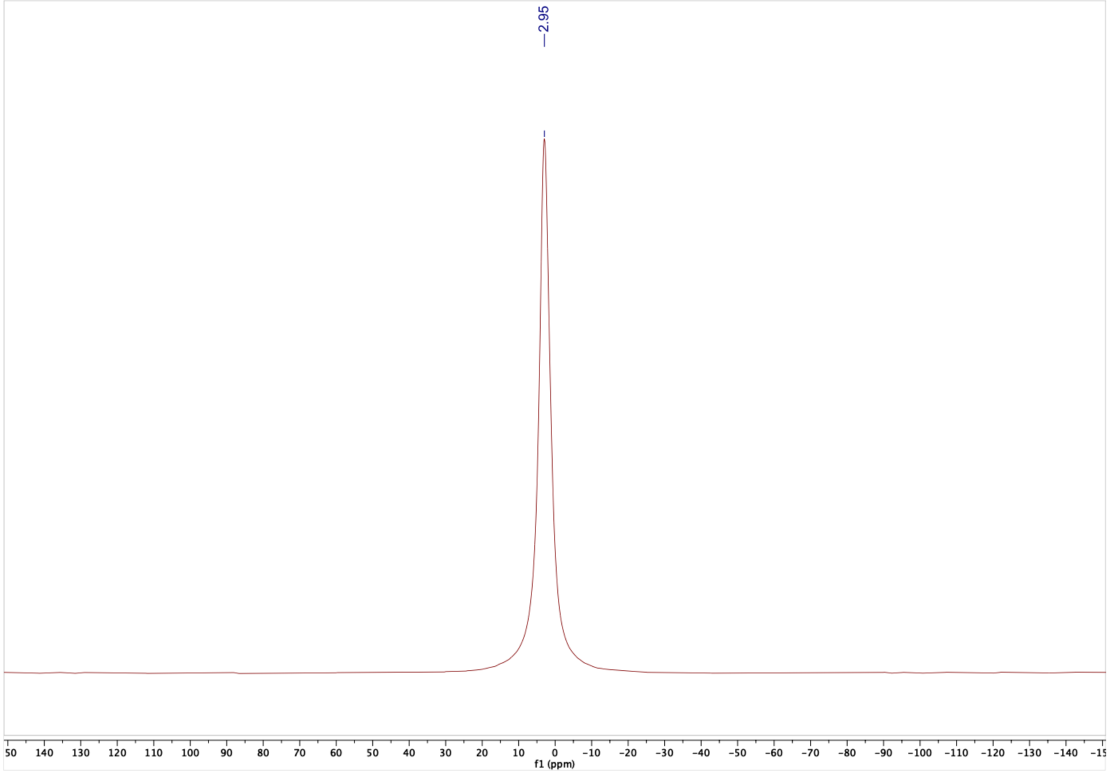
2.95
150
140
130
120
110
100
90
80
70
60
50
40
30
20
10
0
-10
-20
-30
-40
-50
-60
-70
-80
-90
-100
-110
-120
-130
-140
-150
f1 (ppm)

400 MHz ^{1}H NMR spectrum; $CDCl_3$ of **S3**

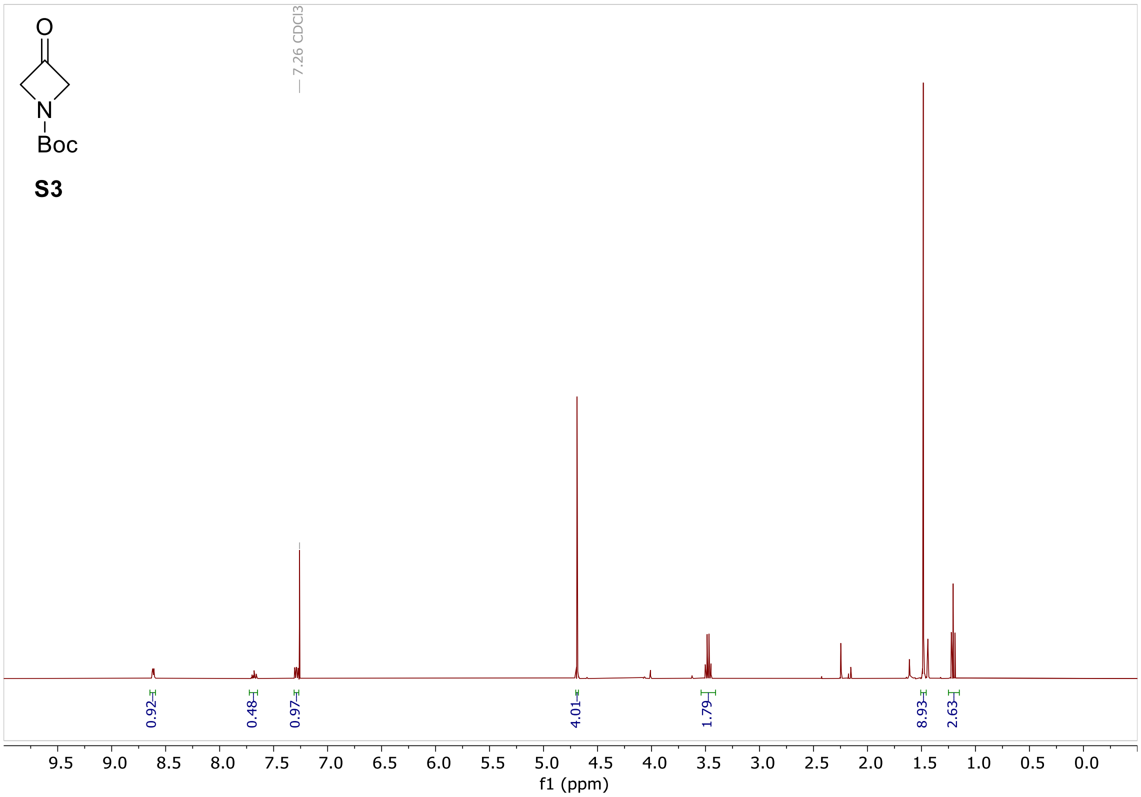

400 MHz ^{1}H NMR spectrum; $CDCl_3$ of **S4**

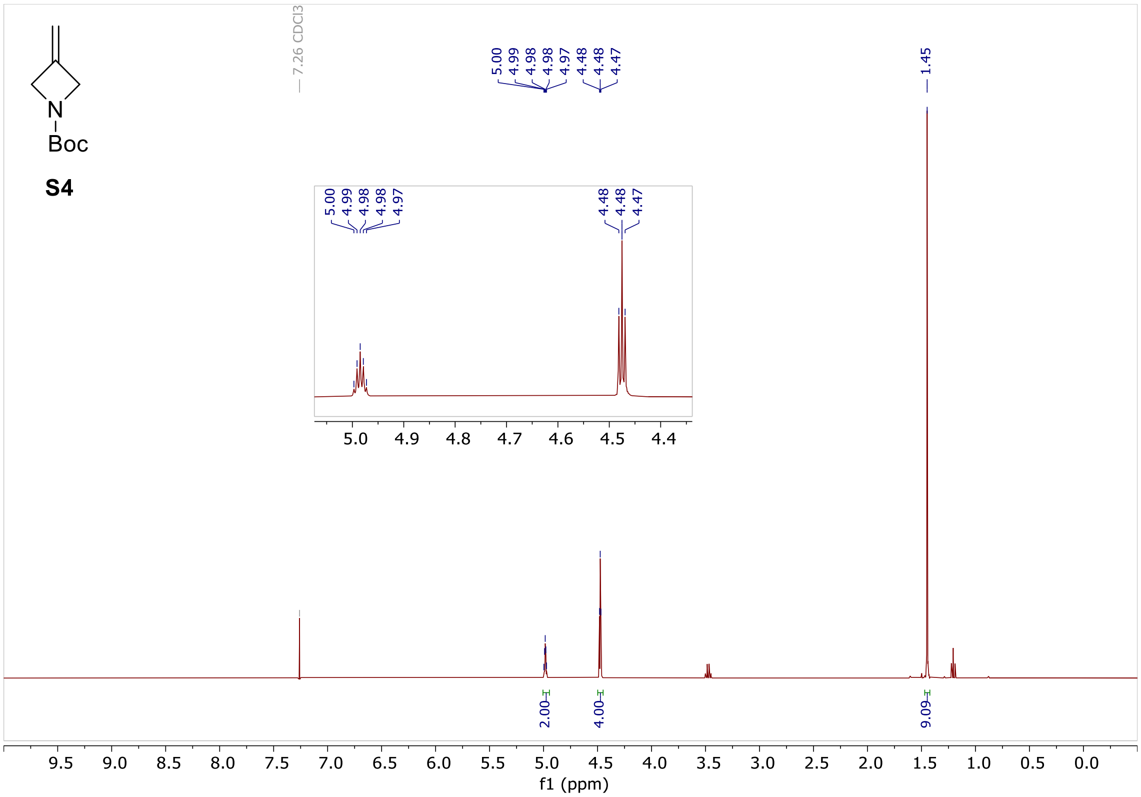

400 MHz ^{1}H NMR spectrum; 100.6 MHz ^{13}C NMR spectrum; $CDCl_3$ of **S5**

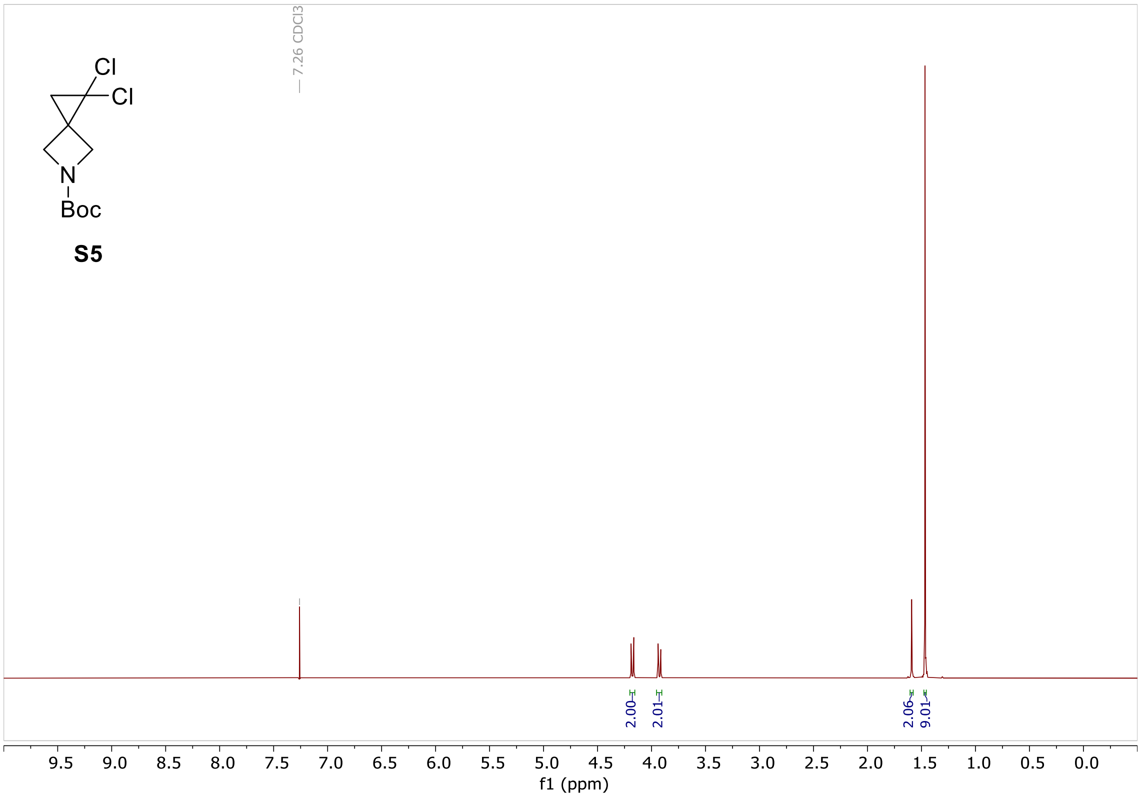

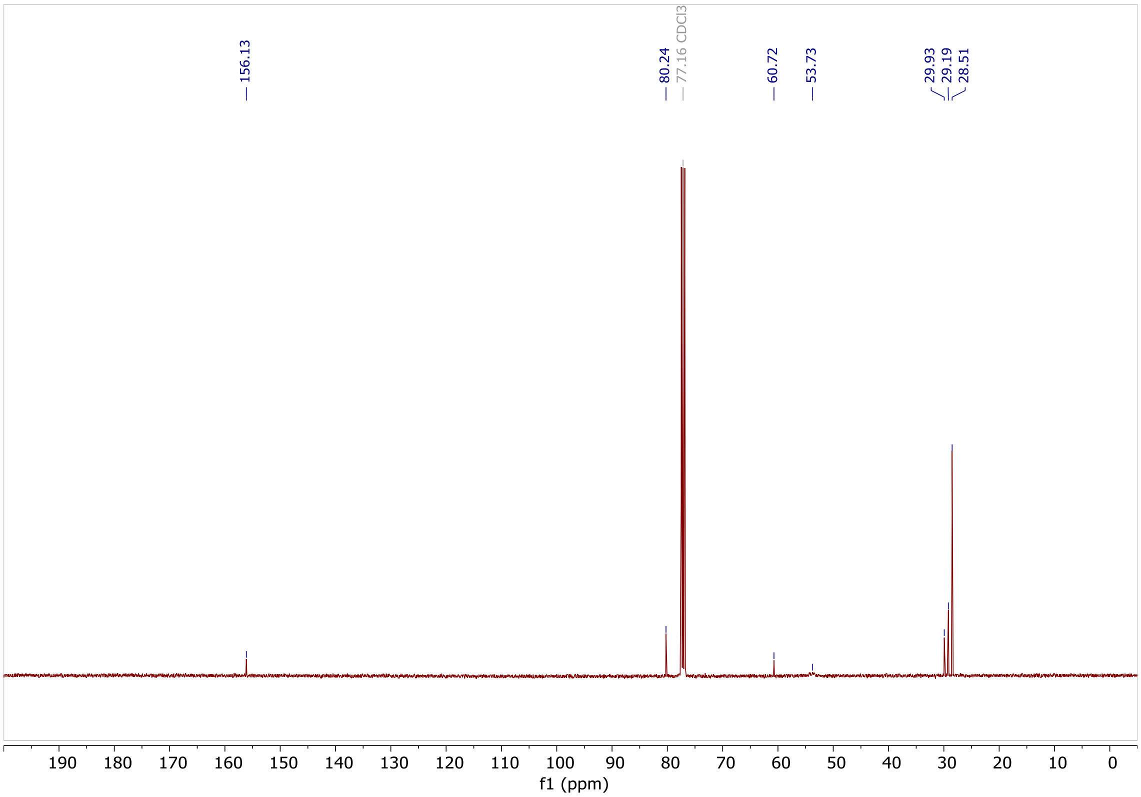

400 MHz ^{1}H NMR spectrum; 100.6 MHz ^{13}C NMR spectrum; $CDCl_3$ of **S6**

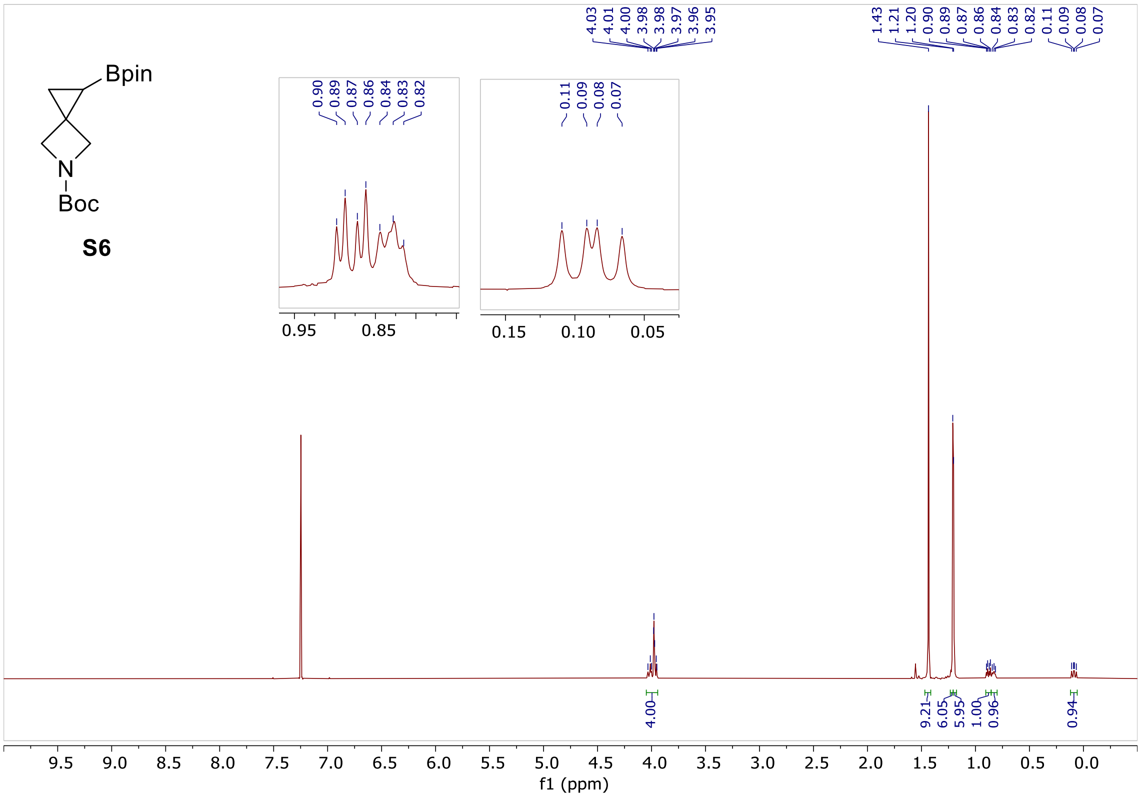

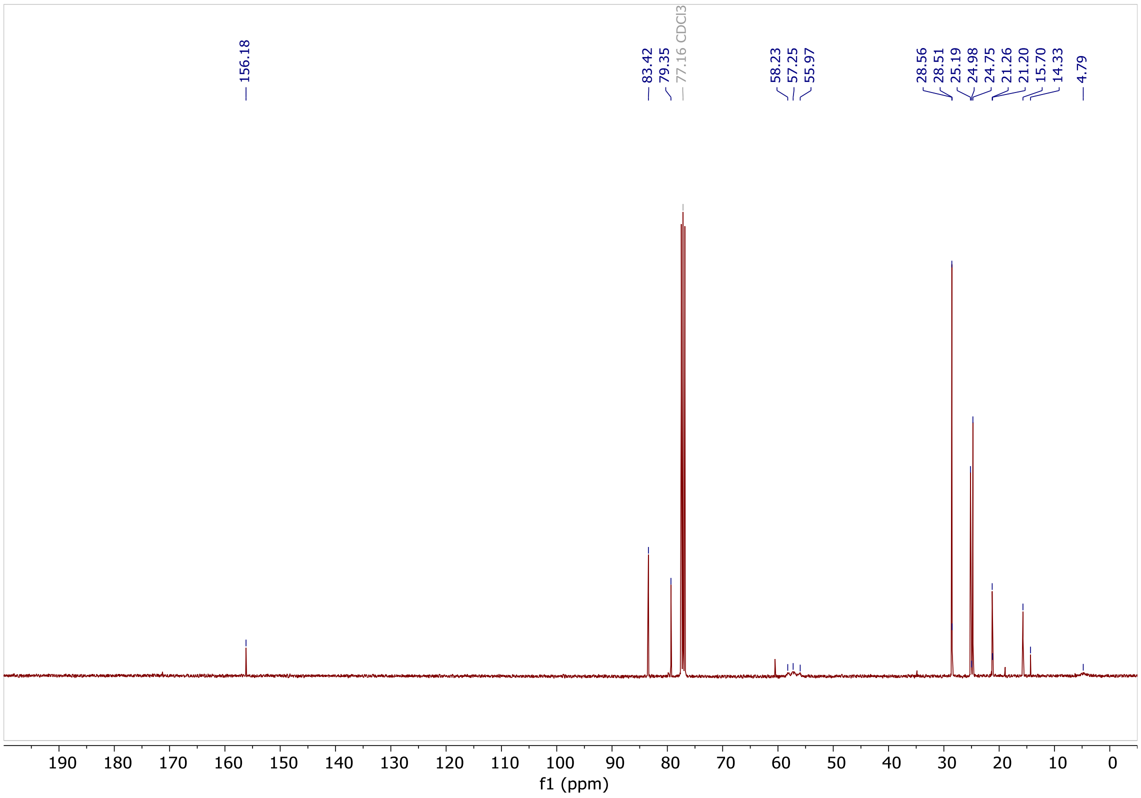

400 MHz ^{1}H NMR spectrum; 100.6 MHz ^{13}C NMR spectrum; 128.4 MHz ^{11}B NMR spectrum; acetone-d_6 of **1c**

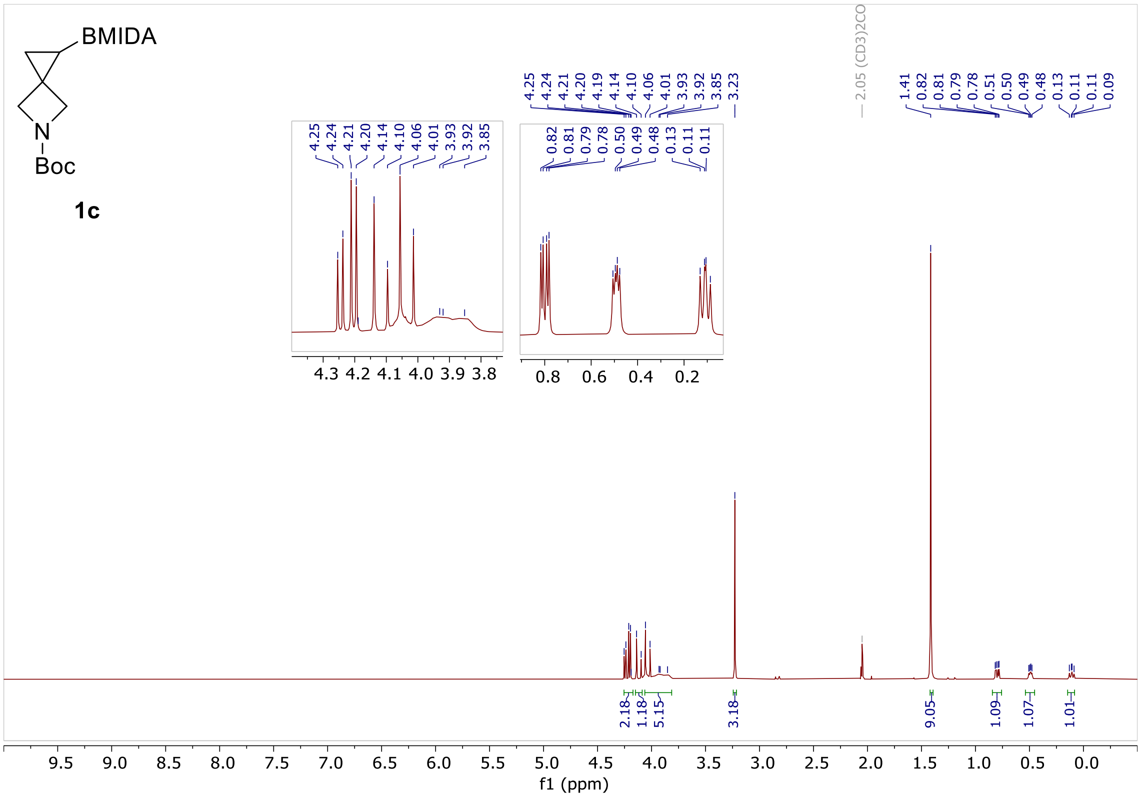

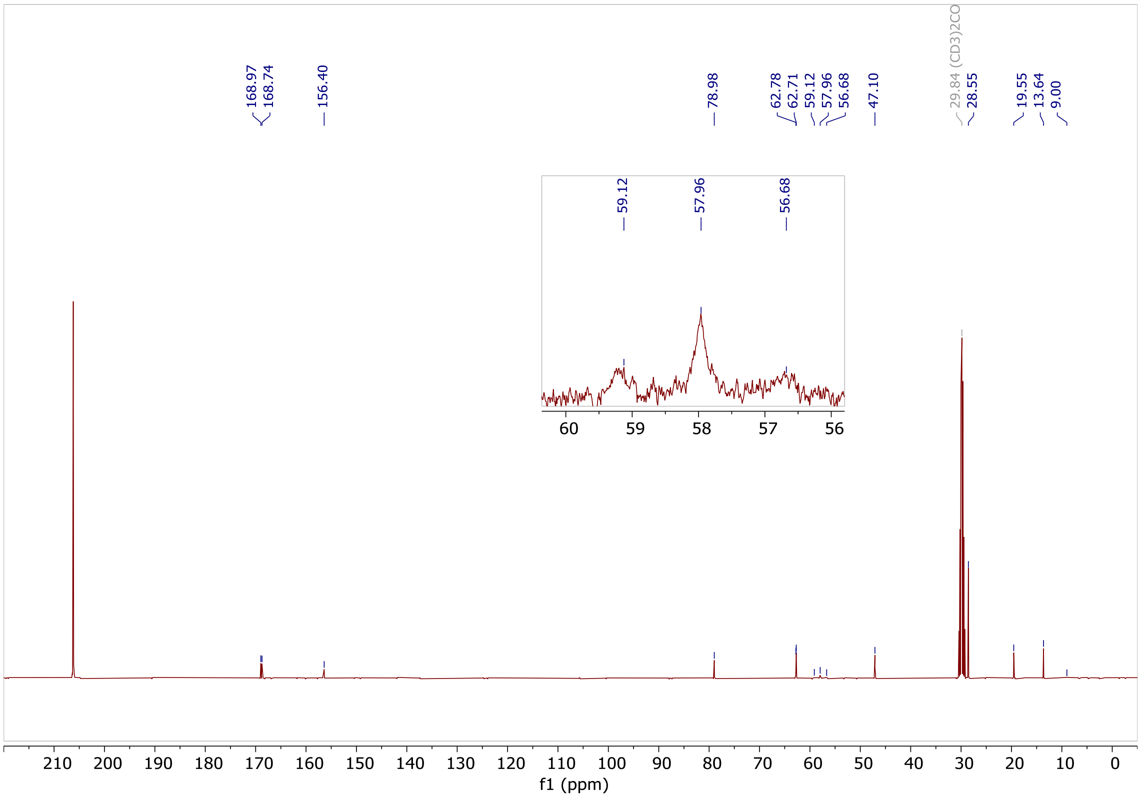

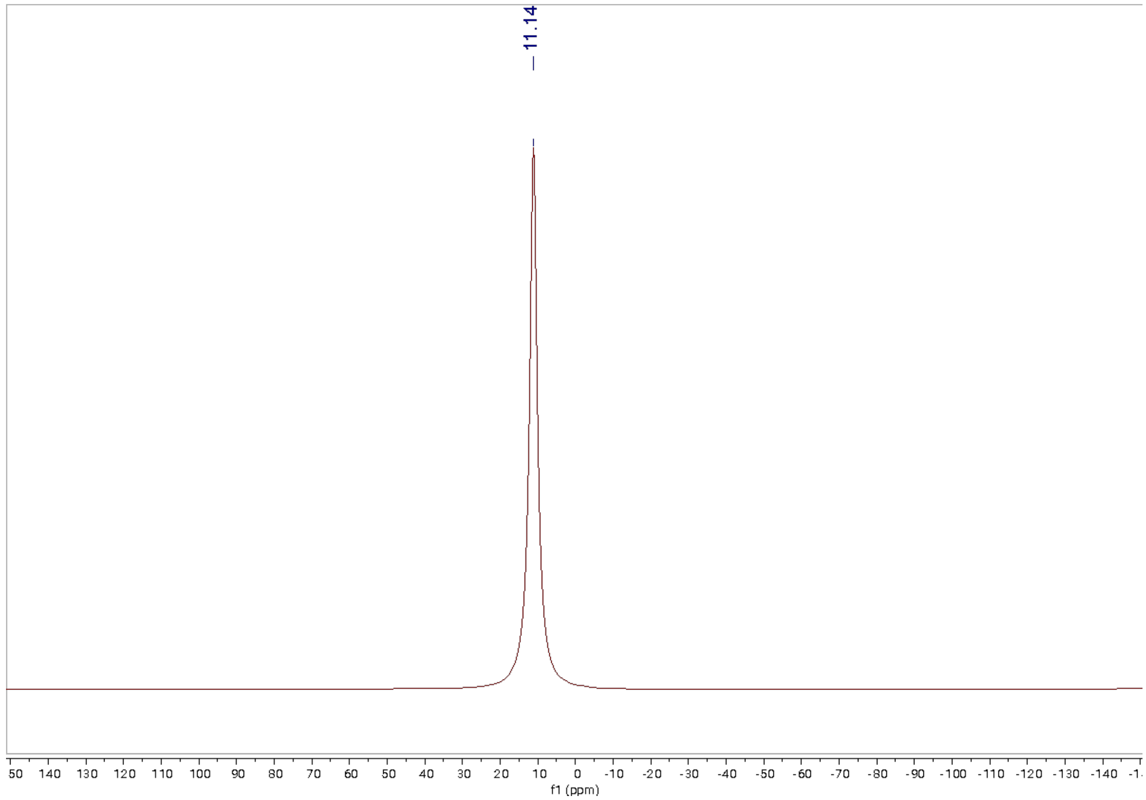
11.14
50
140
130
120
110
100
90
80
70
60
50
40
30
20
10
0
-10
-20
-30
-40
-50
-60
-70
-80
-90
-100
-110
-120
-130
-140
f1 (ppm)

400 MHz ^{1}H NMR spectrum; 100.6 MHz ^{13}C NMR spectrum; 128.4 MHz ^{11}B NMR spectrum; DMSO-d_6 of **S7**

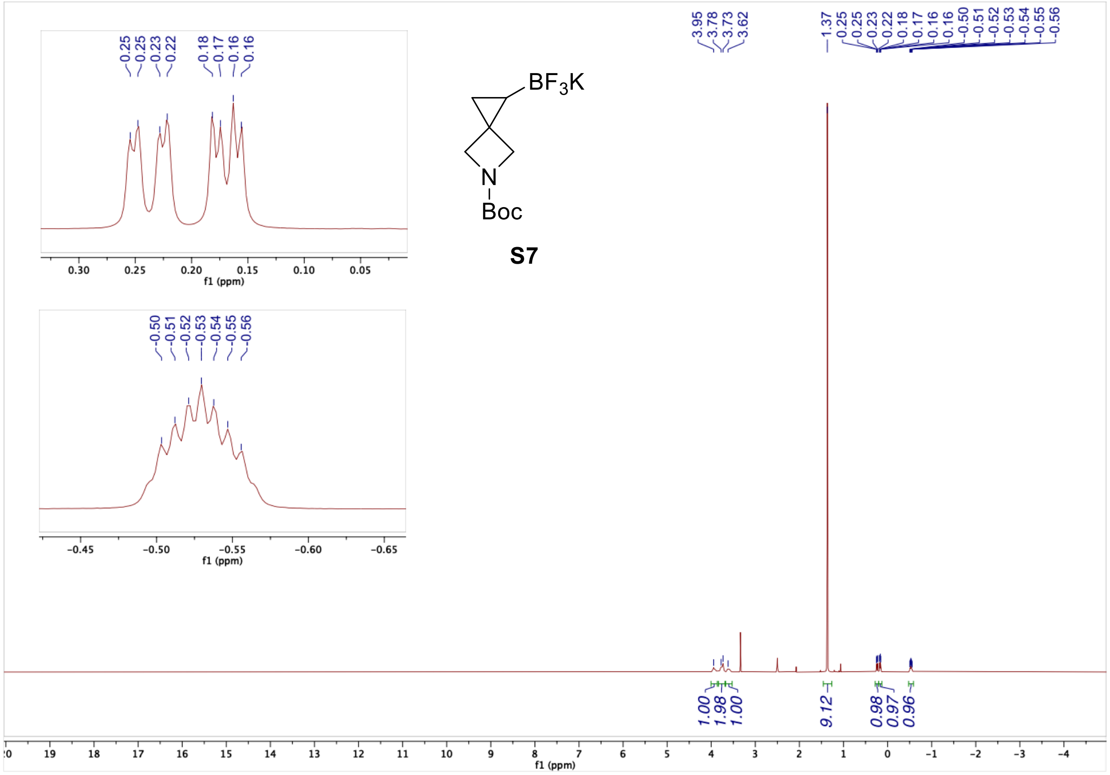

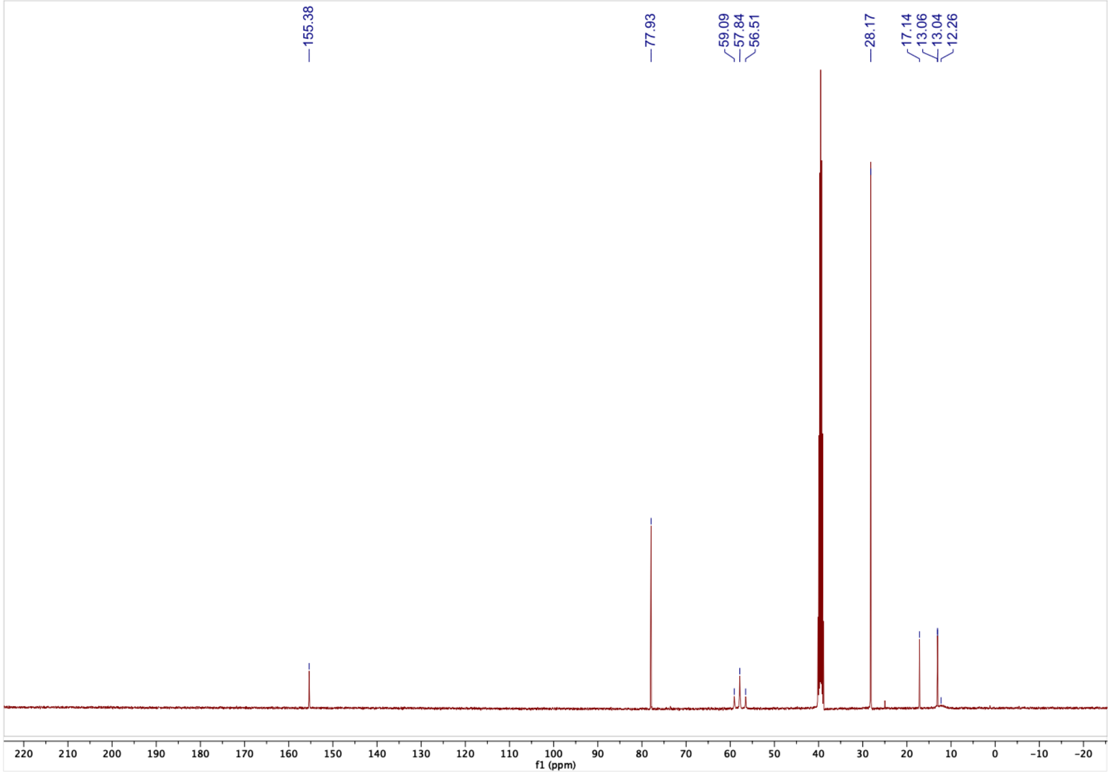

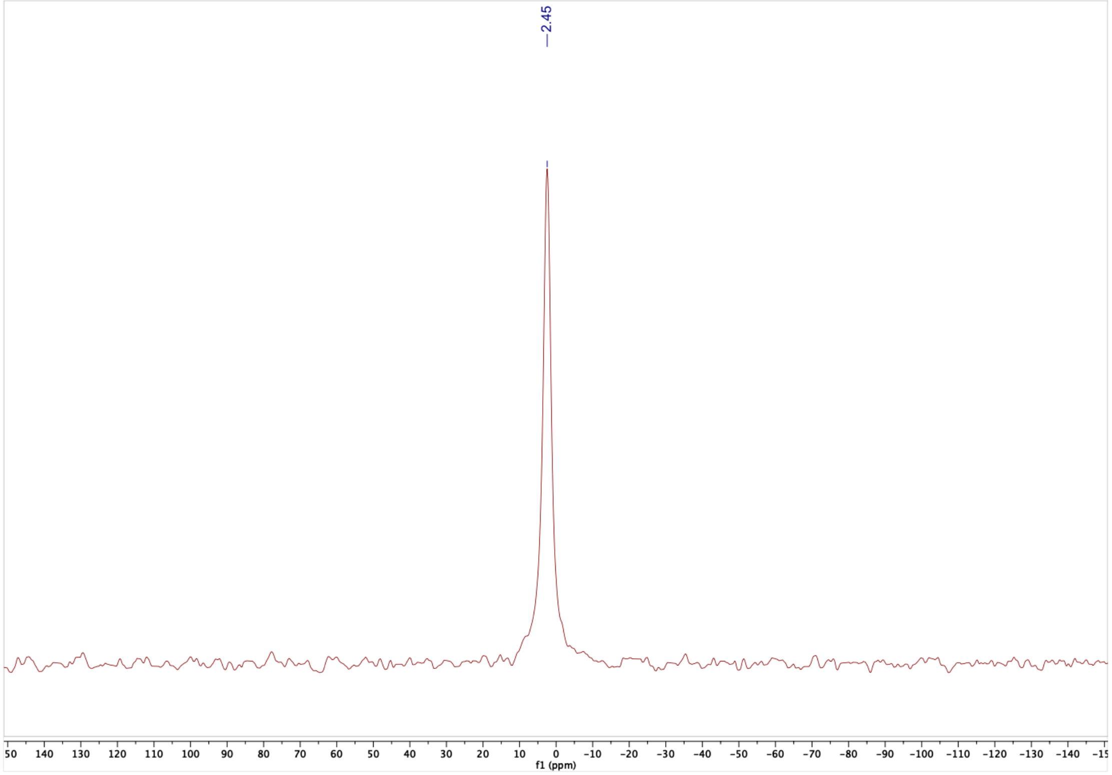
2.45
150
140
130
120
110
100
90
80
70
60
50
40
30
20
10
0
-10
-20
-30
-40
-50
-60
-70
-80
-90
-100
-110
-120
-130
-140
-150
f1 (ppm)

400 MHz ^{1}H NMR spectrum; $CDCl_3$ of **S8**

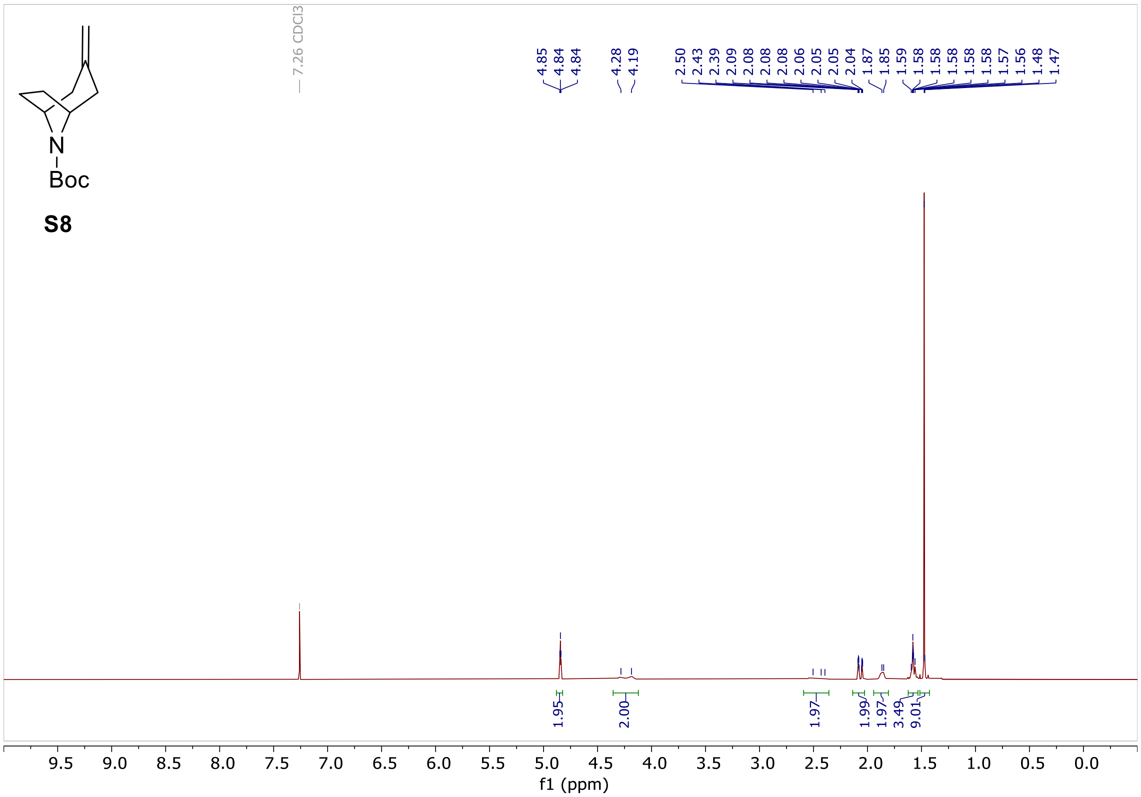

400 MHz ^1H NMR spectrum; 100.6 MHz ^{13}C NMR spectrum; $CDCl_3$ of **S9**

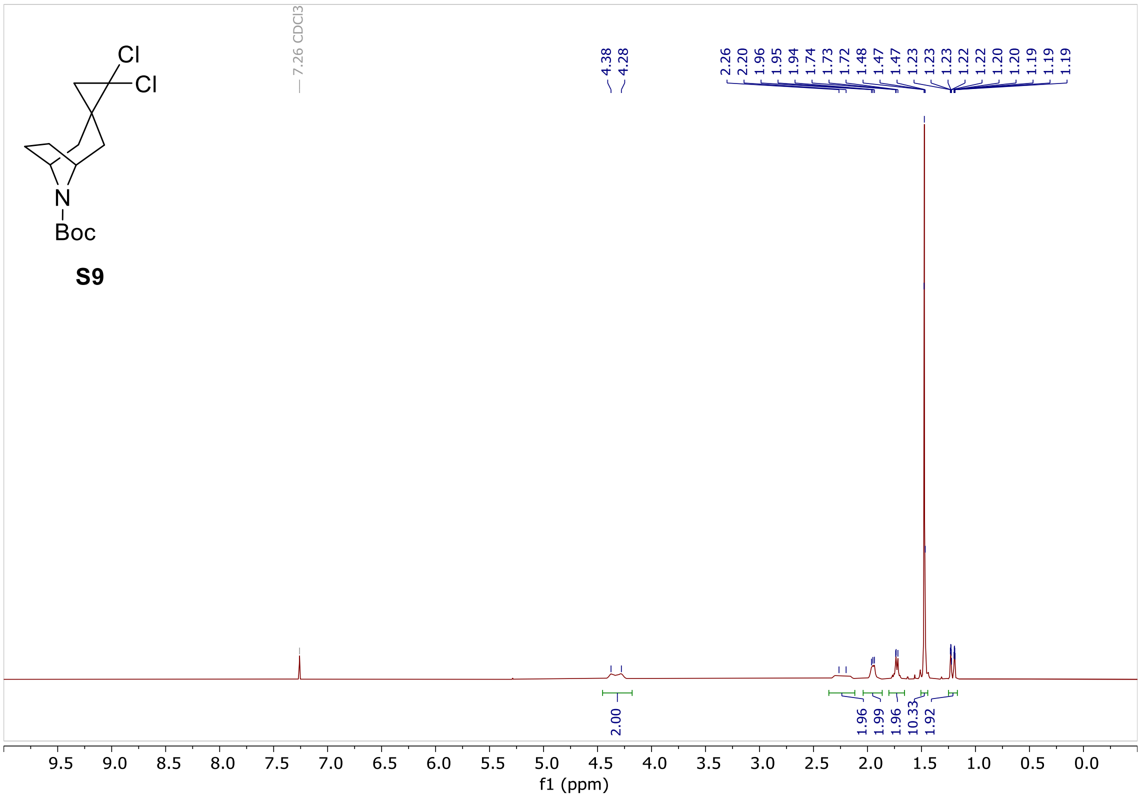

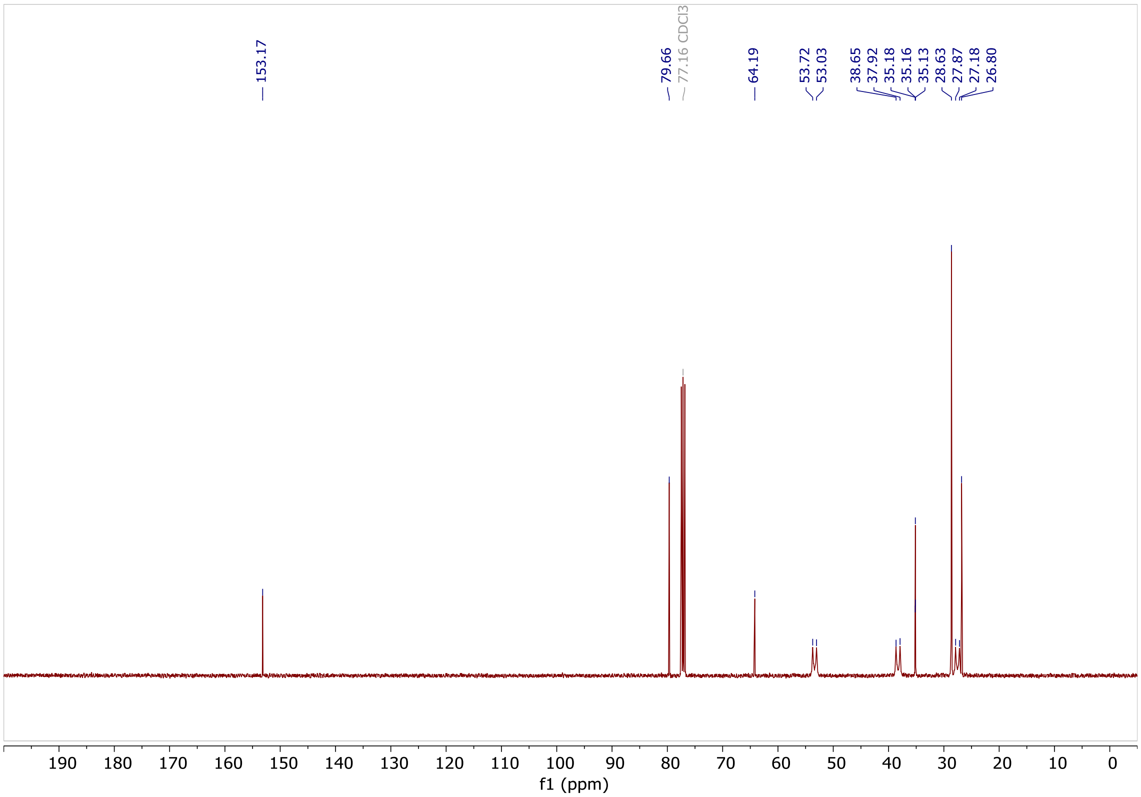

400 MHz ^{1}H NMR spectrum; 100.6 MHz ^{13}C NMR spectrum; 128.4 MHz ^{11}B NMR spectrum; $CDCl_3$ of **S10**

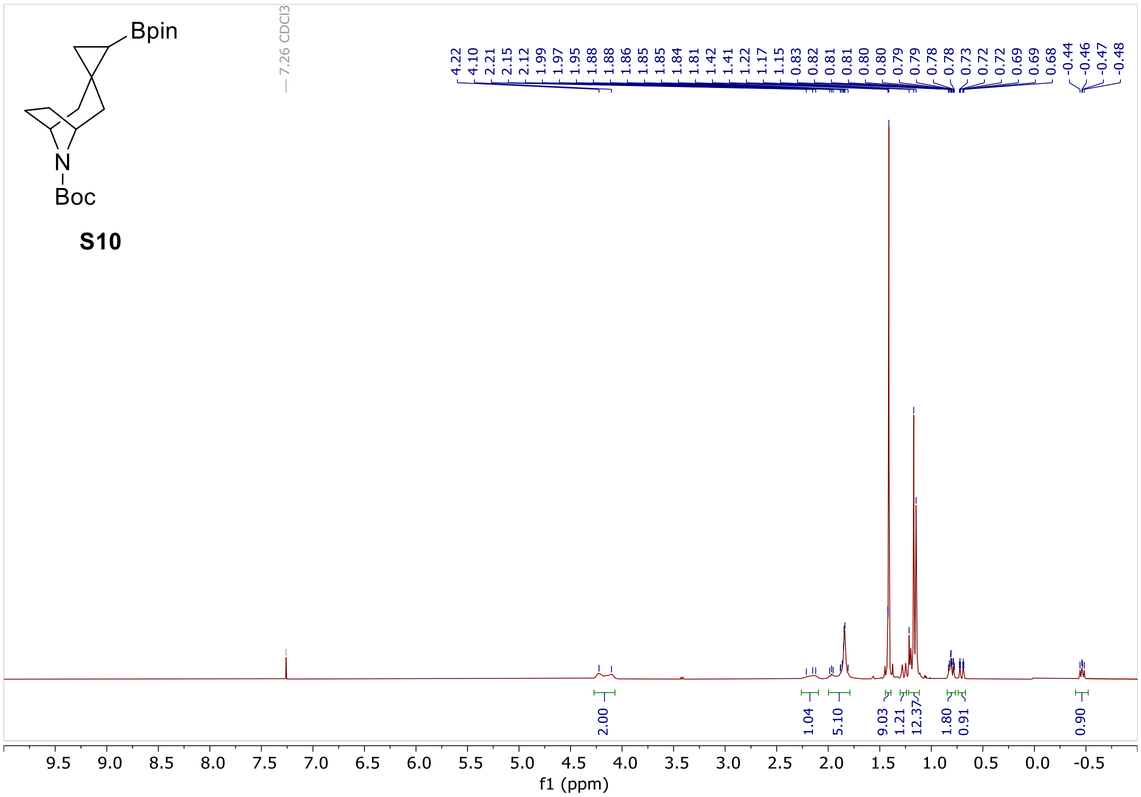

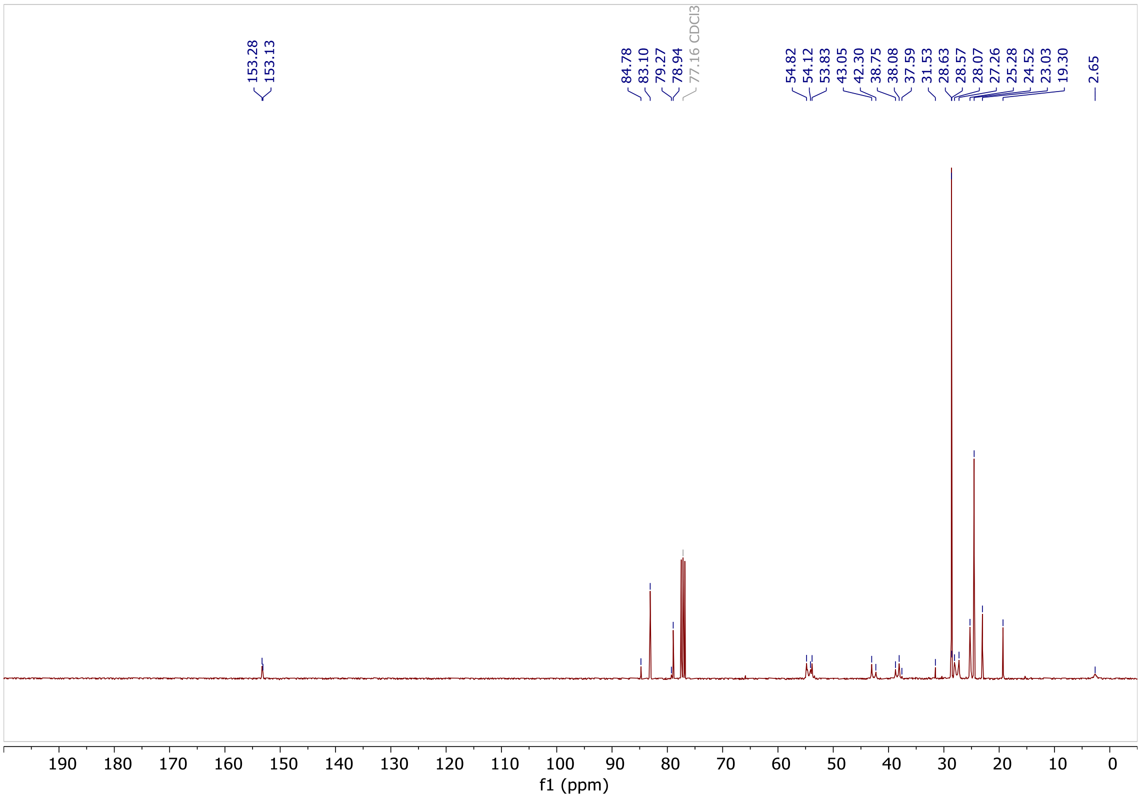

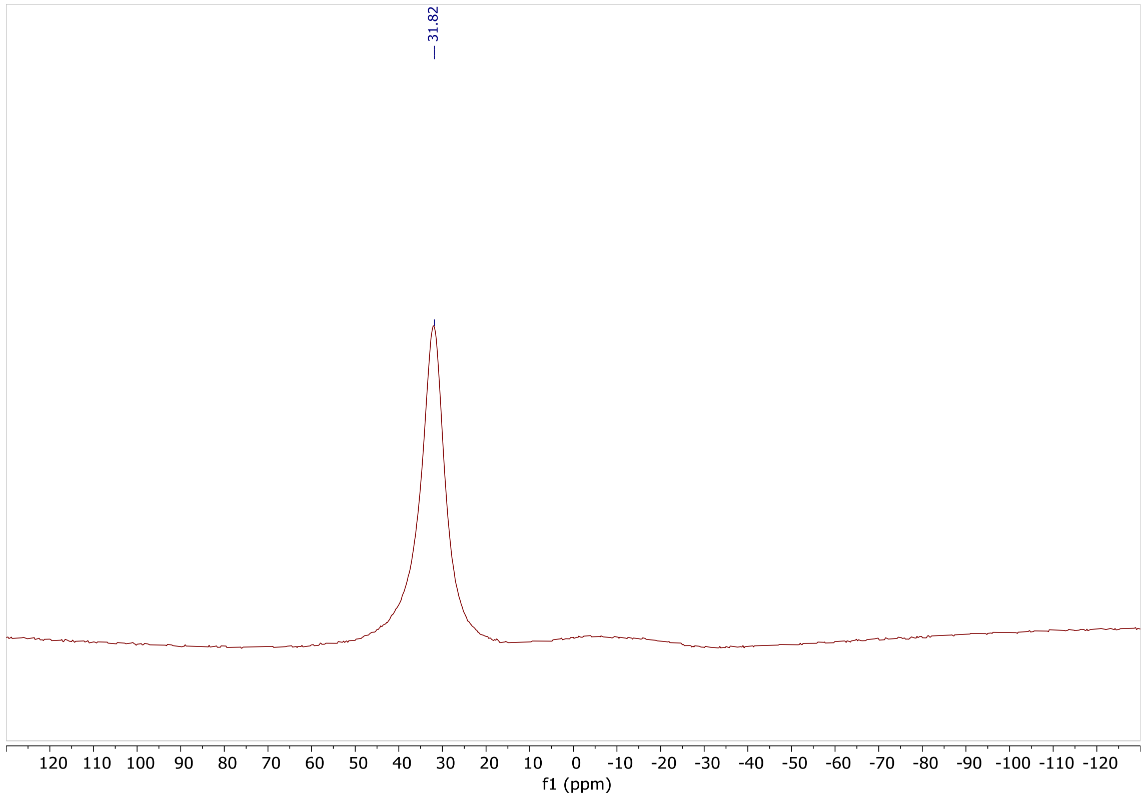
31.82
120 110 100 90 80 70 60 50 40 30 20 10 0 -10 -20 -30 -40 -50 -60 -70 -80 -90 -100 -110 -120
f1 (ppm)

400 MHz ^{1}H NMR spectrum; 100.6 MHz ^{13}C NMR spectrum; 128.4 MHz ^{11}B NMR spectrum; DMSO-d_6 of **1f**

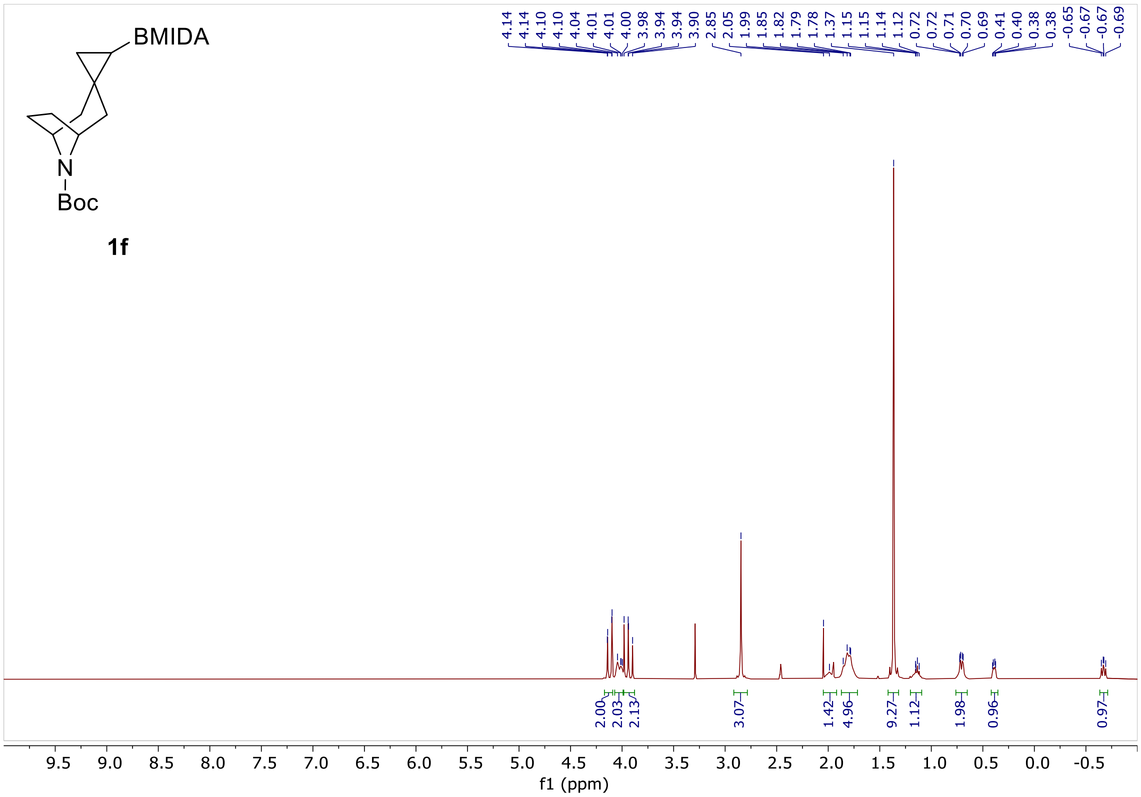

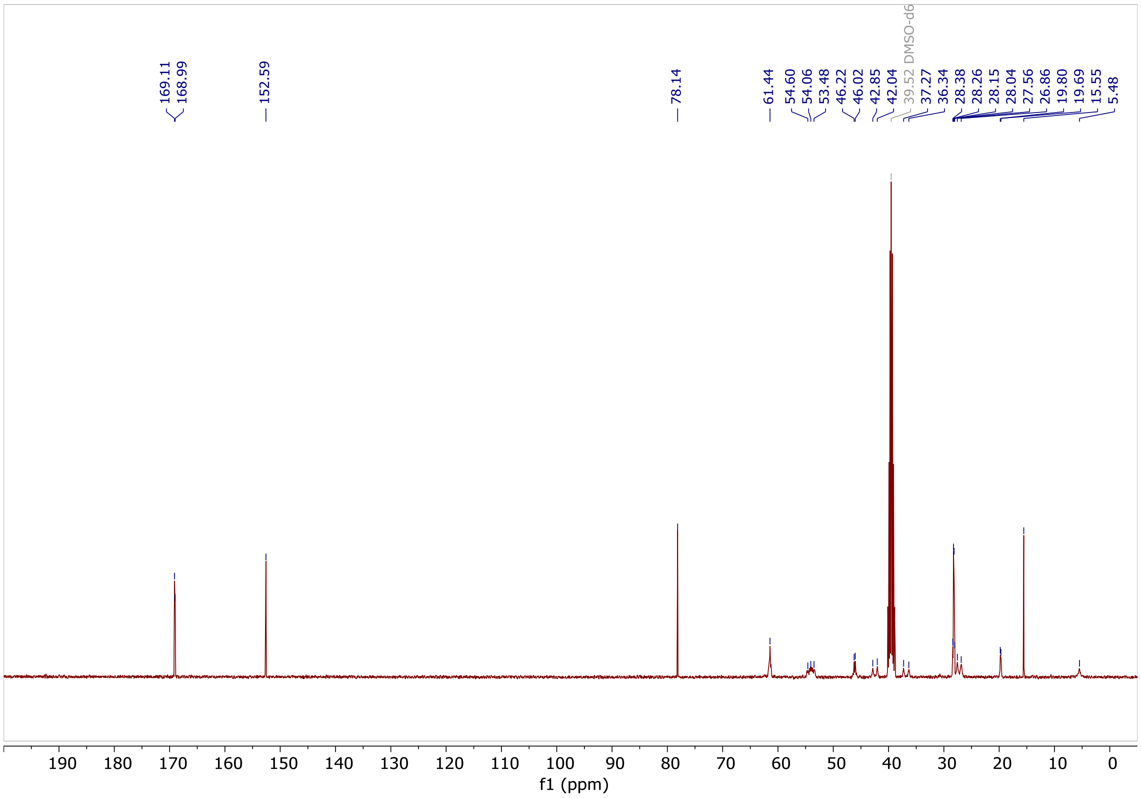

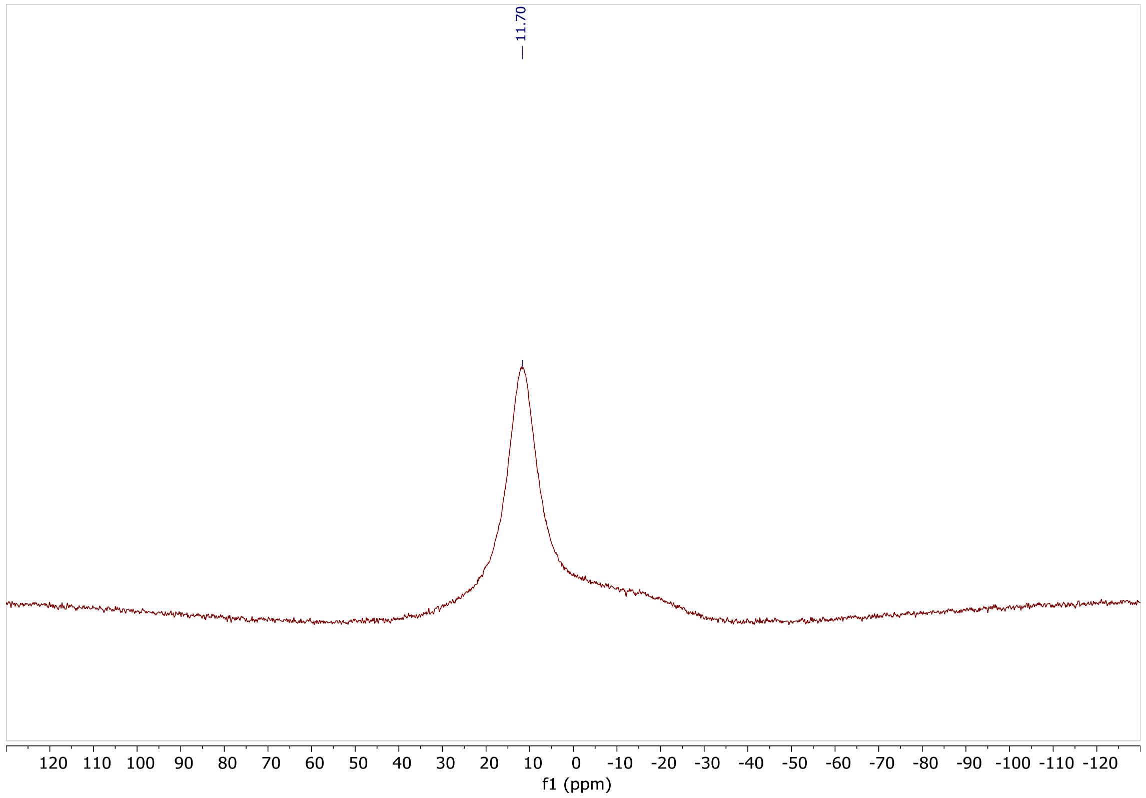
11.70
120 110 100 90 80 70 60 50 40 30 20 10 0 -10 -20 -30 -40 -50 -60 -70 -80 -90 -100 -110 -120
f1 (ppm)

400 MHz ^{1}H NMR spectrum; 100.6 MHz ^{13}C NMR spectrum; DMSO-d_6 of **S11**

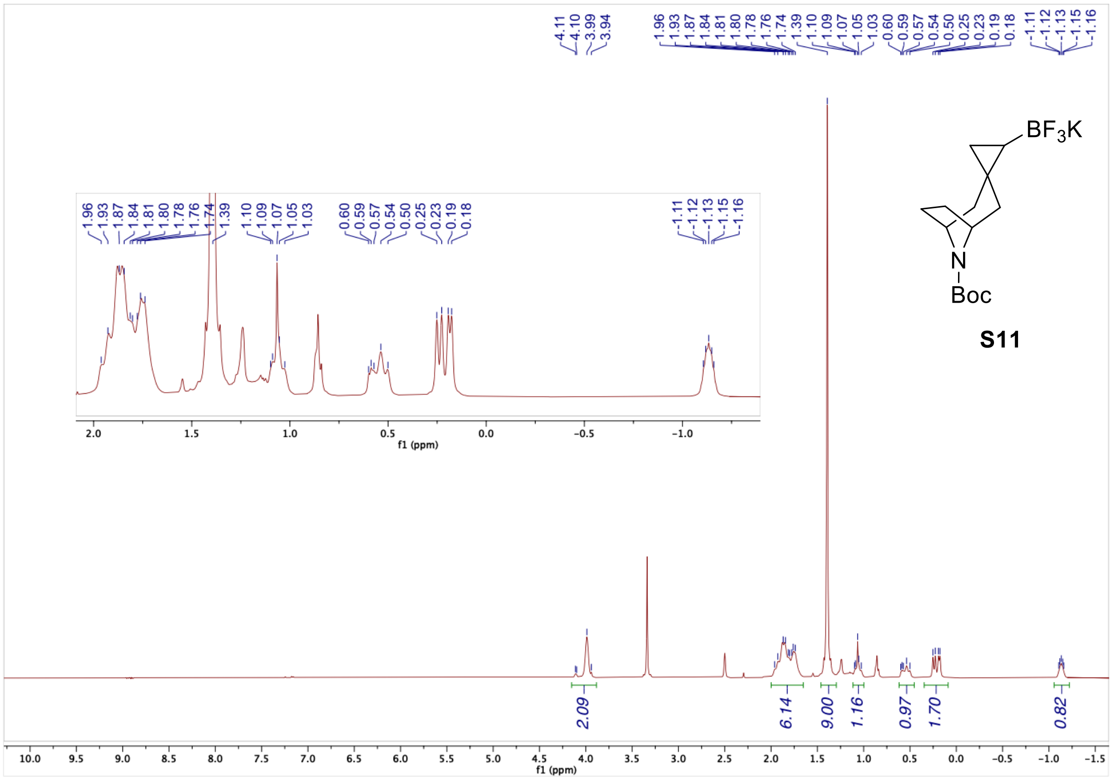

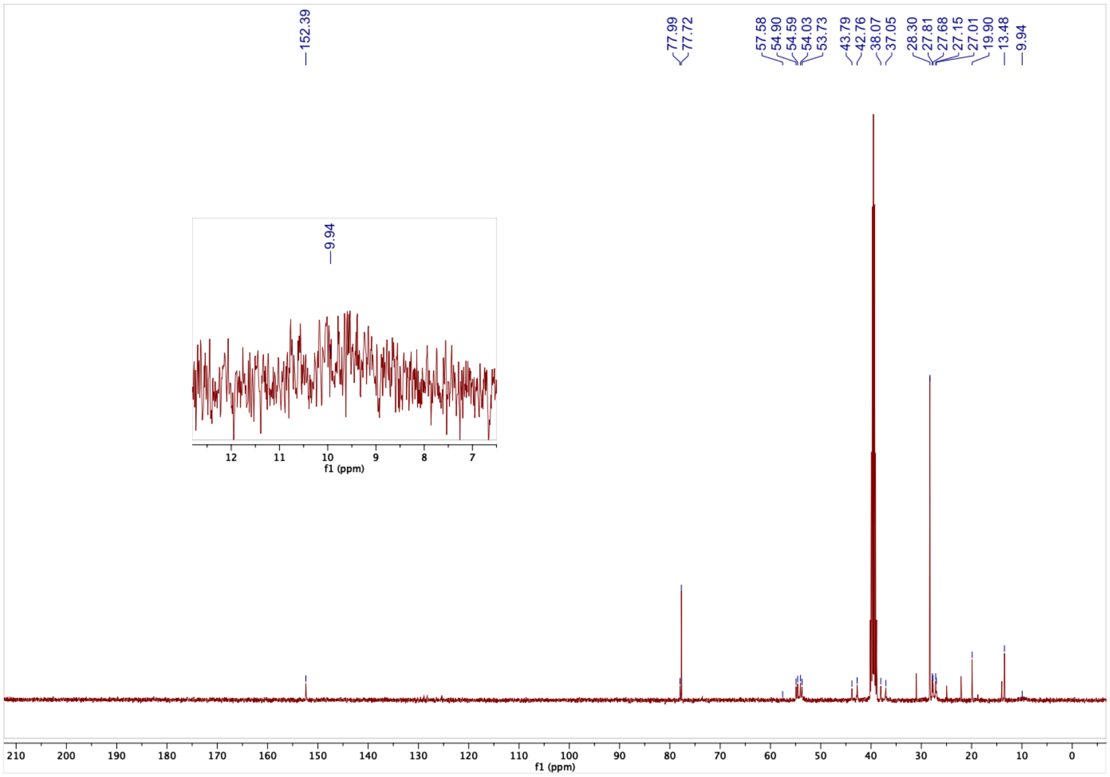

400 MHz ^{1}H NMR spectrum; 100.6 MHz ^{13}C NMR spectrum; $CDCl_3$ of **9**

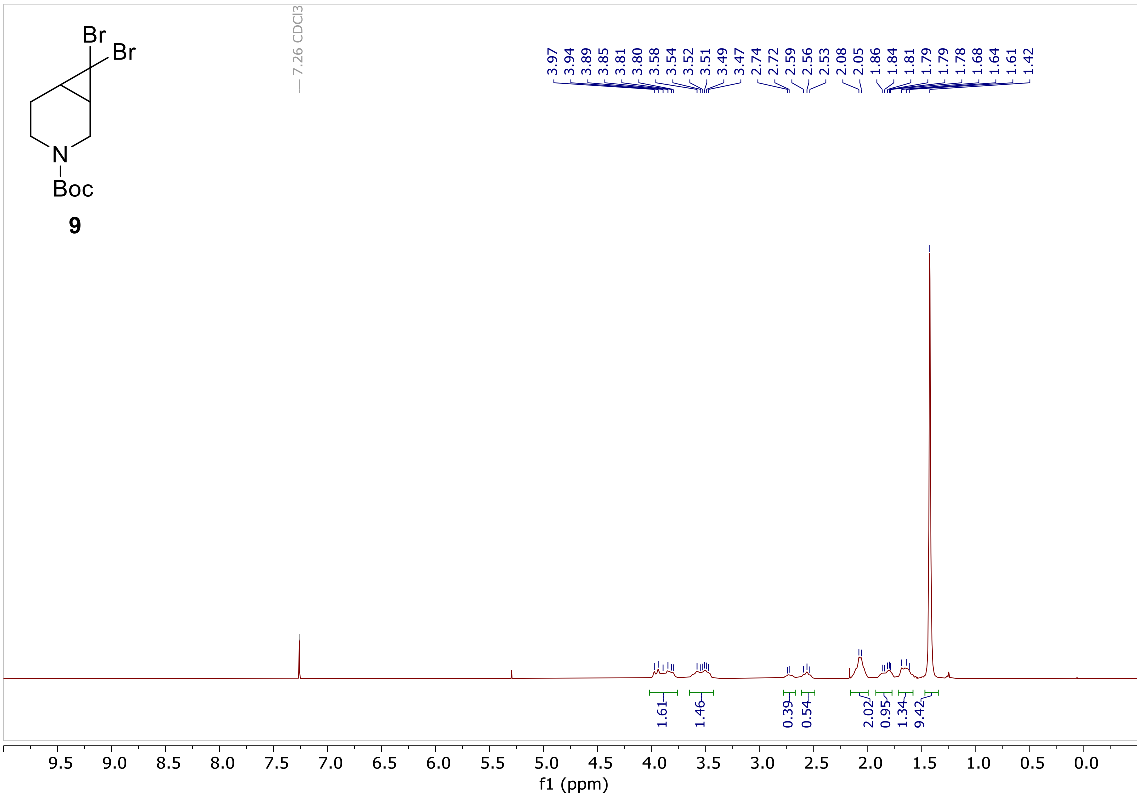

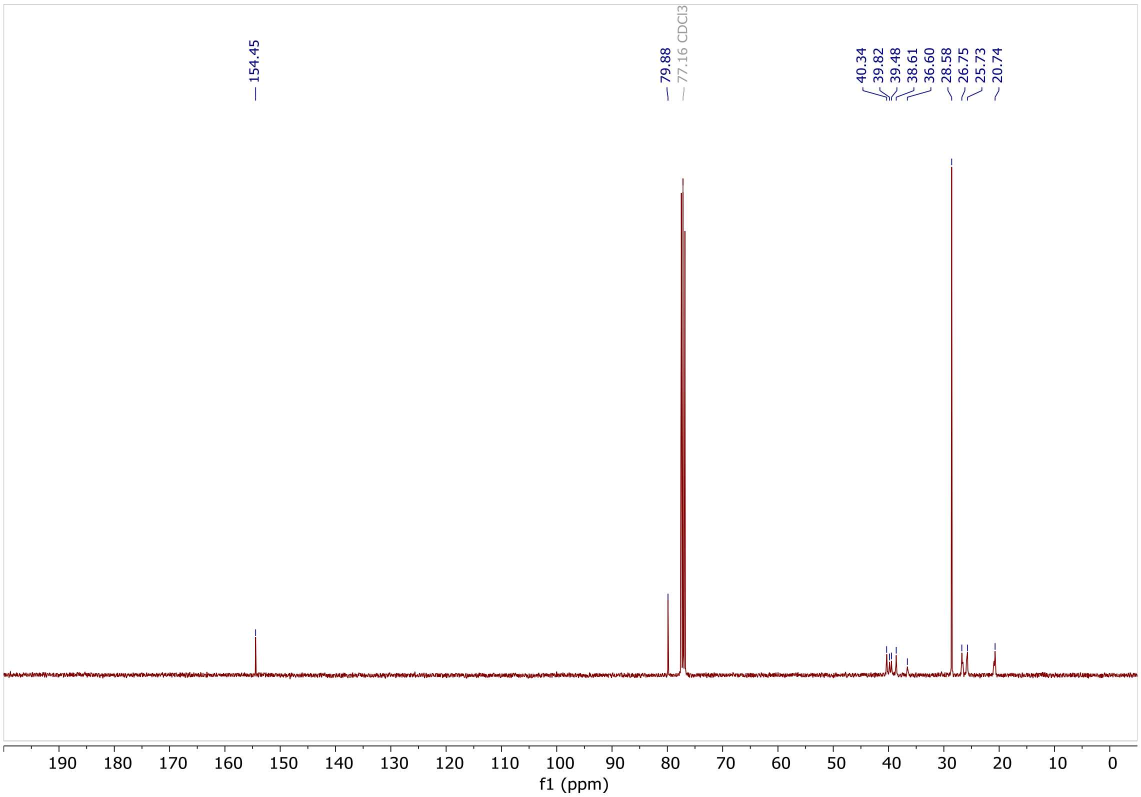

400 MHz ^{1}H NMR spectrum; 100.6 MHz ^{13}C NMR spectrum; $CDCl_3$ of **10**

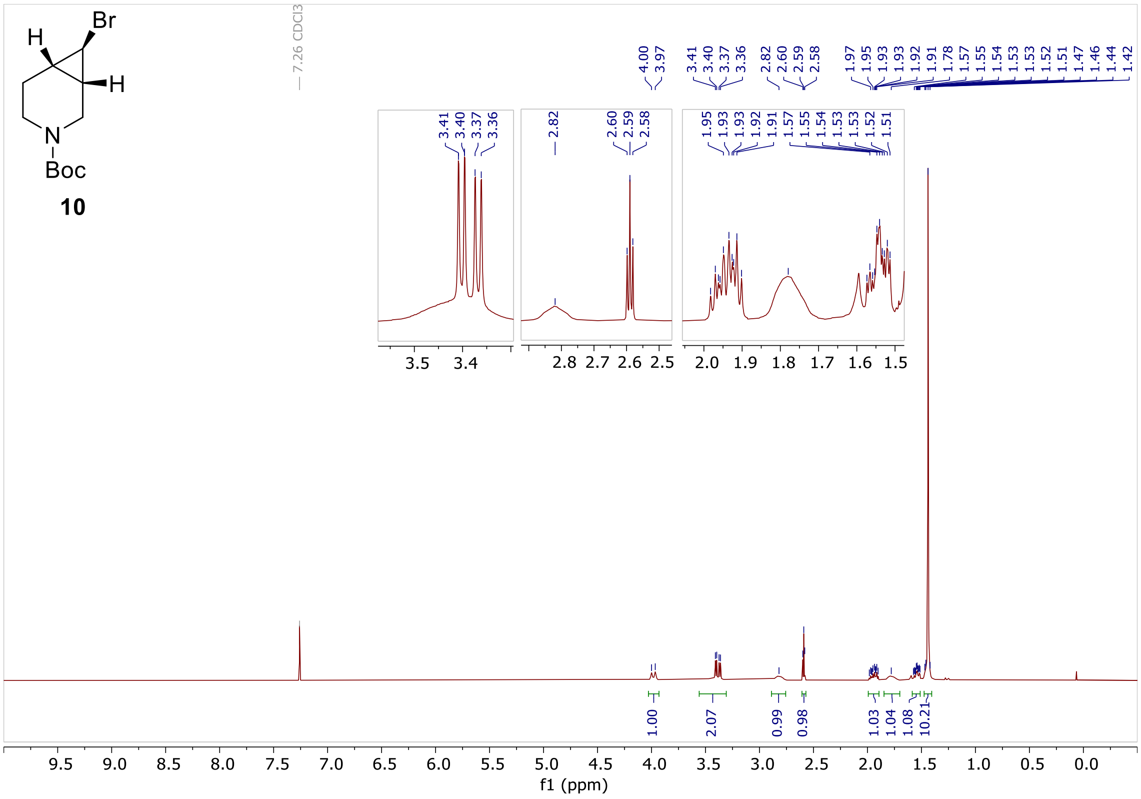

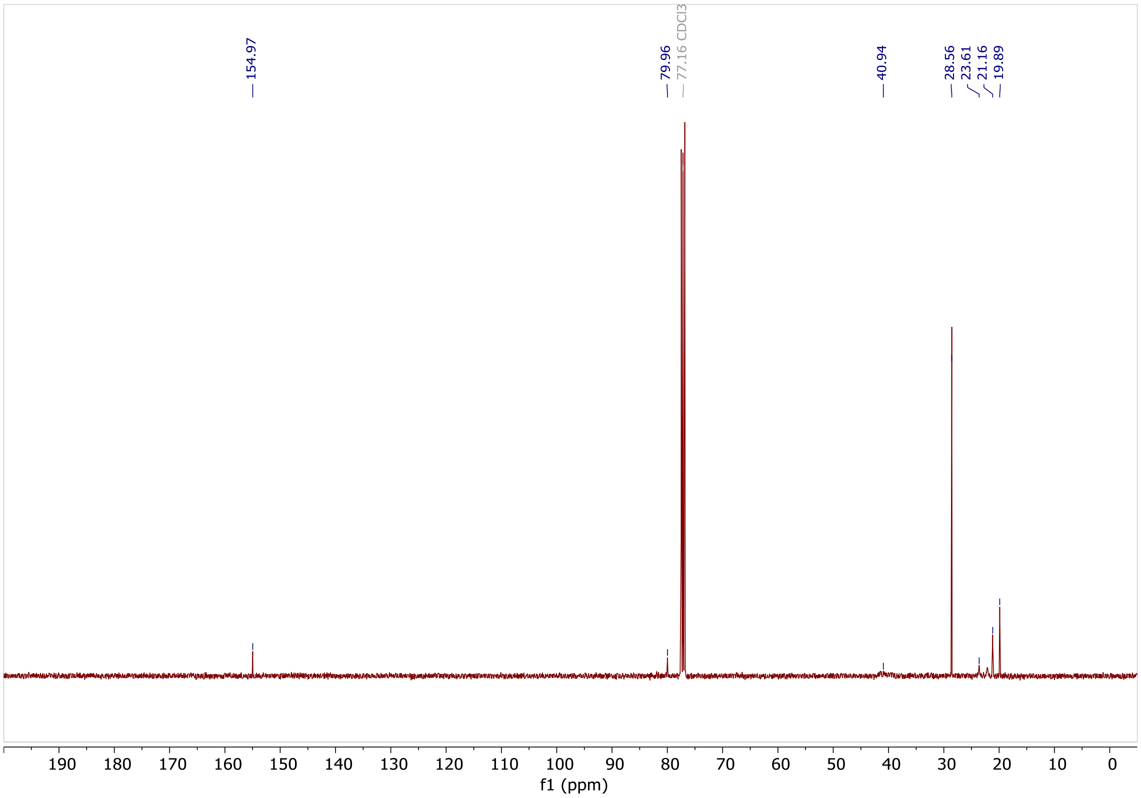

400 MHz ^{1}H NMR spectrum; 100.6 MHz ^{13}C NMR spectrum; $CDCl_3$ of **11**

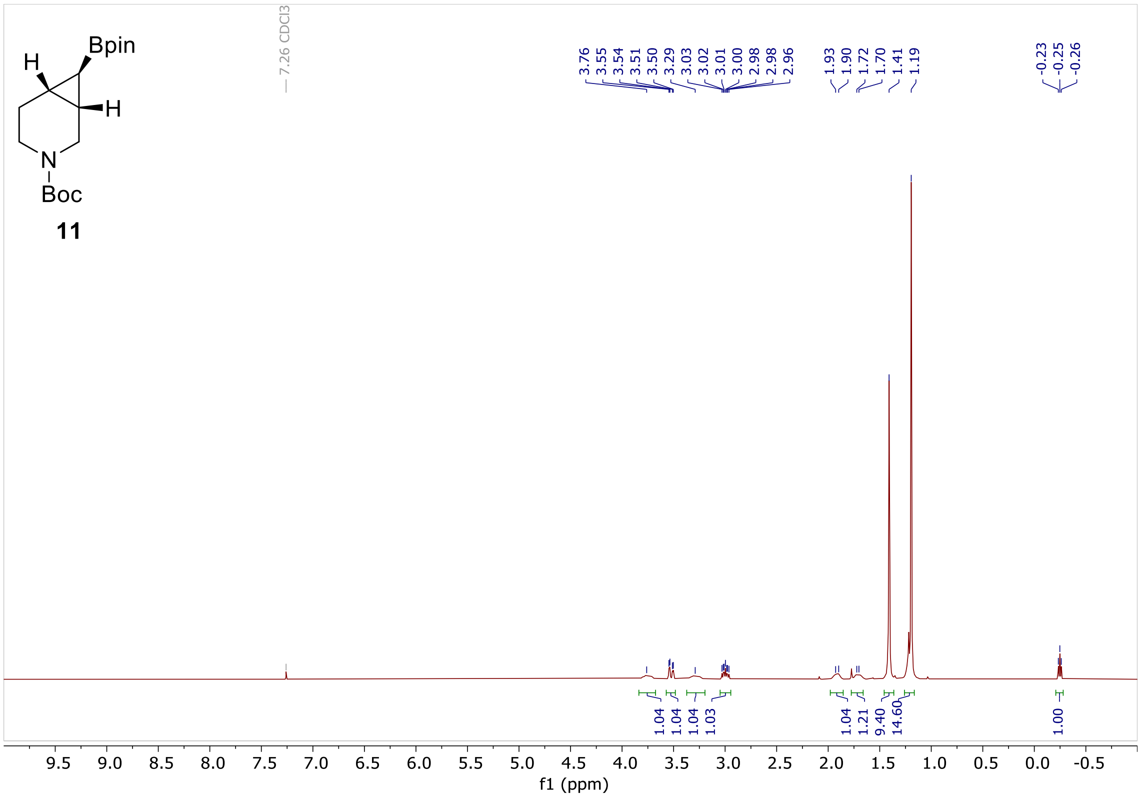

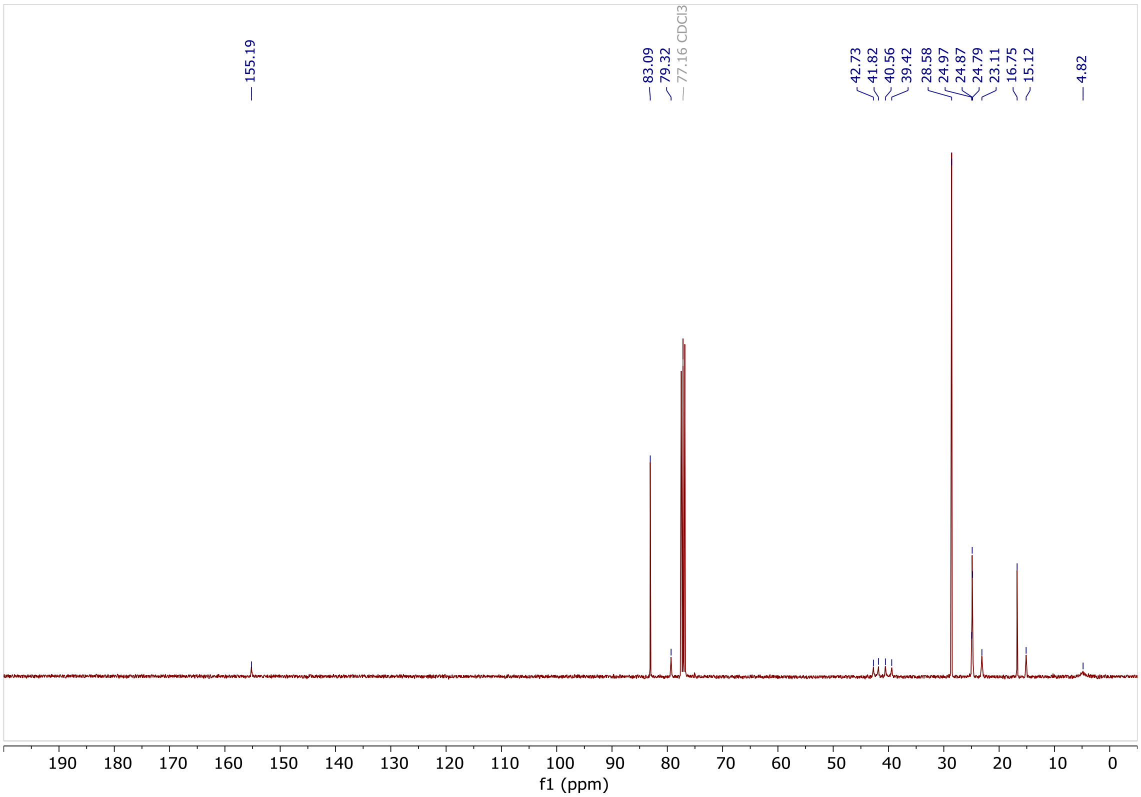

400 MHz ^{1}H NMR spectrum; 100.6 MHz ^{13}C NMR spectrum; d_6-acetone of **1g**

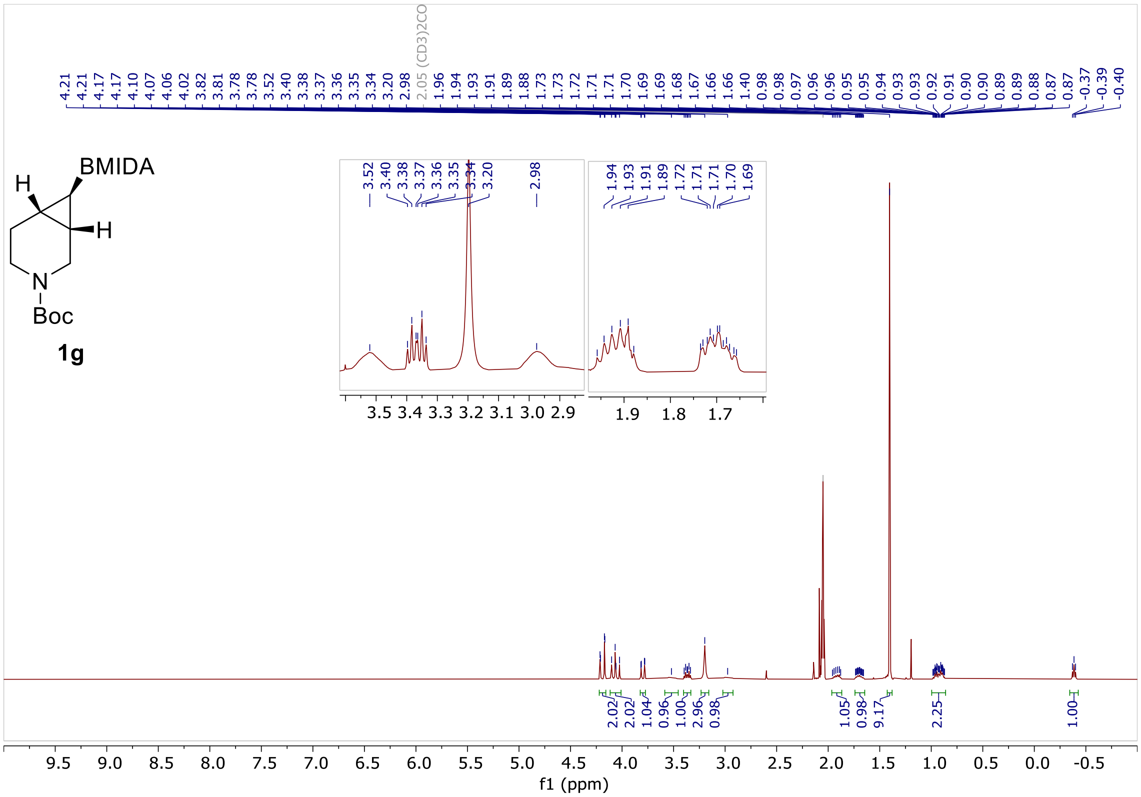

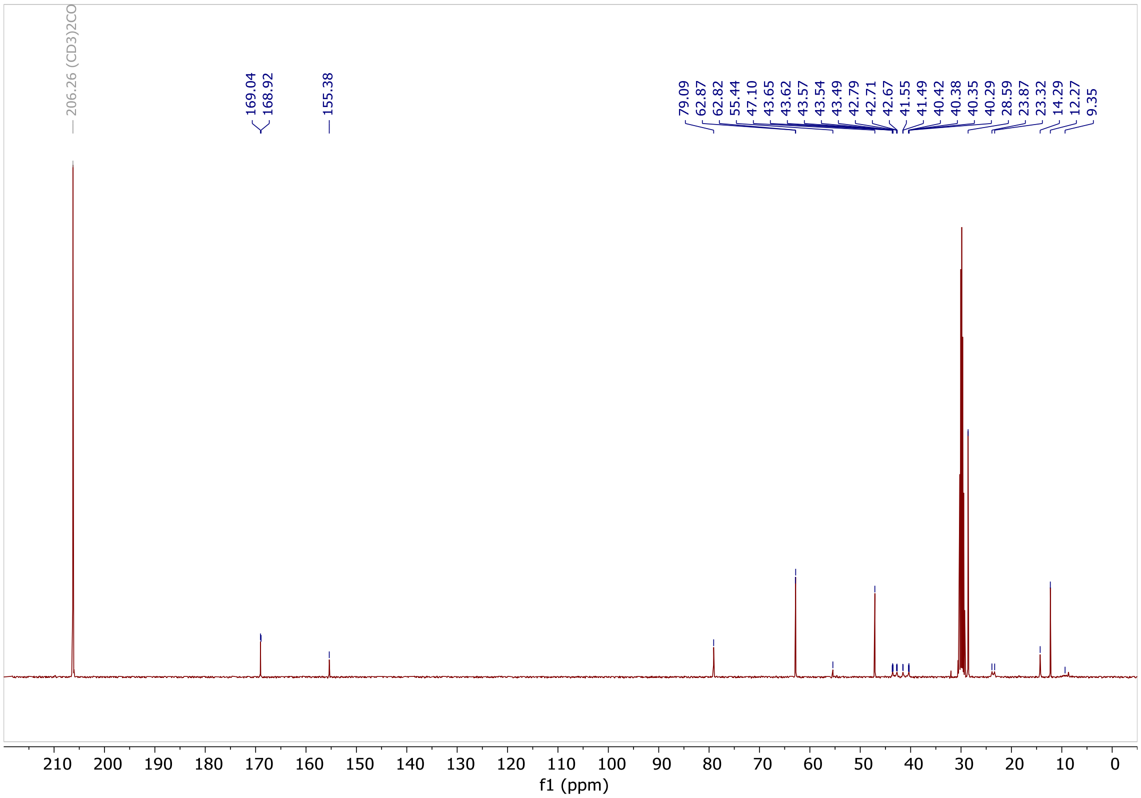

400 MHz ^{1}H NMR spectrum; 100.6 MHz ^{13}C NMR spectrum; 128.4 MHz ^{11}B spectrum; DMSO-d_6 of **S12**

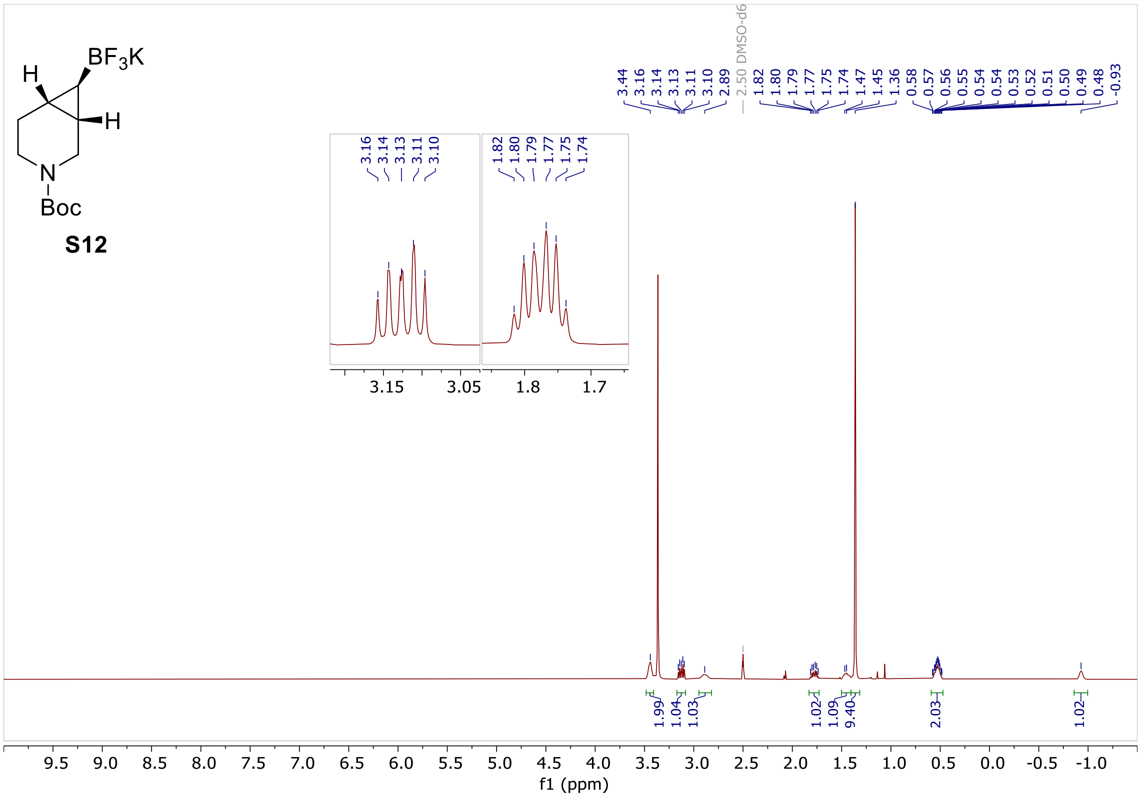

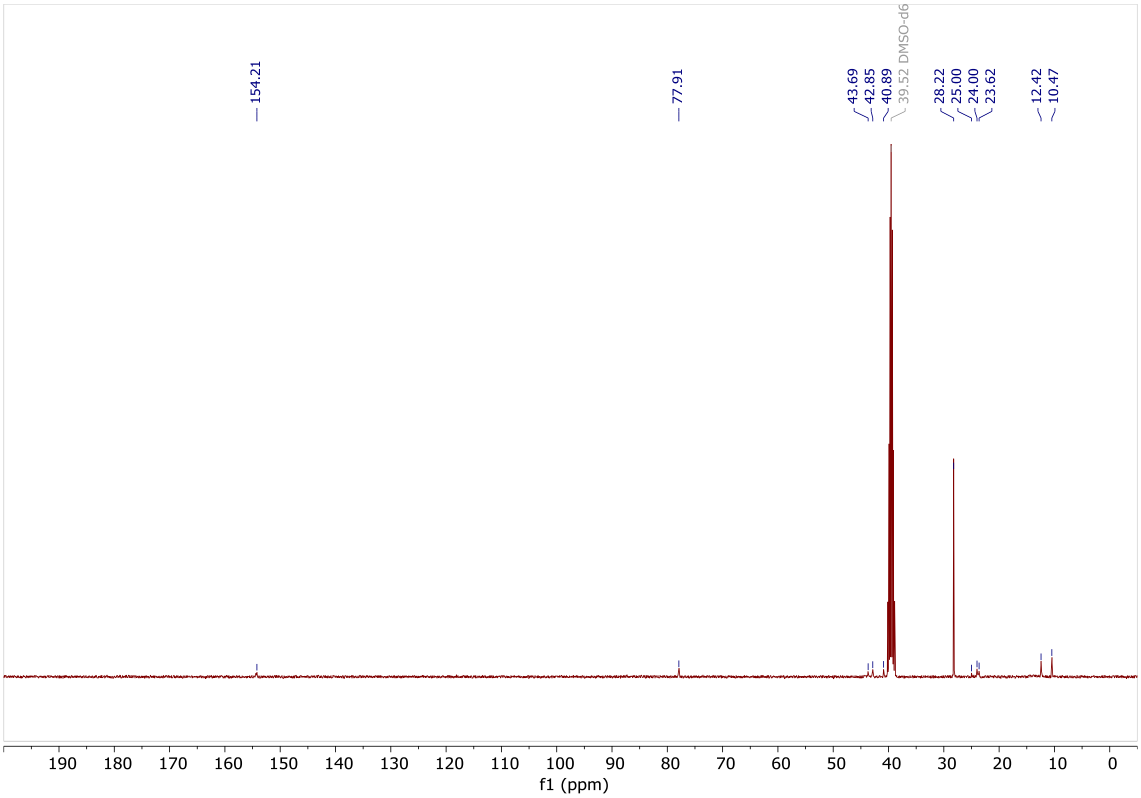

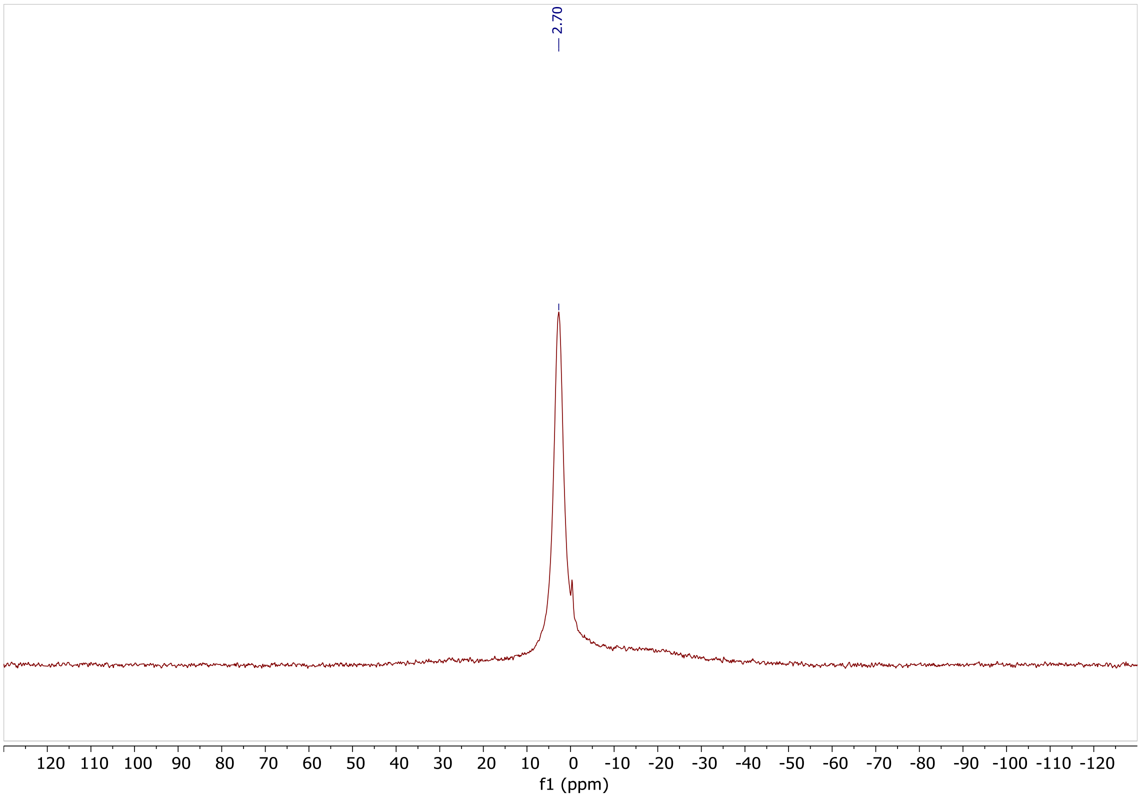
2.70
120 110 100 90 80 70 60 50 40 30 20 10 0 -10 -20 -30 -40 -50 -60 -70 -80 -90 -100 -110 -120
f1 (ppm)

400 MHz ^{1}H NMR spectrum; 100.6 MHz ^{13}C NMR spectrum; $CDCl_3$ of **S13**

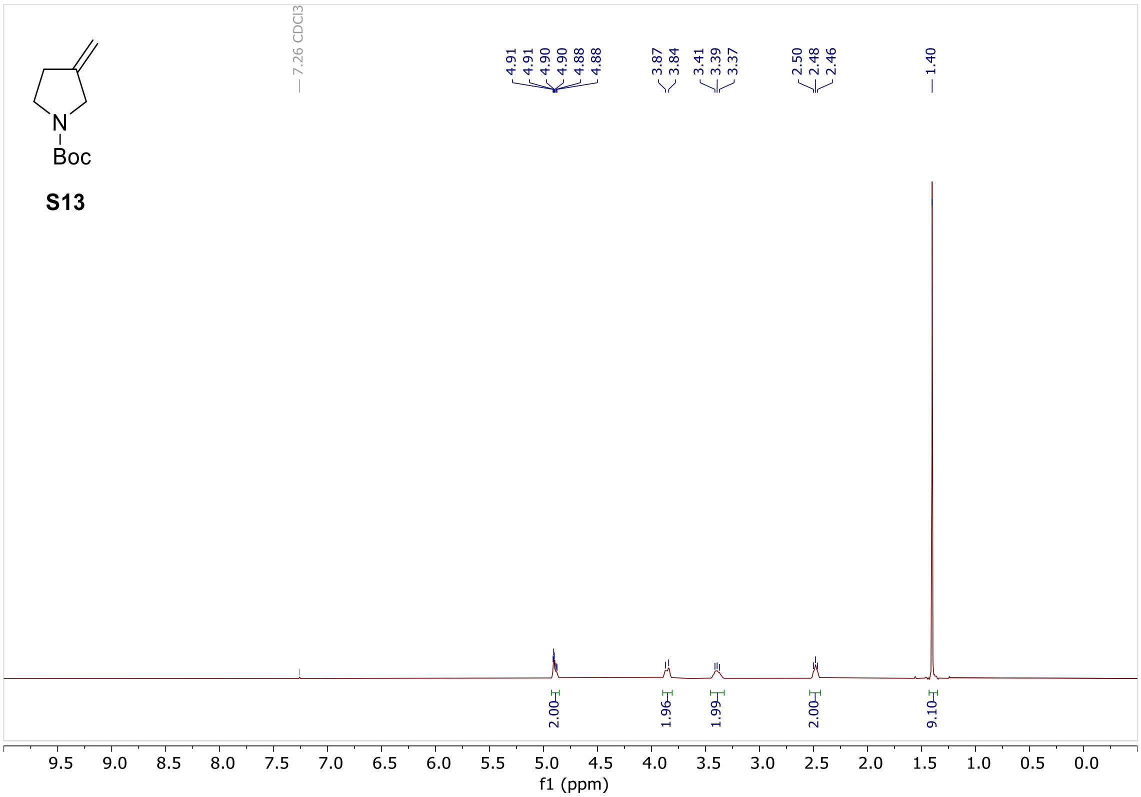

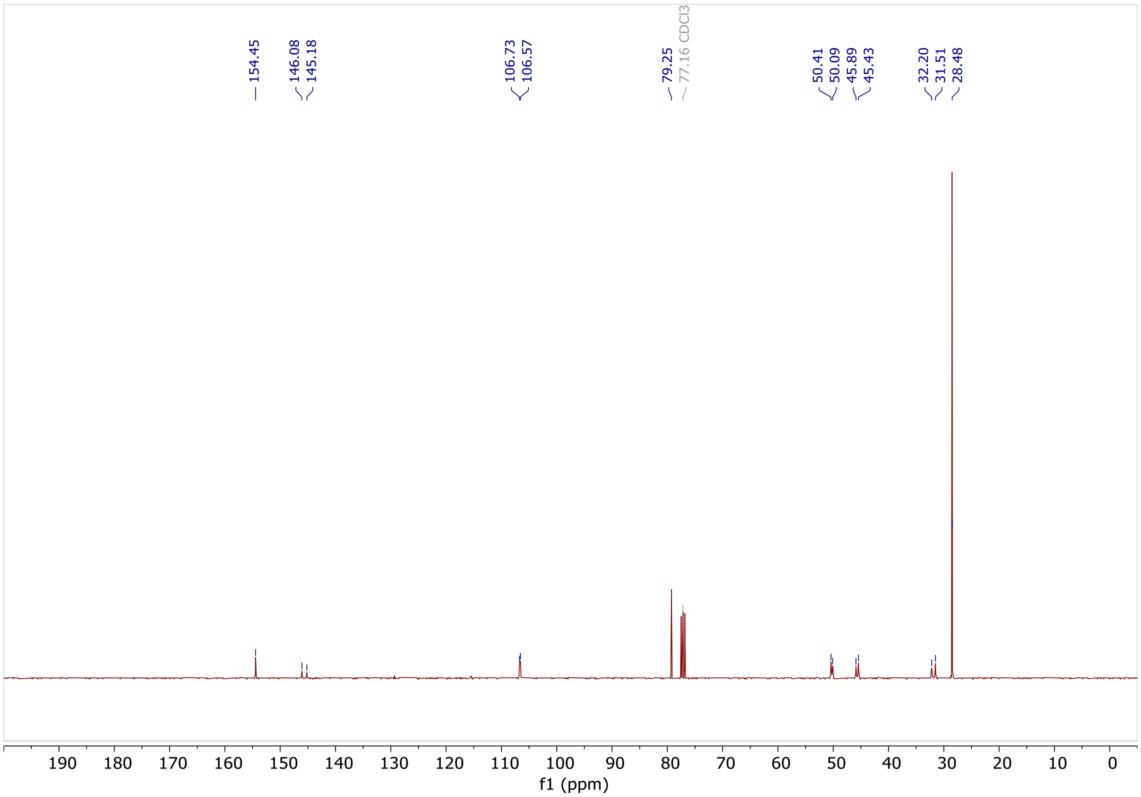

400 MHz ^1H NMR spectrum; 100.6 MHz ^{13}C NMR spectrum; $CDCl_3$ of **S14**

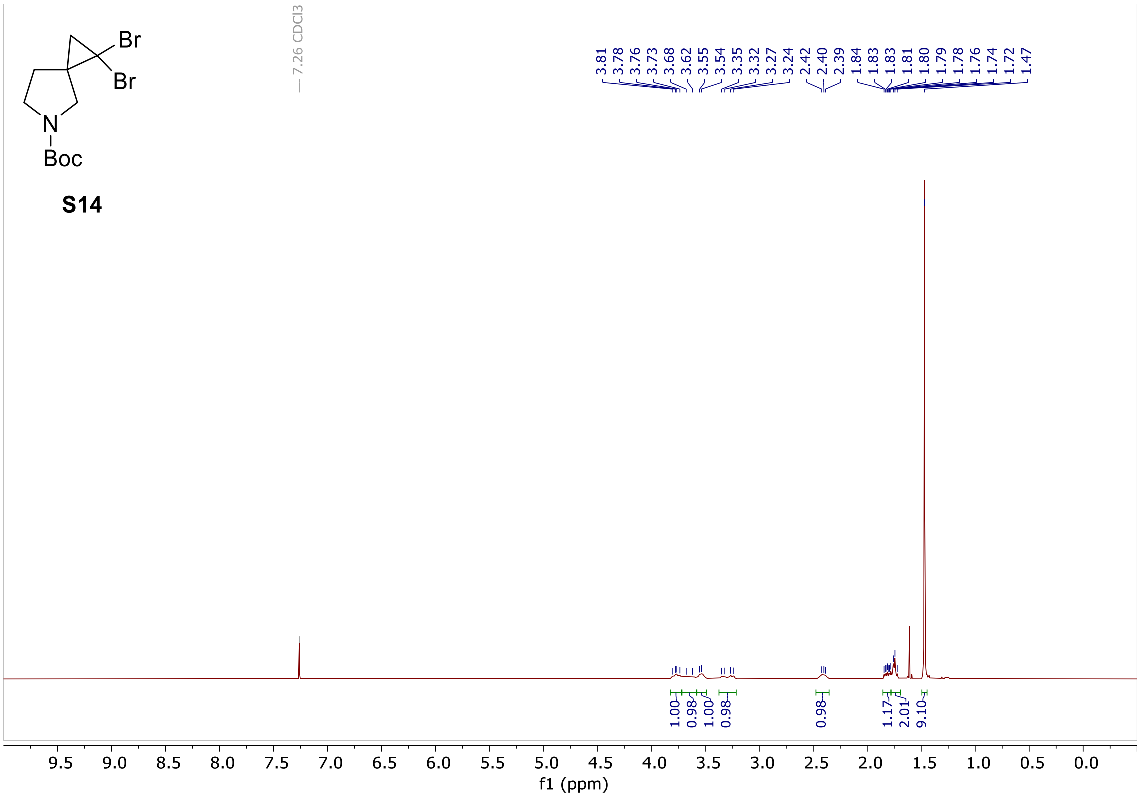

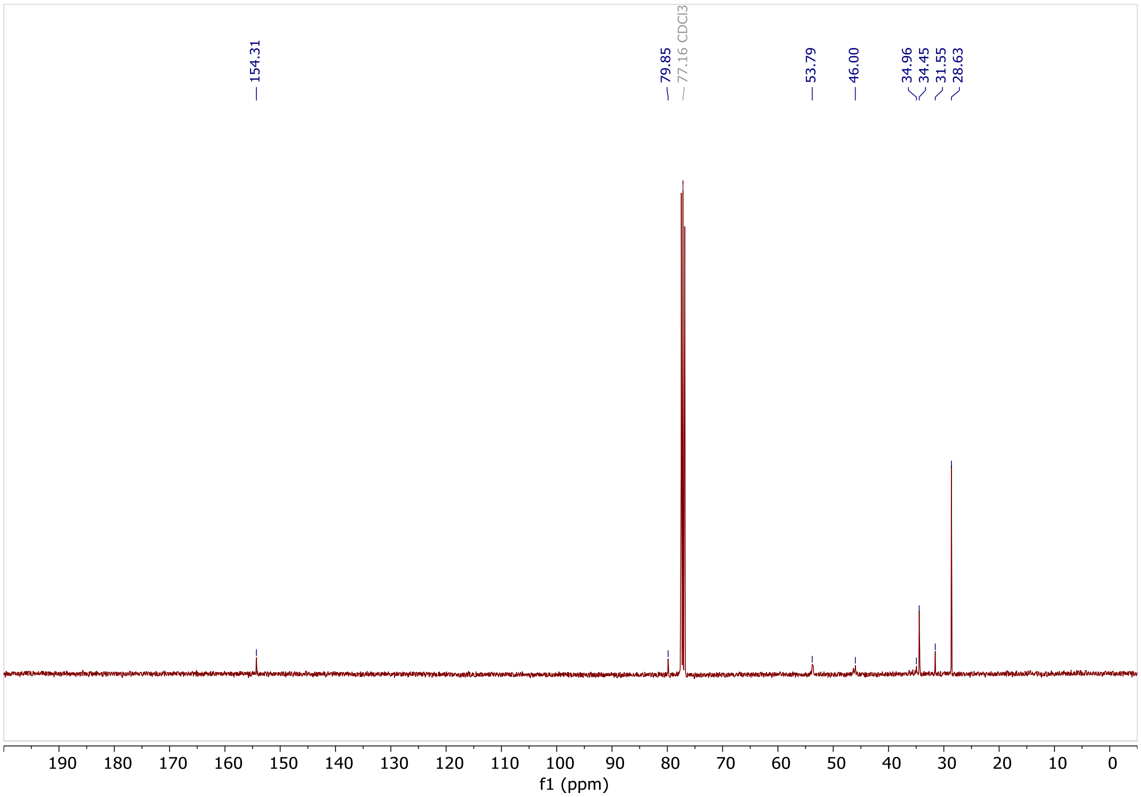

400 MHz ^1H NMR spectrum; 100.6 MHz ^{13}C NMR spectrum; $CDCl_3$ of **S15**

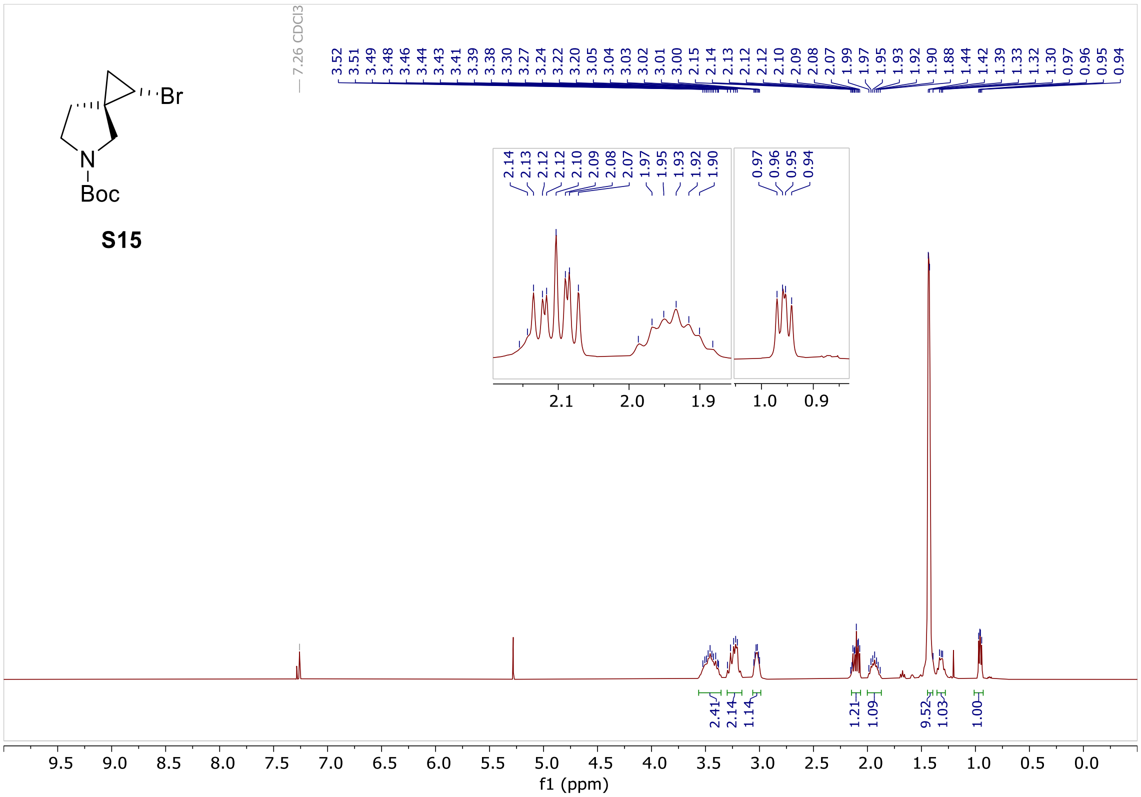

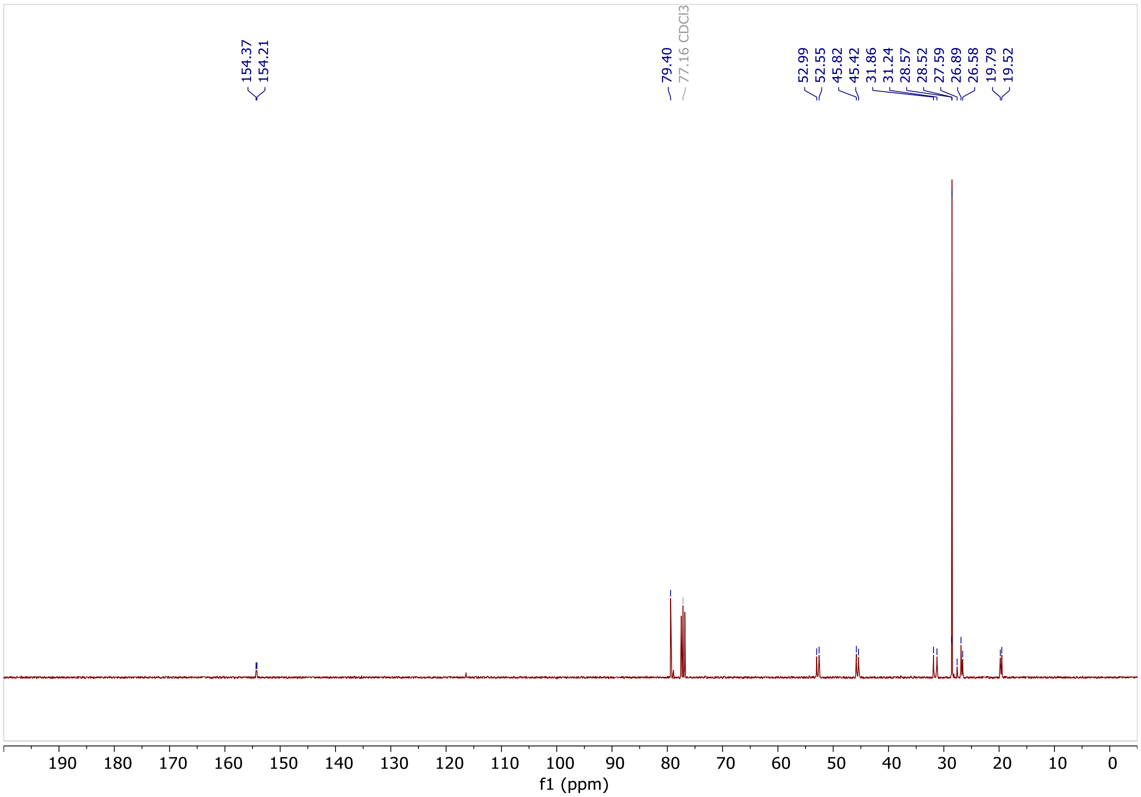

400 MHz ^{1}H NMR spectrum; 100.6 MHz ^{13}C NMR spectrum; $CDCl_3$ of **S16**

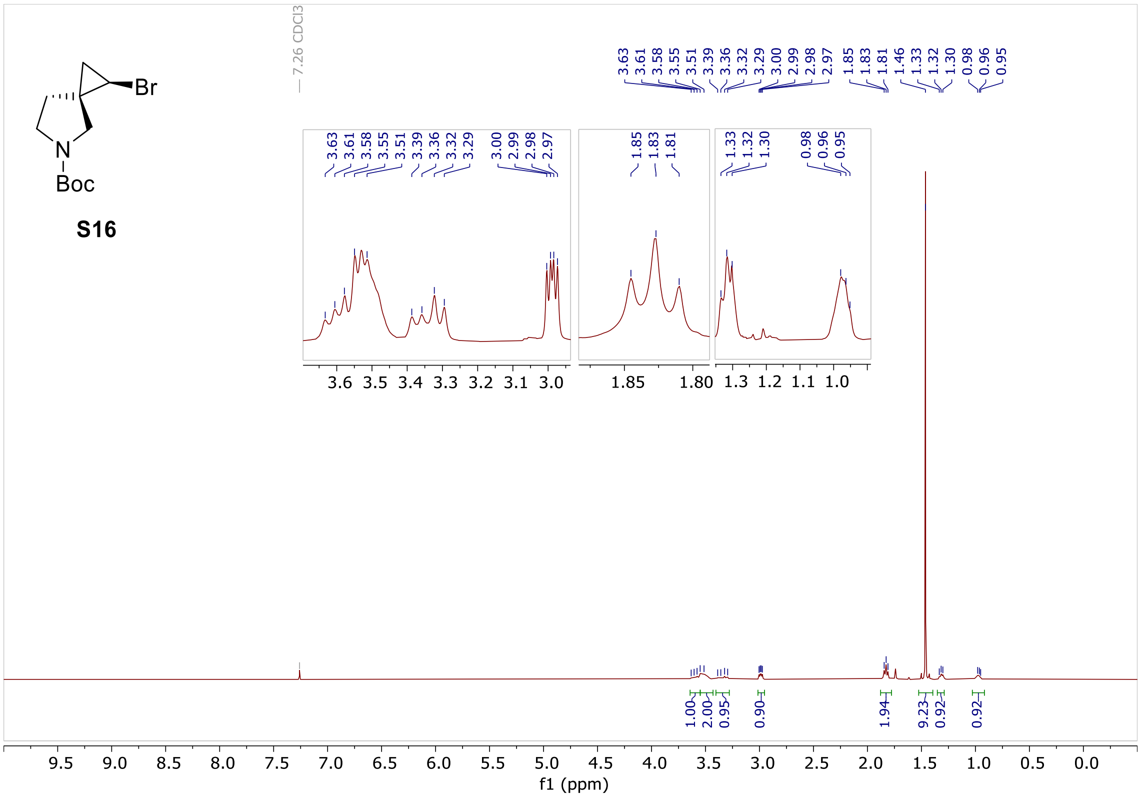

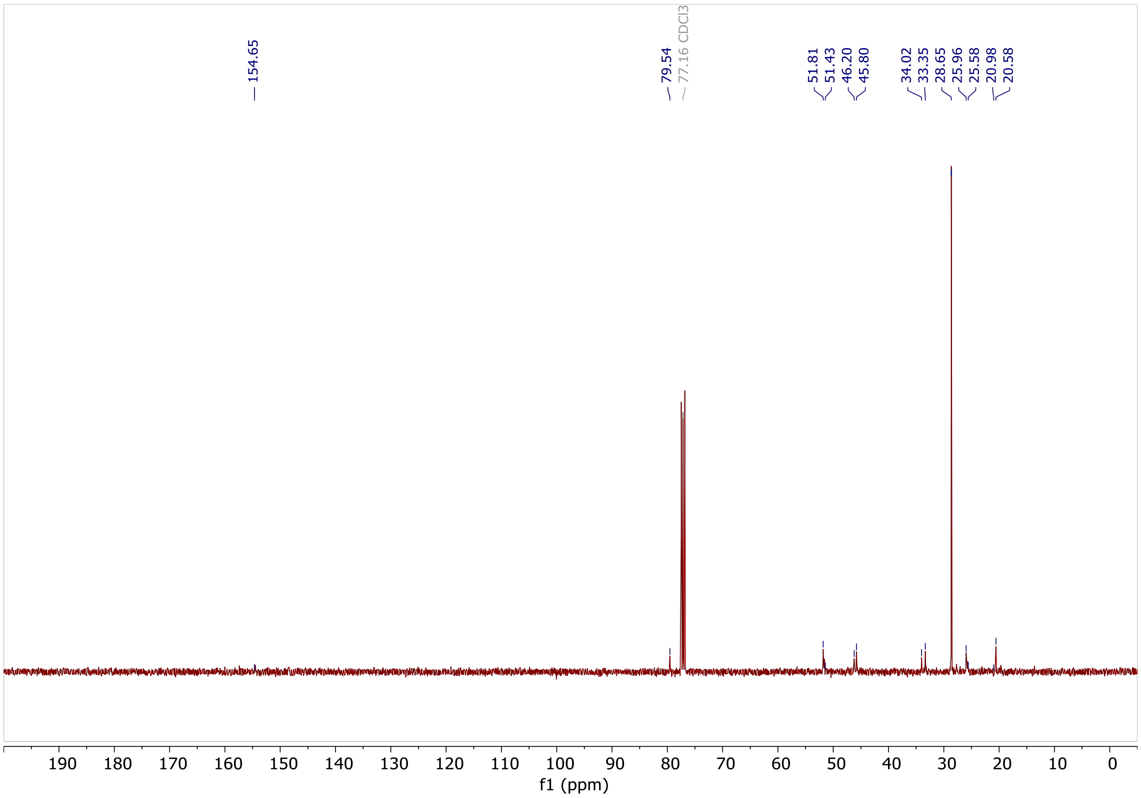

400 MHz ^{1}H NMR spectrum; 100.6 MHz ^{13}C NMR spectrum; 128.4 MHz ^{11}B NMR spectrum; $CDCl_3$ of **S17**

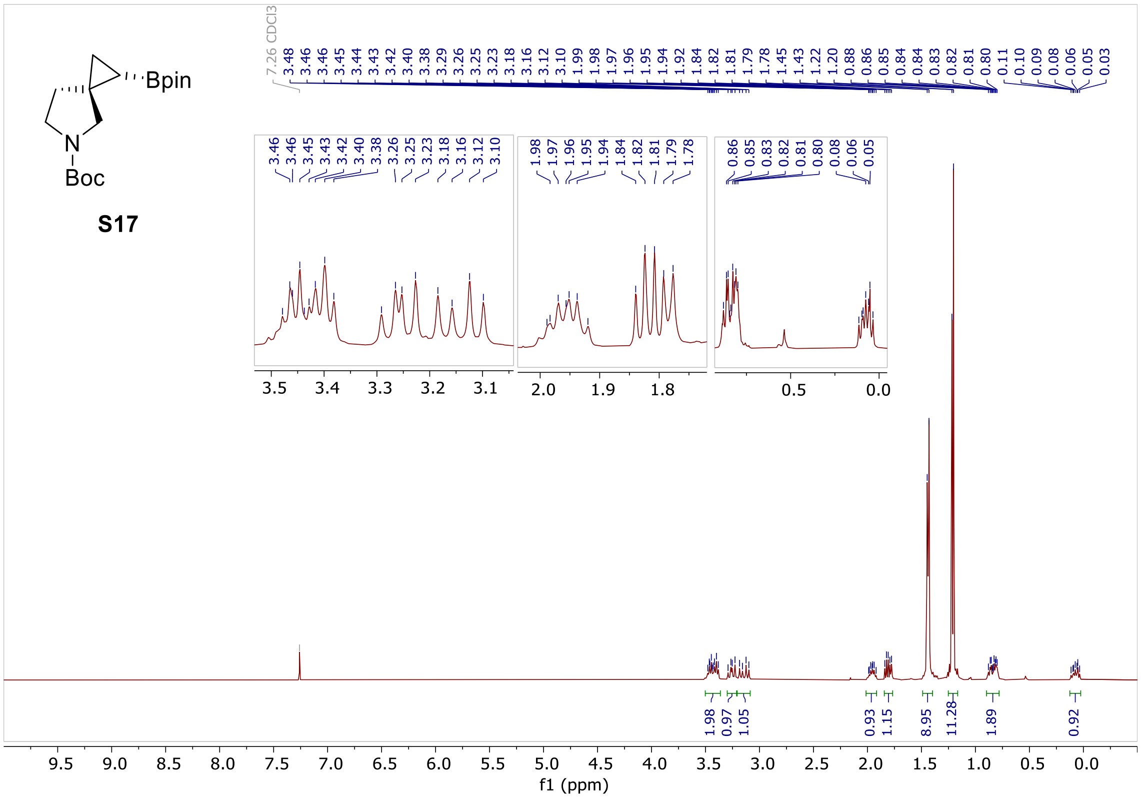

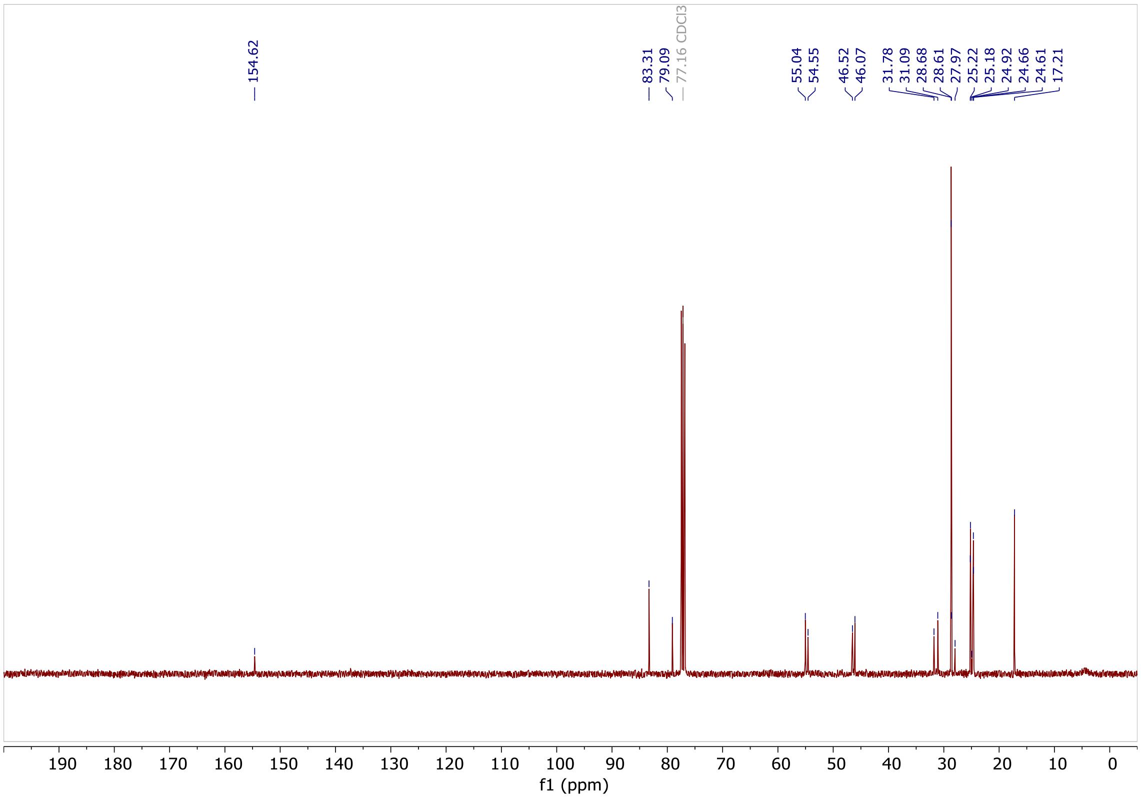

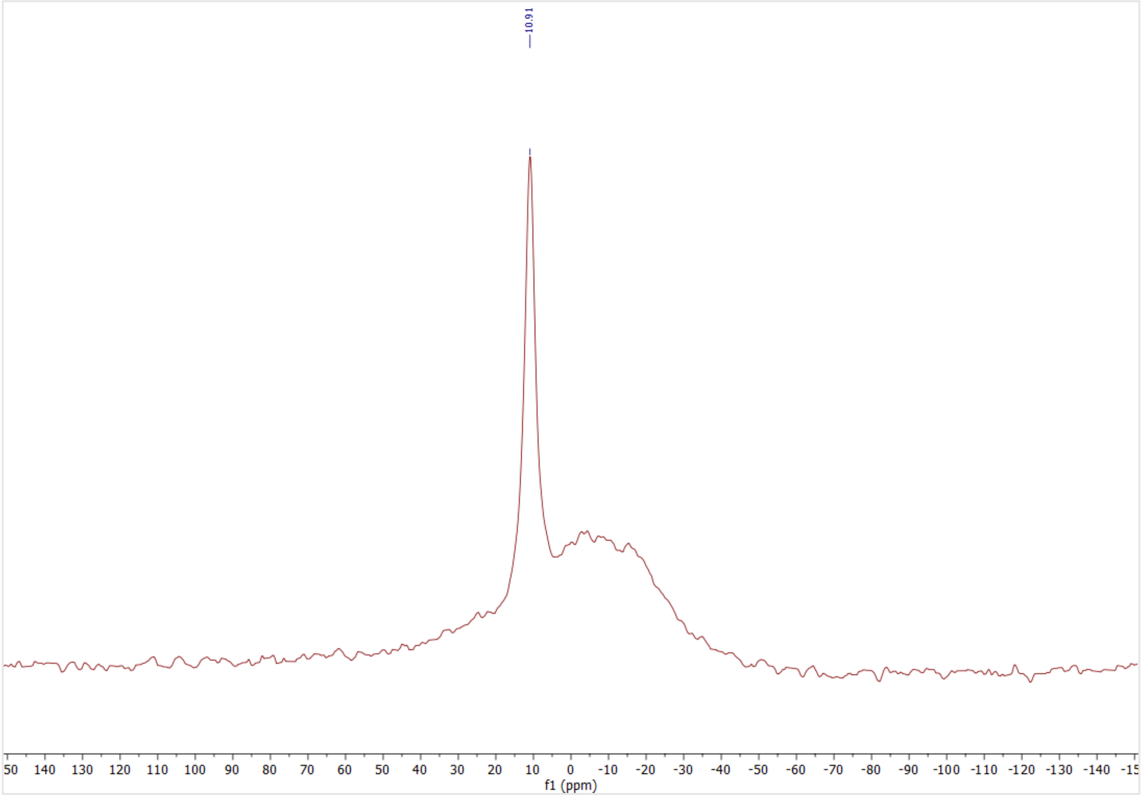

10.91
150 140 130 120 110 100 90 80 70 60 50 40 30 20 10 0 -10 -20 -30 -40 -50 -60 -70 -80 -90 -100 -110 -120 -130 -140 -150
f1 (ppm)

400 MHz ^{1}H NMR spectrum; 100.6 MHz ^{13}C NMR spectrum; 128.4 MHz ^{11}B NMR spectrum; $CDCl_3$ of **1d**

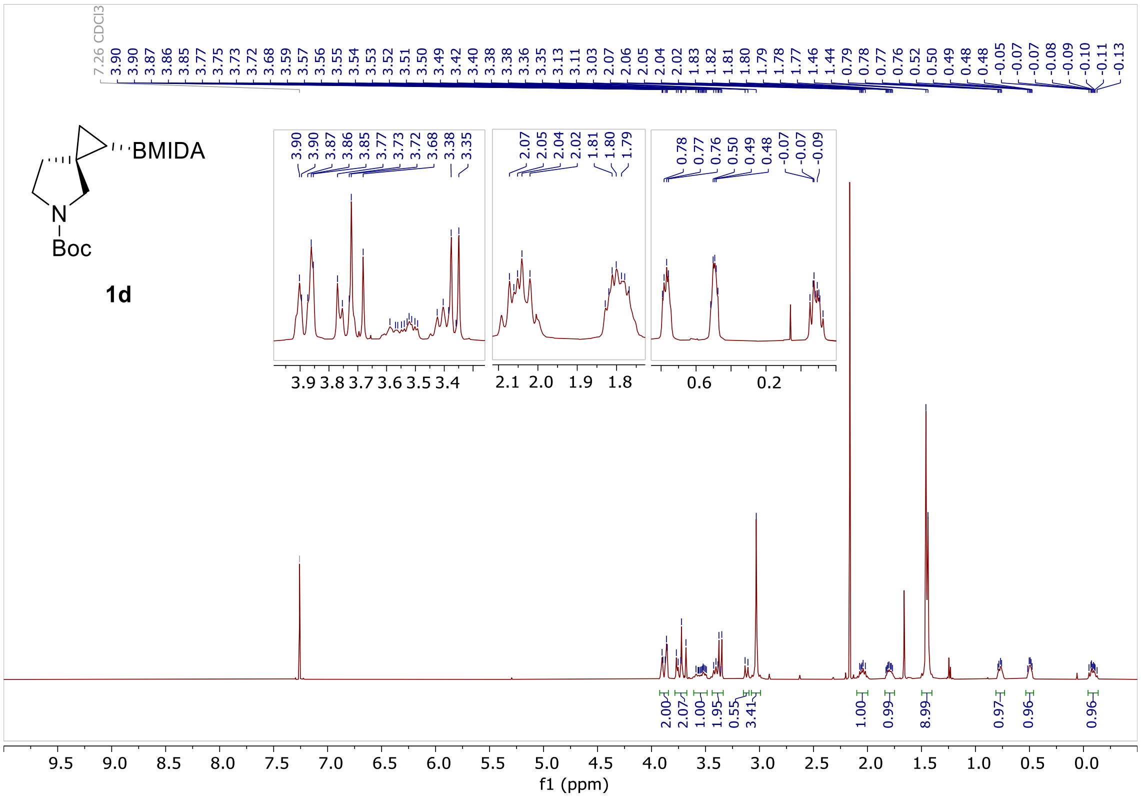

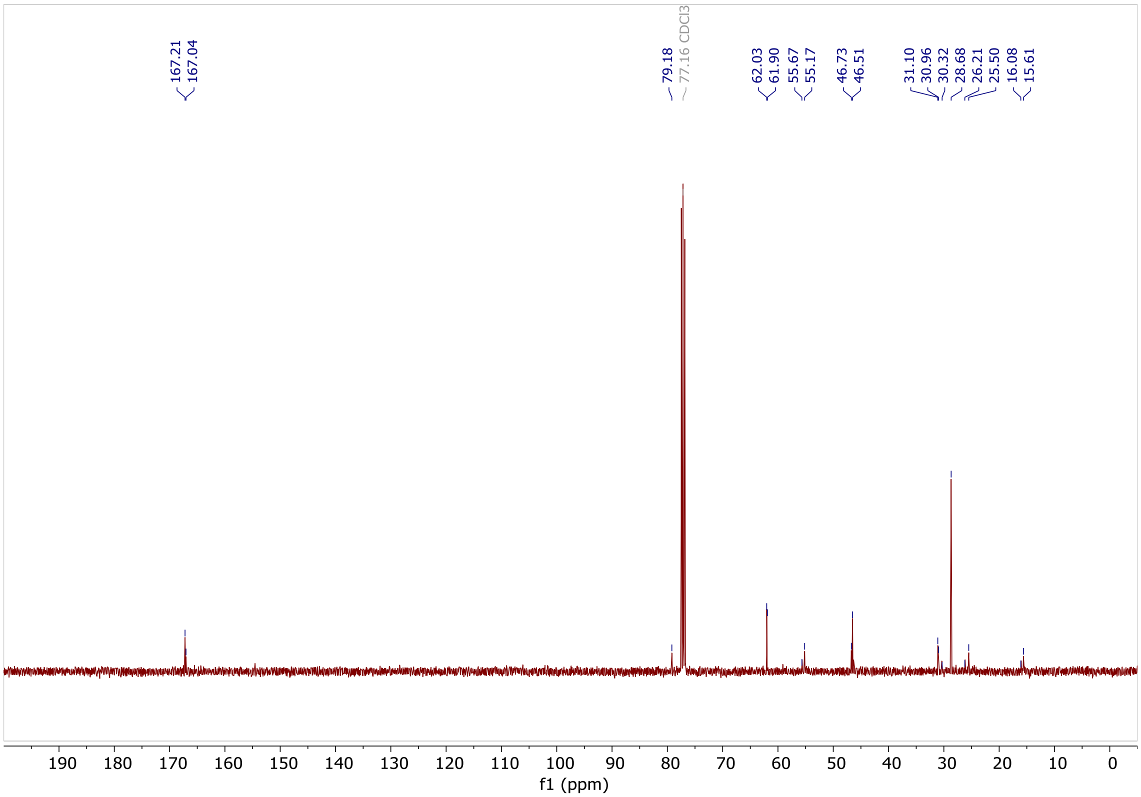

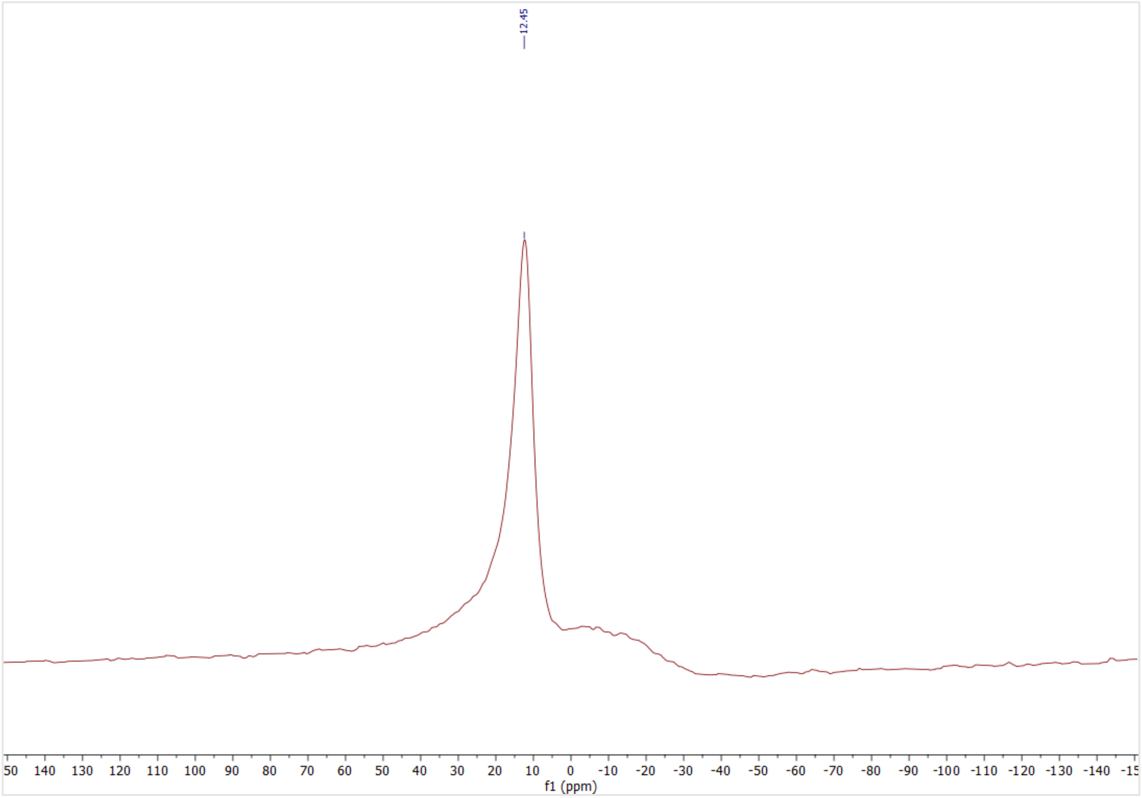

12.45
150 140 130 120 110 100 90 80 70 60 50 40 30 20 10 0 -10 -20 -30 -40 -50 -60 -70 -80 -90 -100 -110 -120 -130 -140 -150
f1 (ppm)

400 MHz ^{1}H NMR spectrum; 100.6 MHz ^{13}C NMR spectrum; 128.4 MHz ^{11}B NMR spectrum; $CDCl_3$ of **S18**

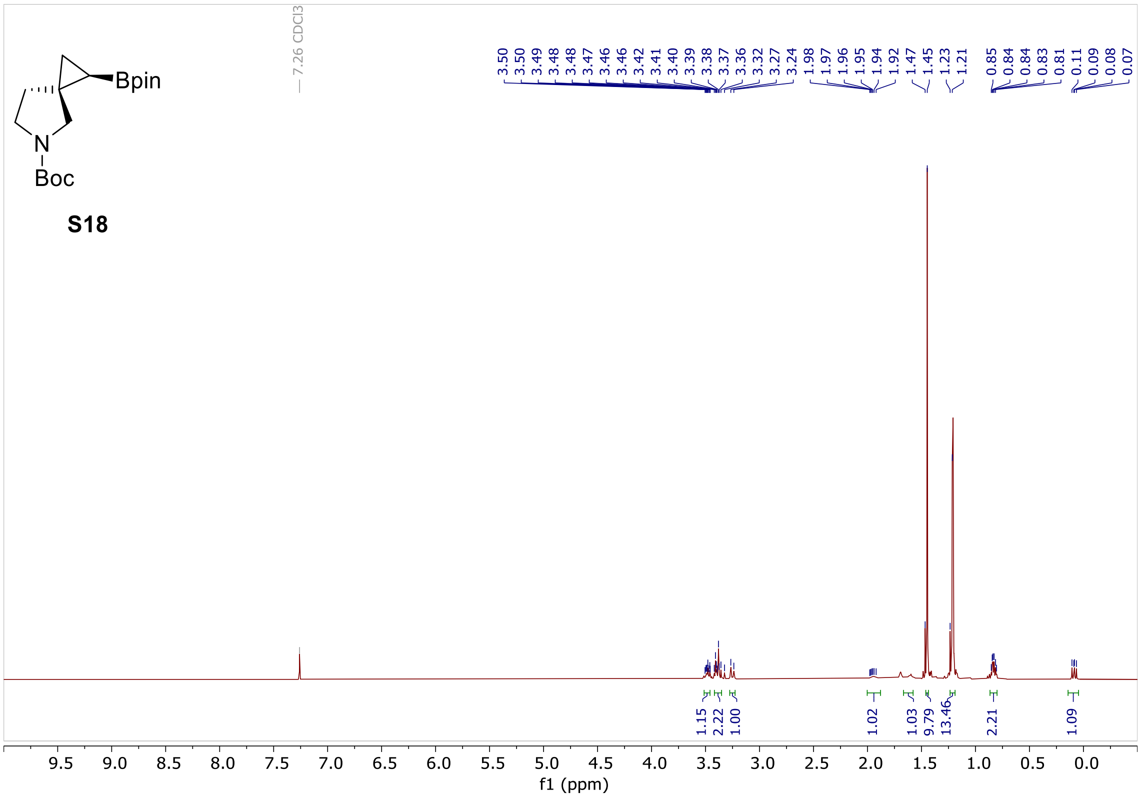

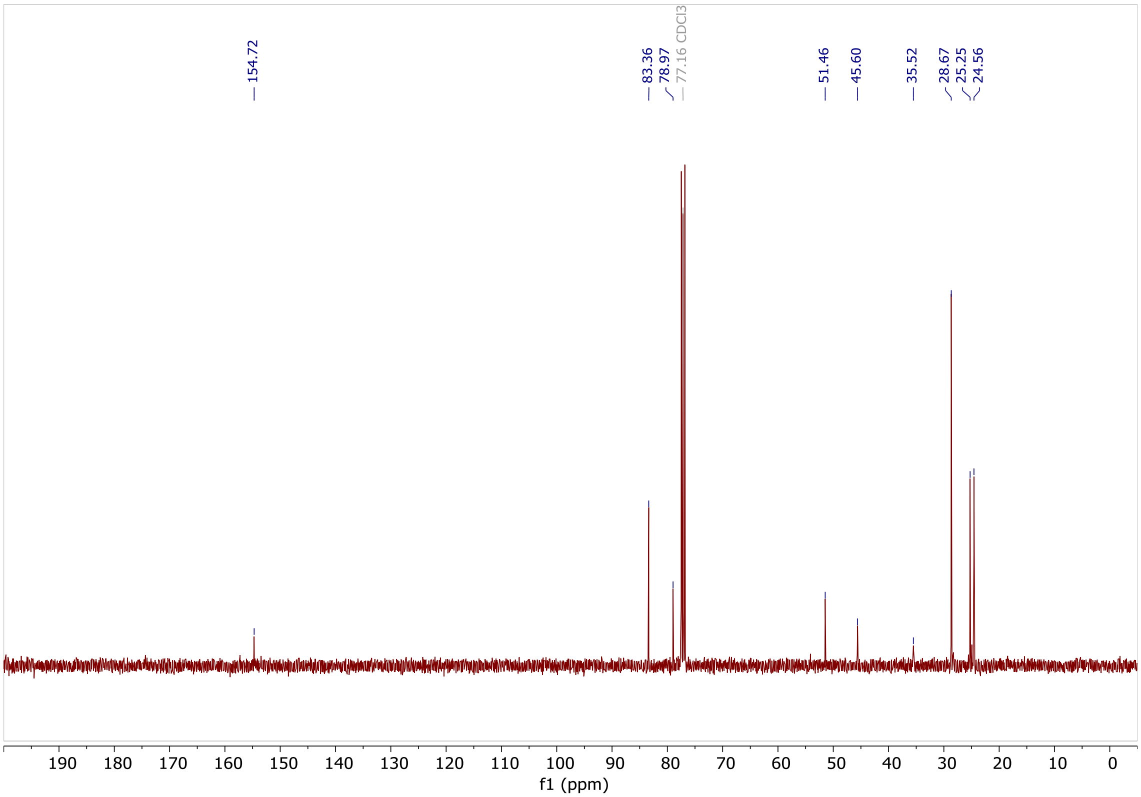

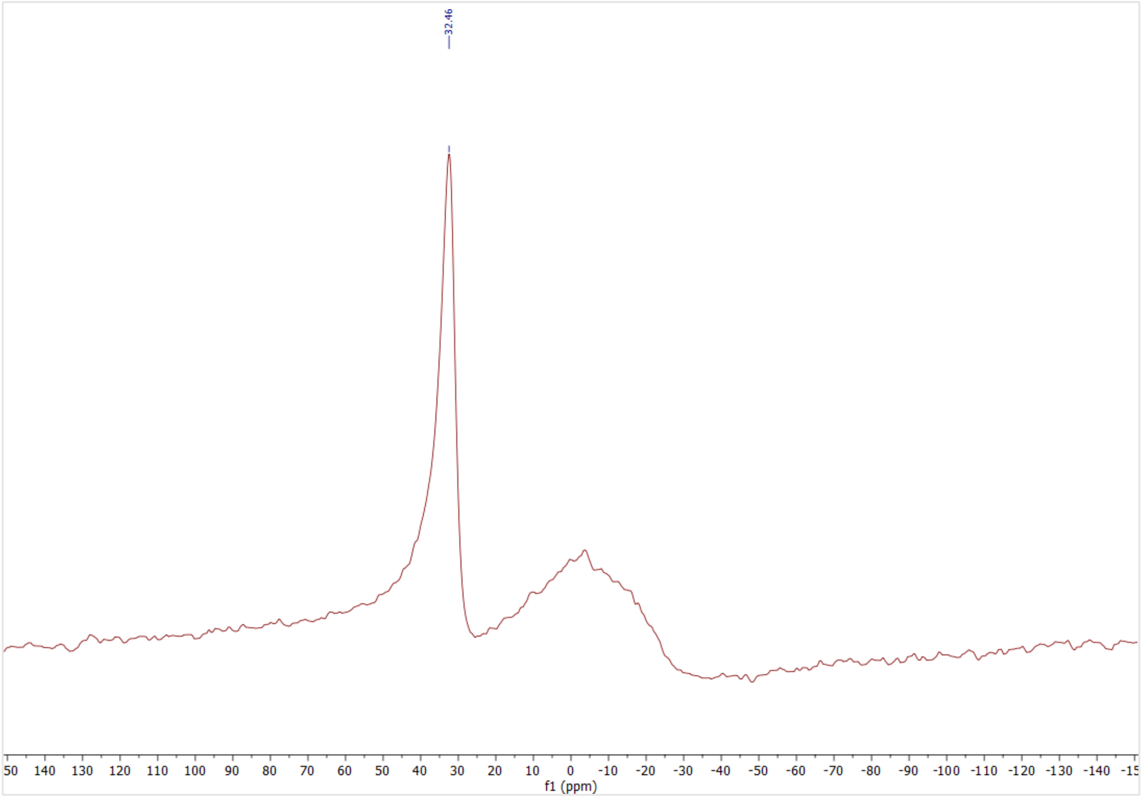
32.46
150
140
130
120
110
100
90
80
70
60
50
40
30
20
10
0
-10
-20
-30
-40
-50
-60
-70
-80
-90
-100
-110
-120
-130
-140
-150
f1 (ppm)

400 MHz ^{1}H NMR spectrum; 100.6 MHz ^{13}C NMR spectrum; 128.4 MHz ^{11}B NMR spectrum; $CDCl_3$ of **1e**

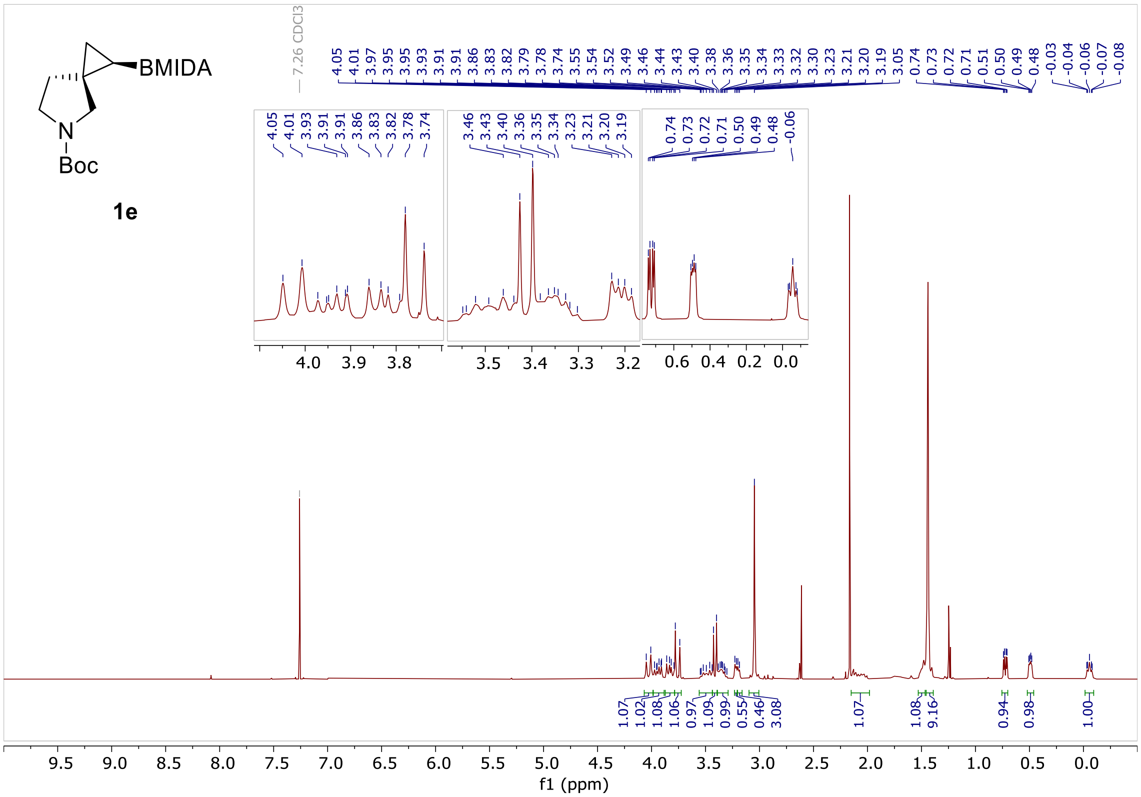

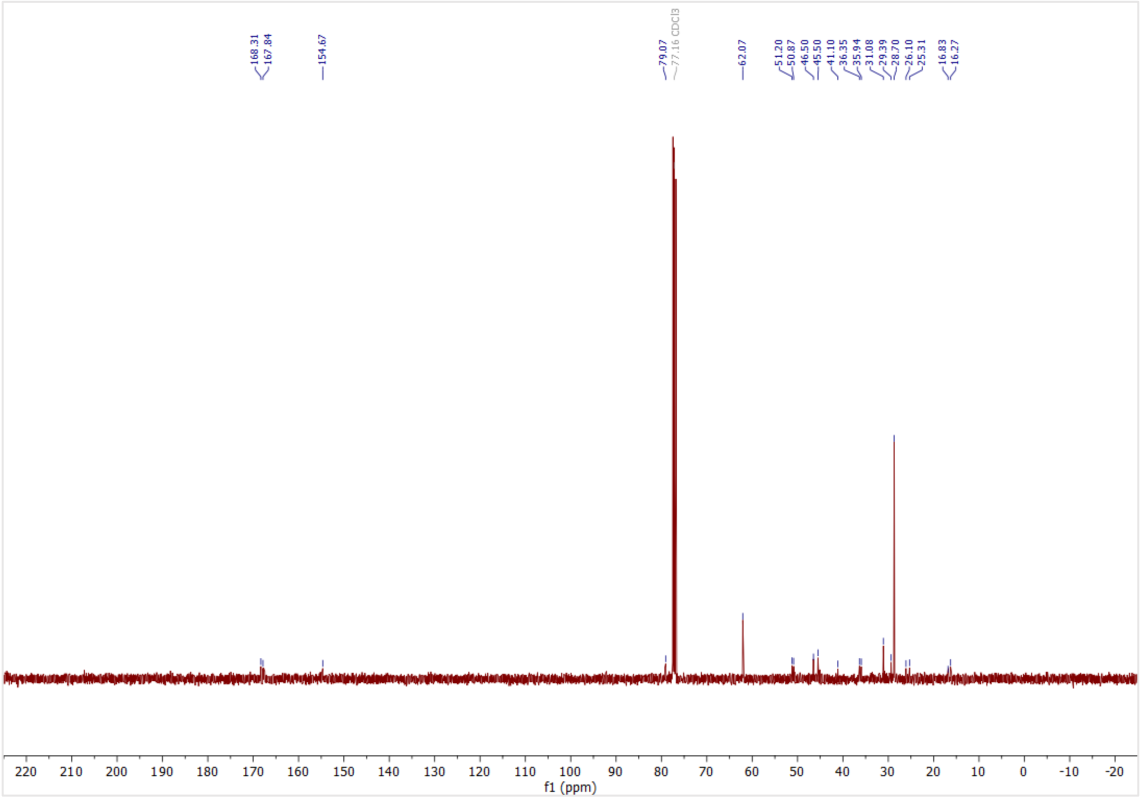

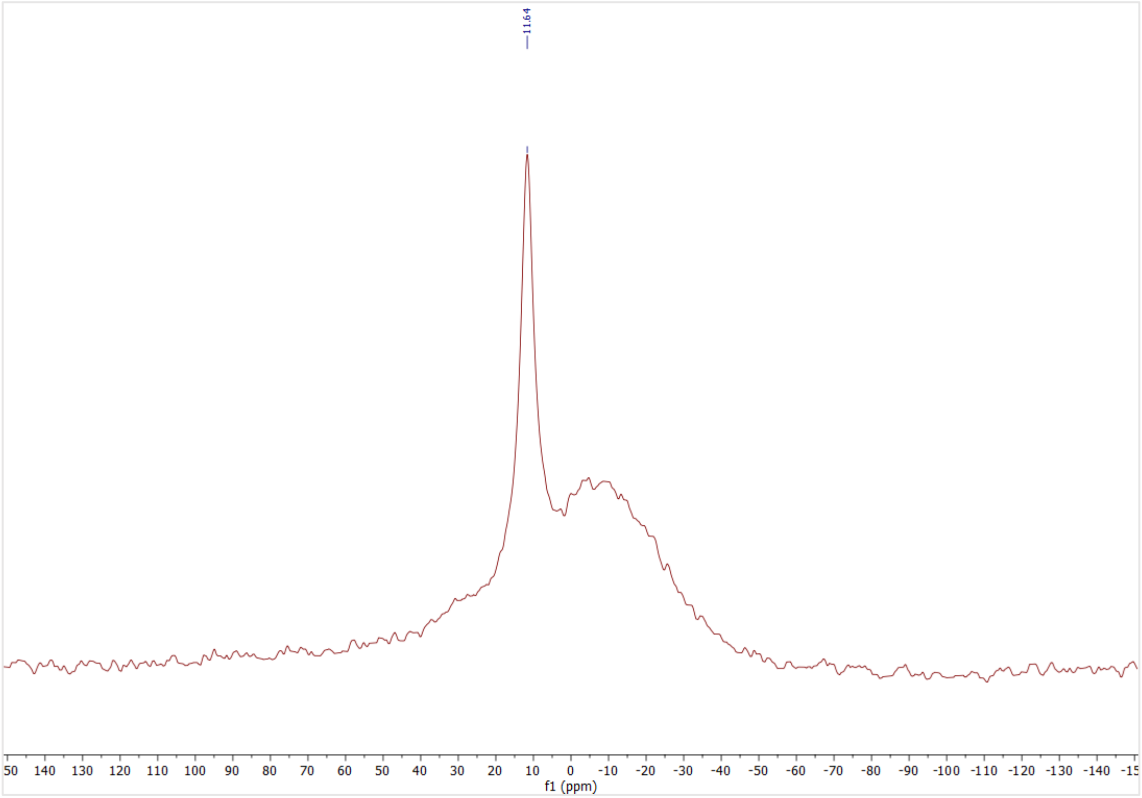
11.64
50 140 130 120 110 100 90 80 70 60 50 40 30 20 10 0 -10 -20 -30 -40 -50 -60 -70 -80 -90 -100 -110 -120 -130 -140 -15
f1 (ppm)

400 MHz ^{1}H NMR spectrum; 100.6 MHz ^{13}C NMR spectrum; $CDCl_3$ of **S19**

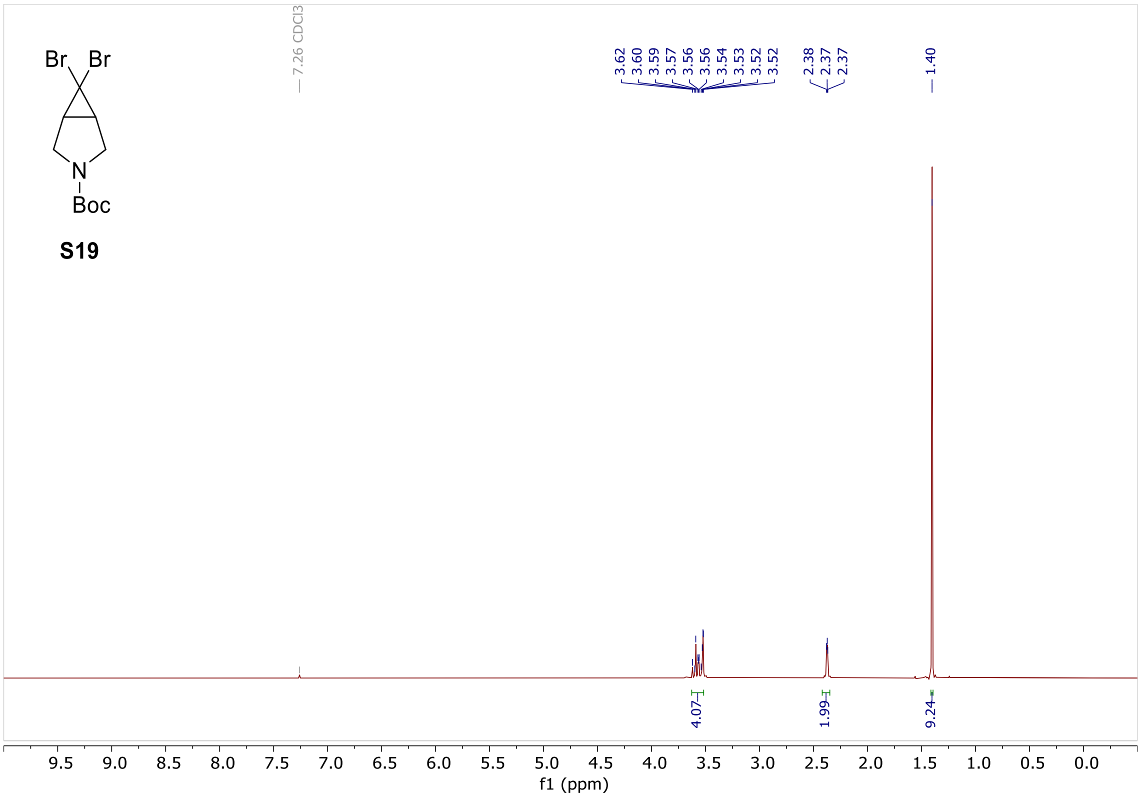

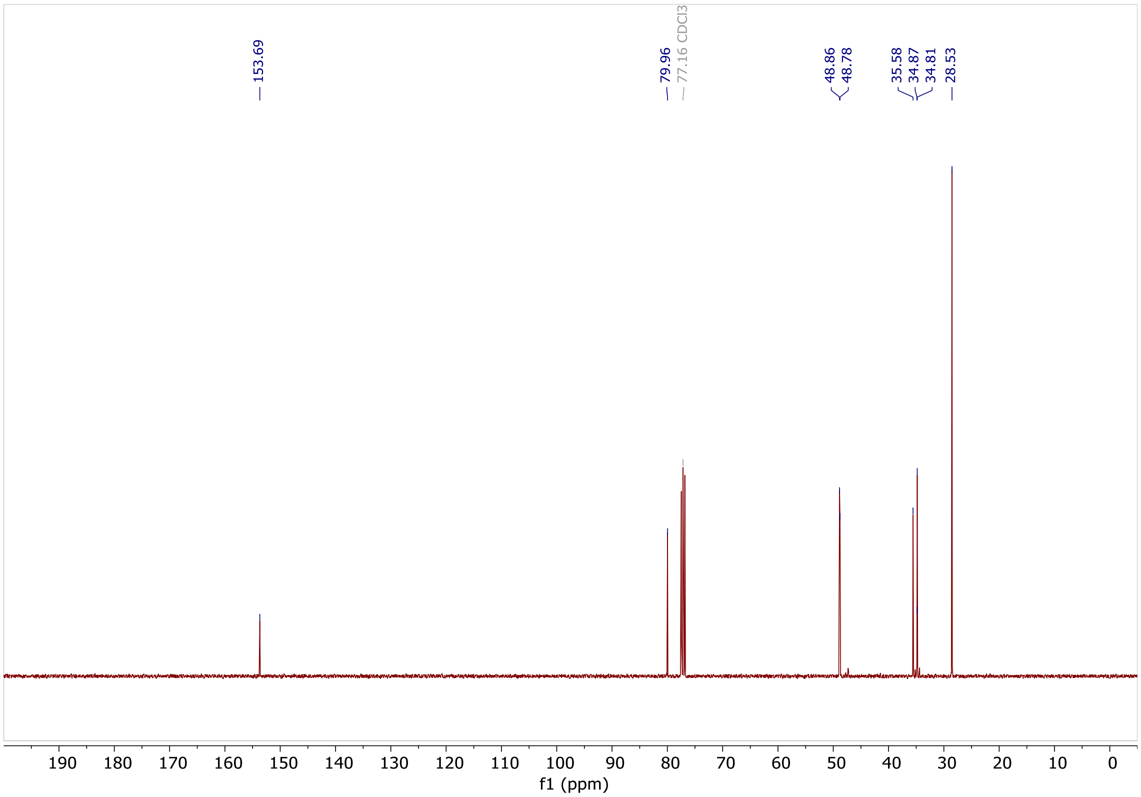

400 MHz ^{1}H NMR spectrum; 100.6 MHz ^{13}C NMR spectrum; $CDCl_3$ of **S20**

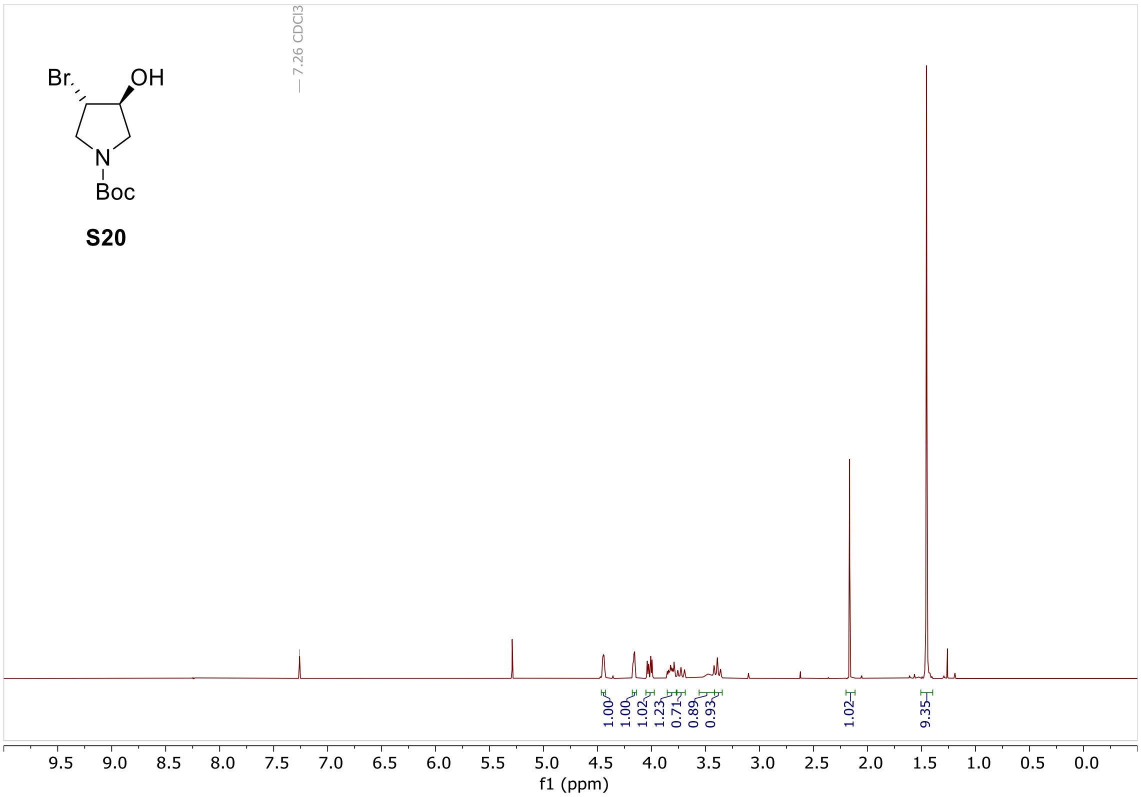

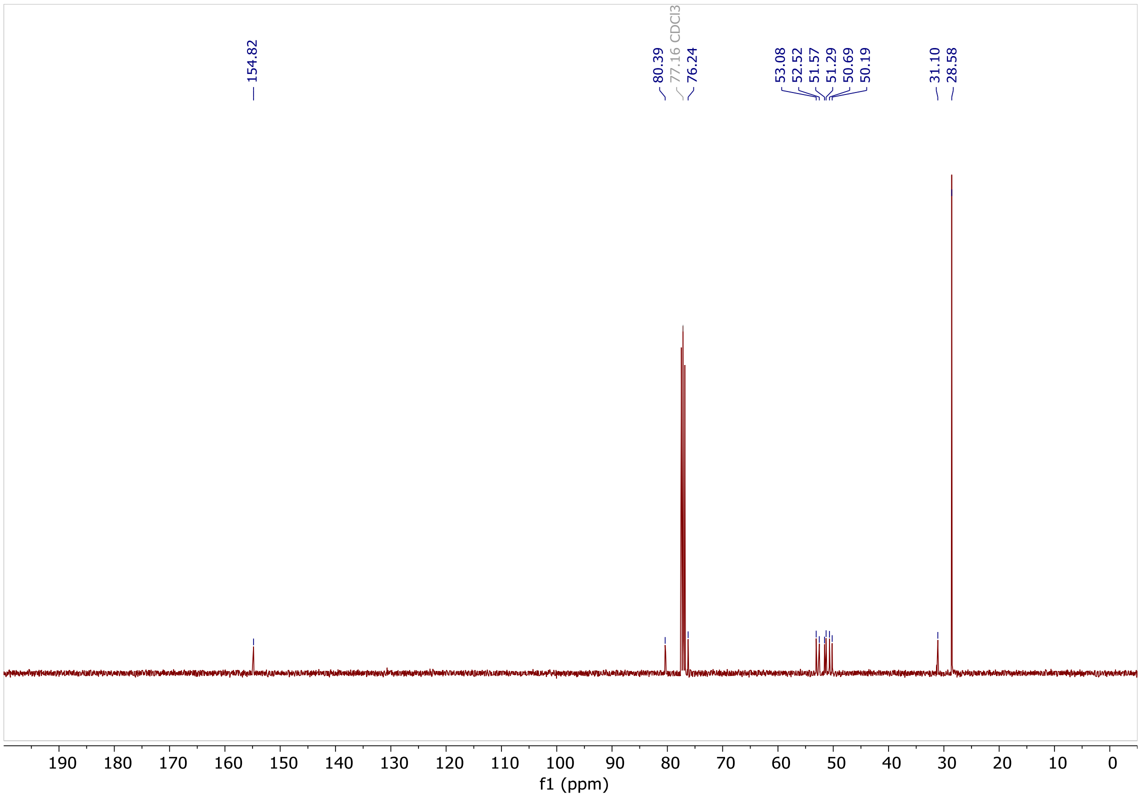

400 MHz ^{1}H NMR spectrum; 100.6 MHz ^{13}C NMR spectrum; $CDCl_3$ of **S21**

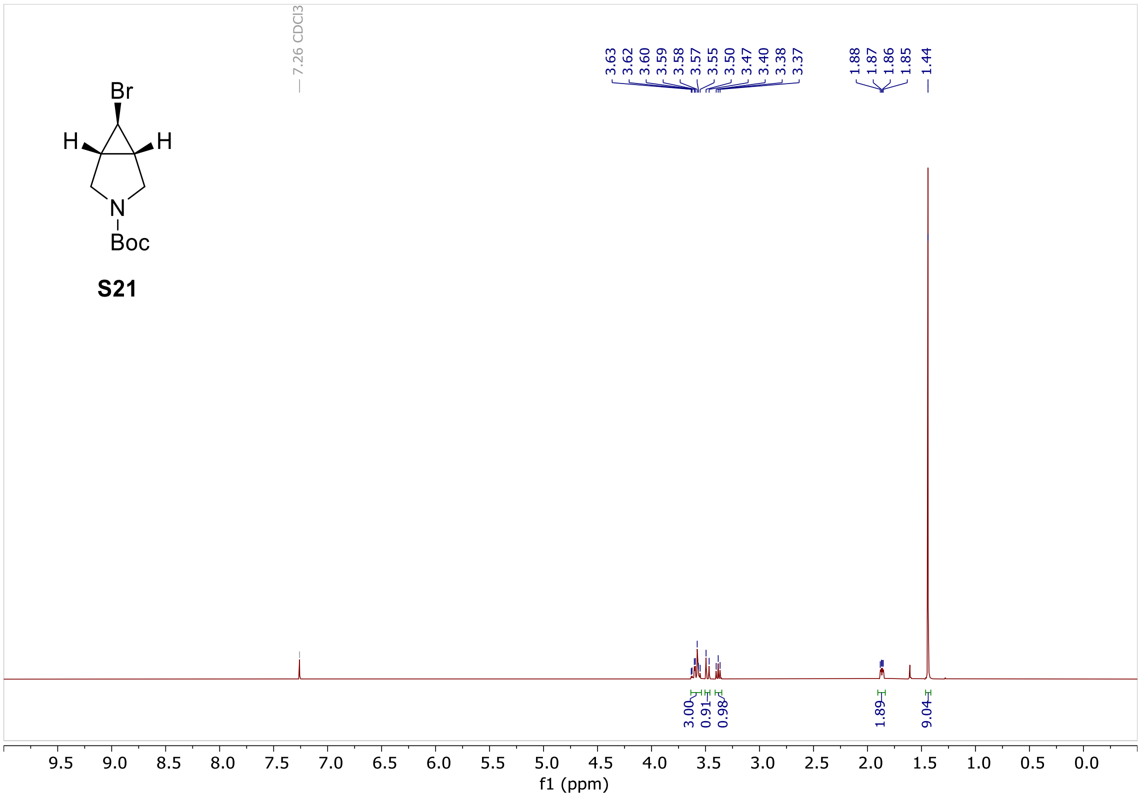

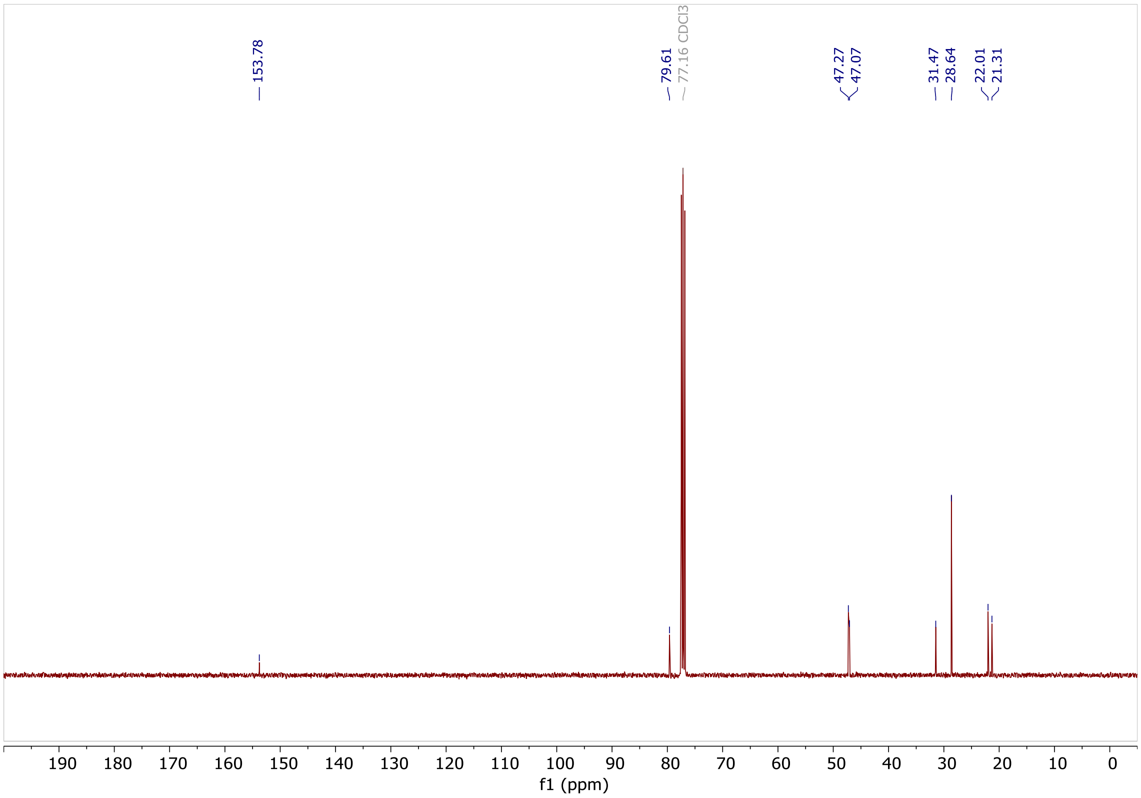

400 MHz ^{1}H NMR spectrum; 100.6 MHz ^{13}C NMR spectrum; $CDCl_3$ of **S22**

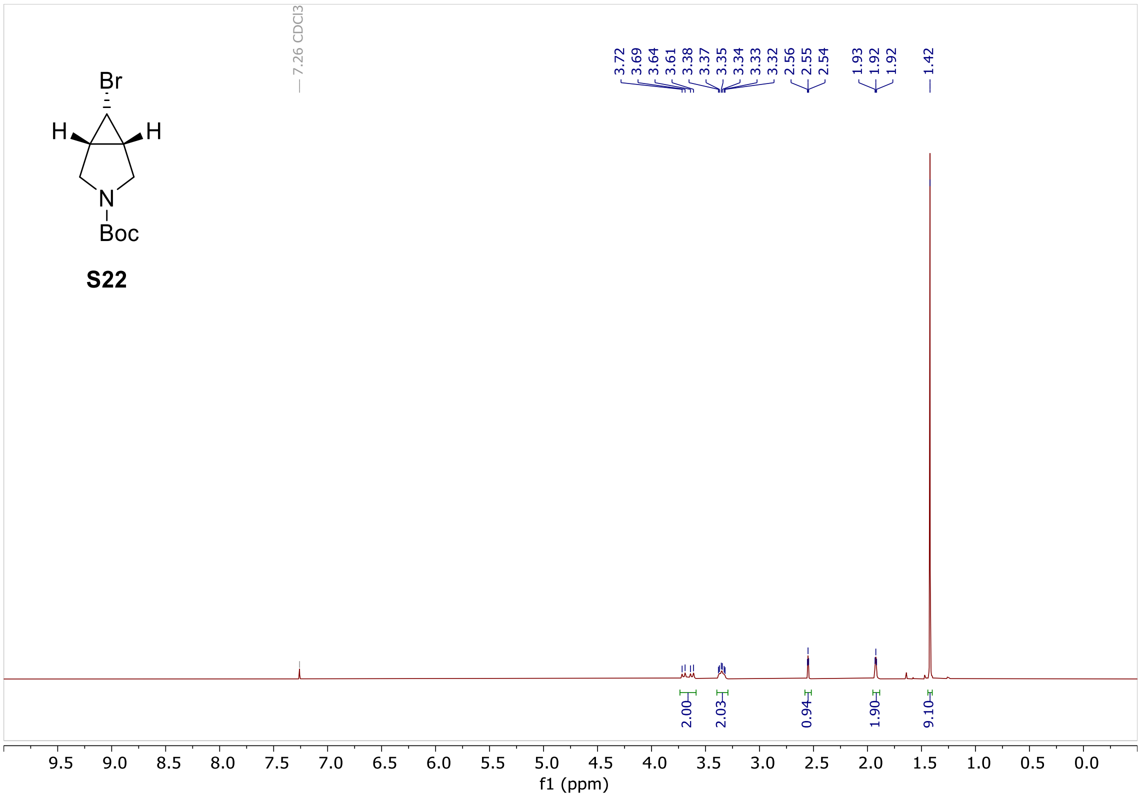

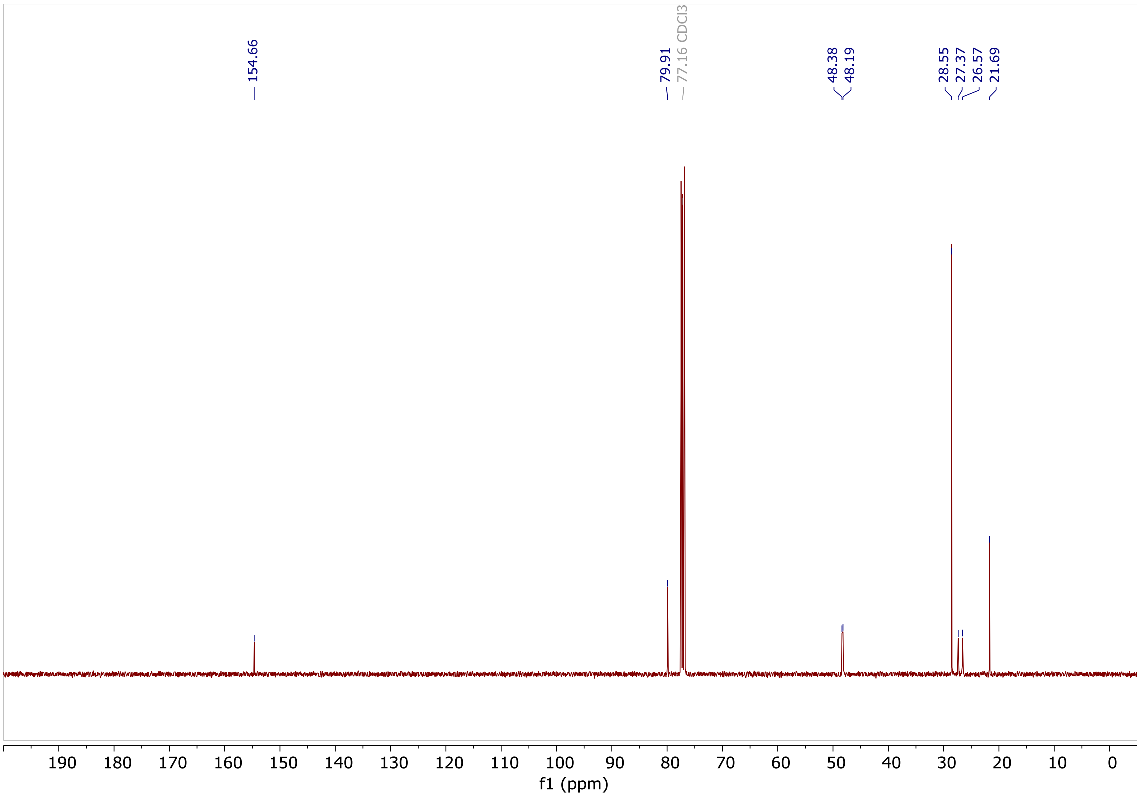

400 MHz ^{1}H NMR spectrum; 100.6 MHz ^{13}C NMR spectrum; 128.4 MHz ^{11}B NMR spectrum; $CDCl_3$ of **S23**

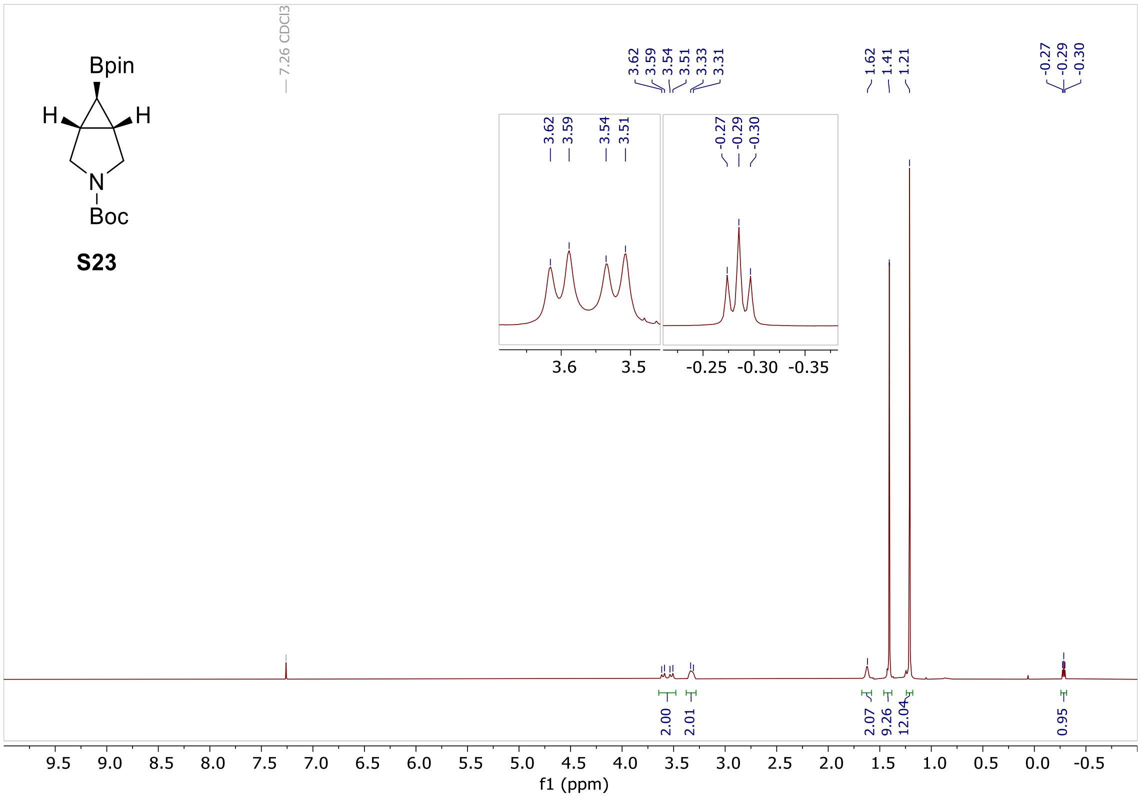

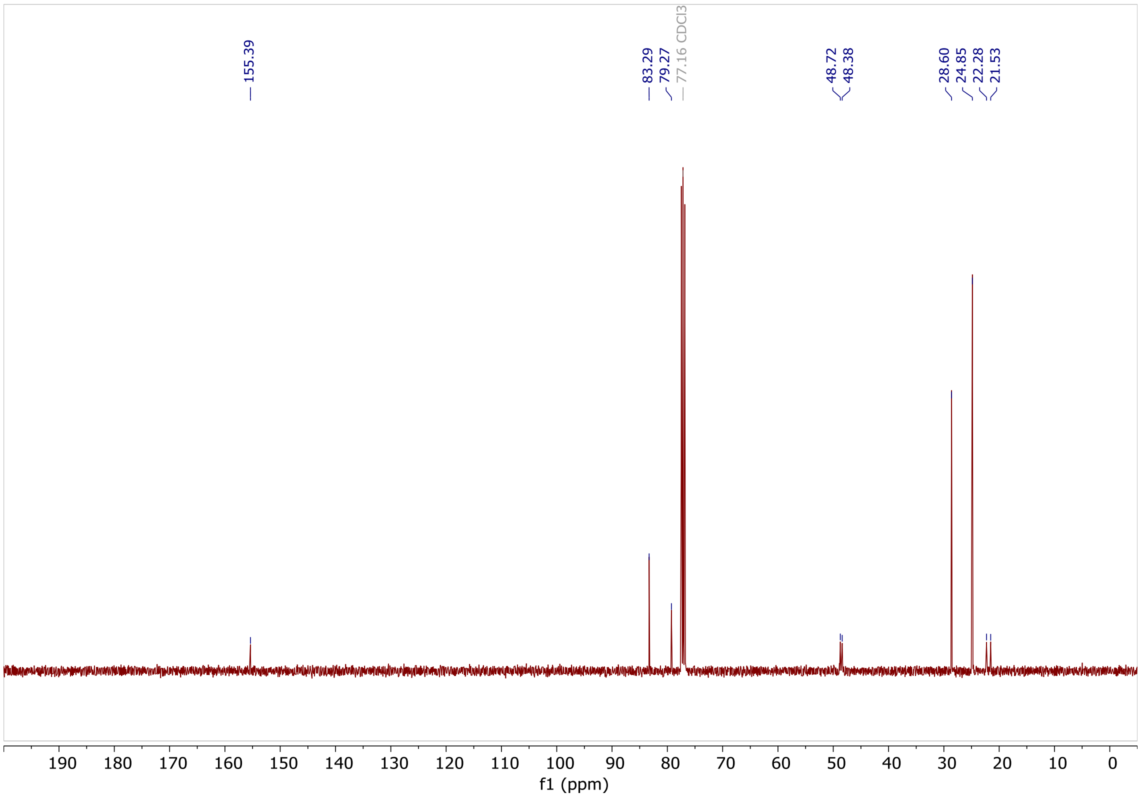

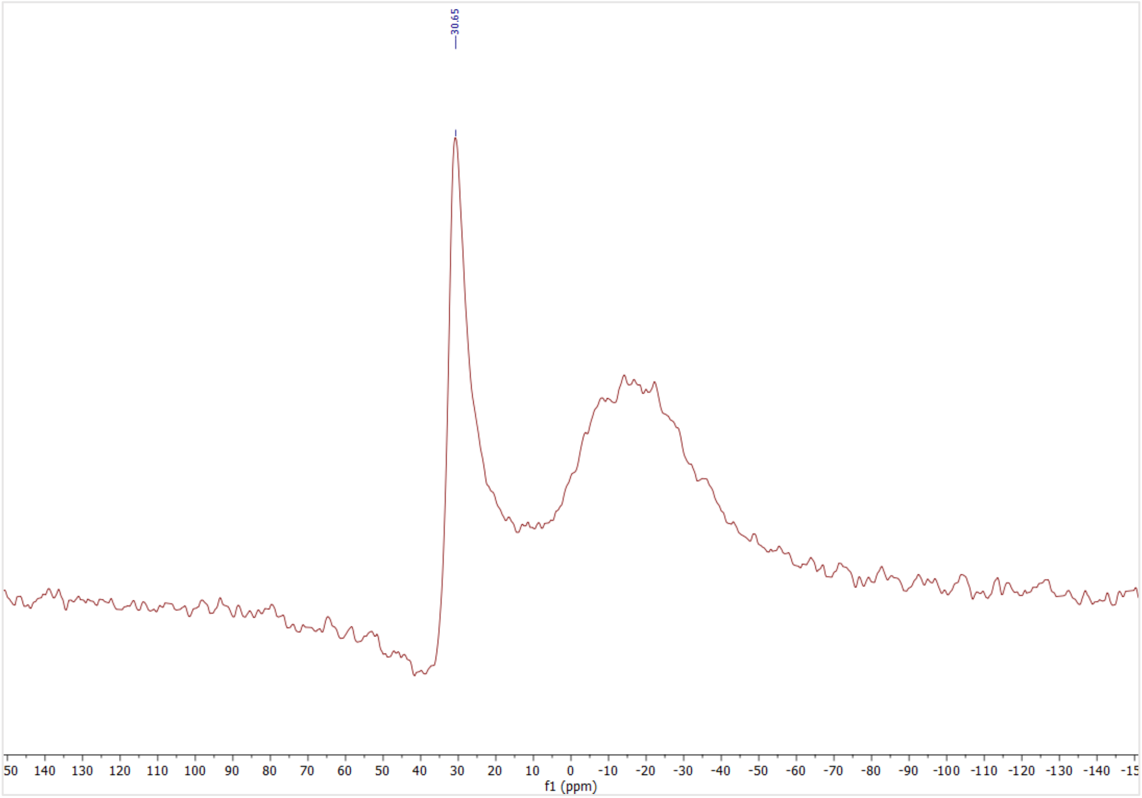
30.65
150 140 130 120 110 100 90 80 70 60 50 40 30 20 10 0 -10 -20 -30 -40 -50 -60 -70 -80 -90 -100 -110 -120 -130 -140 -15
f1 (ppm)

400 MHz ^{1}H NMR spectrum; 100.6 MHz ^{13}C NMR spectrum; 128.4 MHz ^{11}B NMR spectrum; $CDCl_3$ of **1h**

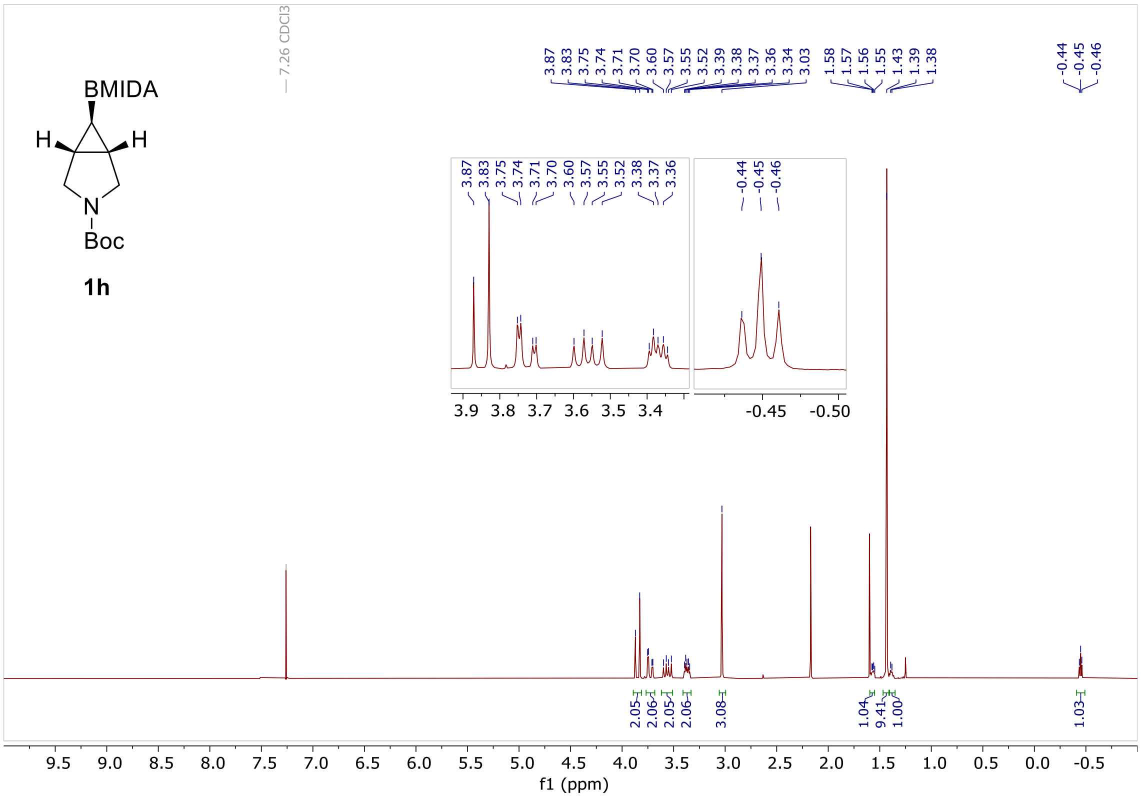

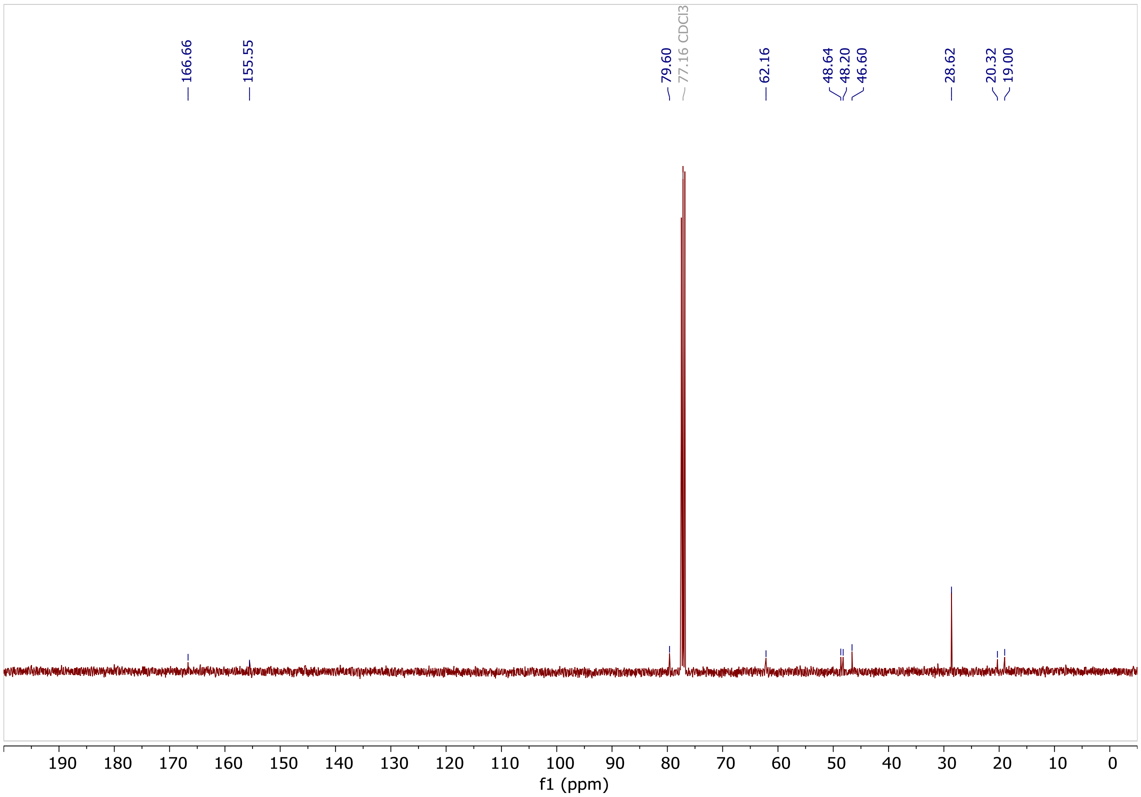

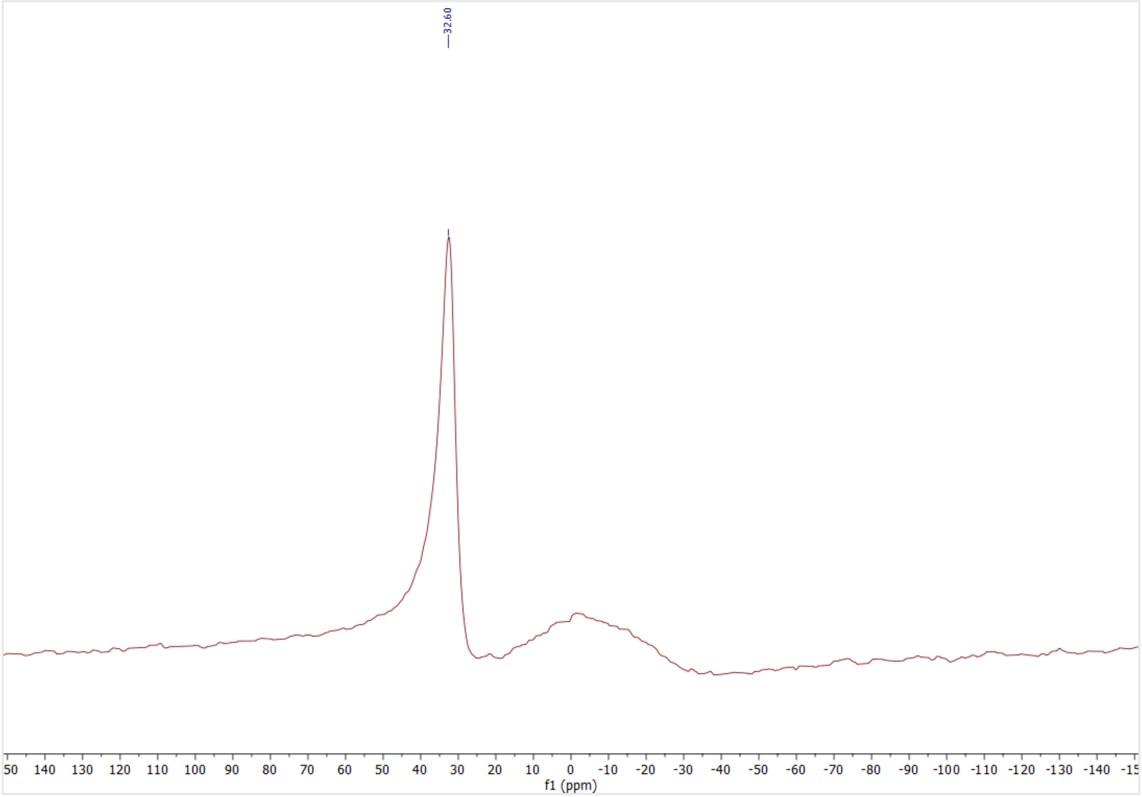
32.60
50
140
130
120
110
100
90
80
70
60
50
40
30
20
10
0
-10
-20
-30
-40
-50
-60
-70
-80
-90
-100
-110
-120
-130
-140
-15
f1 (ppm)

400 MHz ^{1}H NMR spectrum; 100.6 MHz ^{13}C NMR spectrum; $CDCl_3$ of **S26**

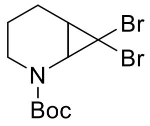

S26

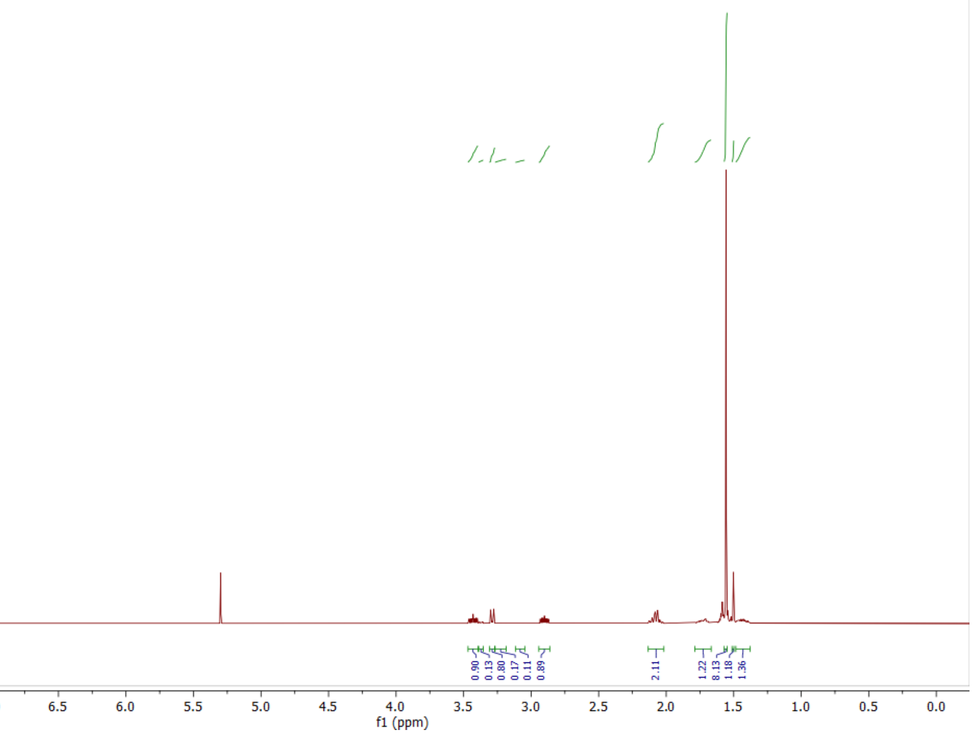

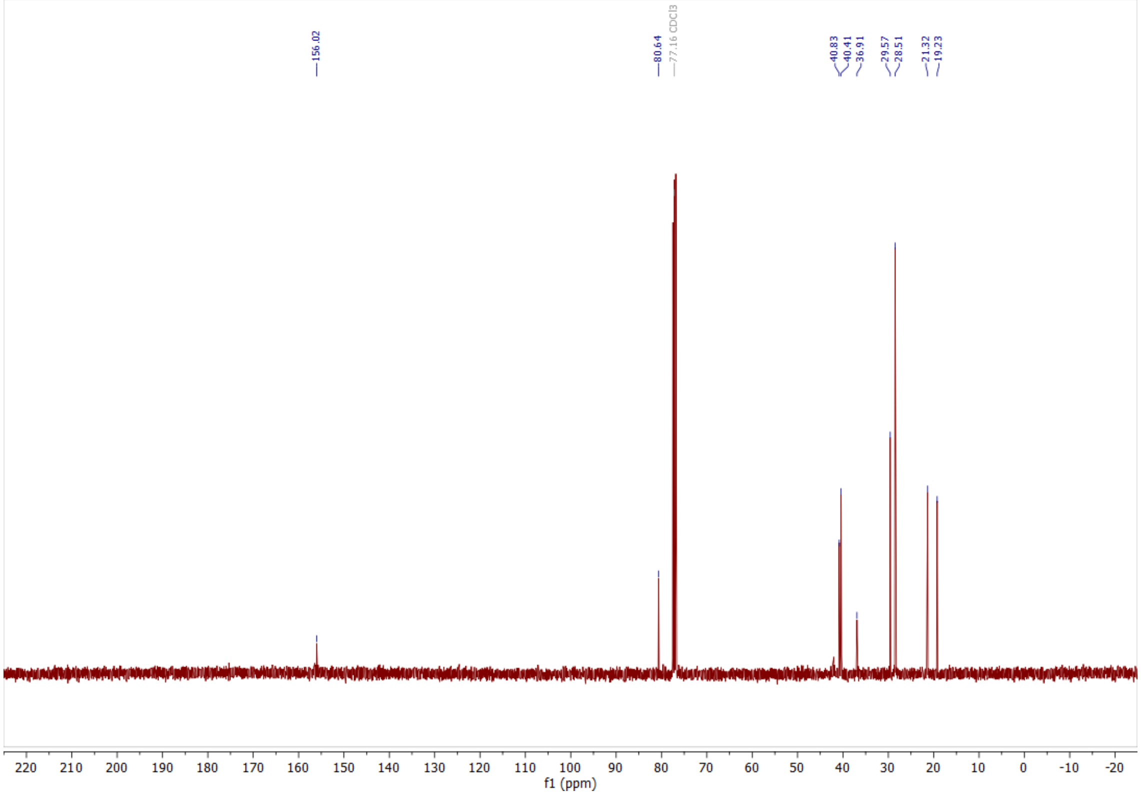

400 MHz ^{1}H NMR spectrum; 100.6 MHz ^{13}C NMR spectrum; $CDCl_3$ of **S27**

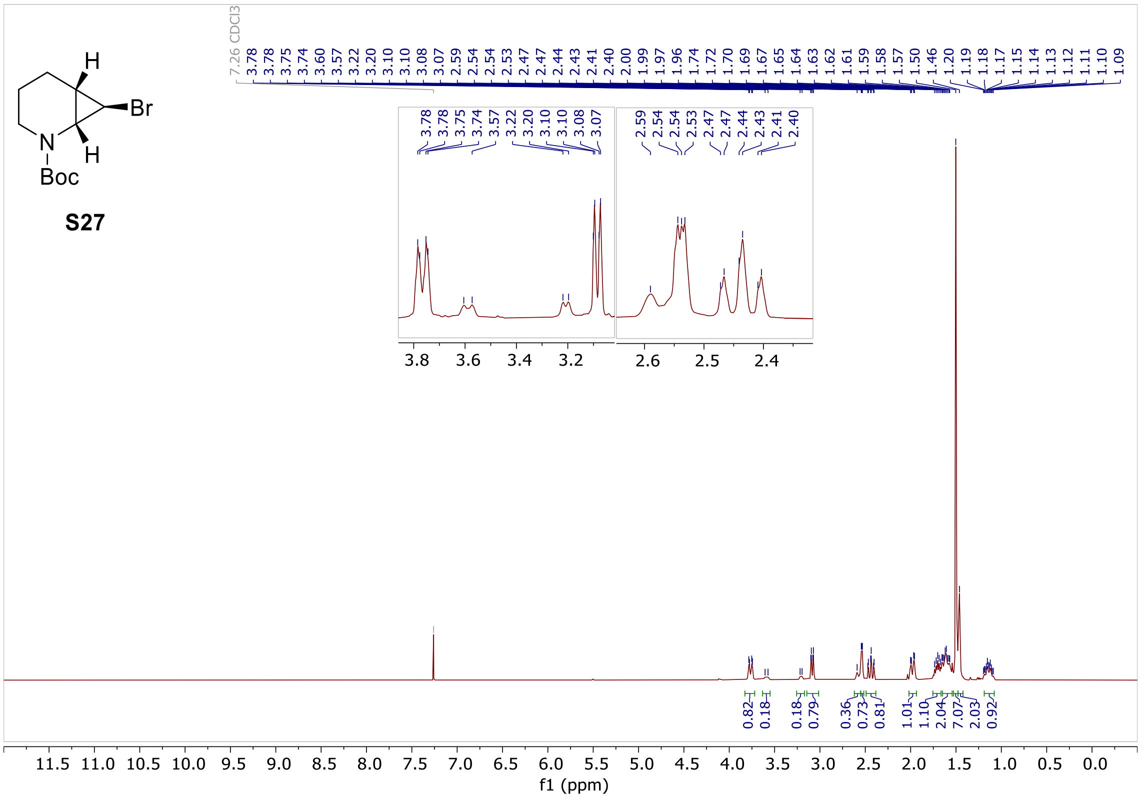

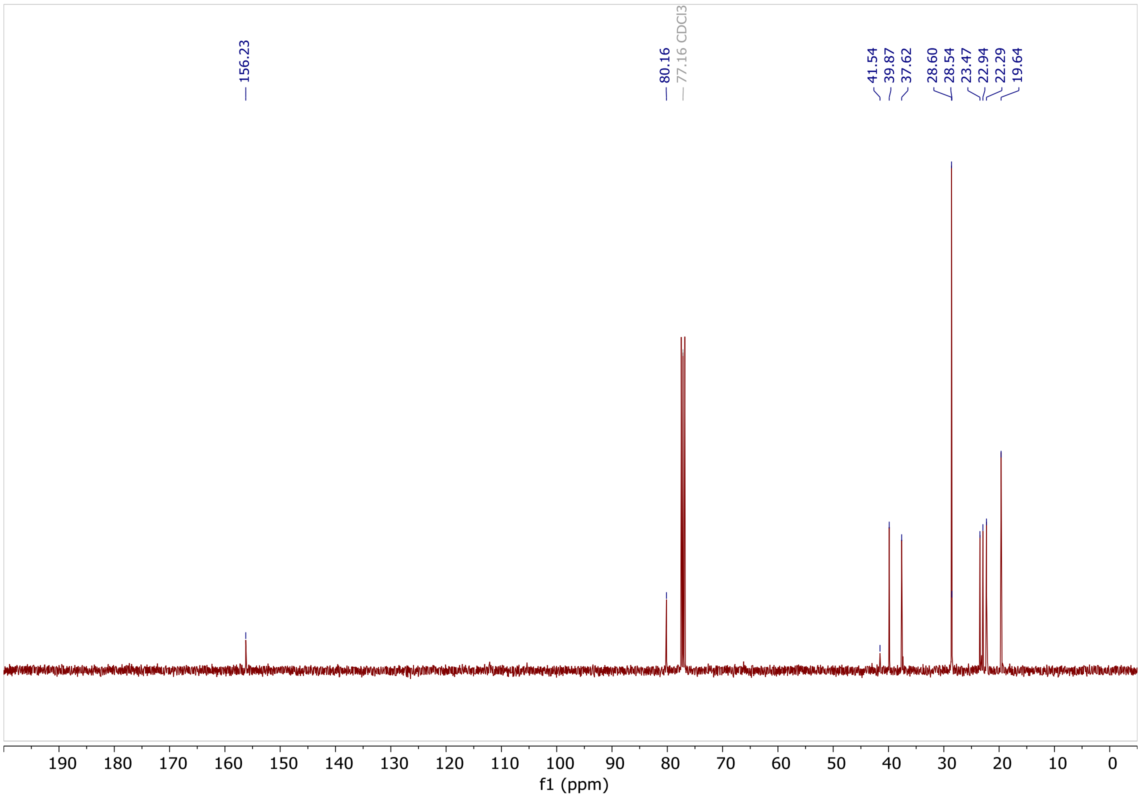

400 MHz ^{1}H NMR spectrum; 100.6 MHz ^{13}C NMR spectrum; 128.4 MHz ^{11}B spectrum; $CDCl_3$ of **S28**

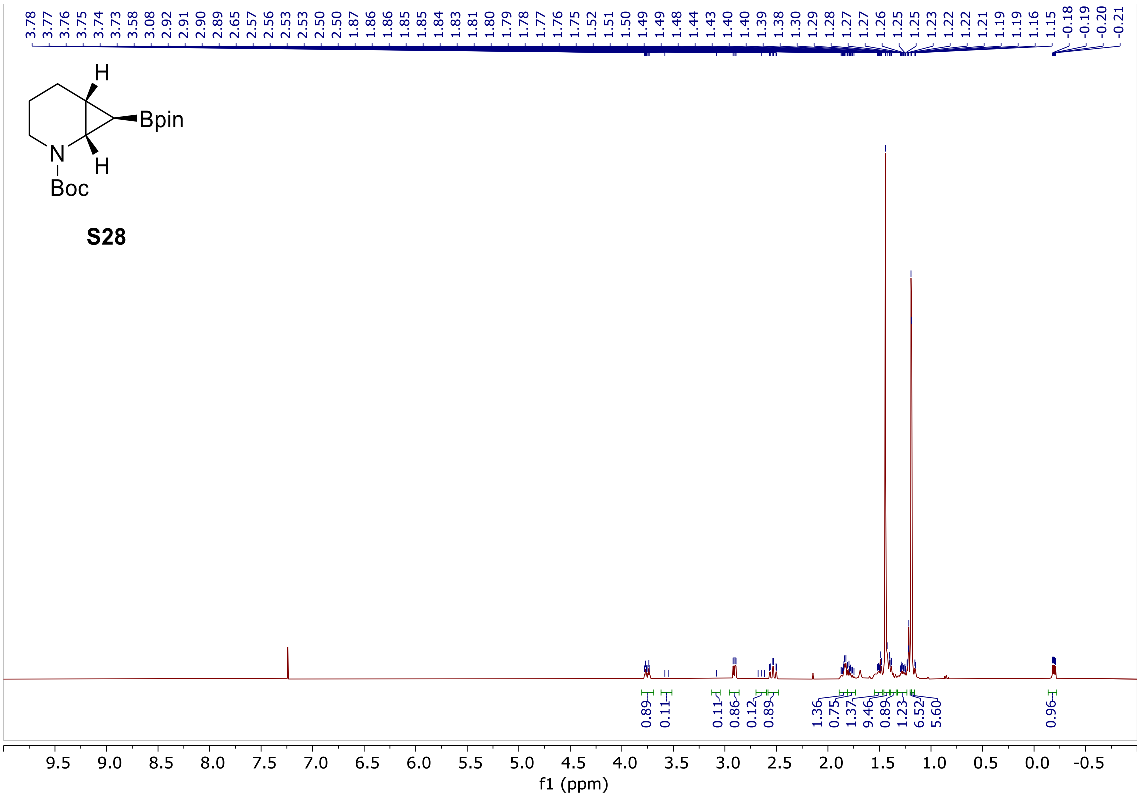

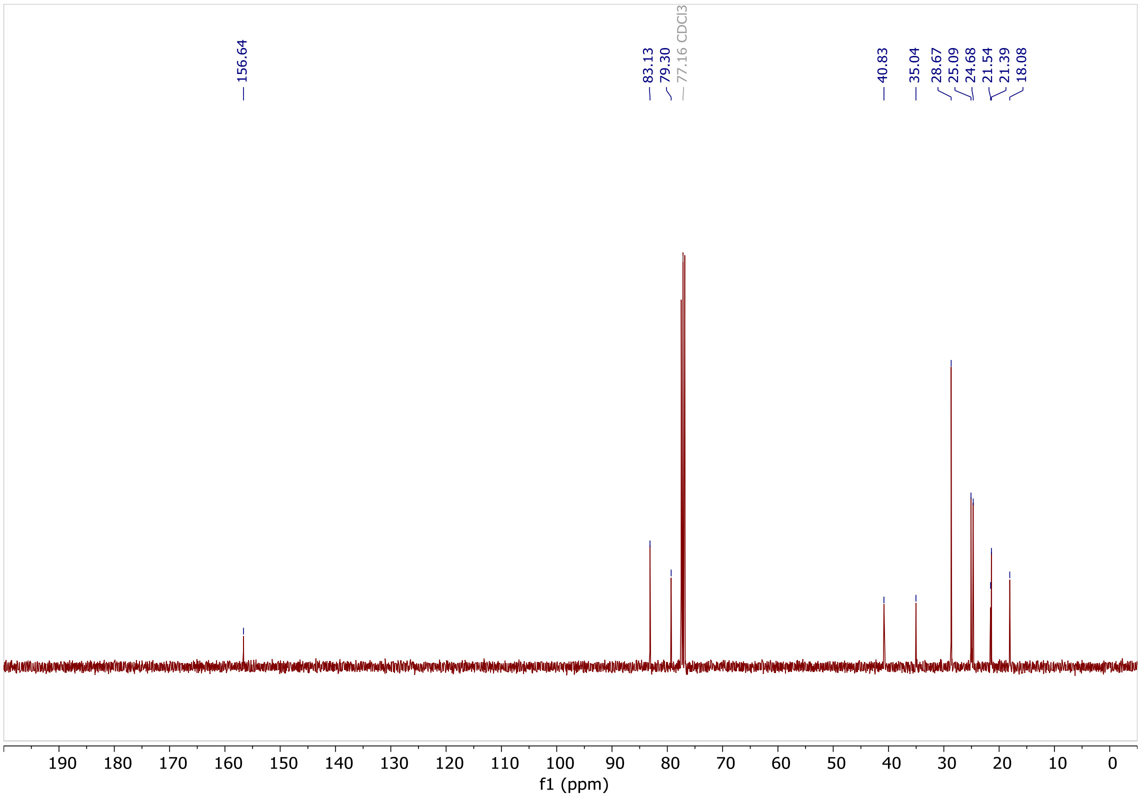

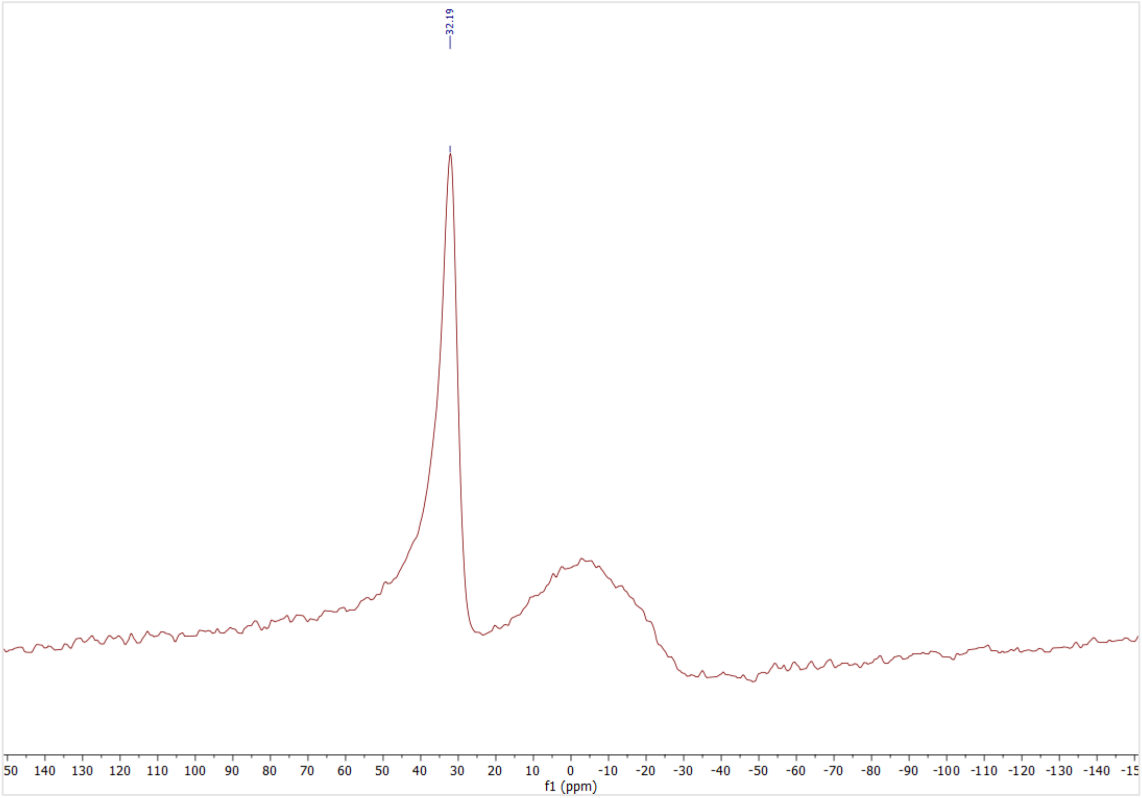

32.19
50
140
130
120
110
100
90
80
70
60
50
40
30
20
10
0
-10
-20
-30
-40
-50
-60
-70
-80
-90
-100
-110
-120
-130
-140
f1 (ppm)

400 MHz ^{1}H NMR spectrum; 100.6 MHz ^{13}C NMR spectrum; 128.4 MHz ^{11}B spectrum; $CDCl_3$ of **1i**

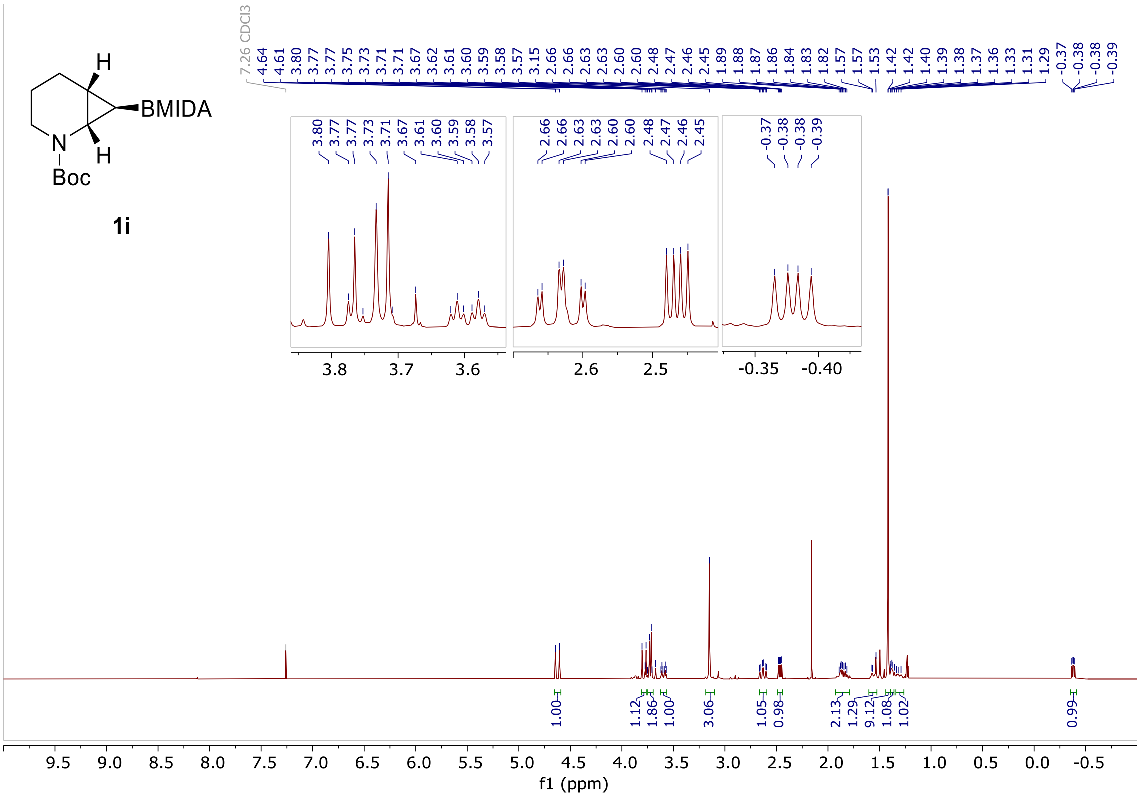

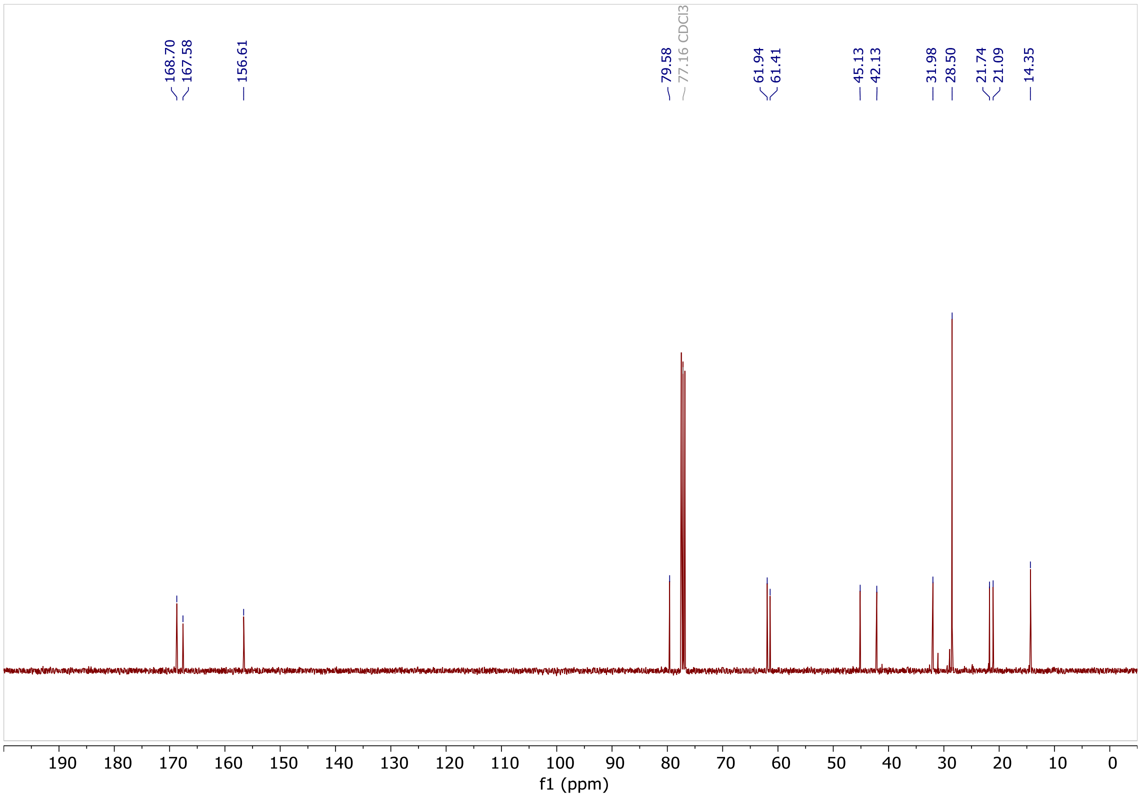

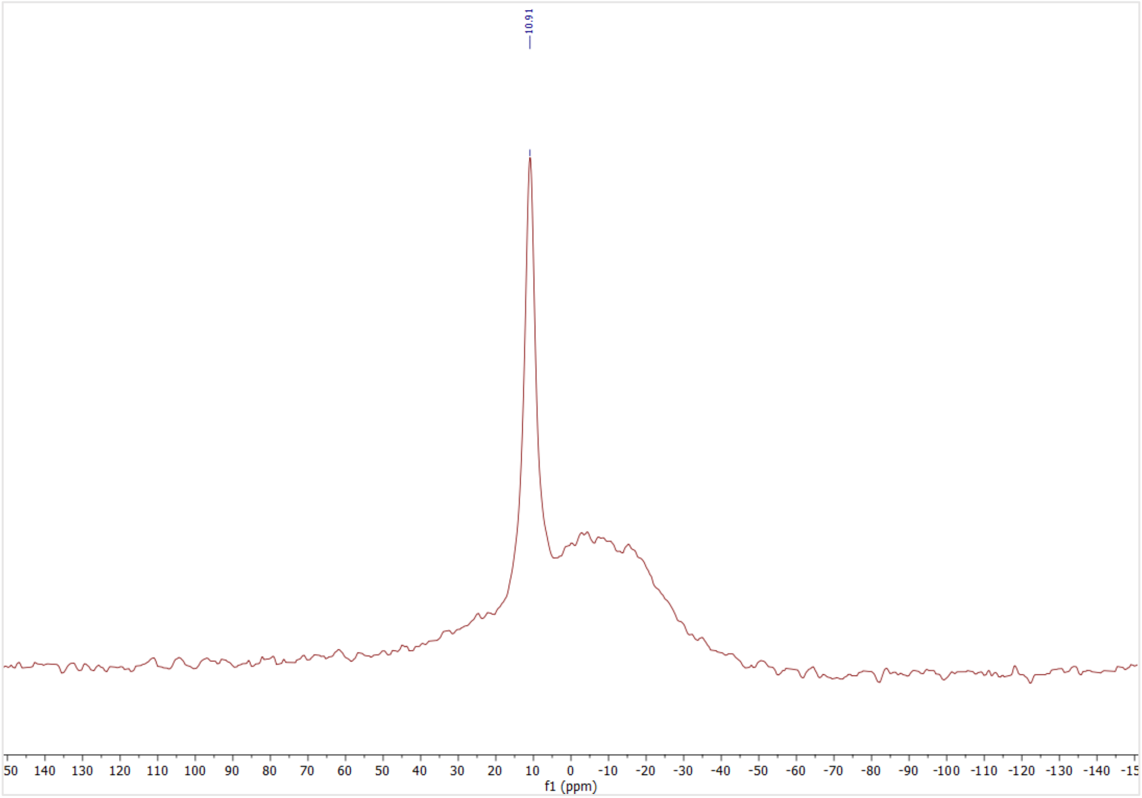

10.91
150 140 130 120 110 100 90 80 70 60 50 40 30 20 10 0 -10 -20 -30 -40 -50 -60 -70 -80 -90 -100 -110 -120 -130 -140 -15
f1 (ppm)

400 MHz ^{1}H NMR spectrum; 100.6 MHz ^{13}C NMR spectrum; $CDCl_3$ of **12**

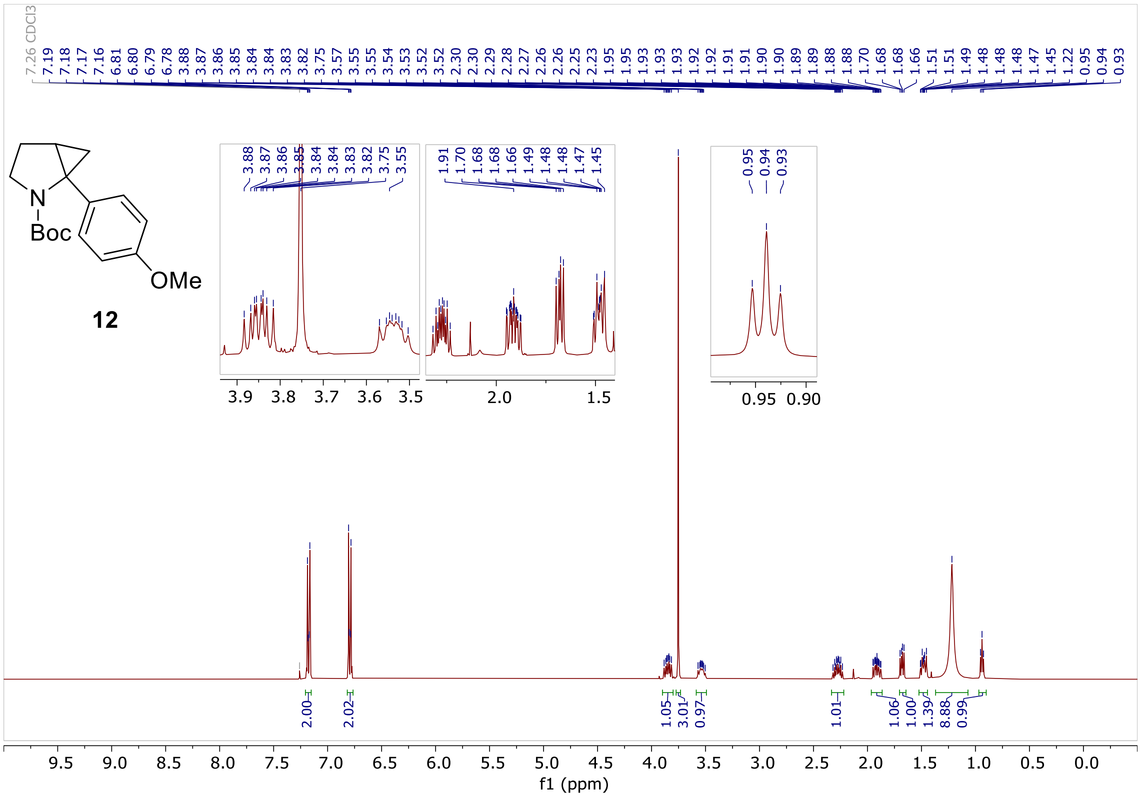

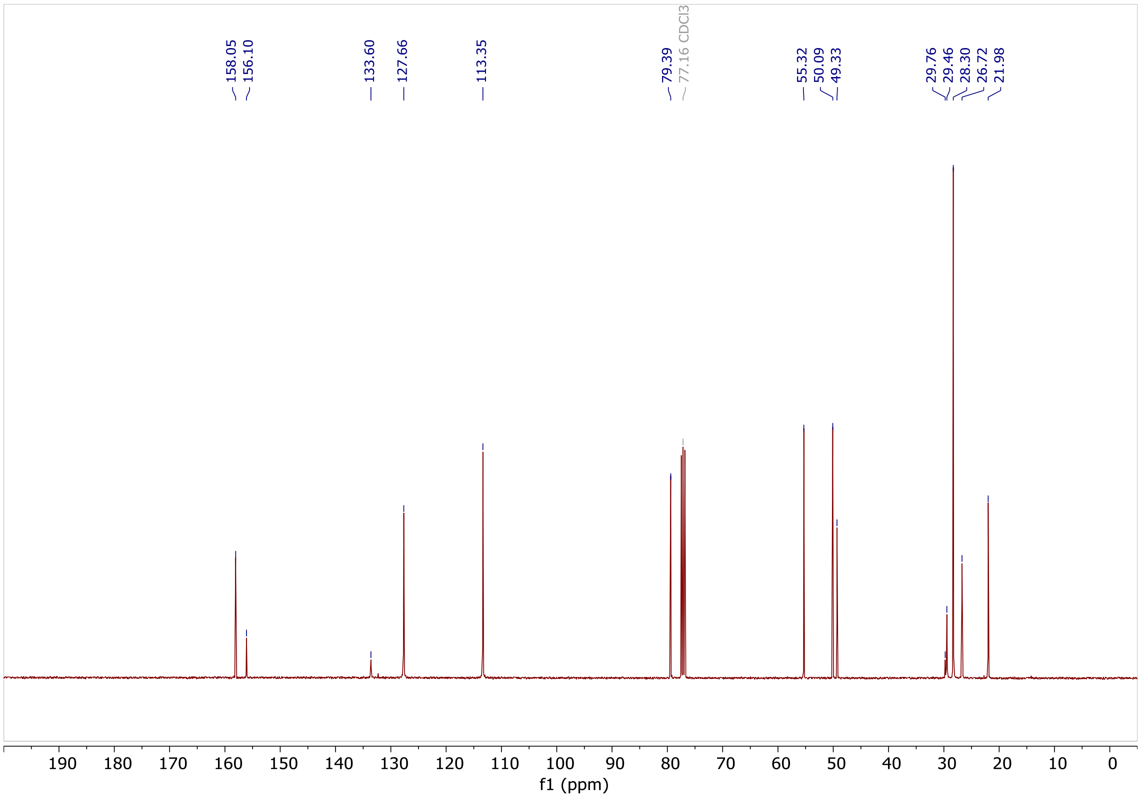

400 MHz ^{1}H NMR spectrum; 100.6 MHz ^{13}C NMR spectrum; $CDCl_3$ of **13**

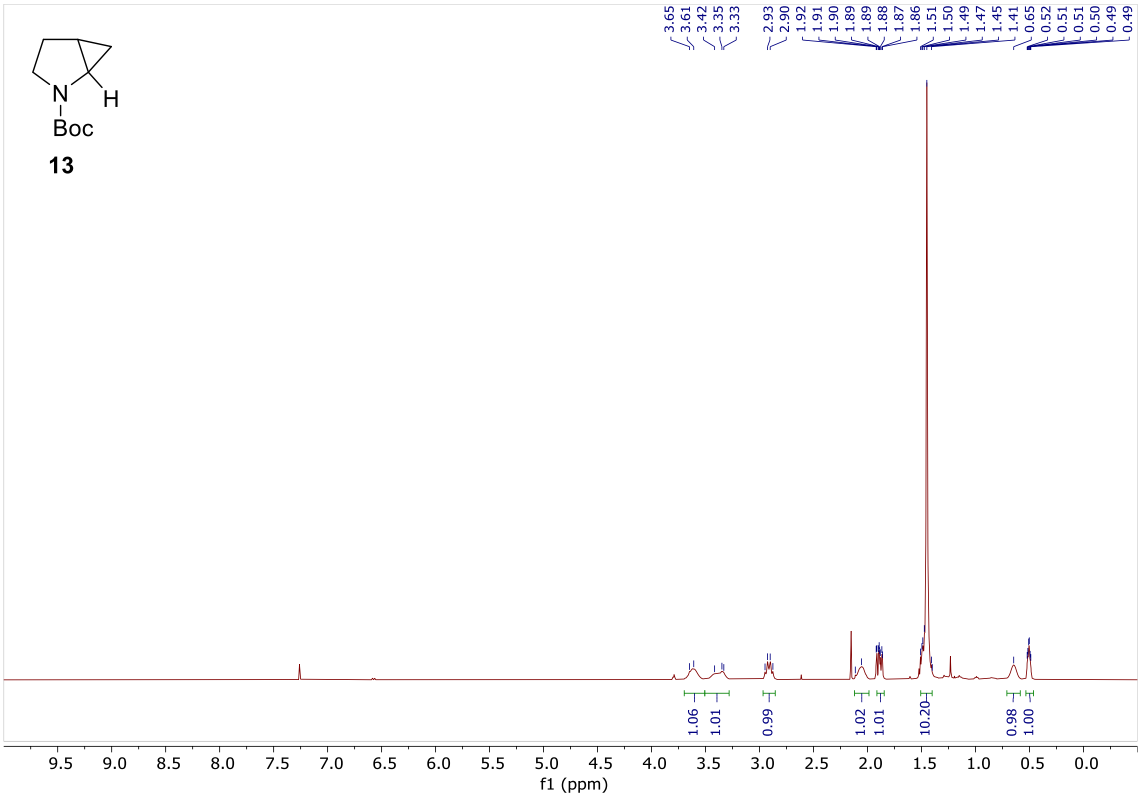

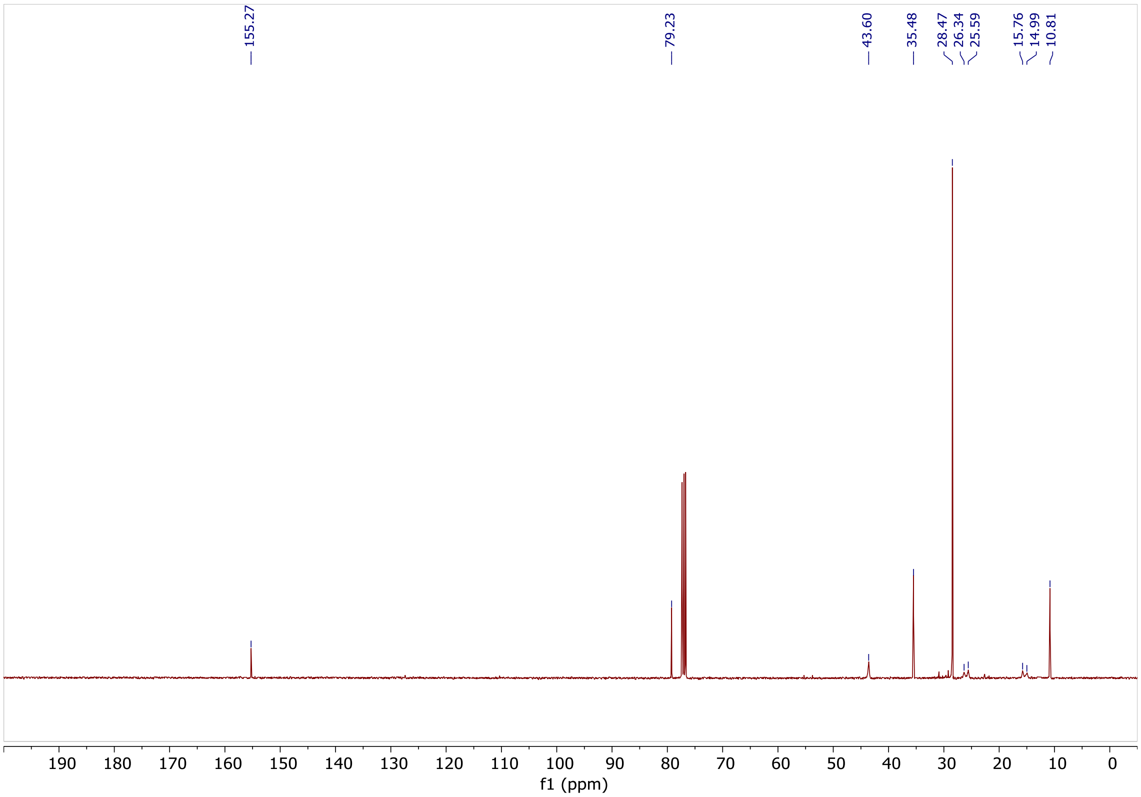

400 MHz ^{1}H NMR spectrum; 100.6 MHz ^{13}C NMR spectrum; $CDCl_3$ of **14**

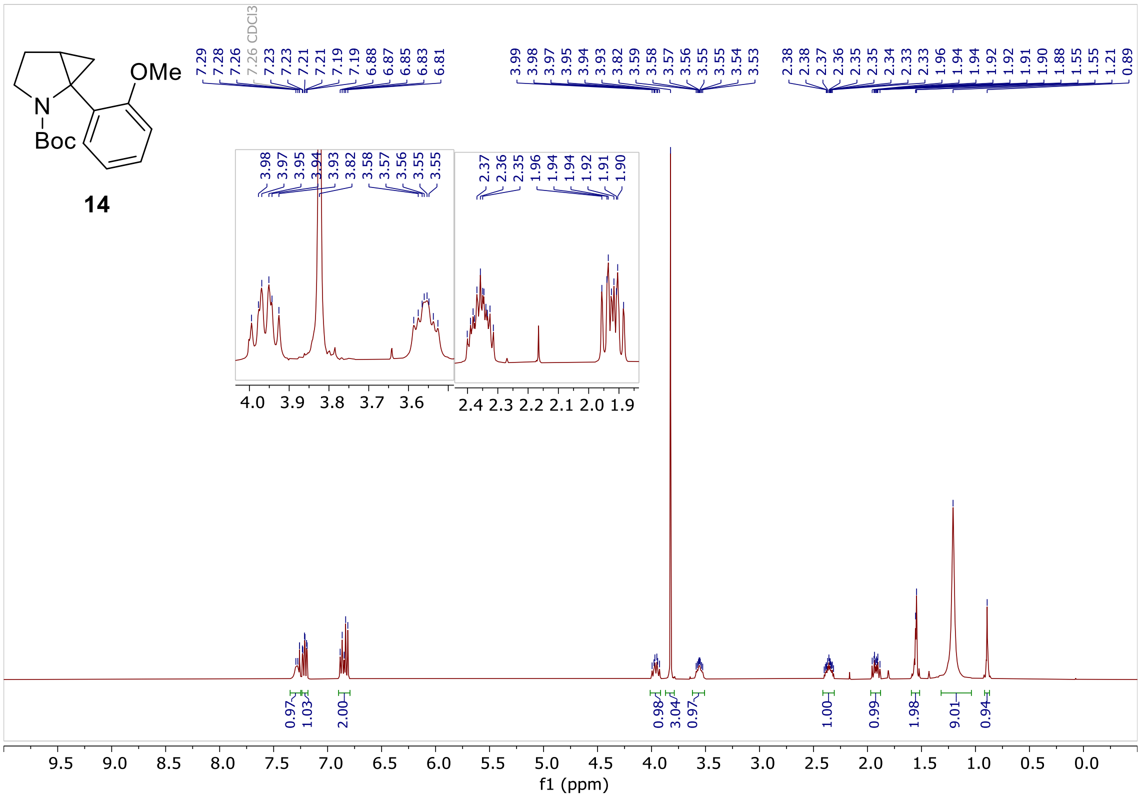

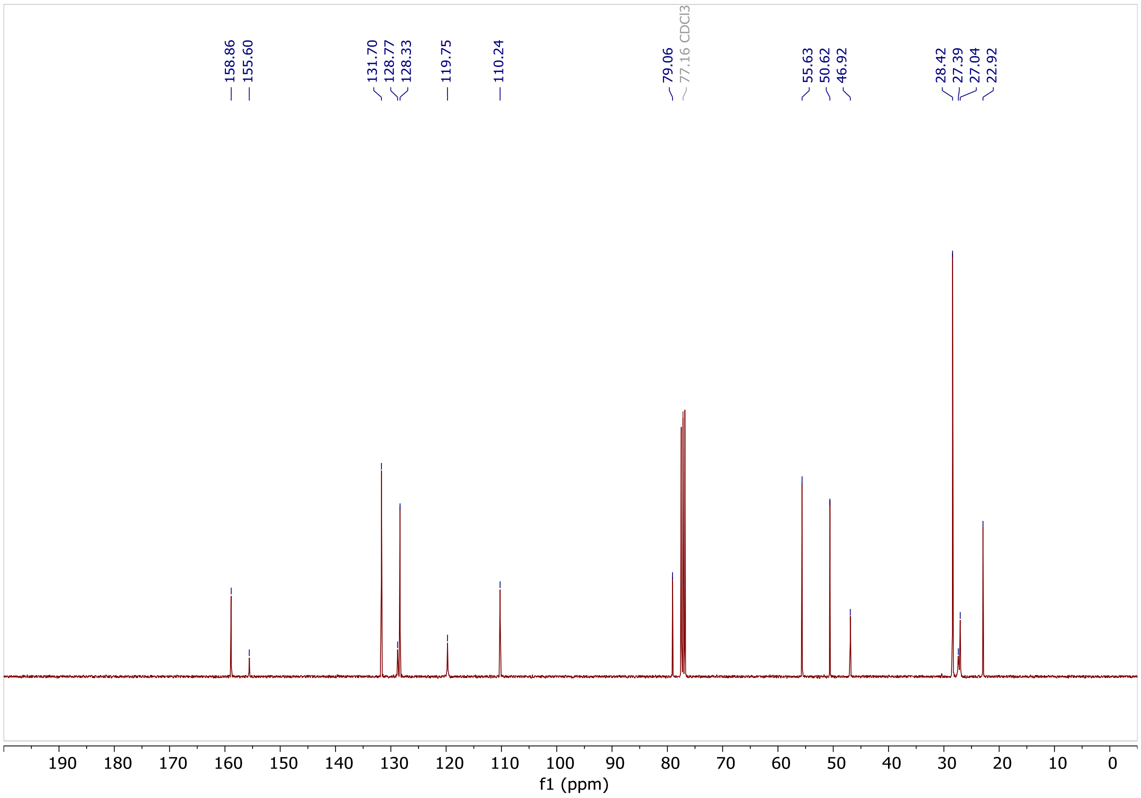

400 MHz ^{1}H NMR spectrum; 100.6 MHz ^{13}C NMR spectrum; $CDCl_3$ of **15**

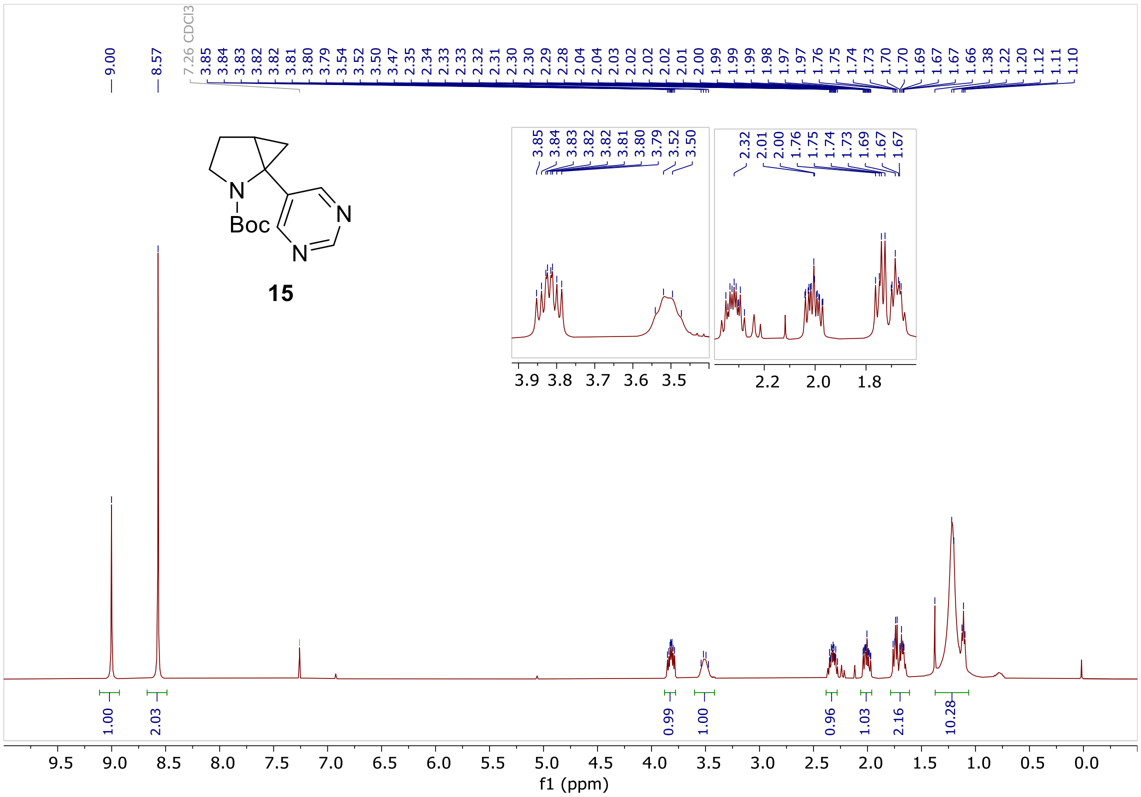

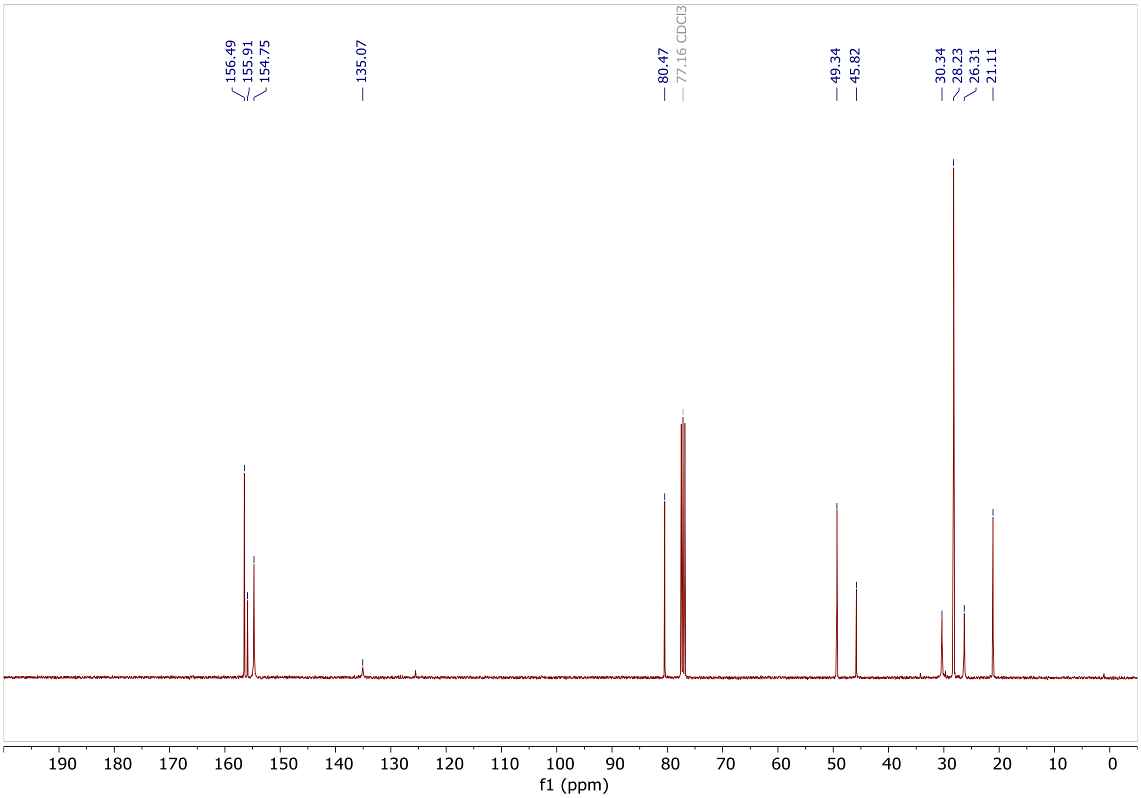

400 MHz ^{1}H NMR spectrum; 100.6 MHz ^{13}C NMR spectrum; $CDCl_3$ of **16**

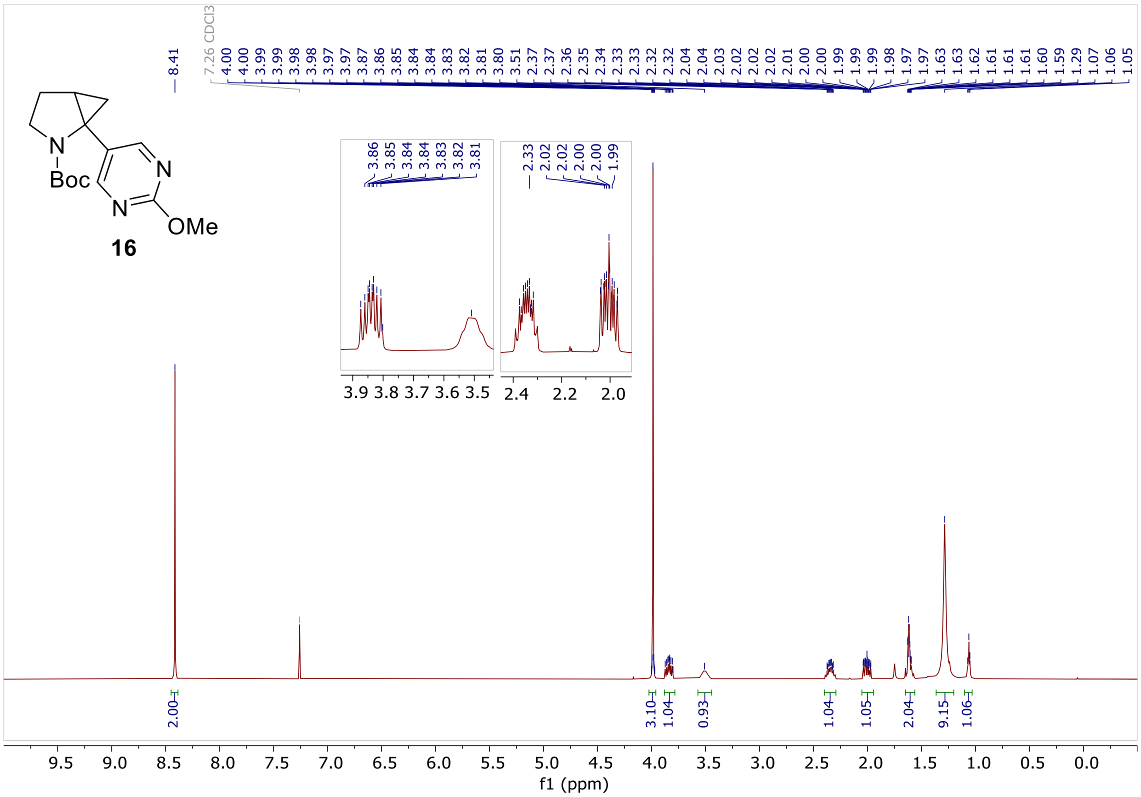

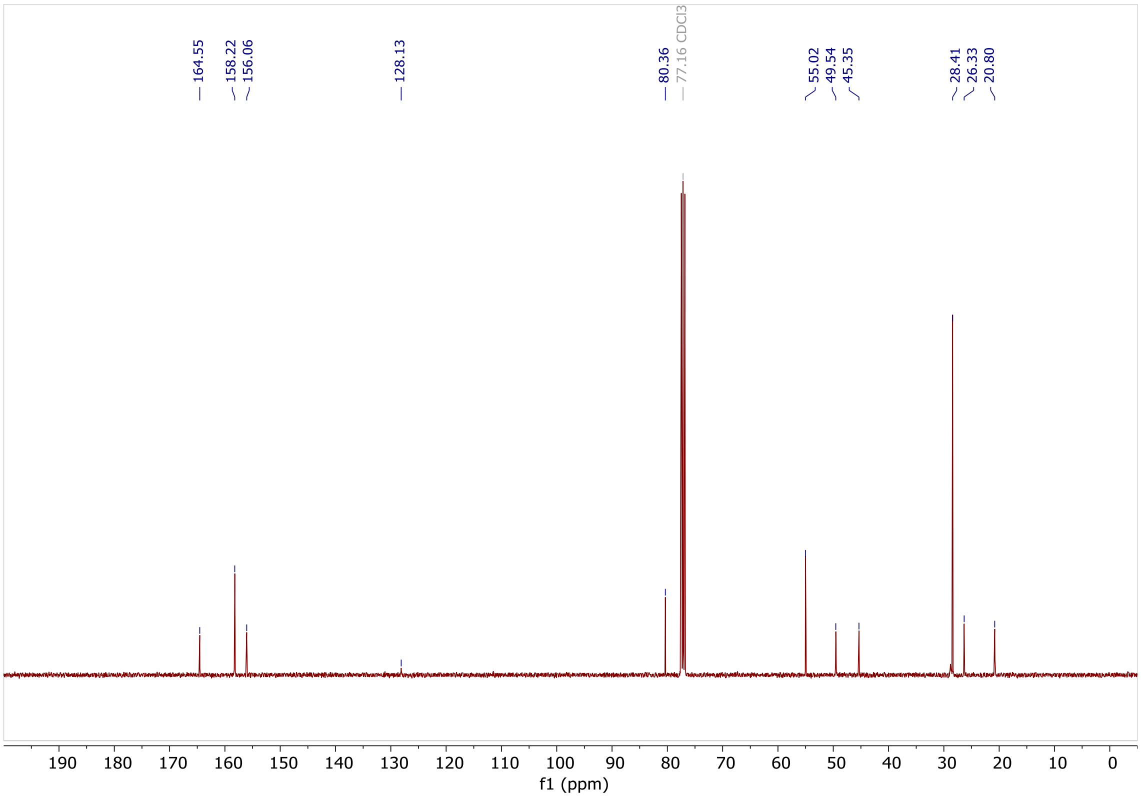

400 MHz ^{1}H NMR spectrum; 100.6 MHz ^{13}C NMR spectrum; $CDCl_3$ of **17**

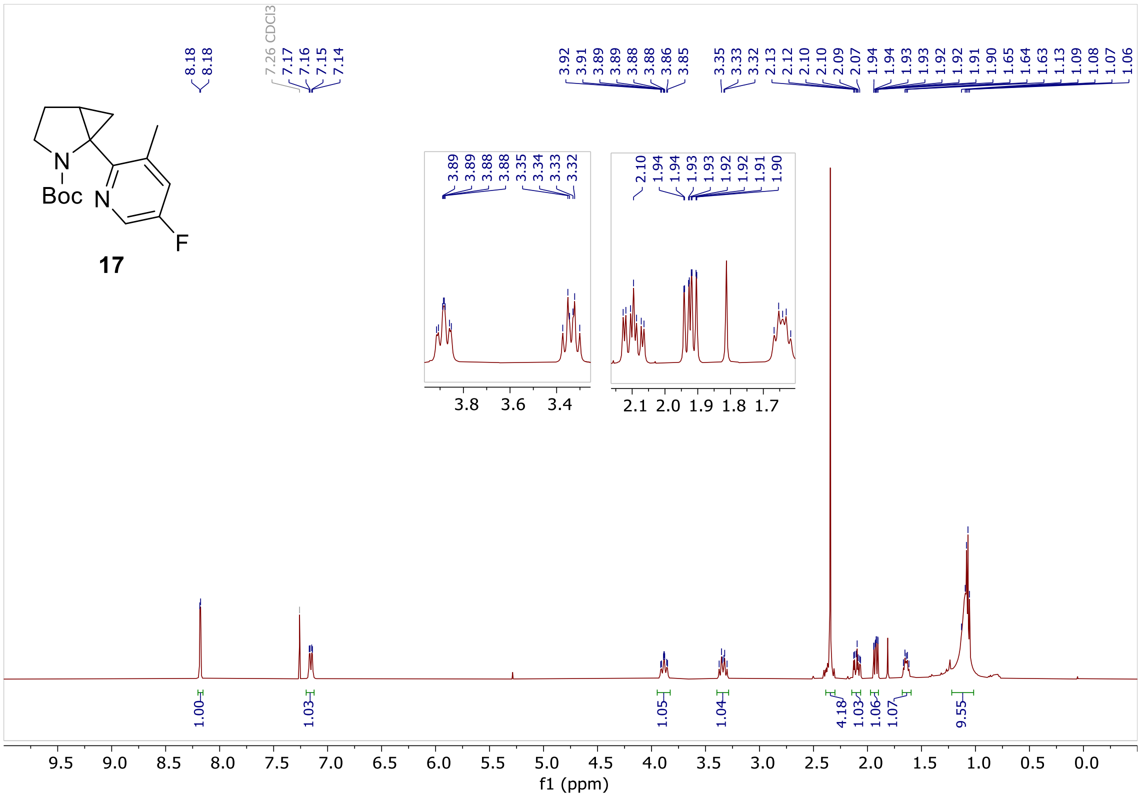

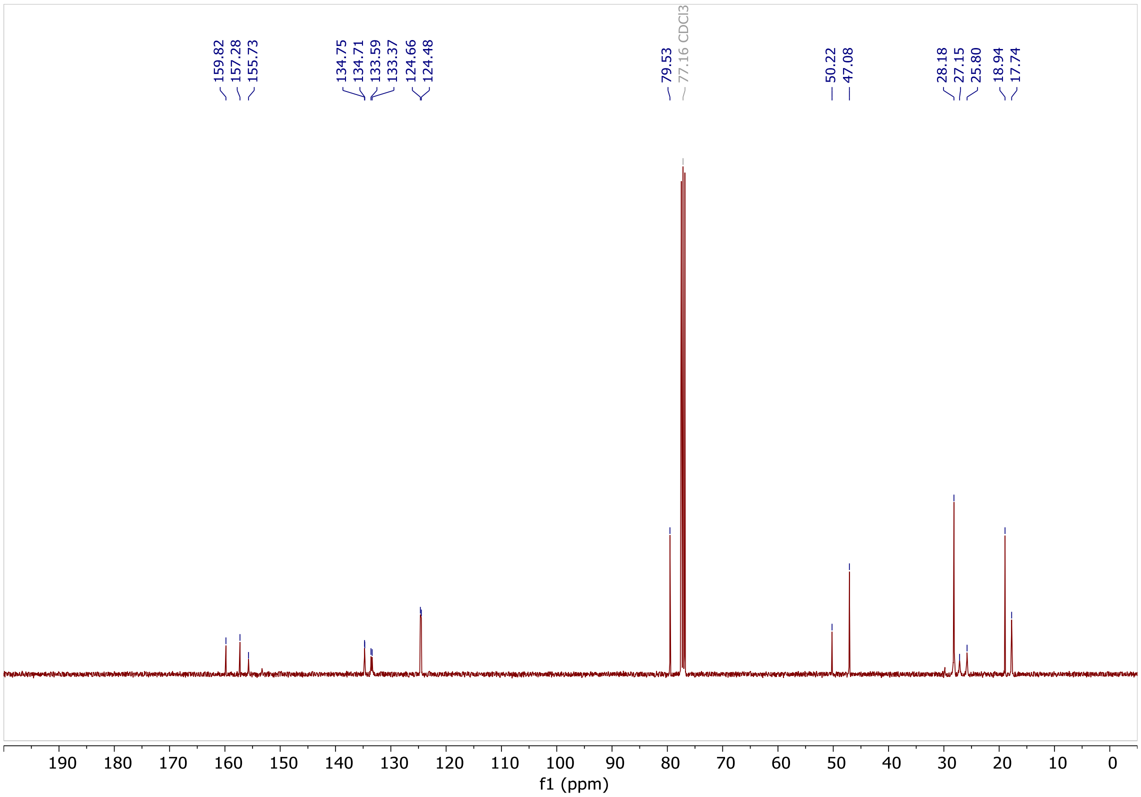

400 MHz ^1H NMR spectrum; 100.6 MHz ^{13}C NMR spectrum; $CDCl_3$ of **S29**

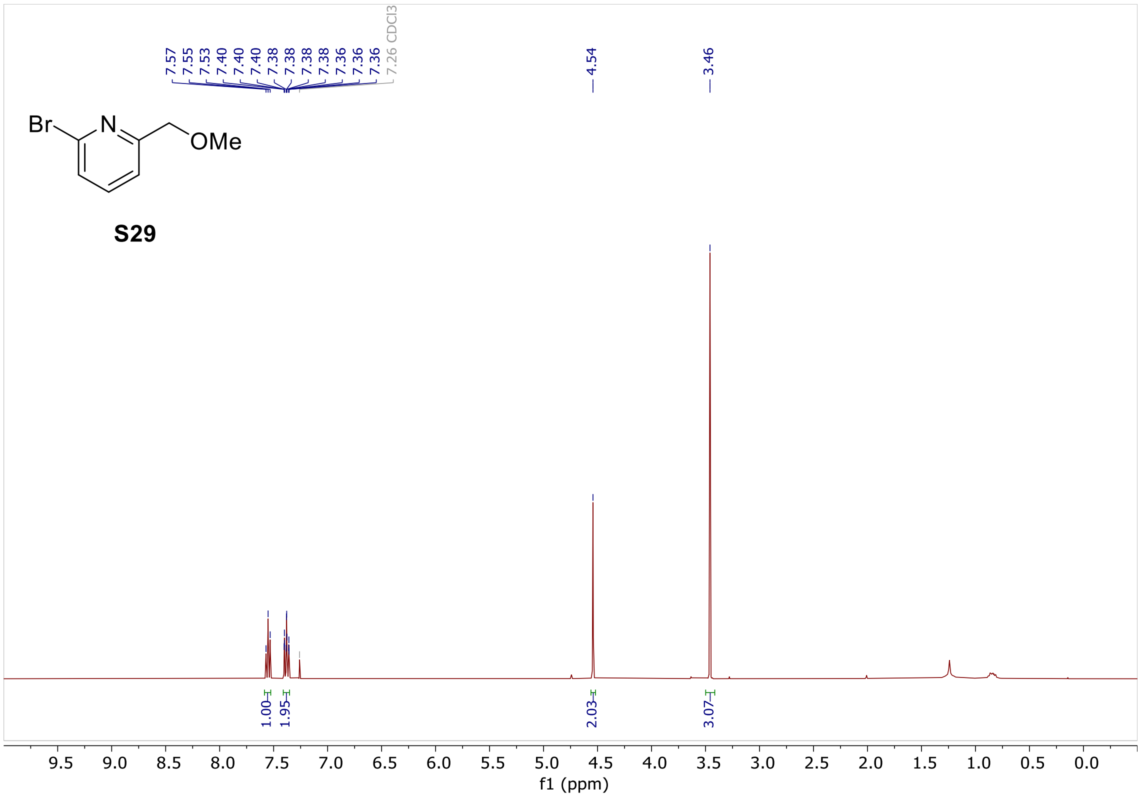

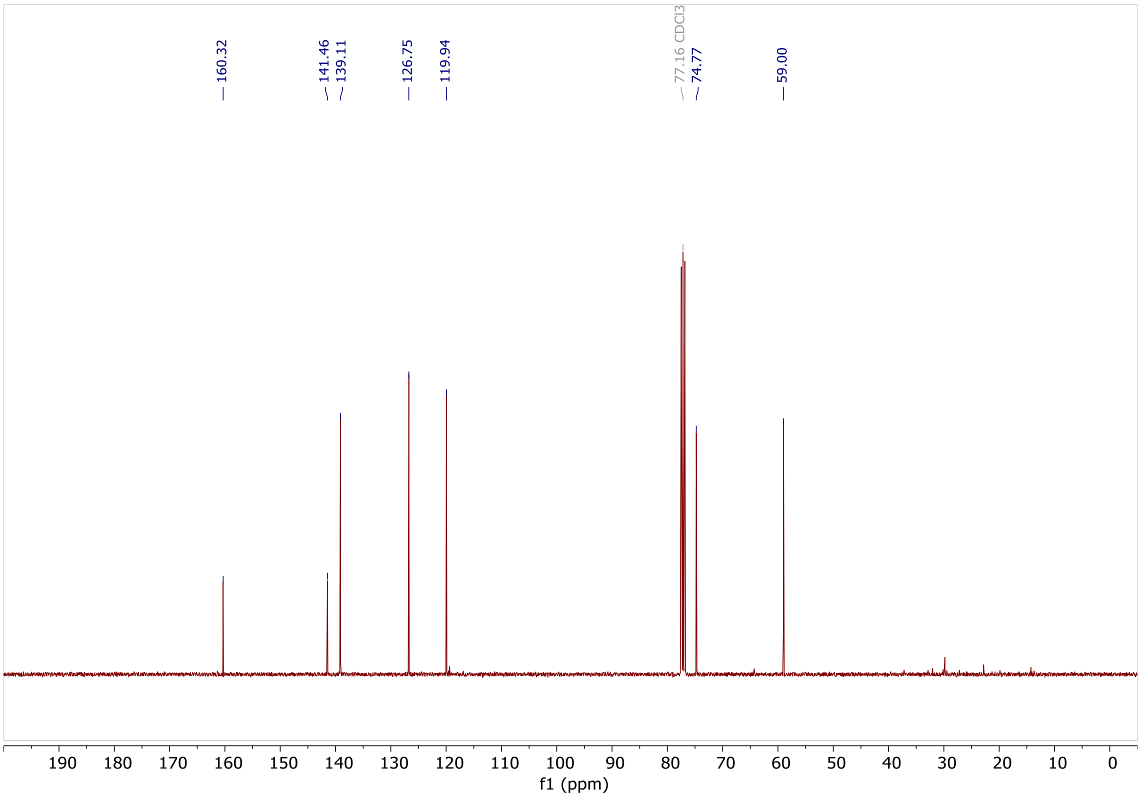

400 MHz ^{1}H NMR spectrum; 100.6 MHz ^{13}C NMR spectrum; $CDCl_3$ of **18**

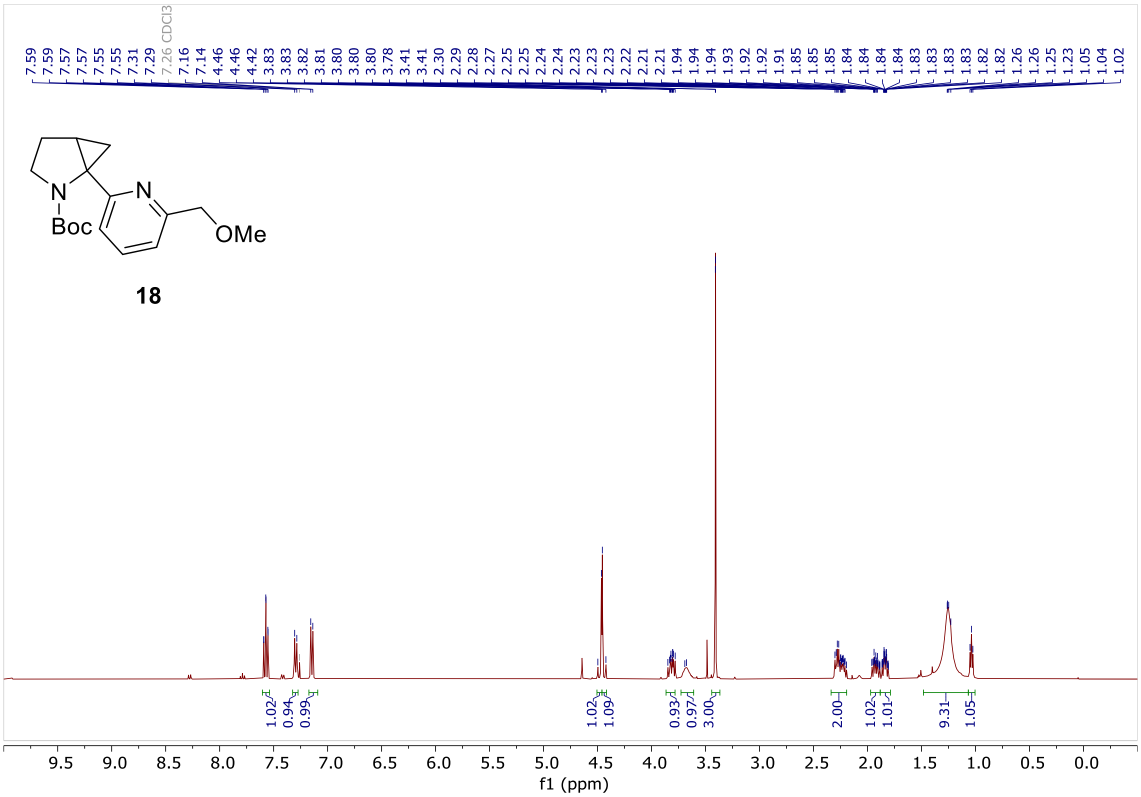

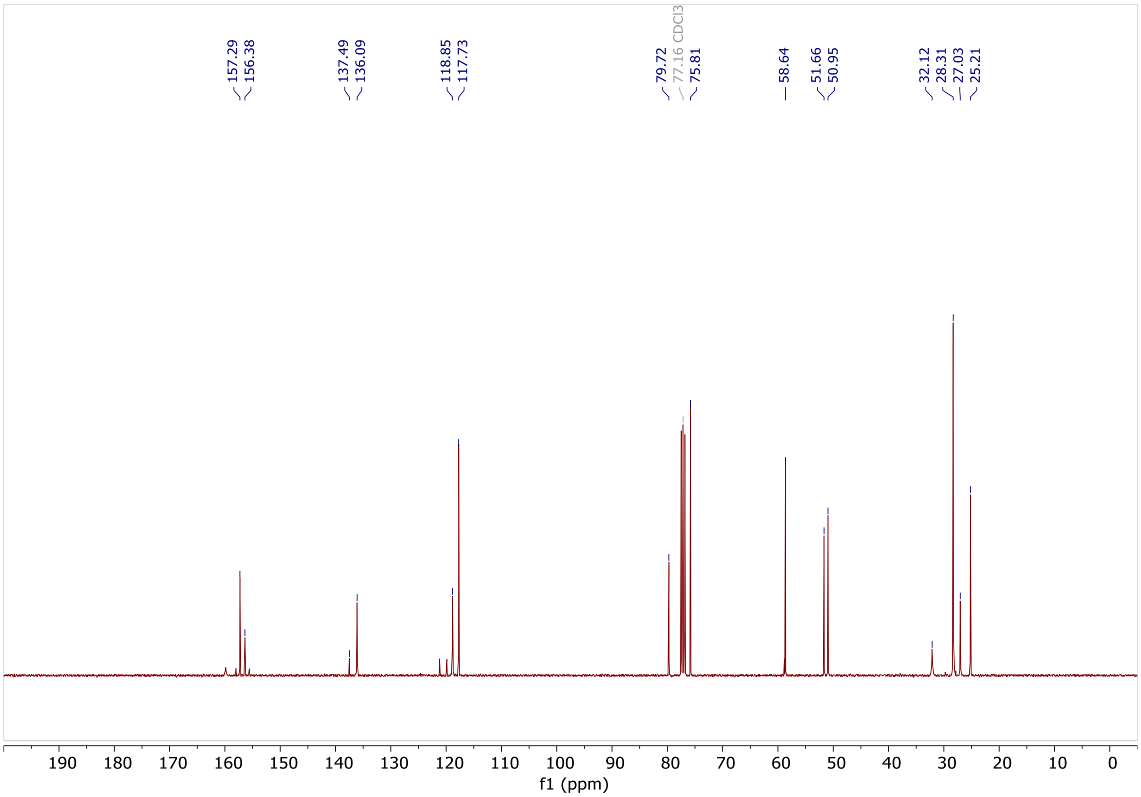

400 MHz ^{1}H NMR spectrum; 100.6 MHz ^{13}C NMR spectrum; $CDCl_3$ of **19**

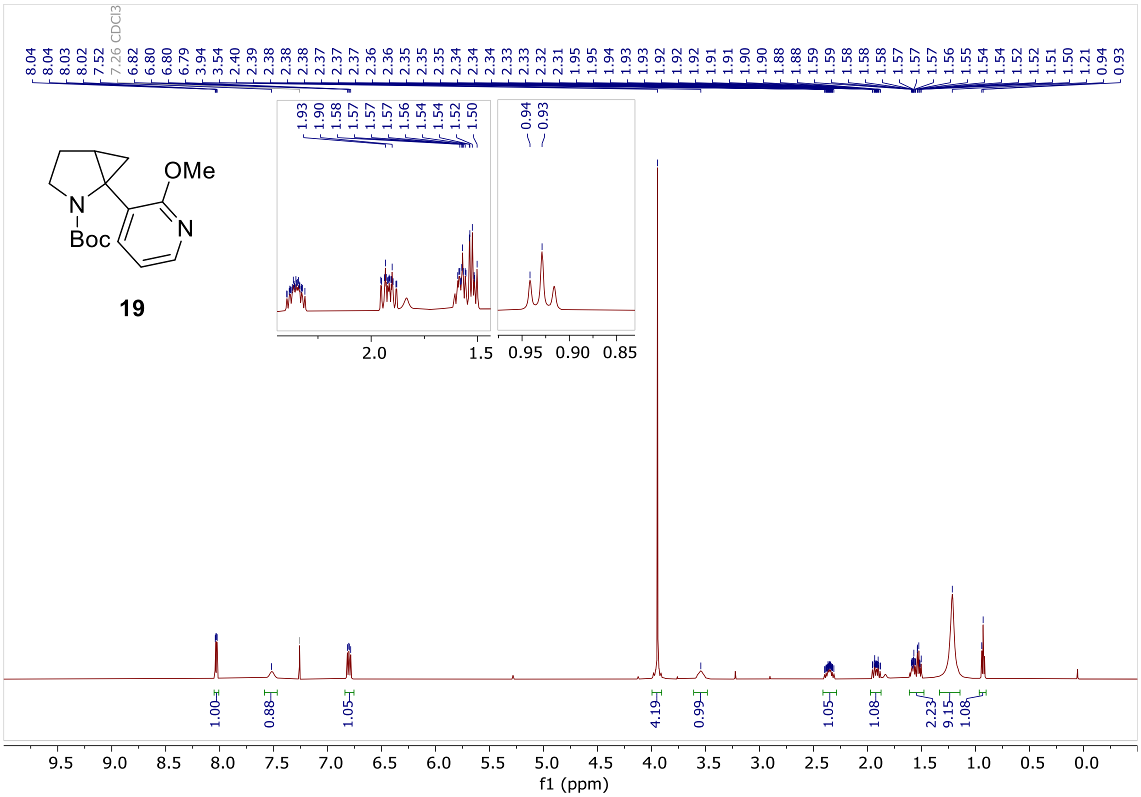

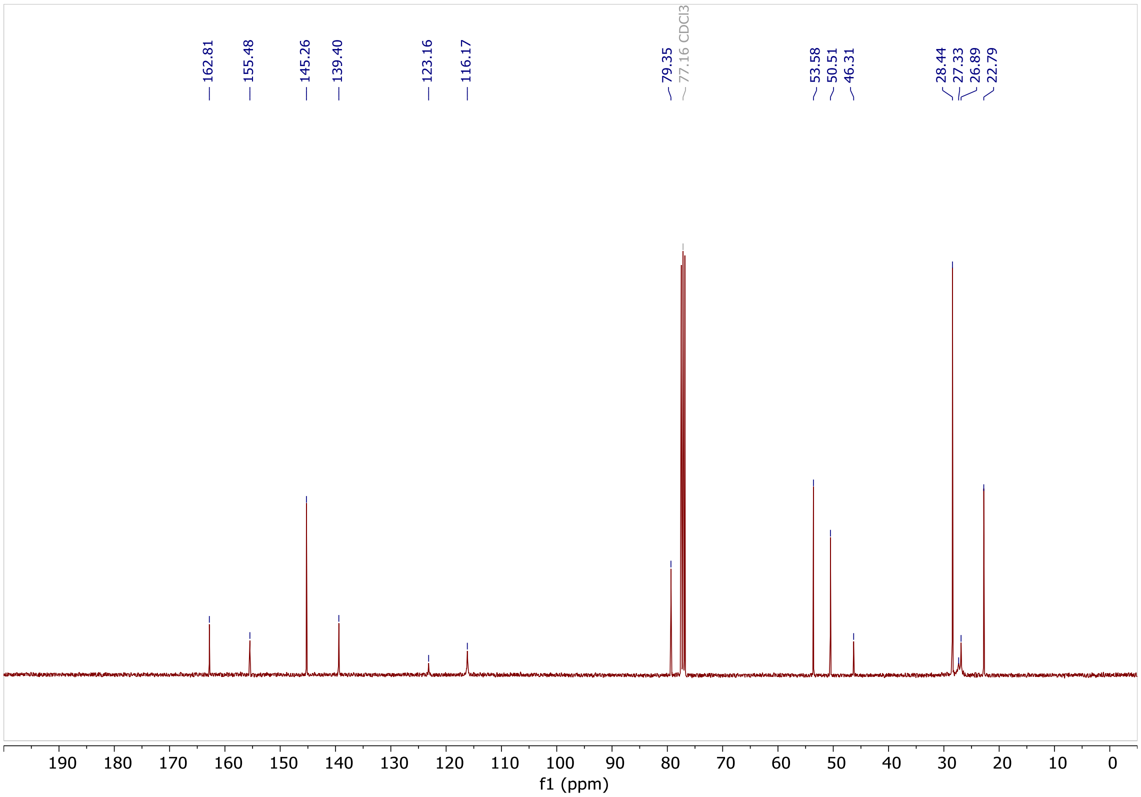

400 MHz ^{1}H NMR spectrum; 100.6 MHz ^{13}C NMR spectrum; $CDCl_3$ of **S30**

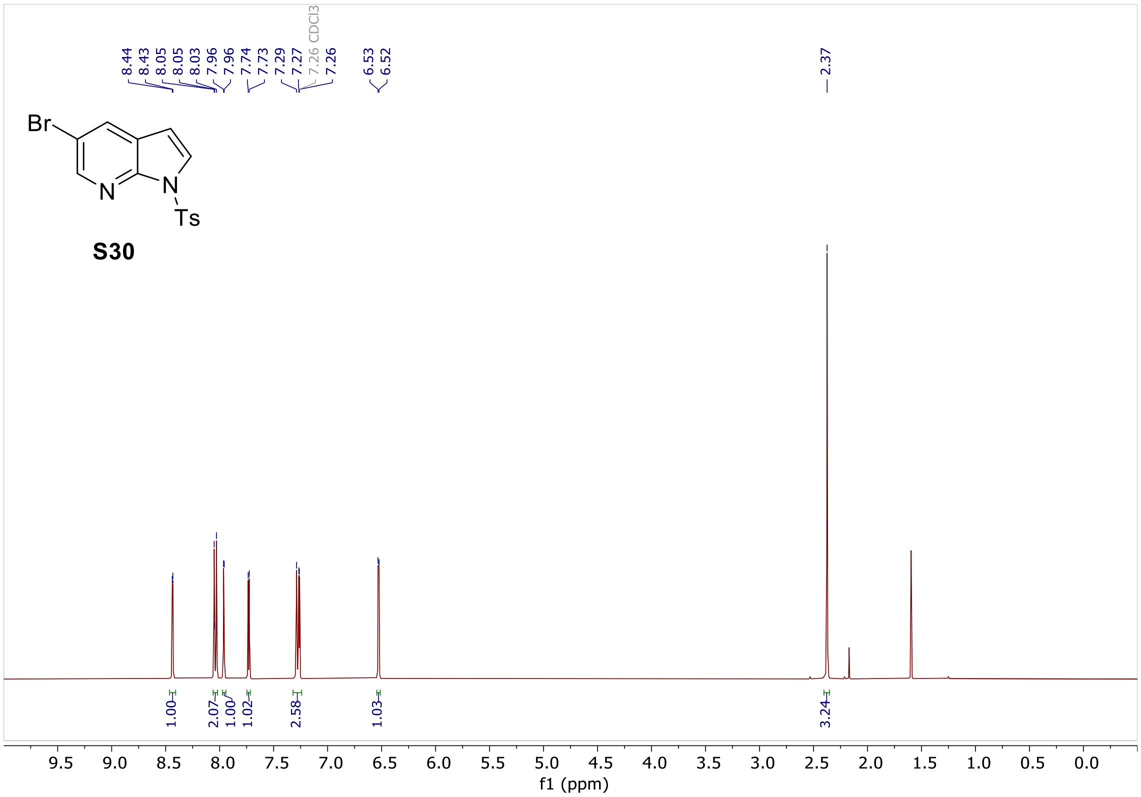

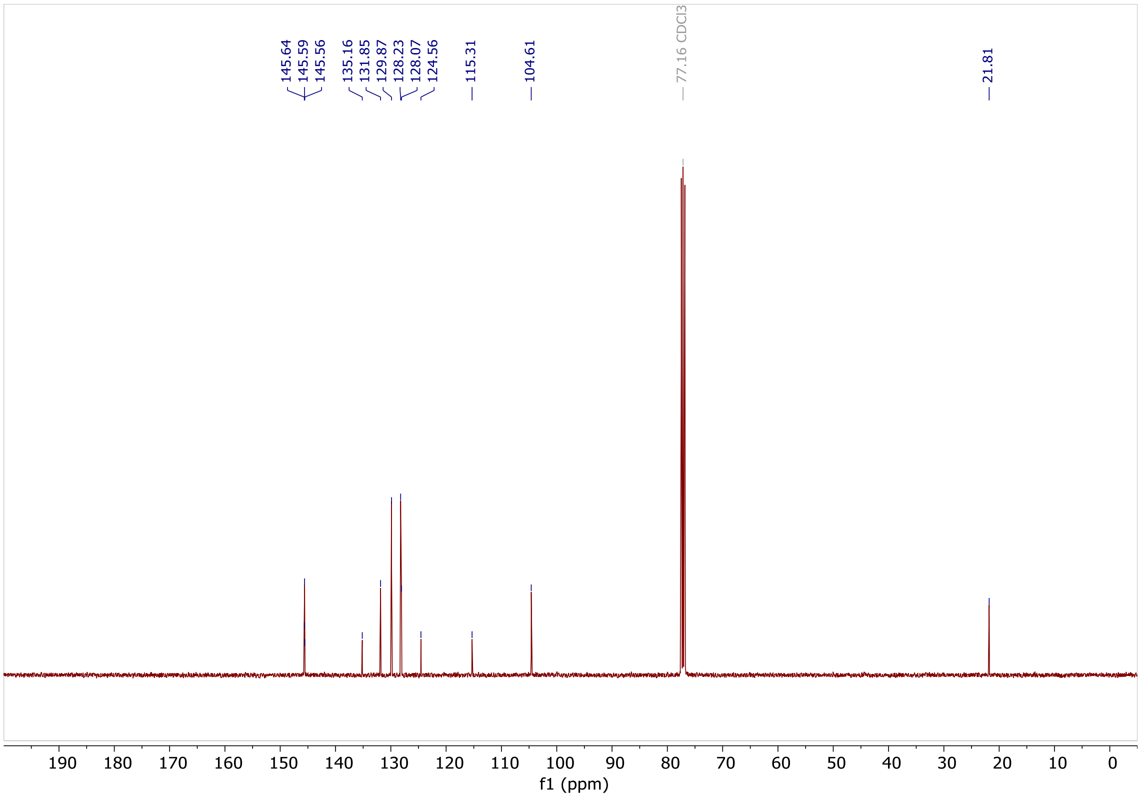

400 MHz ^{1}H NMR spectrum; 100.6 MHz ^{13}C NMR spectrum; $CDCl_3$ of **20**

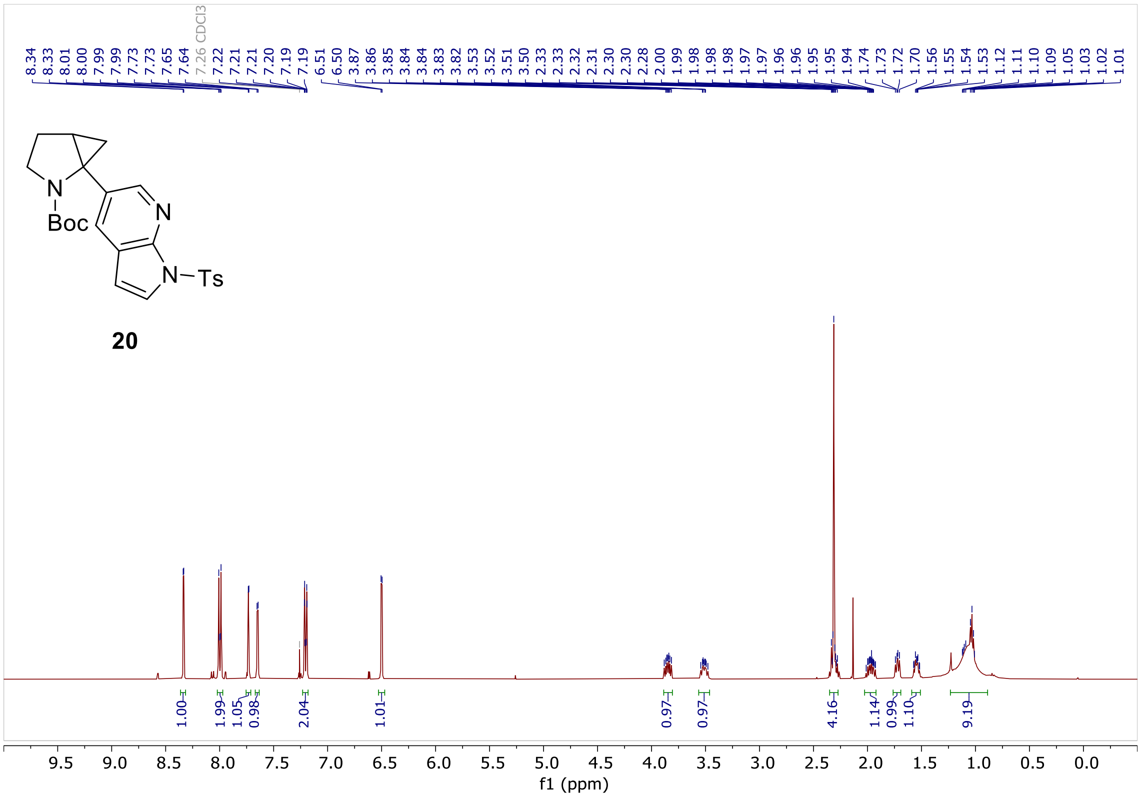

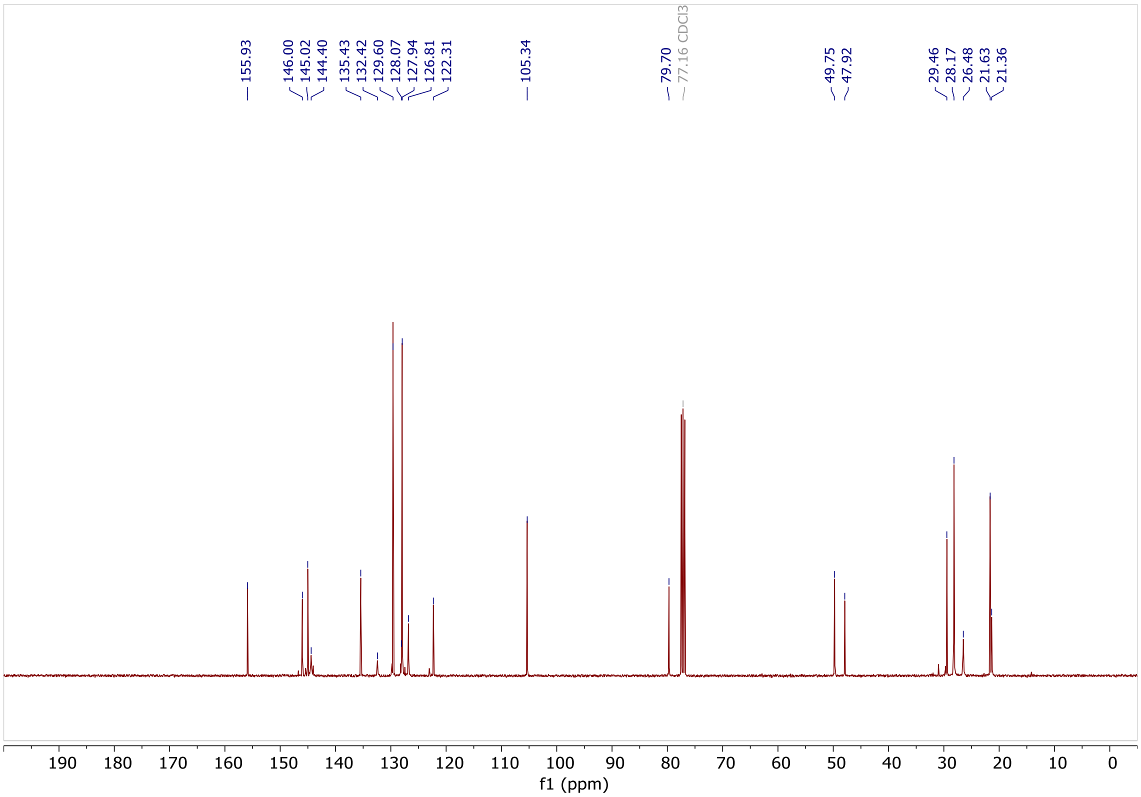

400 MHz ^{1}H NMR spectrum; 100.6 MHz ^{13}C NMR spectrum; $CDCl_3$ of **S31**

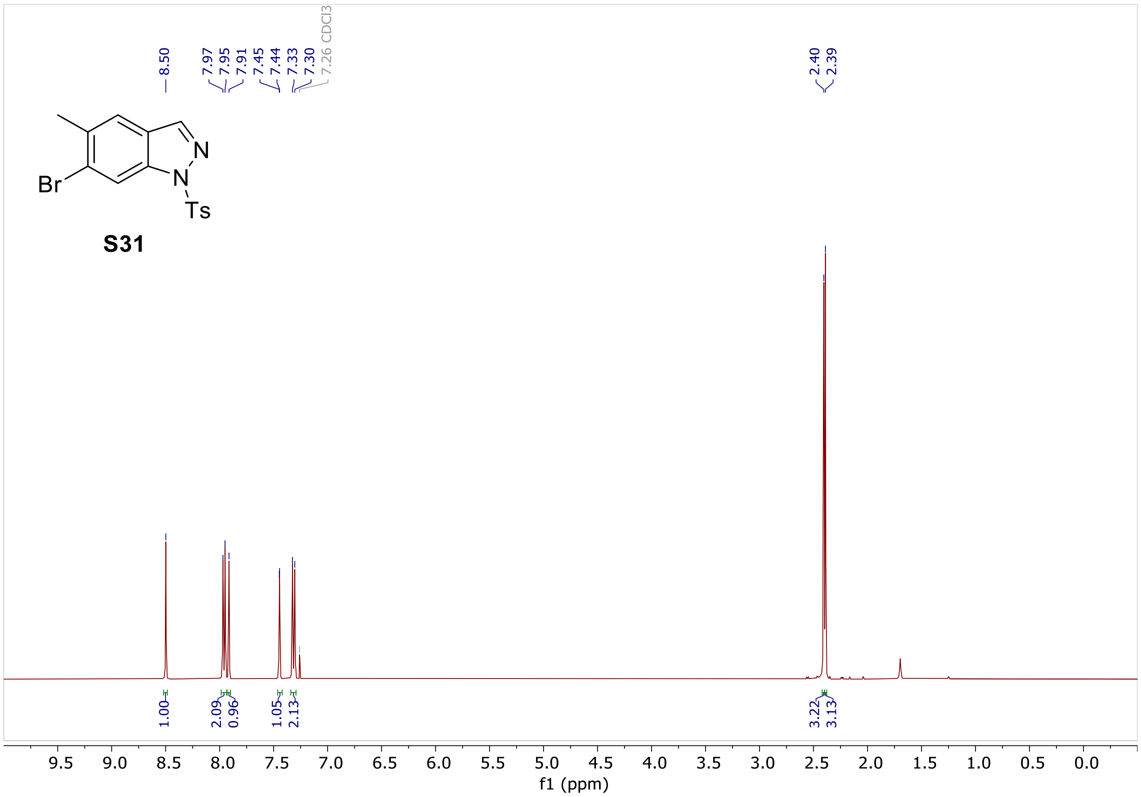

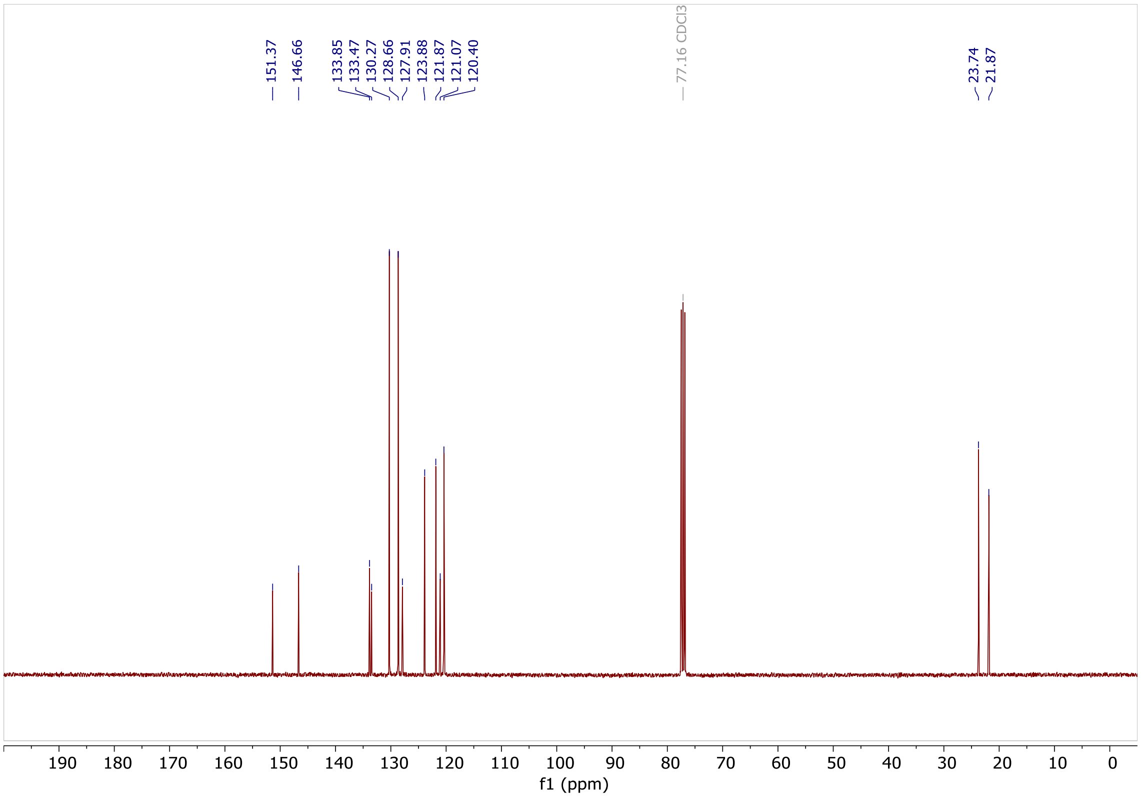

400 MHz ^{1}H NMR spectrum; 100.6 MHz ^{13}C NMR spectrum; $CDCl_3$ of **21**

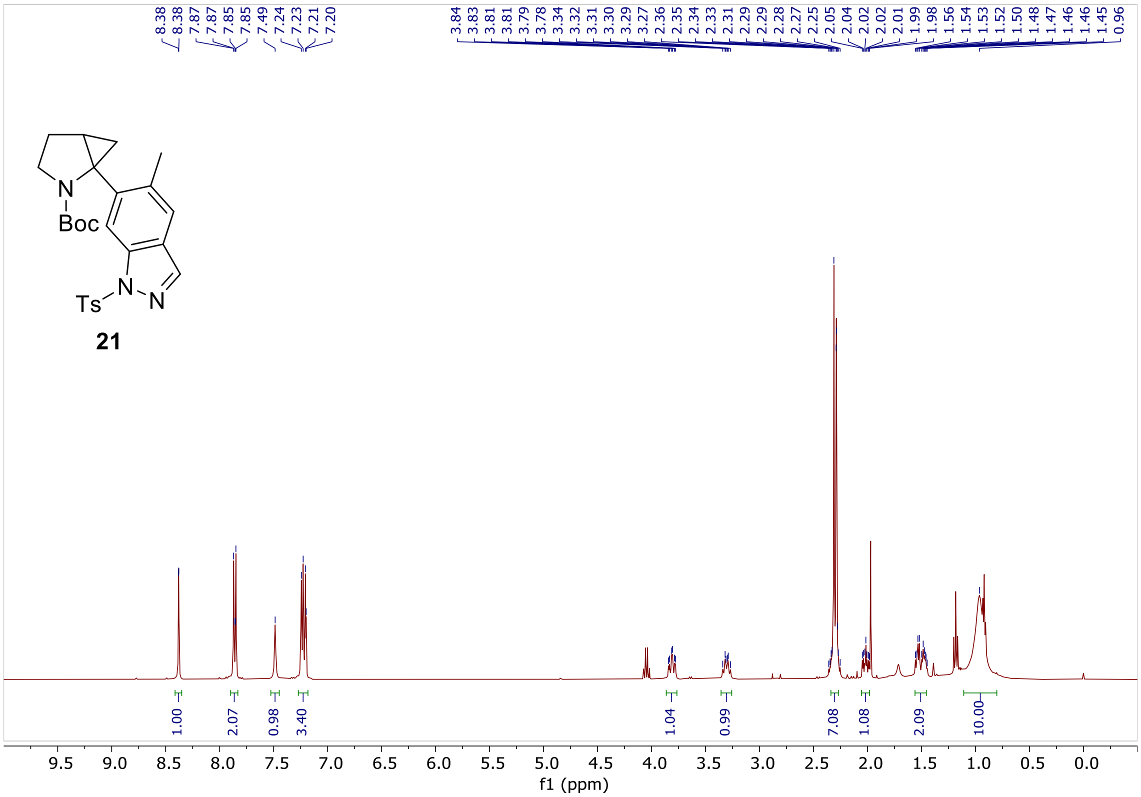

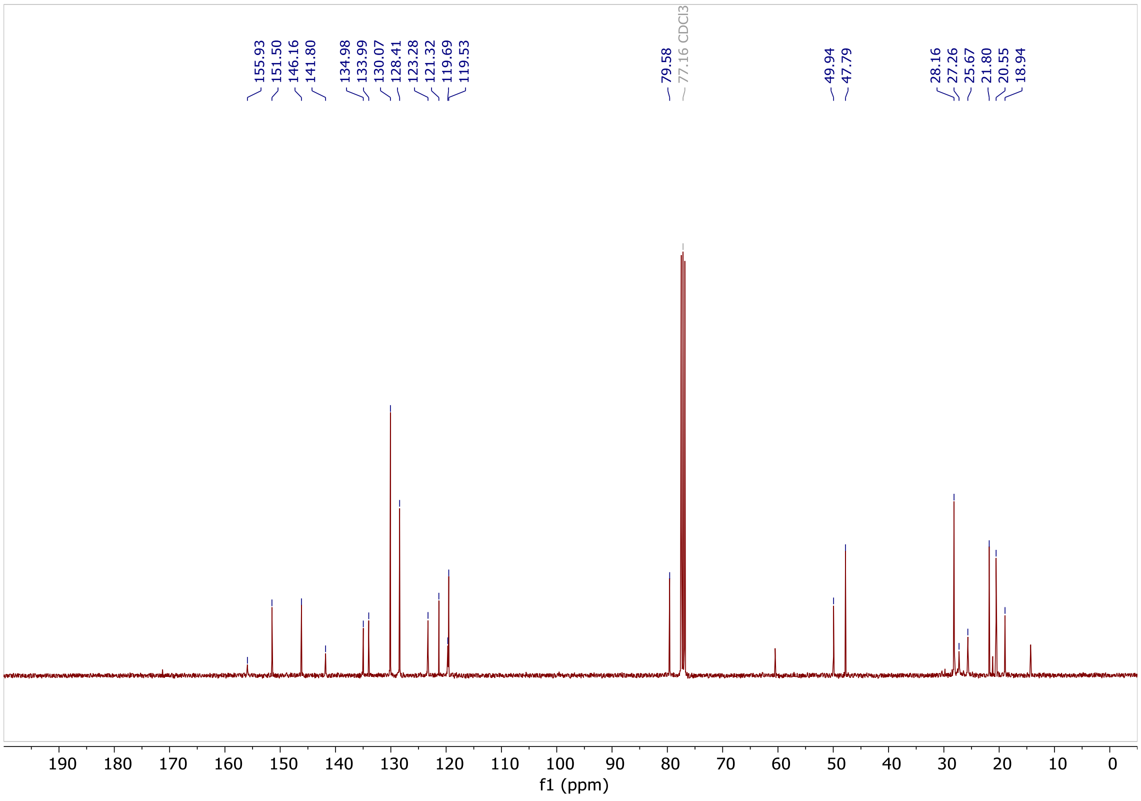

400 MHz ^{1}H NMR spectrum; 100.6 MHz ^{13}C NMR spectrum; $CDCl_3$ of (*S*,*R*)-**S32**

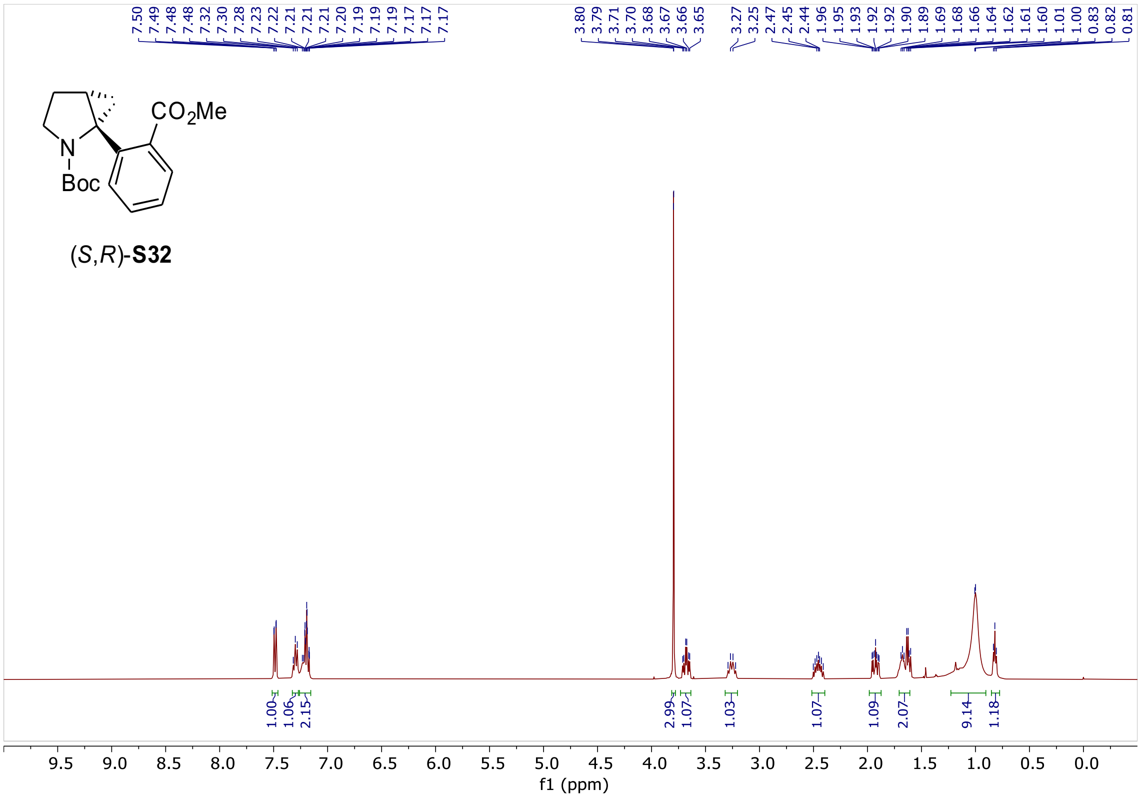

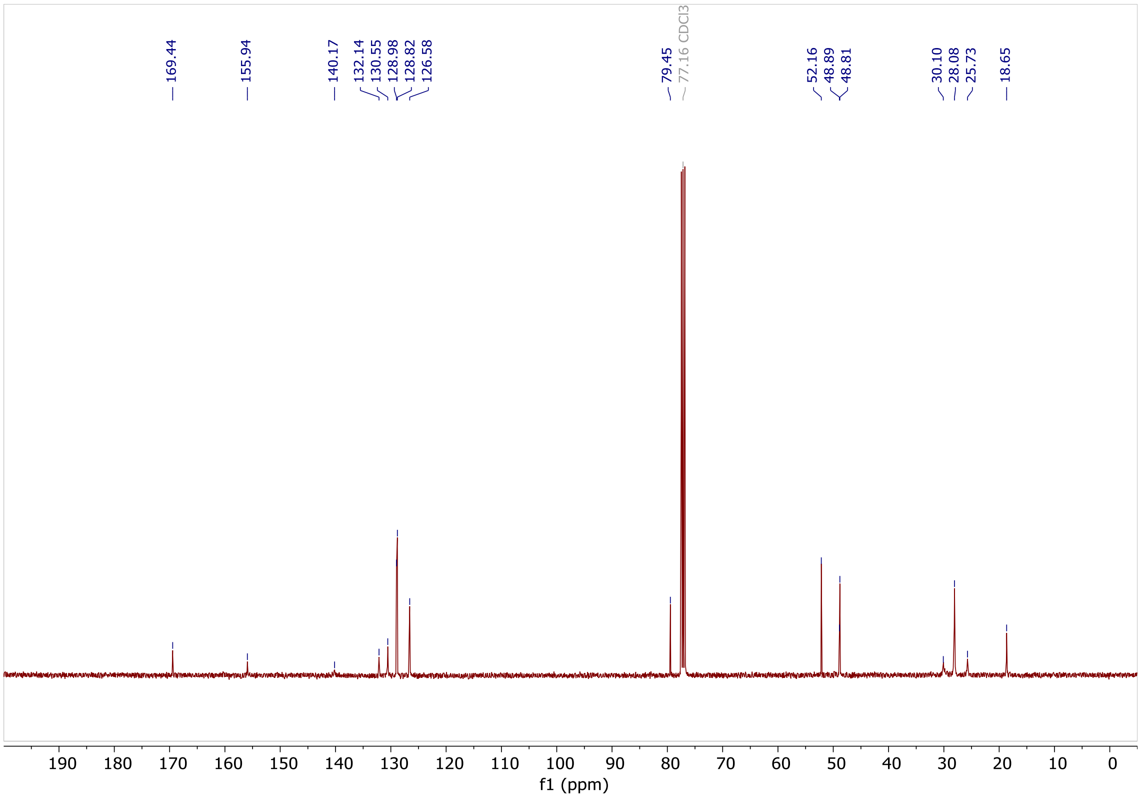

400 MHz ^{1}H NMR spectrum; 100.6 MHz ^{13}C NMR spectrum; $CDCl_3$ of **22**

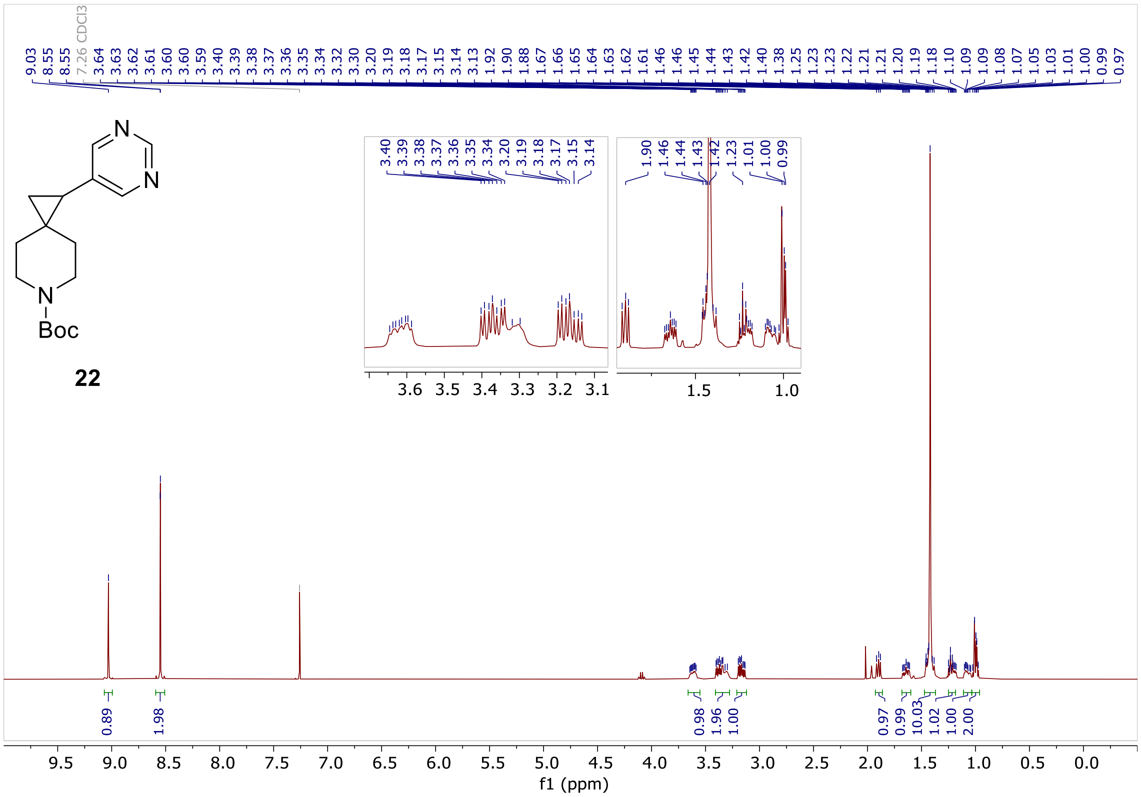

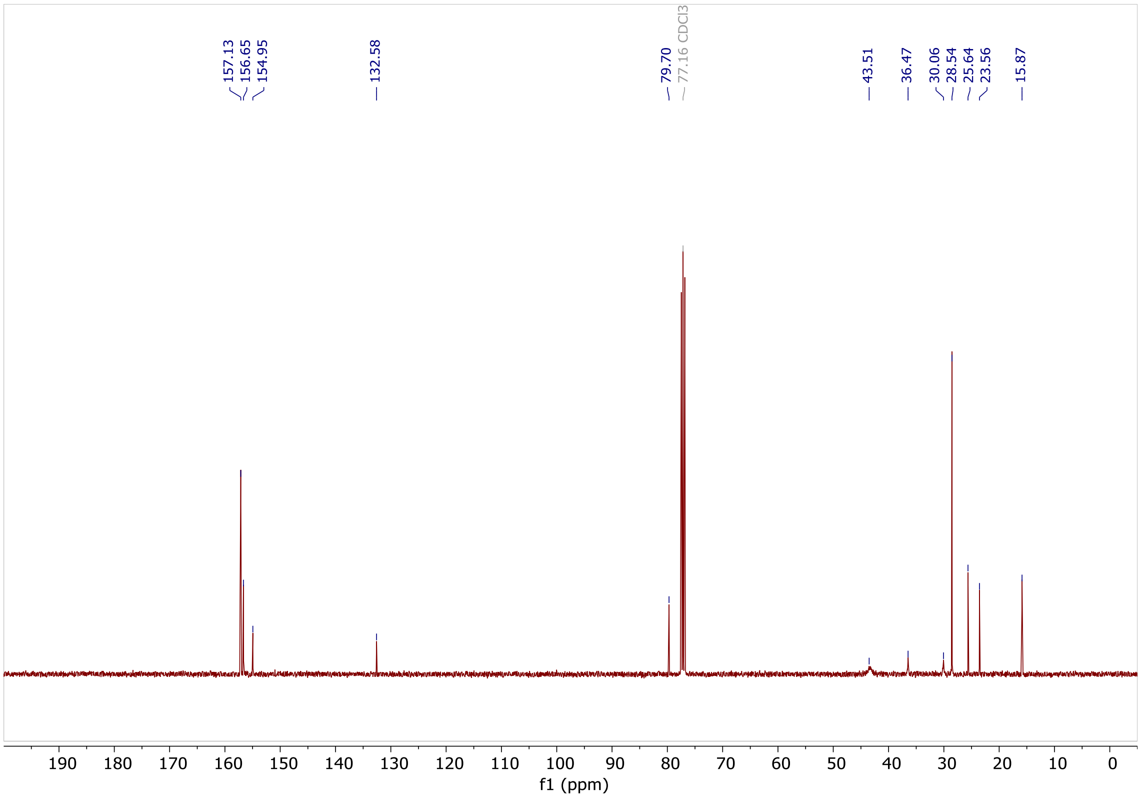

400 MHz ^{1}H NMR spectrum; 100.6 MHz ^{13}C NMR spectrum; $CDCl_3$ of **23**

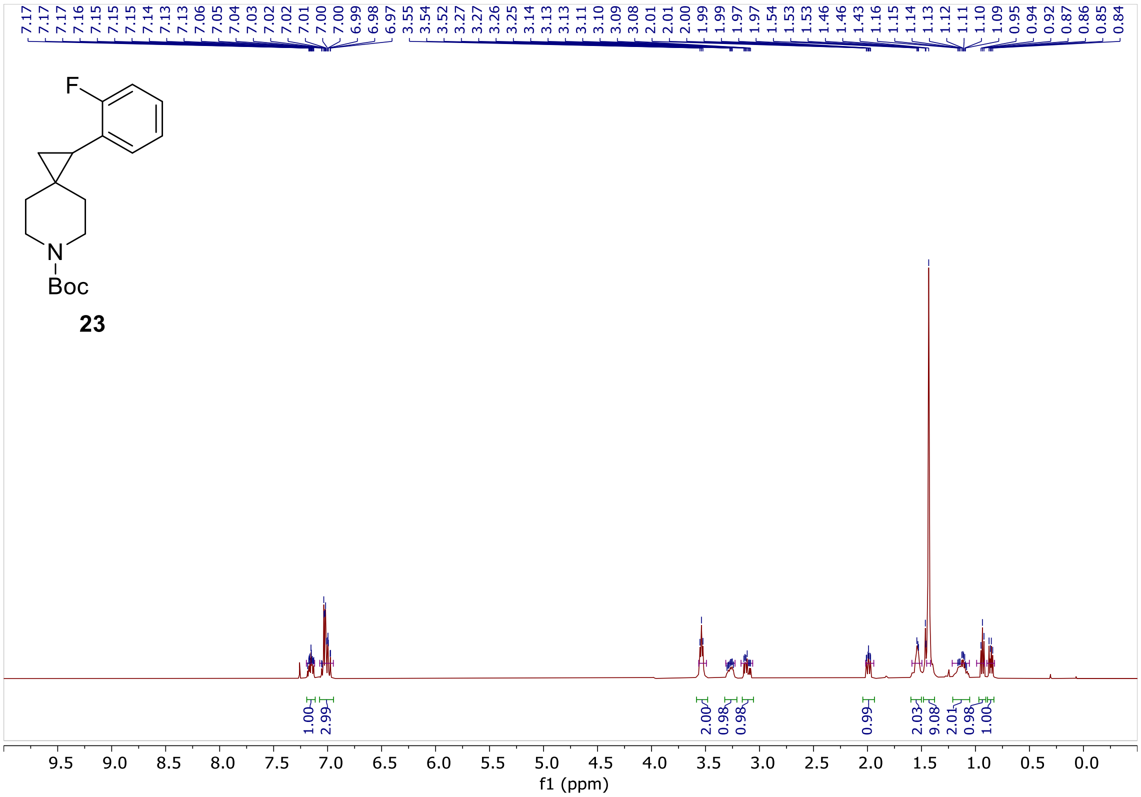

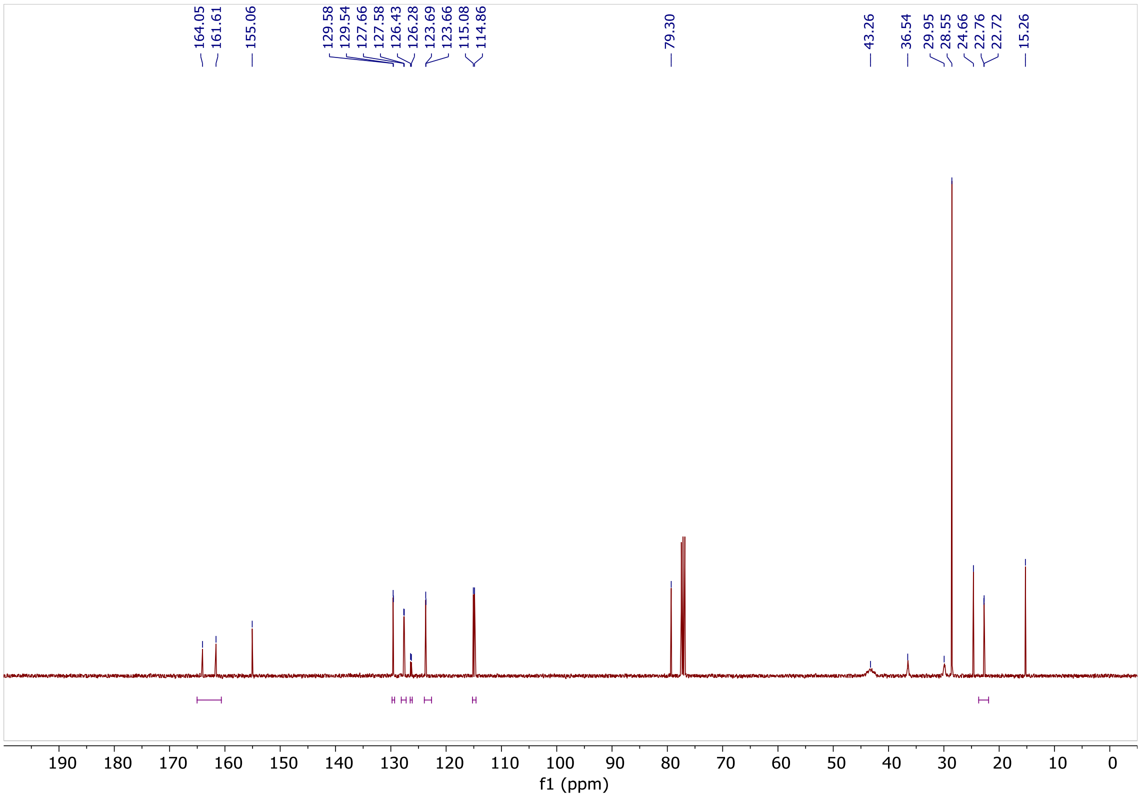

400 MHz ^{1}H NMR spectrum; 100.6 MHz ^{13}C NMR spectrum; $CDCl_3$ of **24**

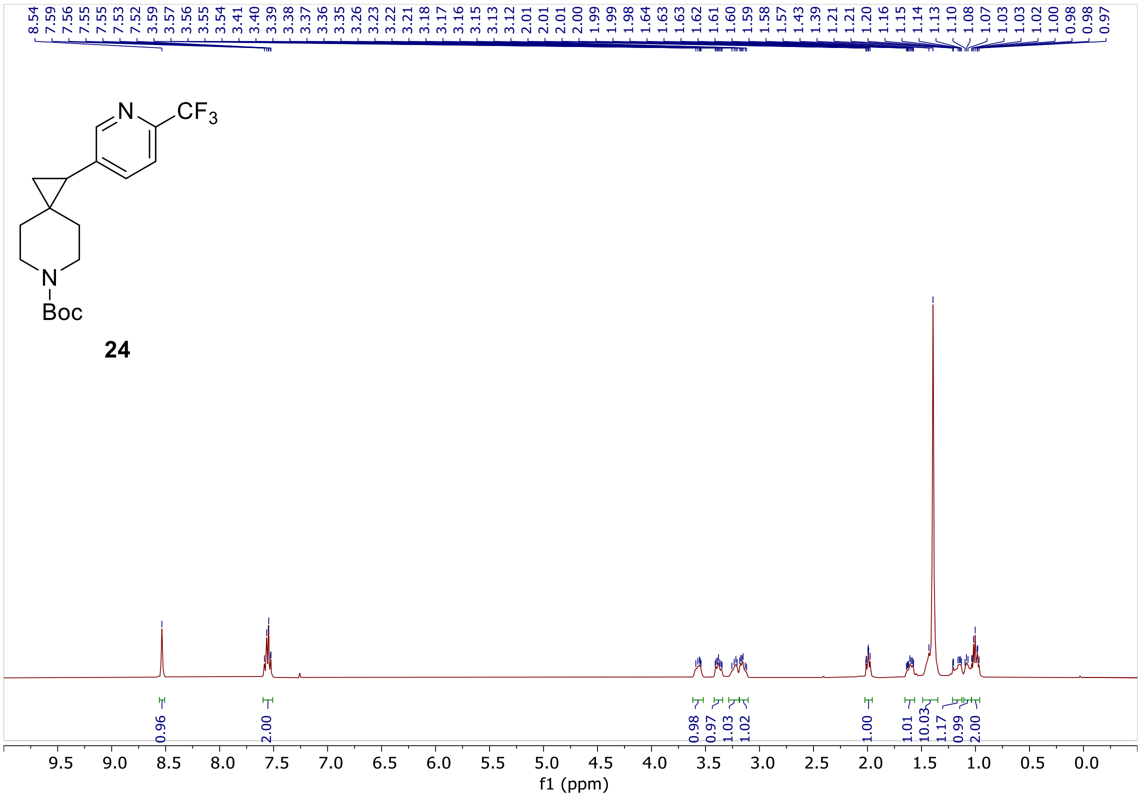

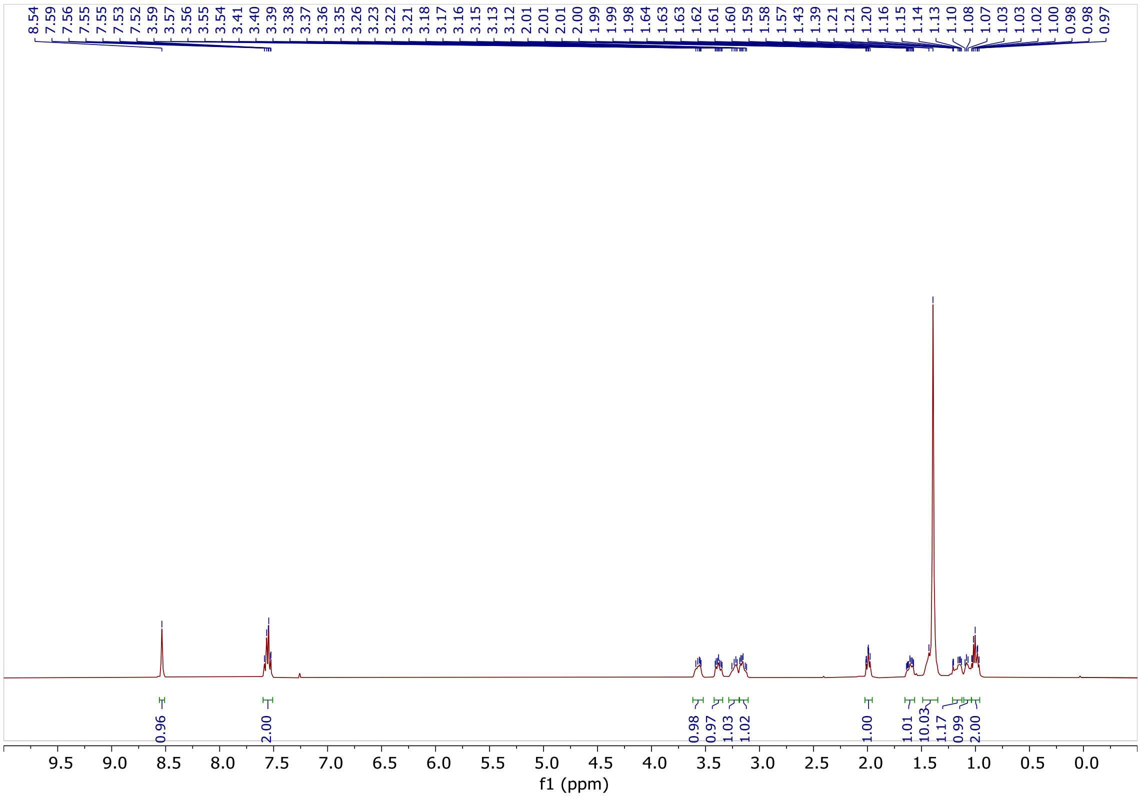

400 MHz ^{1}H NMR spectrum; 100.6 MHz ^{13}C NMR spectrum; $CDCl_3$ of **25**

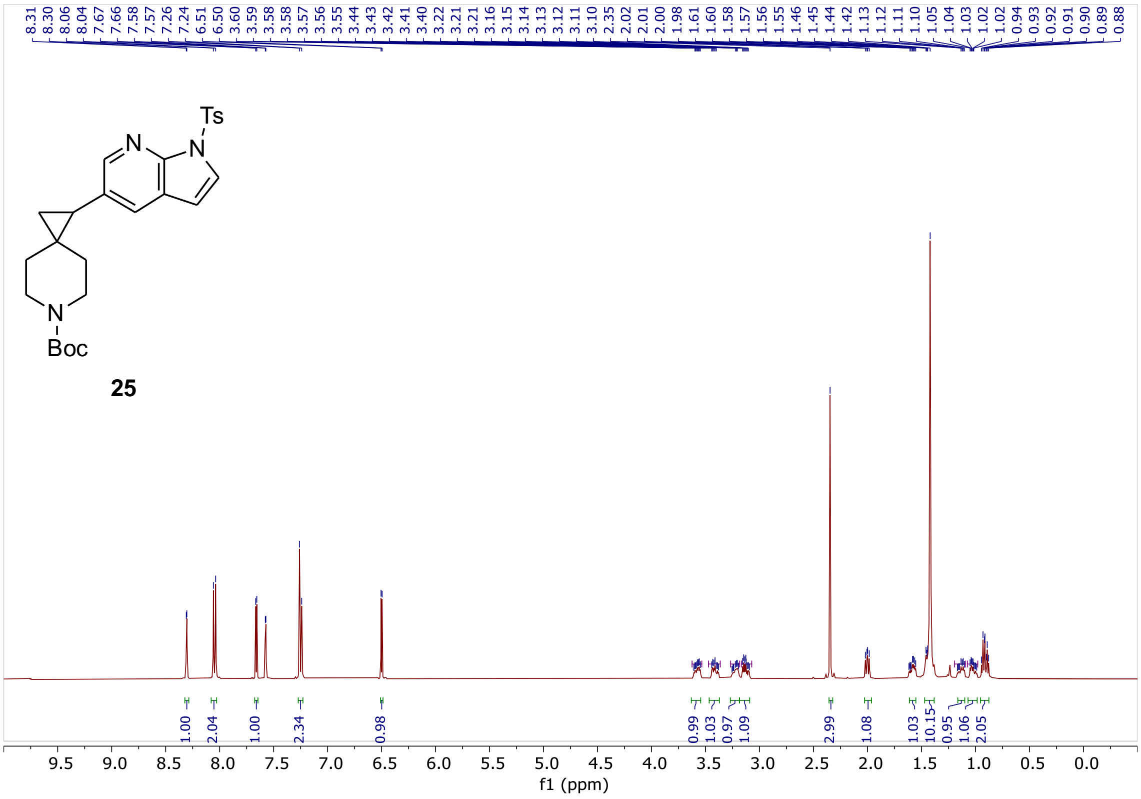

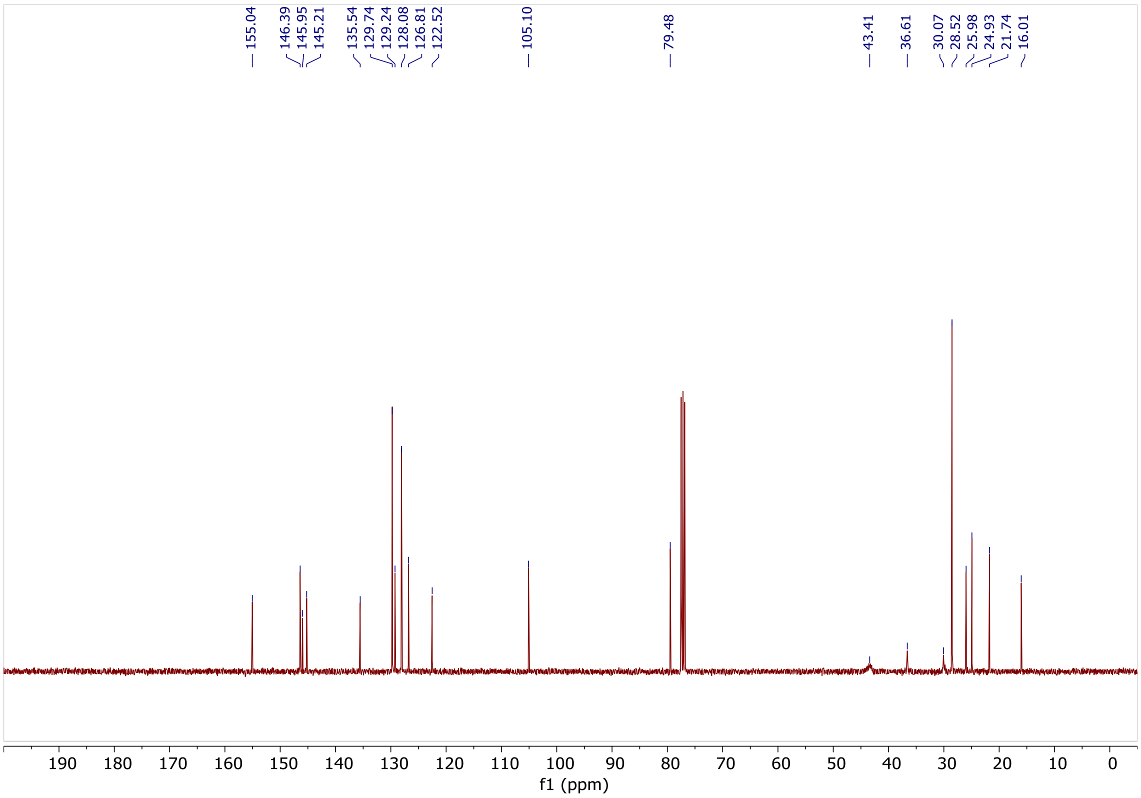

400 MHz ^{1}H NMR spectrum; 100.6 MHz ^{13}C NMR spectrum; $CDCl_3$ of **26**

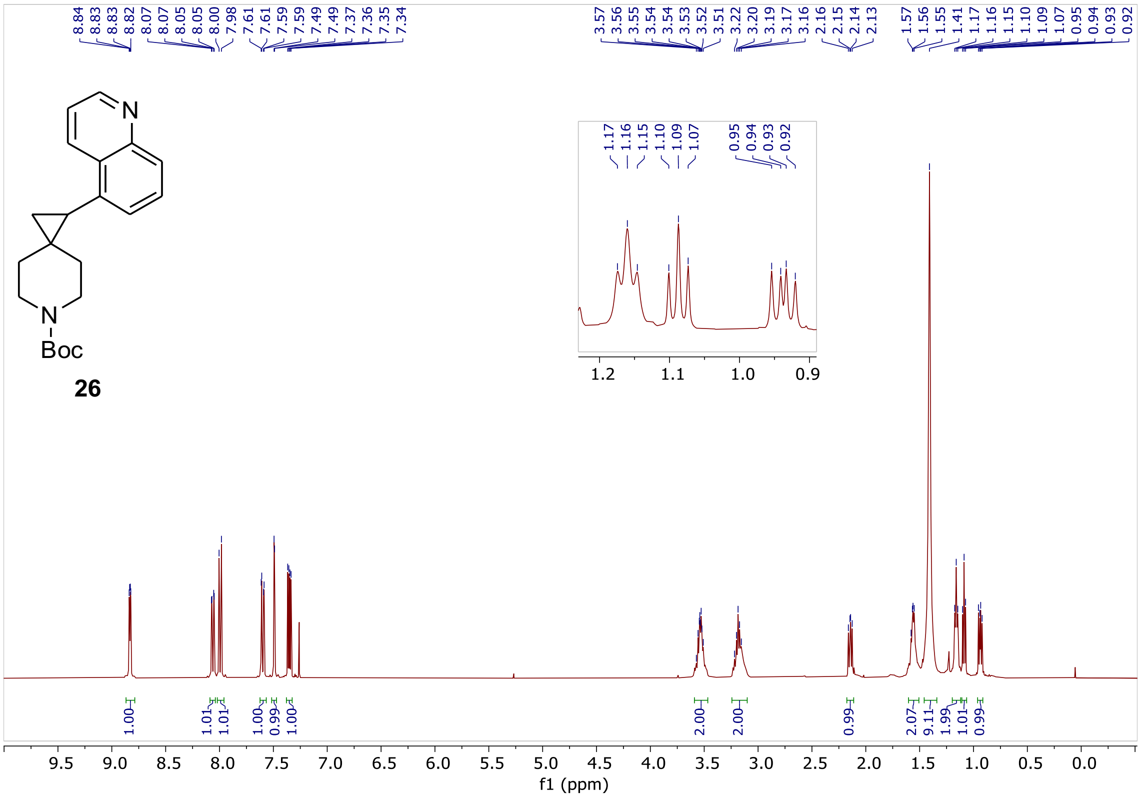

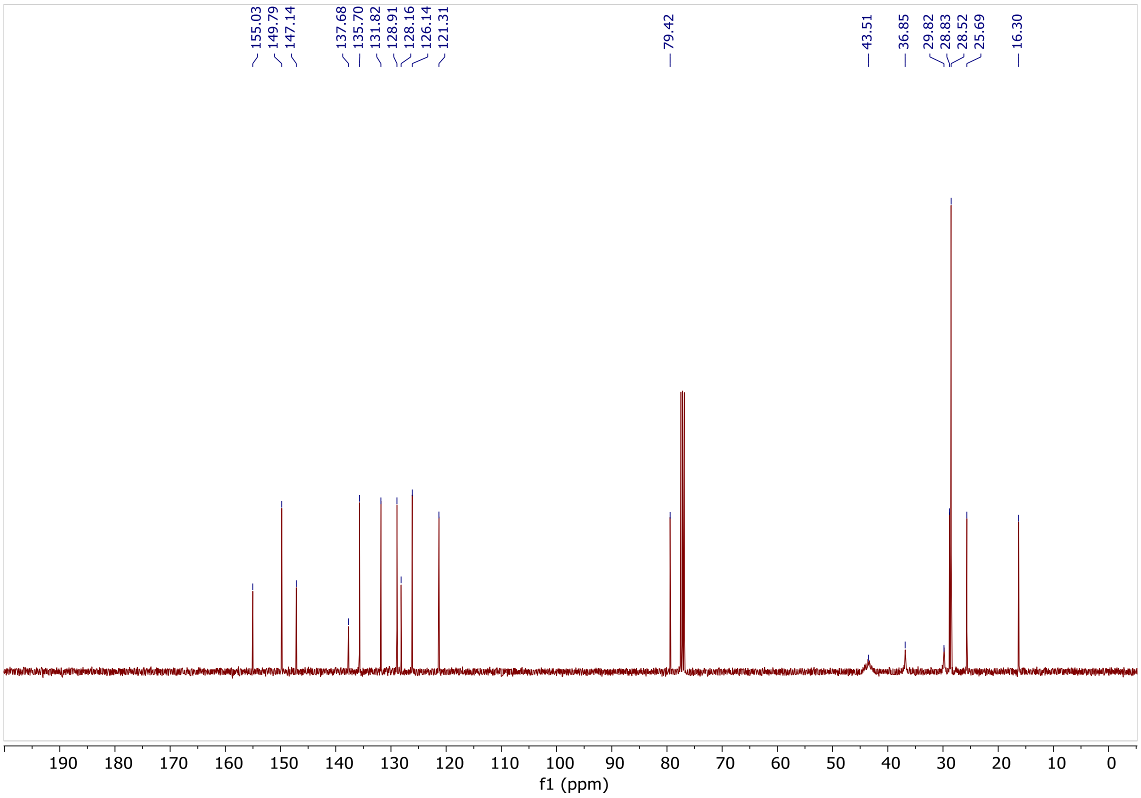

400 MHz ^{1}H NMR spectrum; 100.6 MHz ^{13}C NMR spectrum; $CDCl_3$ of **27**

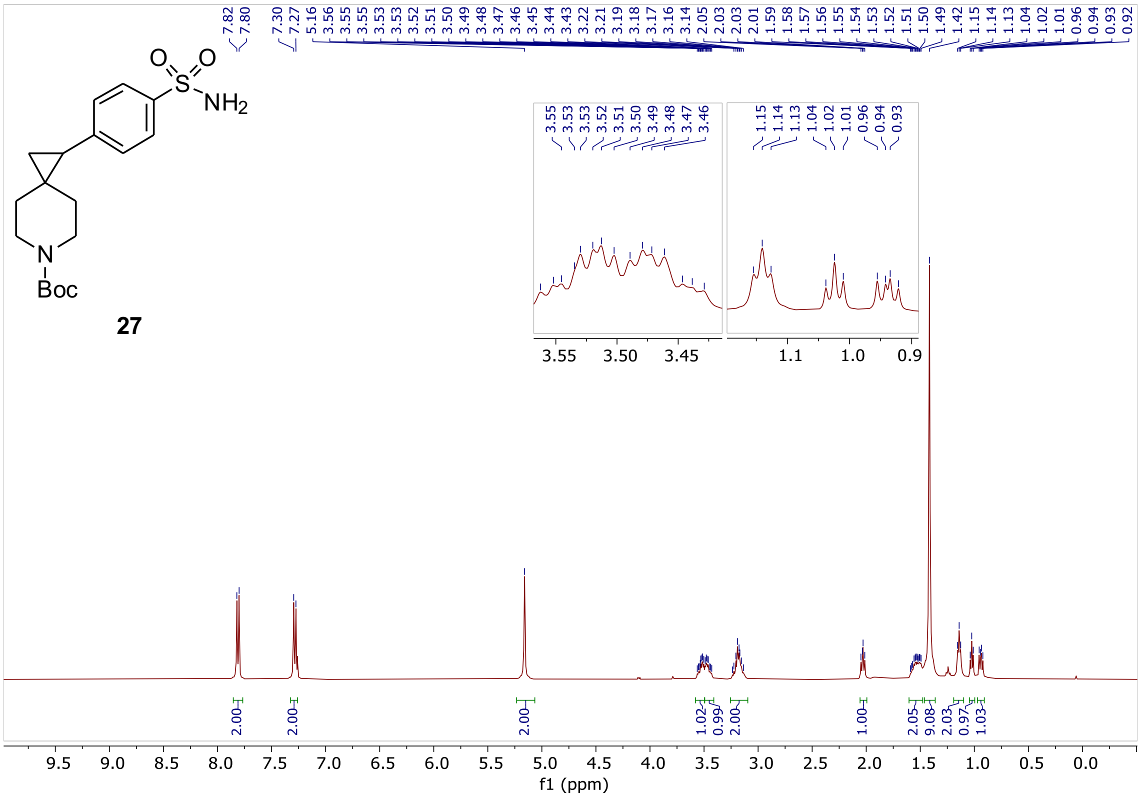

27

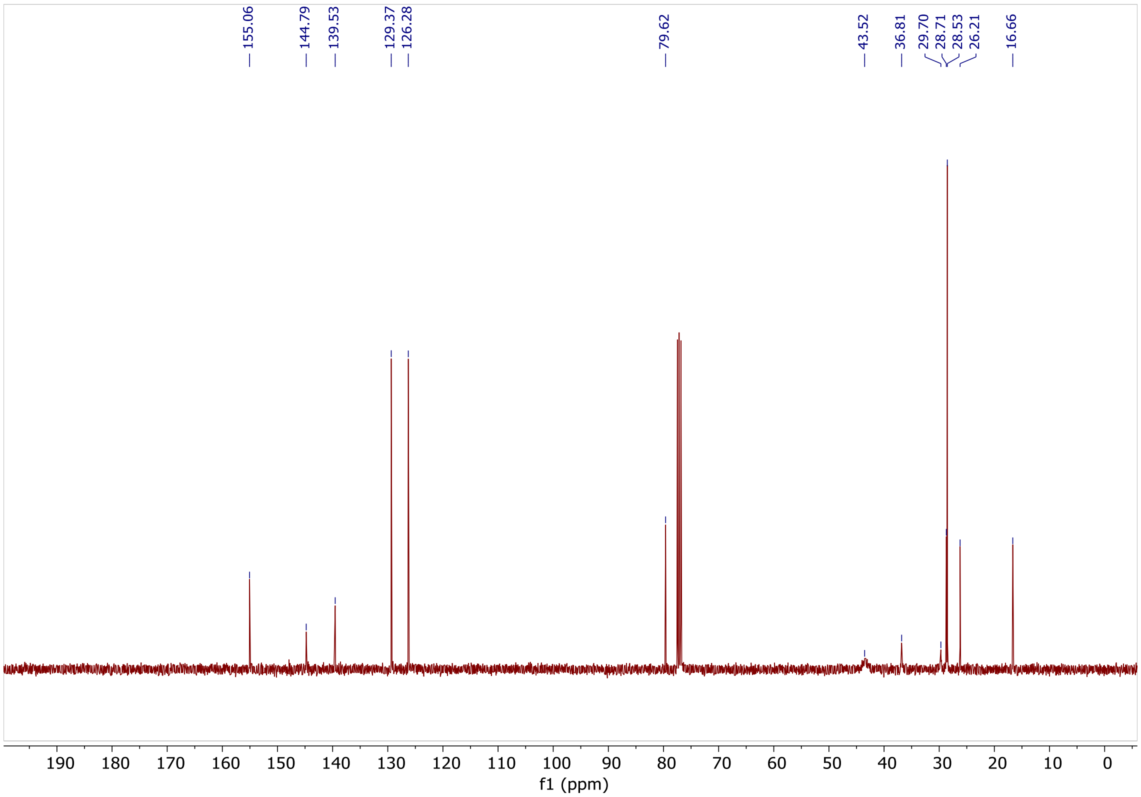

400 MHz ^{1}H NMR spectrum; 100.6 MHz ^{13}C NMR spectrum; $CDCl_3$ of **28**

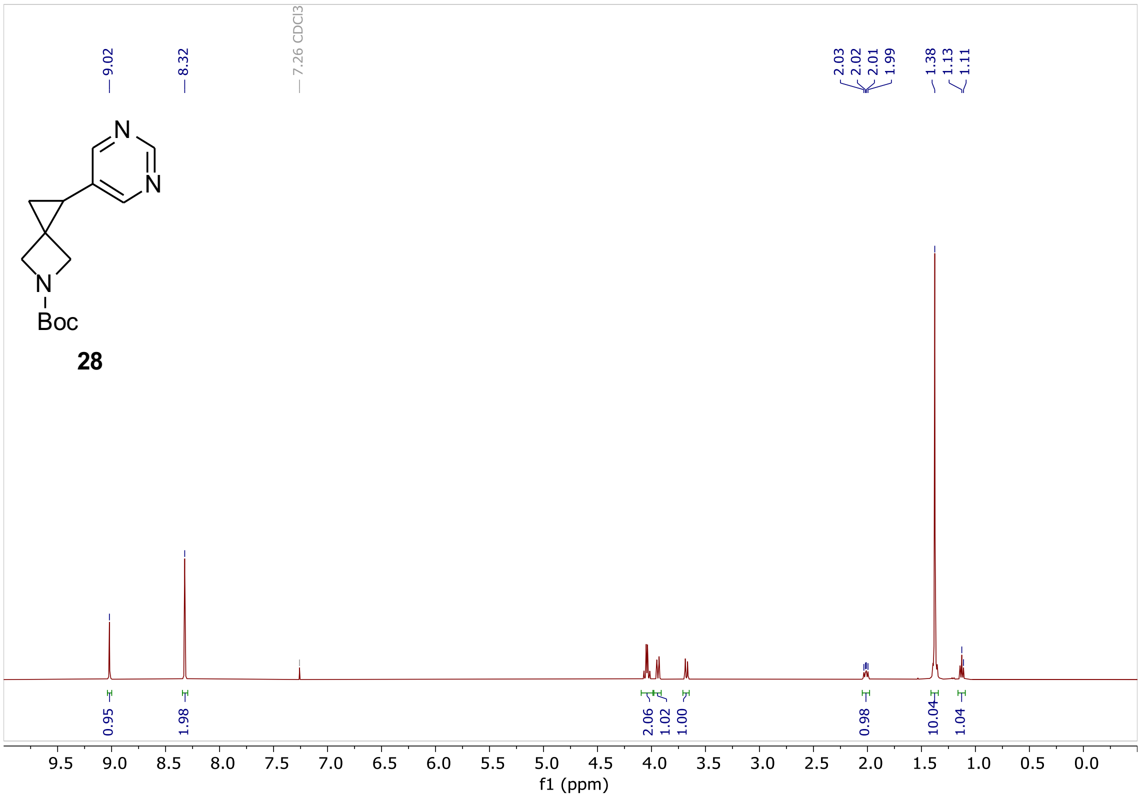

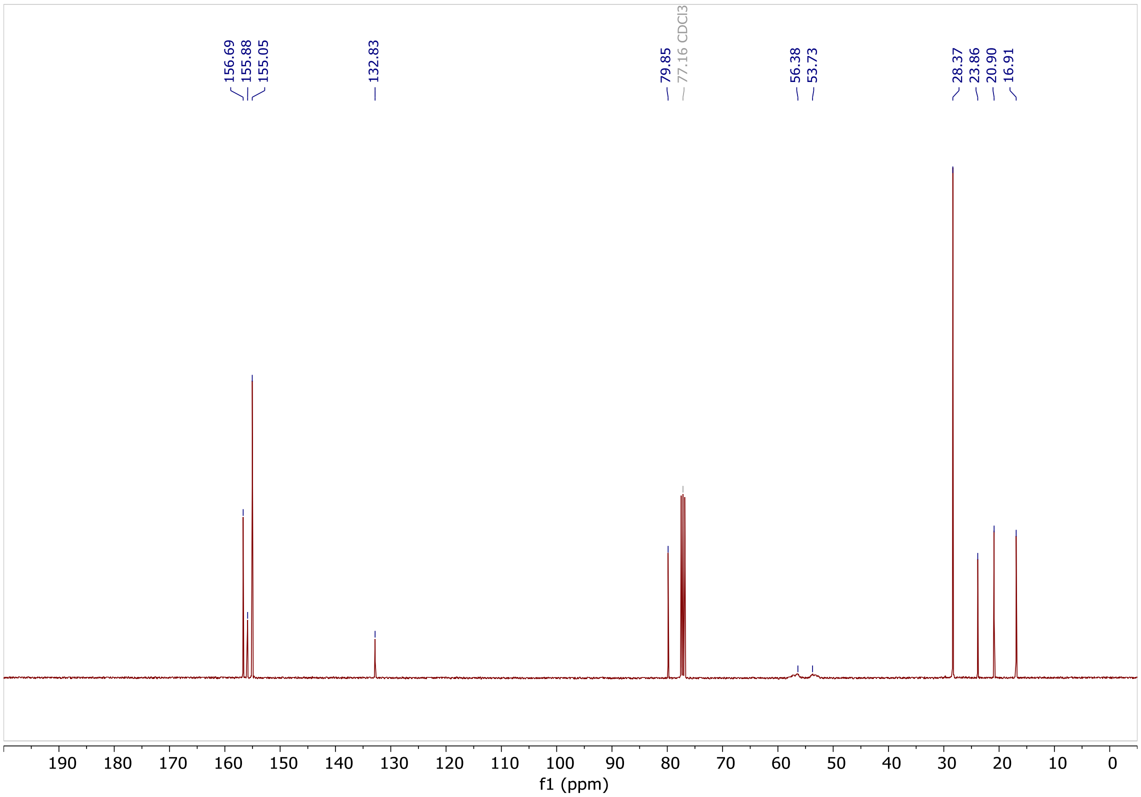

400 MHz ^{1}H NMR spectrum; 100.6 MHz ^{13}C NMR spectrum; $CDCl_3$ of **29**

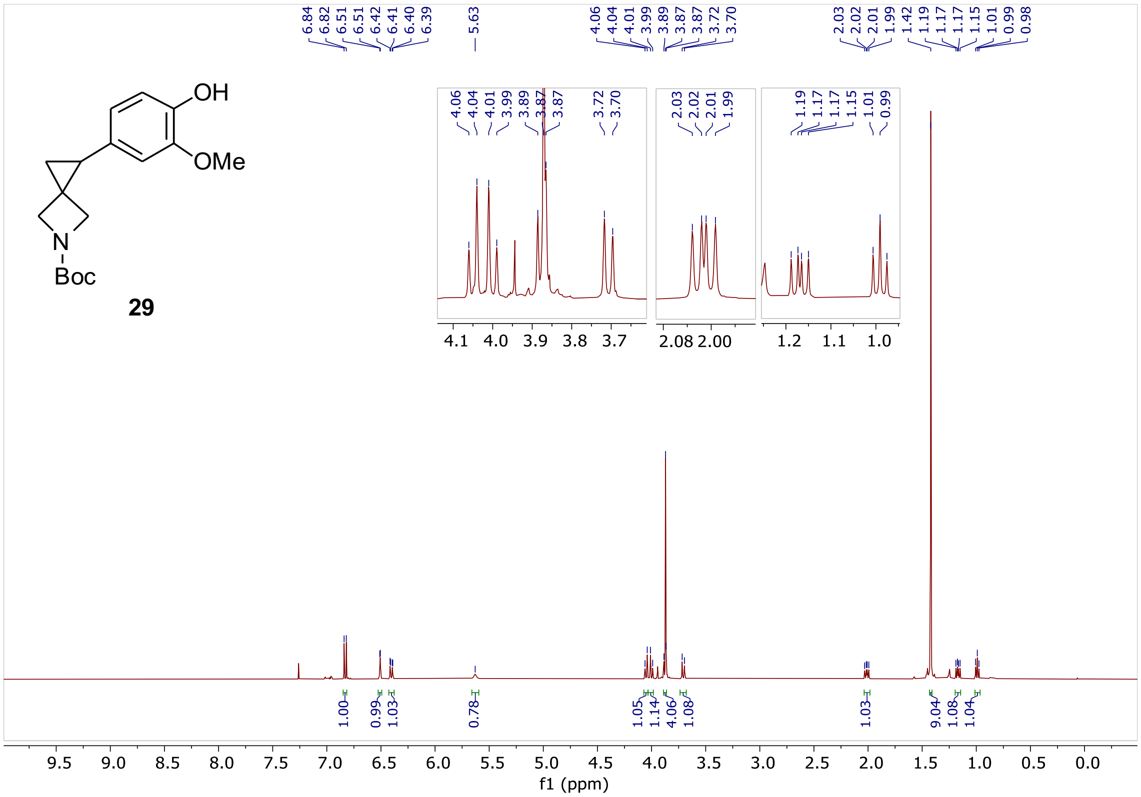

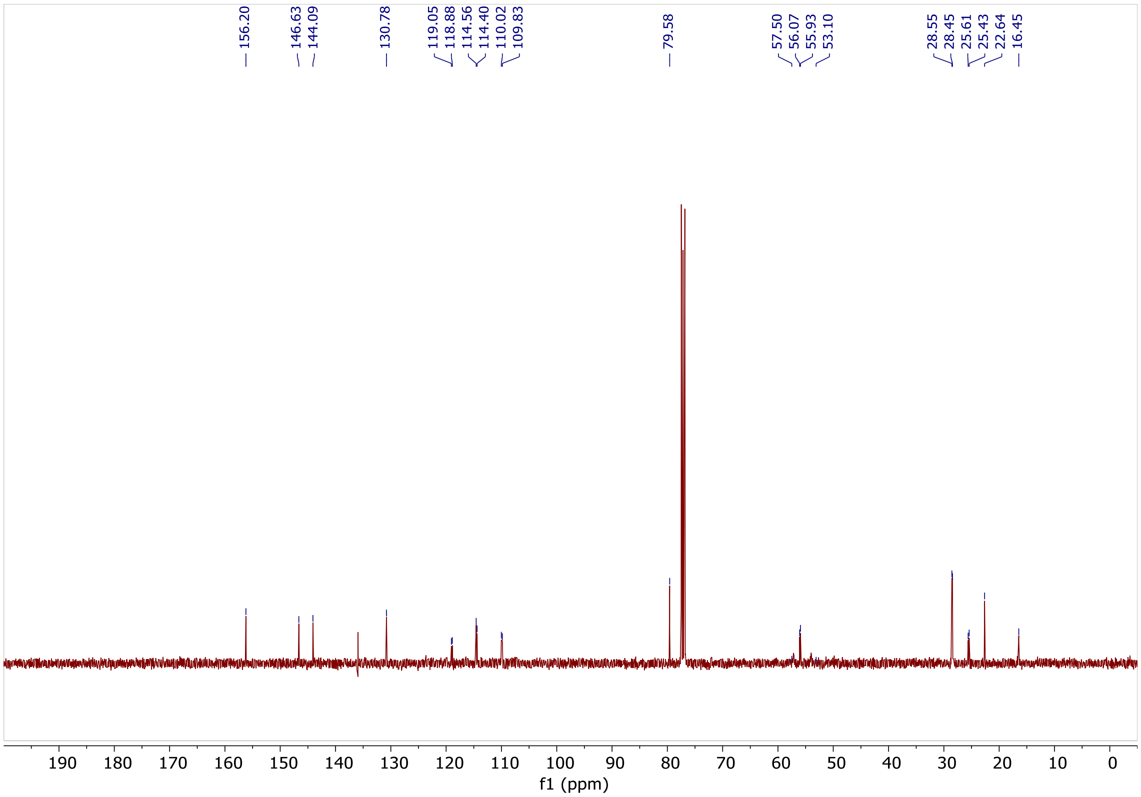

400 MHz ^{1}H NMR spectrum; 100.6 MHz ^{13}C NMR spectrum; $CDCl_3$ of **30**

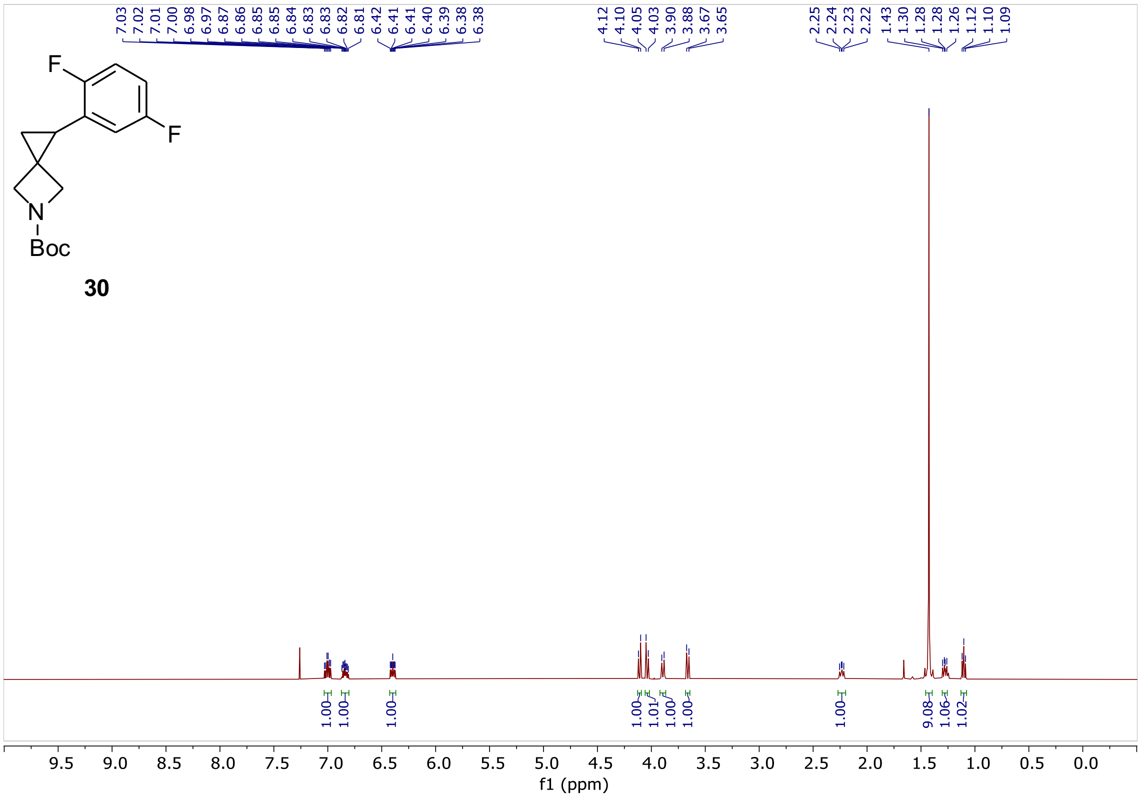

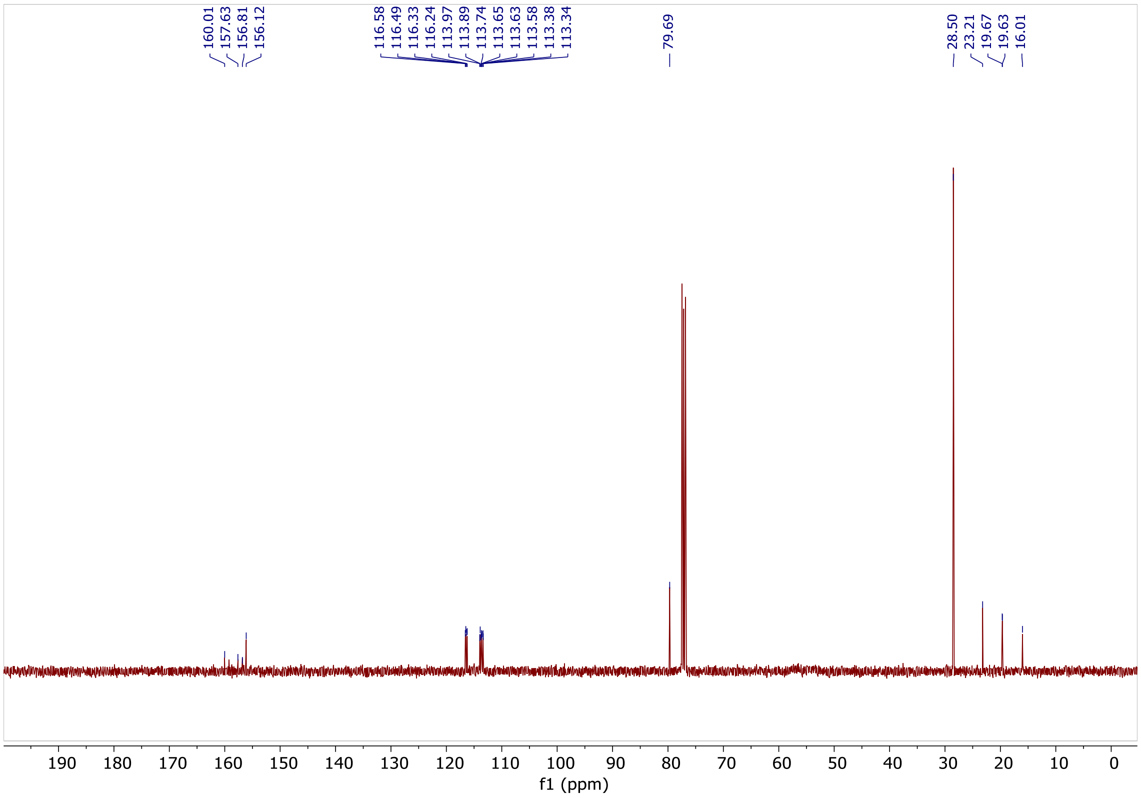

400 MHz ^{1}H NMR spectrum; 100.6 MHz ^{13}C NMR spectrum; $CDCl_3$ of **31**

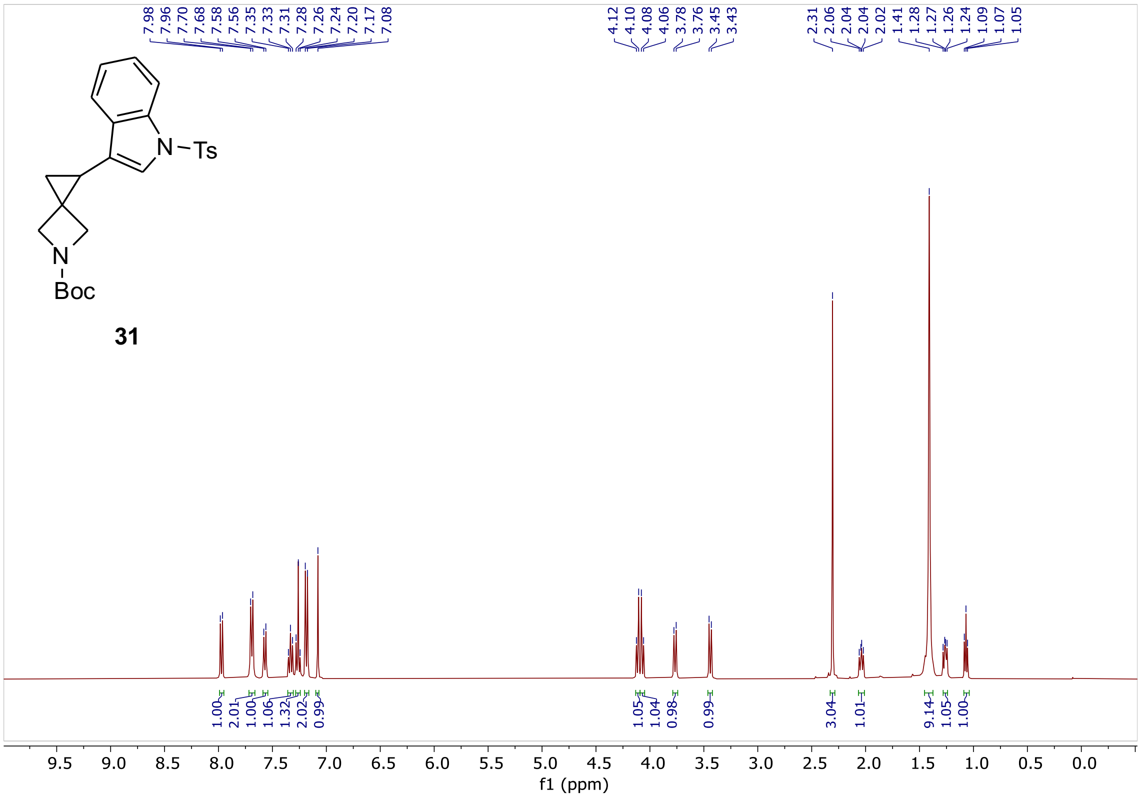

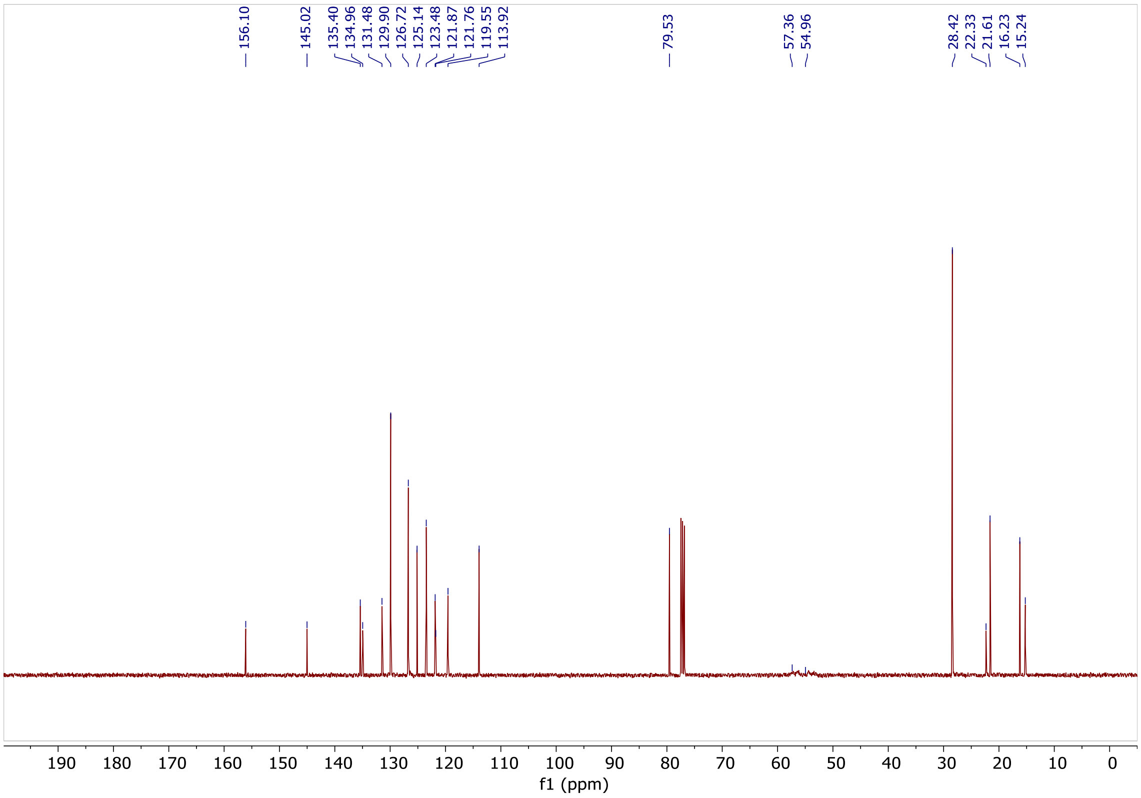

400 MHz ^{1}H NMR spectrum; 100.6 MHz ^{13}C NMR spectrum; $CDCl_3$ of **32**

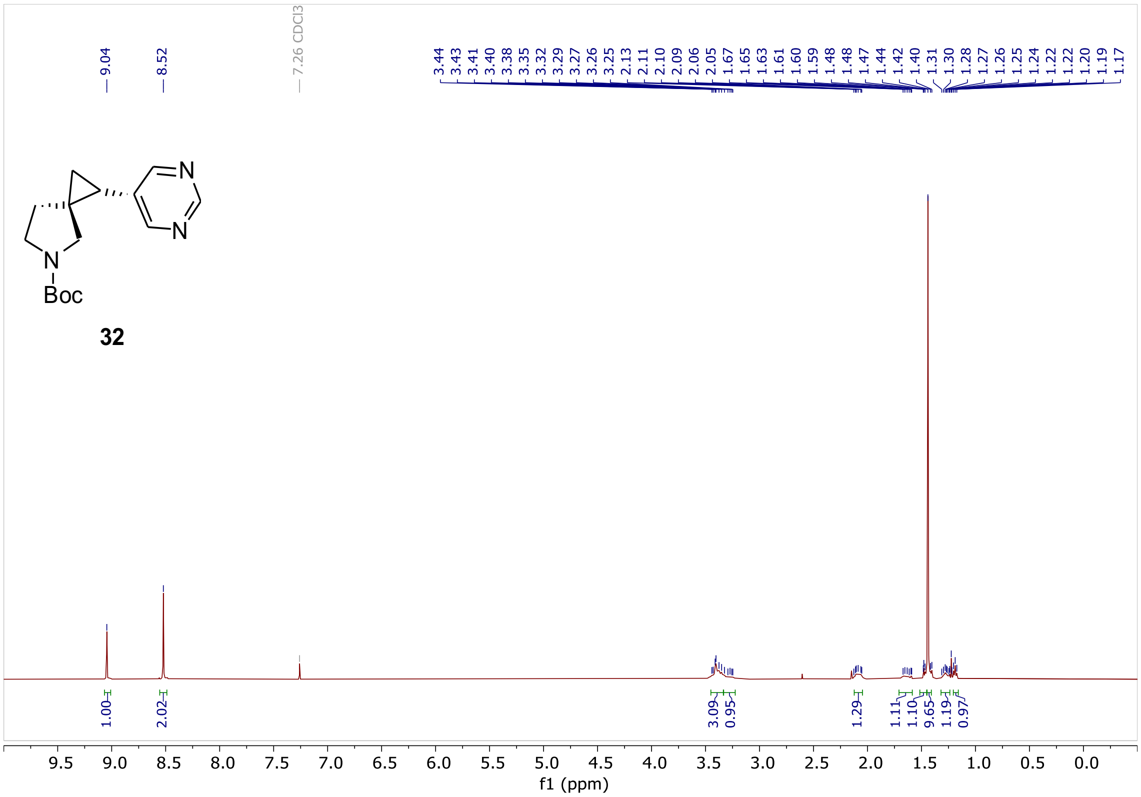

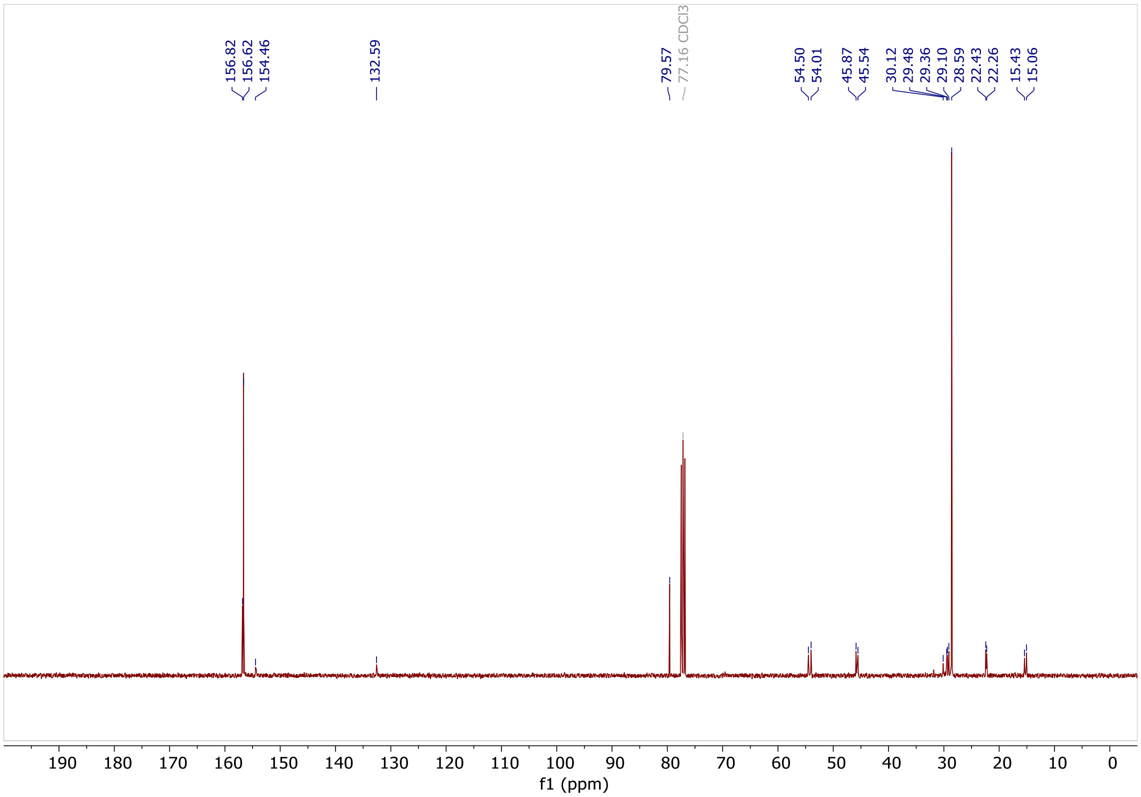

400 MHz ^{1}H NMR spectrum; 100.6 MHz ^{13}C NMR spectrum; $CDCl_3$ of **33**

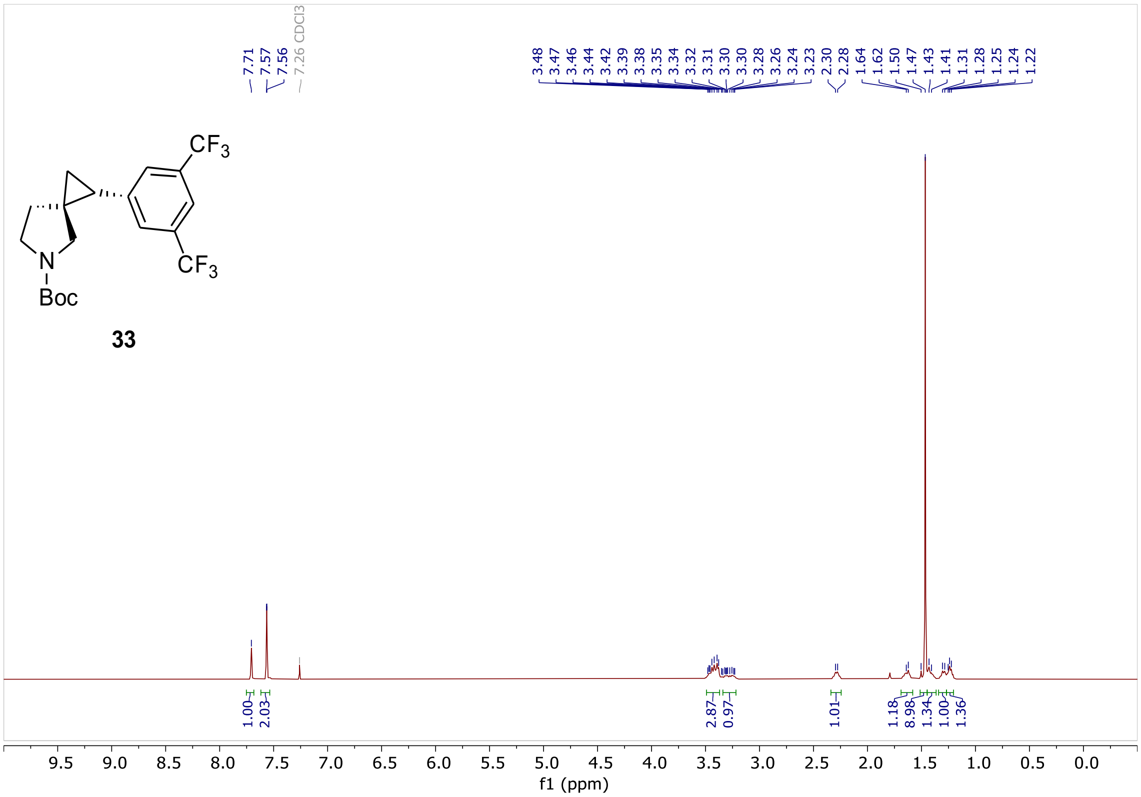

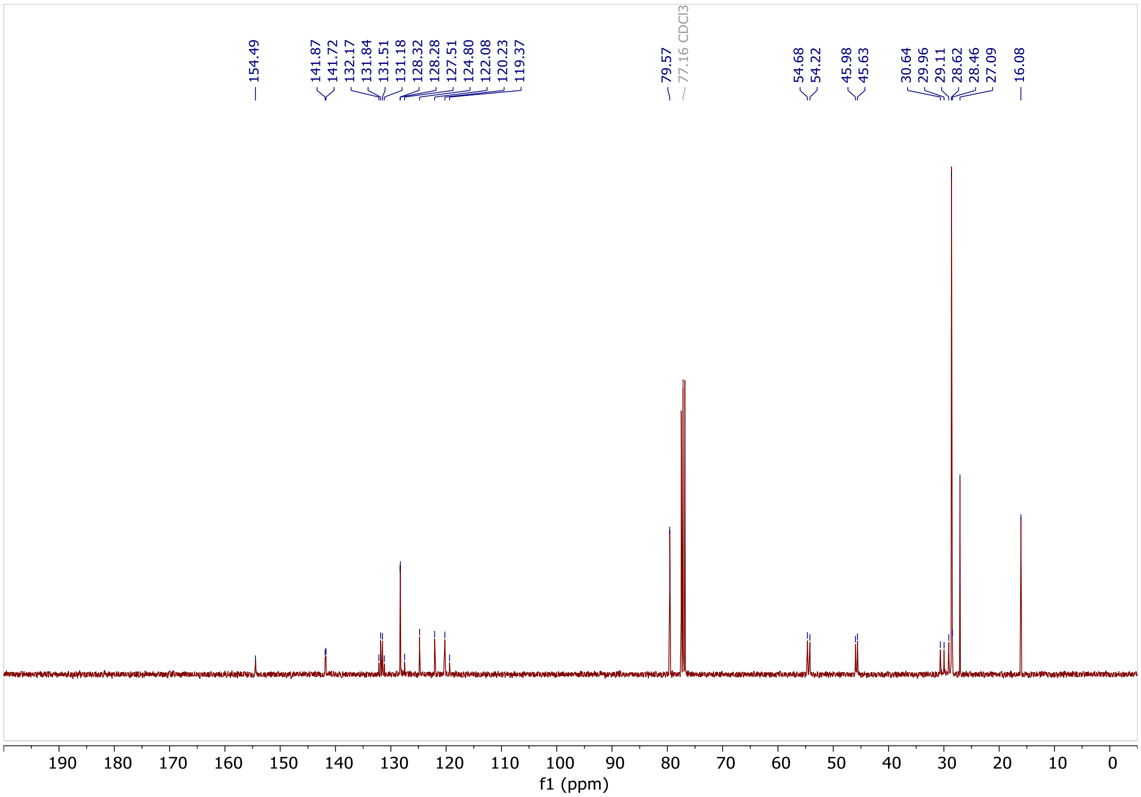

400 MHz ^{1}H NMR spectrum; 100.6 MHz ^{13}C NMR spectrum; $CDCl_3$ of **34**

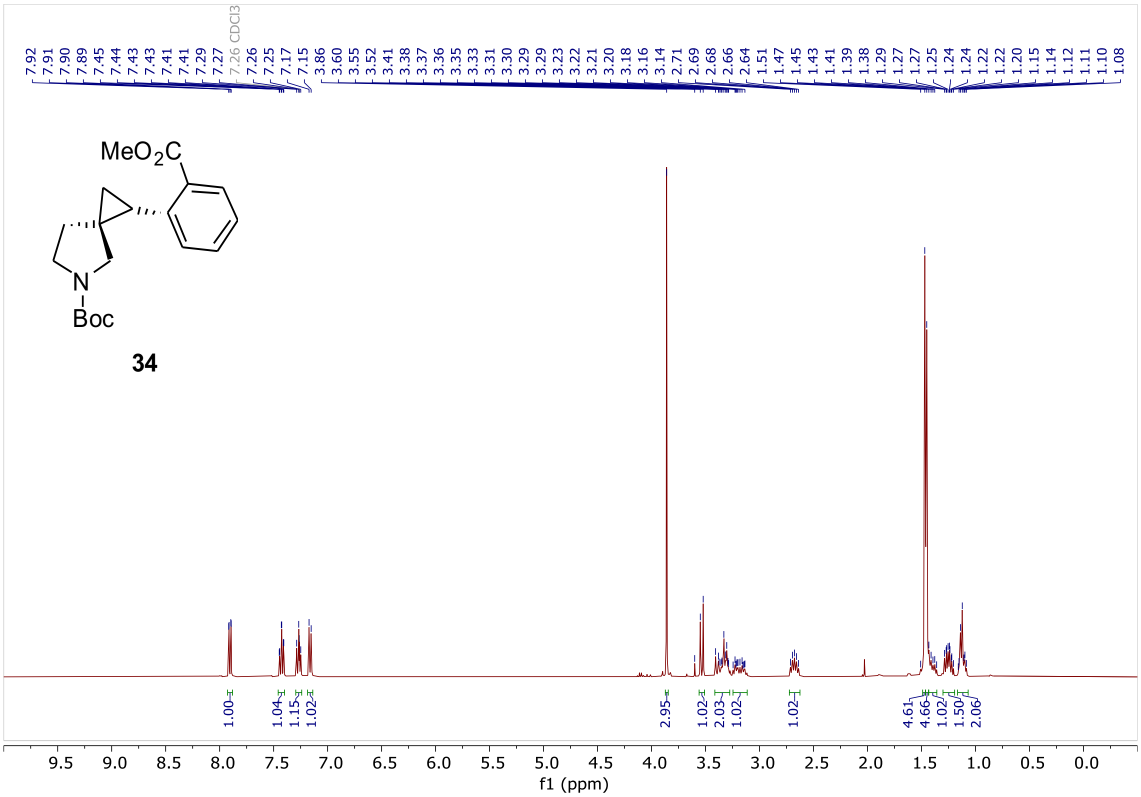

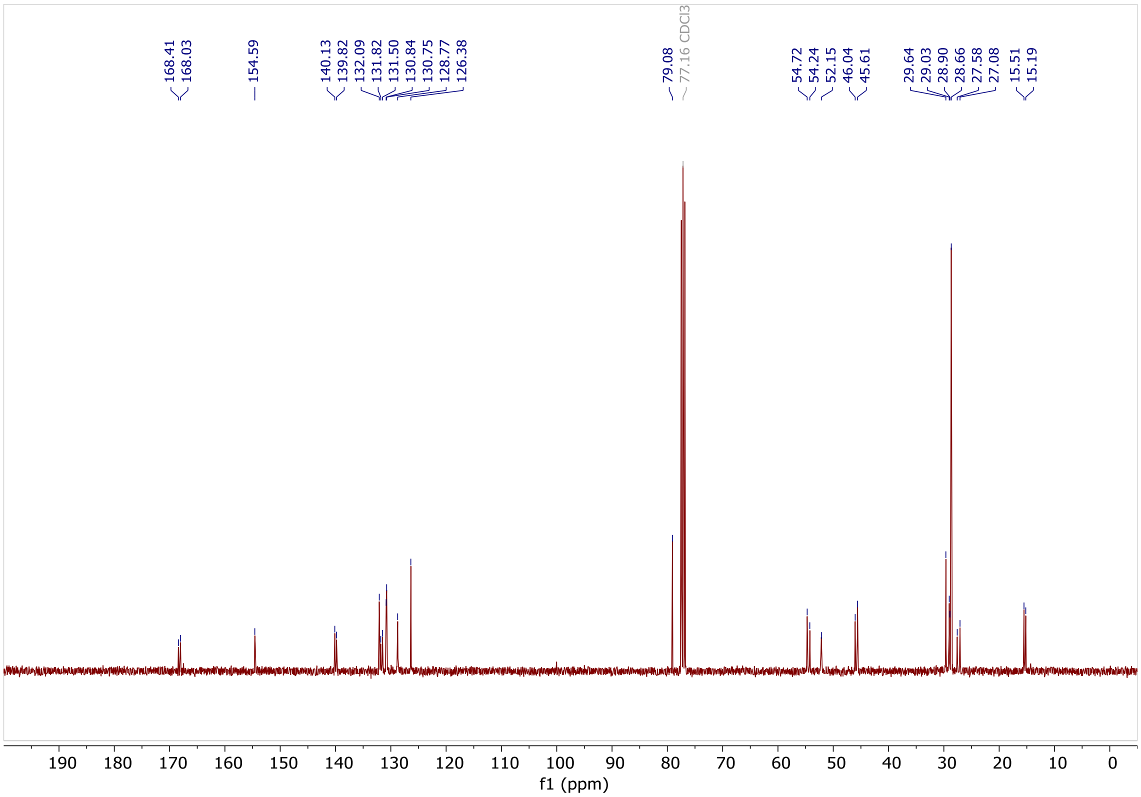

400 MHz ^1H NMR spectrum; 100.6 MHz ^{13}C NMR spectrum; $CDCl_3$ of **35**

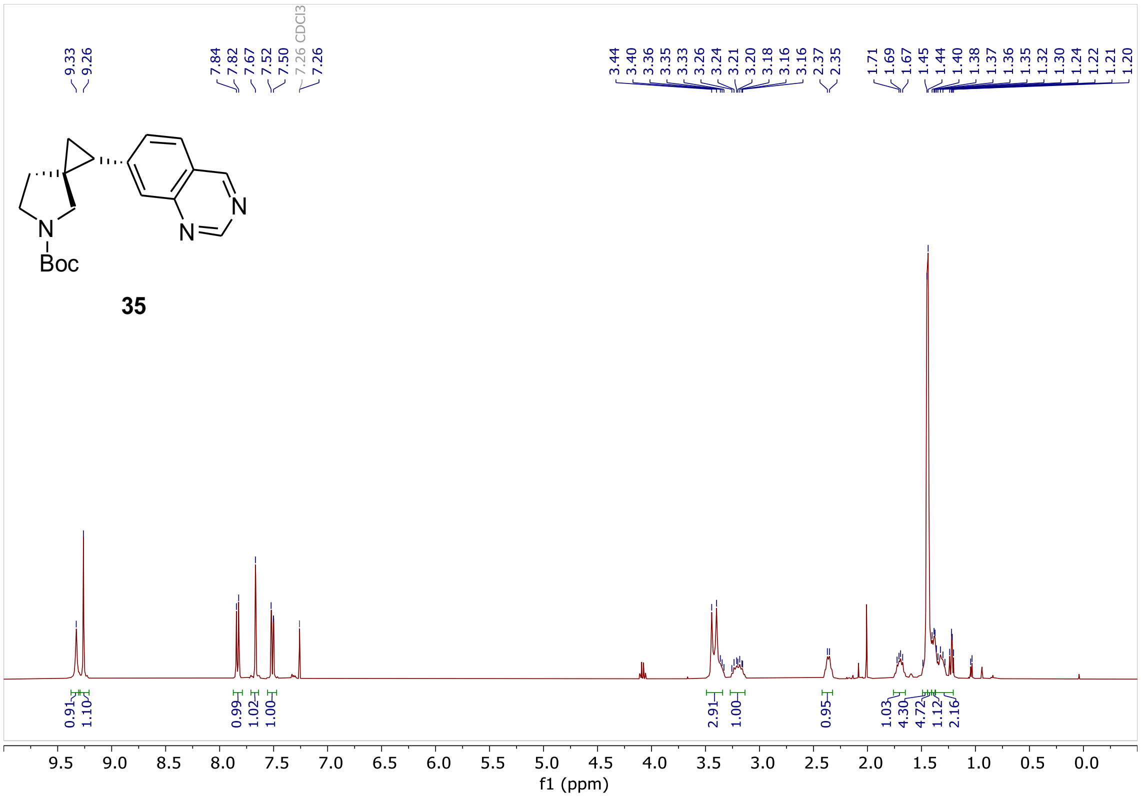

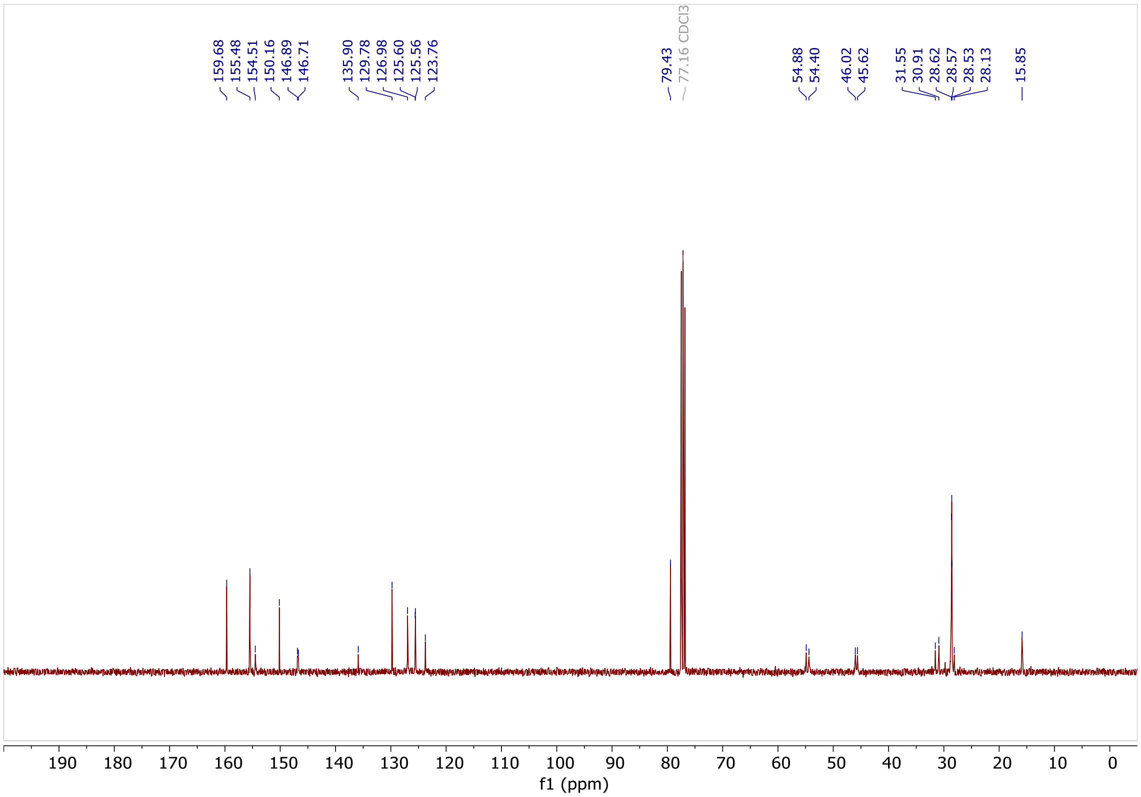

400 MHz ^{1}H NMR spectrum; 100.6 MHz ^{13}C NMR spectrum; $CDCl_3$ of **36**

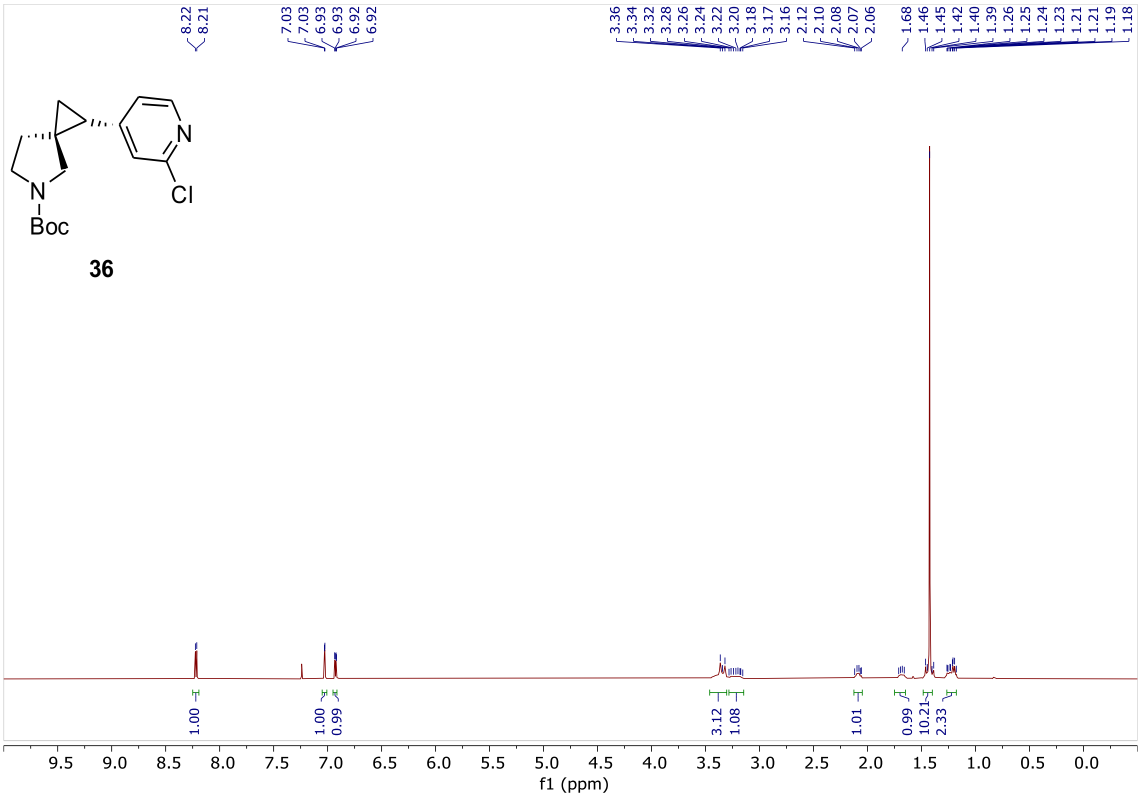

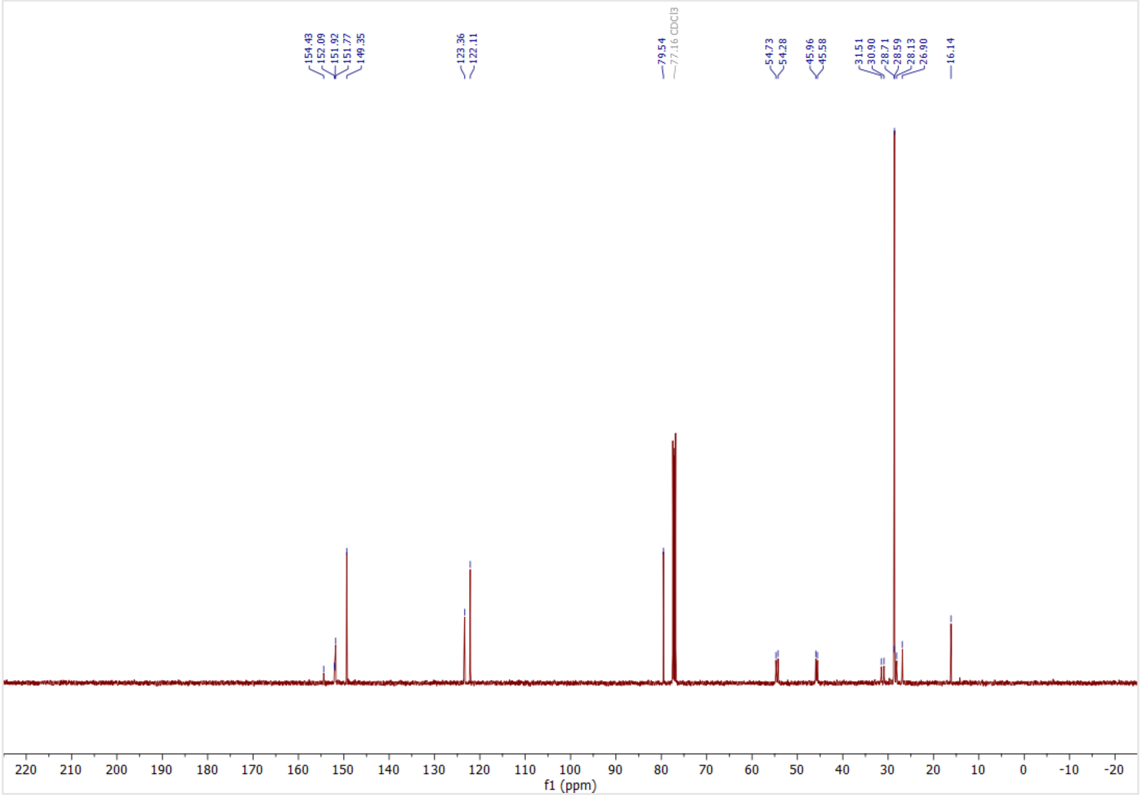

400 MHz ^{1}H NMR spectrum; 100.6 MHz ^{13}C NMR spectrum; $CDCl_3$ of **37**

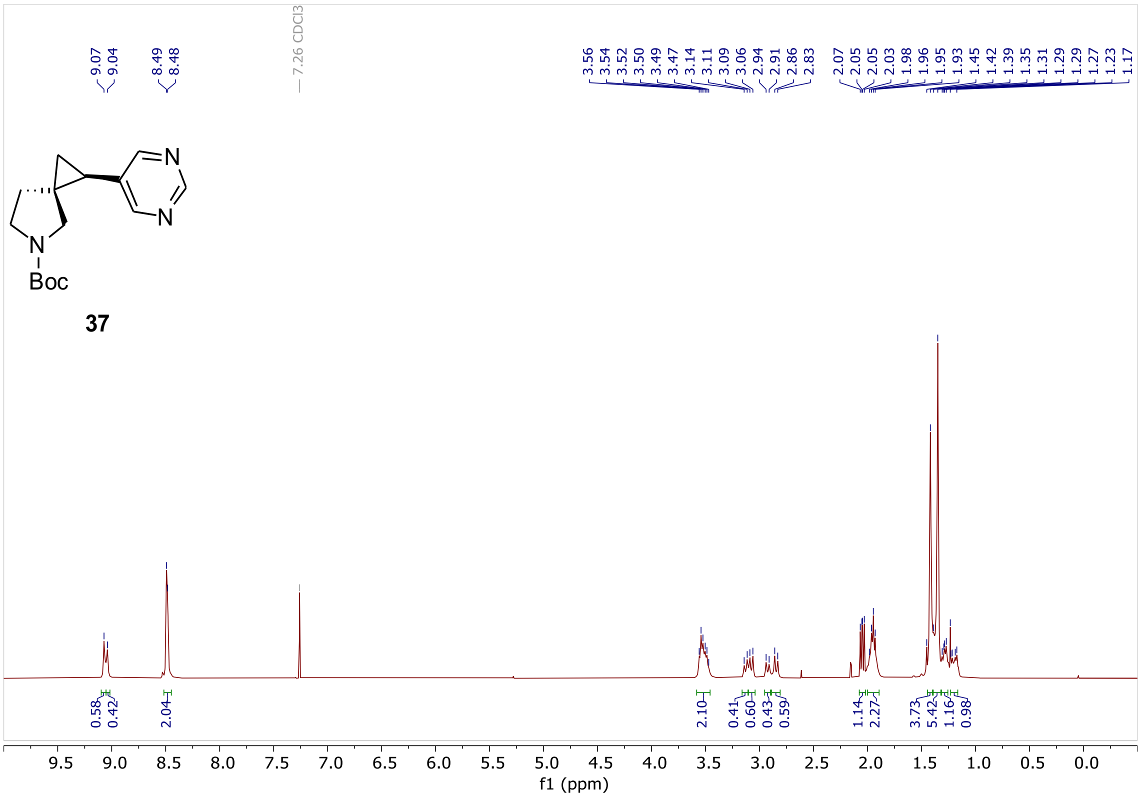

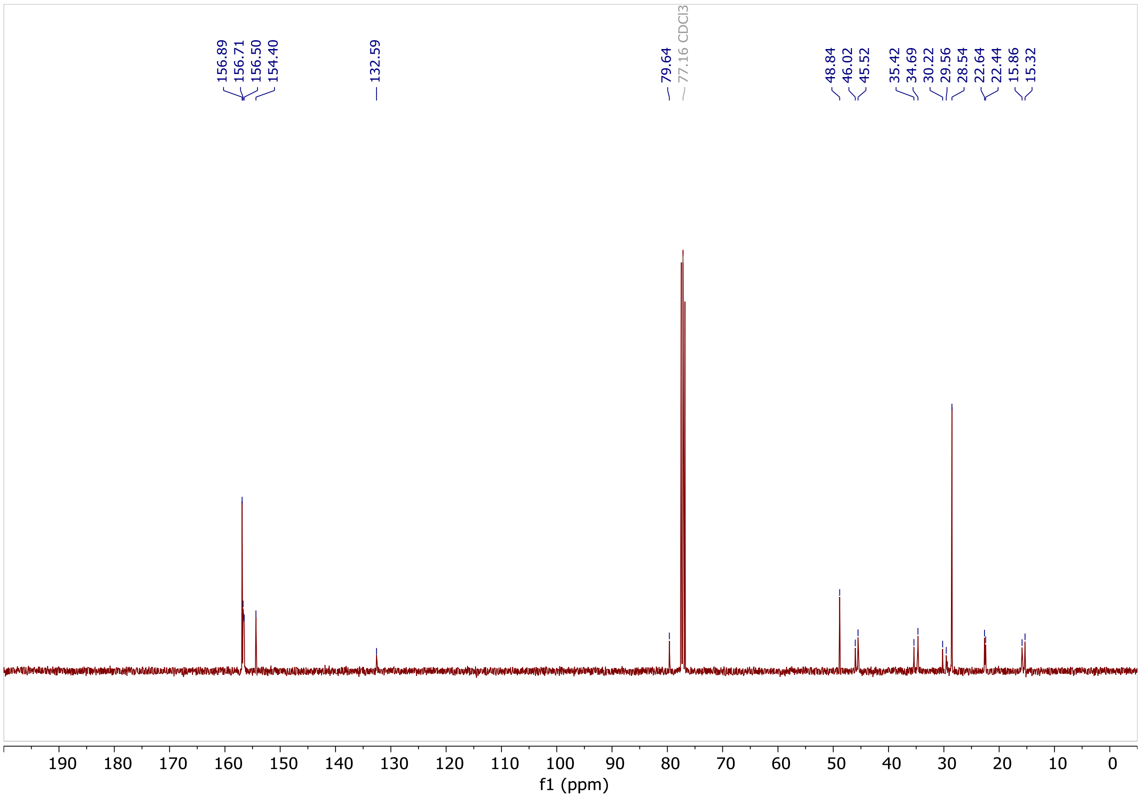

400 MHz ^{1}H NMR spectrum; 100.6 MHz ^{13}C NMR spectrum; $CDCl_3$ of **38**

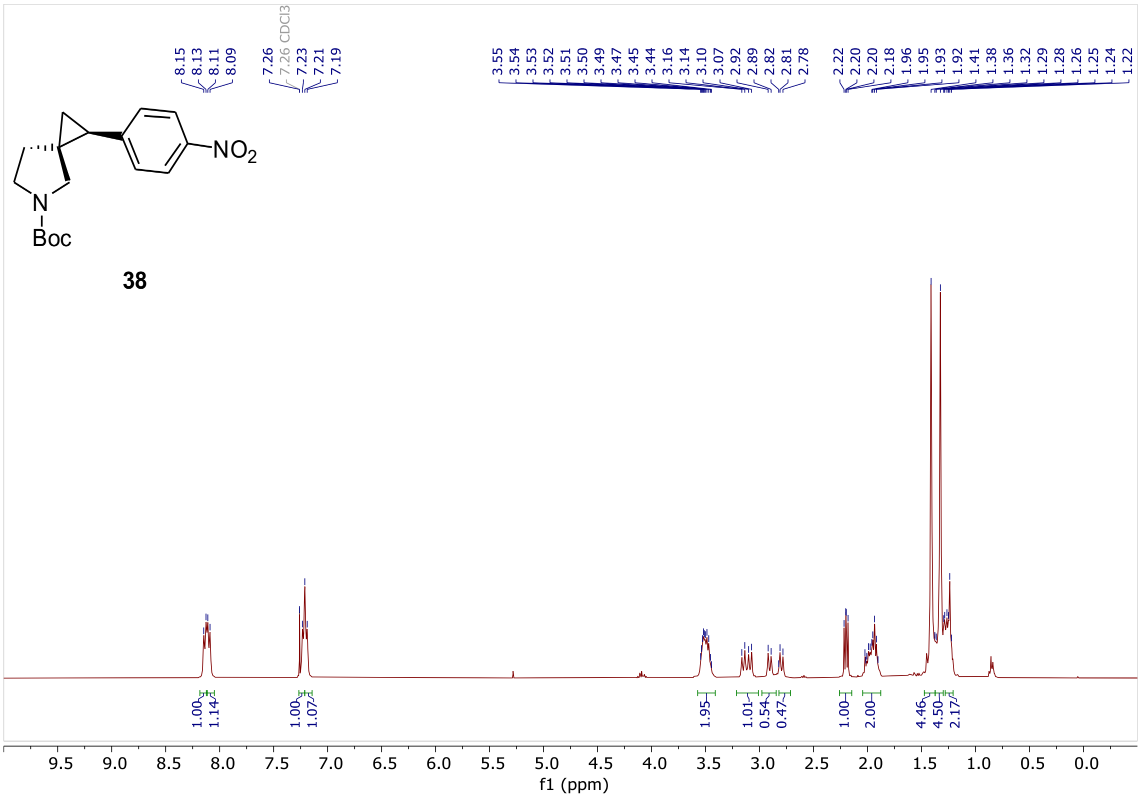

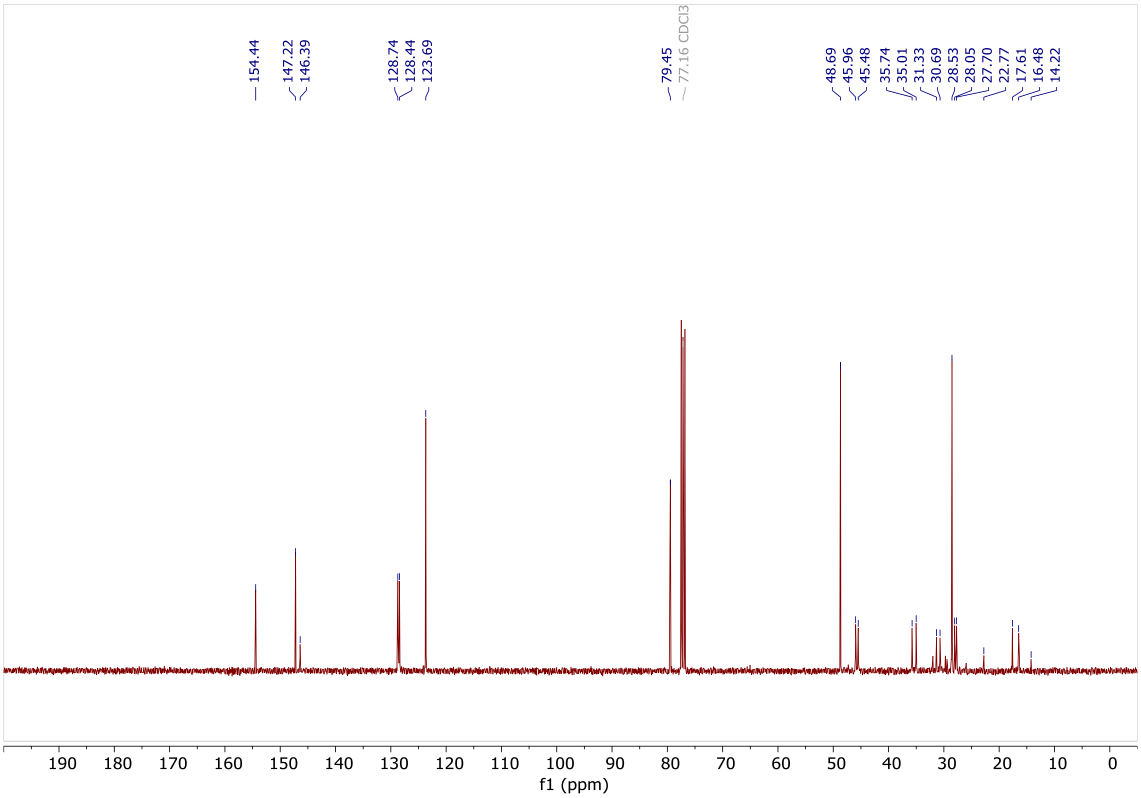

400 MHz ^{1}H NMR spectrum; 100.6 MHz ^{13}C NMR spectrum; $CDCl_3$ of **39**

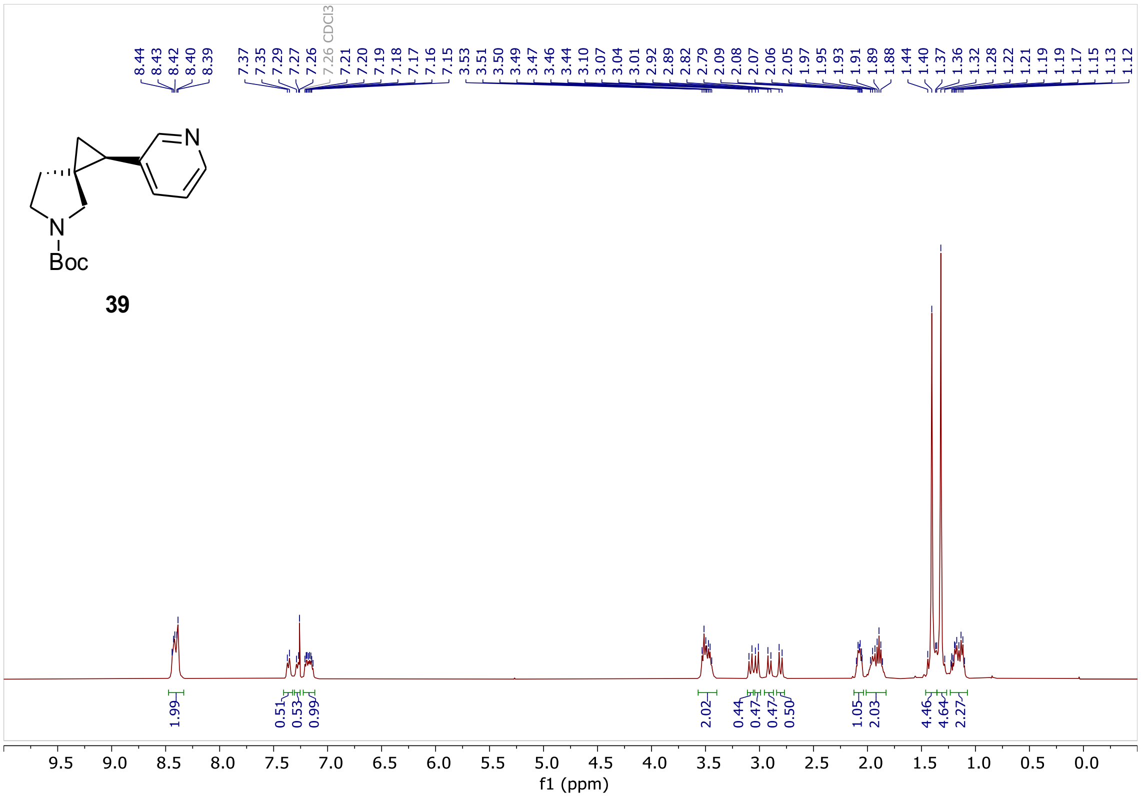

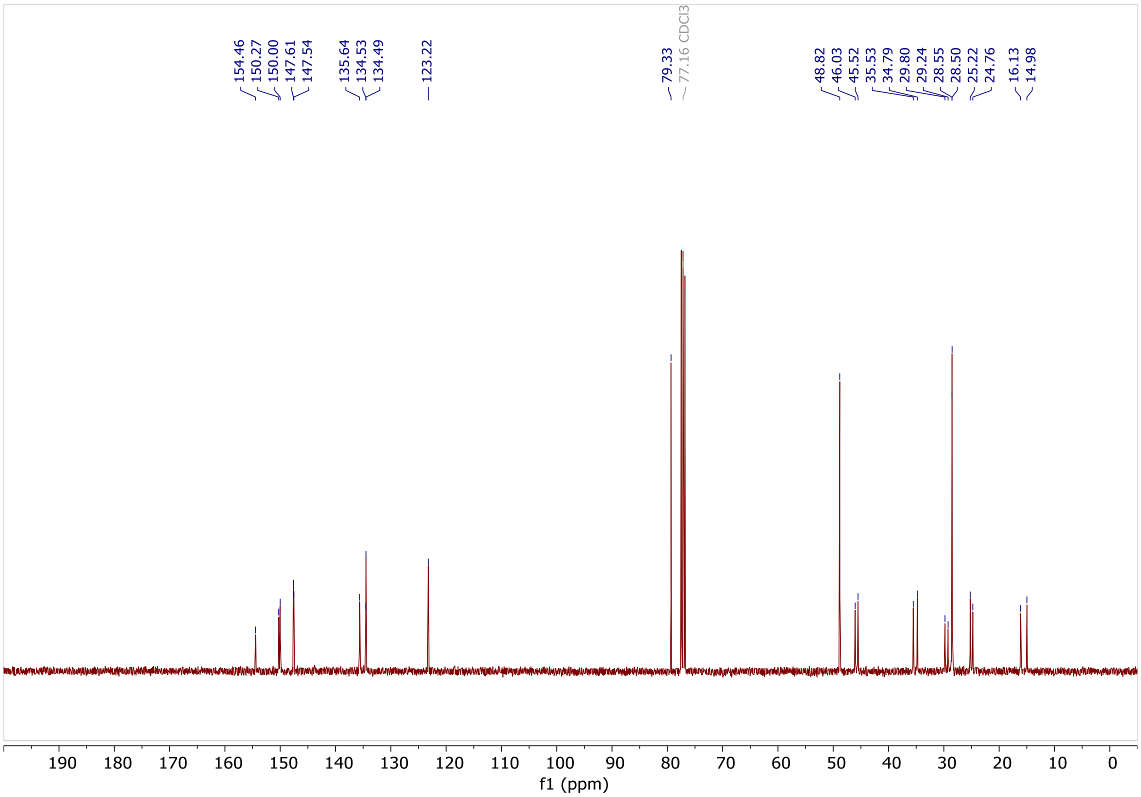

400 MHz ^{1}H NMR spectrum; 100.6 MHz ^{13}C NMR spectrum; $CDCl_3$ of **40**

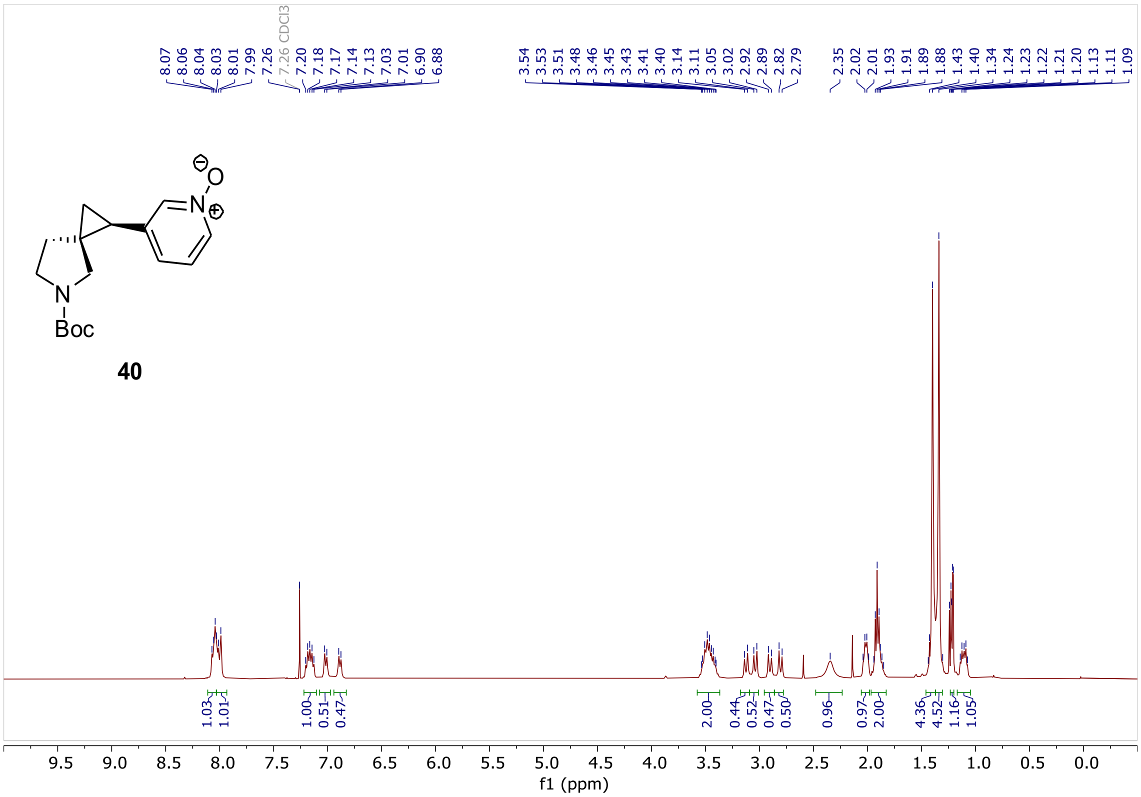

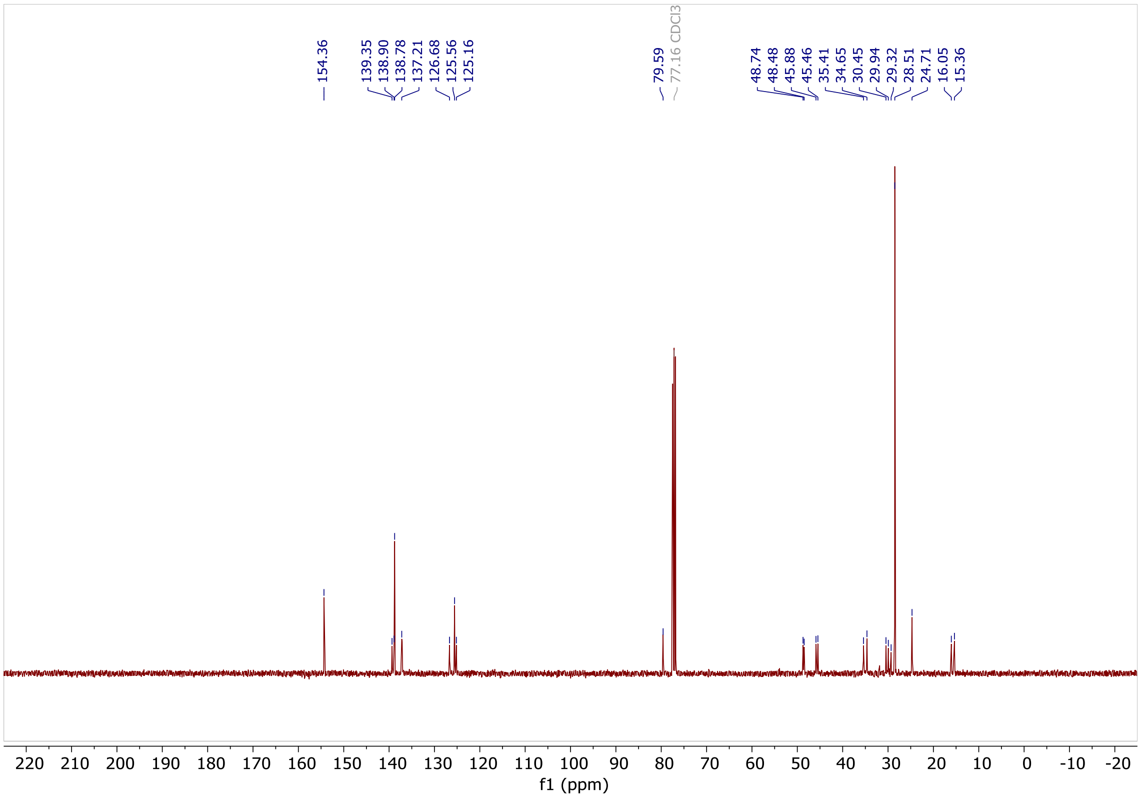

400 MHz ^{1}H NMR spectrum; 100.6 MHz ^{13}C NMR spectrum; $CDCl_3$ of **41**

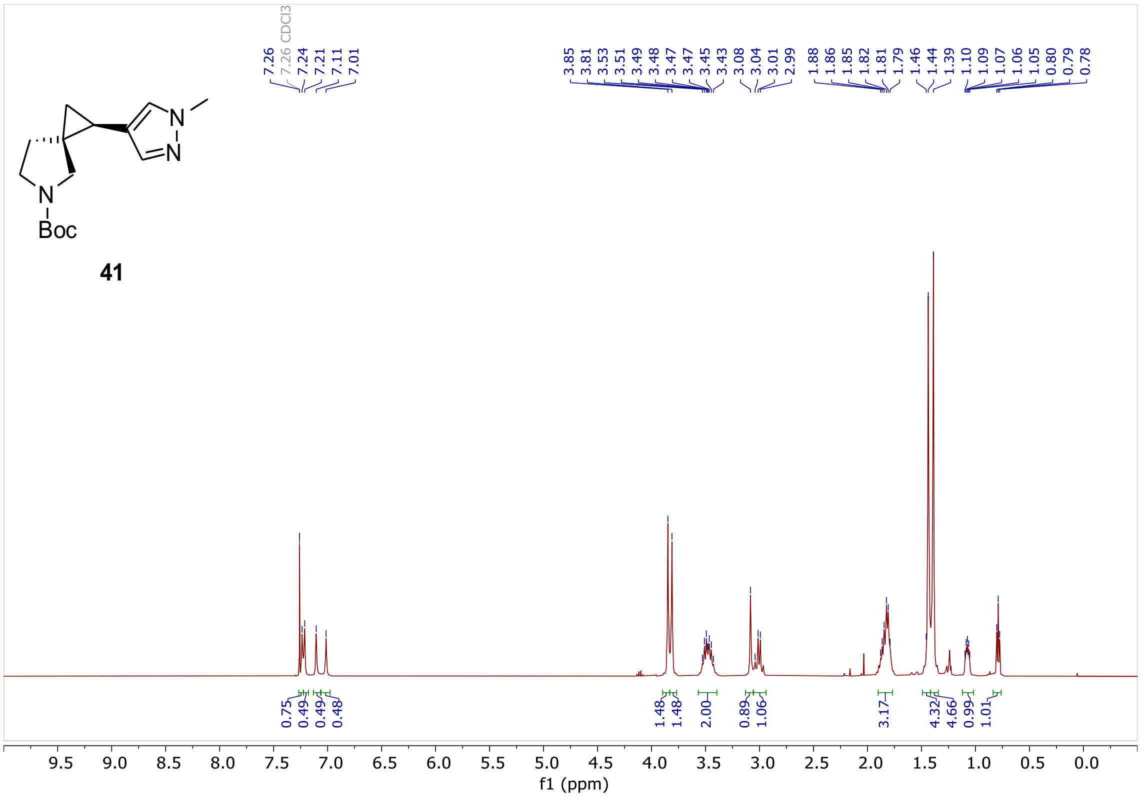

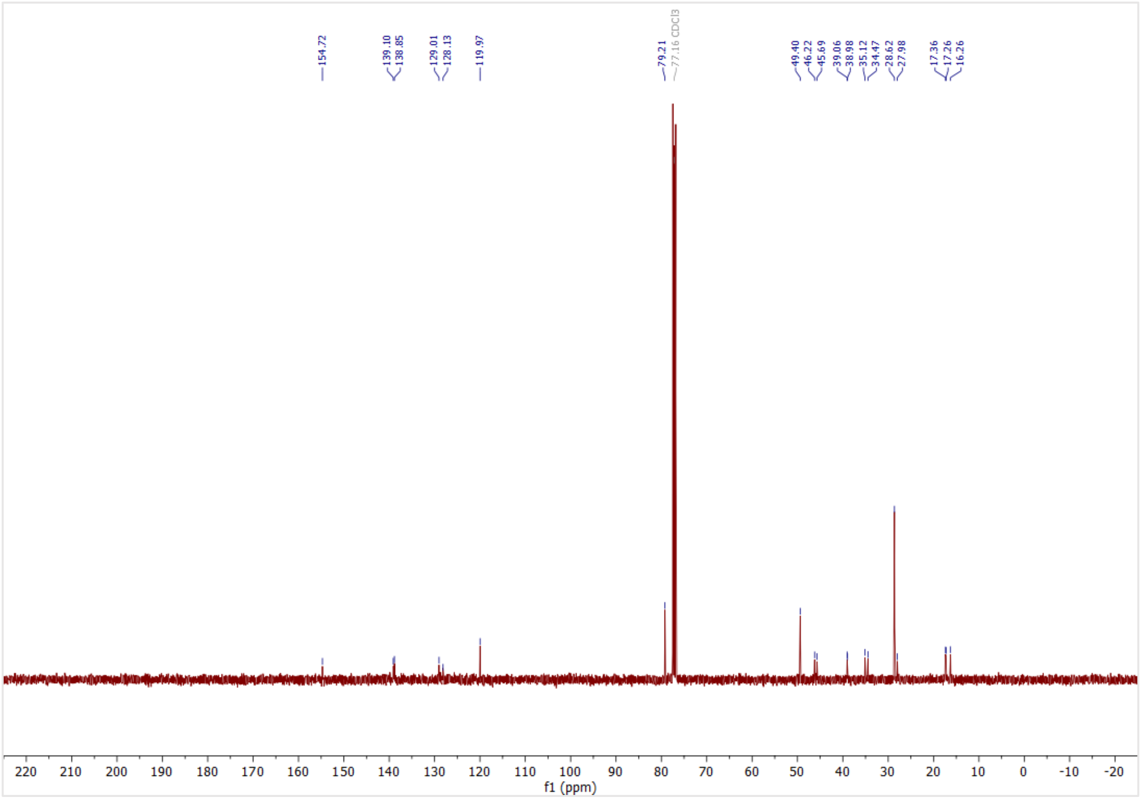

400 MHz ^{1}H NMR spectrum; 100.6 MHz ^{13}C NMR spectrum; $CDCl_3$ of **43**

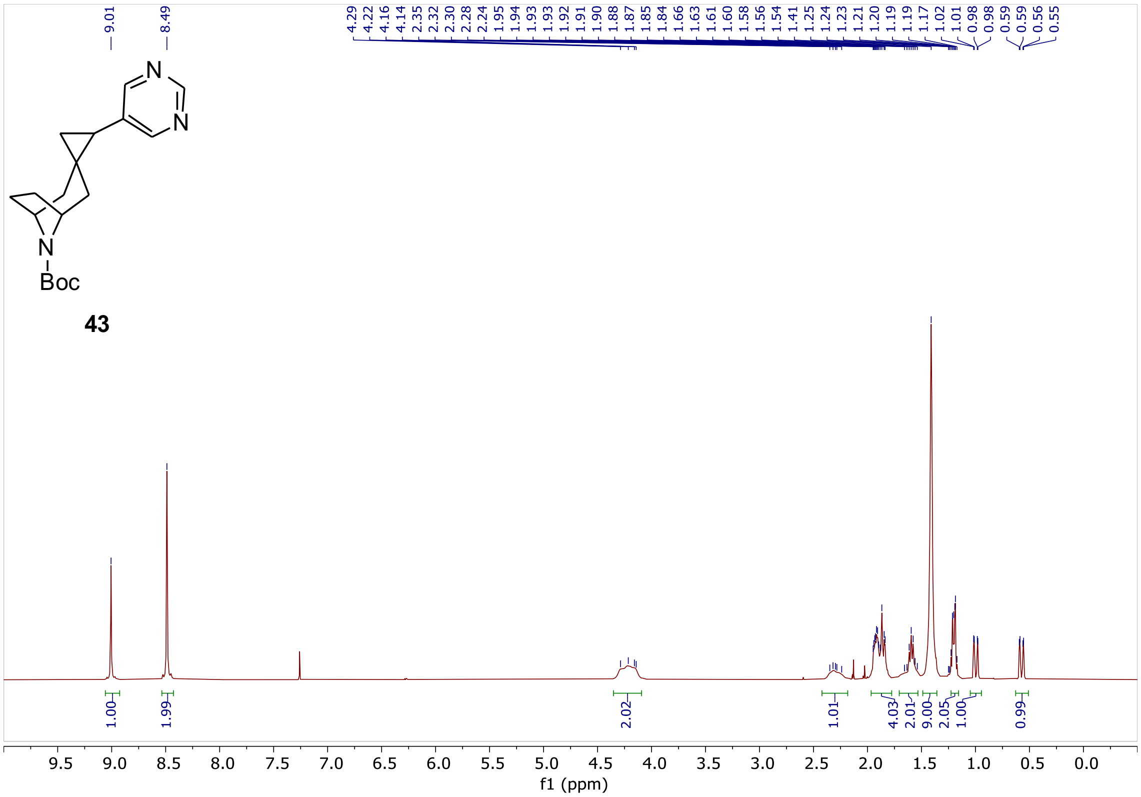

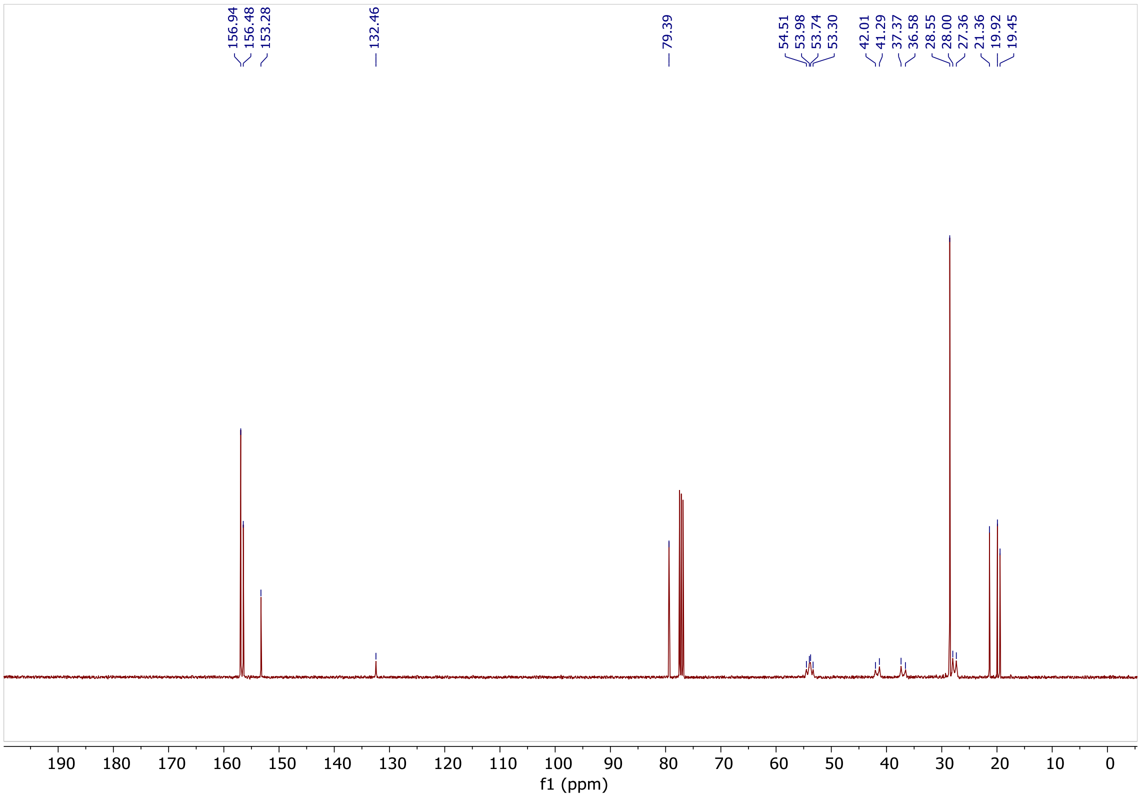

400 MHz ^{1}H NMR spectrum; 100.6 MHz ^{13}C NMR spectrum; $CDCl_3$ of **44**

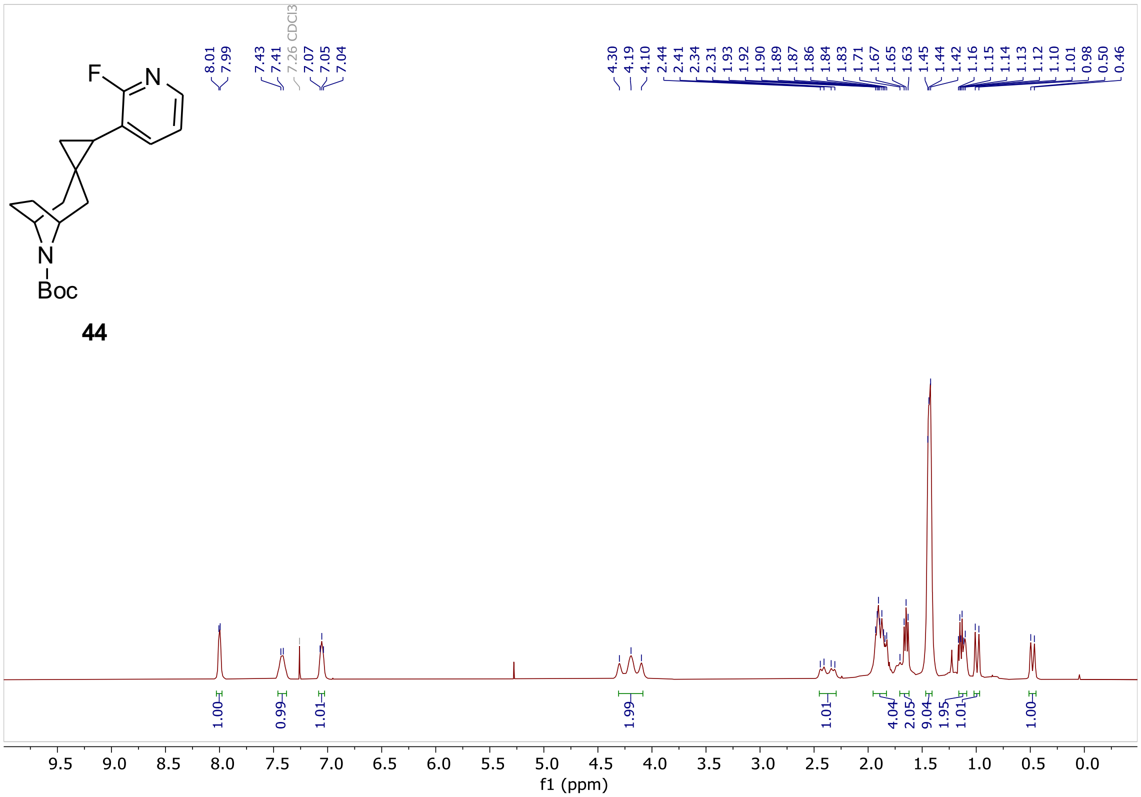

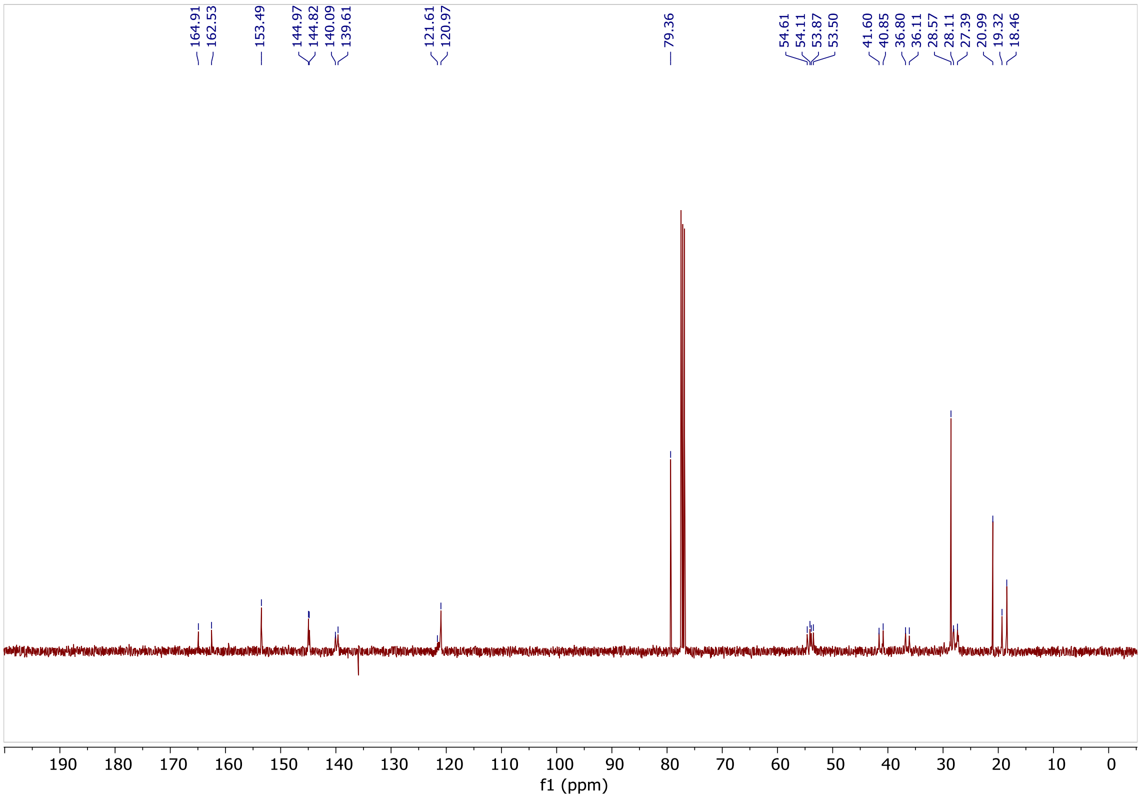

400 MHz ^{1}H NMR spectrum; 100.6 MHz ^{13}C NMR spectrum; $CDCl_3$ of **45**

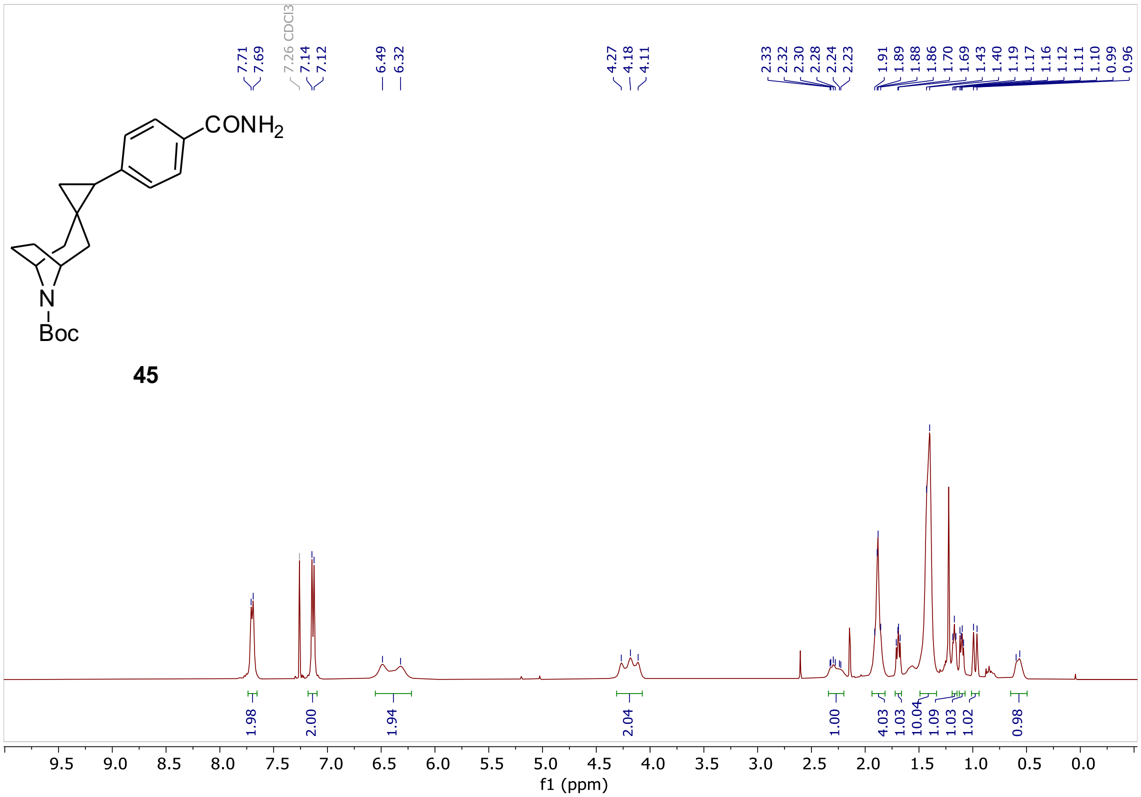

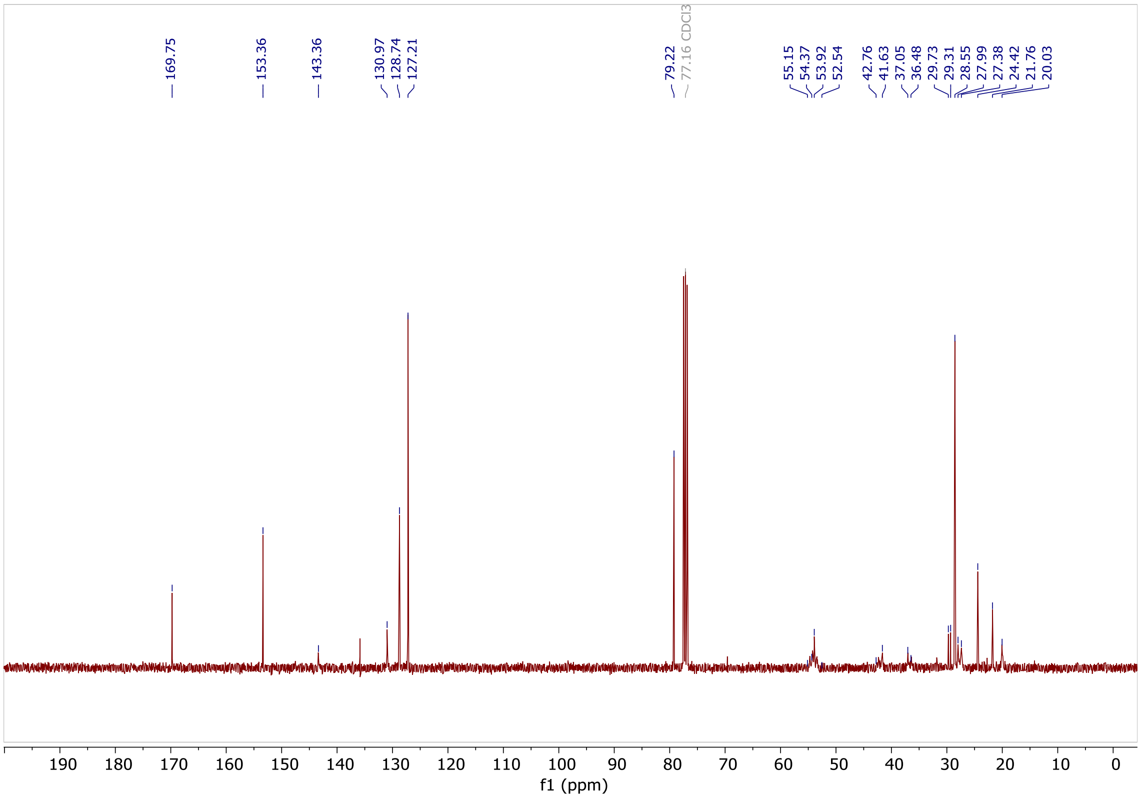

400 MHz ^{1}H NMR spectrum; 100.6 MHz ^{13}C NMR spectrum; $CDCl_3$ of **46**

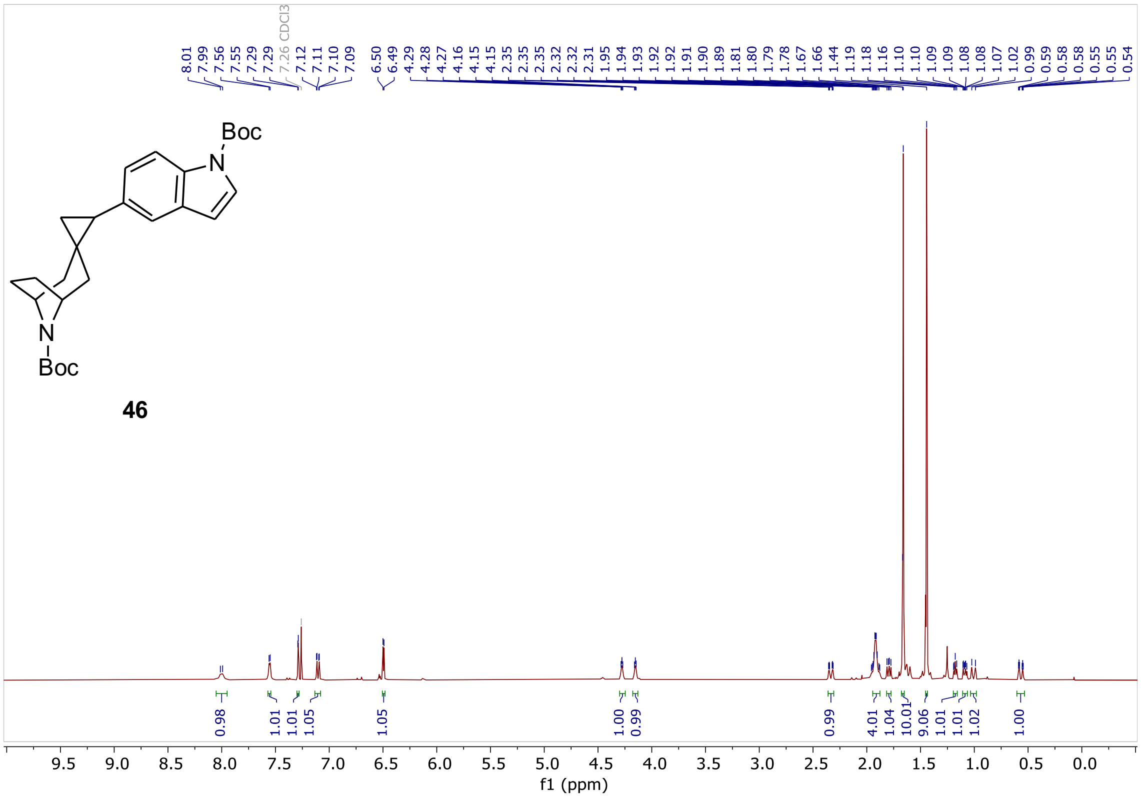

46

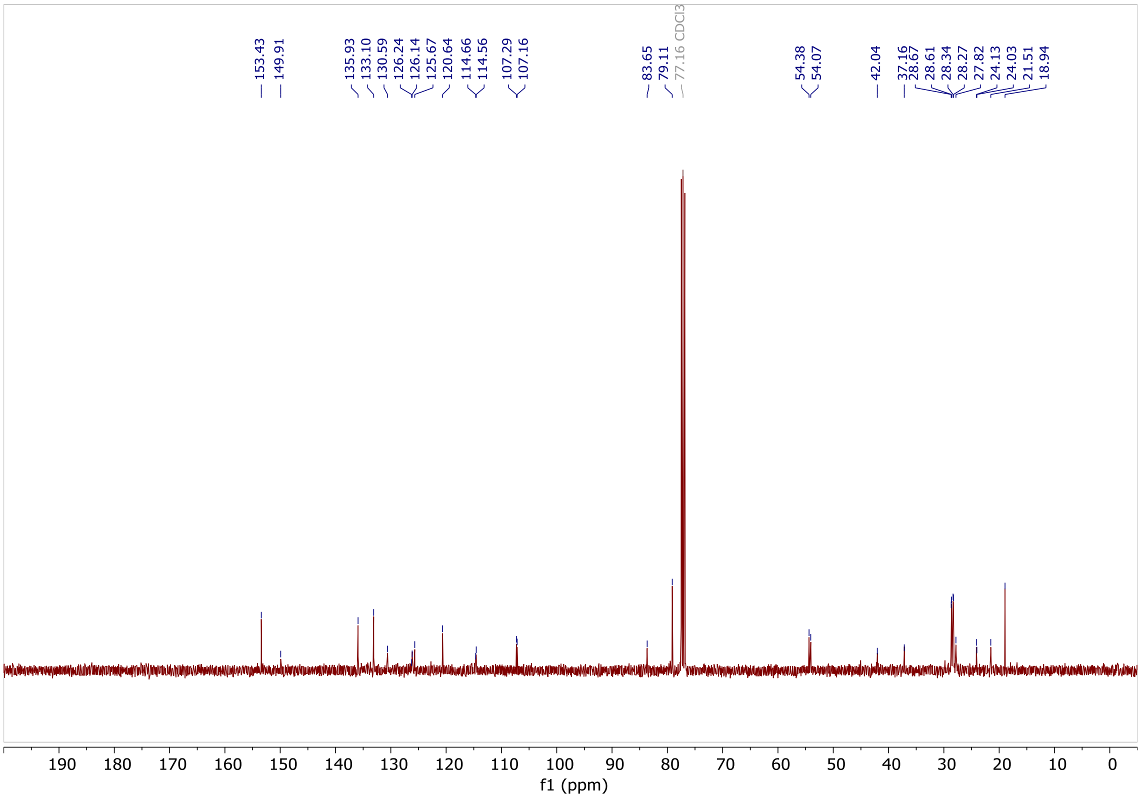

400 MHz ^{1}H NMR spectrum; 100.6 MHz ^{13}C NMR spectrum; $CDCl_3$ of **47**

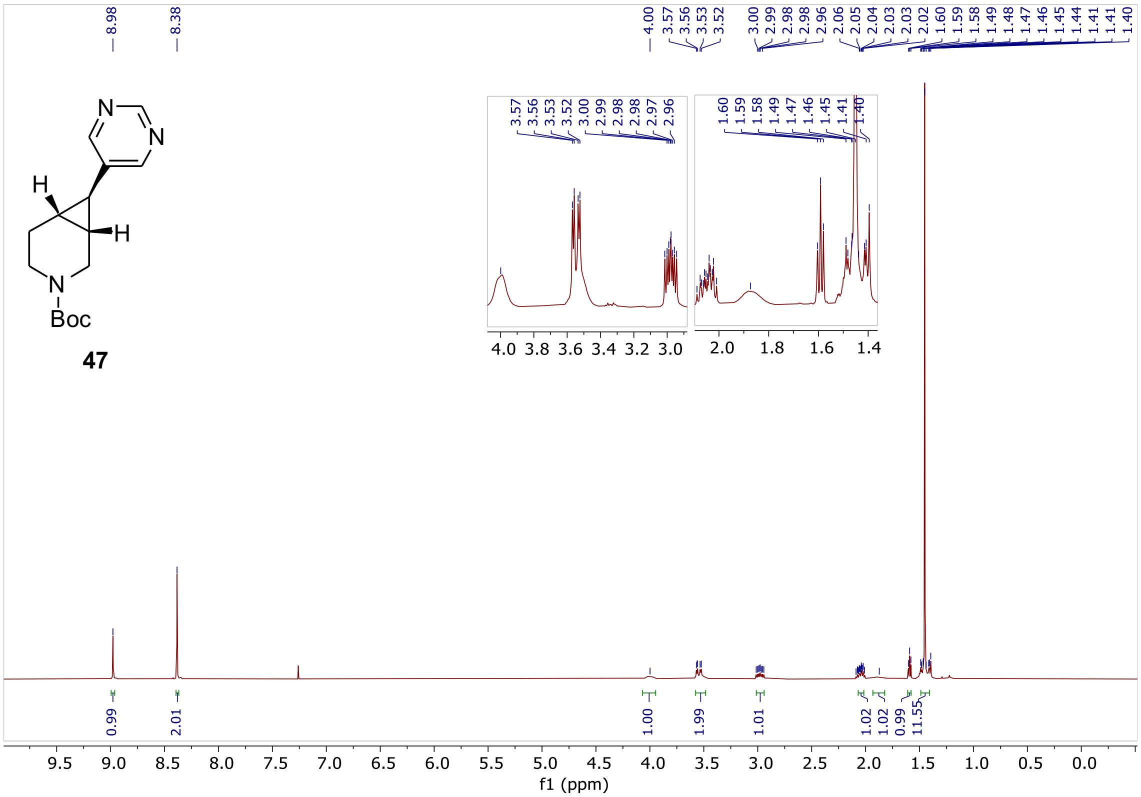

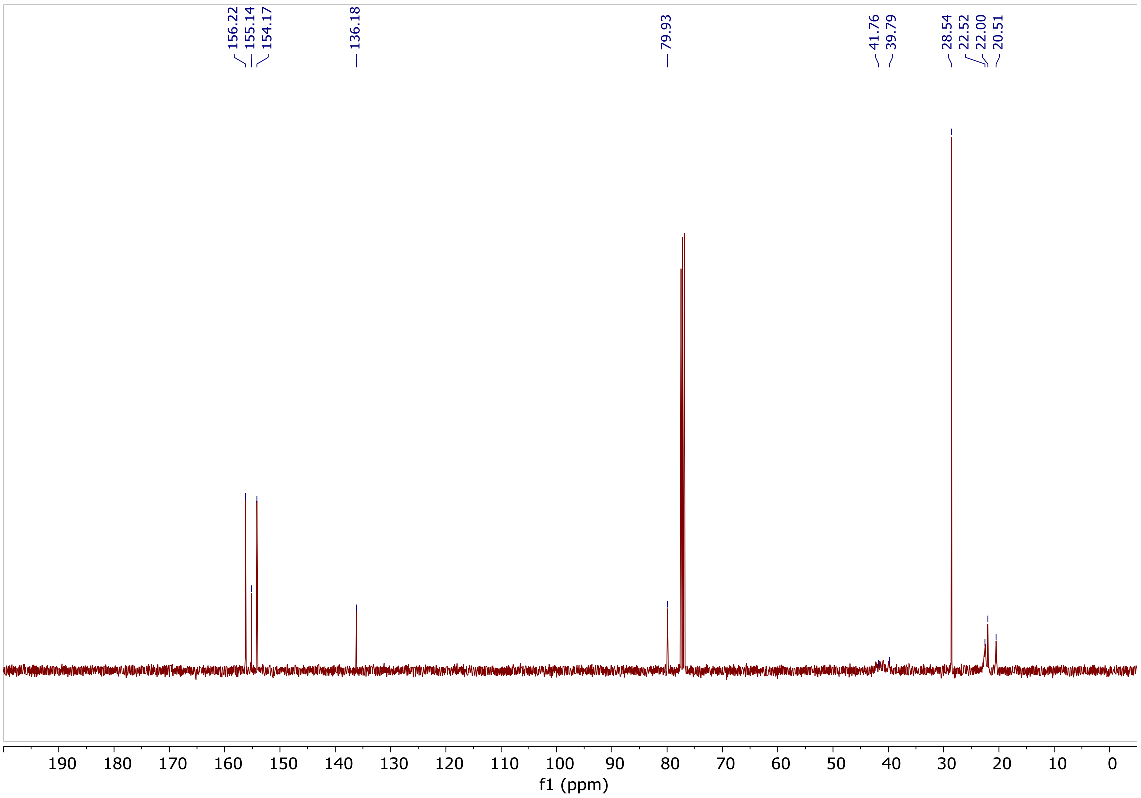

400 MHz ^{1}H NMR spectrum; 100.6 MHz ^{13}C NMR spectrum; $CDCl_3$ of **48**

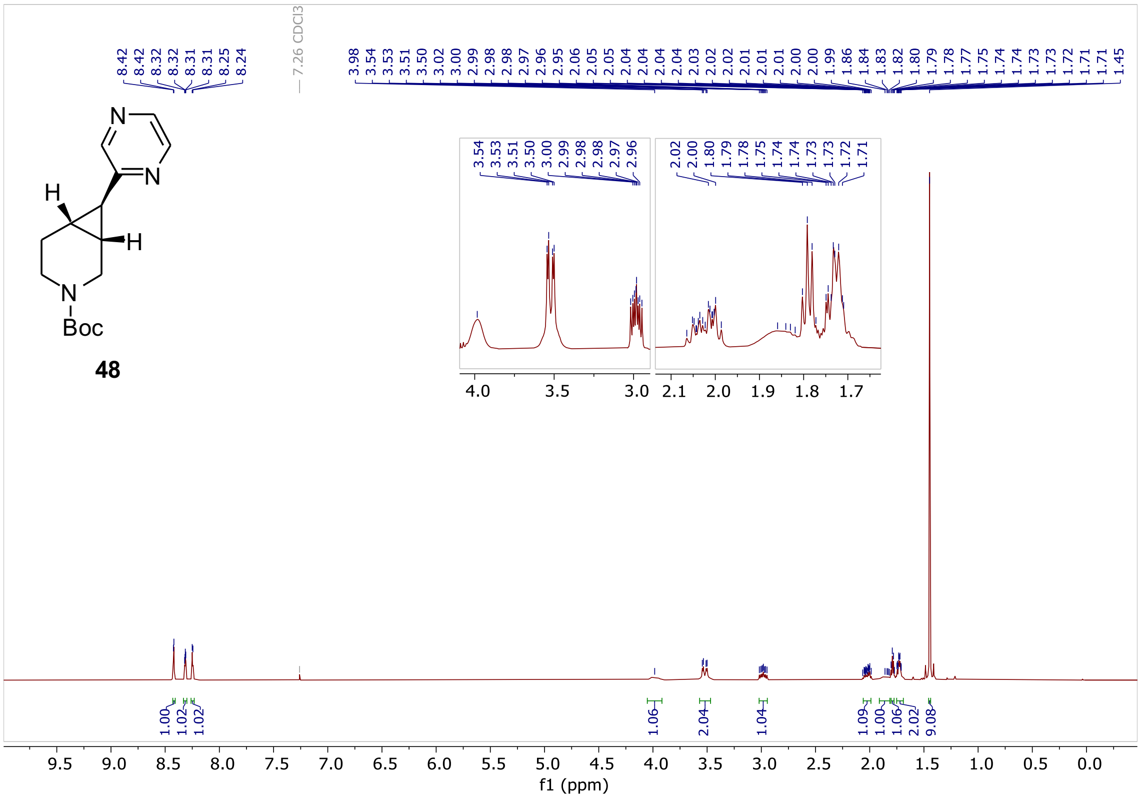

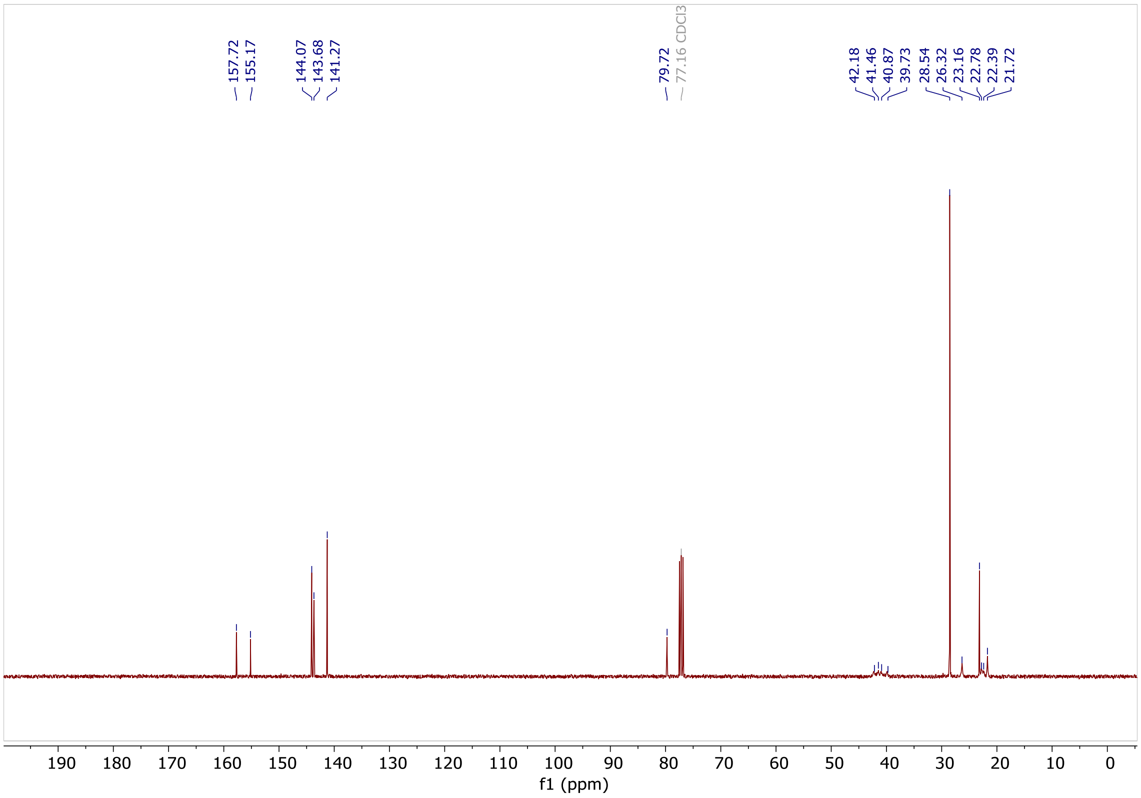

400 MHz ^{1}H NMR spectrum; 100.6 MHz ^{13}C NMR spectrum; $CDCl_3$ of **49**

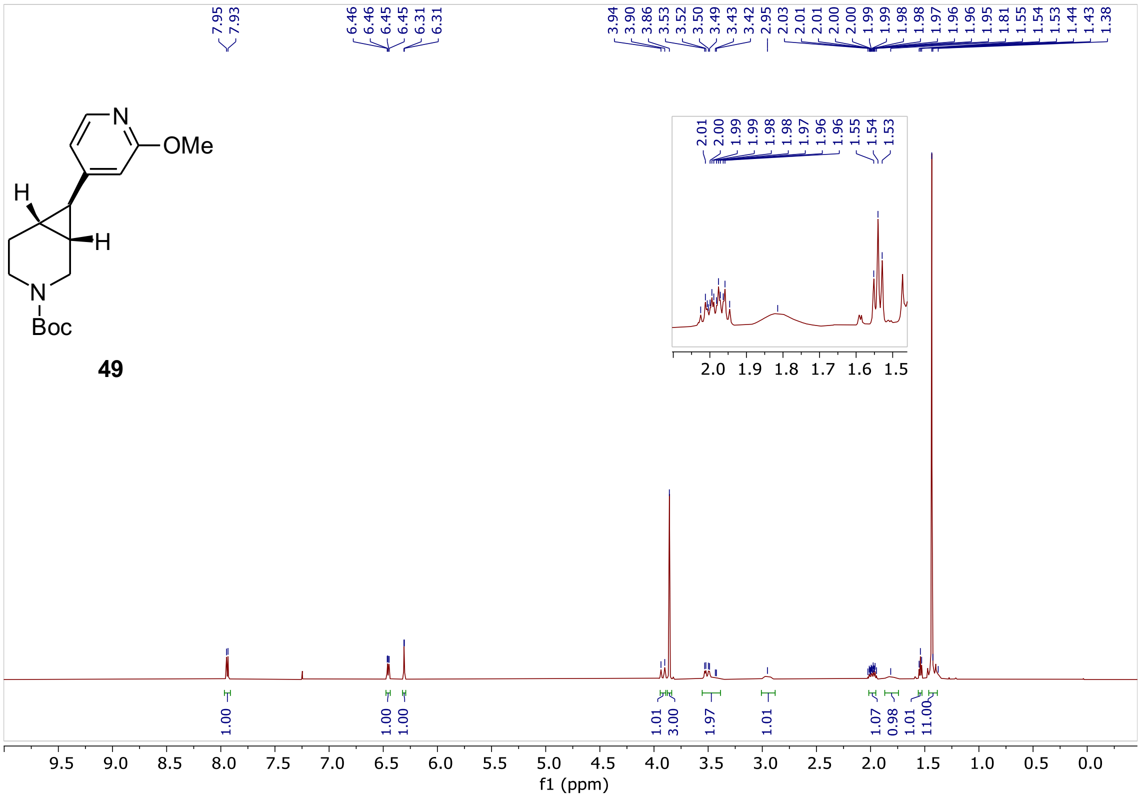

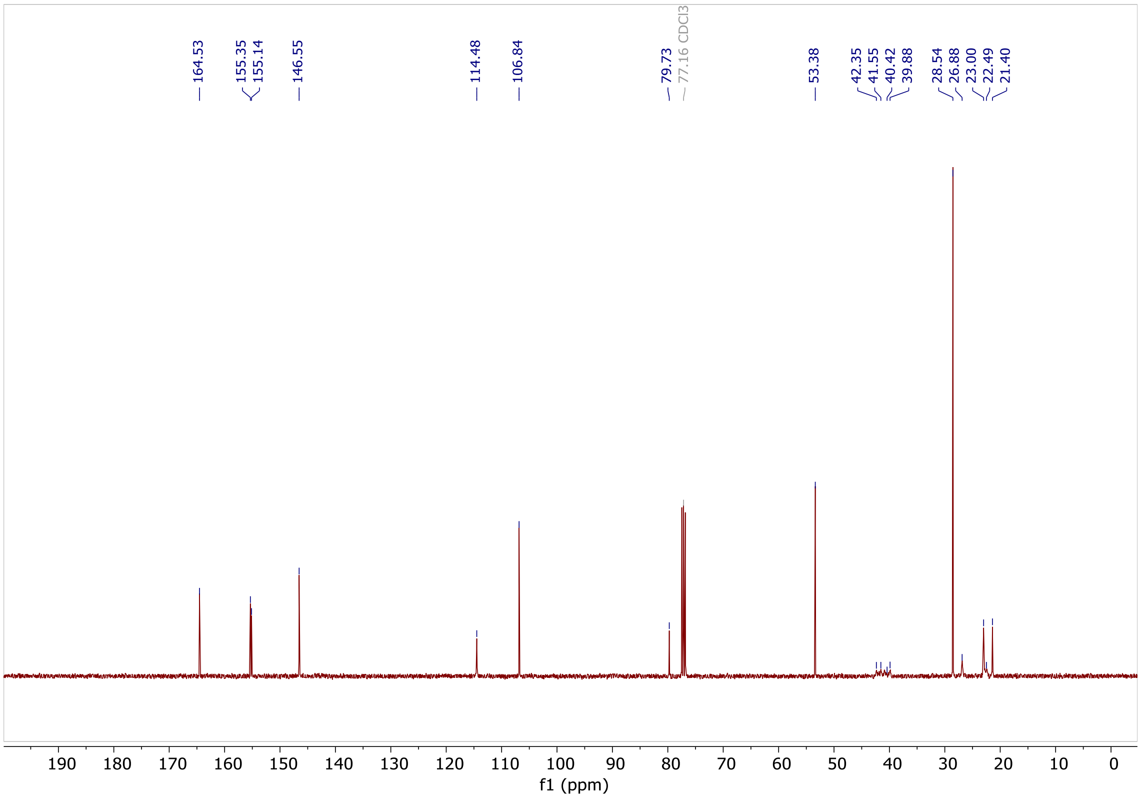

400 MHz ^{1}H NMR spectrum; 100.6 MHz ^{13}C NMR spectrum; $CDCl_3$ of **50**

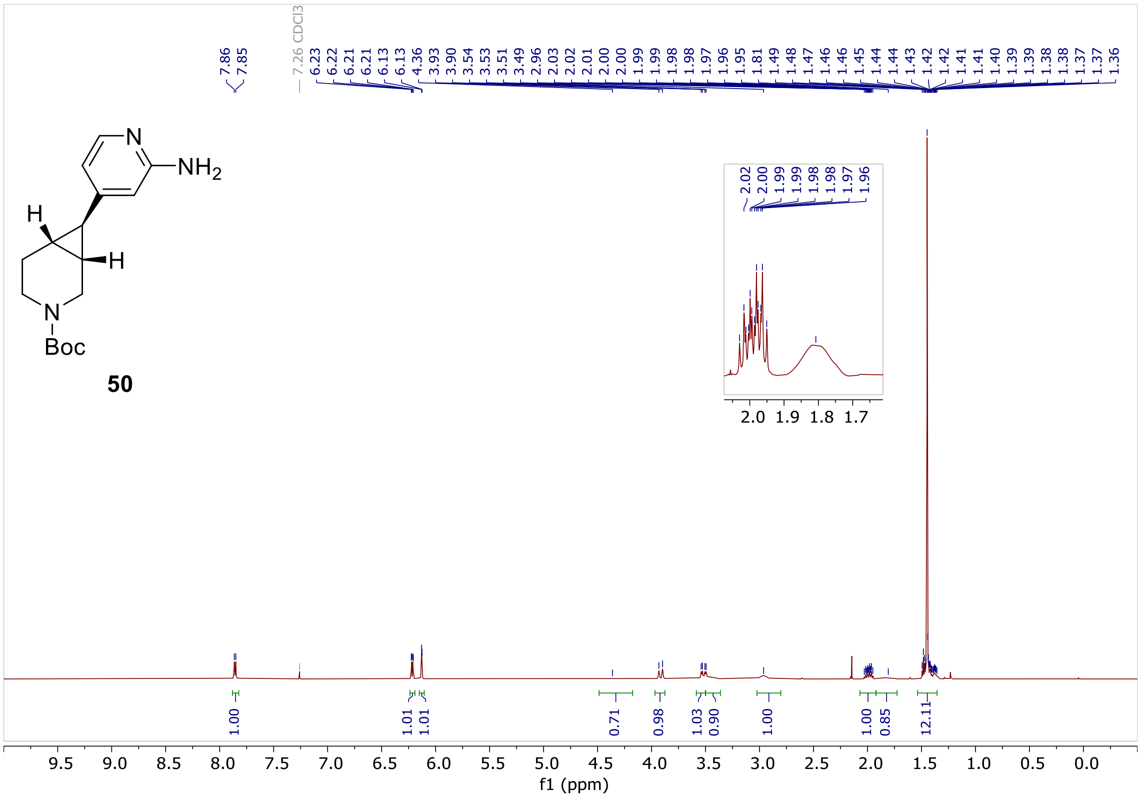

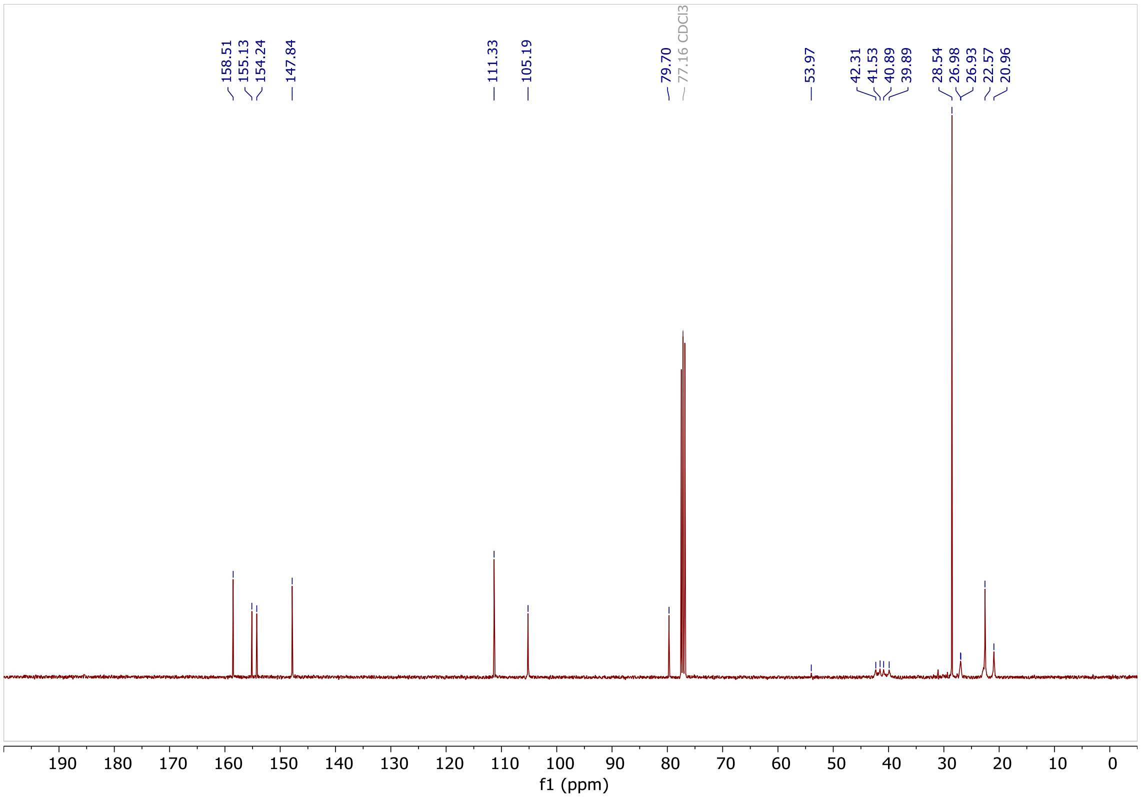

400 MHz ^{1}H NMR spectrum; 100.6 MHz ^{13}C NMR spectrum; $CDCl_3$ of **51**

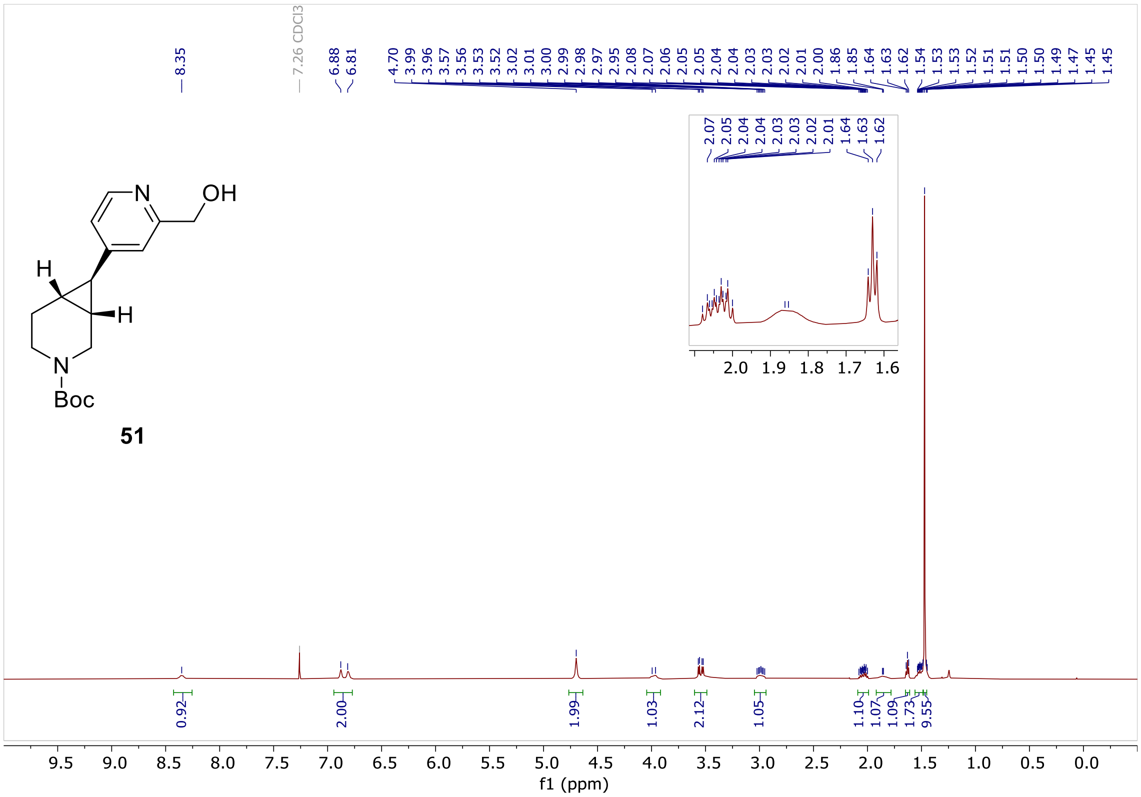

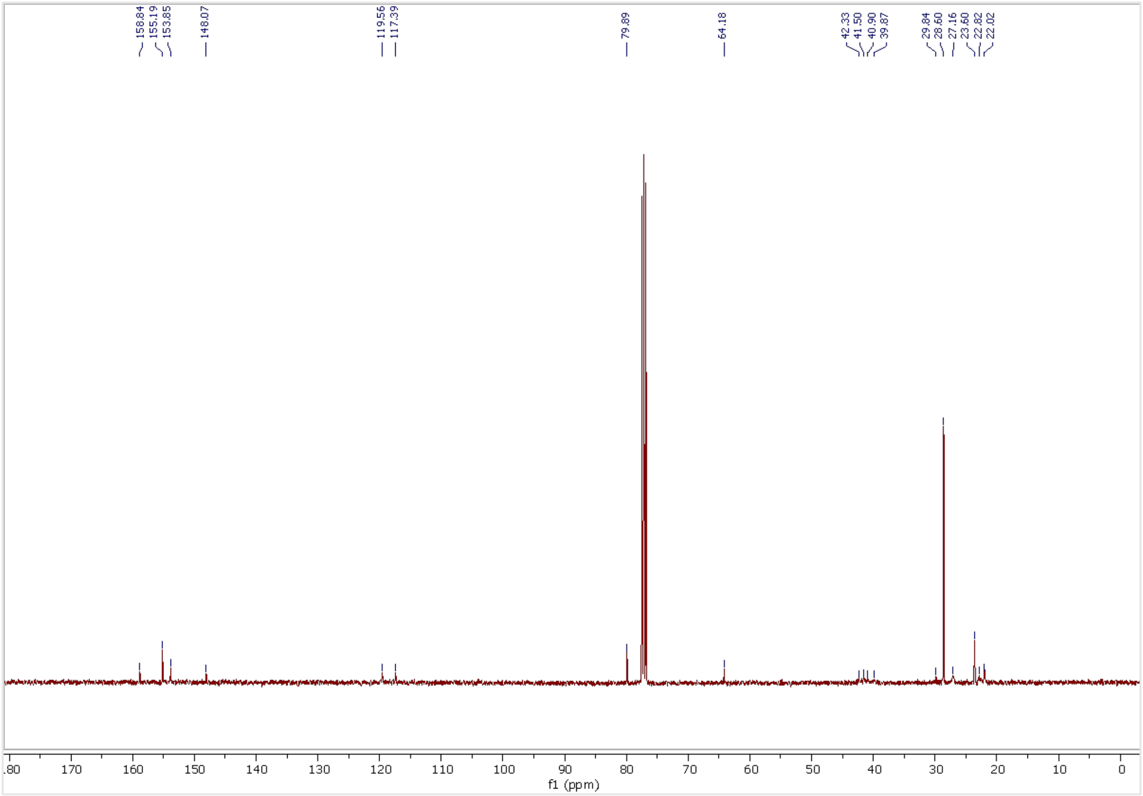

400 MHz ^{1}H NMR spectrum; 100.6 MHz ^{13}C NMR spectrum; $CDCl_3$ of **52**

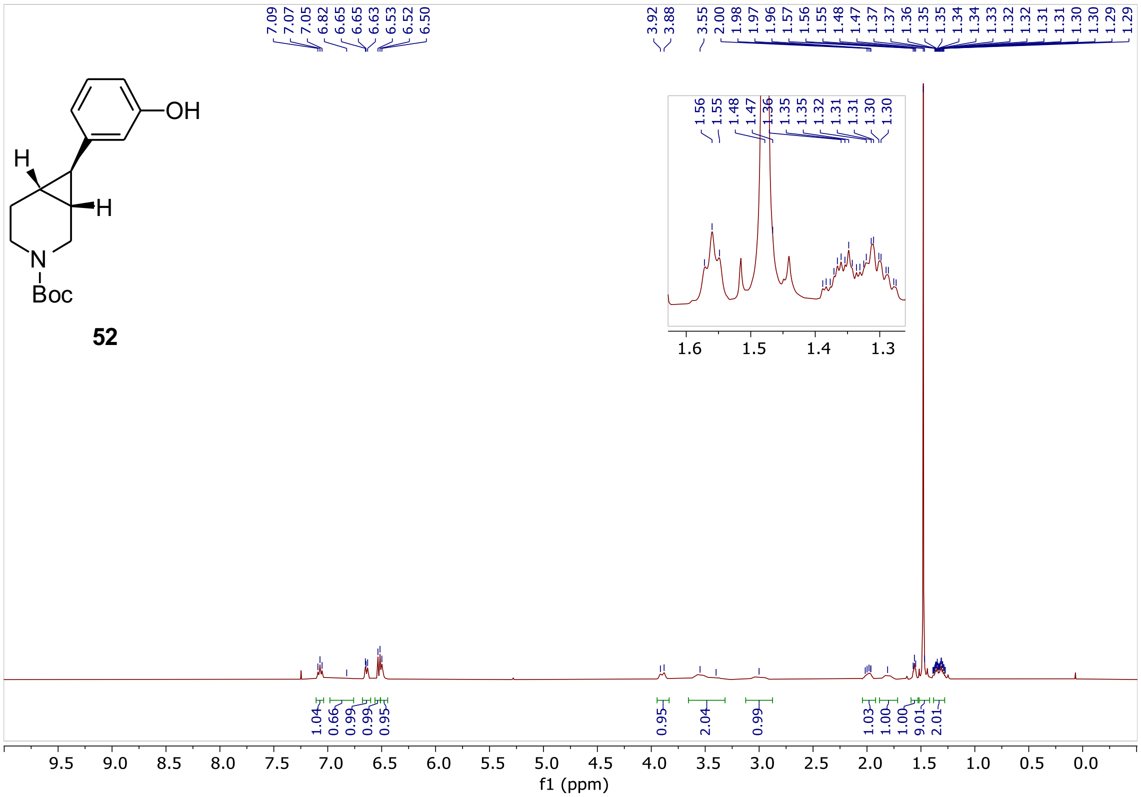

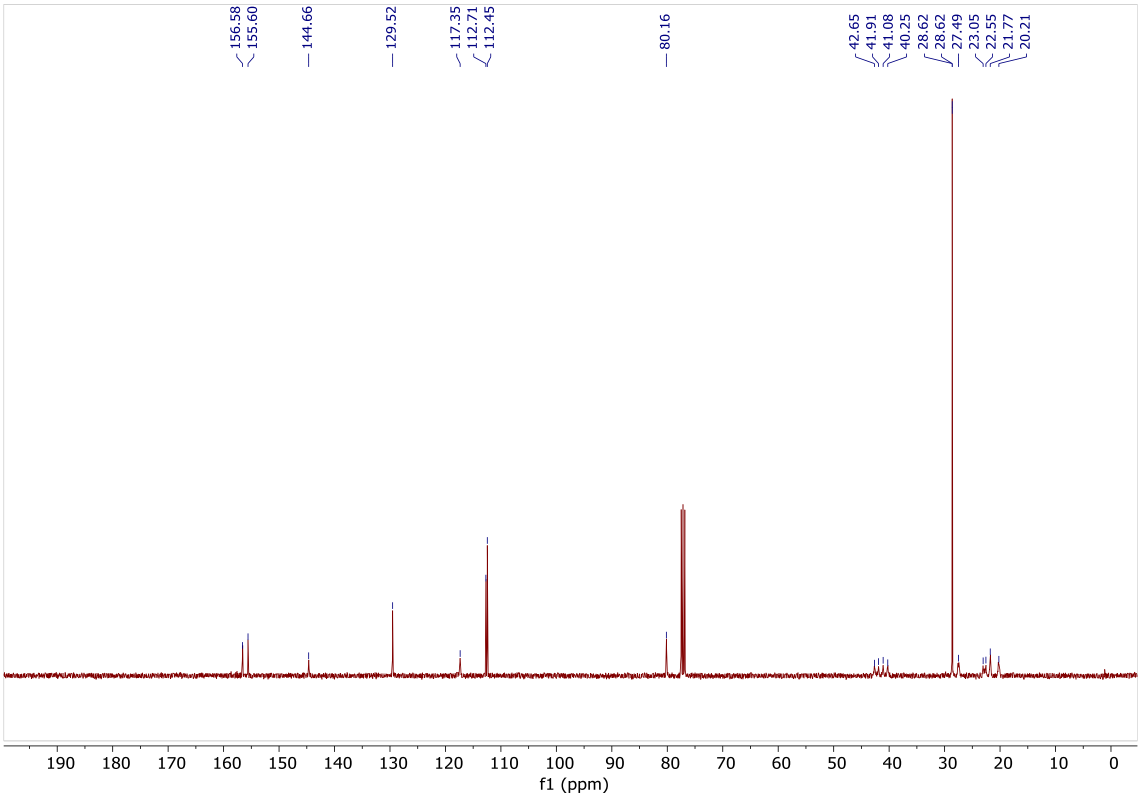

400 MHz ^{1}H NMR spectrum; 100.6 MHz ^{13}C NMR spectrum; $CDCl_3$ of **53**

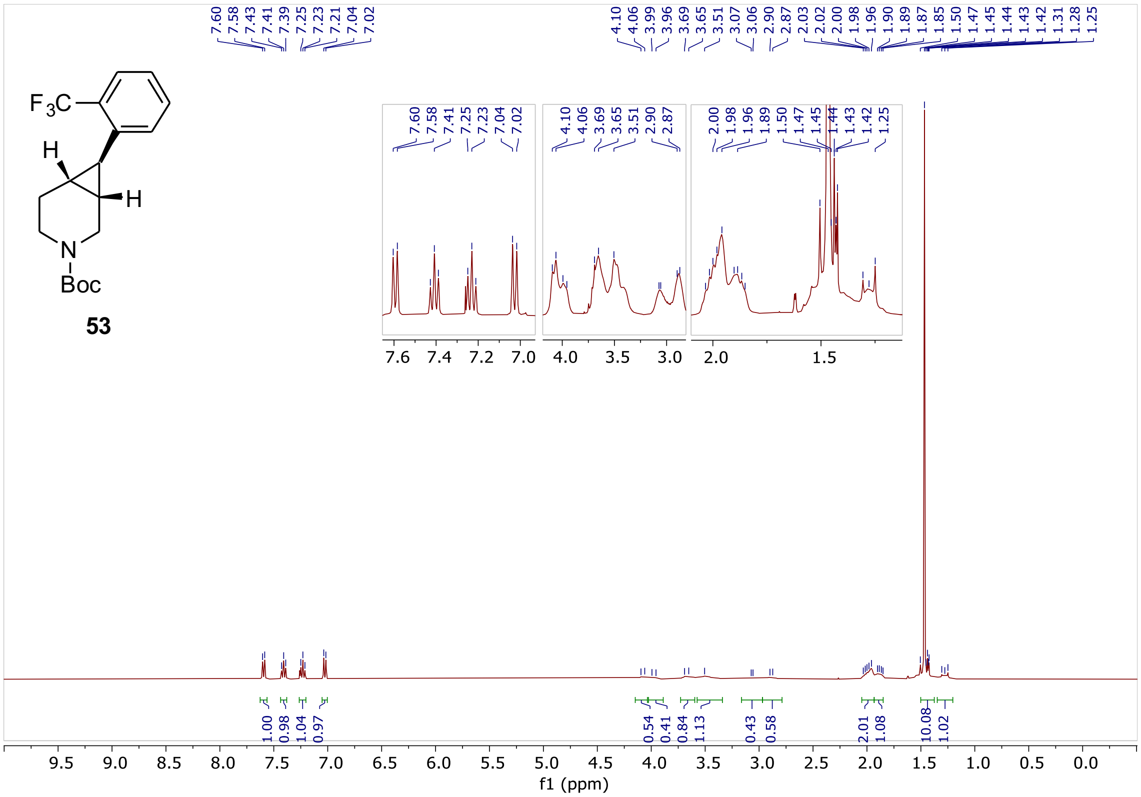

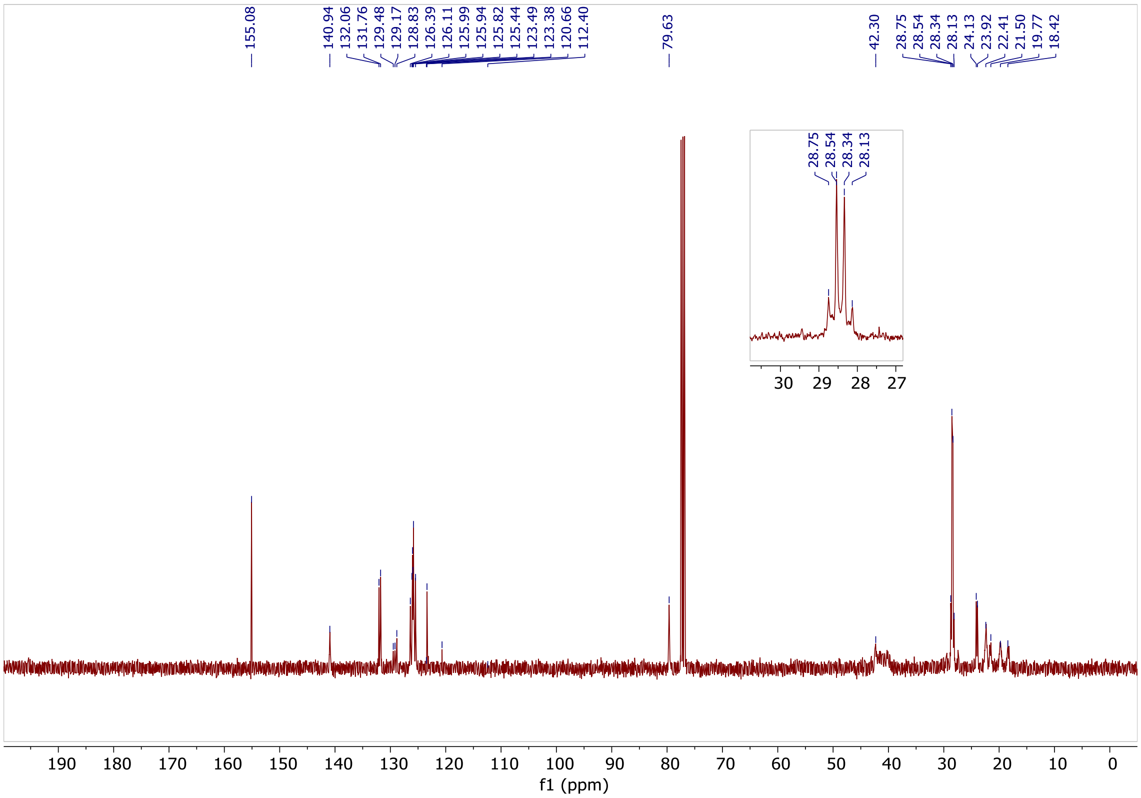

400 MHz ^{1}H NMR spectrum; 100.6 MHz ^{13}C NMR spectrum; $CDCl_3$ of **54**

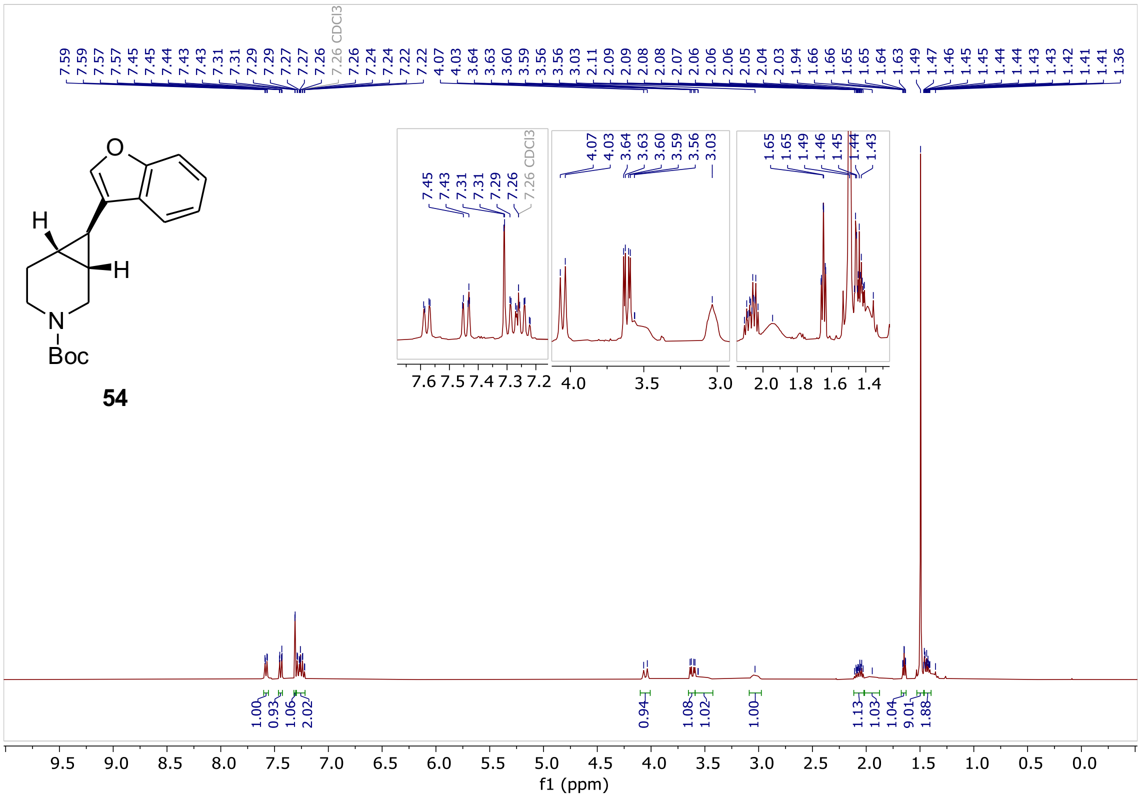

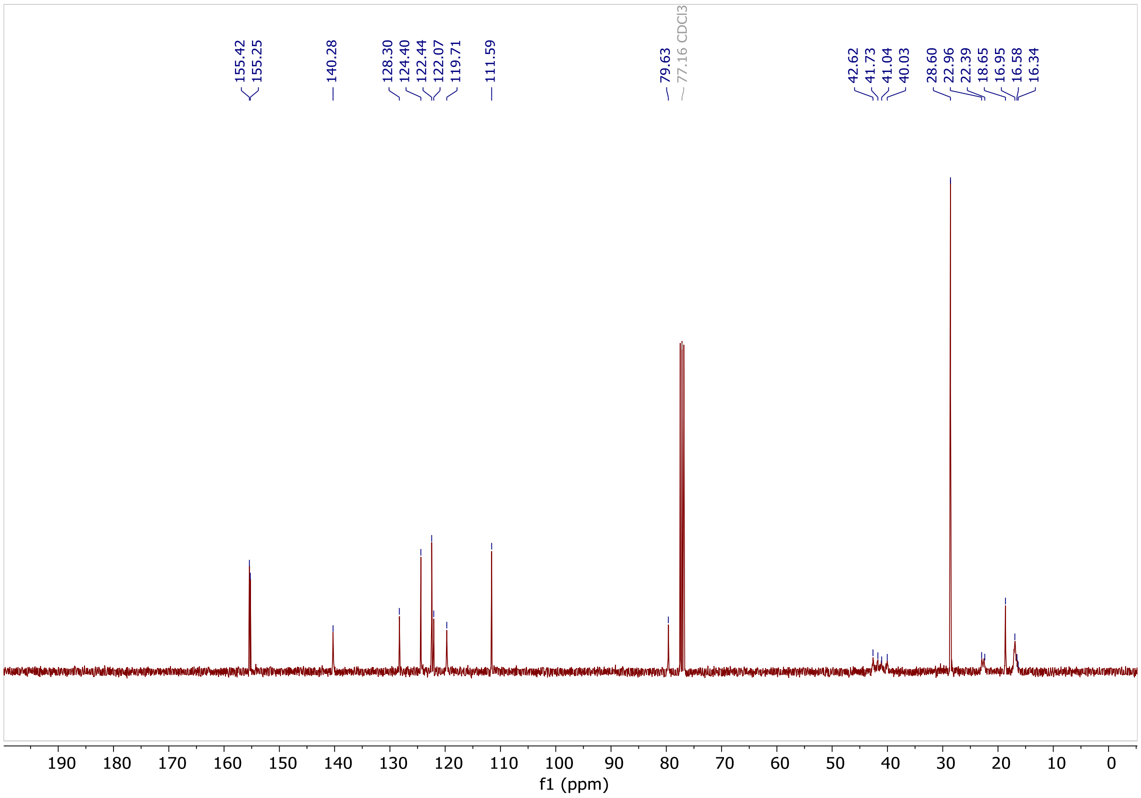

400 MHz ^{1}H NMR spectrum; 100.6 MHz ^{13}C NMR spectrum; $CDCl_3$ of **55**

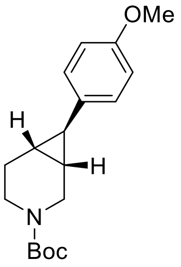

55

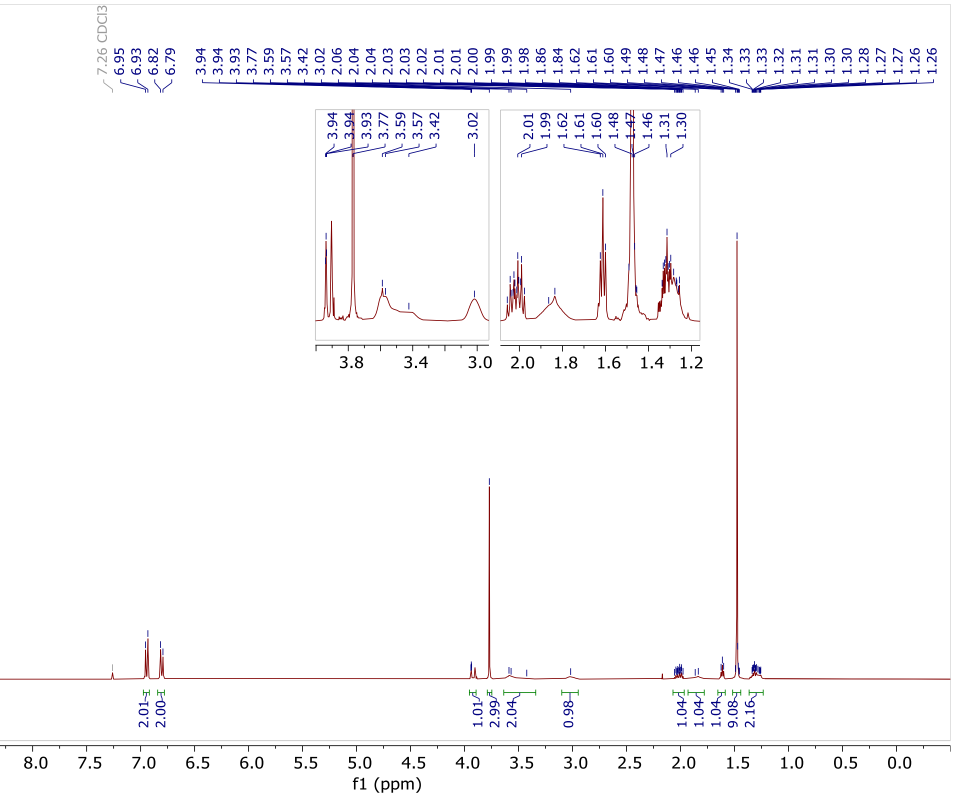

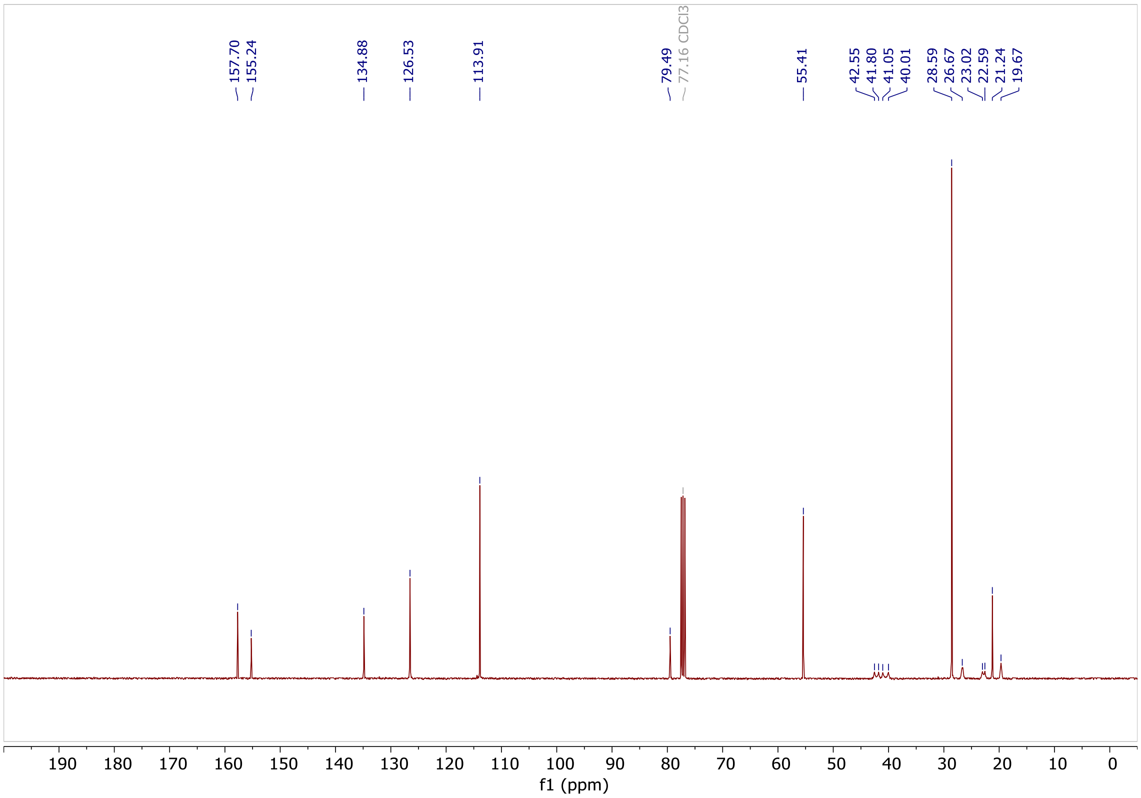

400 MHz ^{1}H NMR spectrum; 100.6 MHz ^{13}C NMR spectrum; $CDCl_3$ of **56**

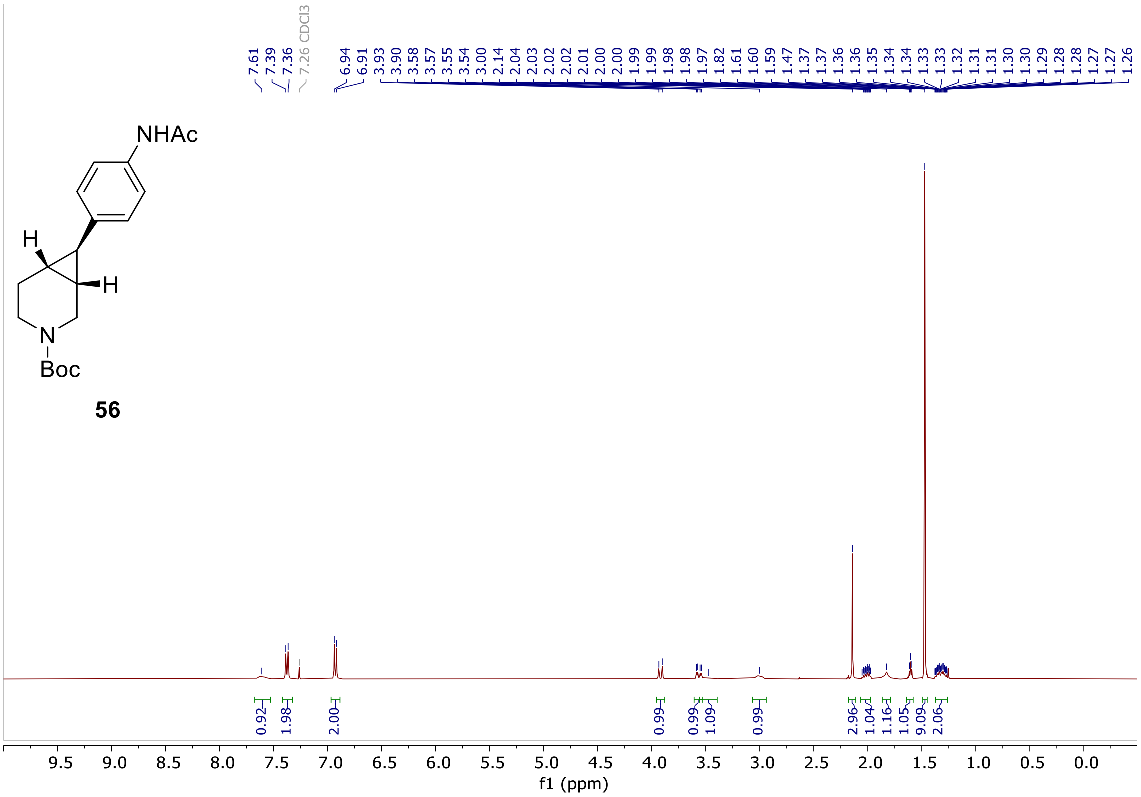

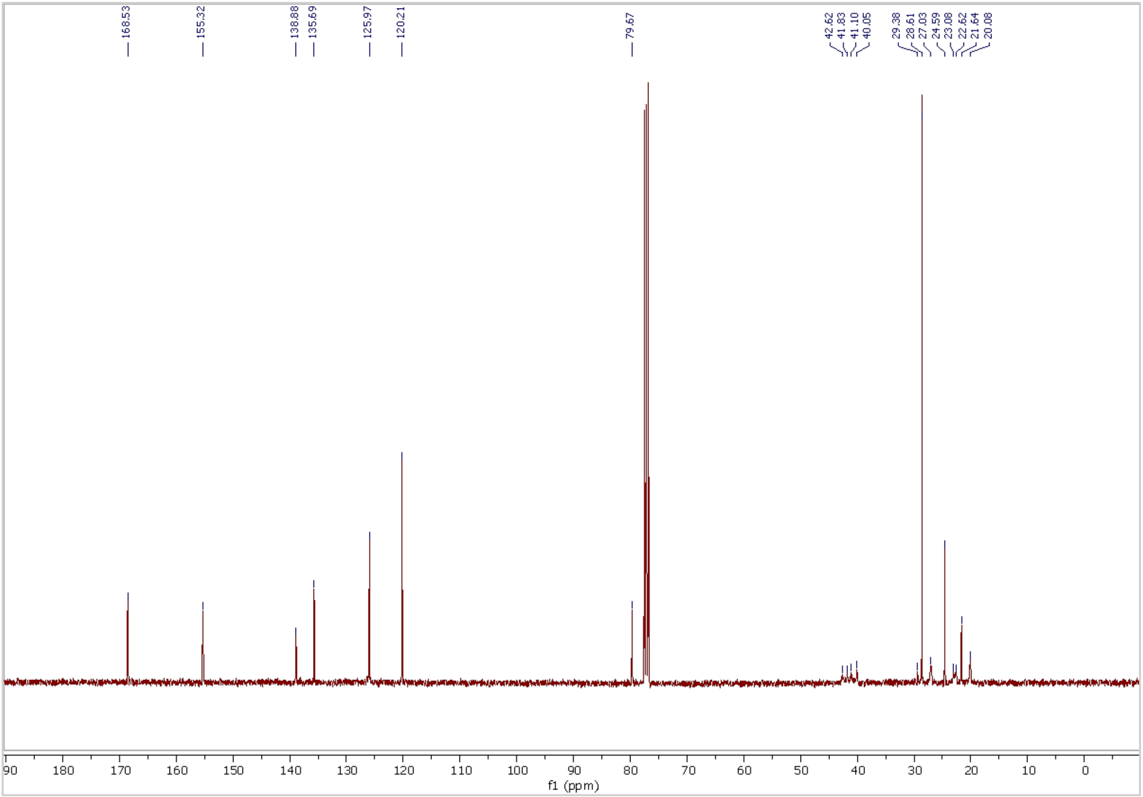

400 MHz ^{1}H NMR spectrum; 100.6 MHz ^{13}C NMR spectrum; $CDCl_3$ of **57**

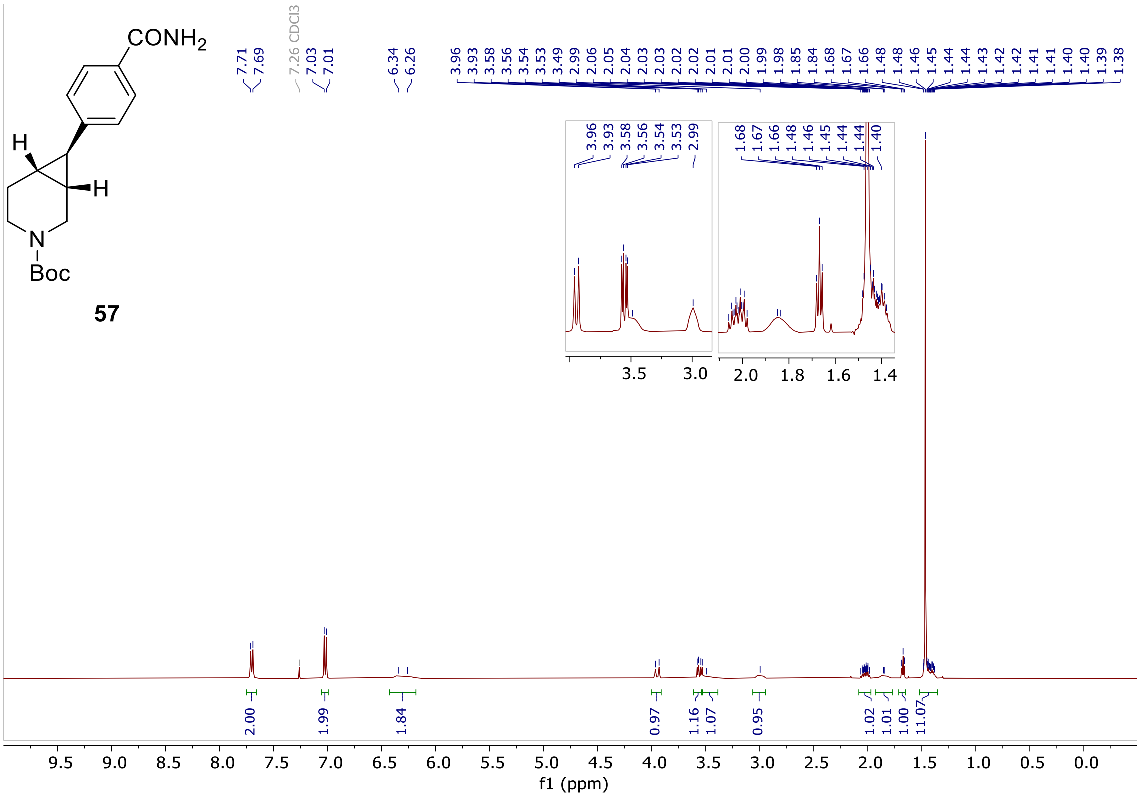

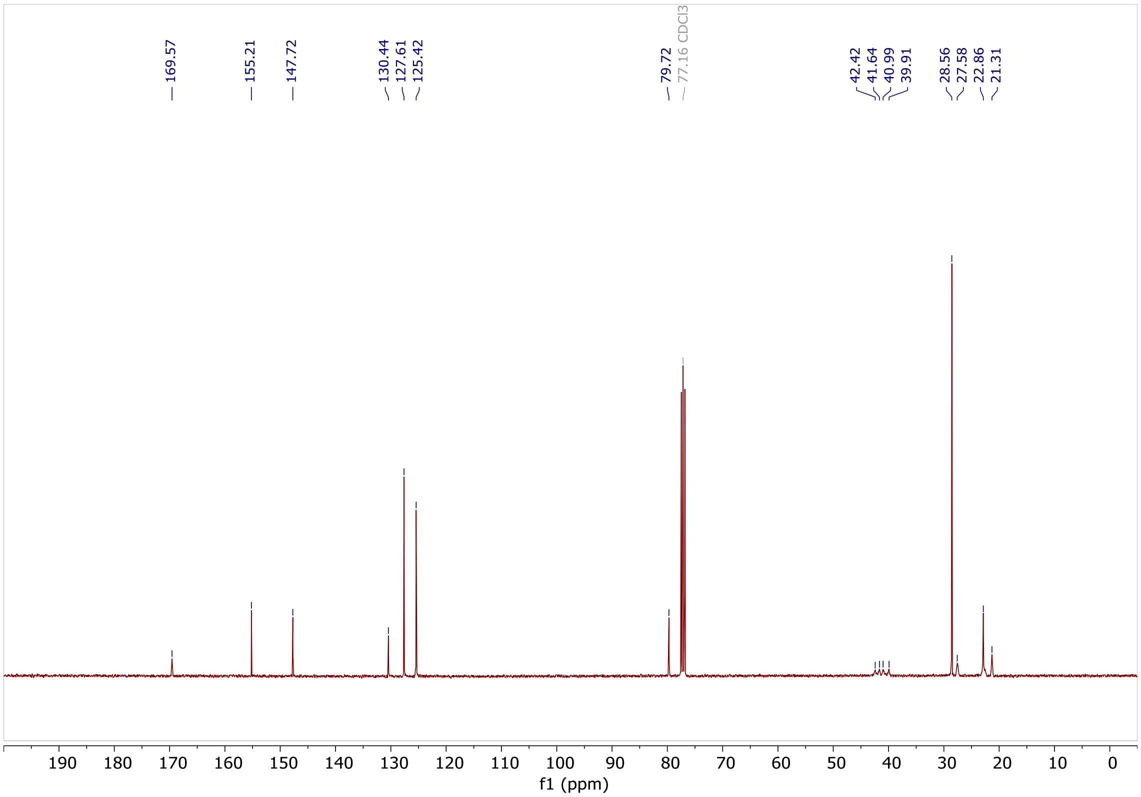

400 MHz ^{1}H NMR spectrum; 100.6 MHz ^{13}C NMR spectrum; $CDCl_3$ of **58**

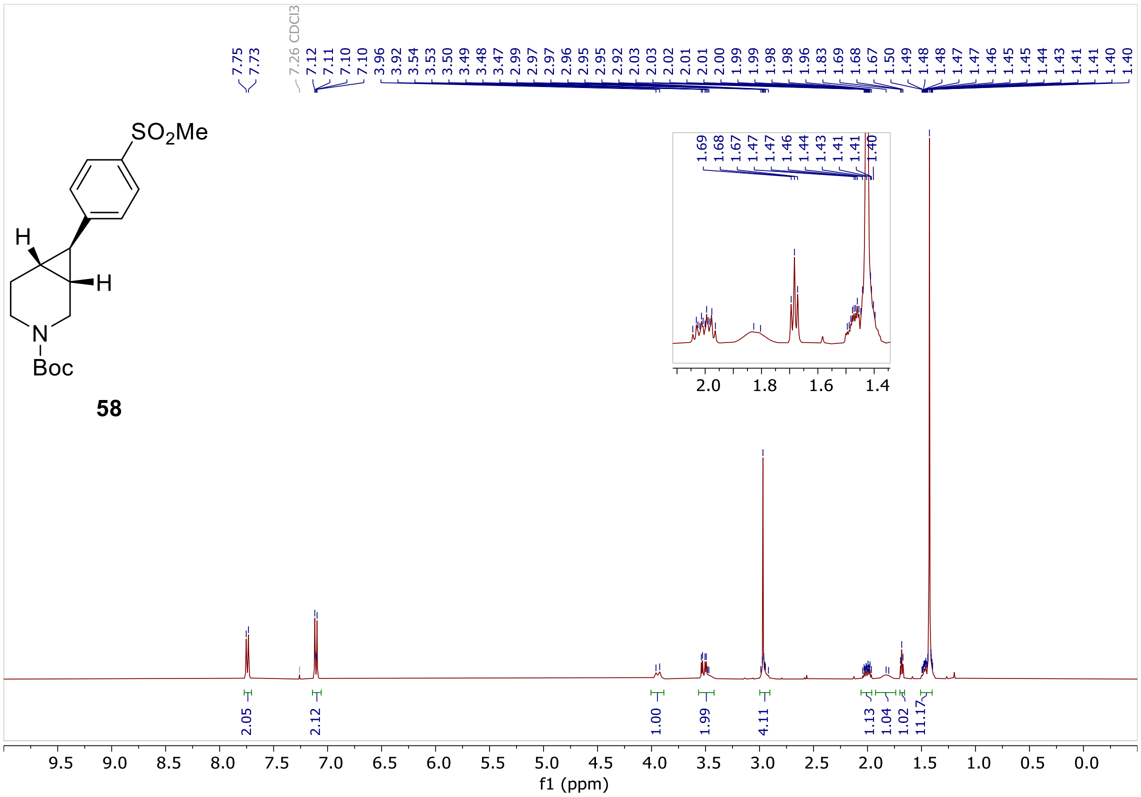

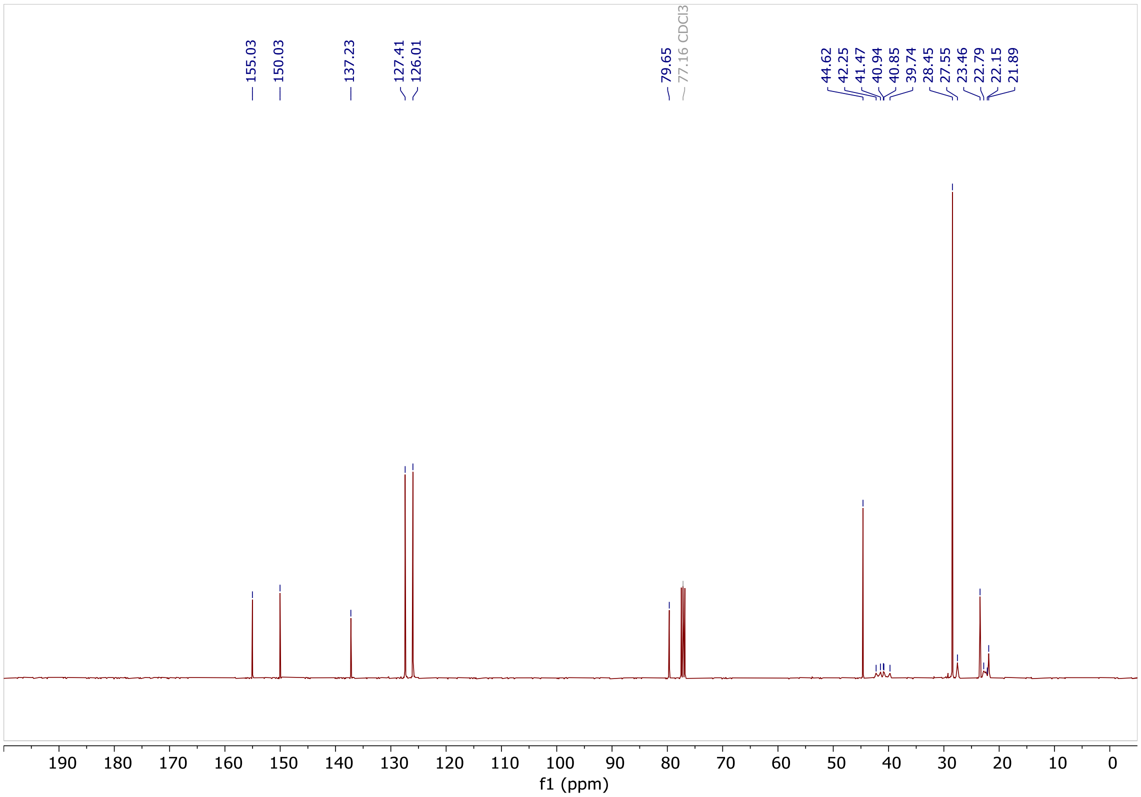

400 MHz ^{1}H NMR spectrum; 100.6 MHz ^{13}C NMR spectrum; $CDCl_3$ of **59**

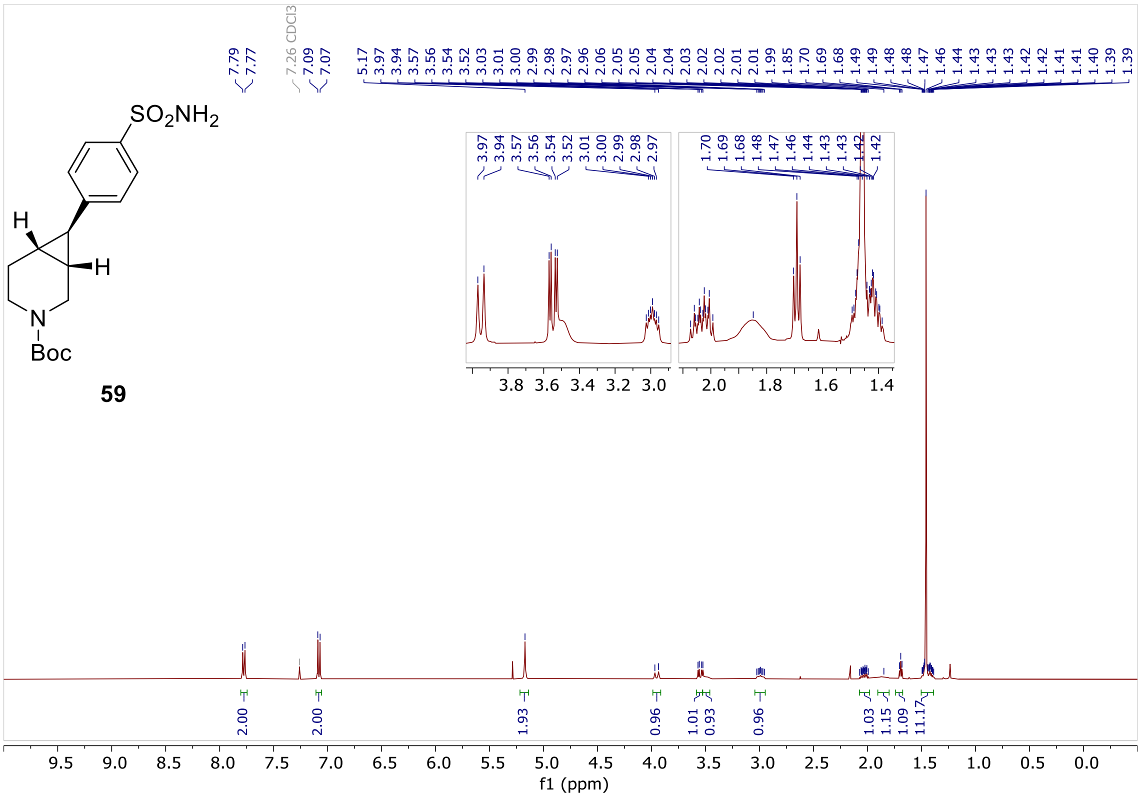

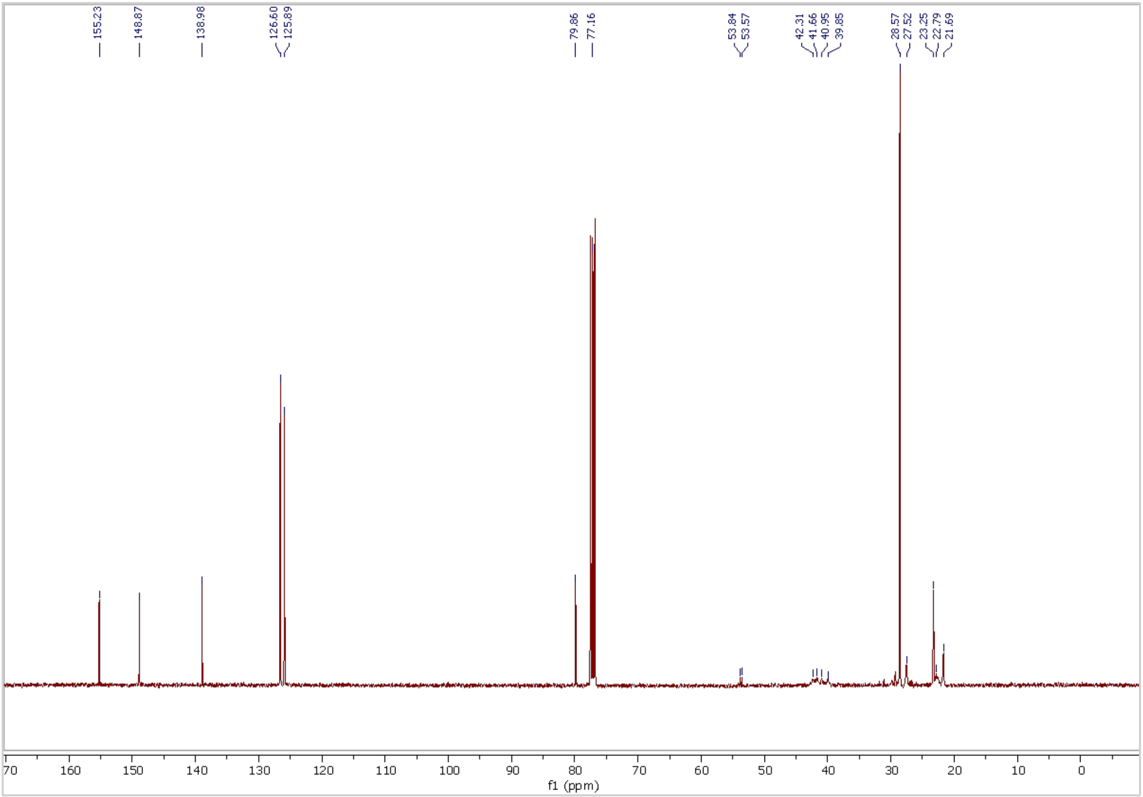

400 MHz ^{1}H NMR spectrum; 100.6 MHz ^{13}C NMR spectrum; $CDCl_3$ of **60**

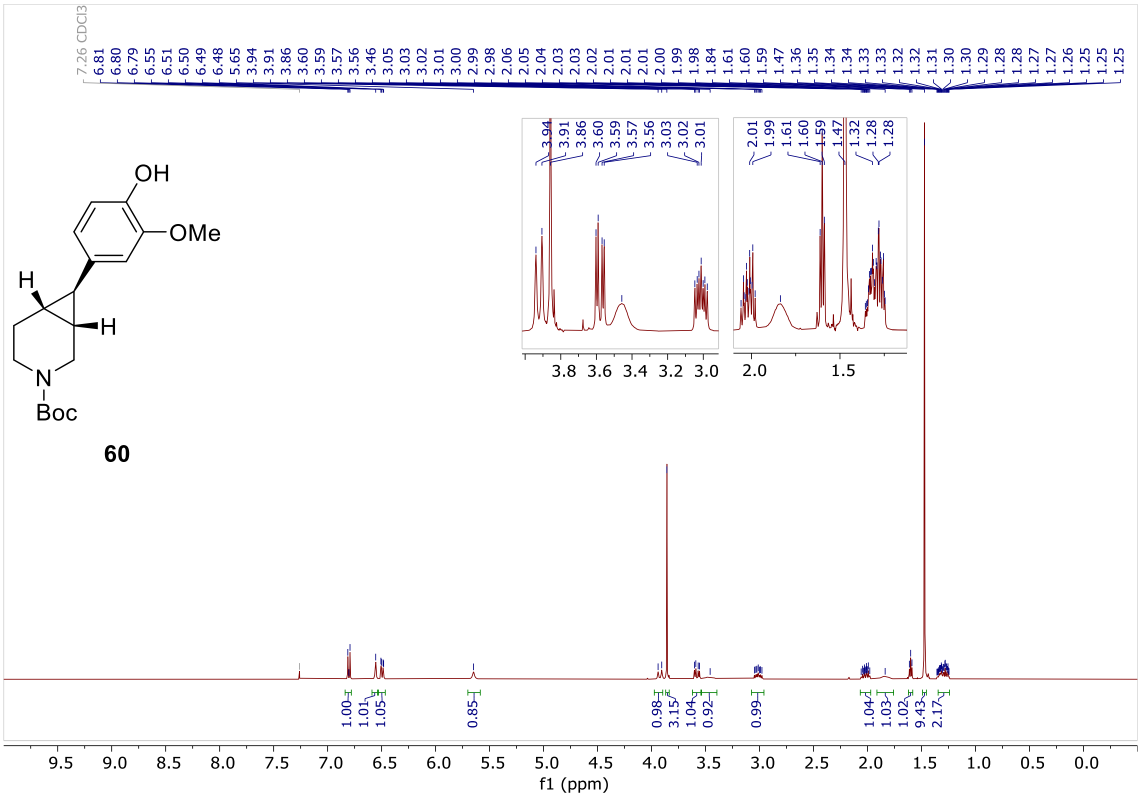

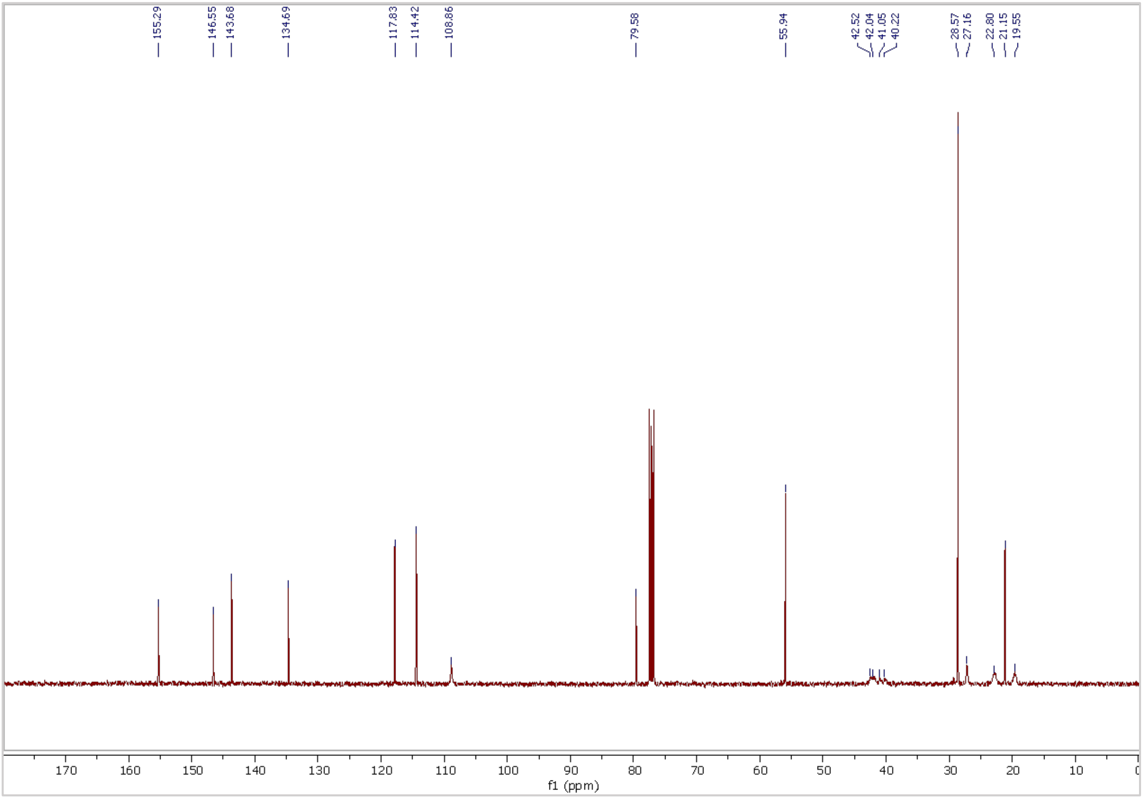

400 MHz ^{1}H NMR spectrum; 100.6 MHz ^{13}C NMR spectrum; $CDCl_3$ of **61**

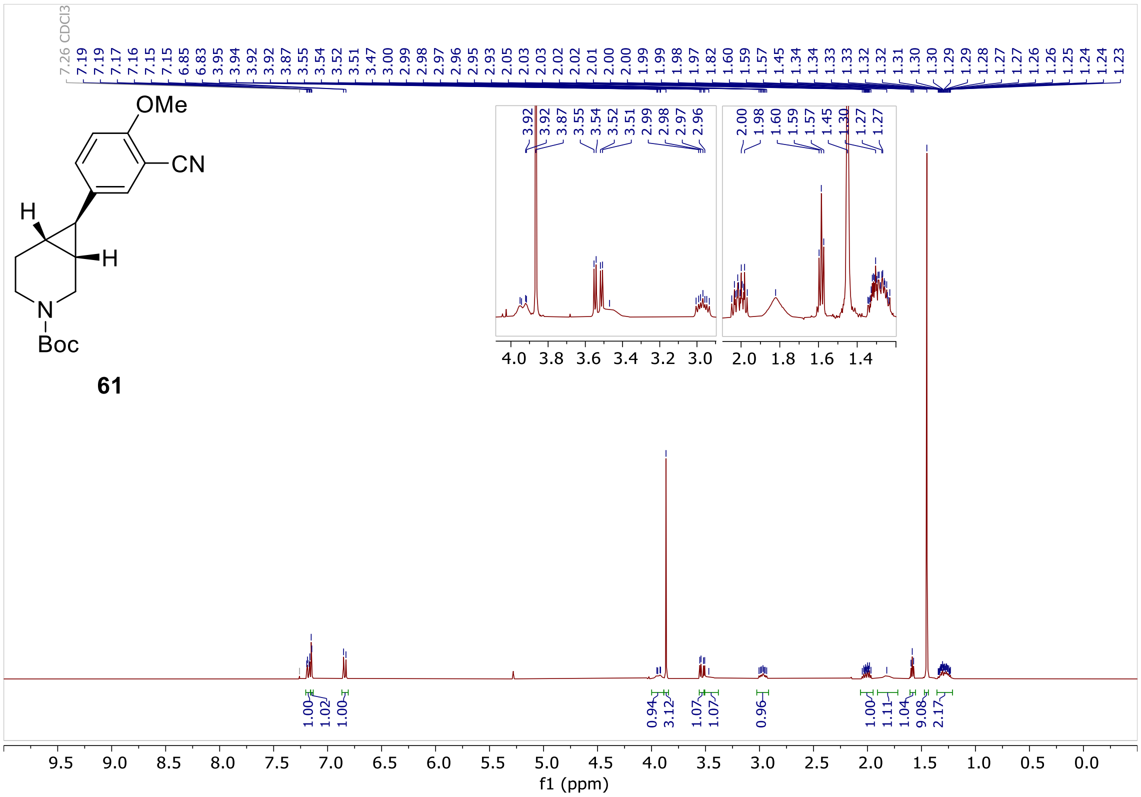

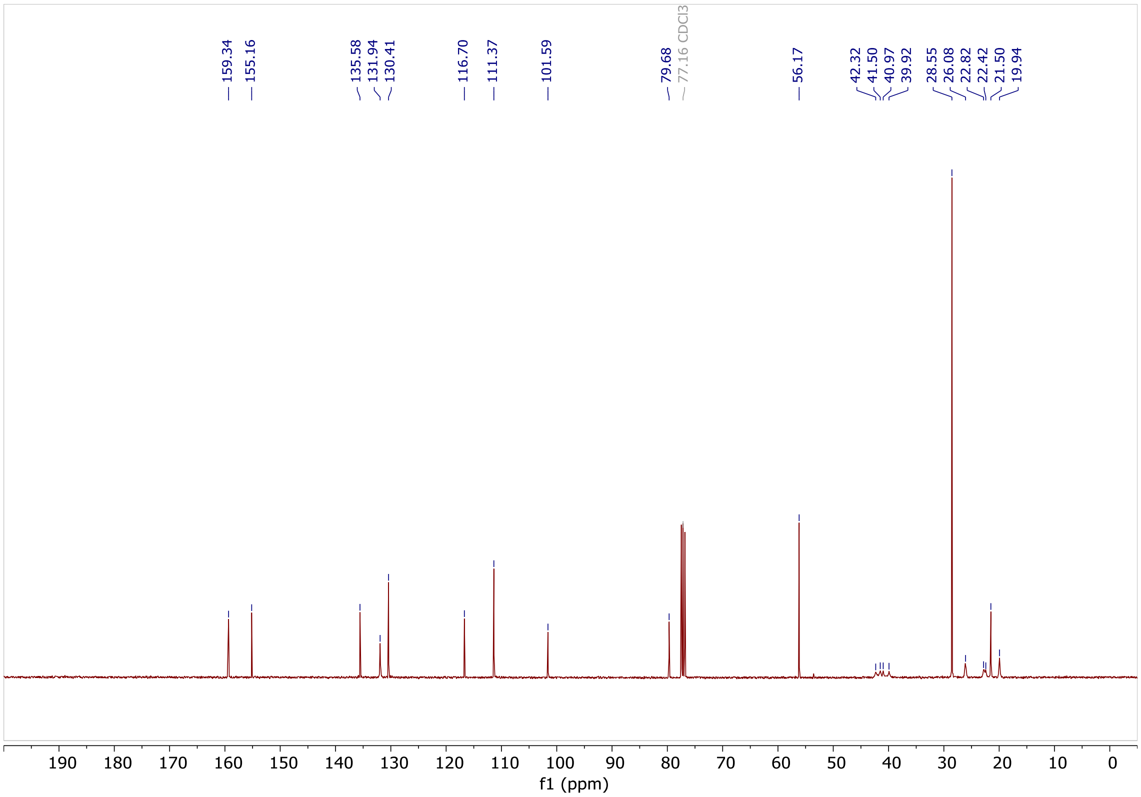

400 MHz ^{1}H NMR spectrum; 100.6 MHz ^{13}C NMR spectrum; $CDCl_3$ of **62**

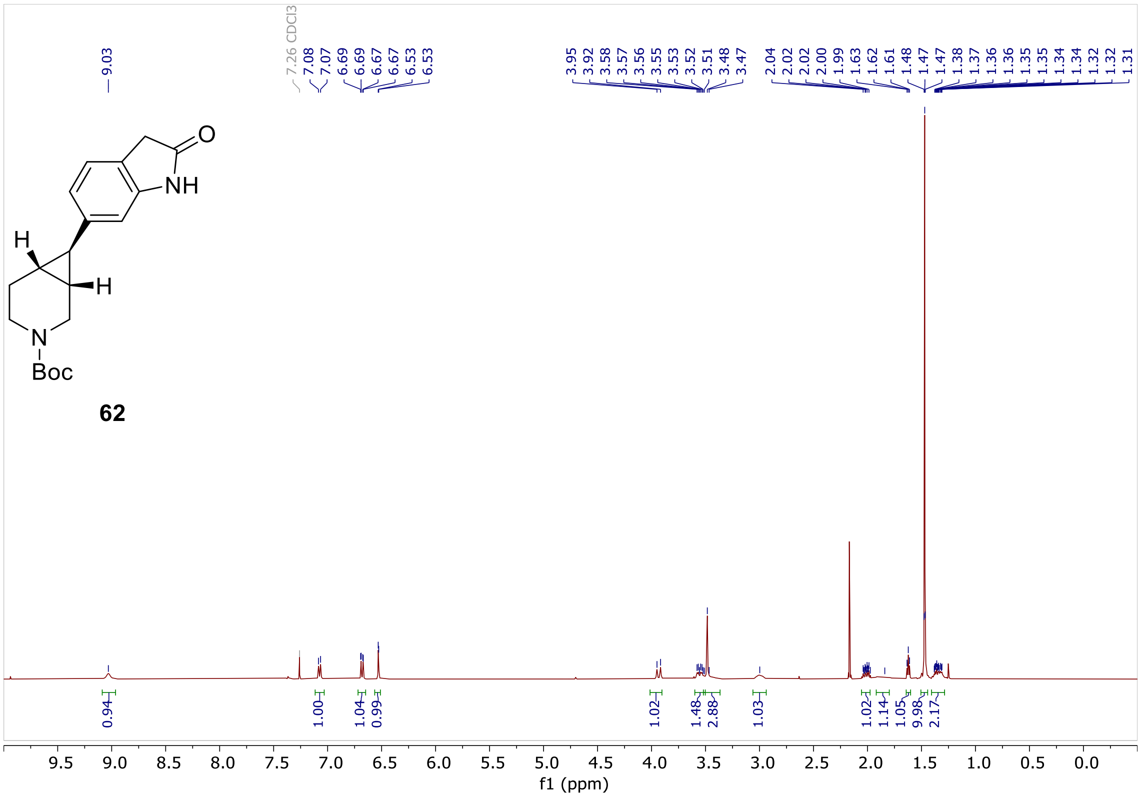

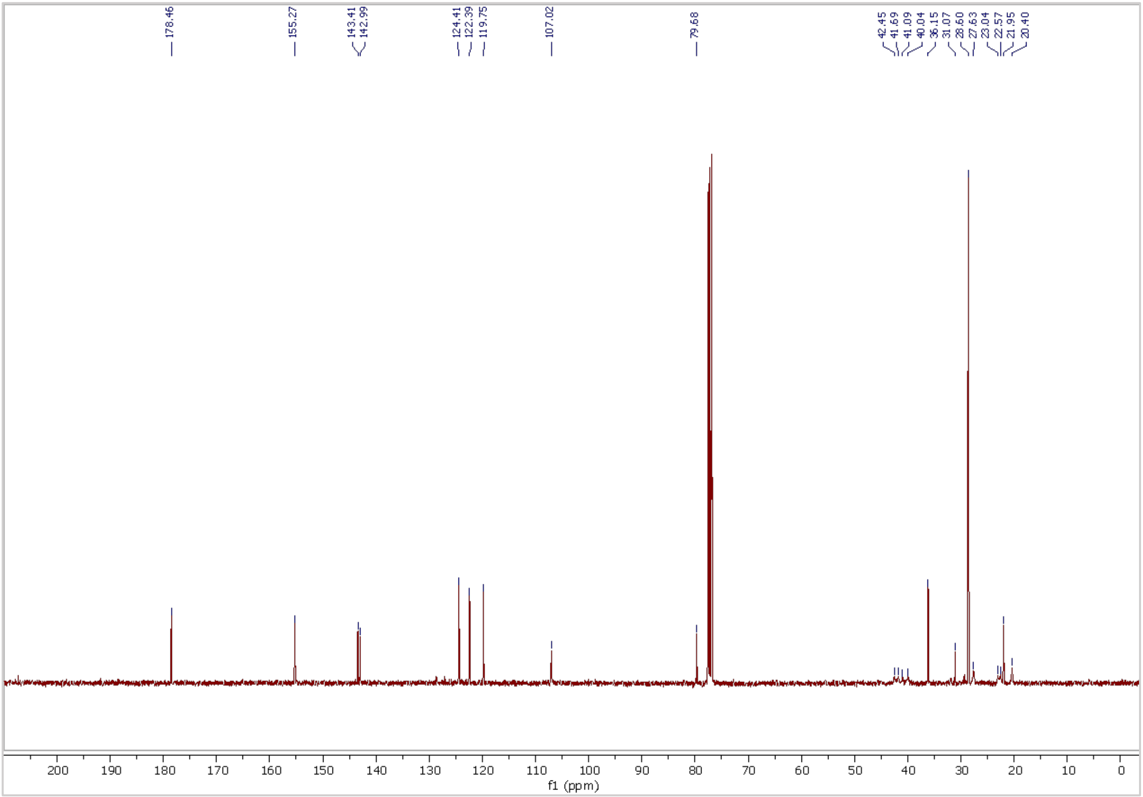

400 MHz ^{1}H NMR spectrum; 100.6 MHz ^{13}C NMR spectrum; $CDCl_3$ of **63**

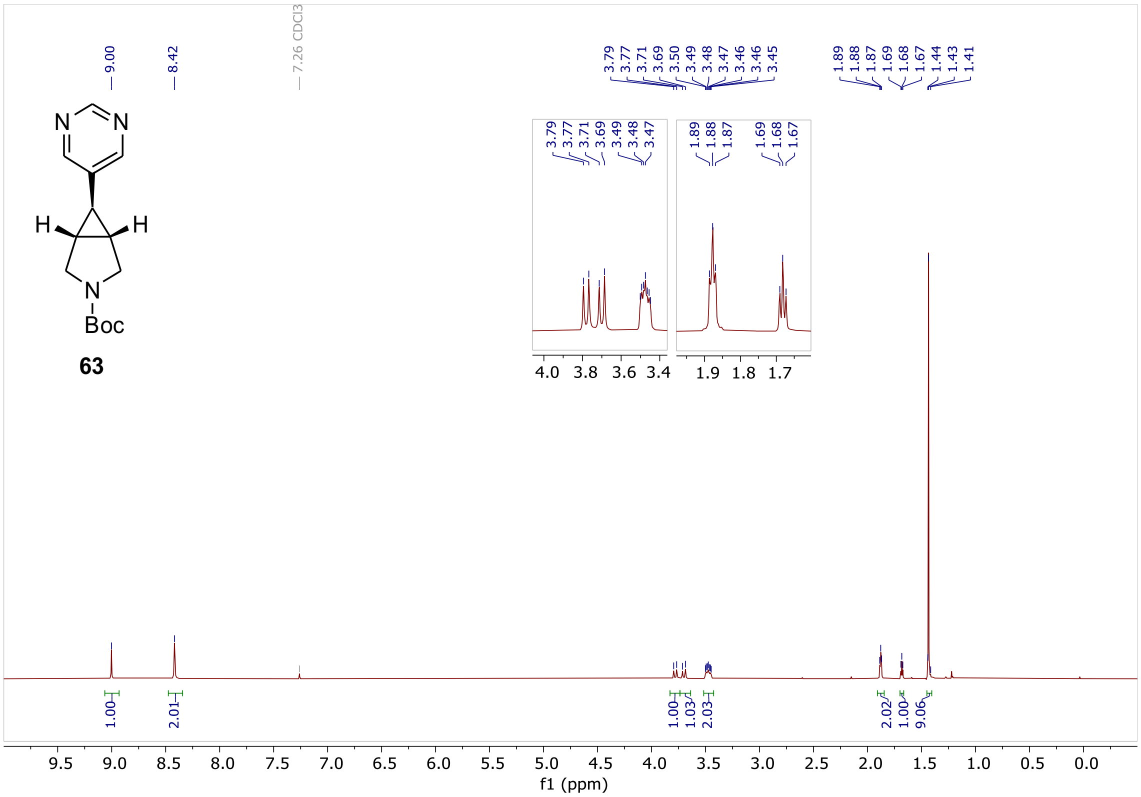

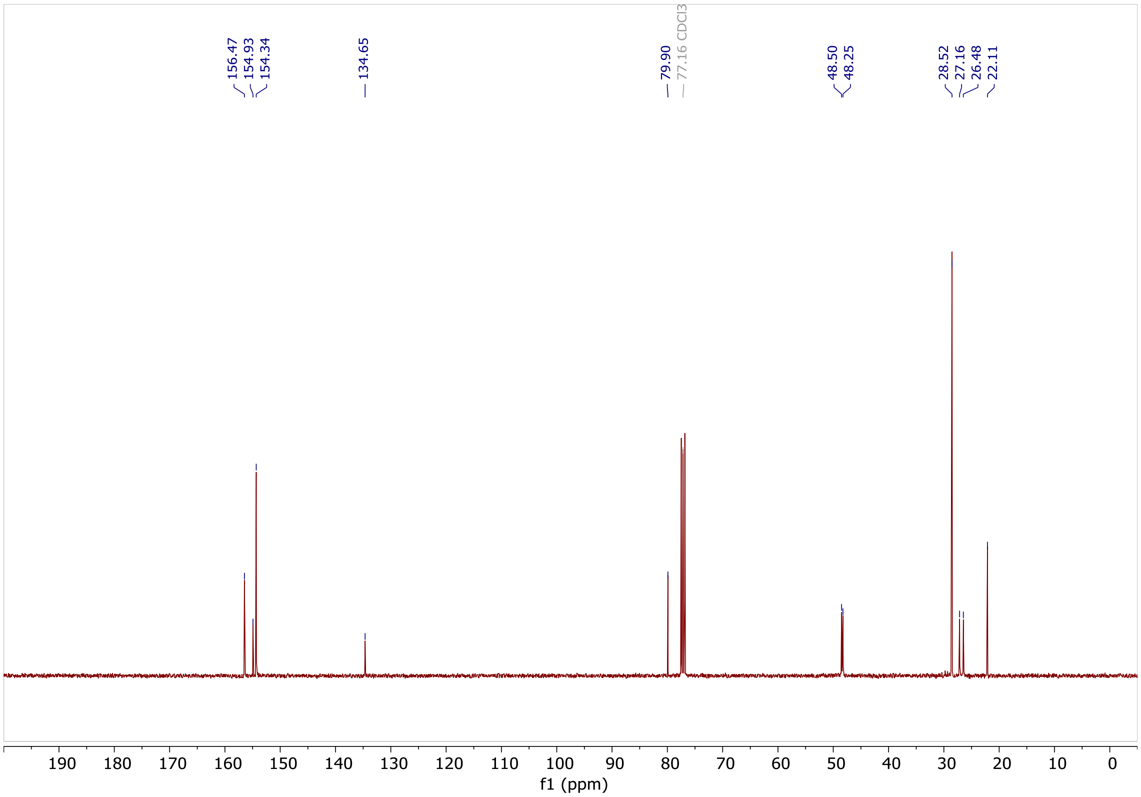

400 MHz ^1H NMR spectrum; 100.6 MHz ^{13}C NMR spectrum; $CDCl_3$ of **64**

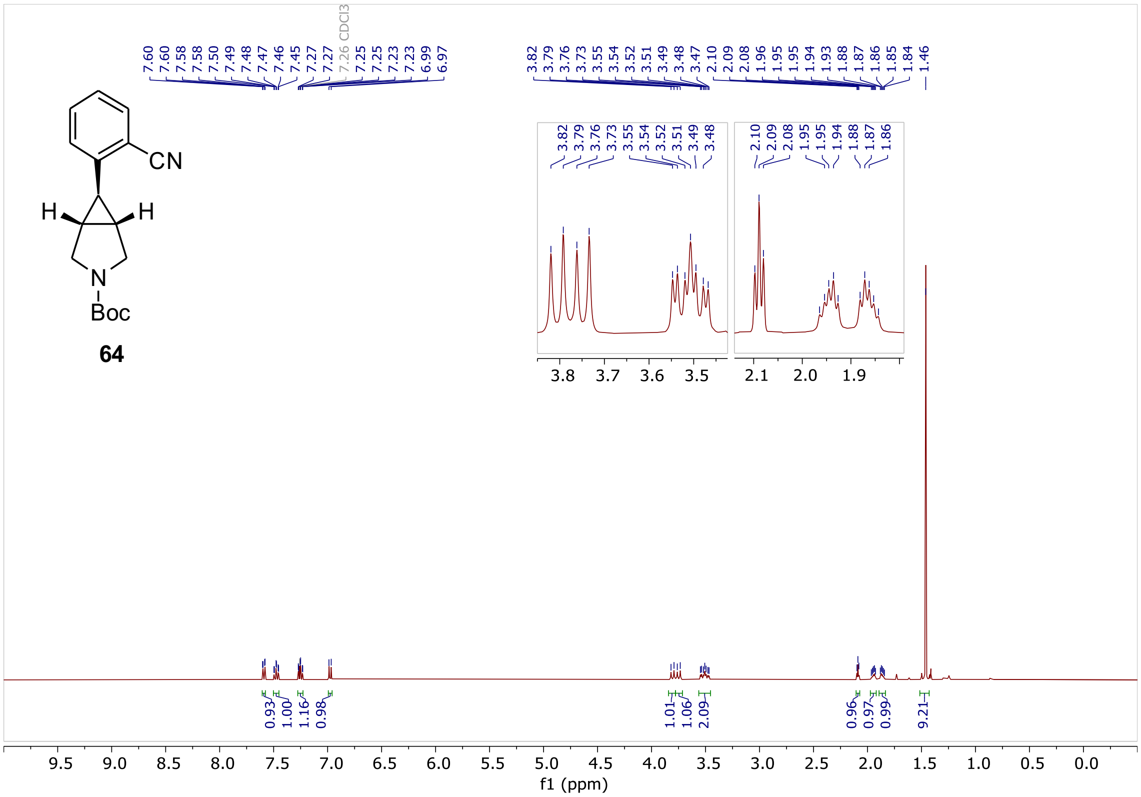

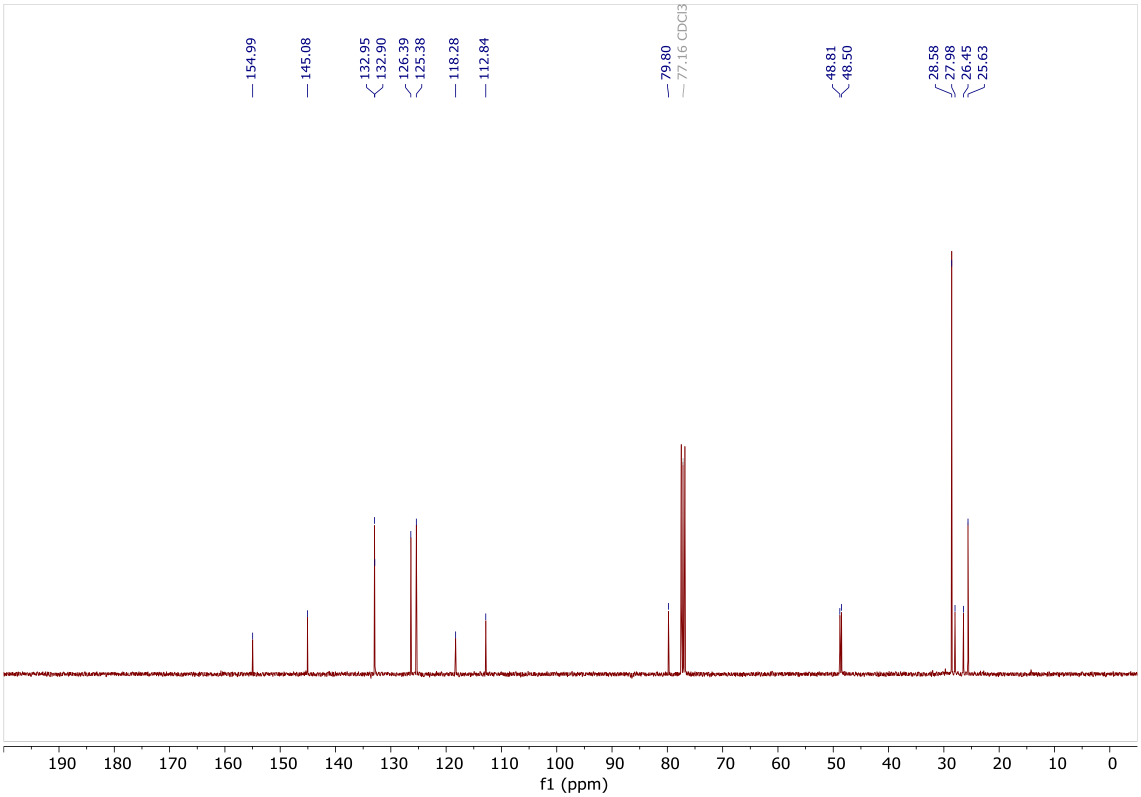

400 MHz ^{1}H NMR spectrum; 100.6 MHz ^{13}C NMR spectrum; $CDCl_3$ of **65**

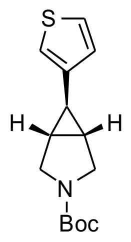

65

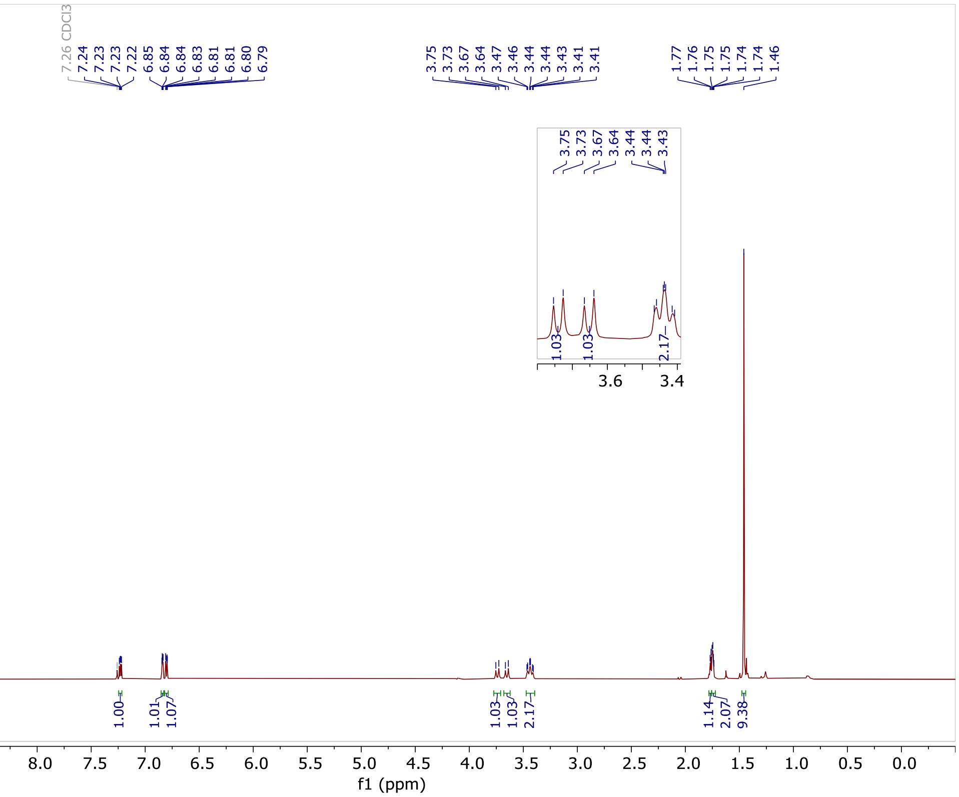

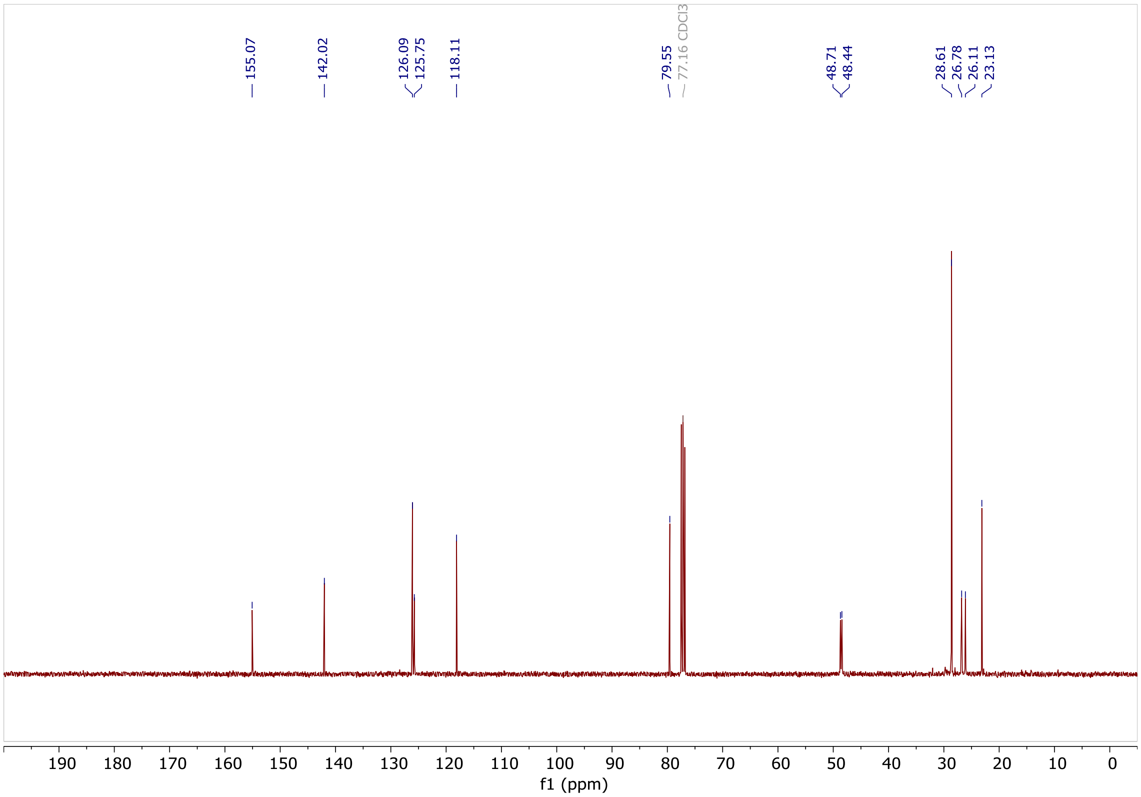

400 MHz ^{1}H NMR spectrum; 100.6 MHz ^{13}C NMR spectrum; $CDCl_3$ of **66**

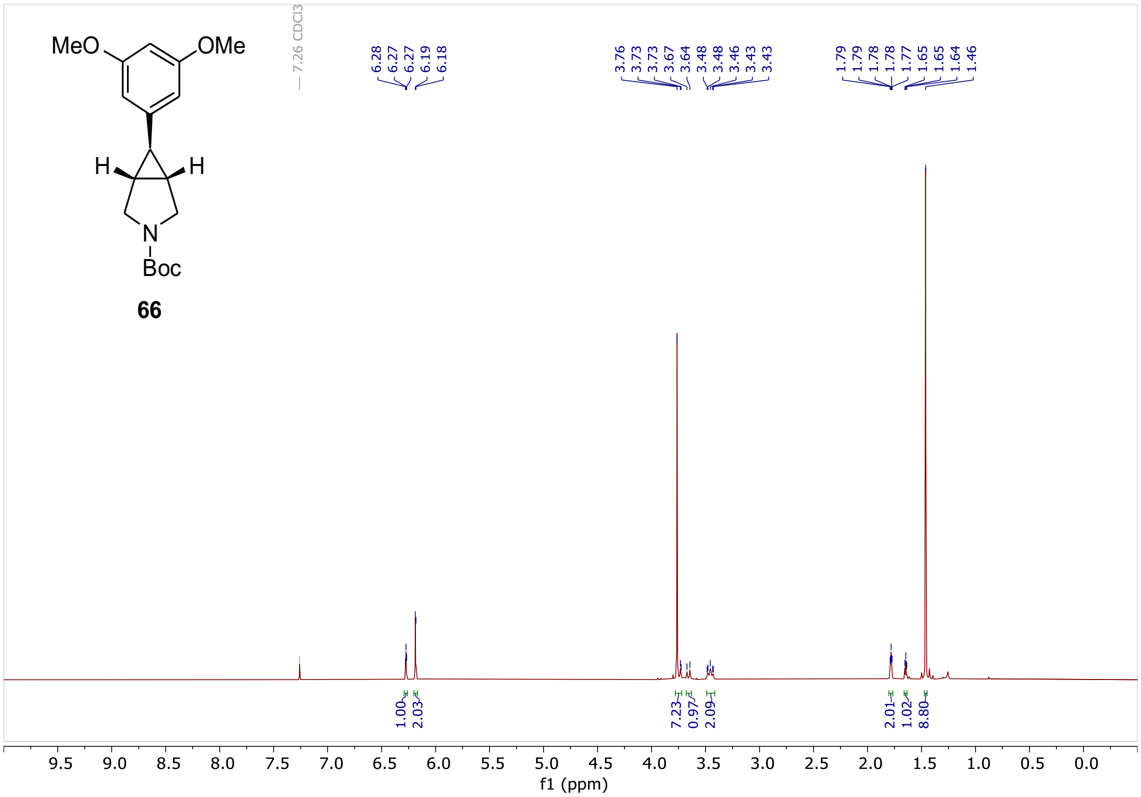

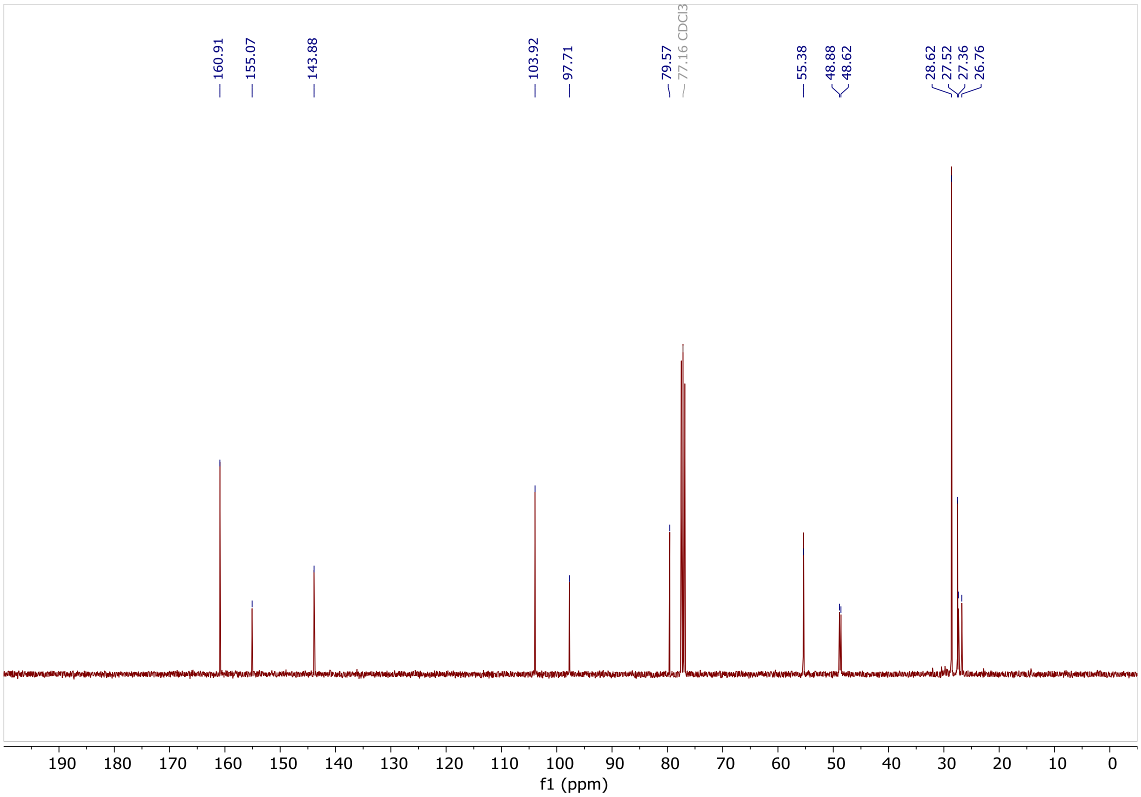

400 MHz ^{1}H NMR spectrum; 100.6 MHz ^{13}C NMR spectrum; $CDCl_3$ of **67**

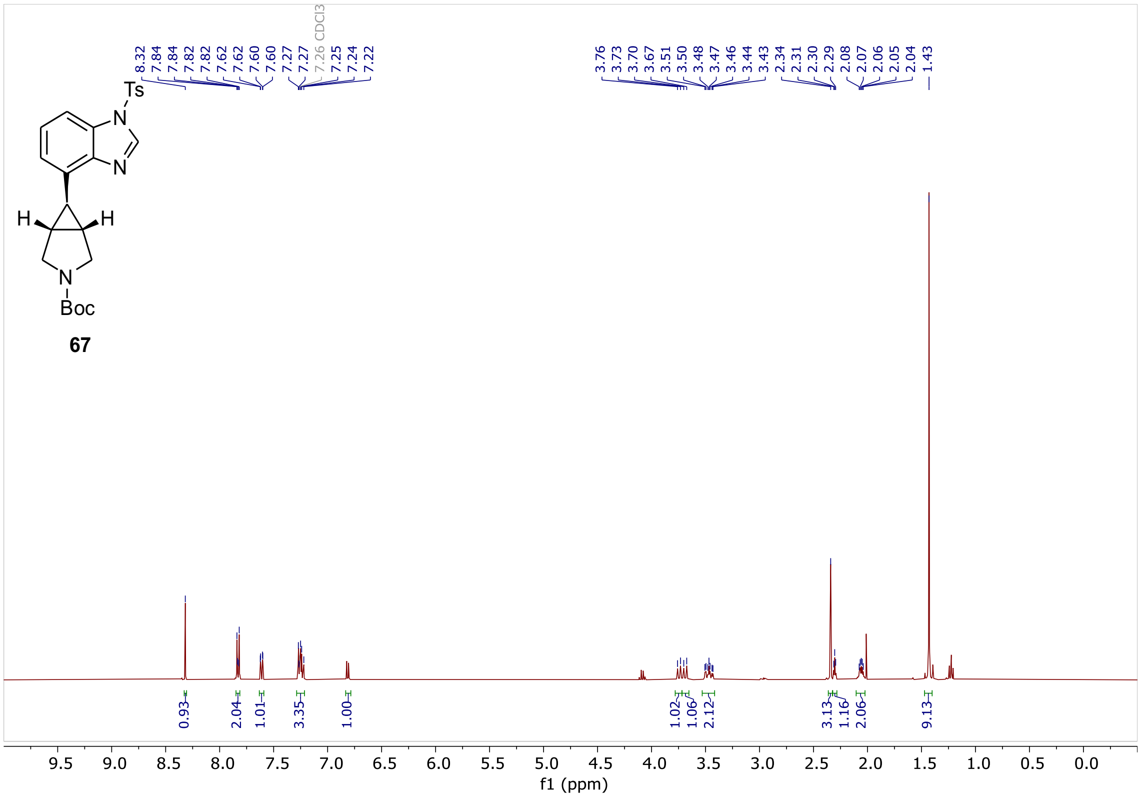

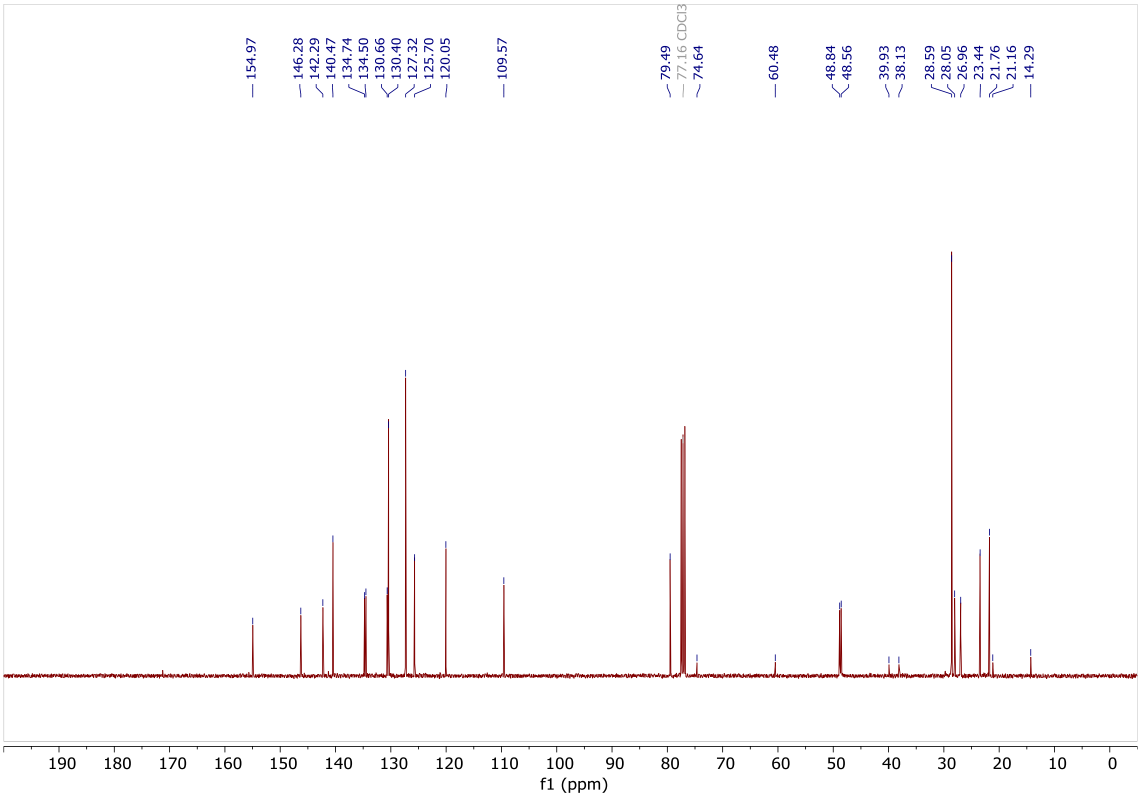

400 MHz ^{1}H NMR spectrum; 100.6 MHz ^{13}C NMR spectrum; $CDCl_3$ of **68**

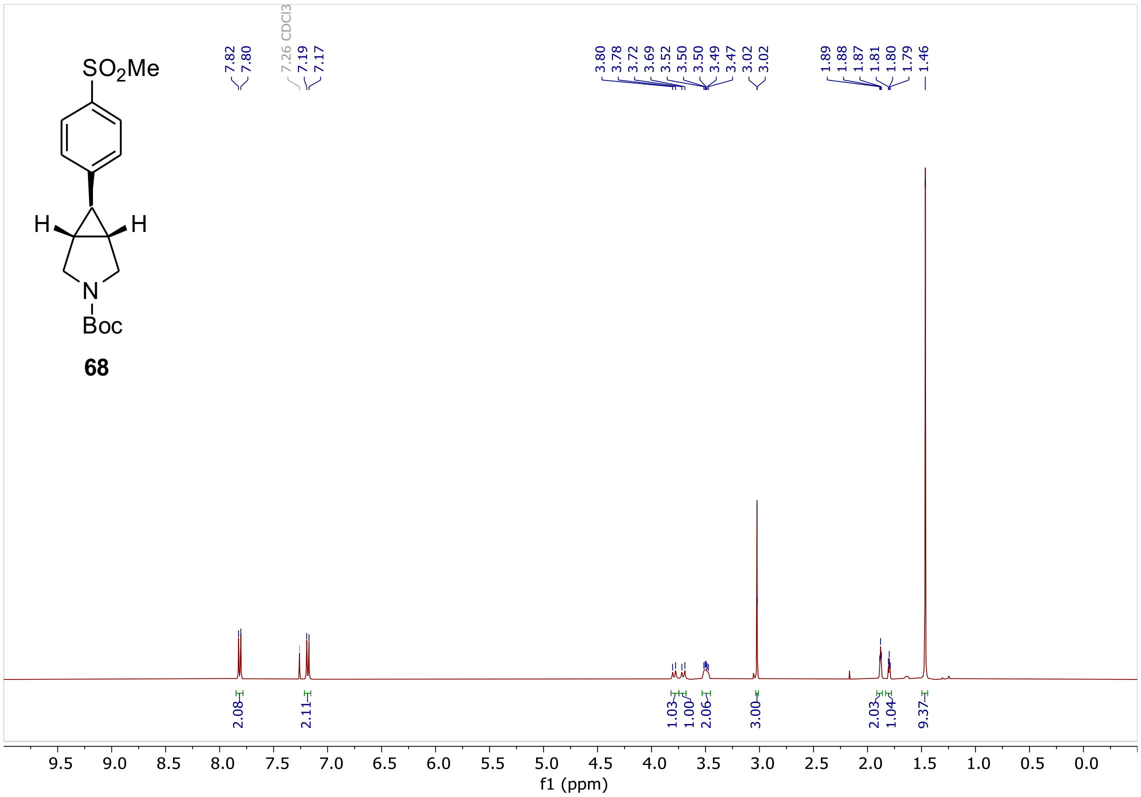

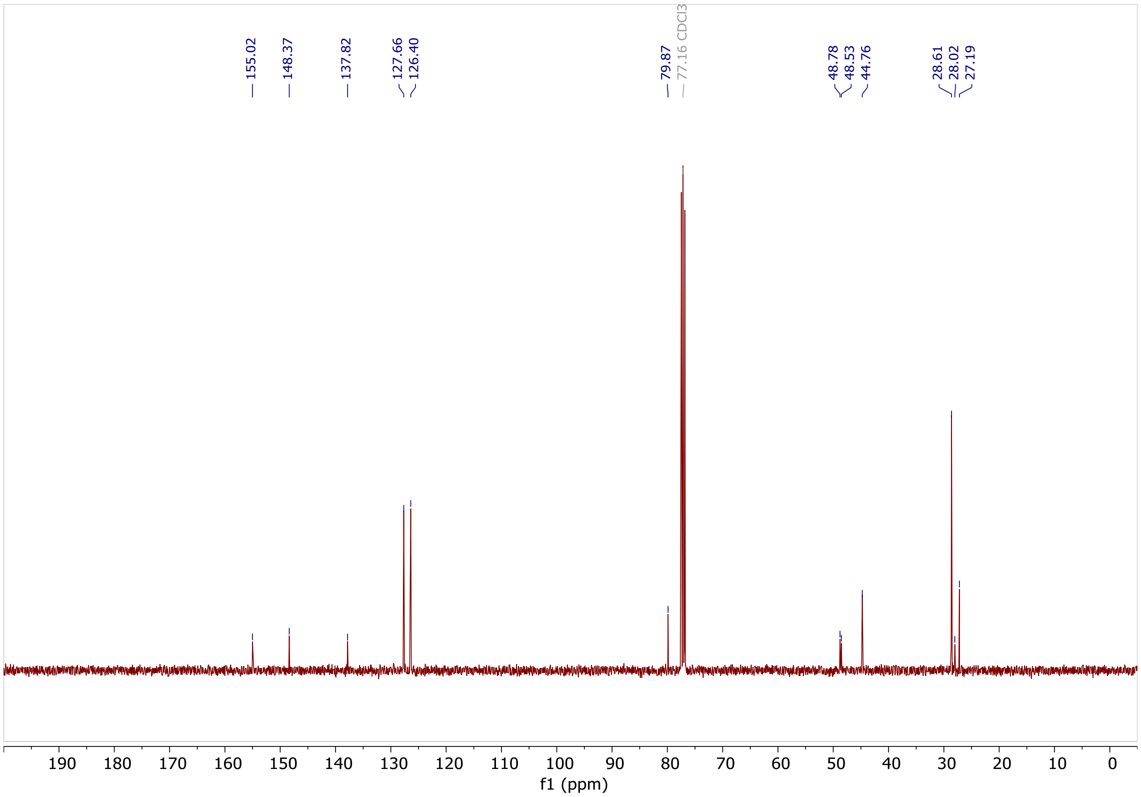

400 MHz ¹H NMR spectrum; 100.6 MHz ¹³C NMR spectrum; $CDCl_3$ of **69**

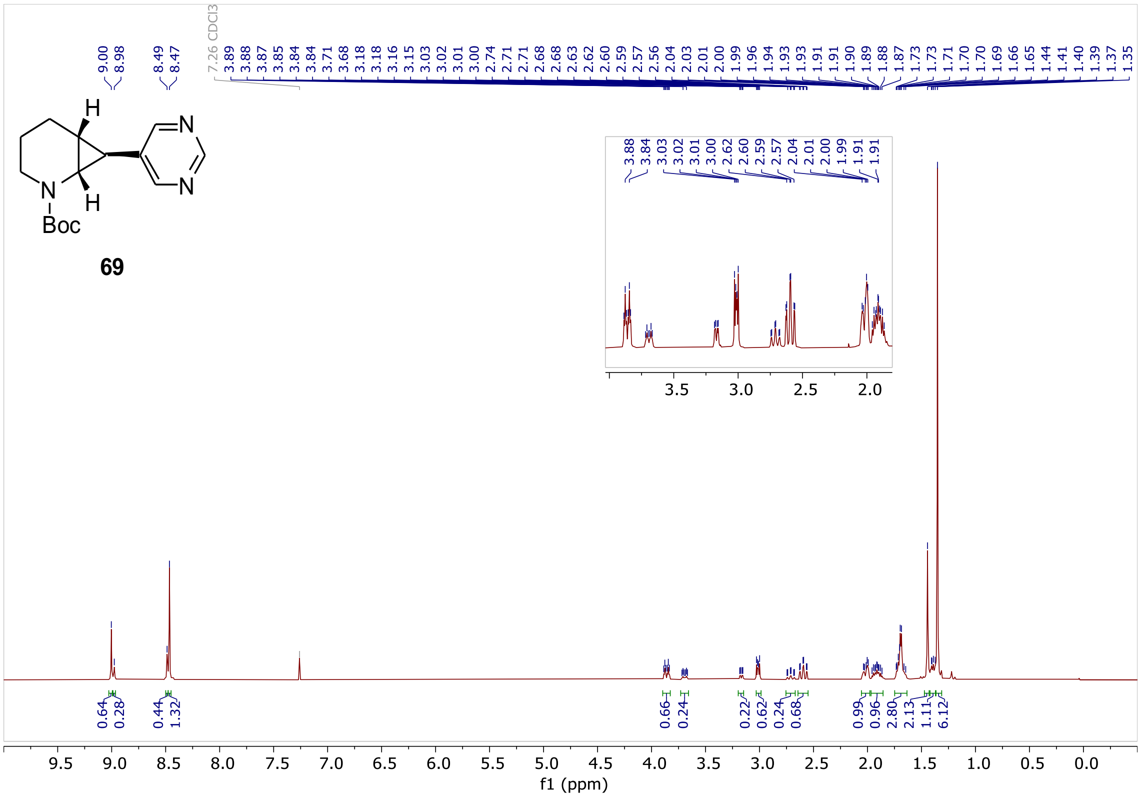

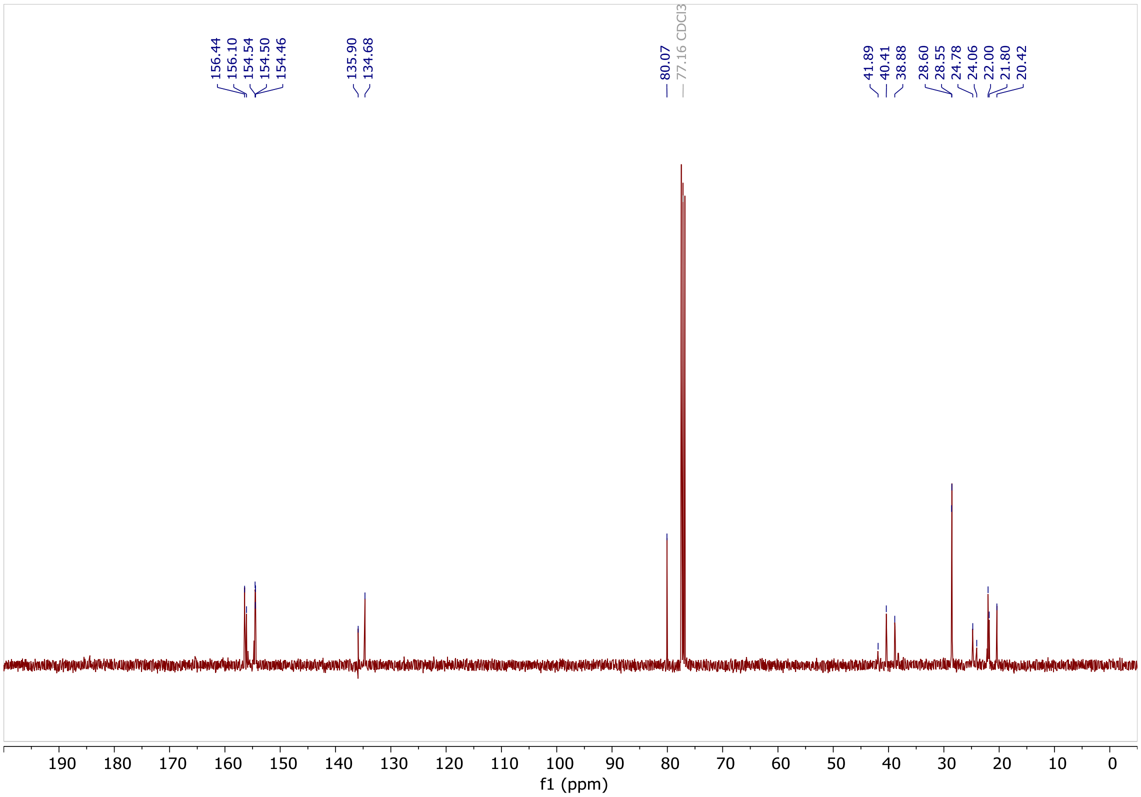

400 MHz ^{1}H NMR spectrum; 100.6 MHz ^{13}C NMR spectrum; $CDCl_3$ of **70**

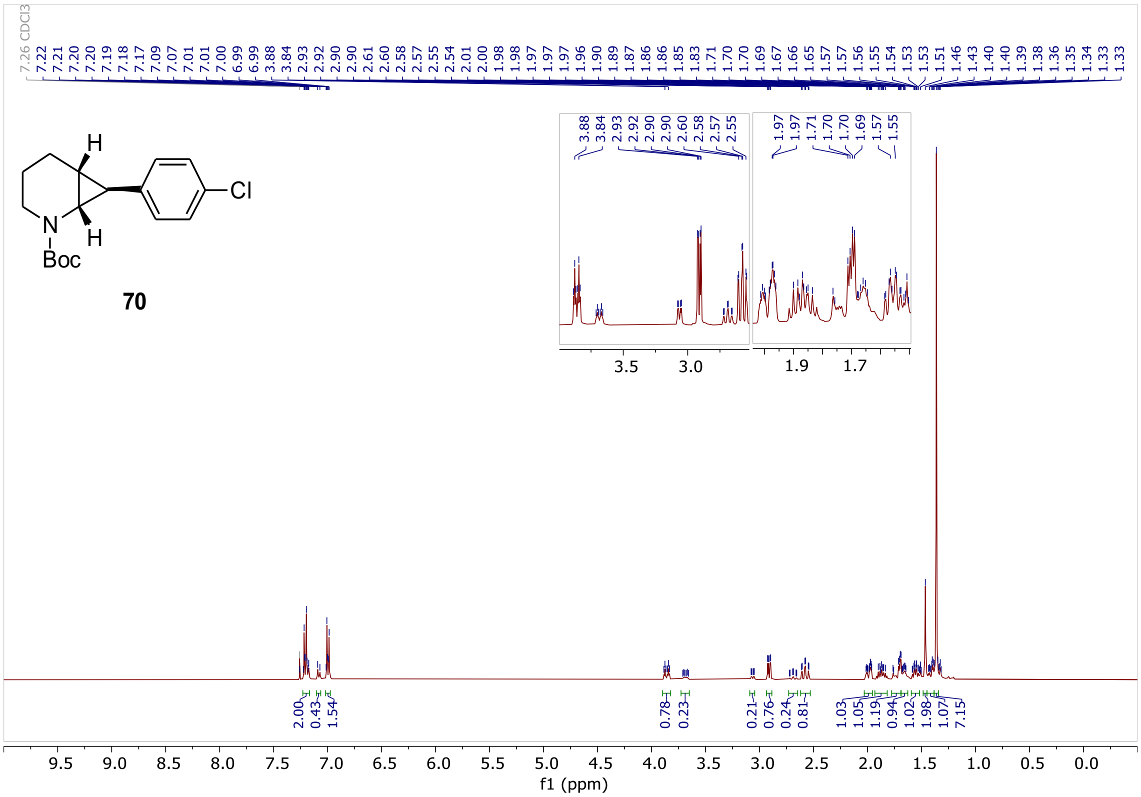

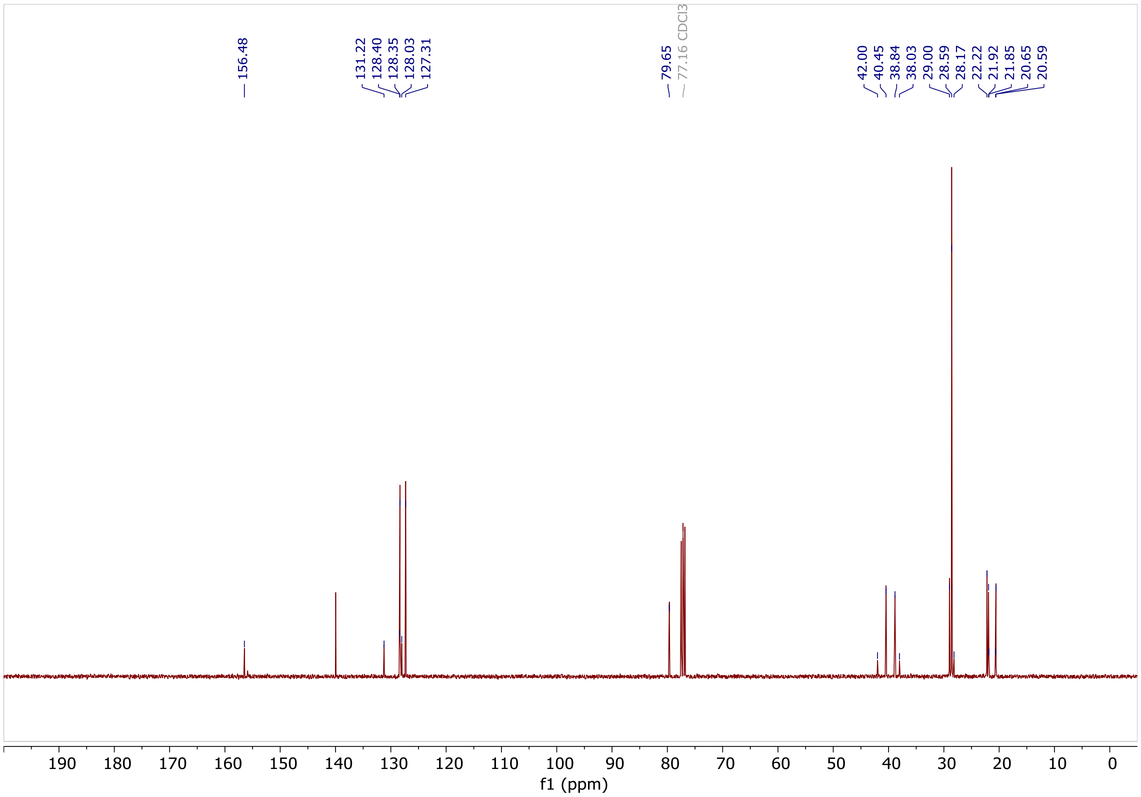

400 MHz ^{1}H NMR spectrum; 100.6 MHz ^{13}C NMR spectrum; $CDCl_3$ of **71**

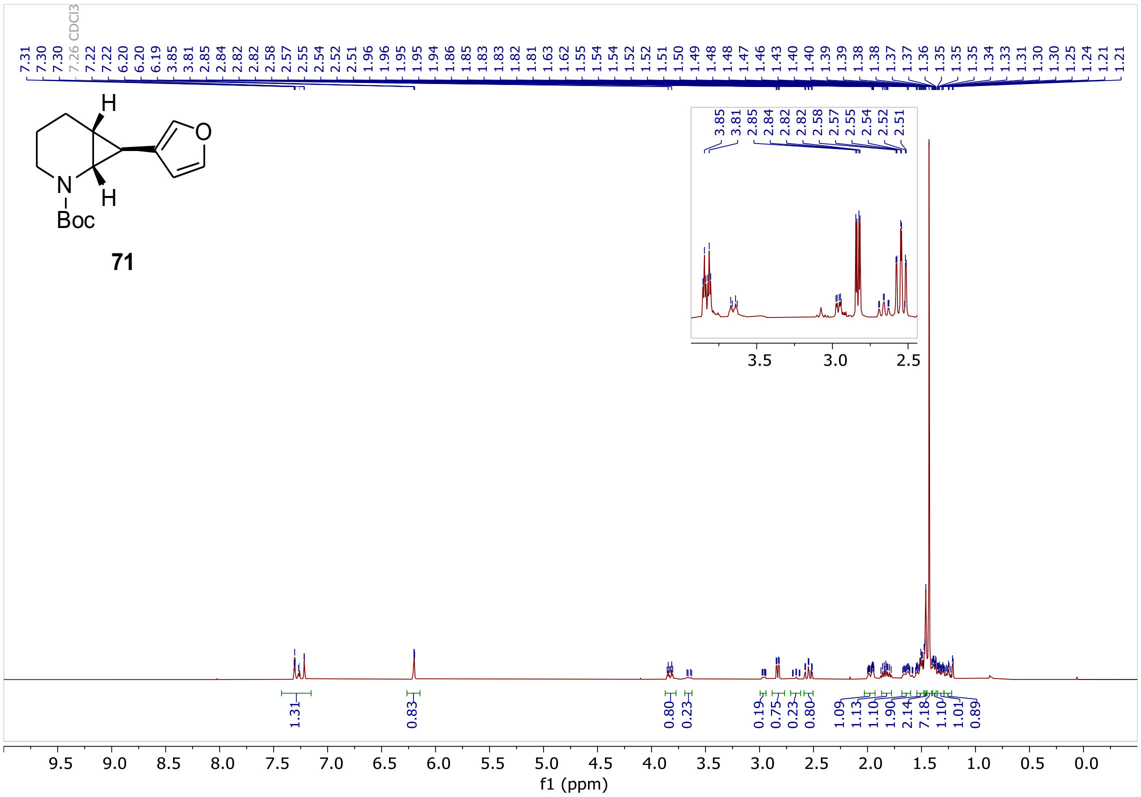

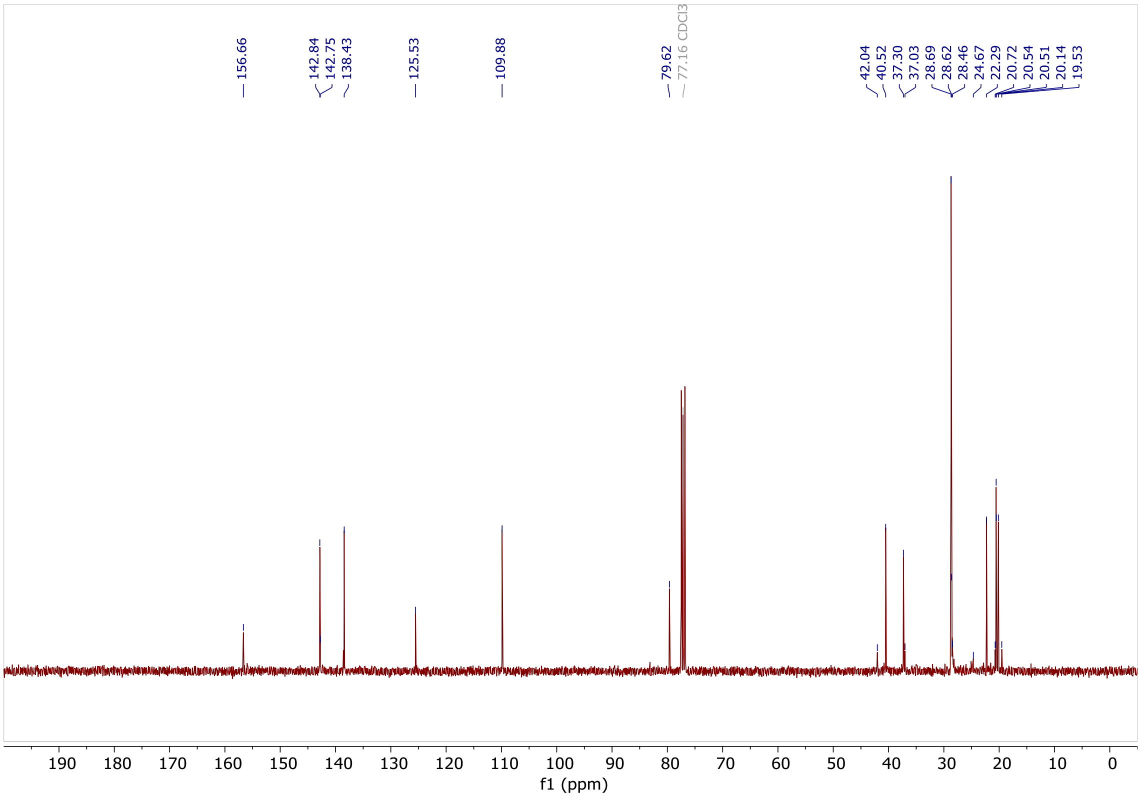

400 MHz ¹H NMR spectrum; 100.6 MHz ¹³C NMR spectrum; $CDCl_3$ of **72**

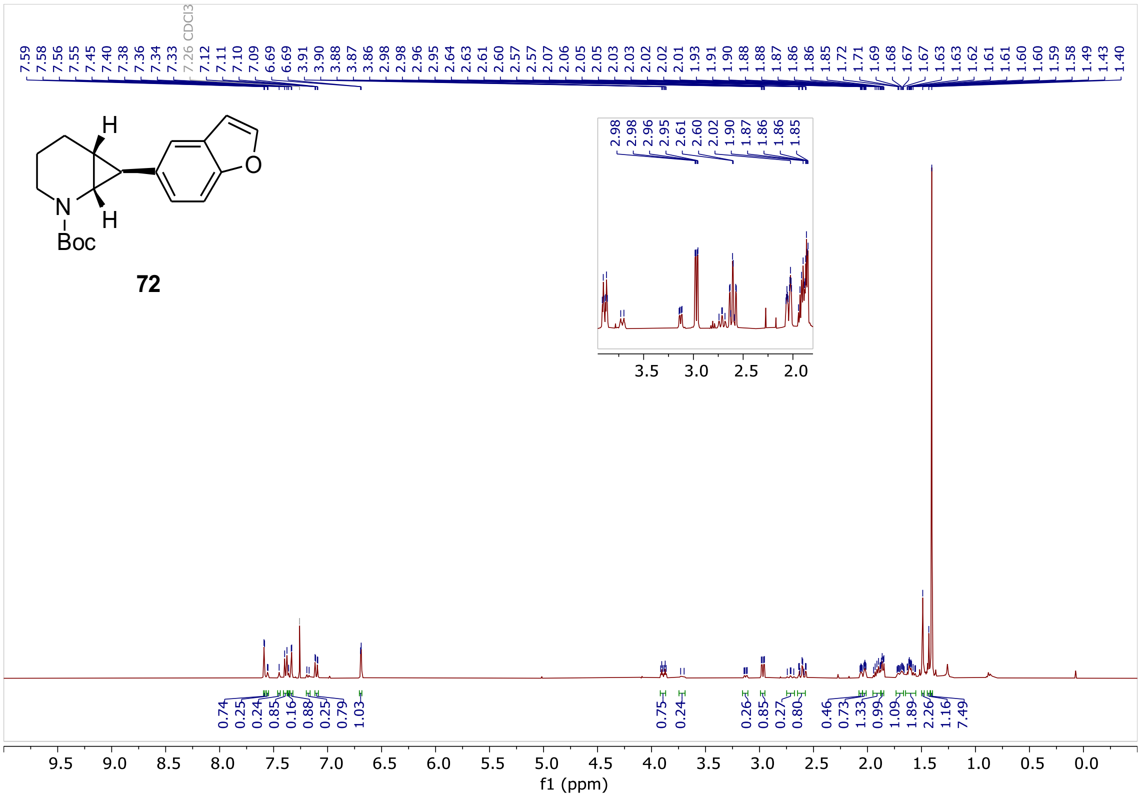

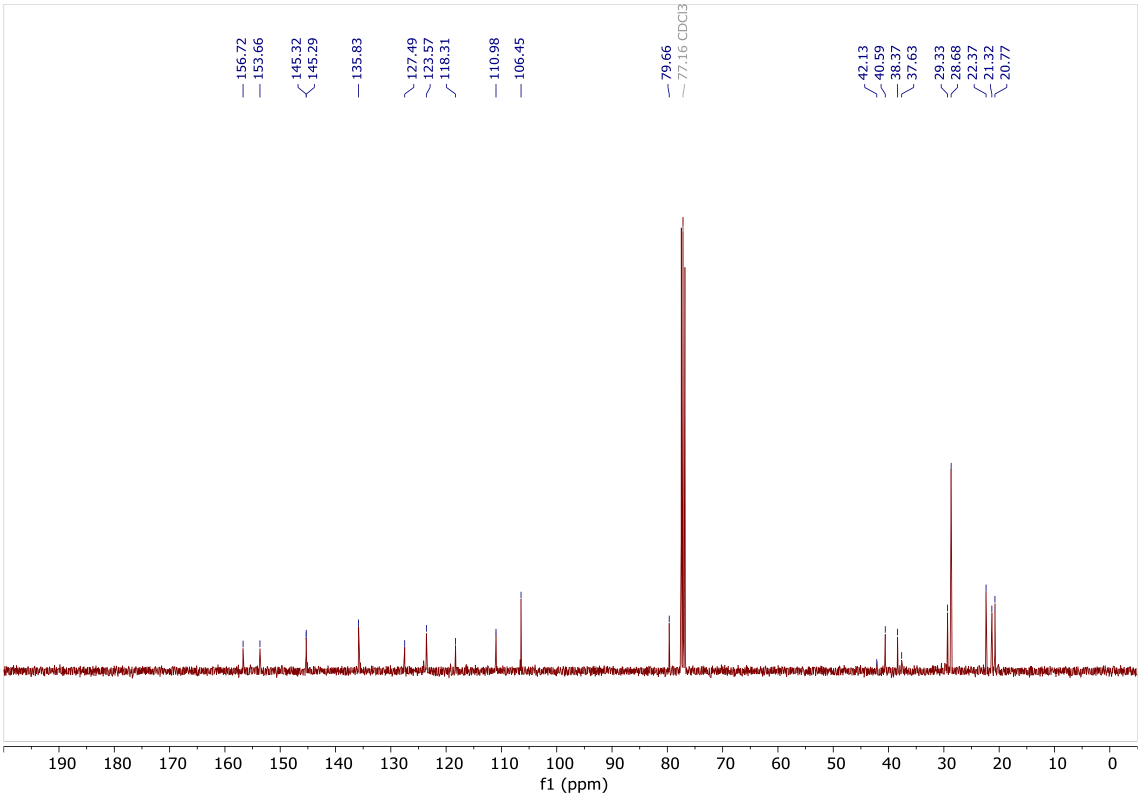

400 MHz ^{1}H NMR spectrum; 100.6 MHz ^{13}C NMR spectrum; $CDCl_3$ of **73**

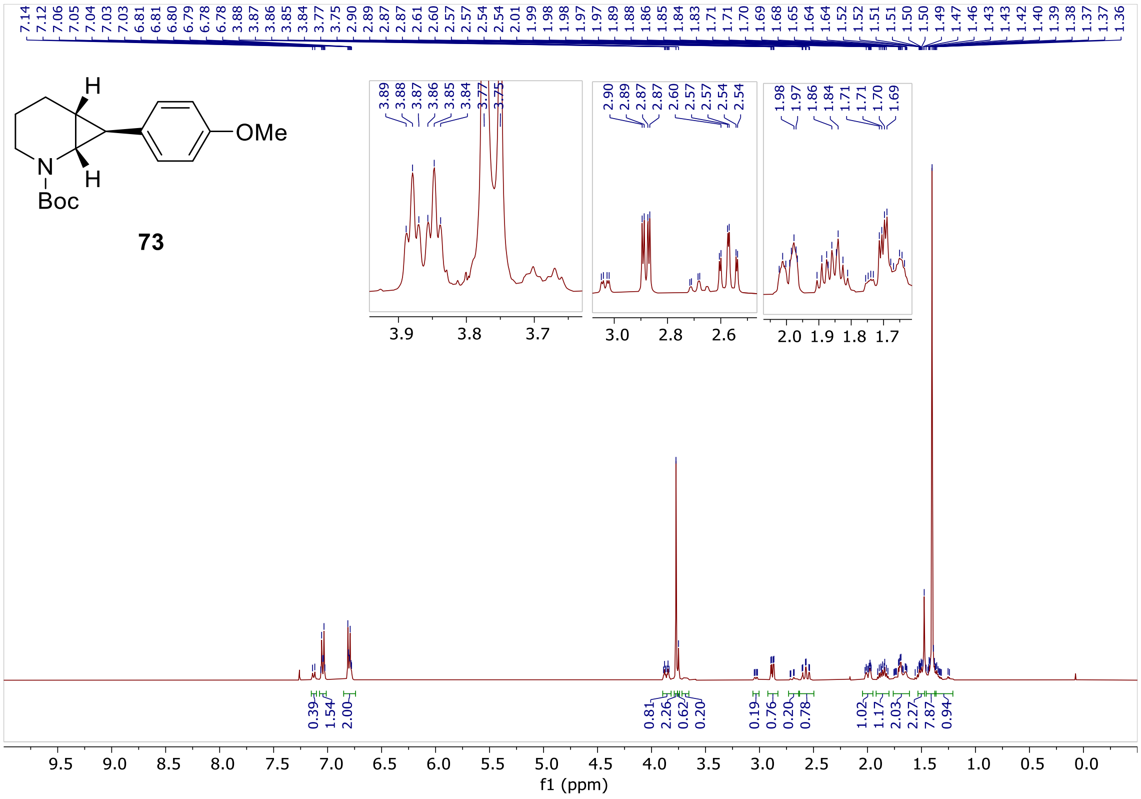

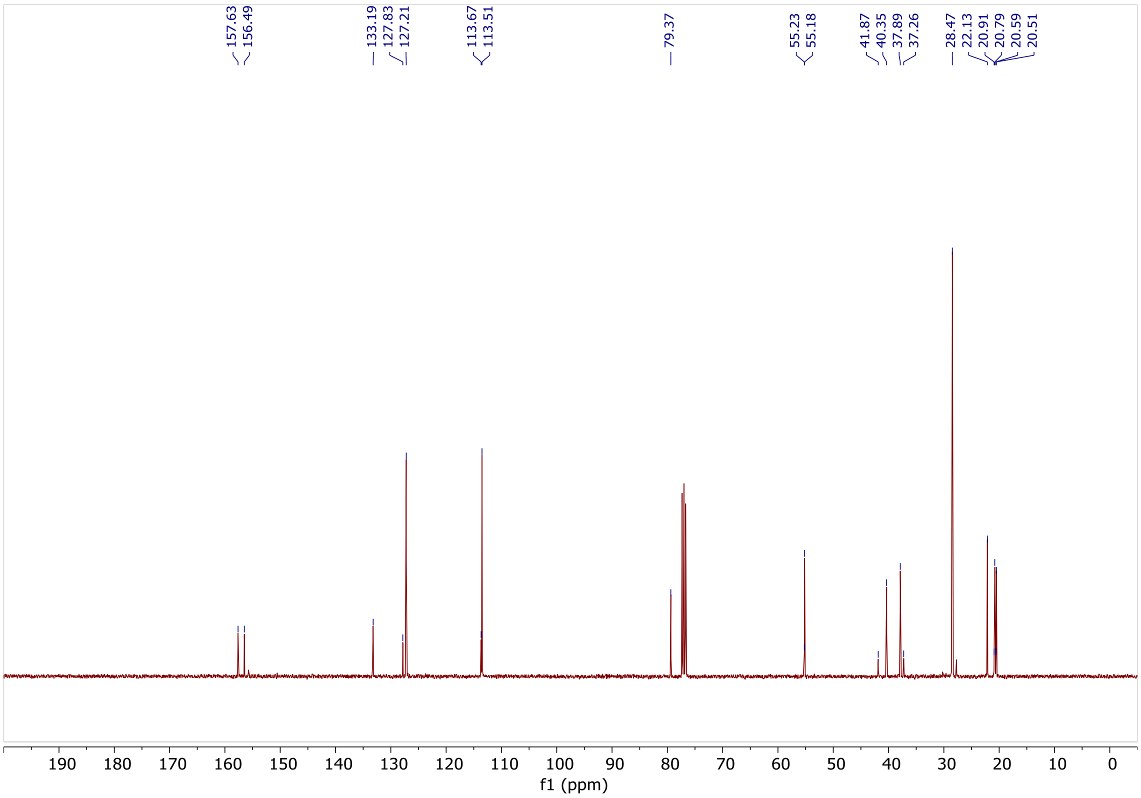

400 MHz ^{1}H NMR spectrum; 100.6 MHz ^{13}C NMR spectrum; $CDCl_3$ of **74**

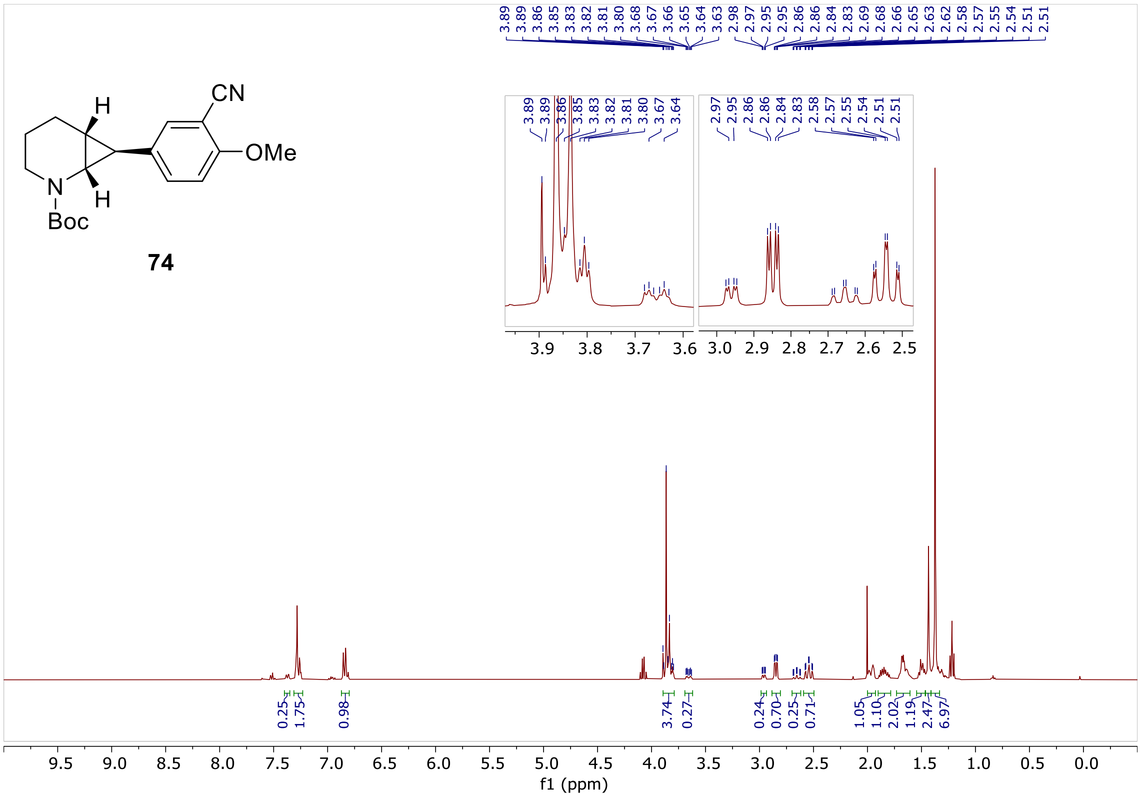

74

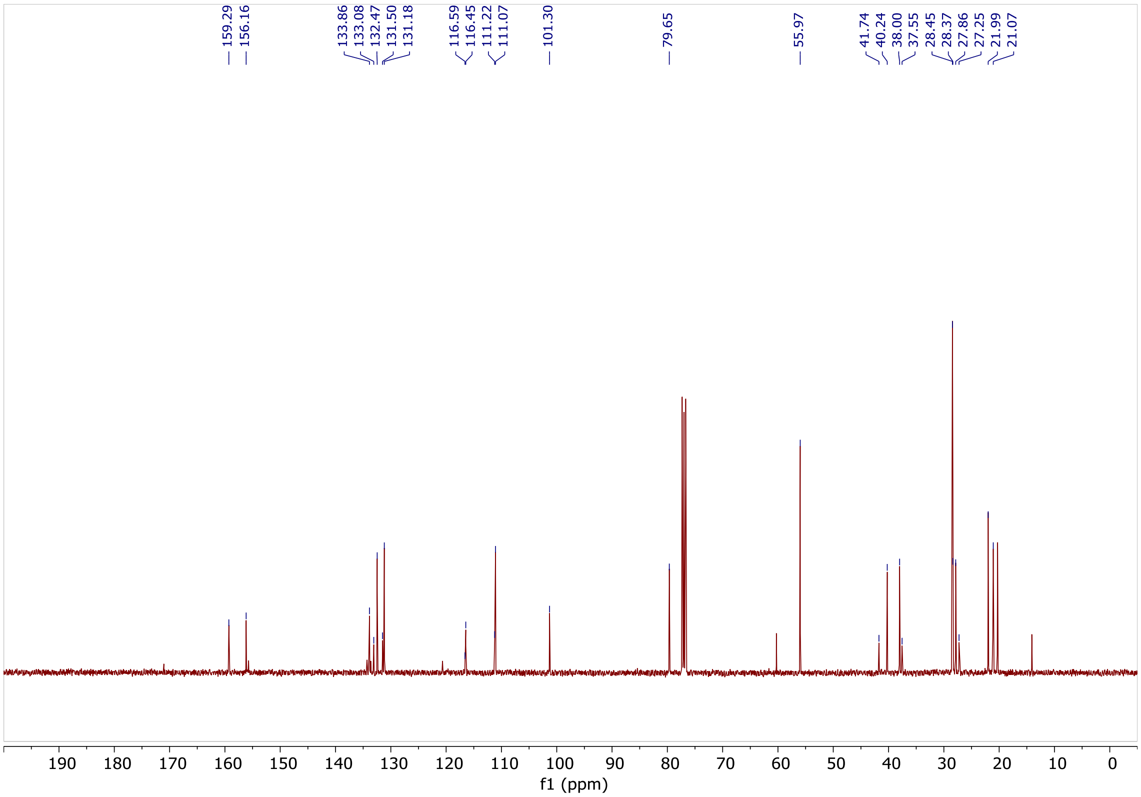

400 MHz ^1H NMR spectrum; 100.6 MHz ^{13}C NMR spectrum; $CDCl_3$ of **75**

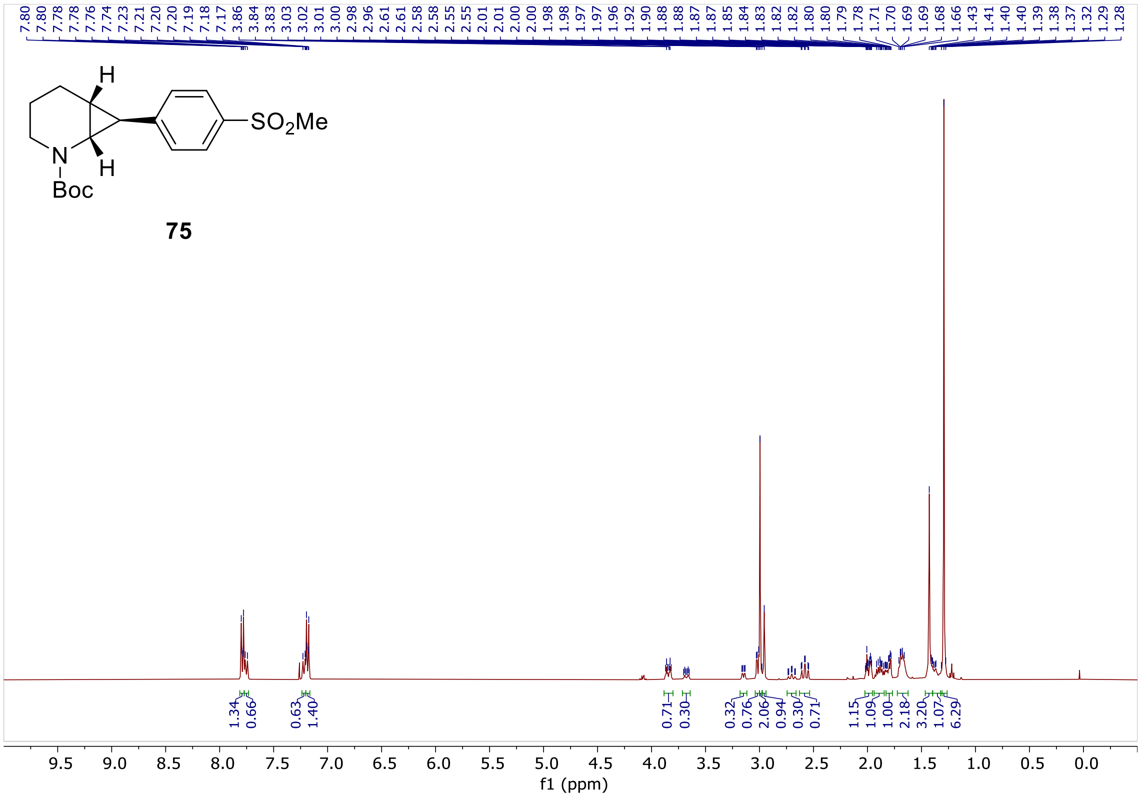

75

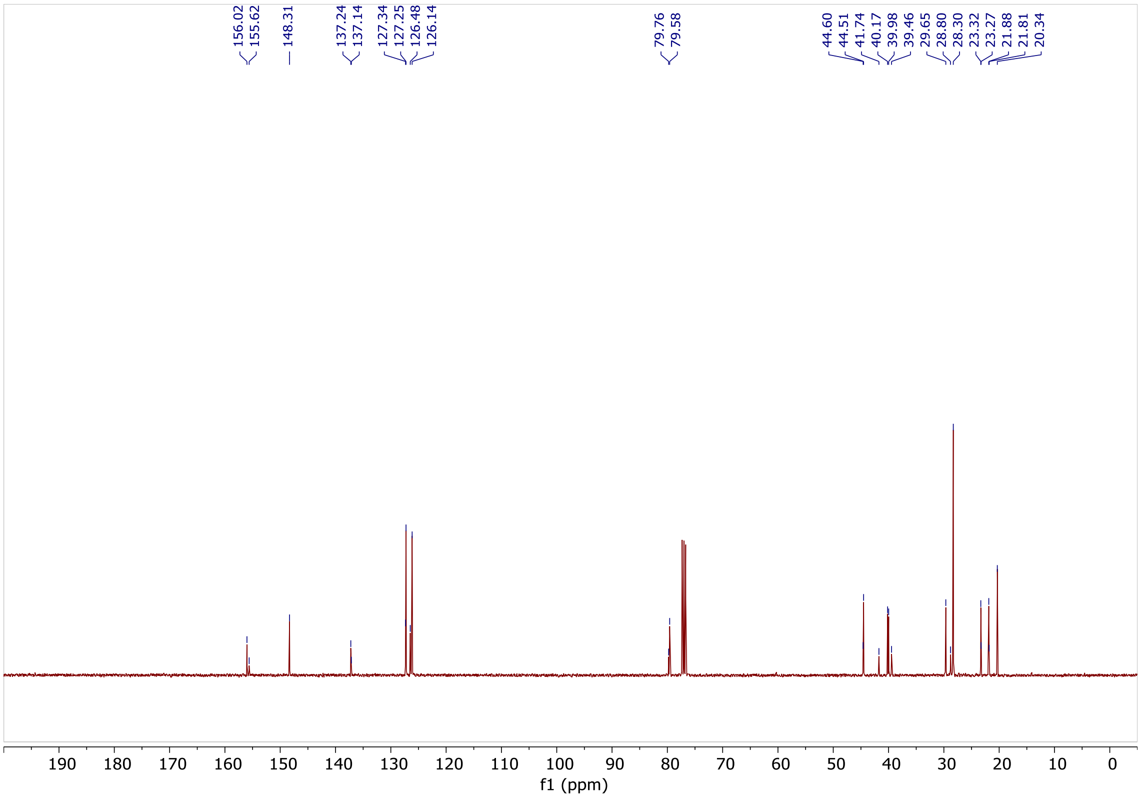

400 MHz ^{1}H NMR spectrum; 100.6 MHz ^{13}C NMR spectrum; $CDCl_3$ of **76**

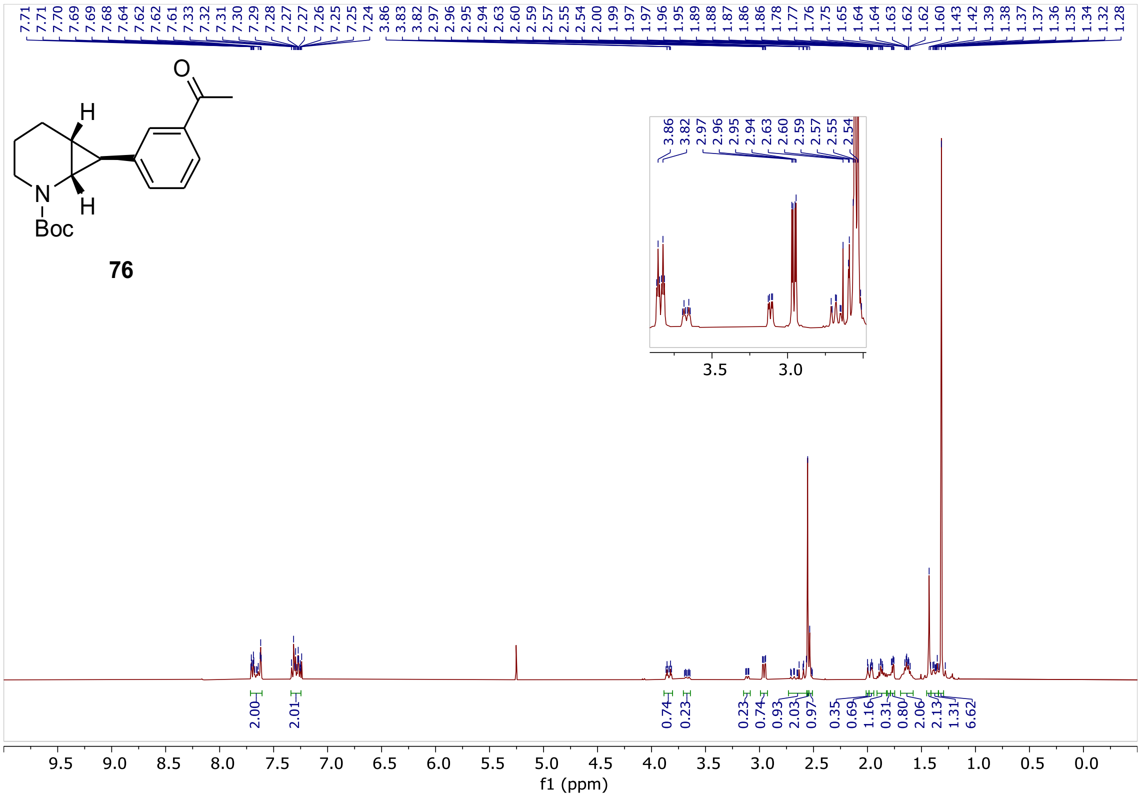

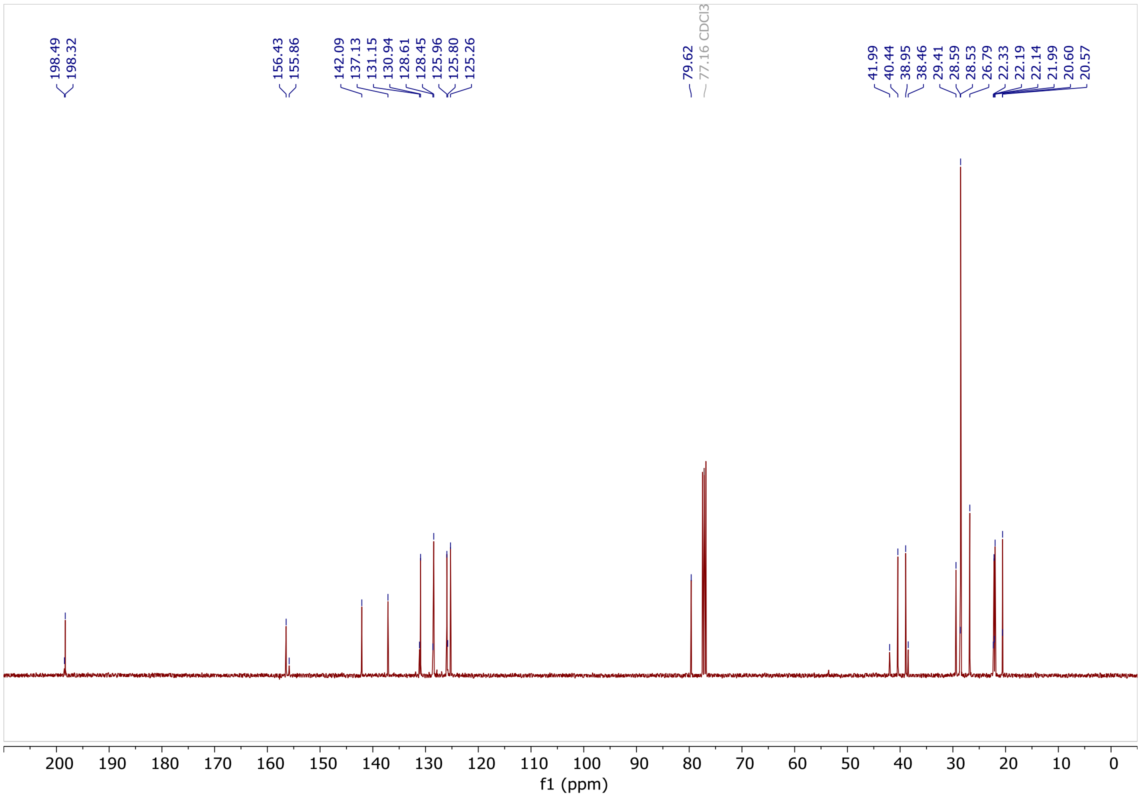

400 MHz ^{1}H NMR spectrum; 100.6 MHz ^{13}C NMR spectrum; $CDCl_3$ of **77**

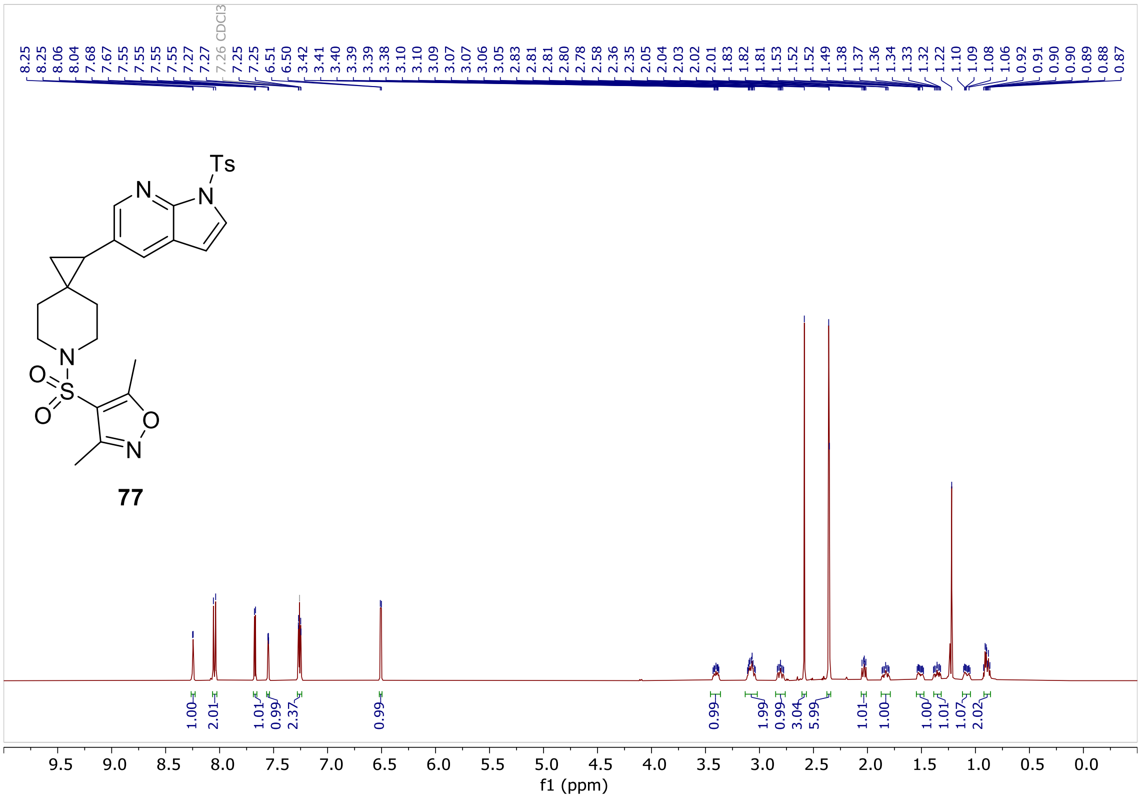

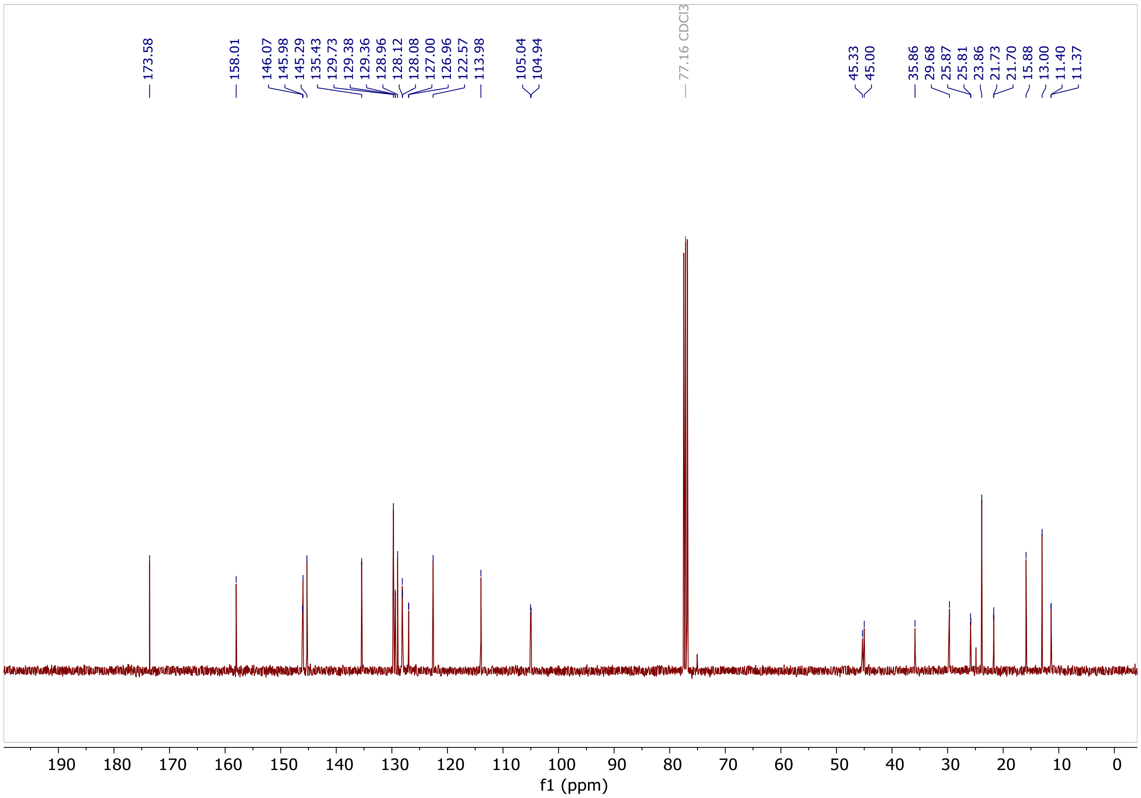

400 MHz ^{1}H NMR spectrum; 100.6 MHz ^{13}C NMR spectrum; $CDCl_3$ of **78**

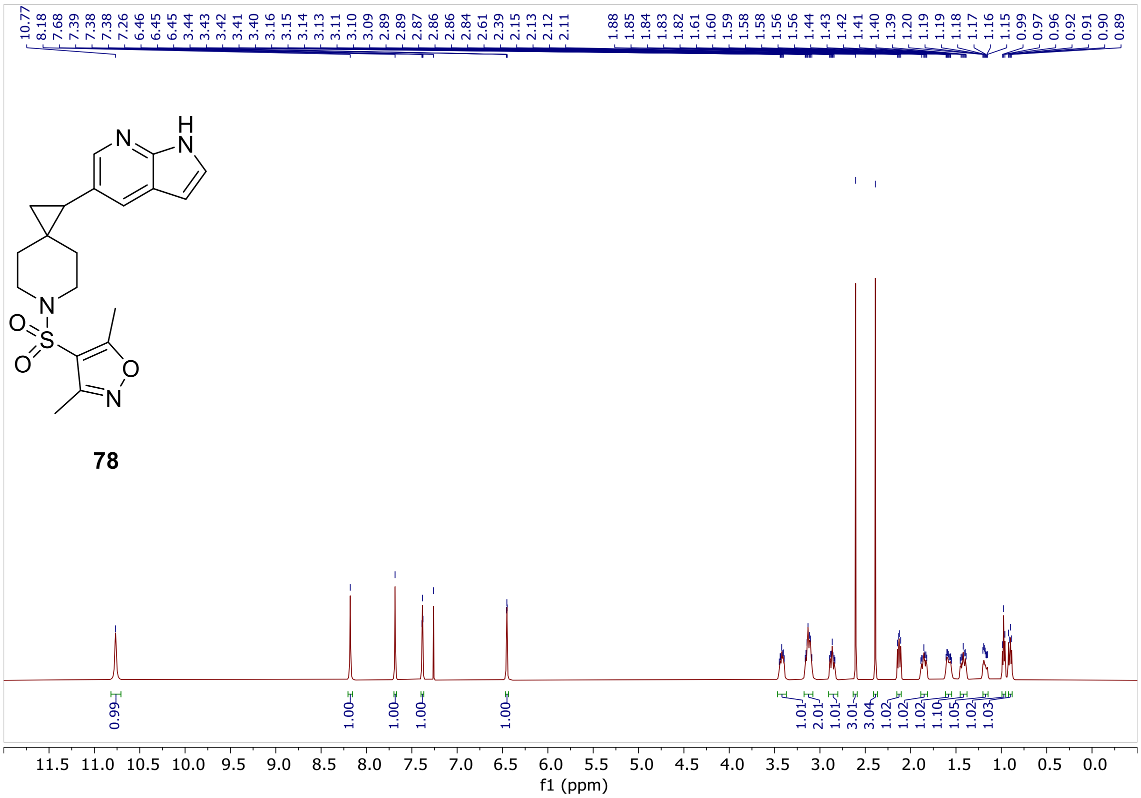

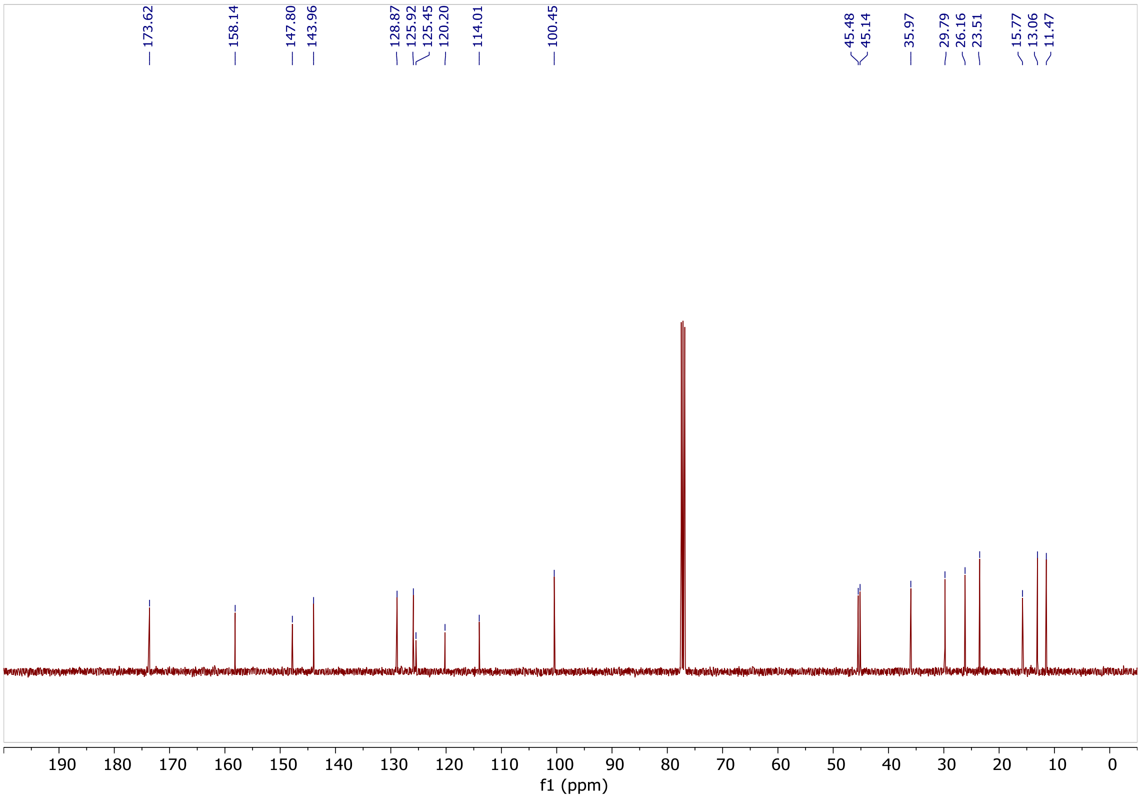

400 MHz ¹H NMR spectrum; 100.6 MHz ¹³C NMR spectrum; $CDCl_3$ of **S33**

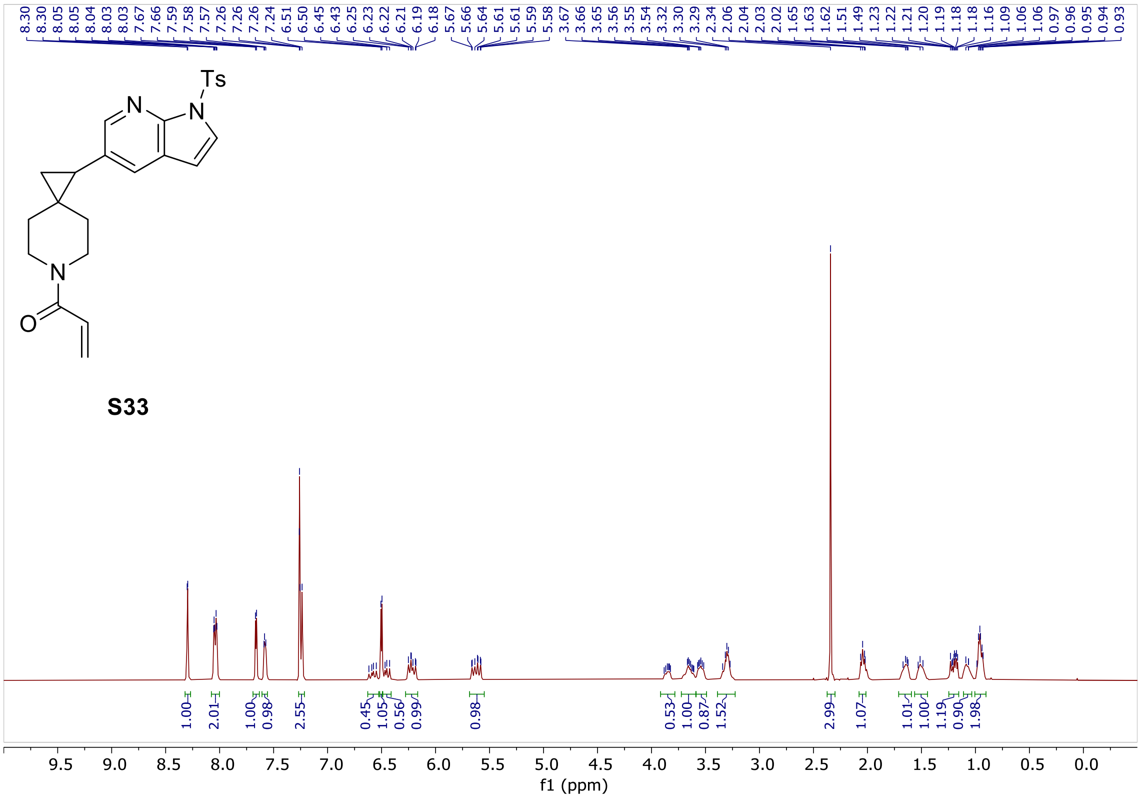

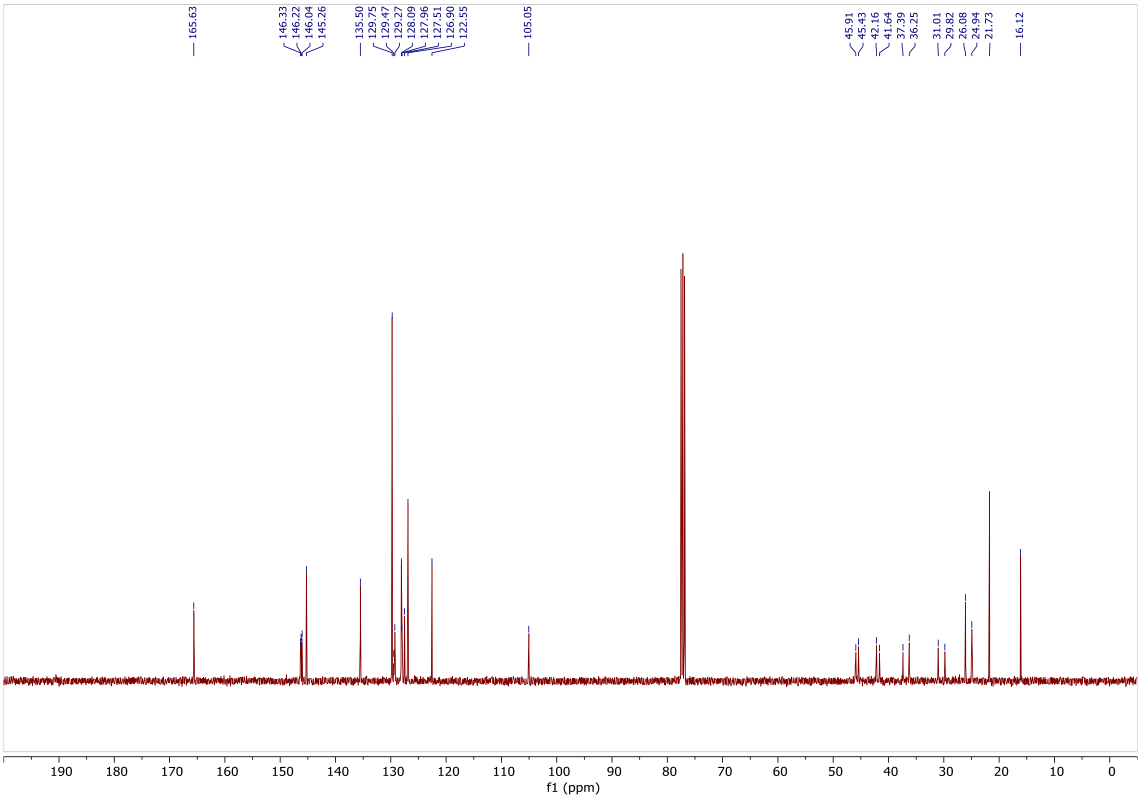

400 MHz ^{1}H NMR spectrum; 100.6 MHz ^{13}C NMR spectrum; $CDCl_3$ of **79**

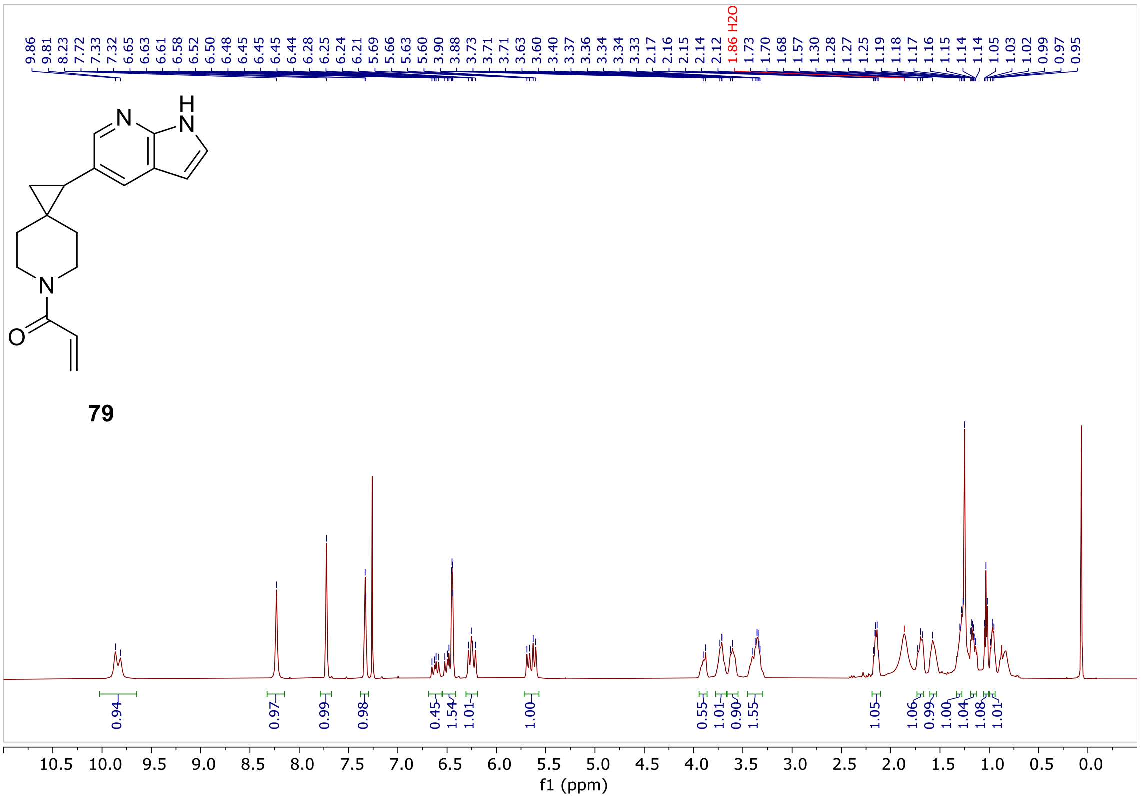

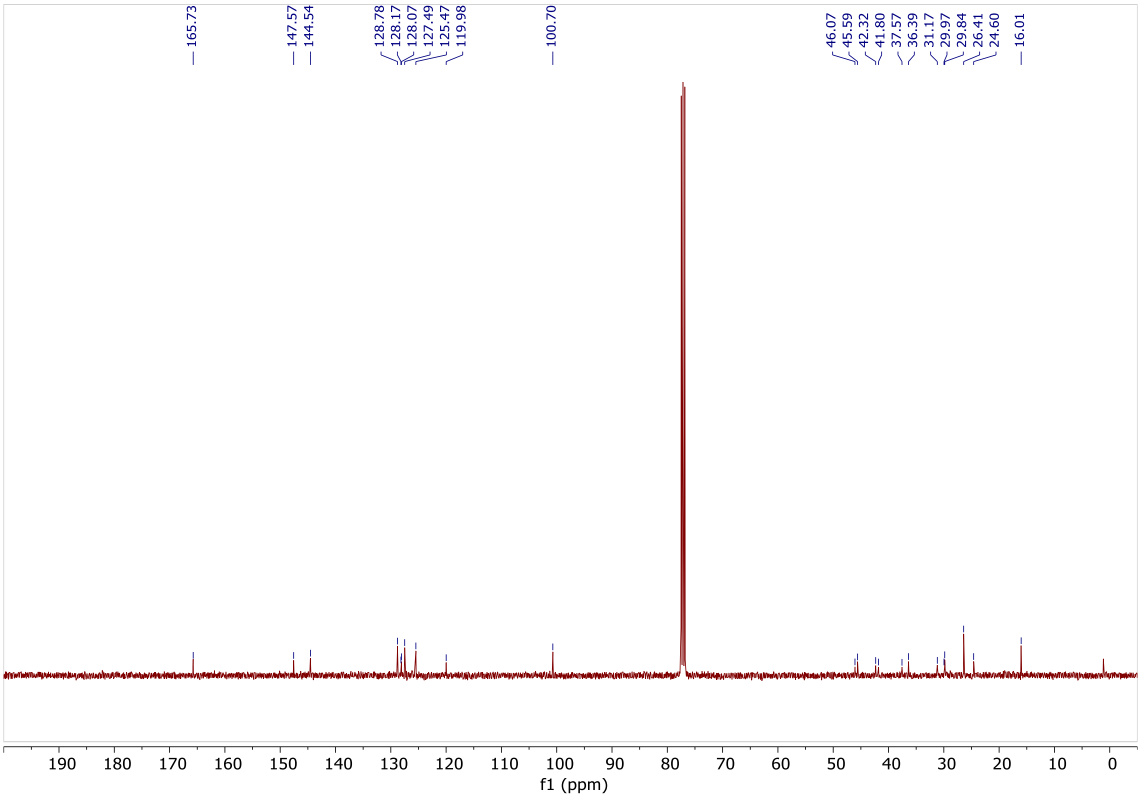

400 MHz ^{1}H NMR spectrum; $CDCl_3$ of **80**

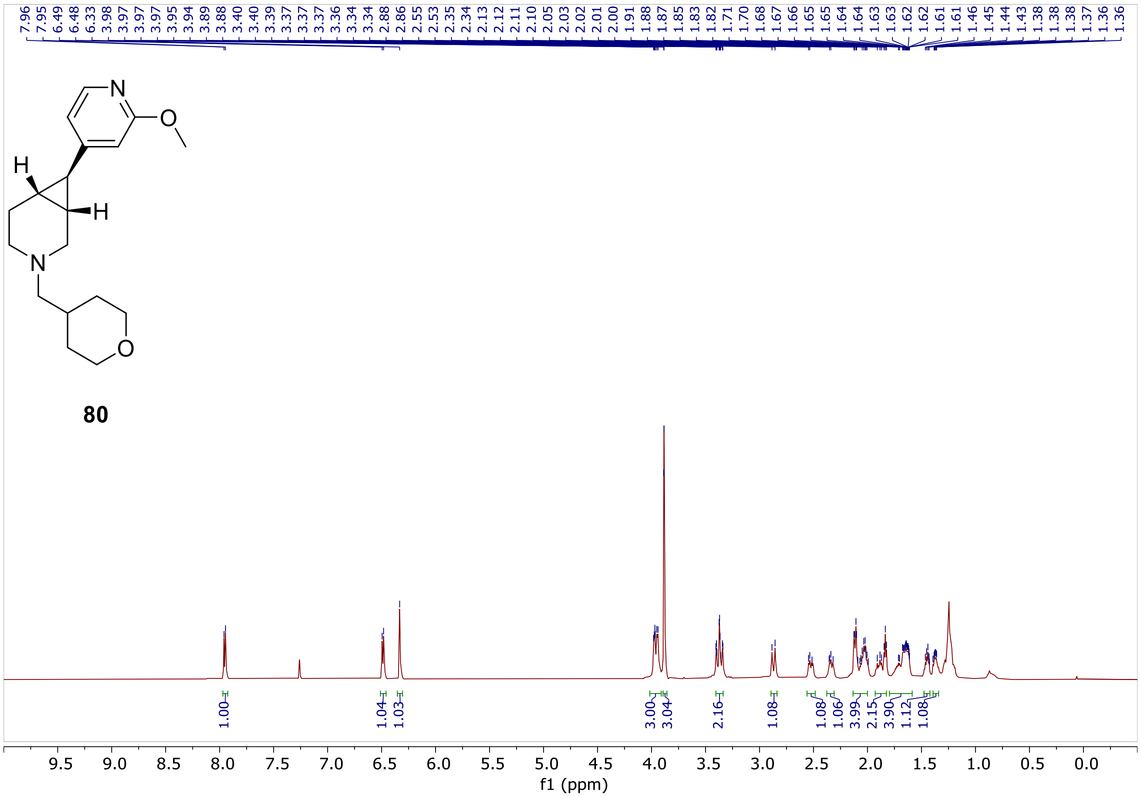

100.6 MHz ^{13}C NMR spectrum; CD_2Cl_2 of **80**

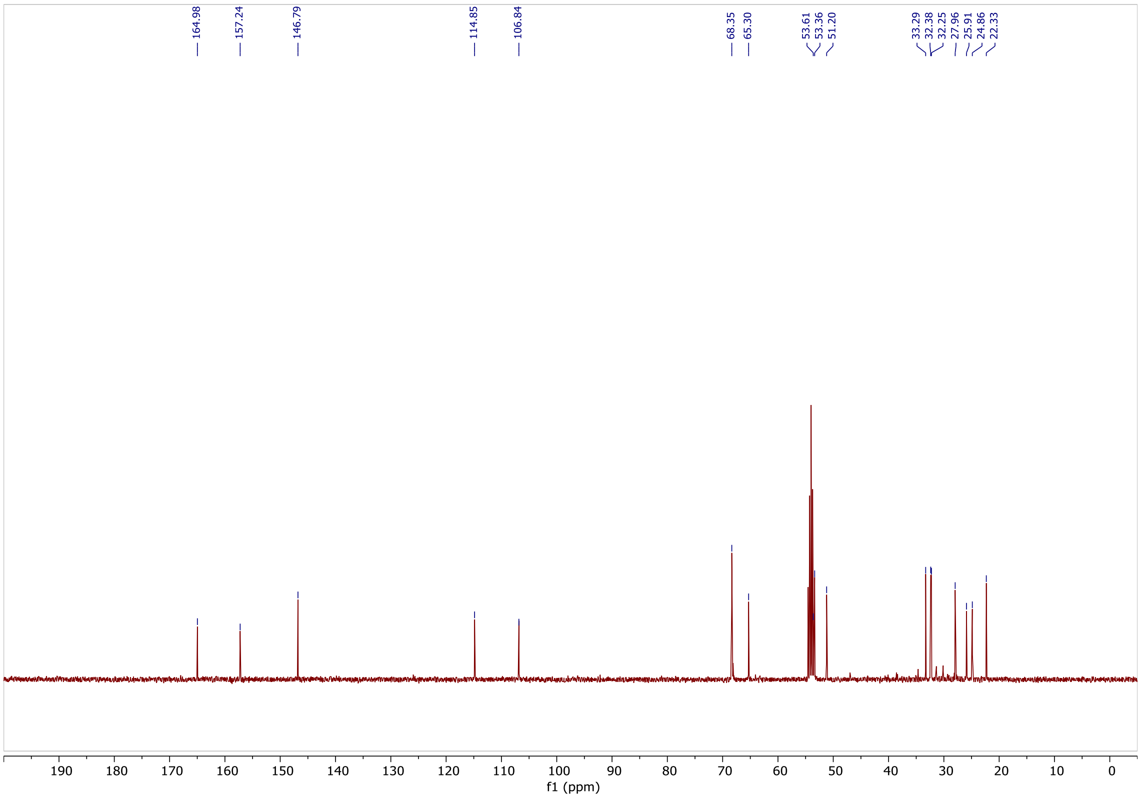

400 MHz ^{1}H NMR spectrum; 100.6 MHz ^{13}C NMR spectrum; $CDCl_3$ of **81**

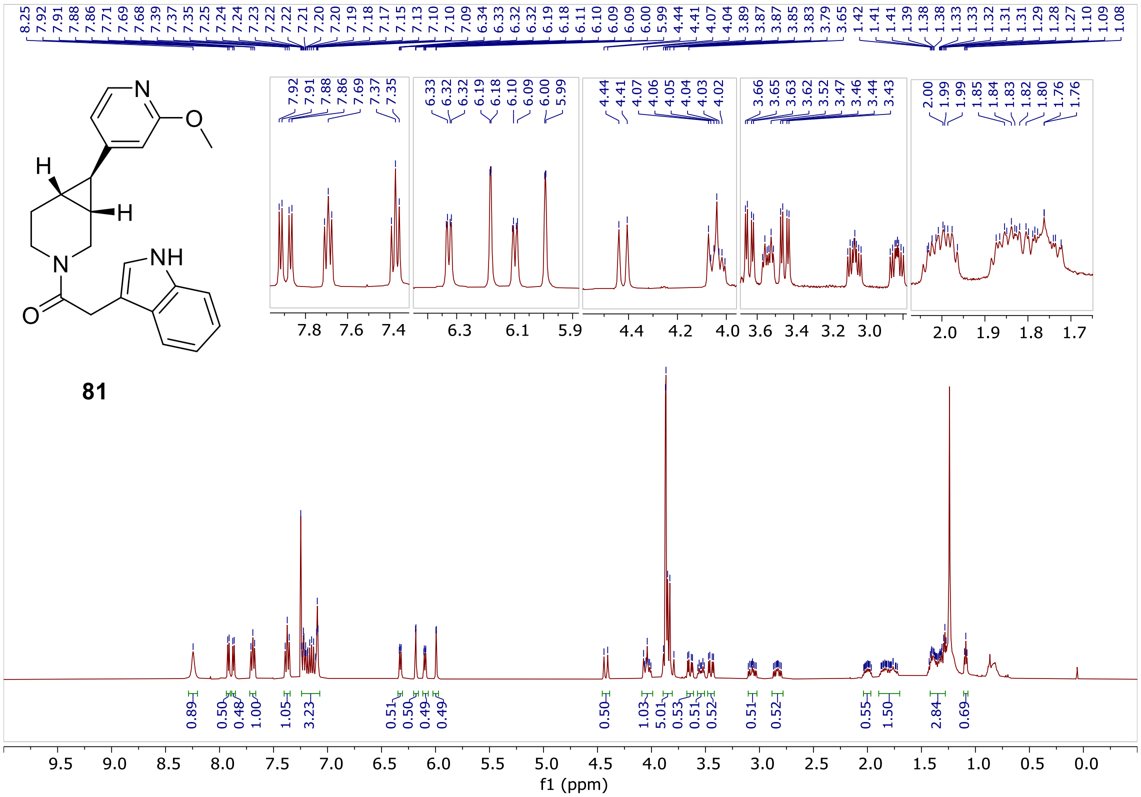

81

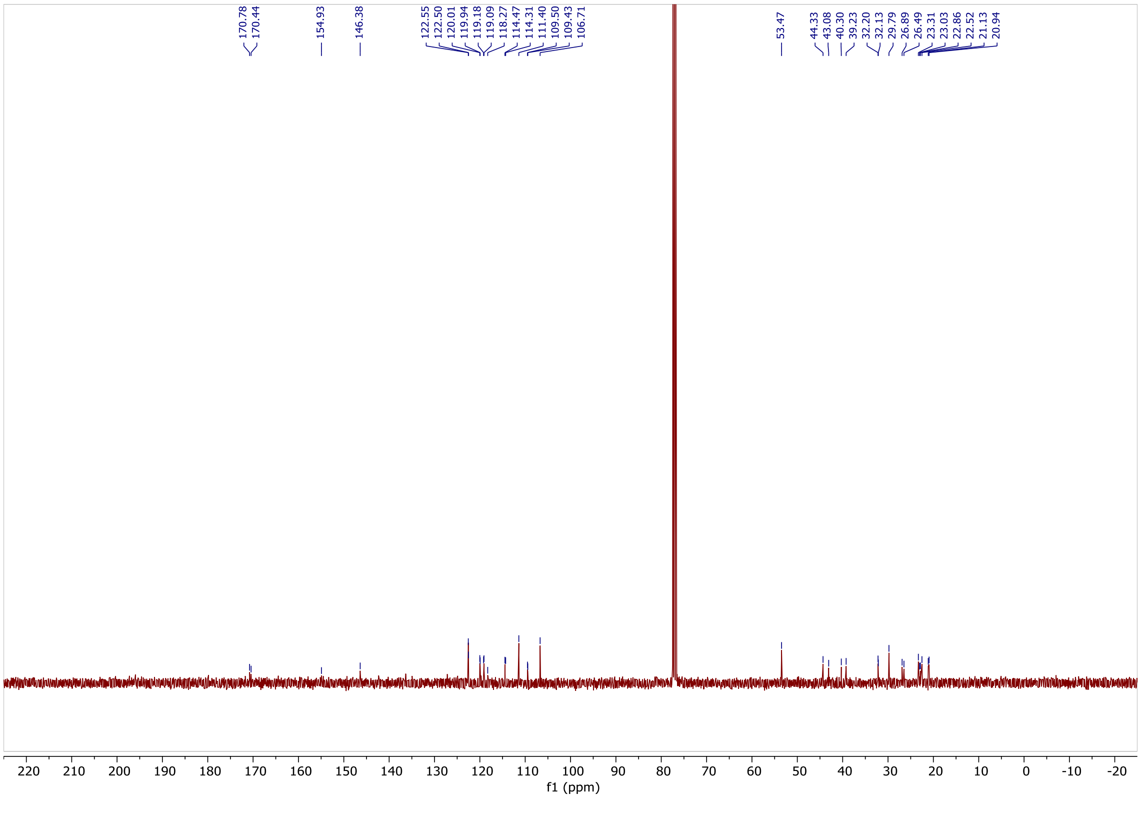

400 MHz ^{1}H NMR spectrum; 100.6 MHz ^{13}C NMR spectrum; $CDCl_3$ of **82**

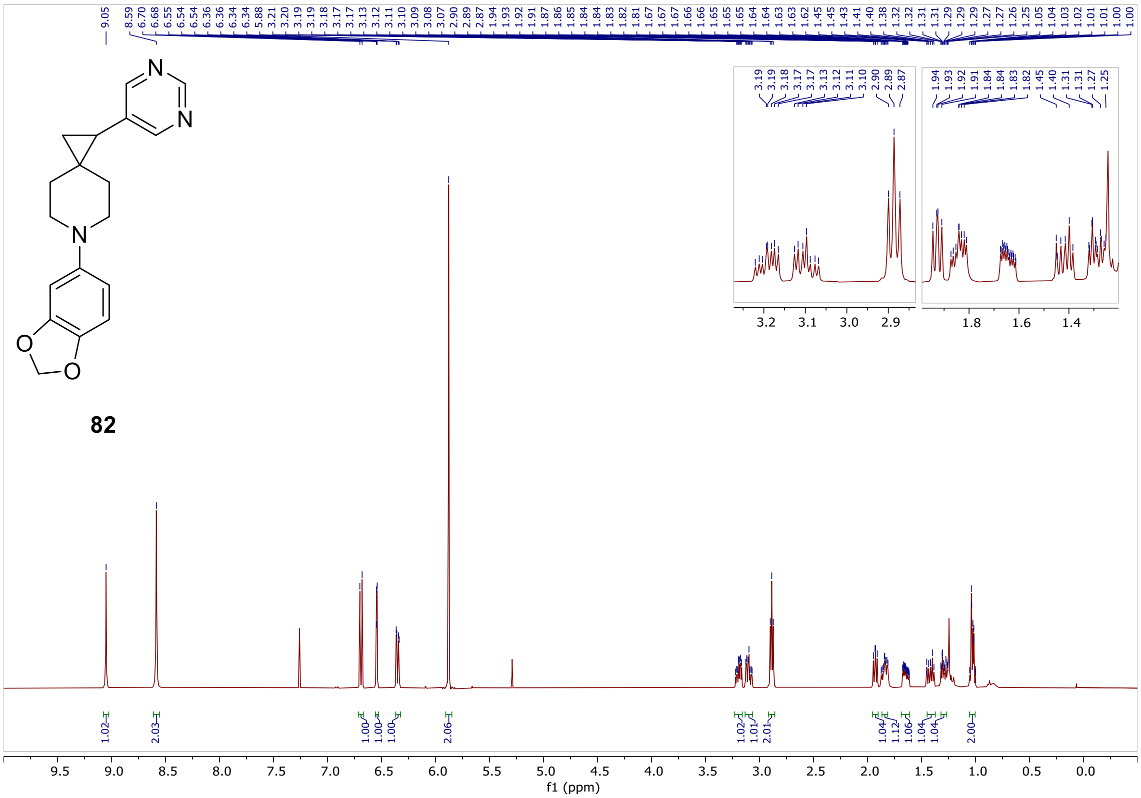

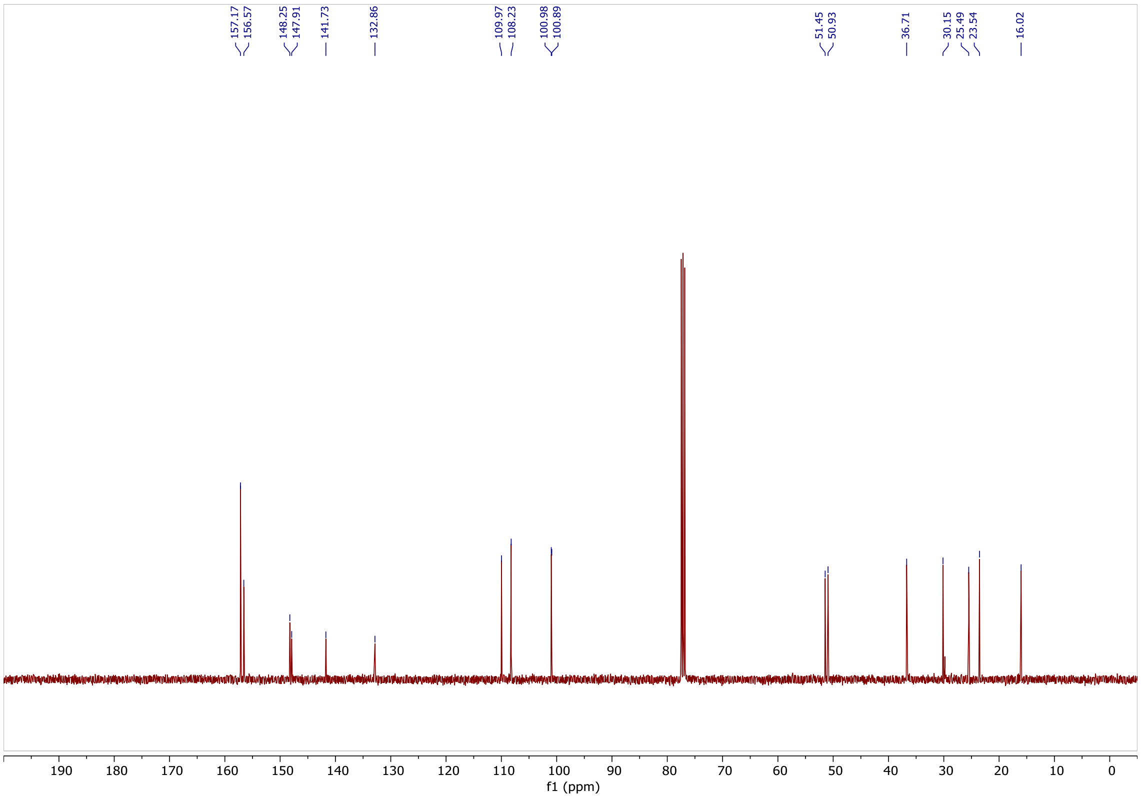

400 MHz ^{1}H NMR spectrum; 100.6 MHz ^{13}C NMR spectrum; $CDCl_3$ of **83**

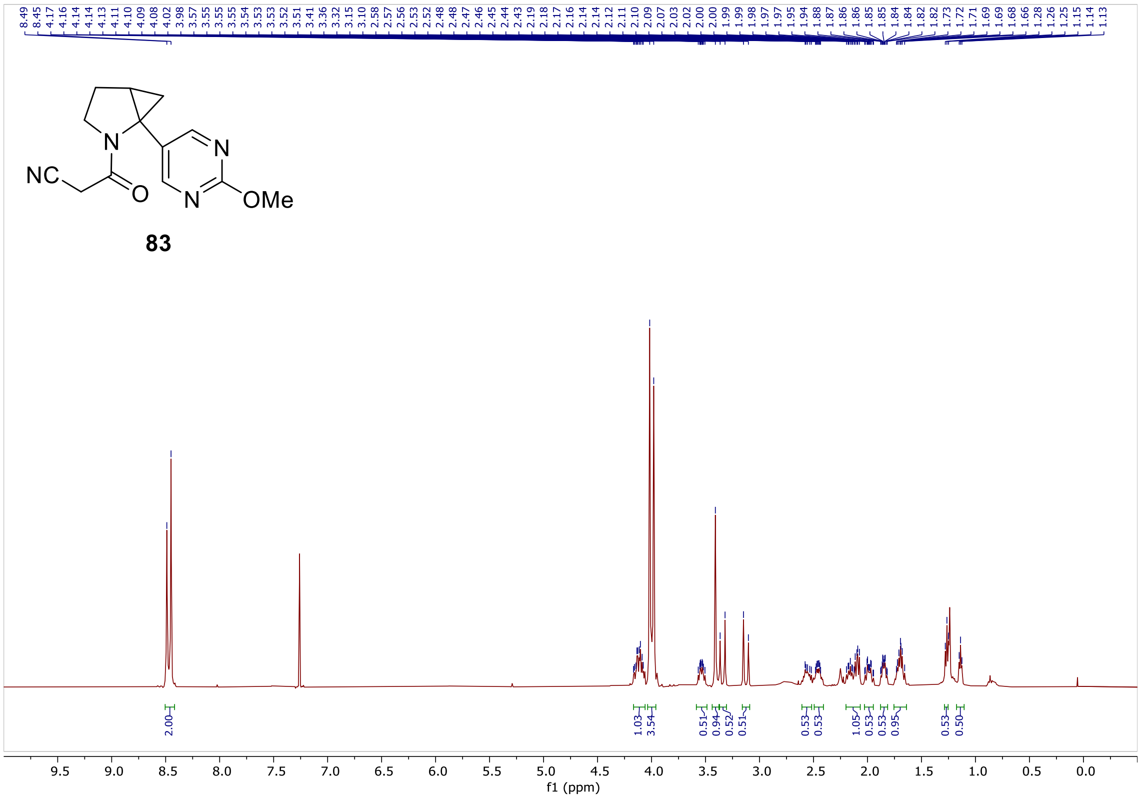

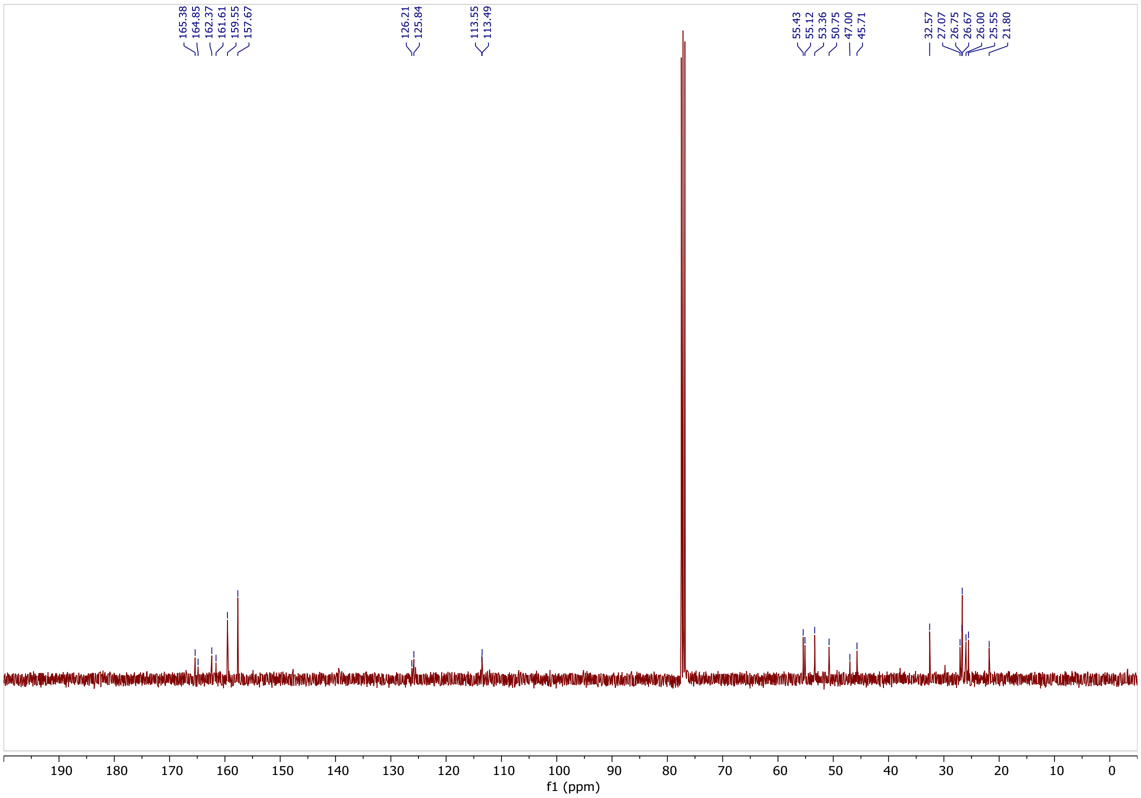

400 MHz ^{1}H NMR spectrum; $CDCl_3$ of **85**

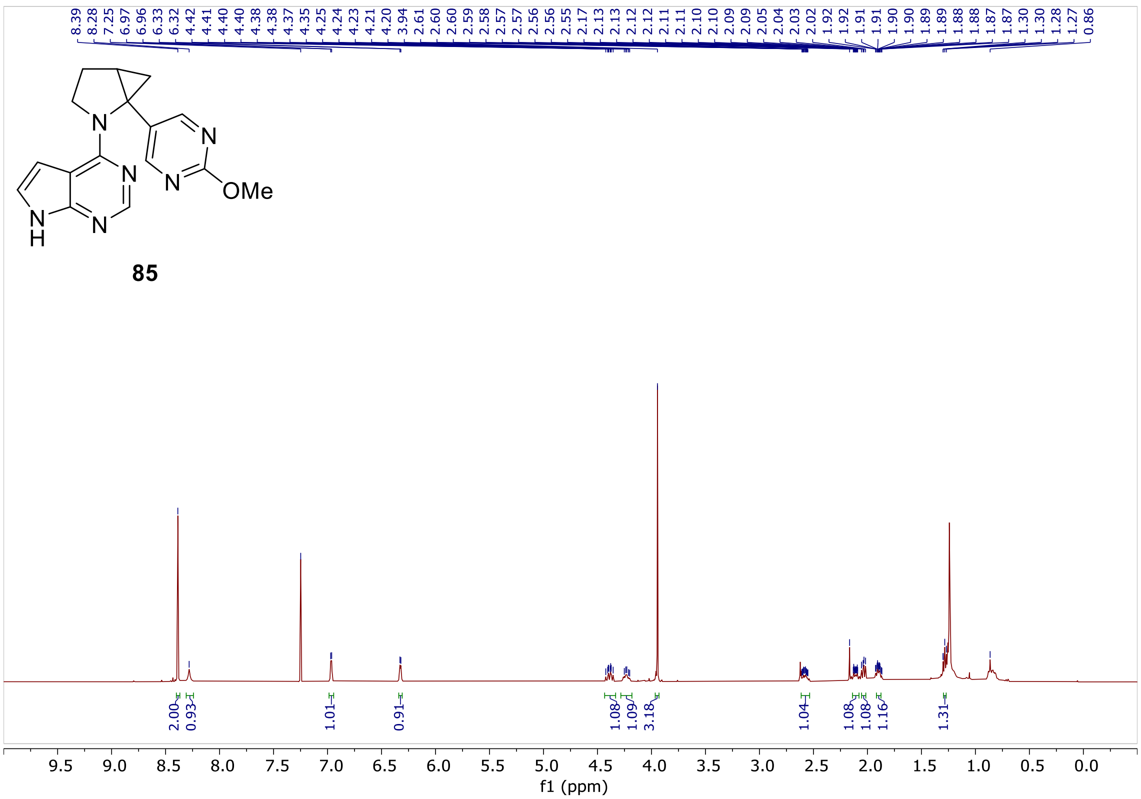

100.6 MHz ^{13}C NMR spectrum; Acetone-*d*6 of **85**

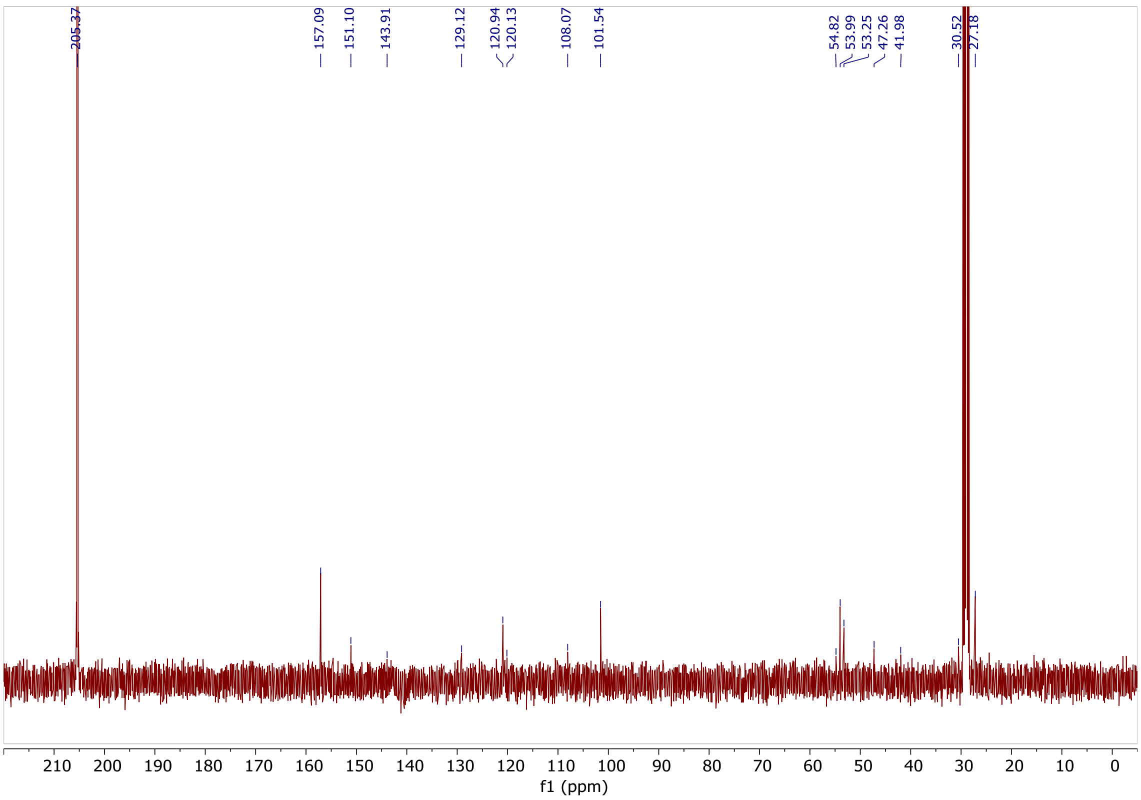

400 MHz ^{1}H NMR spectrum; 100.6 MHz ^{13}C NMR spectrum; $CDCl_3$ of **86**

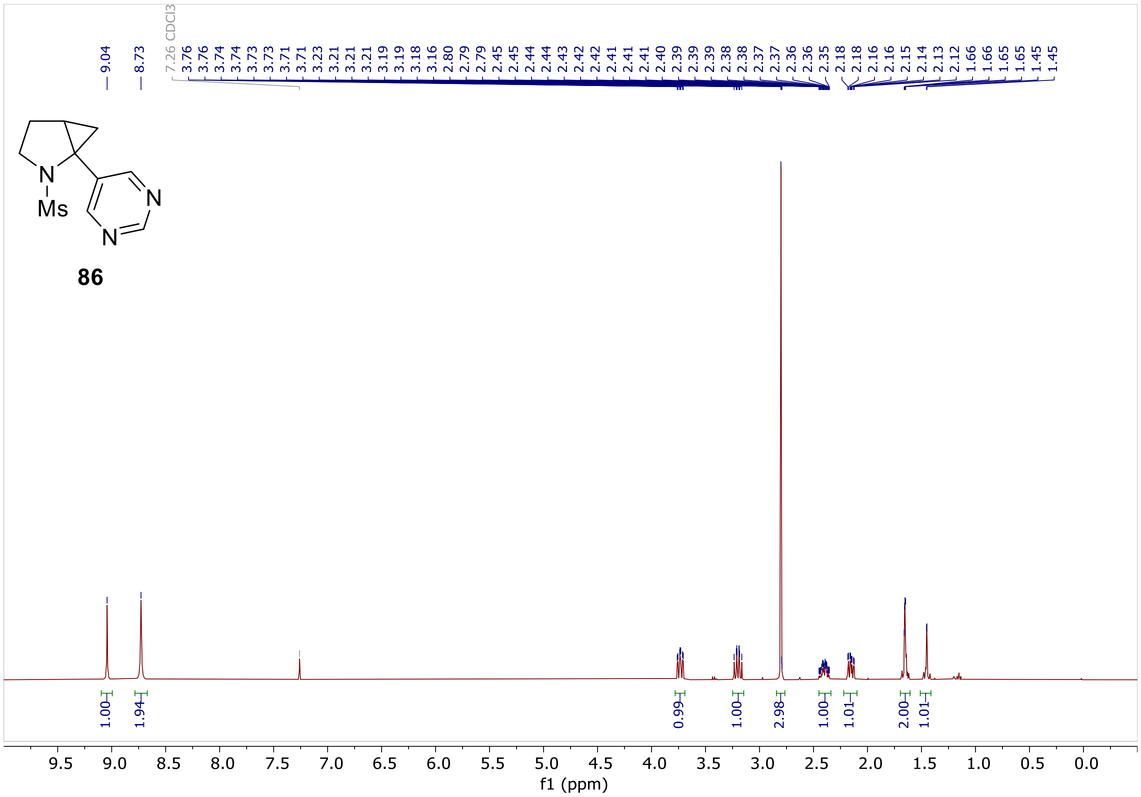

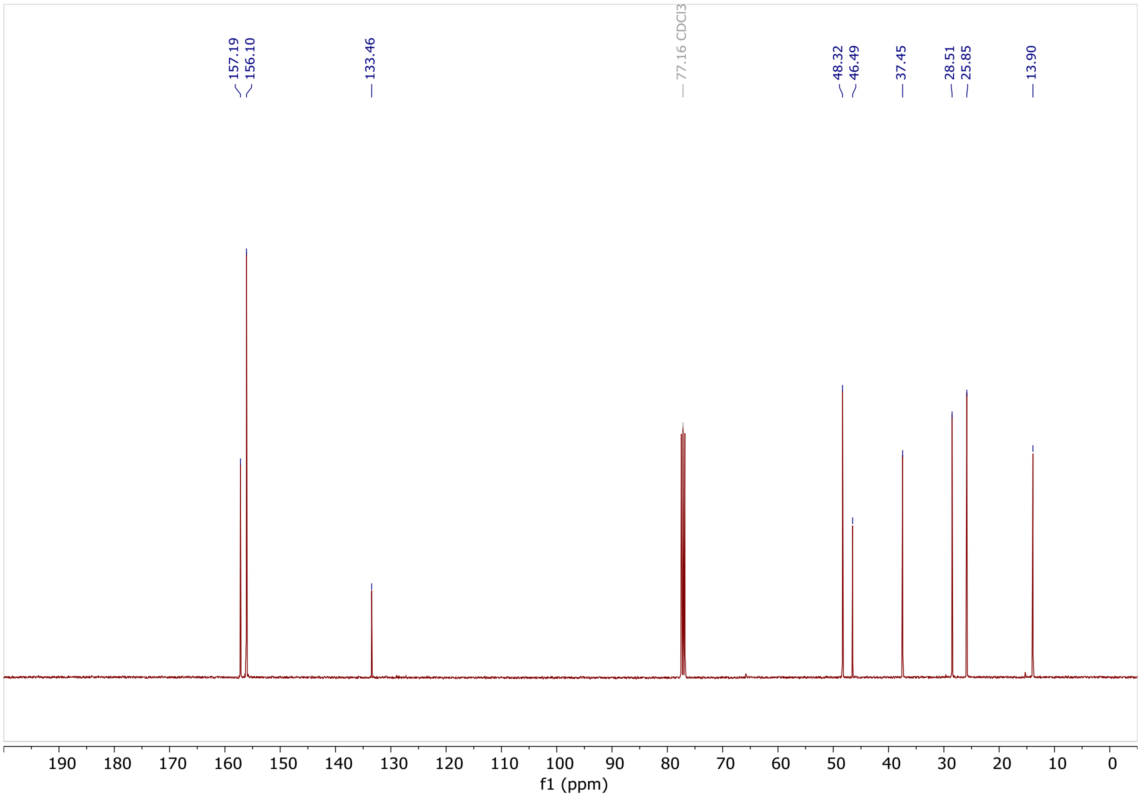

400 MHz ^{1}H NMR spectrum; 100.6 MHz ^{13}C NMR spectrum; $CDCl_3$ of **87**

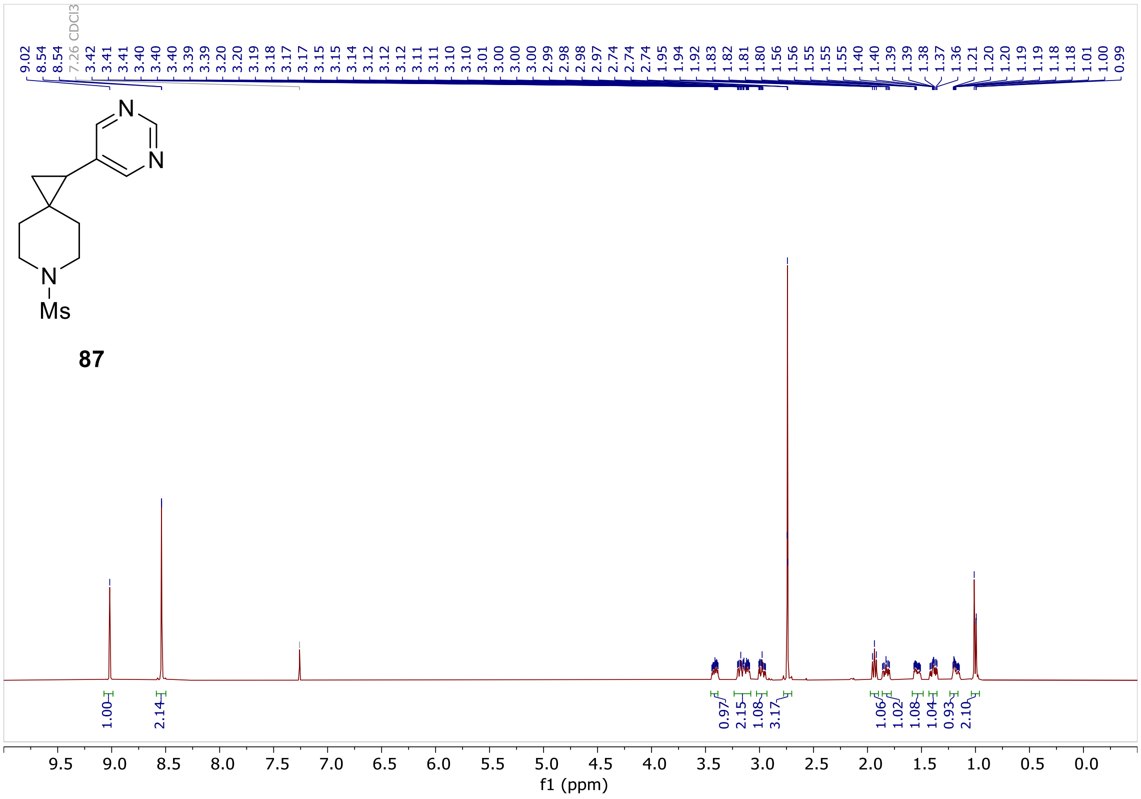

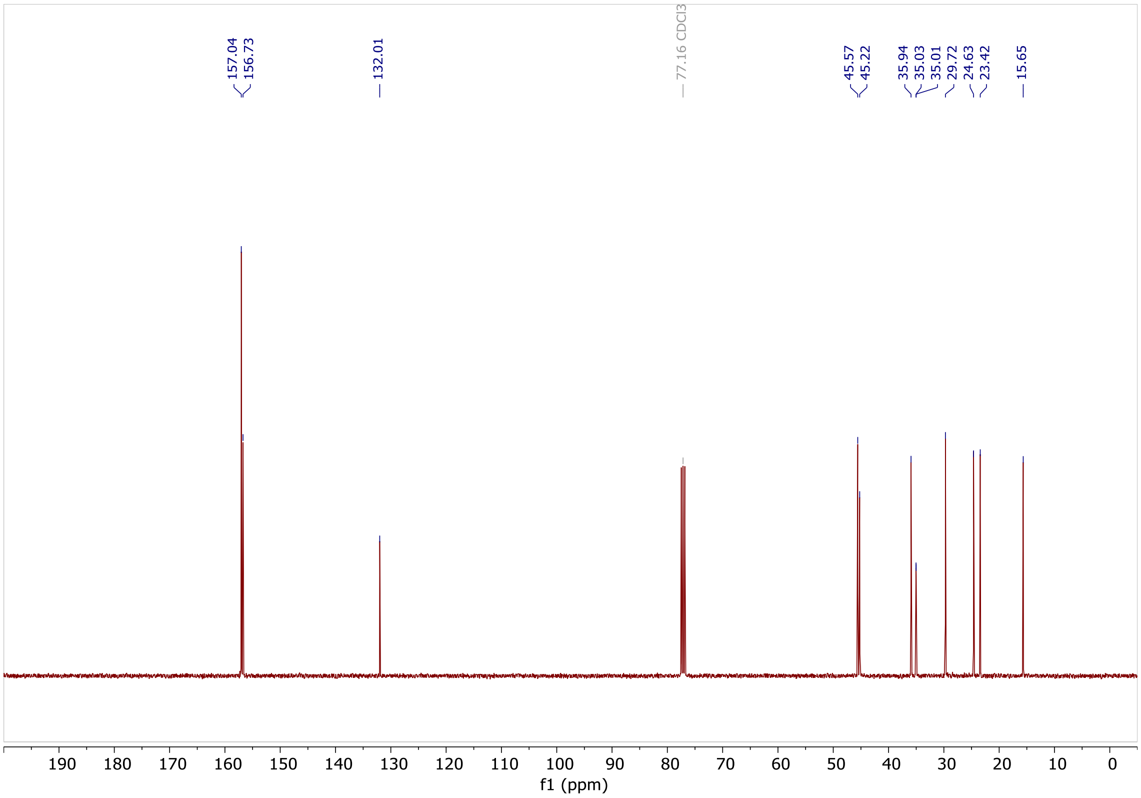

400 MHz ^{1}H NMR spectrum; 100.6 MHz ^{13}C NMR spectrum; $CDCl_3$ of **88**

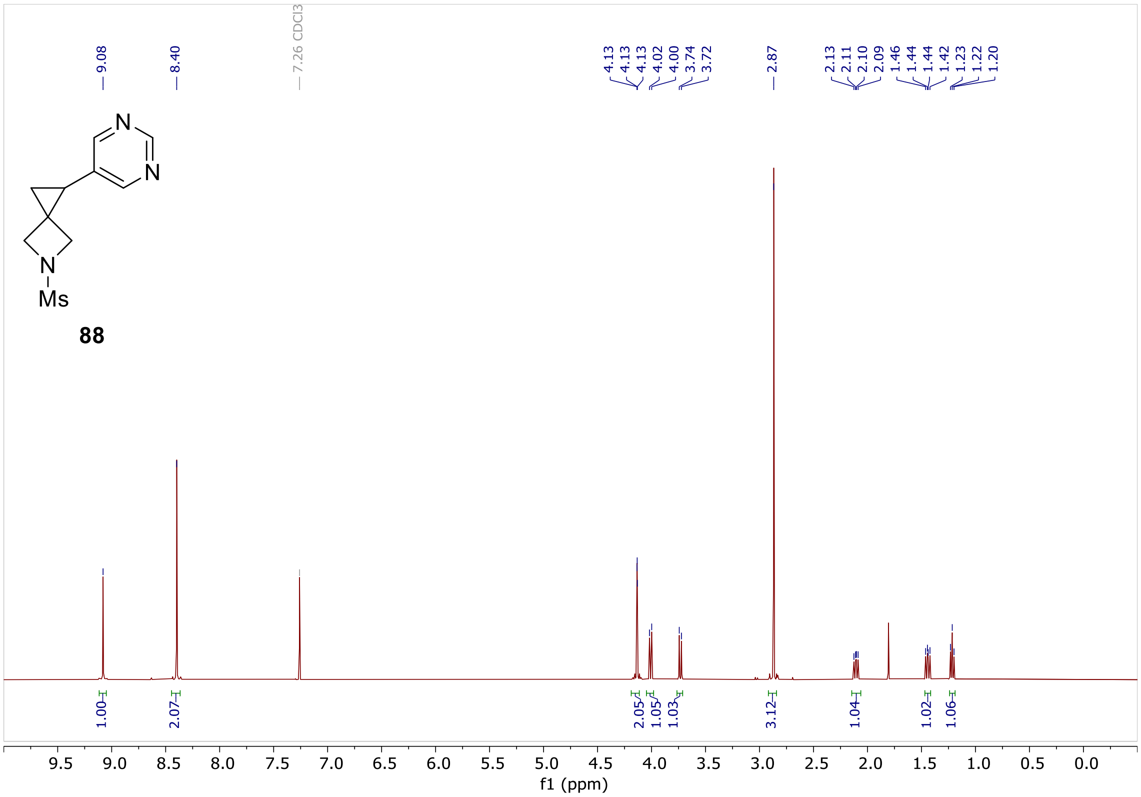

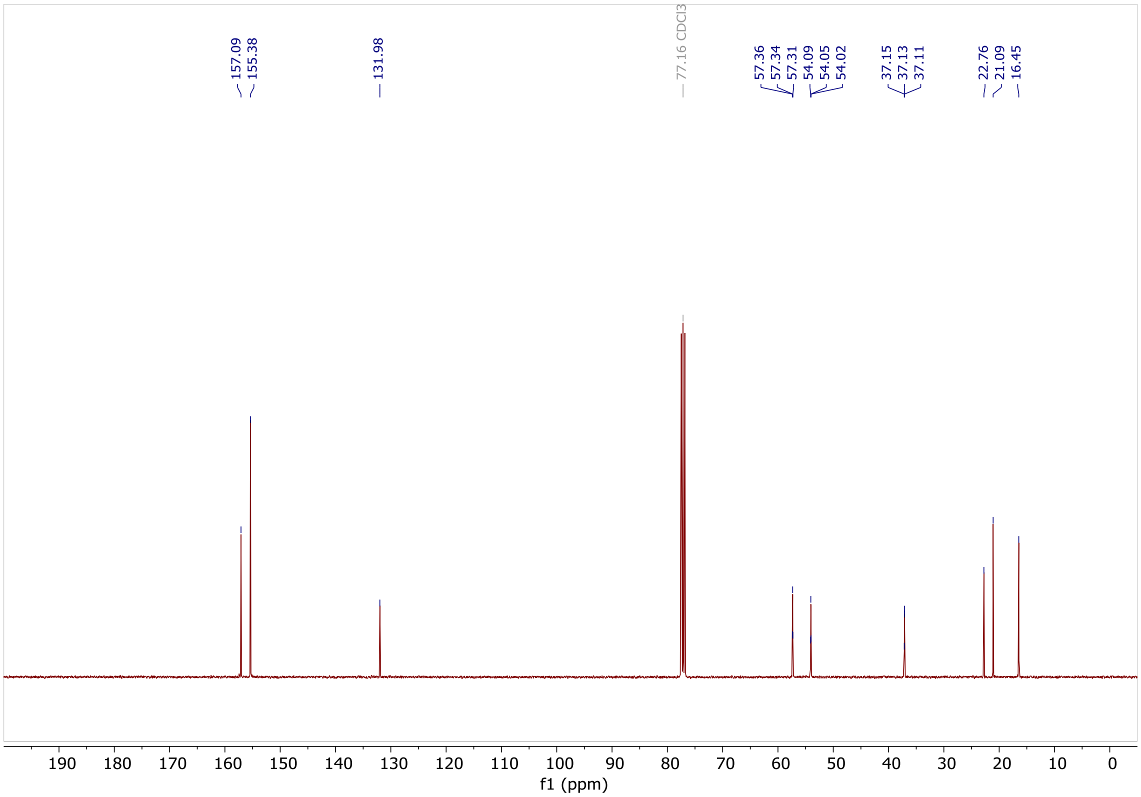

400 MHz ^{1}H NMR spectrum; 100.6 MHz ^{13}C NMR spectrum; $CDCl_3$ of **89**

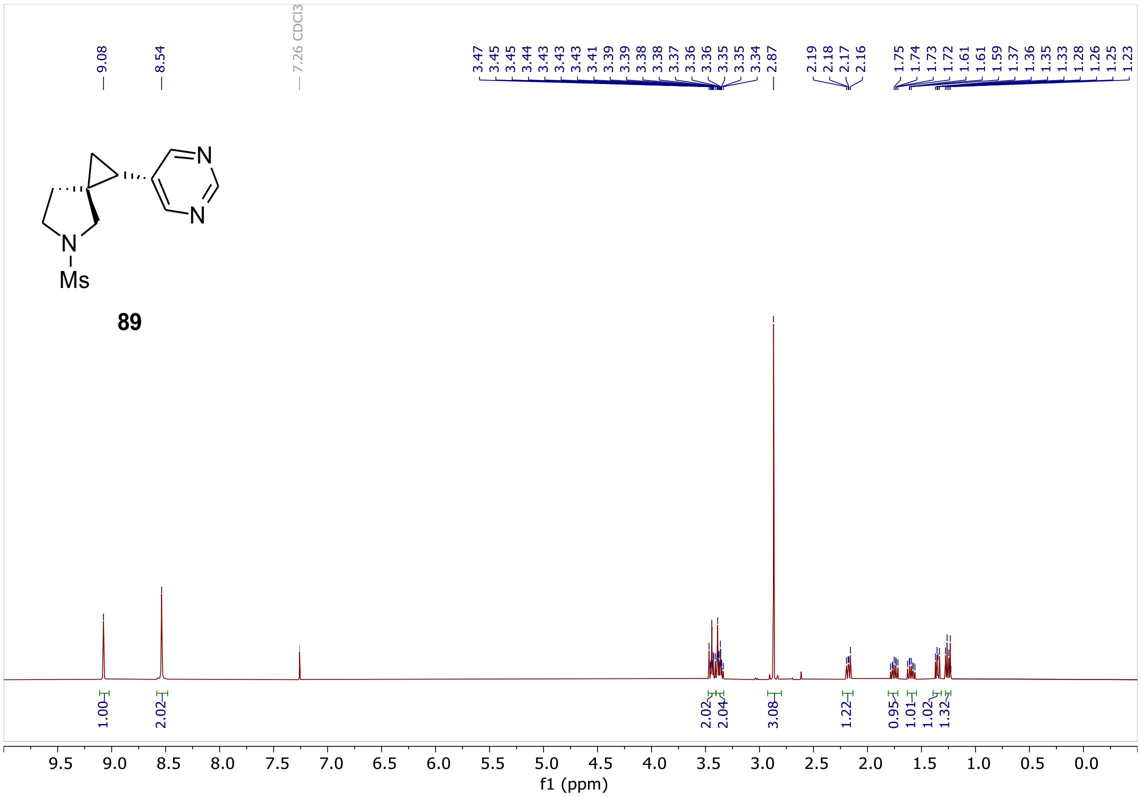

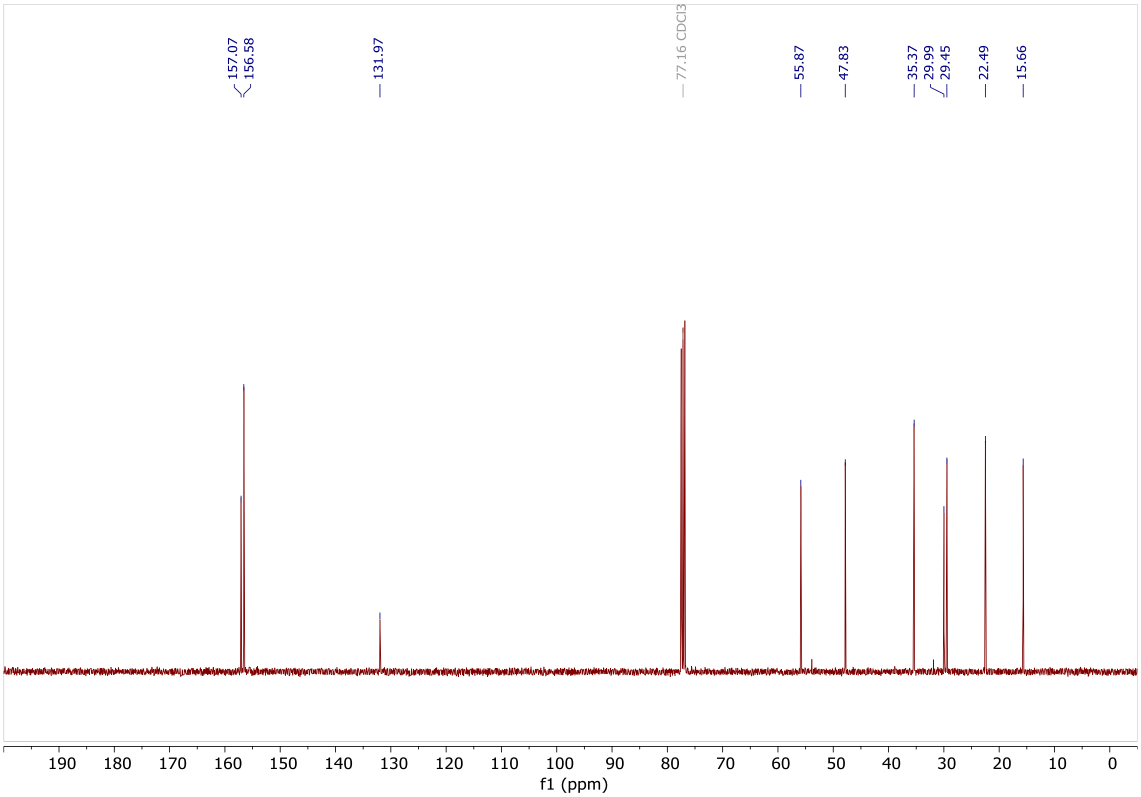

400 MHz ^{1}H NMR spectrum; 100.6 MHz ^{13}C NMR spectrum; $CDCl_3$ of **90**

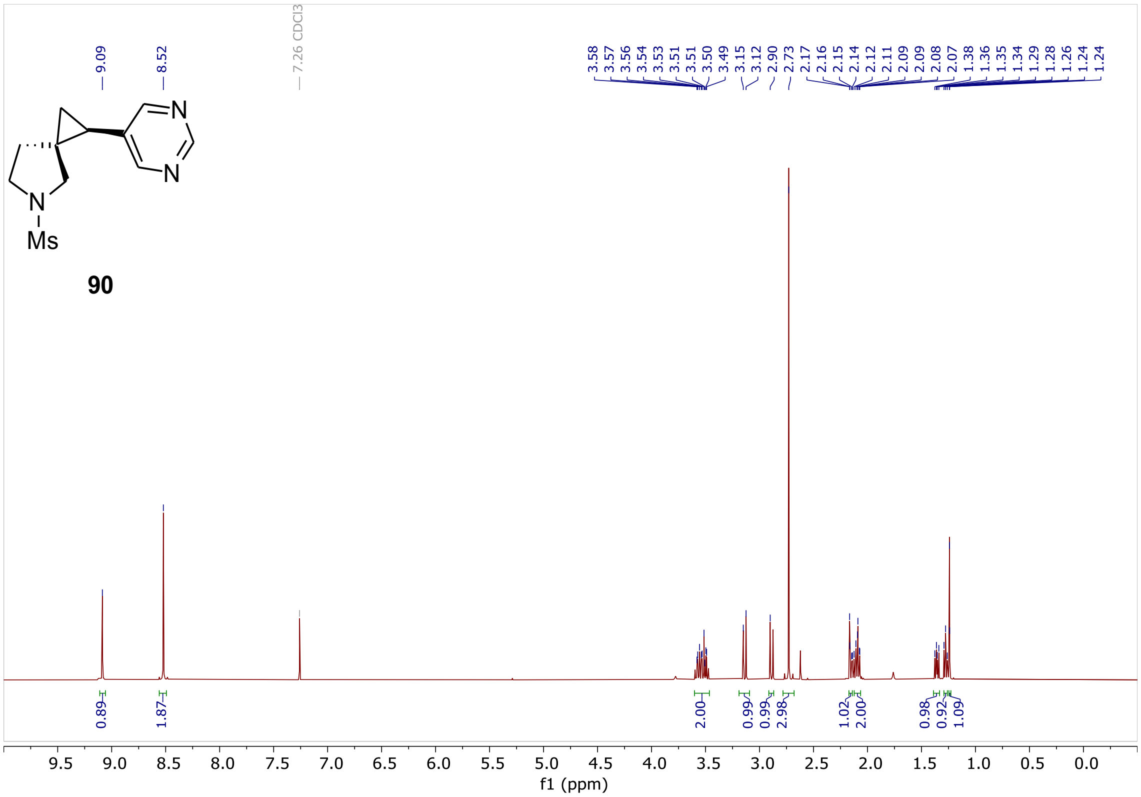

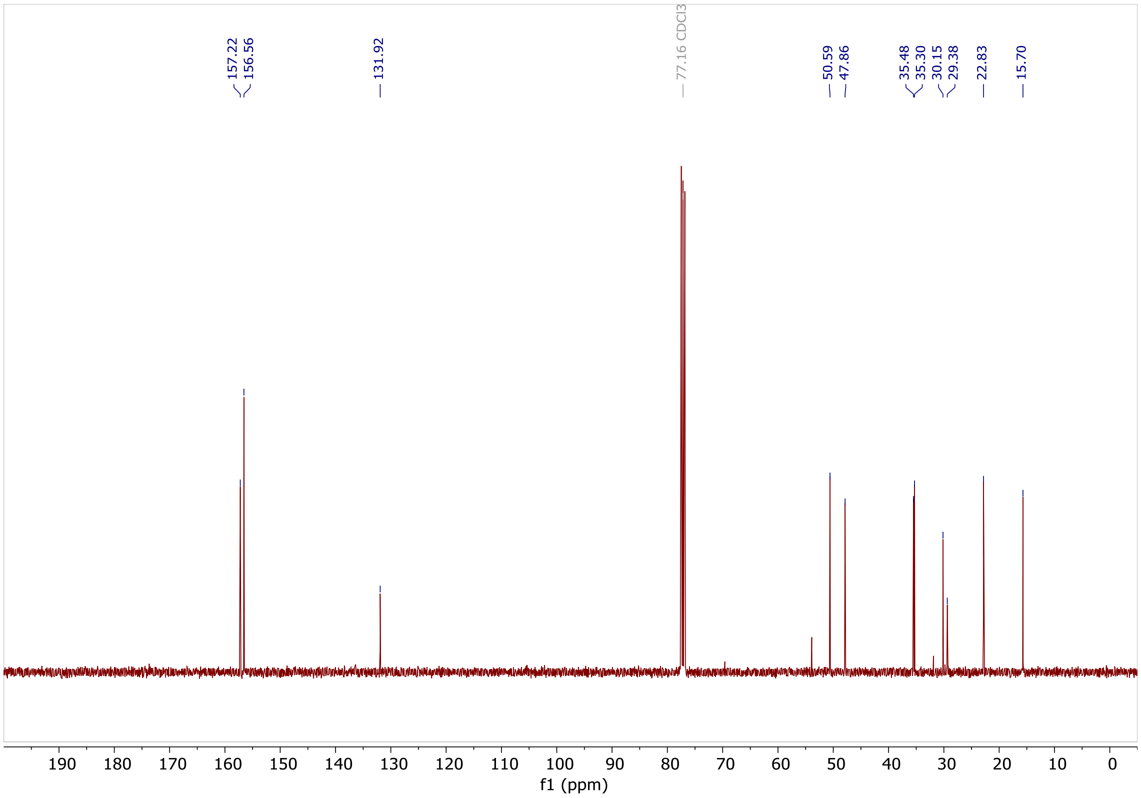

400 MHz ^{1}H NMR spectrum; 100.6 MHz ^{13}C NMR spectrum; DMSO-d_6 of **91**

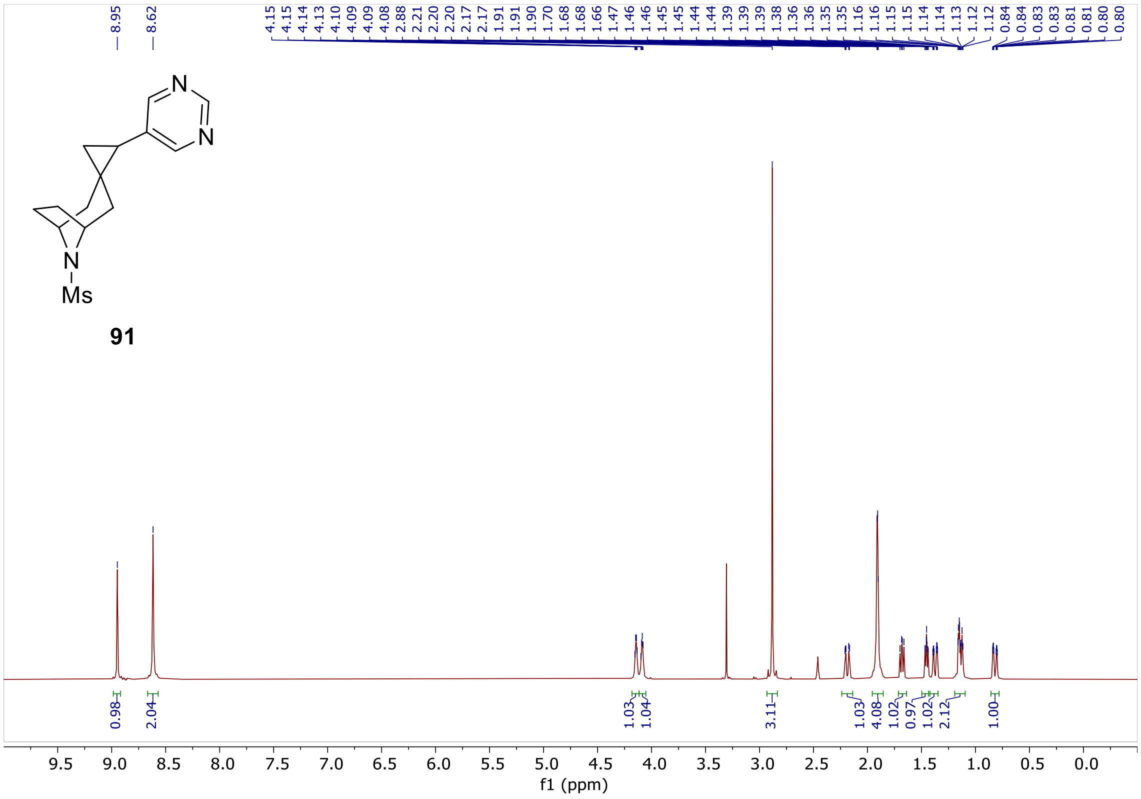

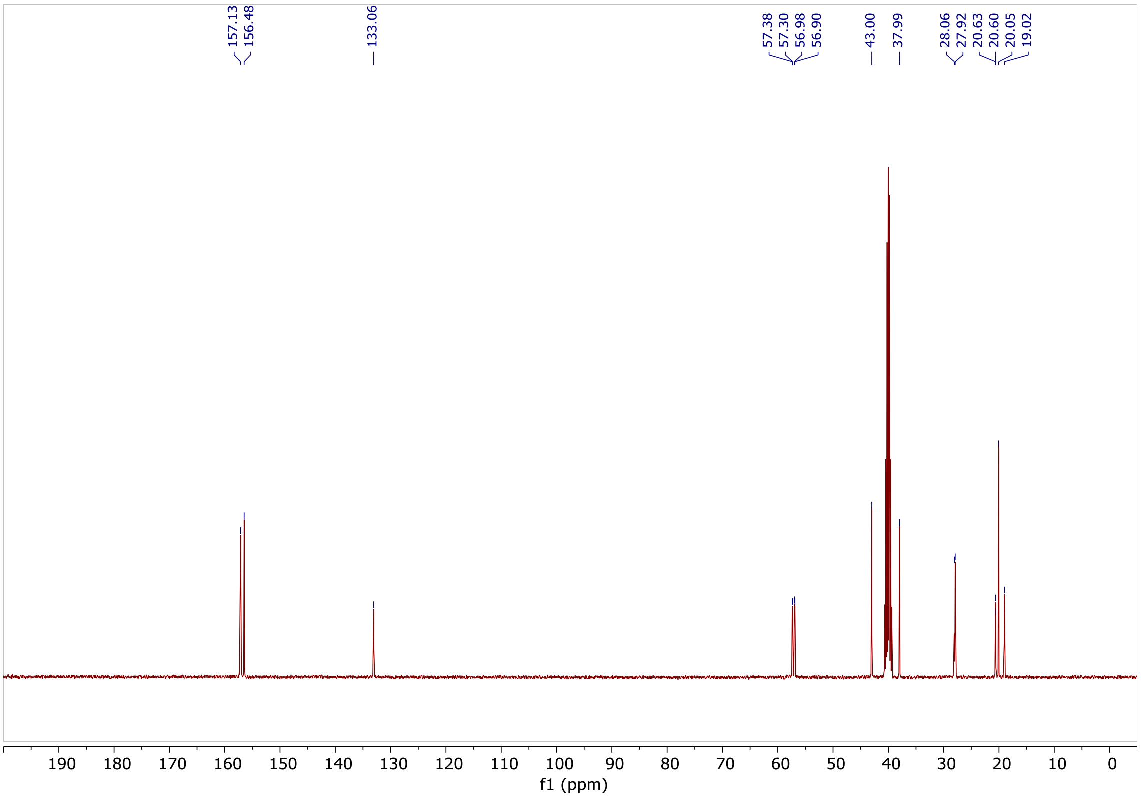

400 MHz ^{1}H NMR spectrum; 100.6 MHz ^{13}C NMR spectrum; acetone-d_6 of **92**

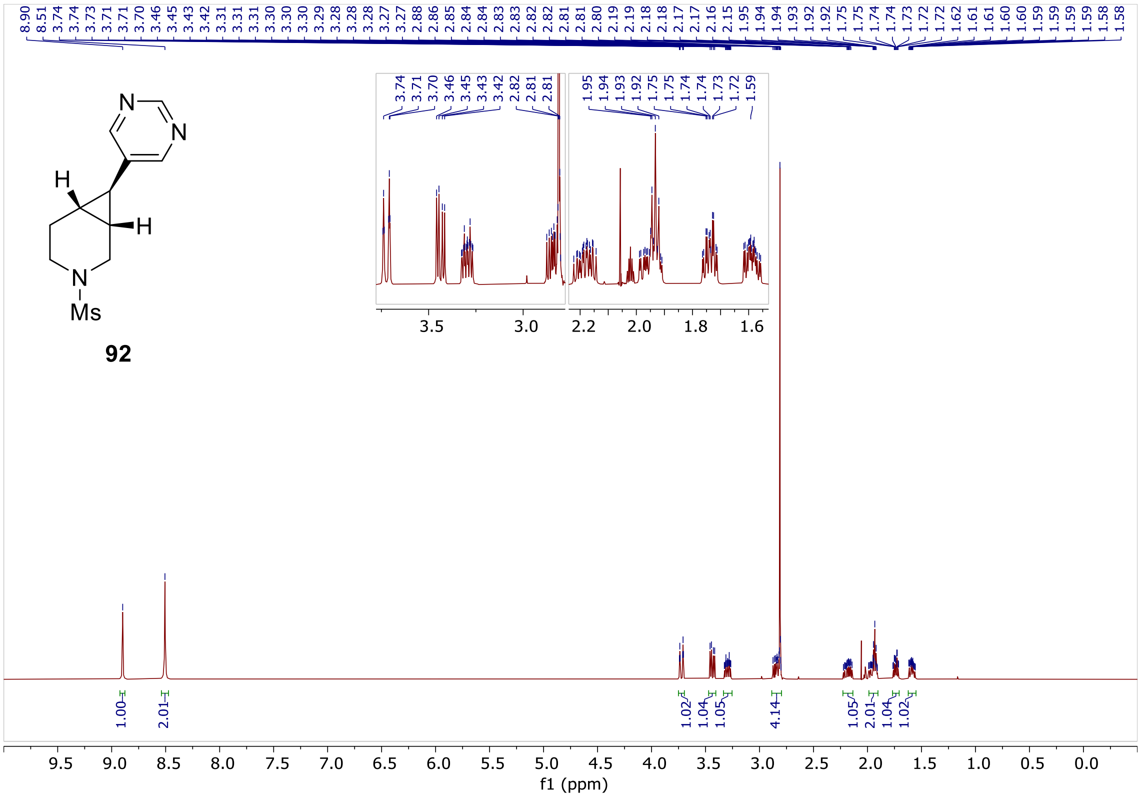

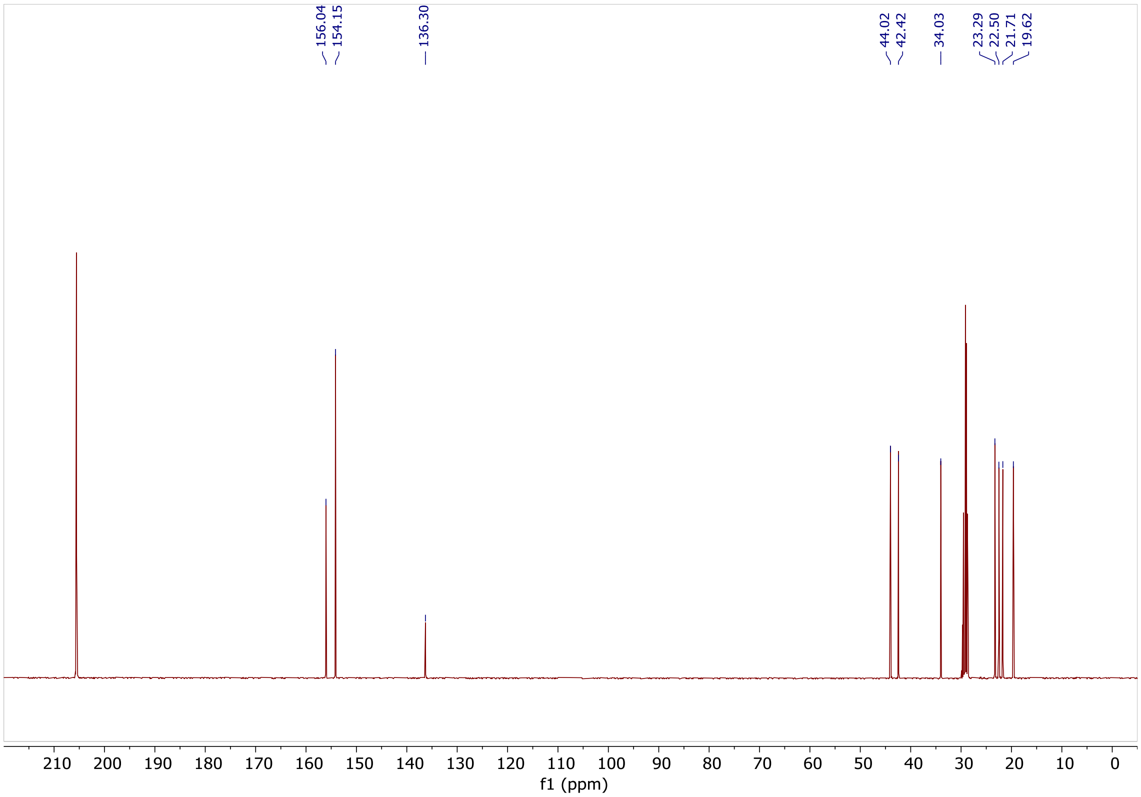

400 MHz ^{1}H NMR spectrum; 100.6 MHz ^{13}C NMR spectrum; $CDCl_3$ of **93**

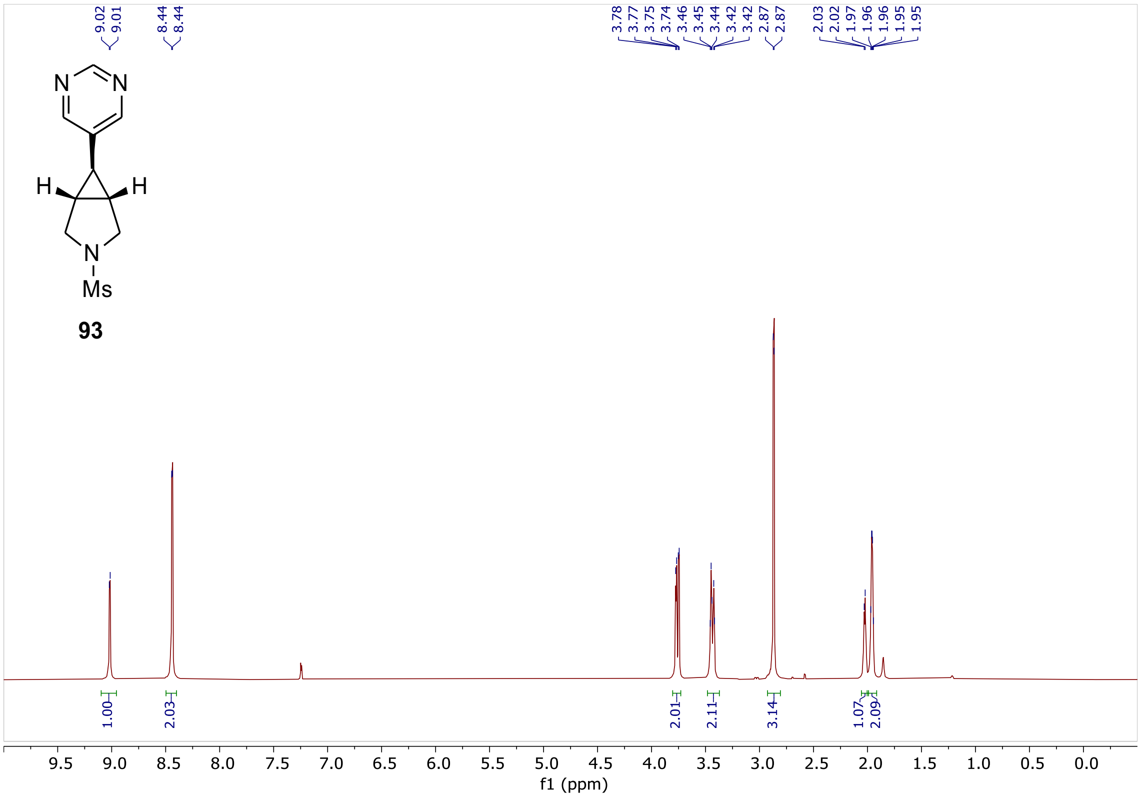

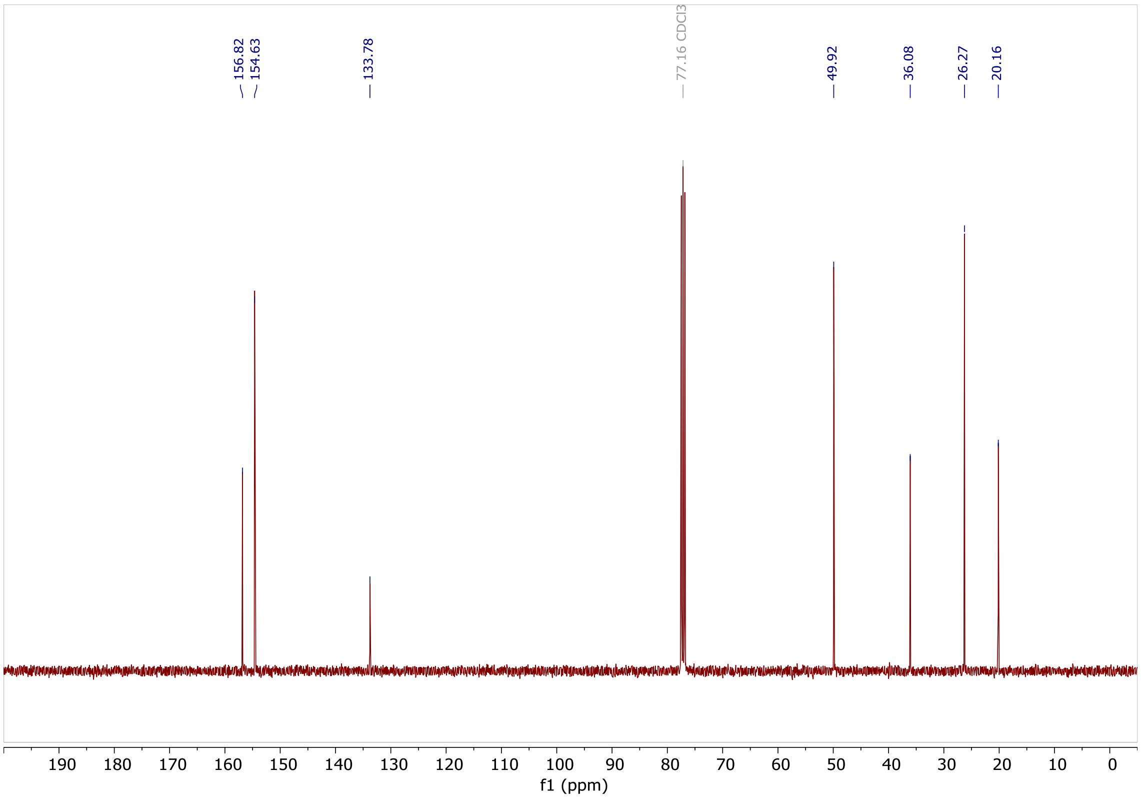

400 MHz ^{1}H NMR spectrum; 100.6 MHz ^{13}C NMR spectrum; $CDCl_3$ of **94**

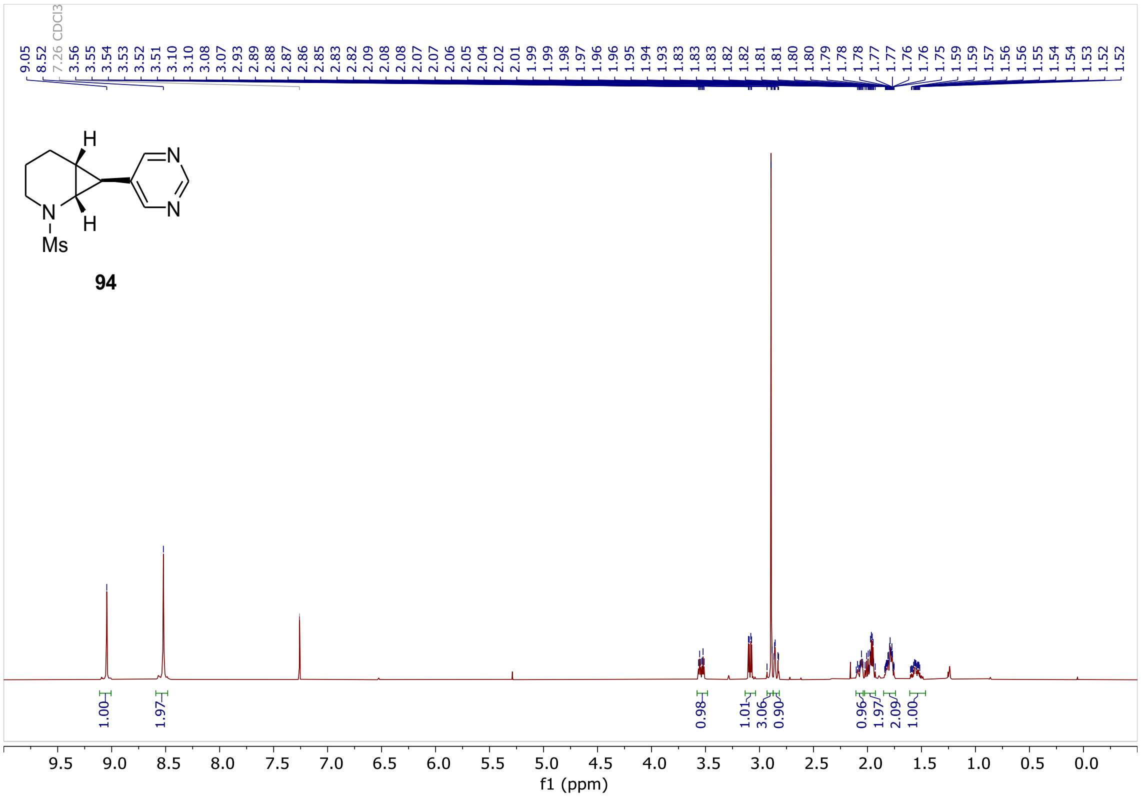

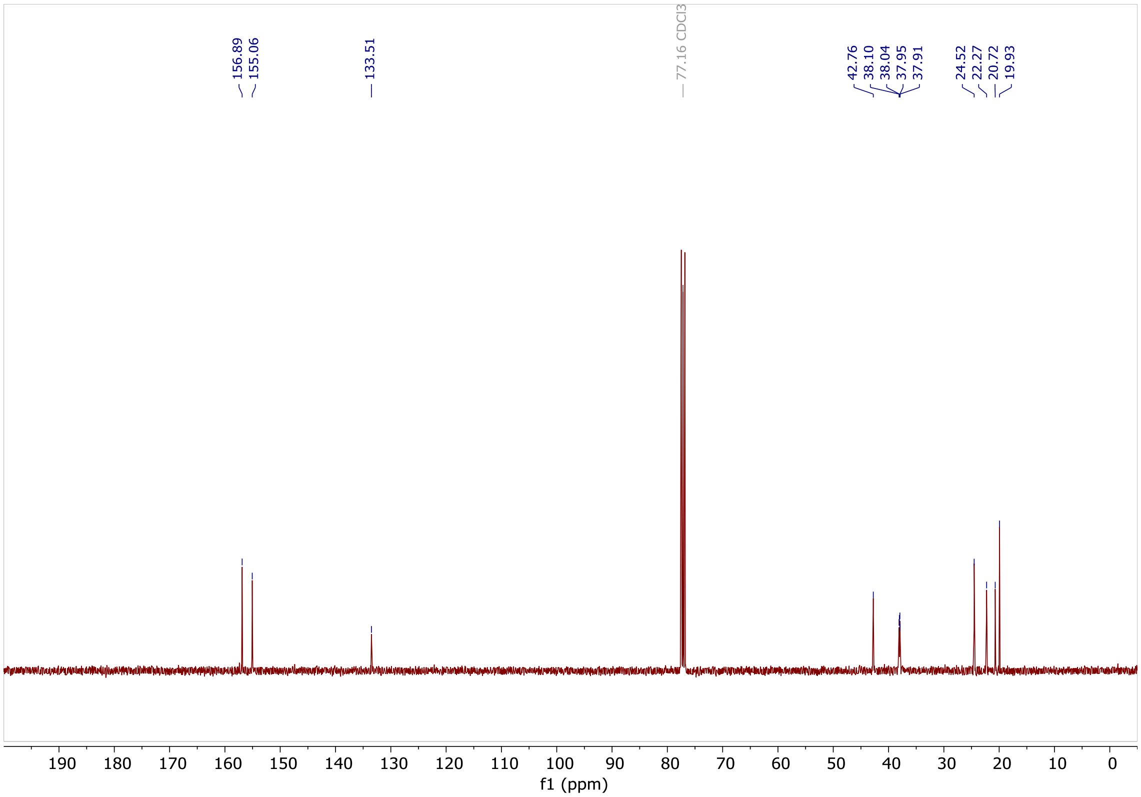

400 MHz ^{1}H NMR spectrum; 100.6 MHz ^{13}C NMR spectrum; $CDCl_3$ of **S34**

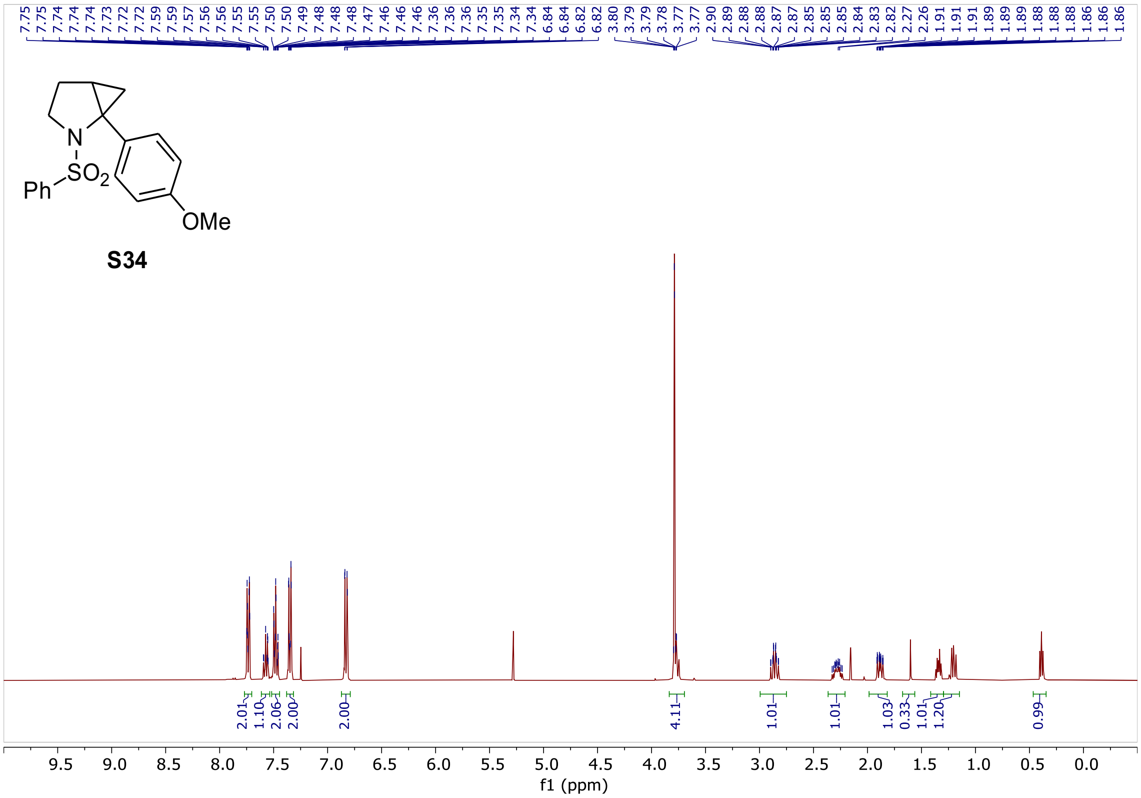

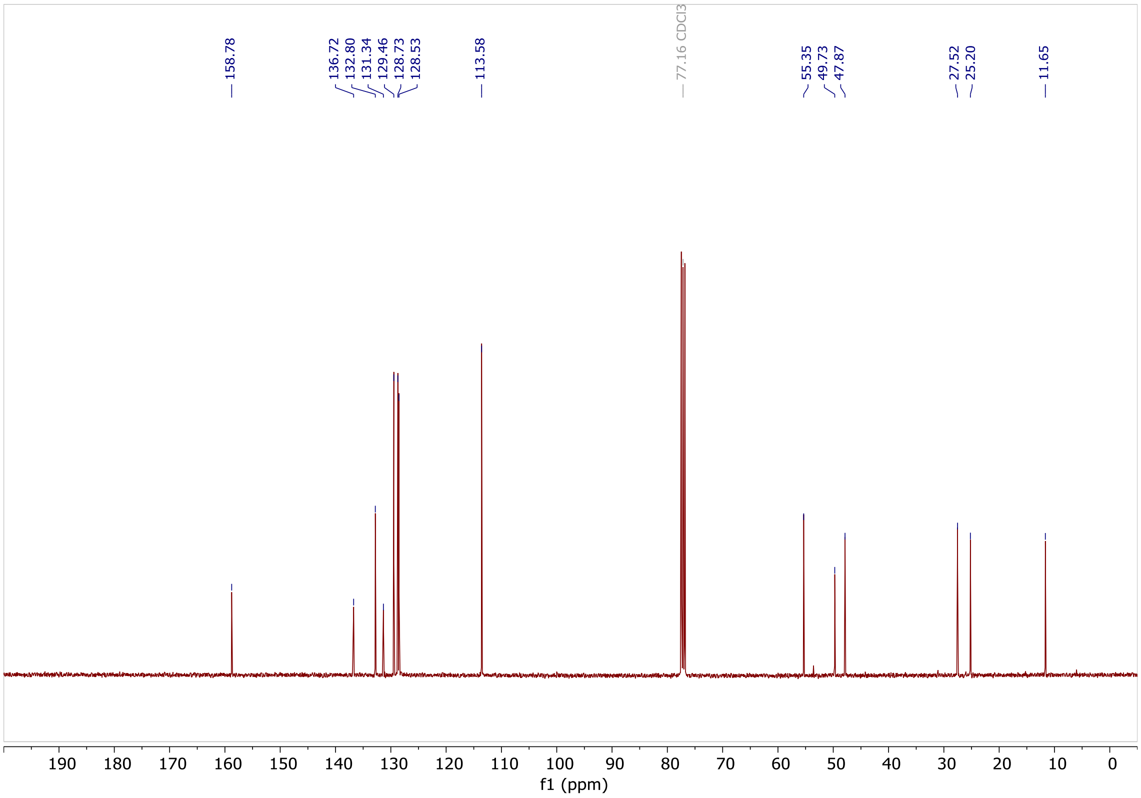

400 MHz ^{1}H NMR spectrum; 100.6 MHz ^{13}C NMR spectrum; $CDCl_3$ of **S35**

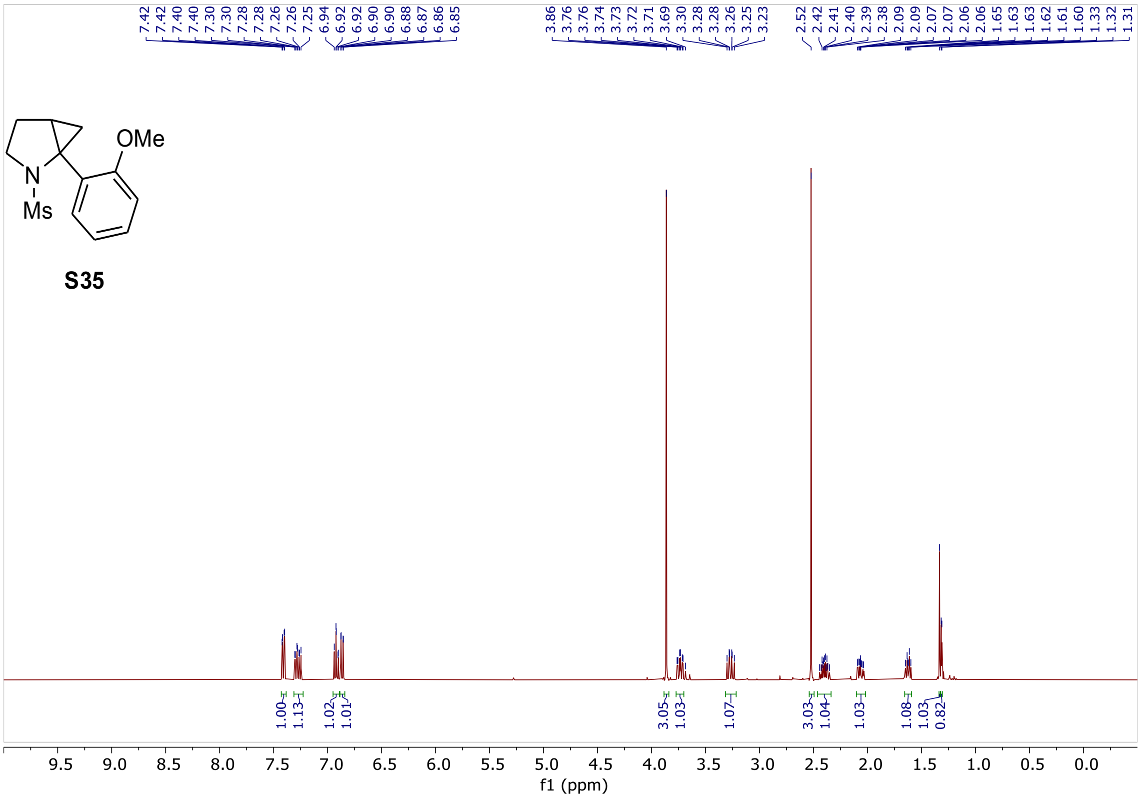

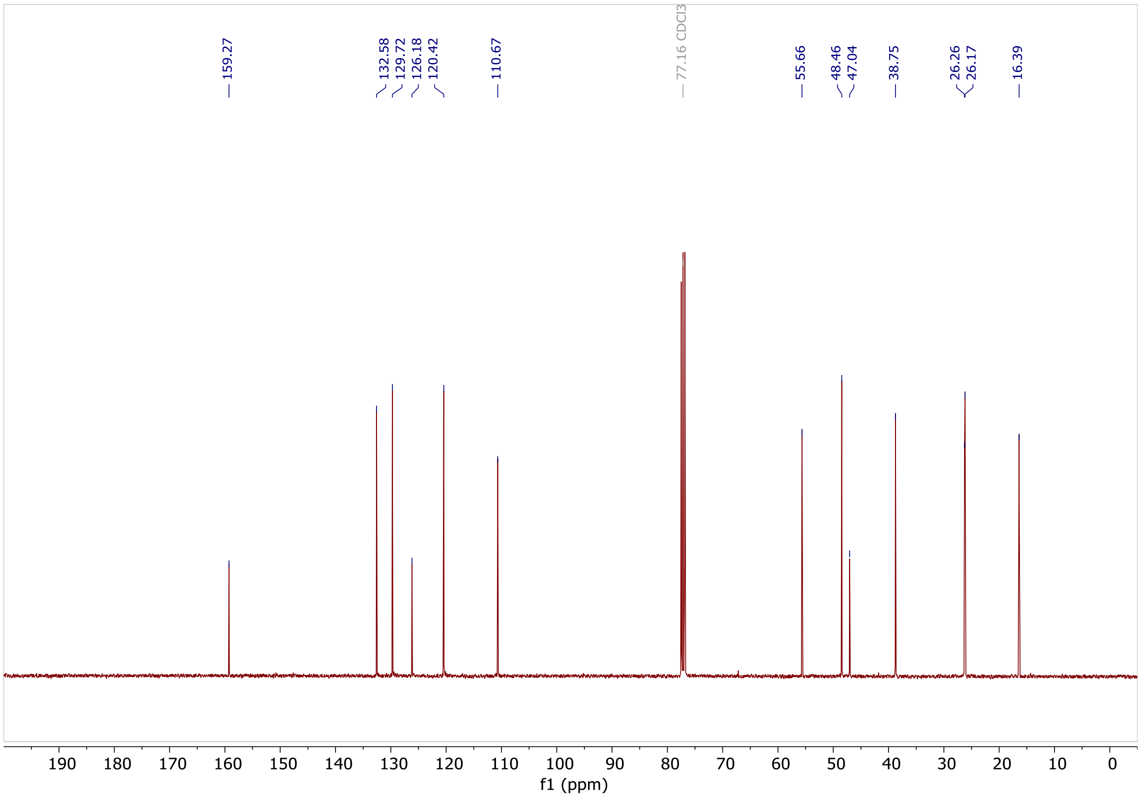

400 MHz ^{1}H NMR spectrum; 100.6 MHz ^{13}C NMR spectrum; $CDCl_3$ of **S36**

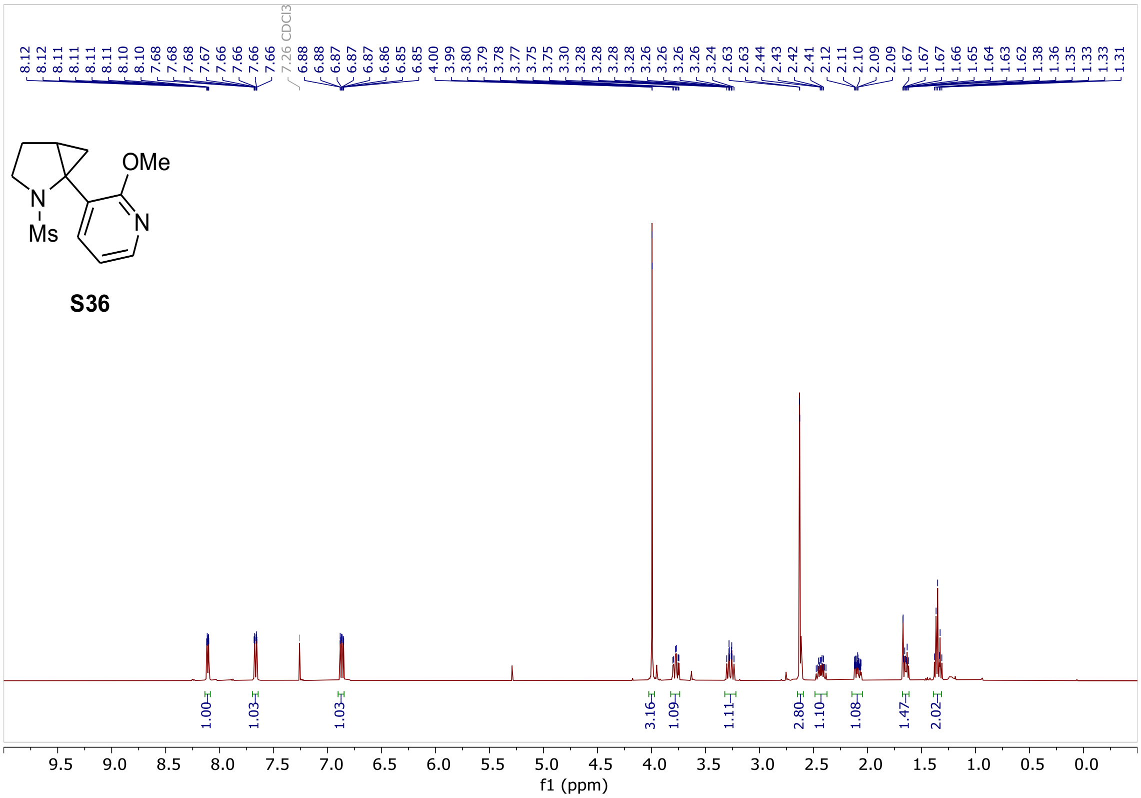

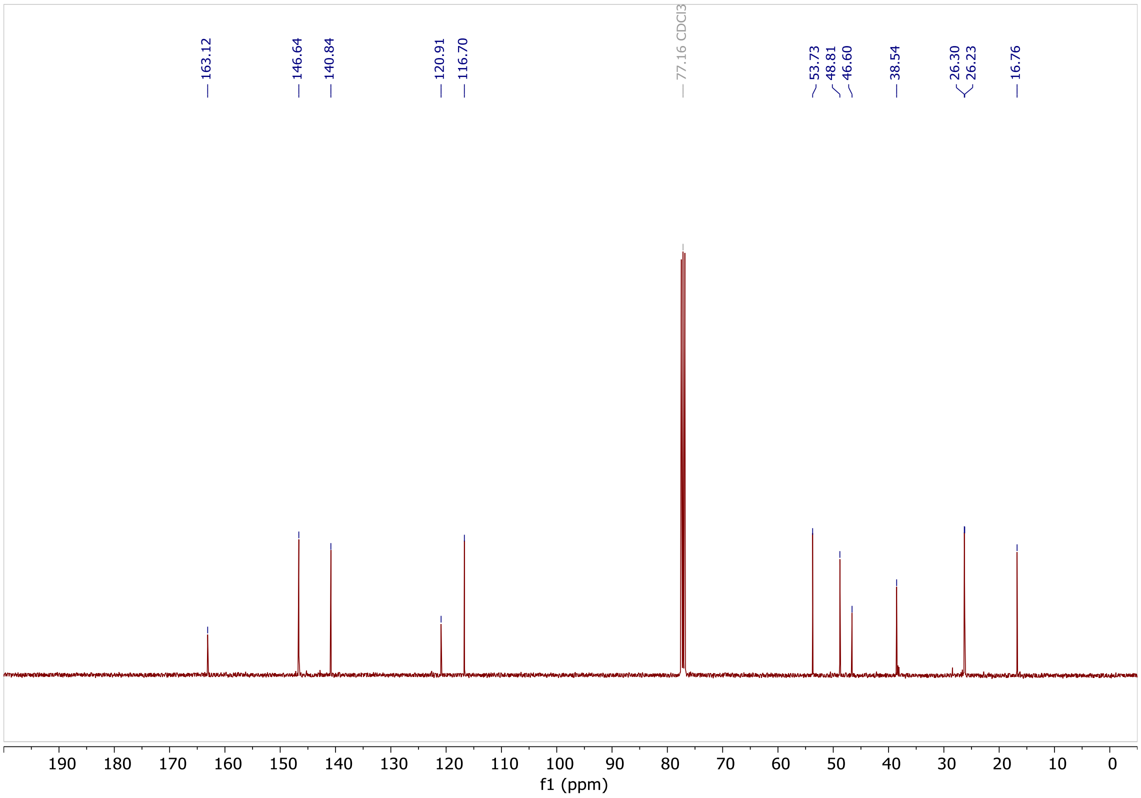

400 MHz ^{1}H NMR spectrum; 100.6 MHz ^{13}C NMR spectrum; $CDCl_3$ of **S37**

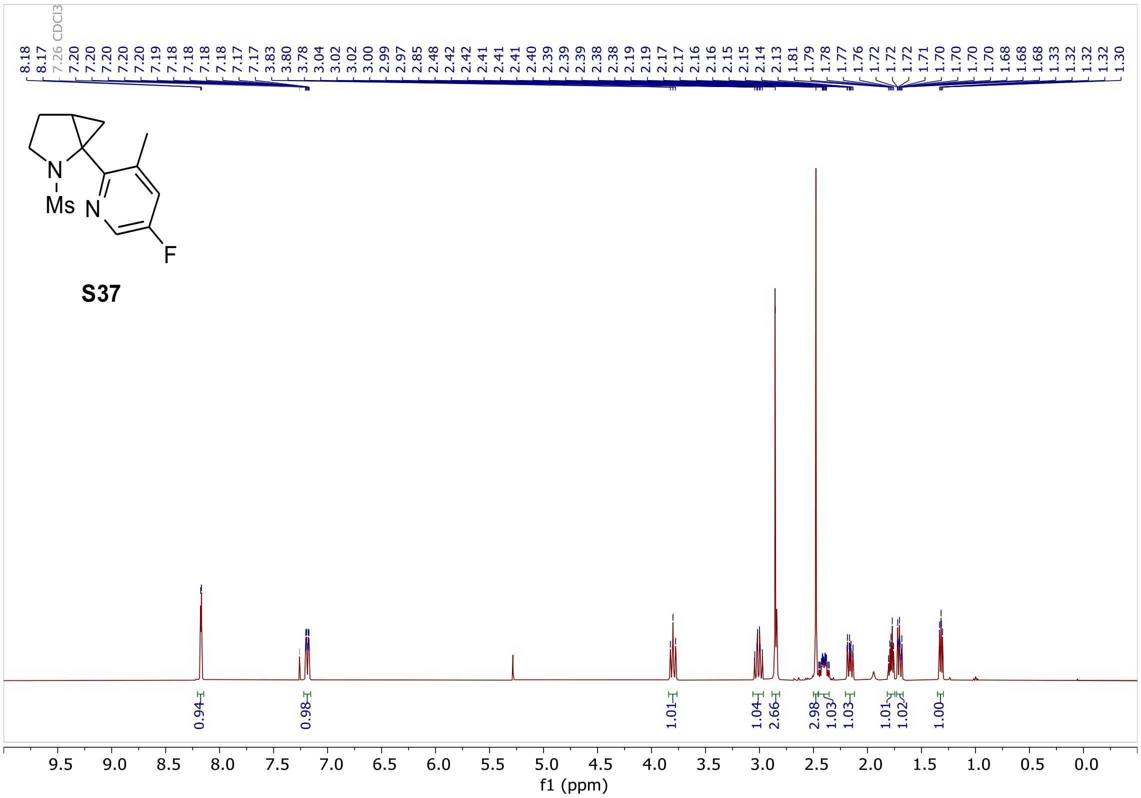

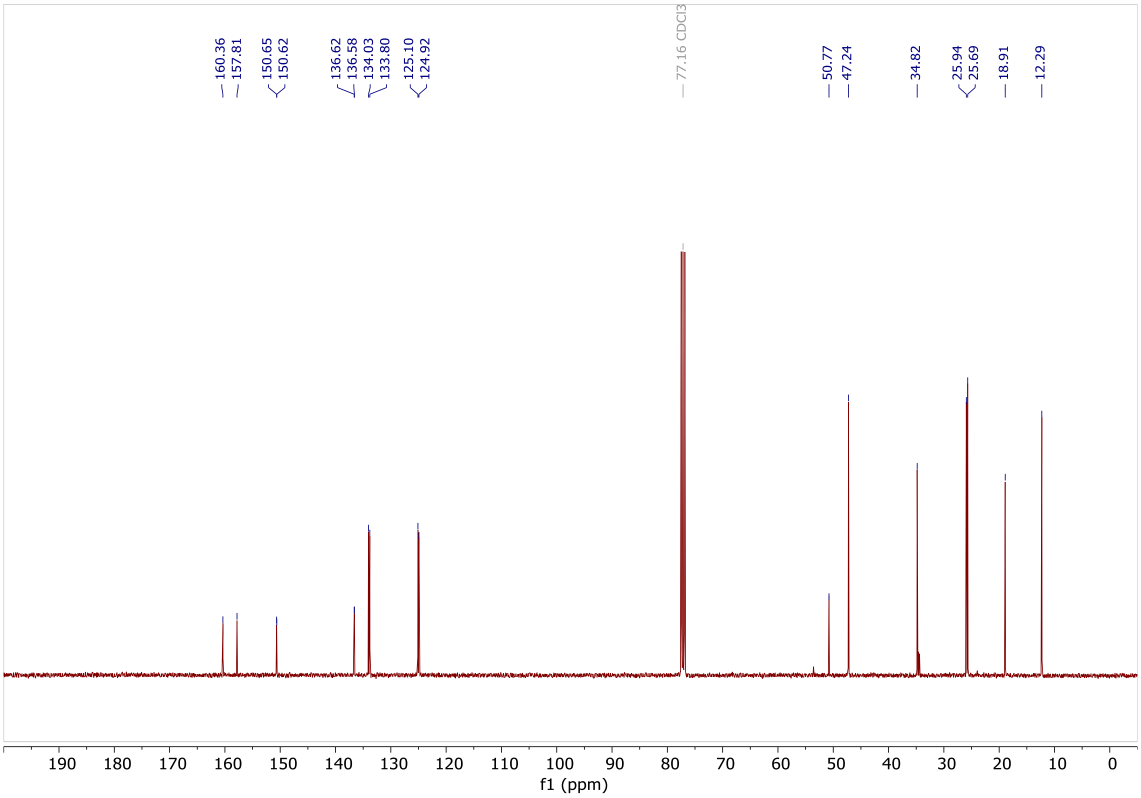

400 MHz ^{1}H NMR spectrum; 100.6 MHz ^{13}C NMR spectrum; $CDCl_3$ of **S38**

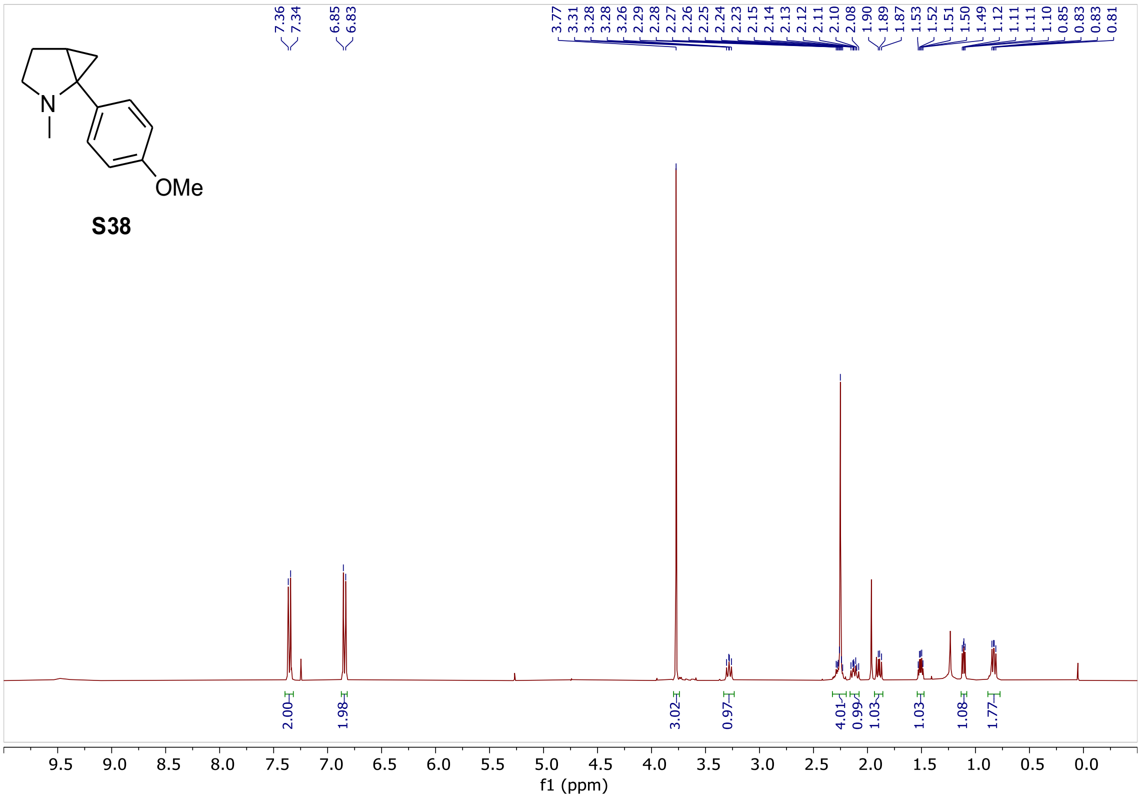

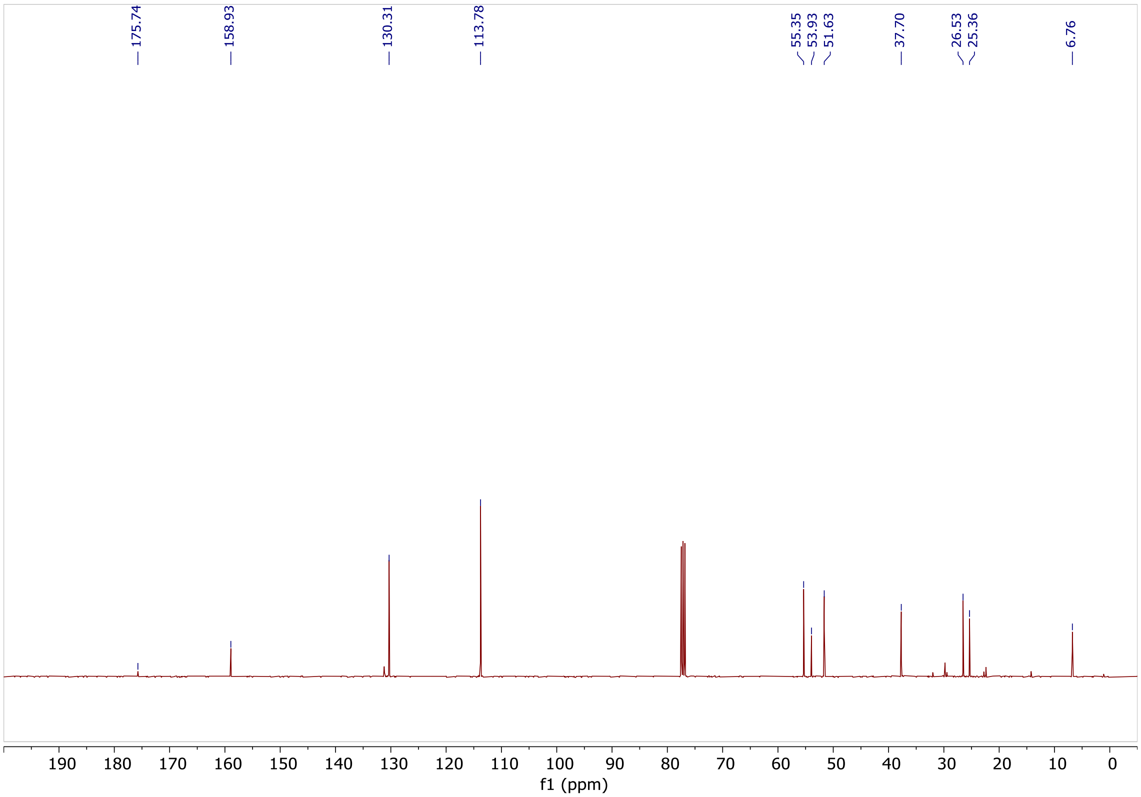

400 MHz ^{1}H NMR spectrum; 100.6 MHz ^{13}C NMR spectrum; $CDCl_3$ of **S39**

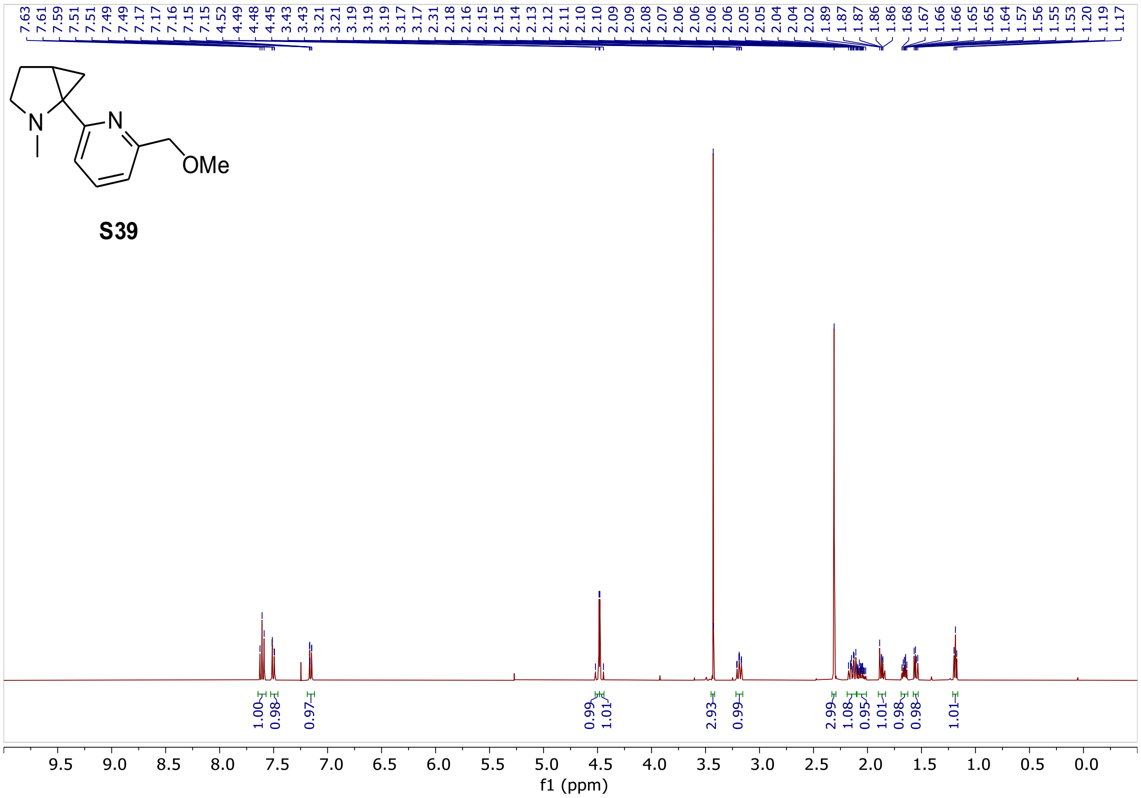

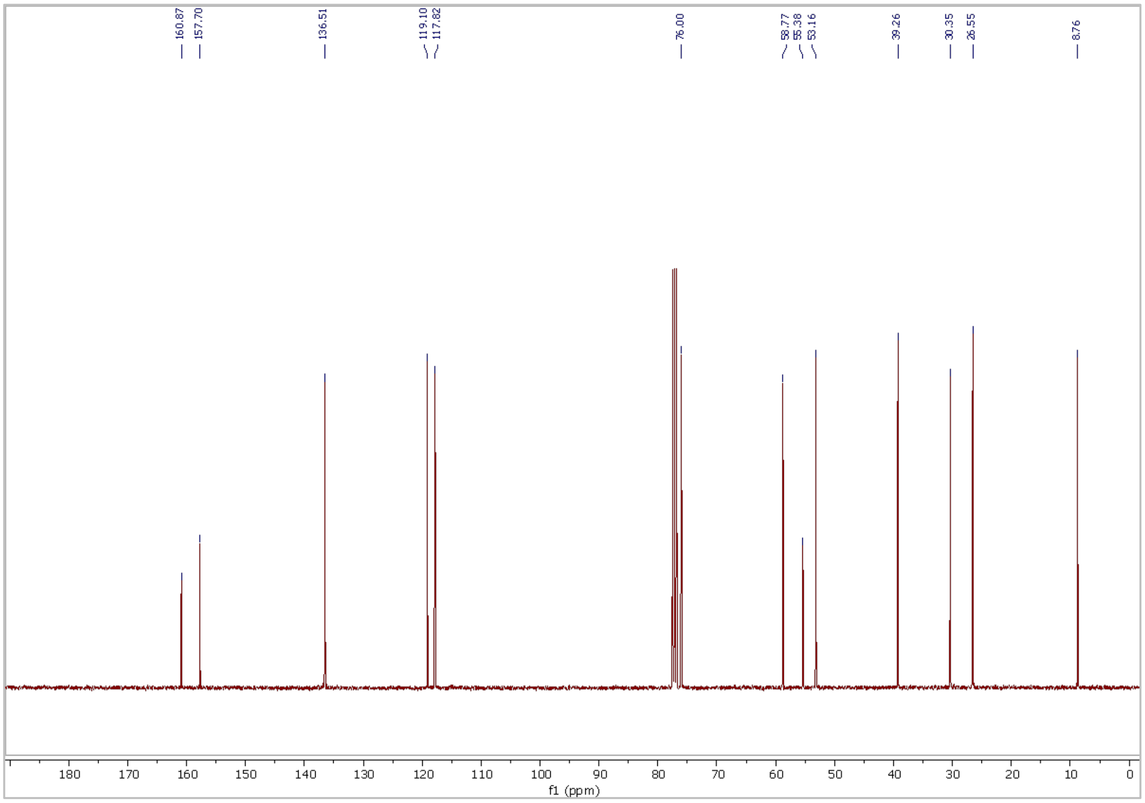

400 MHz ^{1}H NMR spectrum; 100.6 MHz ^{13}C NMR spectrum; $CDCl_3$ of **S40**

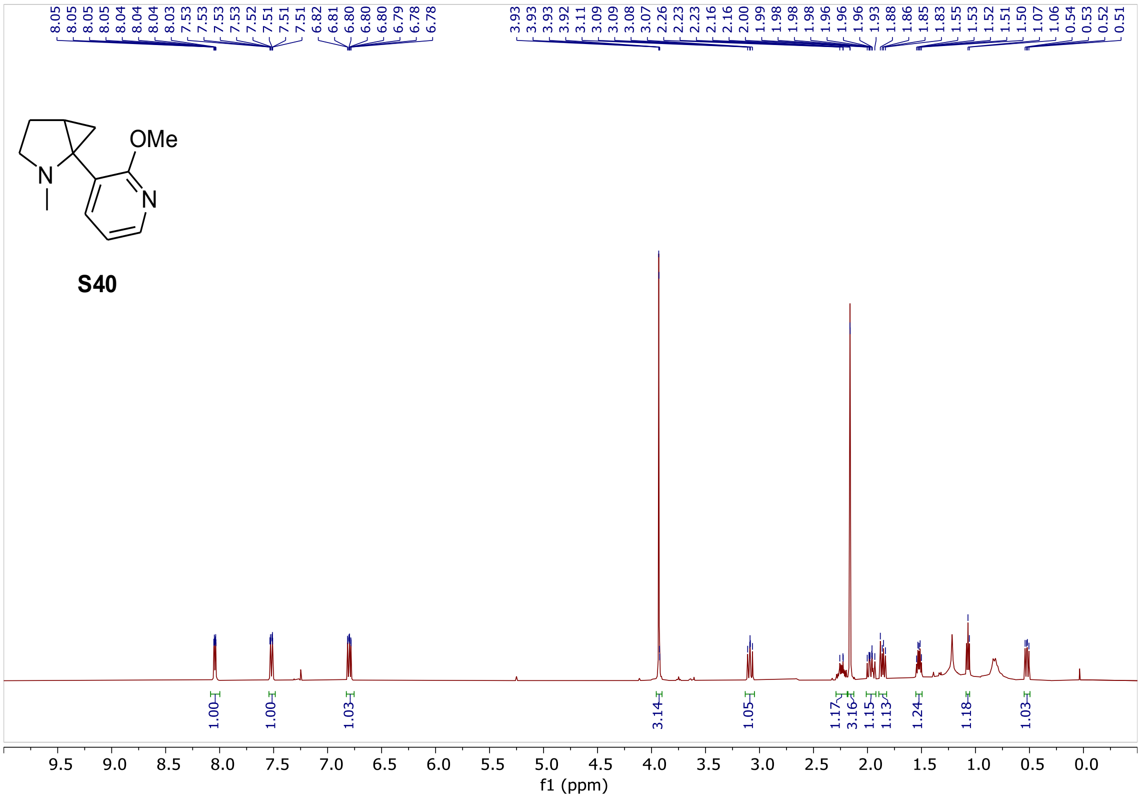

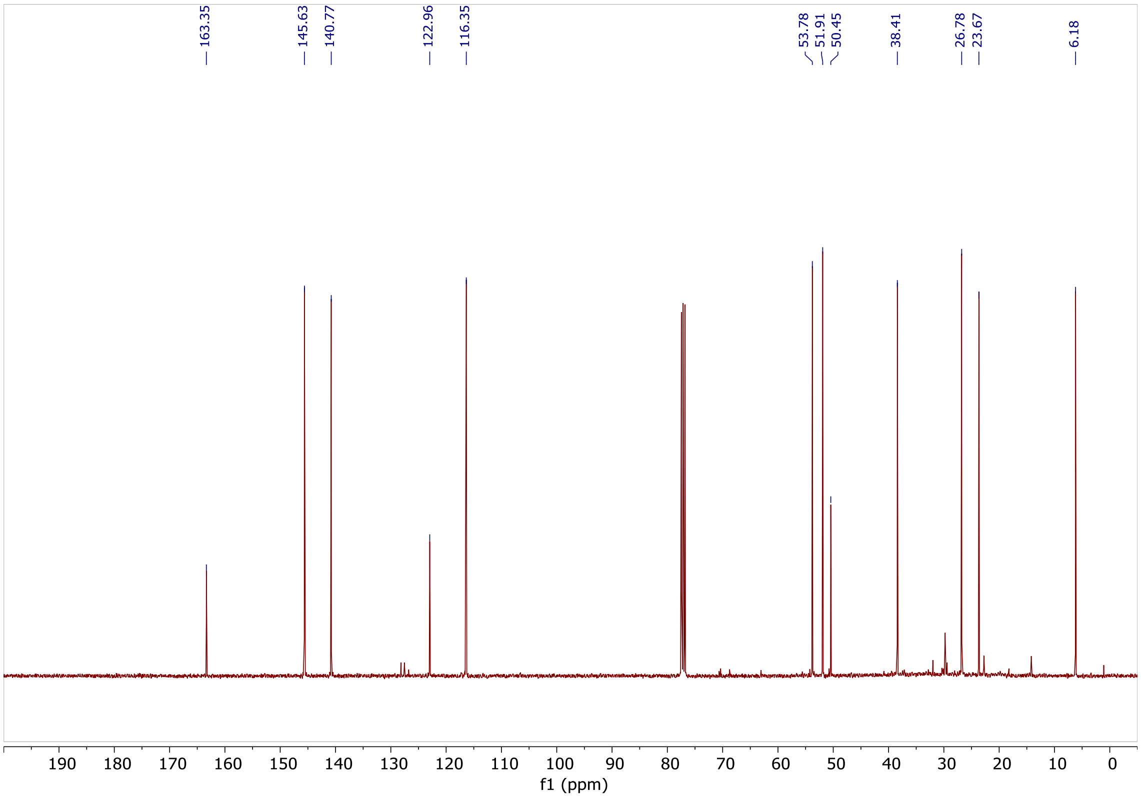

400 MHz ^{1}H NMR spectrum; 100.6 MHz ^{13}C NMR spectrum; $CDCl_3$ of **S41**

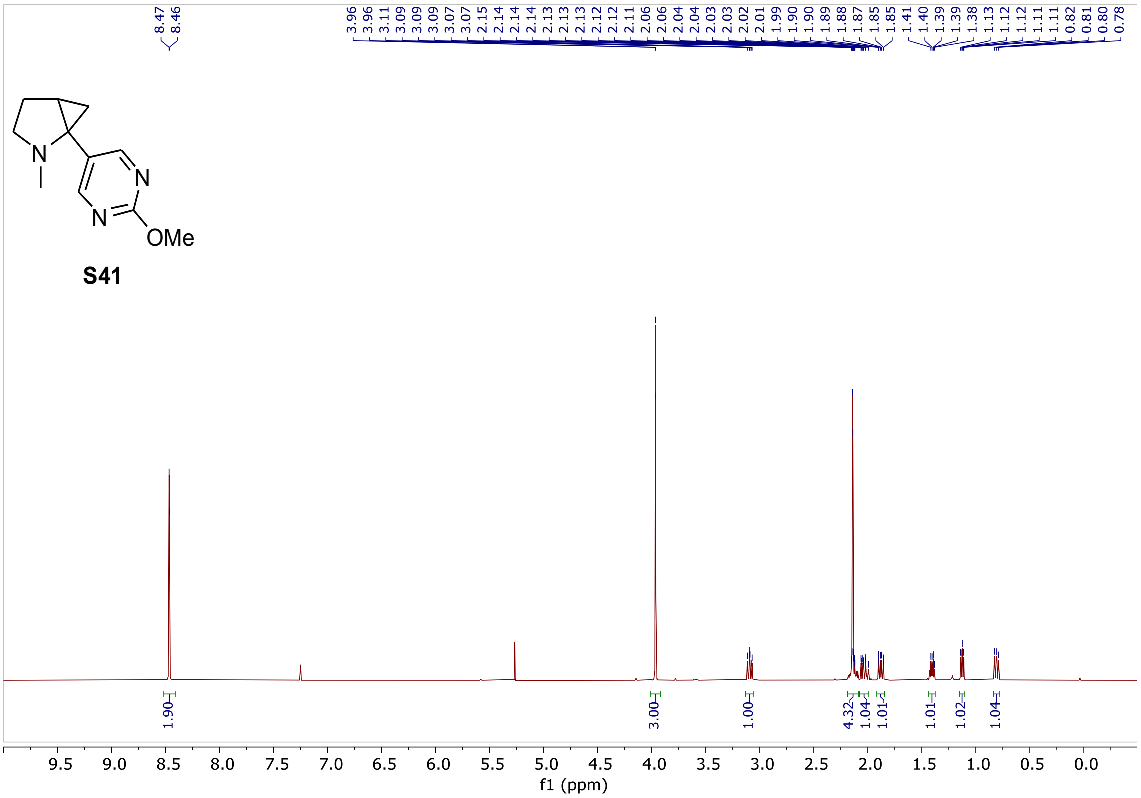

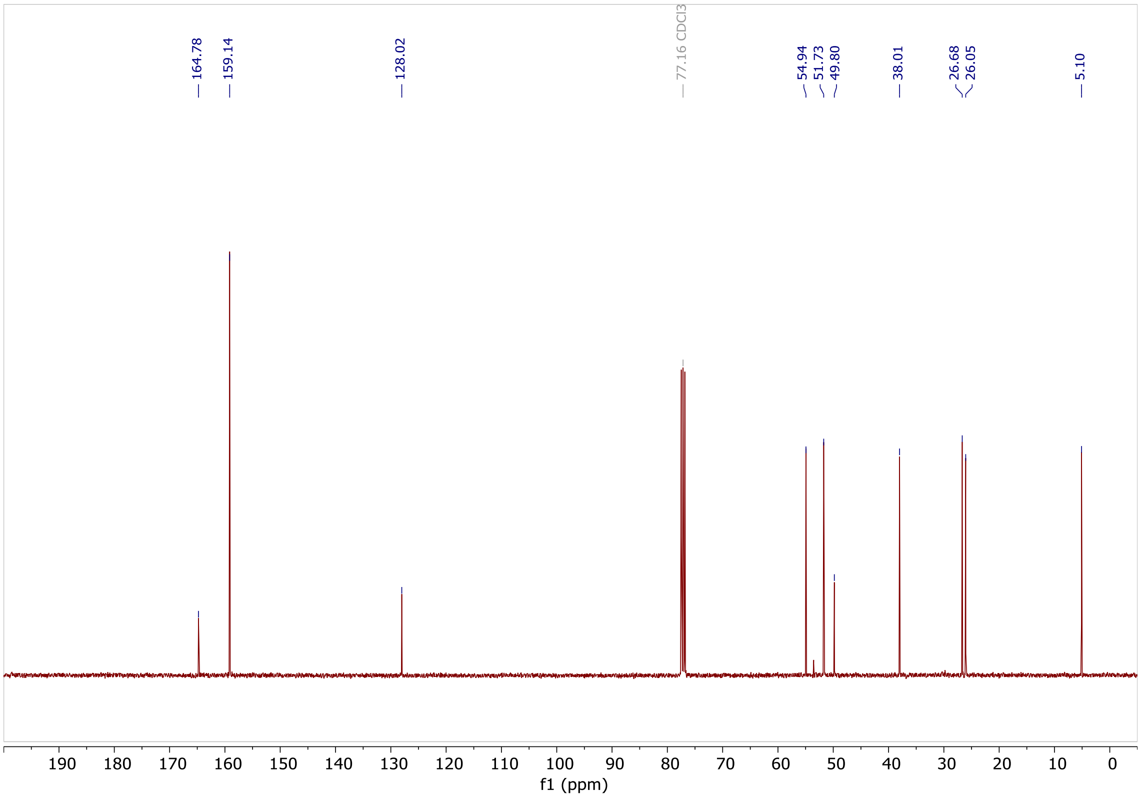

400 MHz ^1H NMR spectrum; 100.6 MHz ^{13}C NMR spectrum; $CDCl_3$ of **S42**

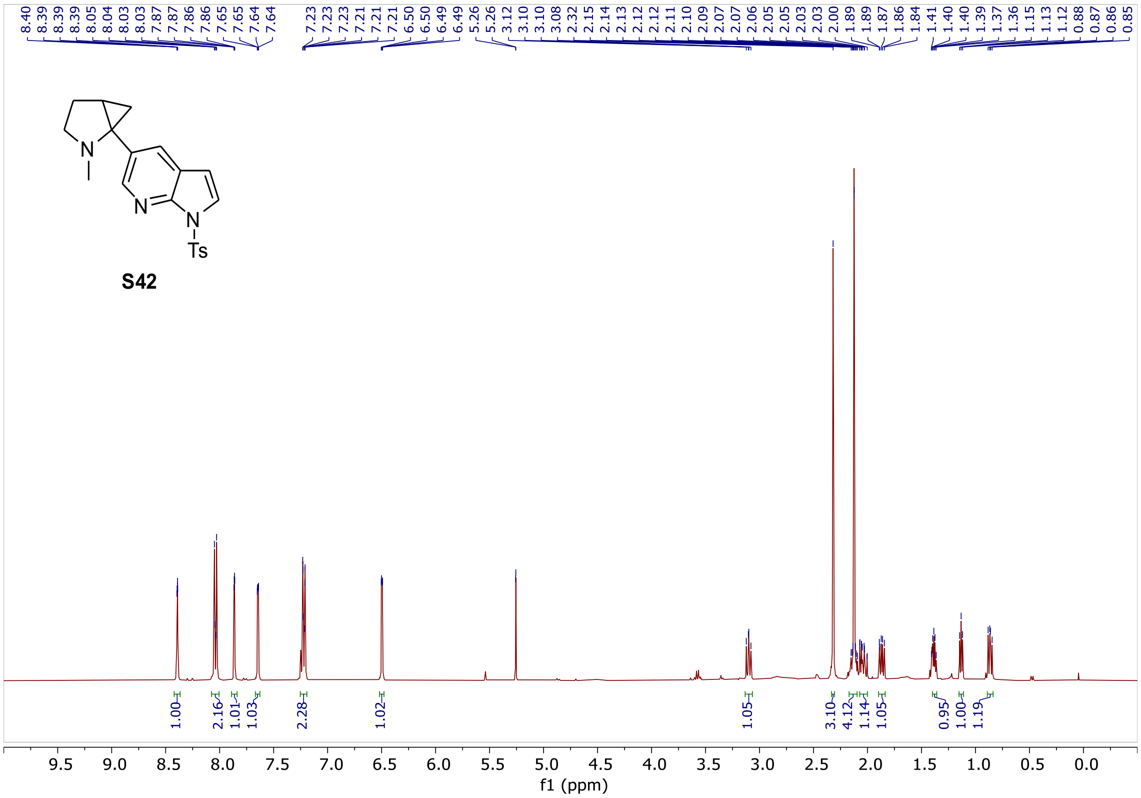

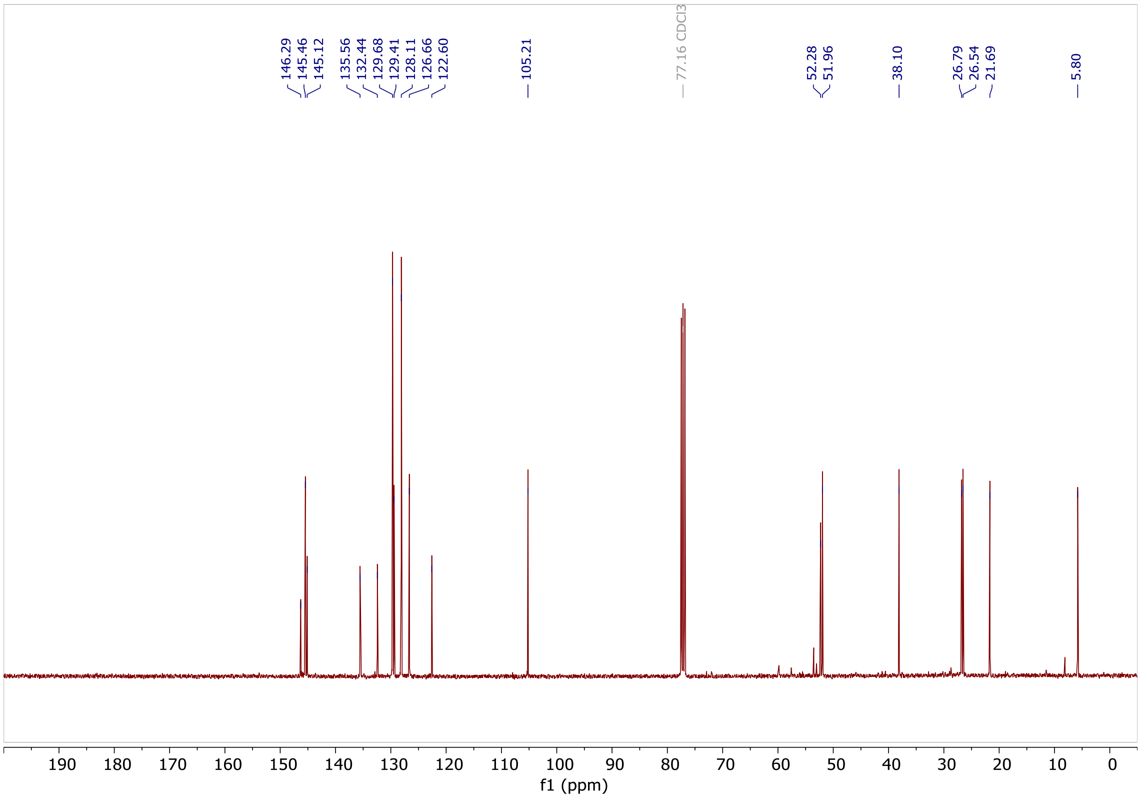

400 MHz ^{1}H NMR spectrum; 100.6 MHz ^{13}C NMR spectrum; $CDCl_3$ of **S43**

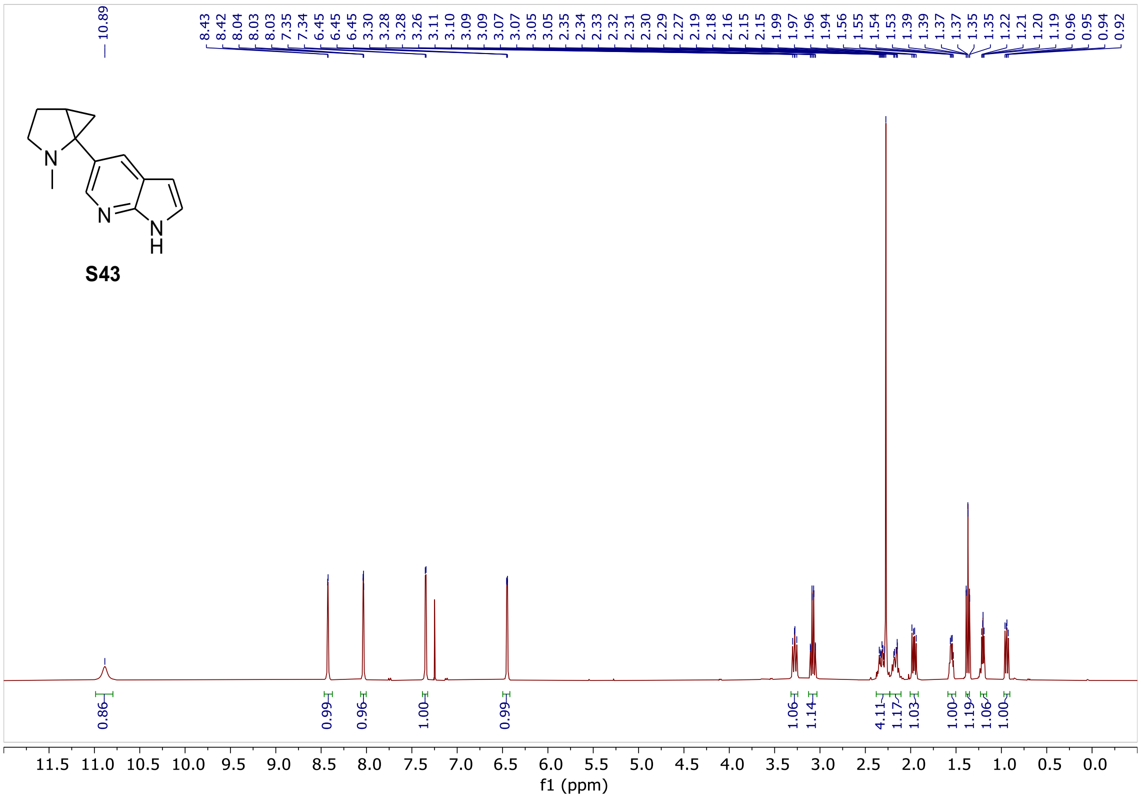

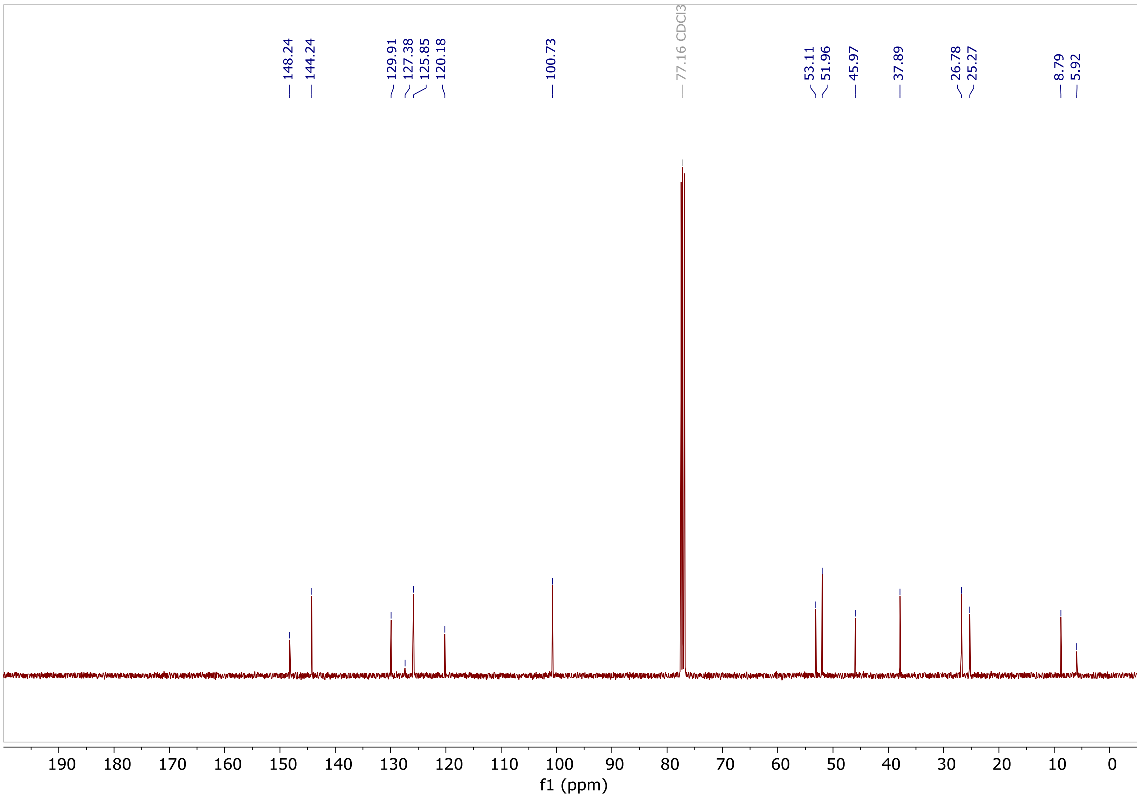

400 MHz ^{1}H NMR spectrum; 100.6 MHz ^{13}C NMR spectrum; $CDCl_3$ of **S44**

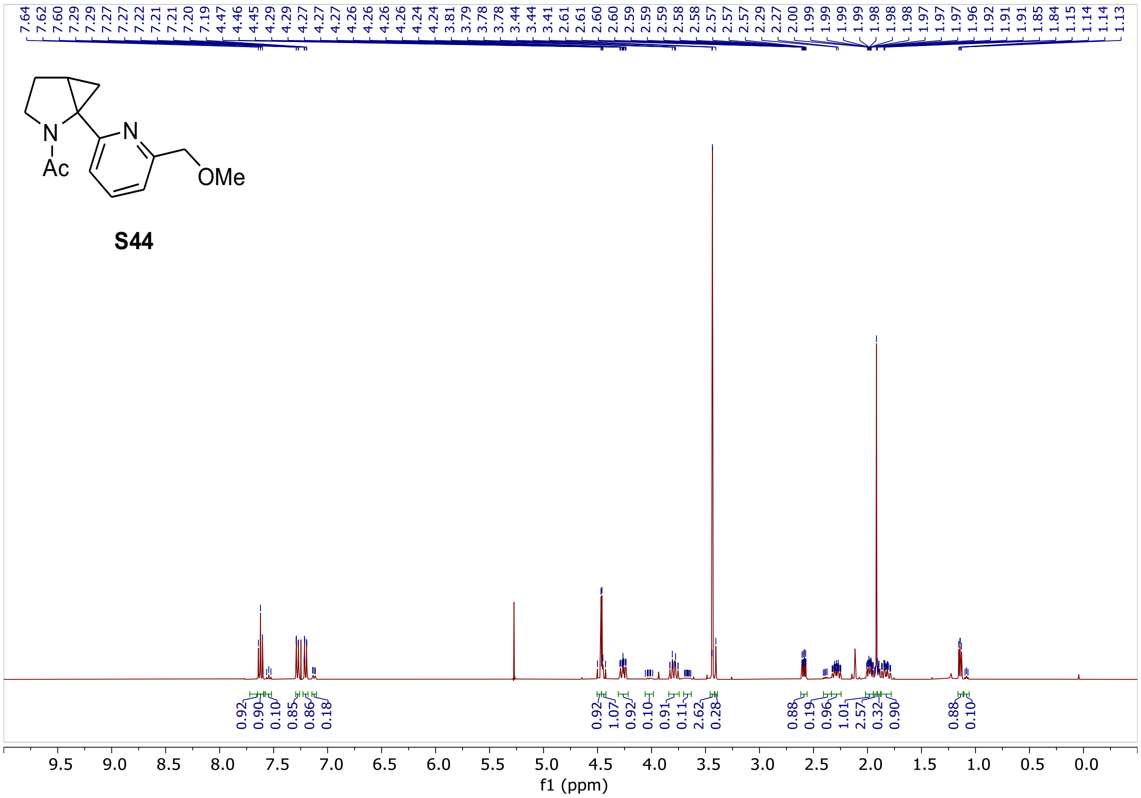

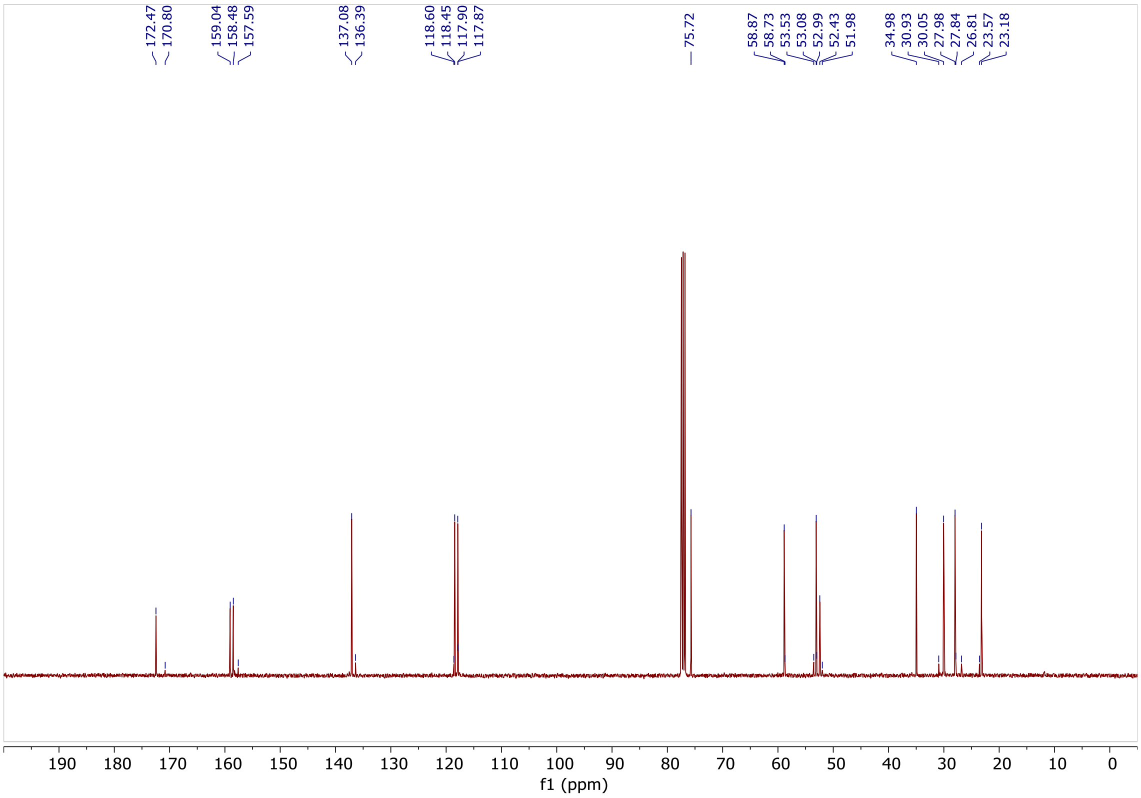

400 MHz ^{1}H NMR spectrum; 100.6 MHz ^{13}C NMR spectrum; $CDCl_3$ of **S45**

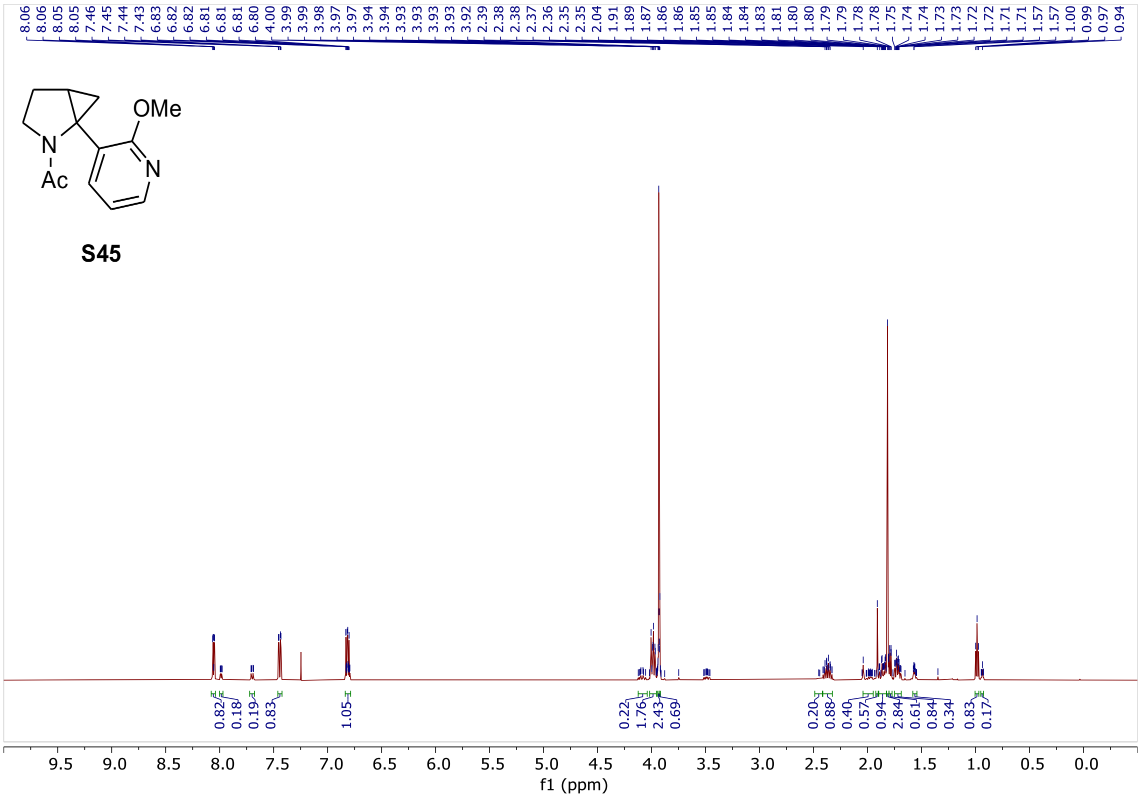

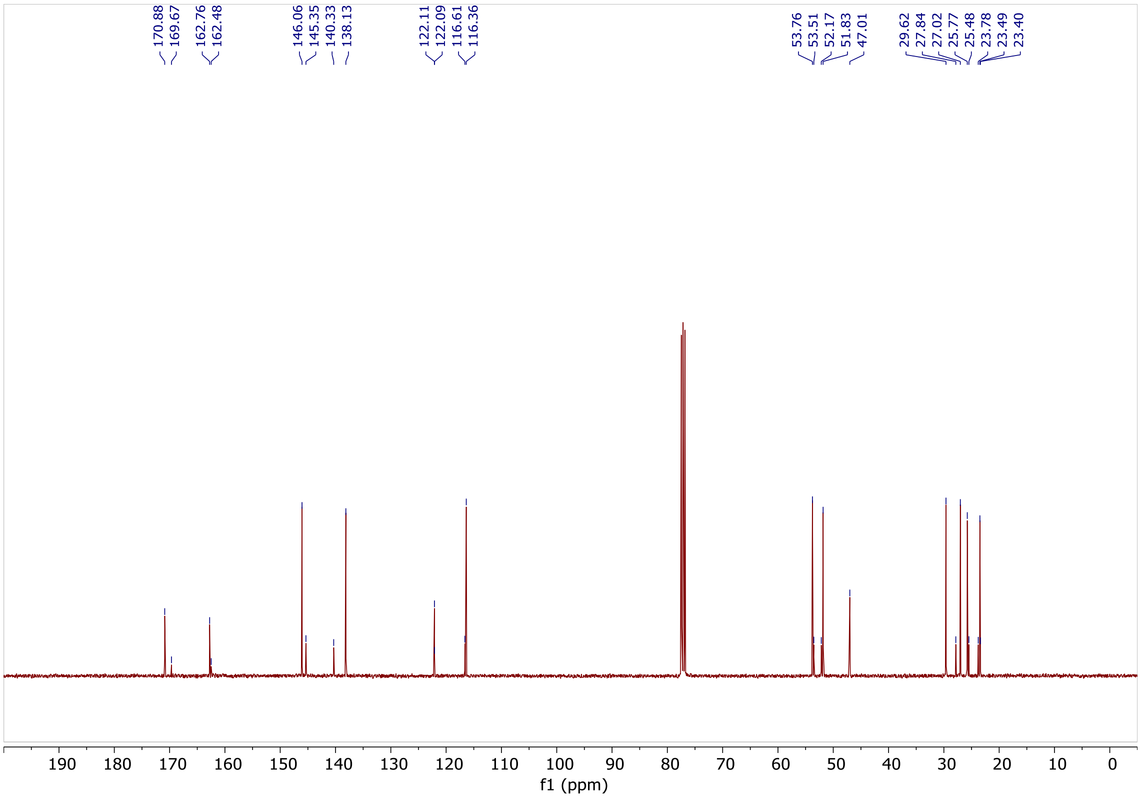

400 MHz ^{1}H NMR spectrum; 100.6 MHz ^{13}C NMR spectrum; $CDCl_3$ of **S46**

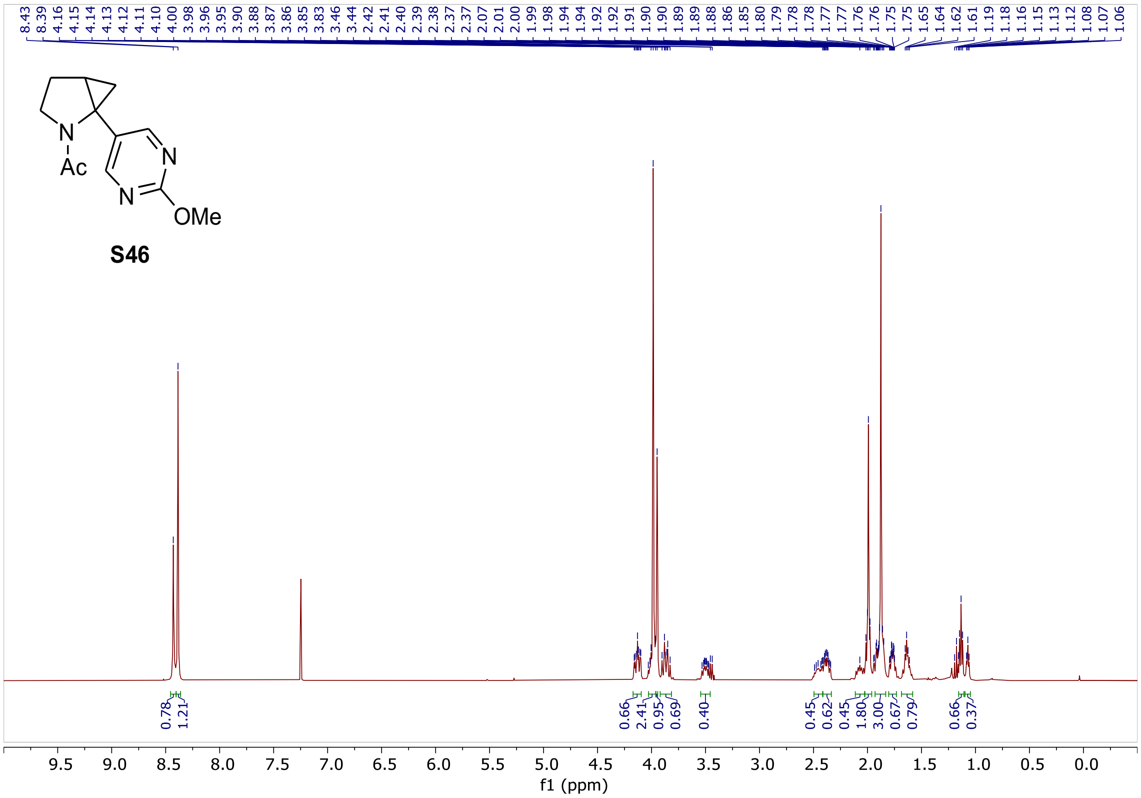

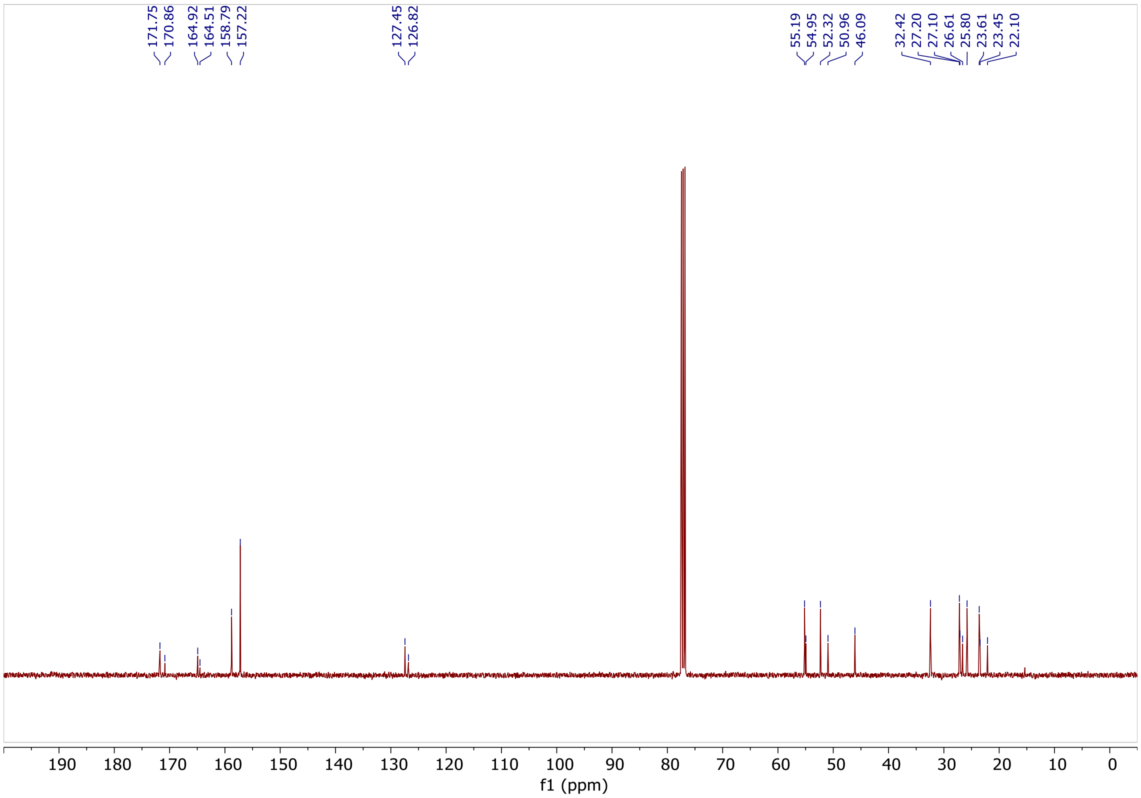

400 MHz ^{1}H NMR spectrum; 100.6 MHz ^{13}C NMR spectrum; $CDCl_3$ of **S47**

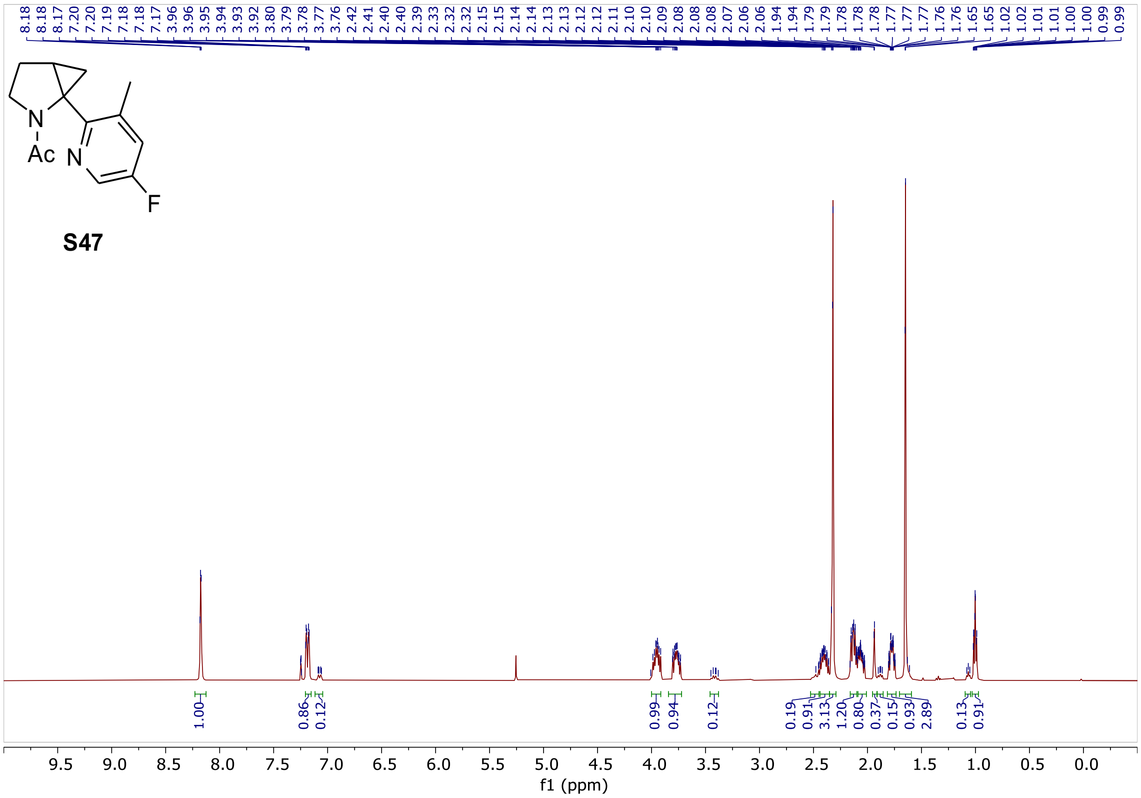

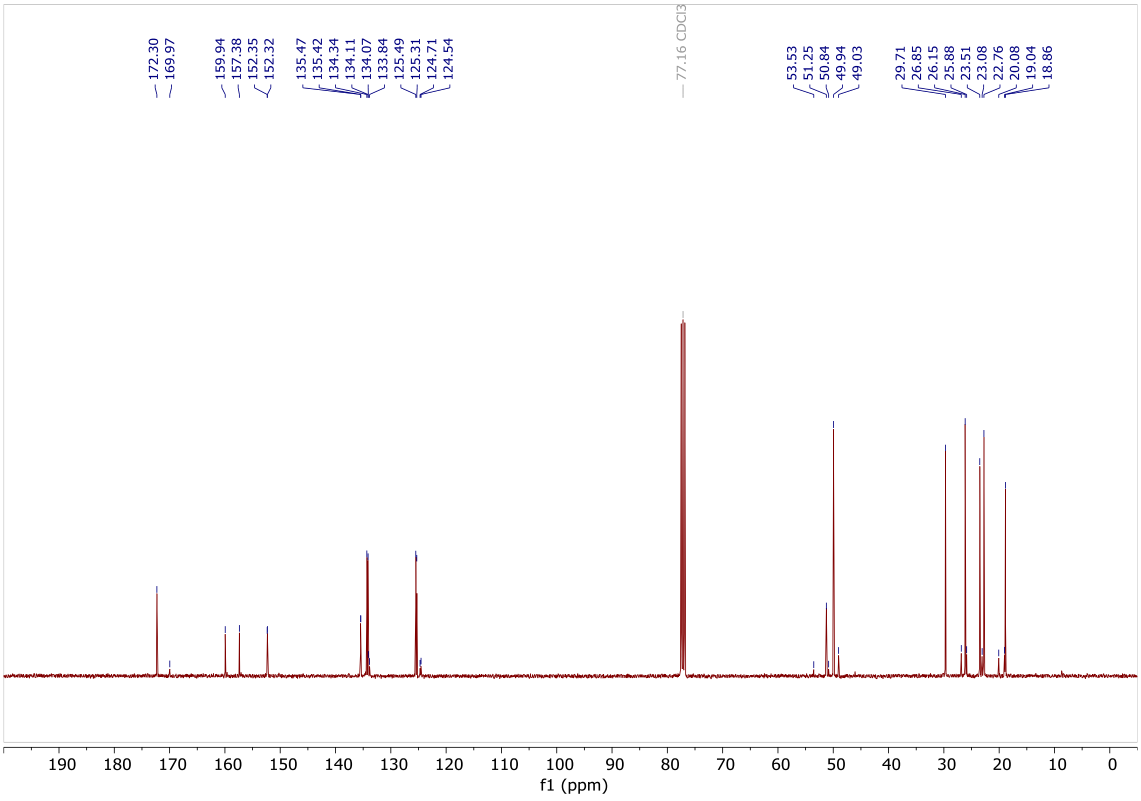

400 MHz ^{1}H NMR spectrum; 100.6 MHz ^{13}C NMR spectrum; $CDCl_3$ of **S48**

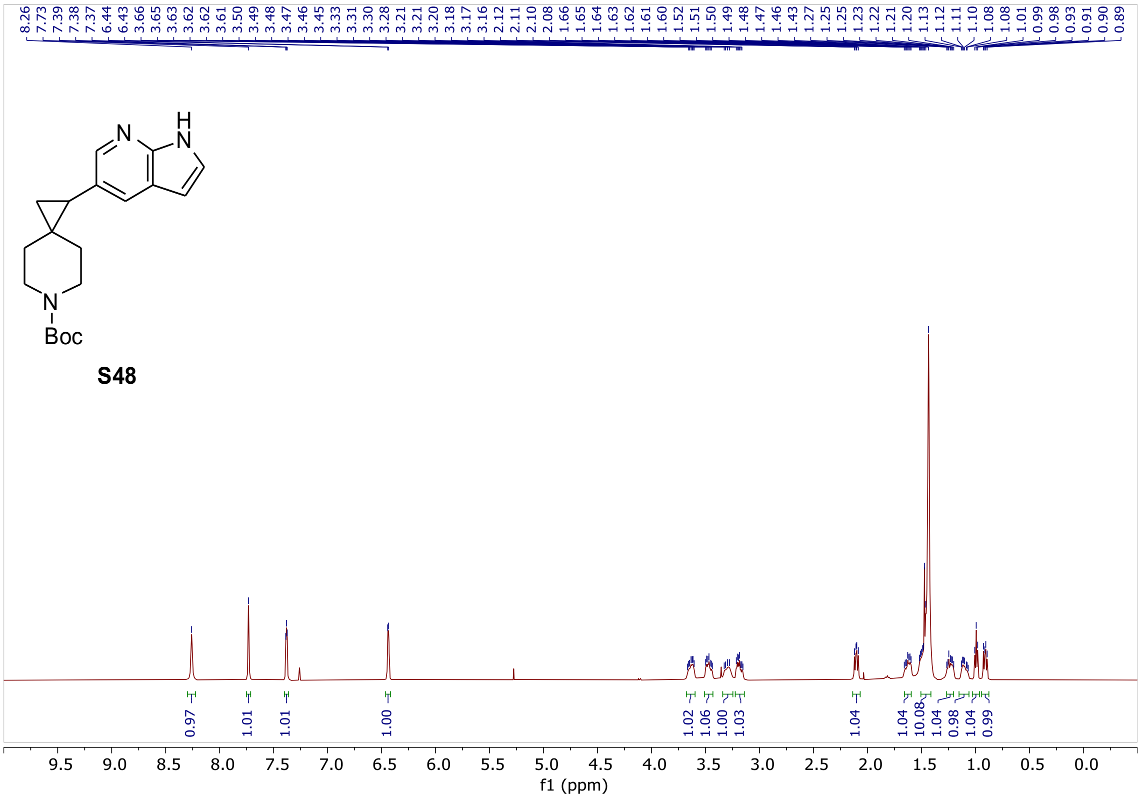

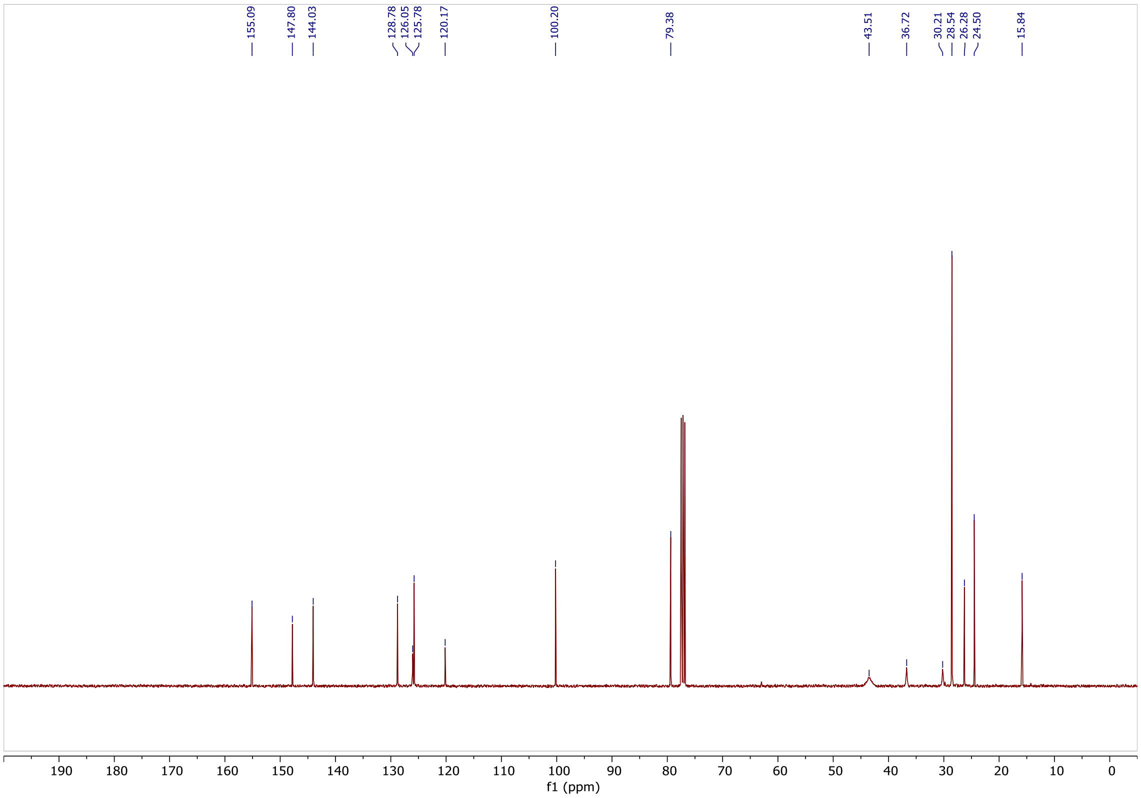

400 MHz ^1H NMR spectrum; 100.6 MHz ^{13}C NMR spectrum; $CDCl_3$ of **S49**

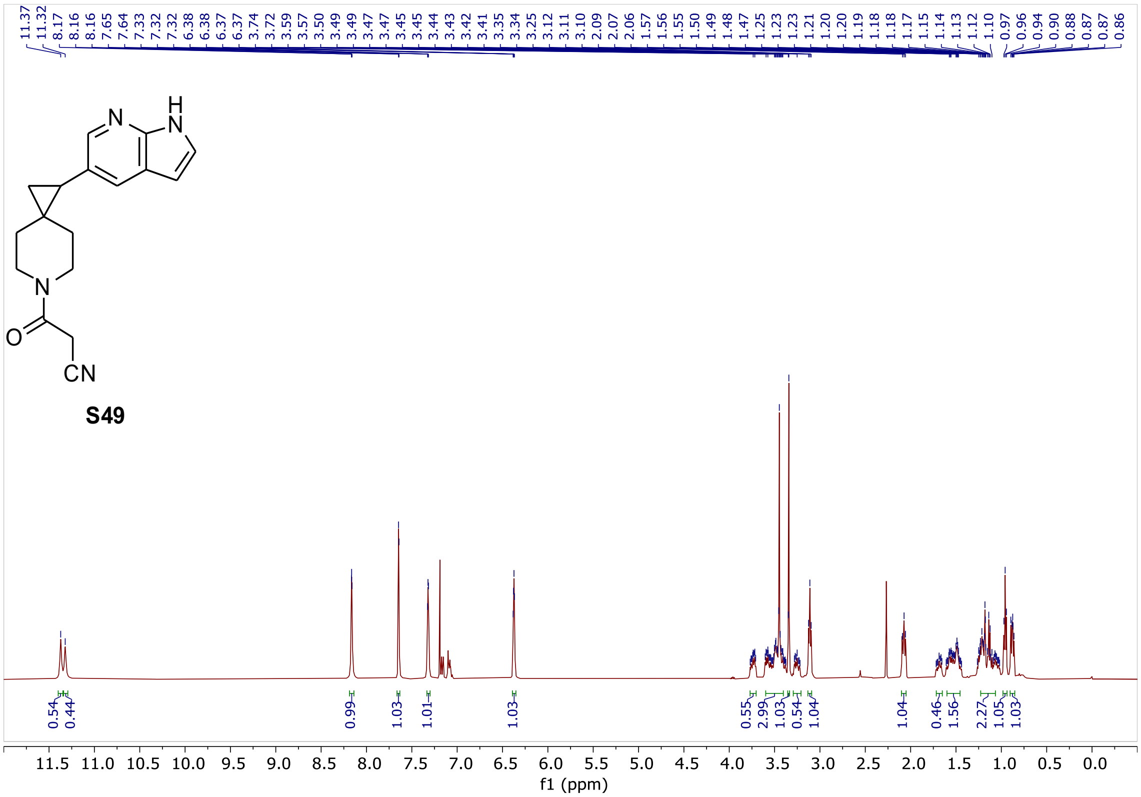

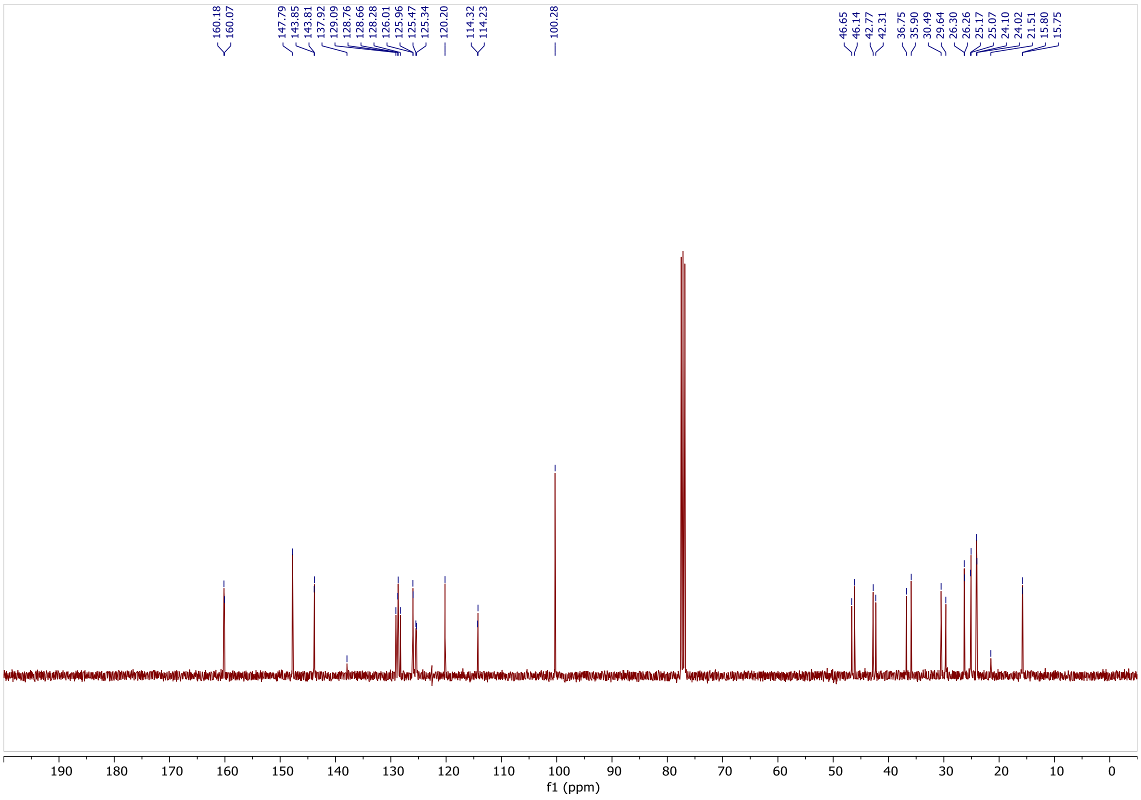

400 MHz ^{1}H NMR spectrum; 100.6 MHz ^{13}C NMR spectrum; $CDCl_3$ of **S50**

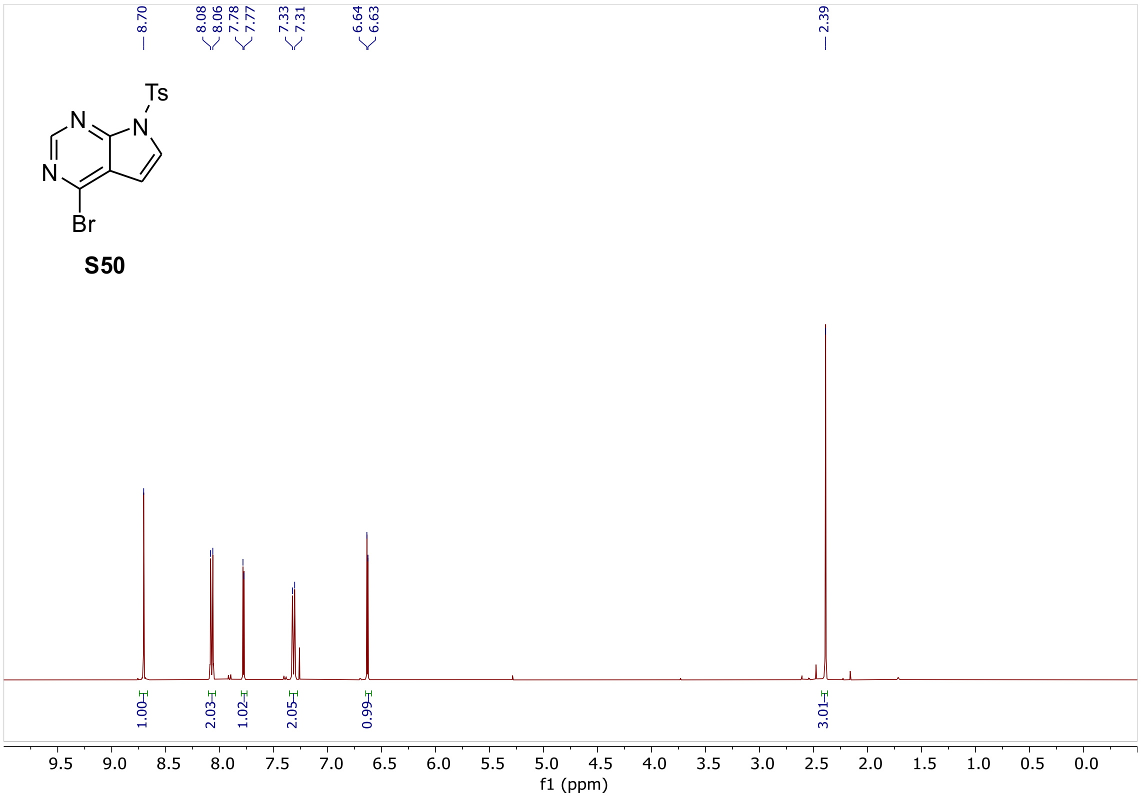

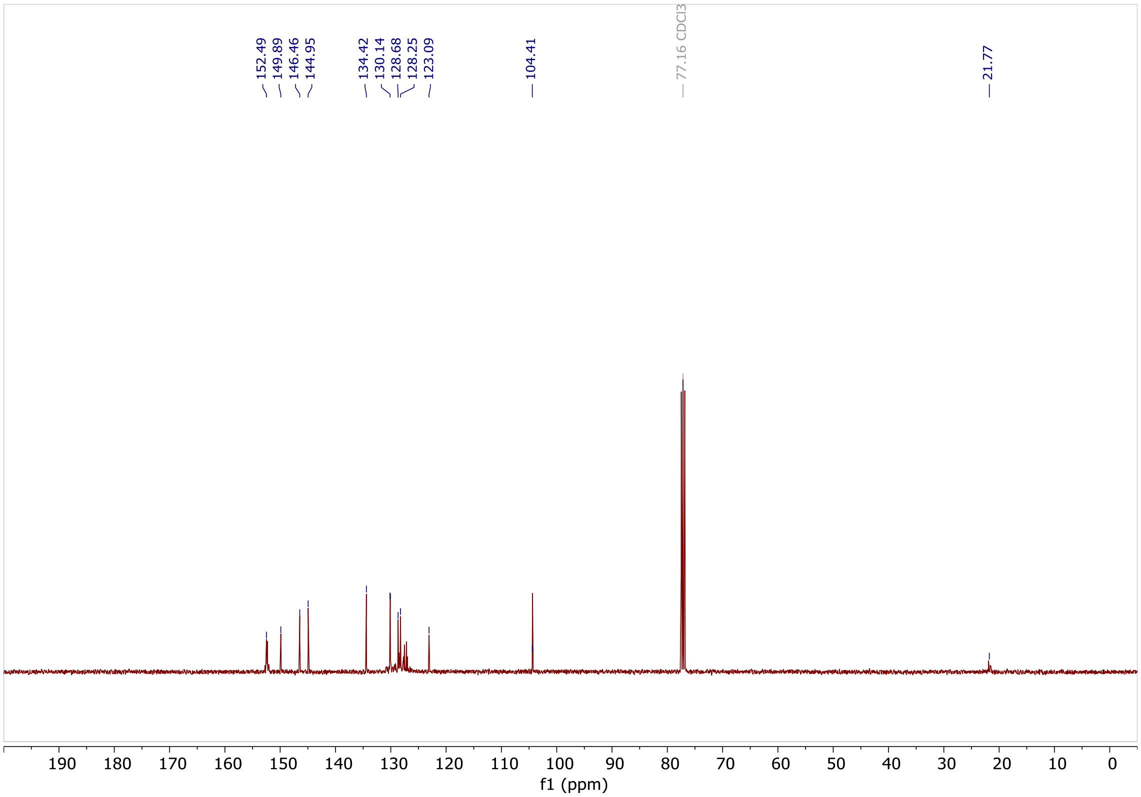

400 MHz ^{1}H NMR spectrum; 100.6 MHz ^{13}C NMR spectrum; $CDCl_3$ of **S51**

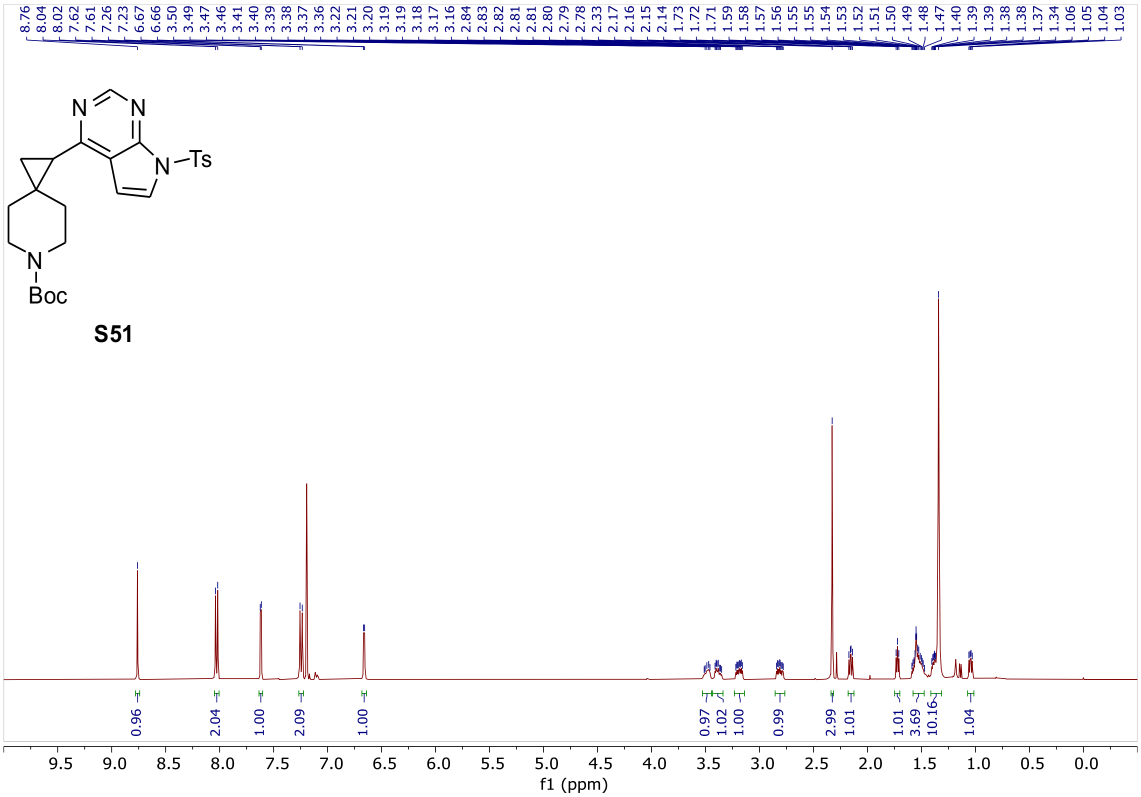

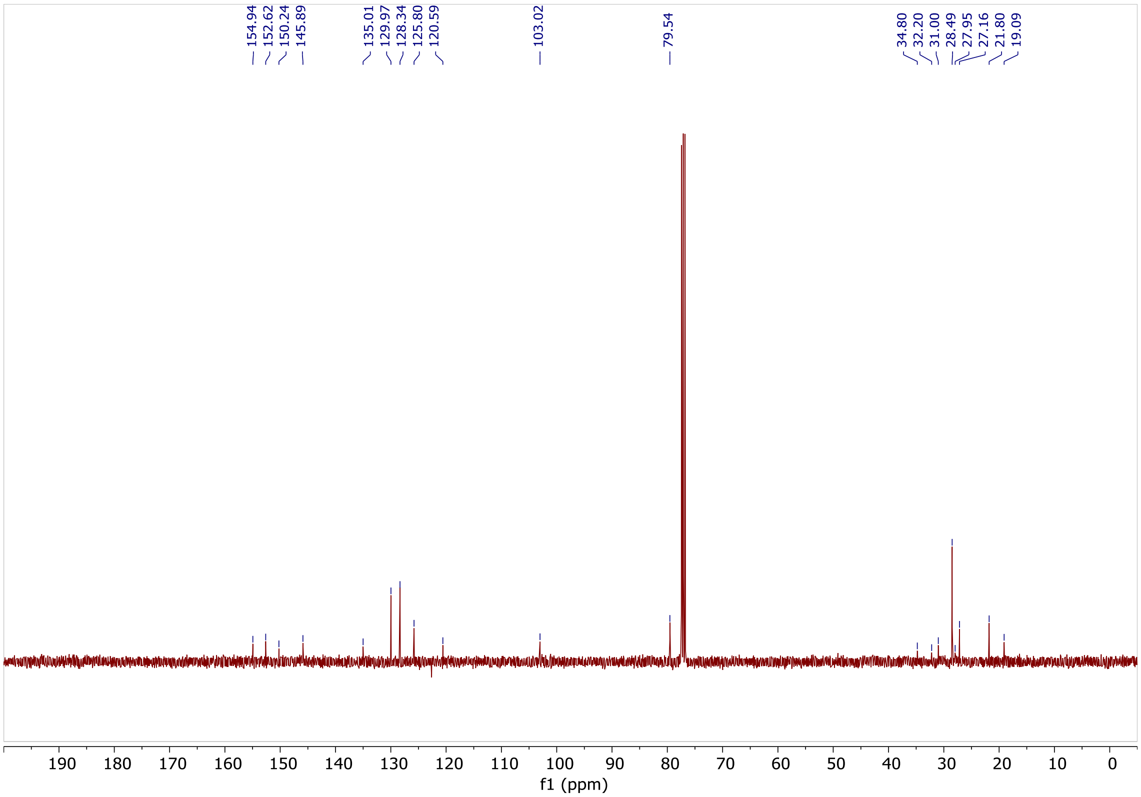

400 MHz ^{1}H NMR spectrum; 100.6 MHz ^{13}C NMR spectrum; $CDCl_3$ of **S52**

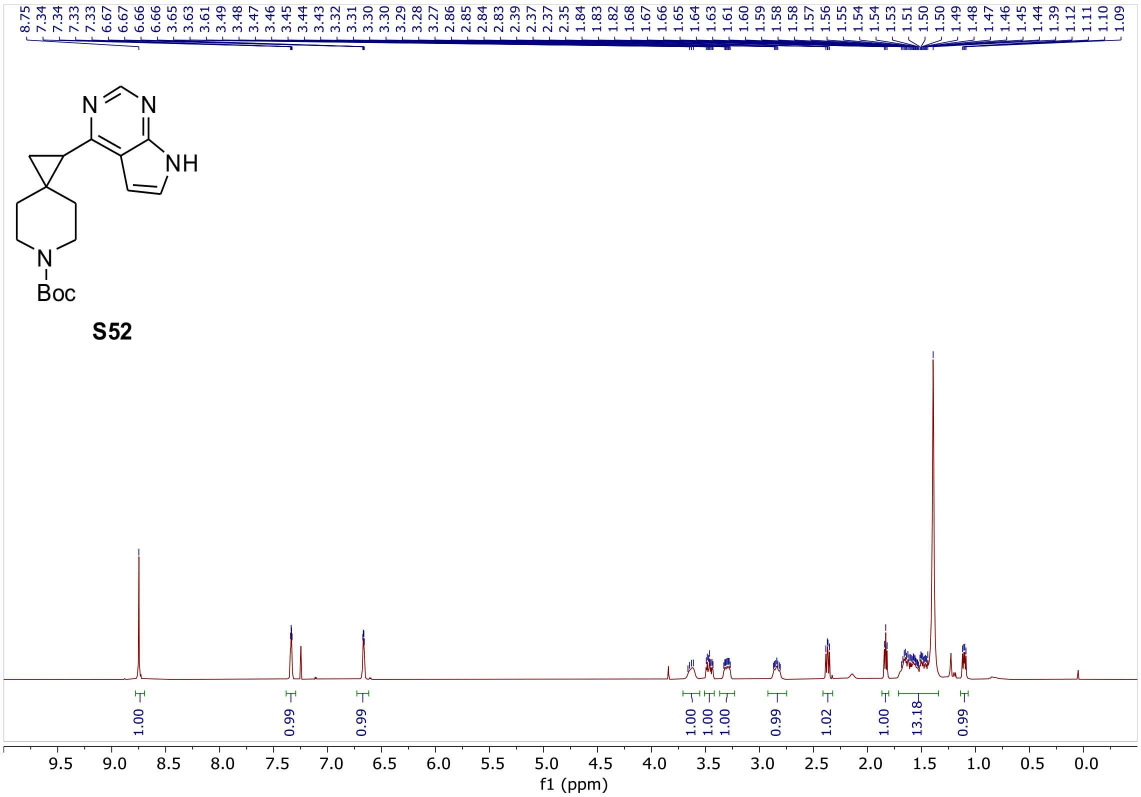

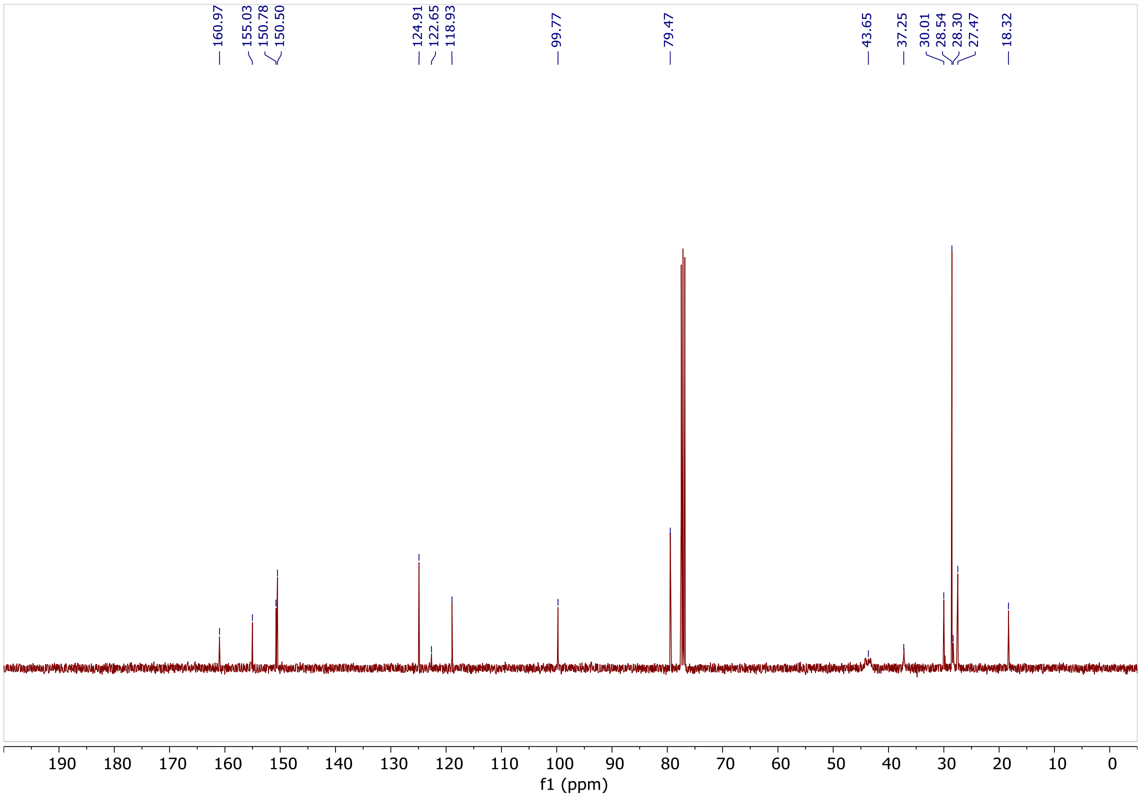

400 MHz ^{1}H NMR spectrum; 100.6 MHz ^{13}C NMR spectrum; DMSO-d_6 of **S53**

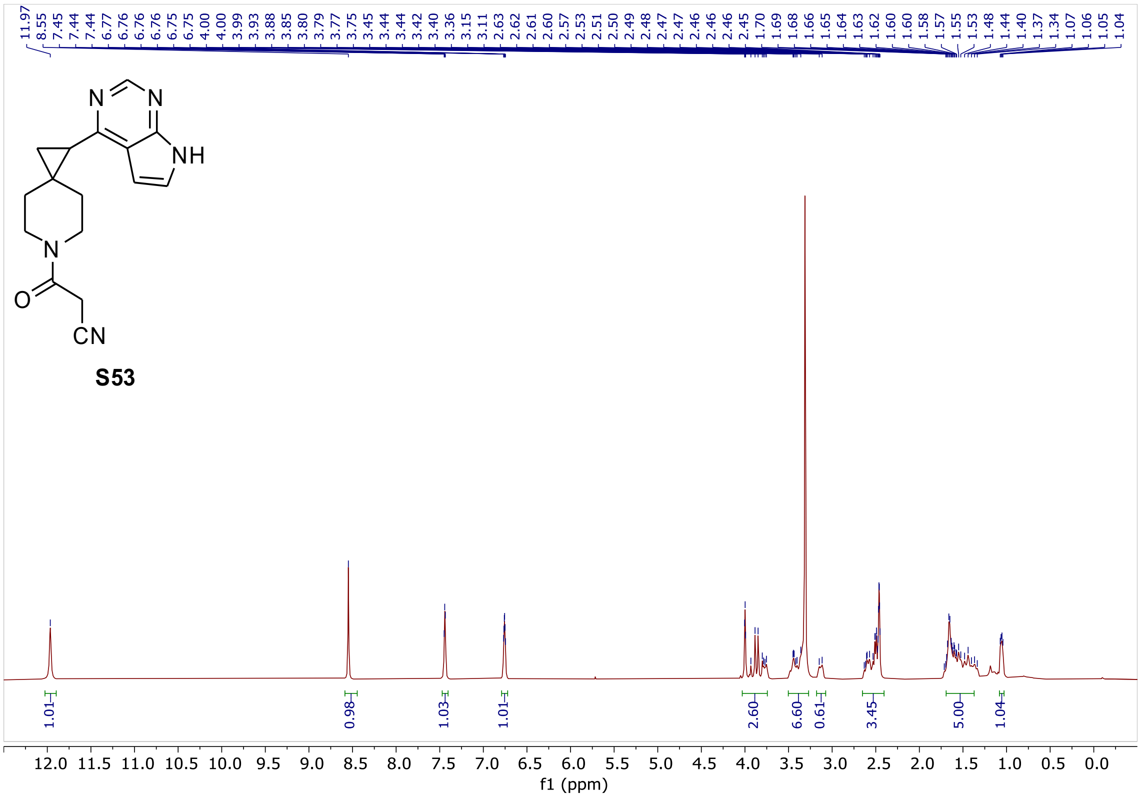

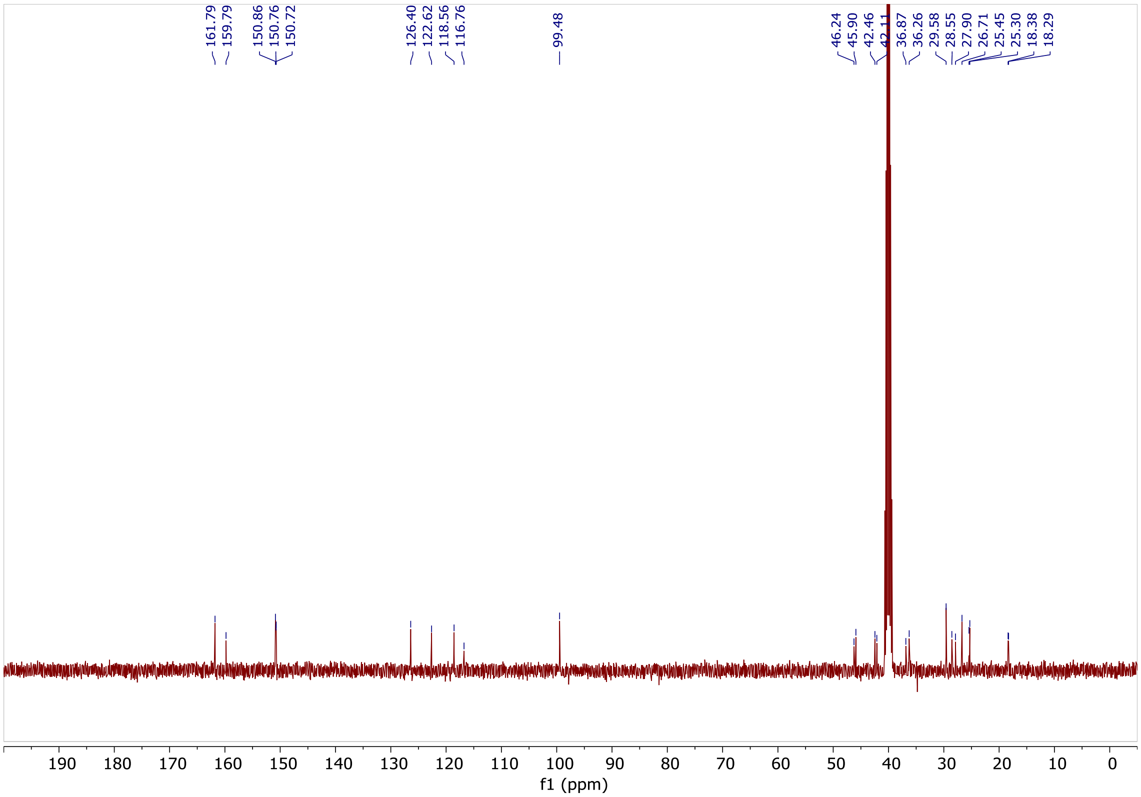

400 MHz ^{1}H NMR spectrum; 100.6 MHz ^{13}C NMR spectrum; $CDCl_3$ of **95**

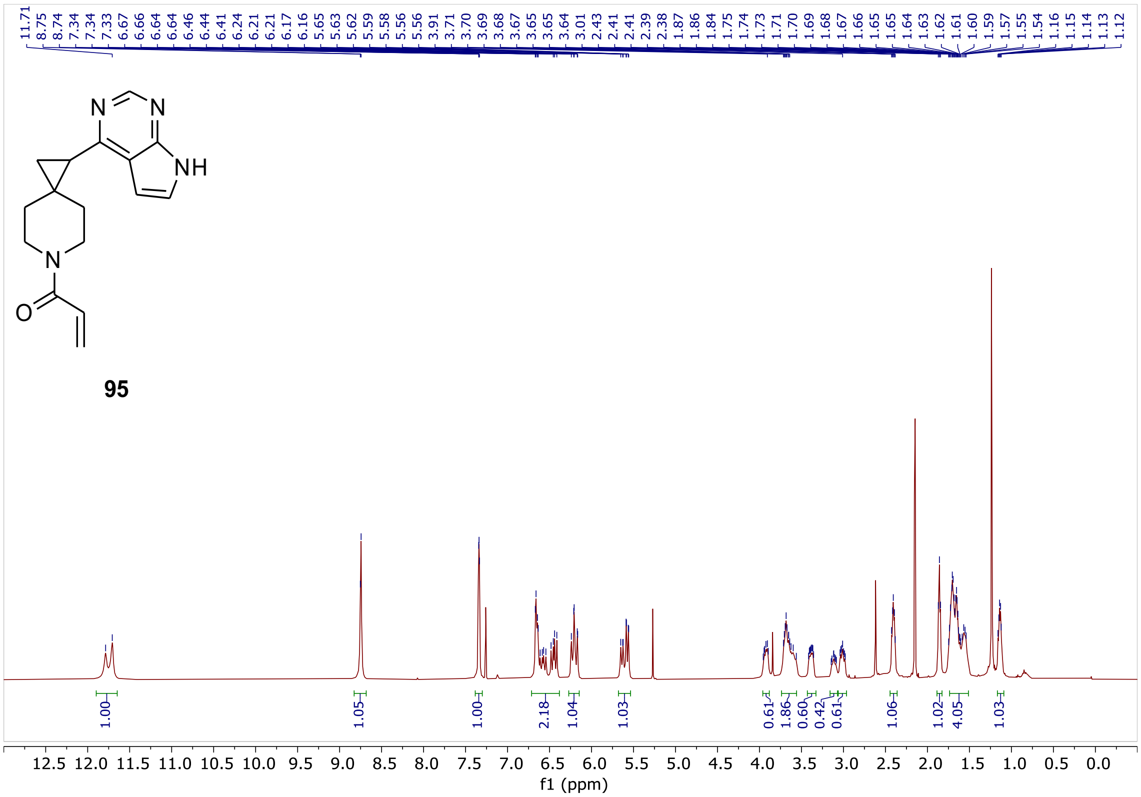

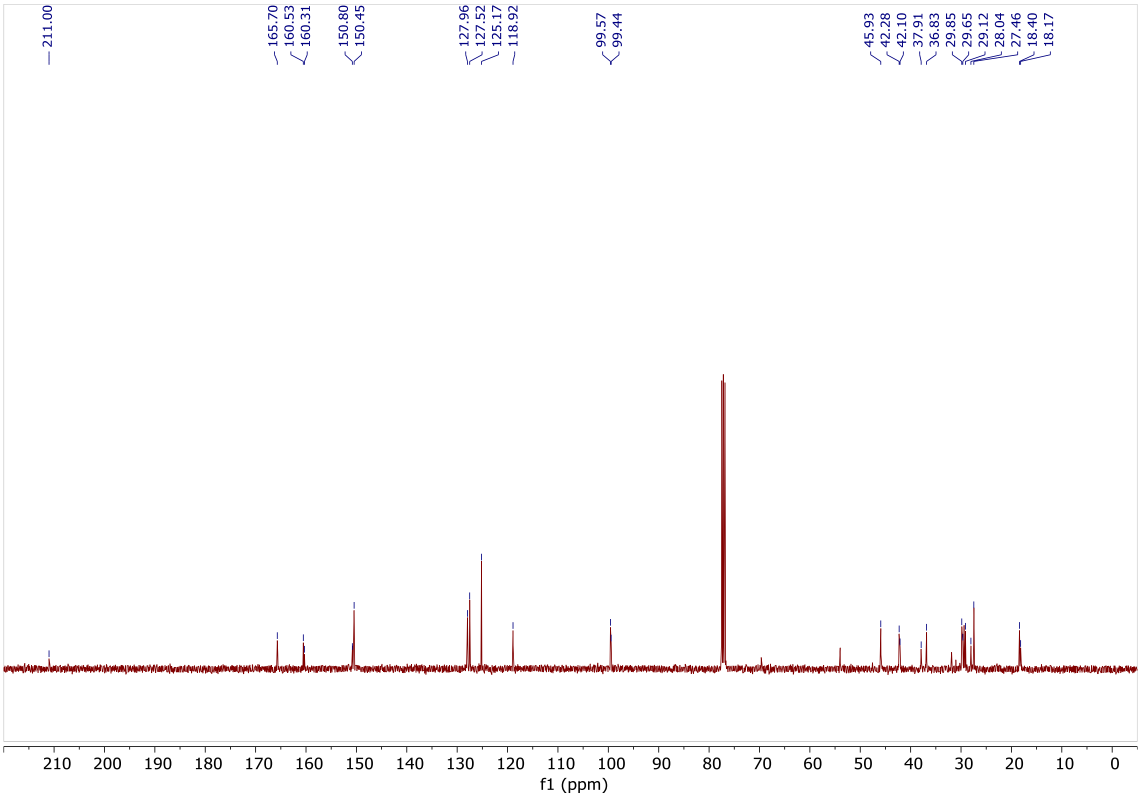

400 MHz ^{1}H NMR spectrum; 100.6 MHz ^{13}C NMR spectrum; $CDCl_3$ of **S54**

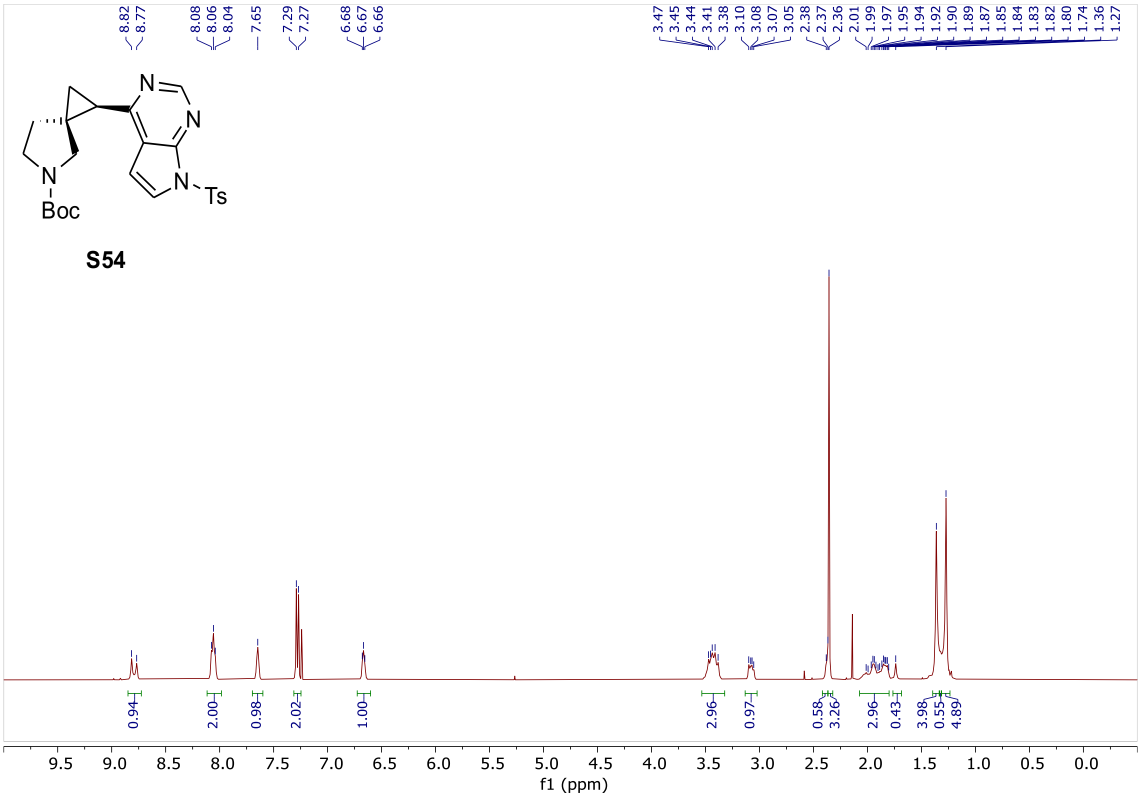

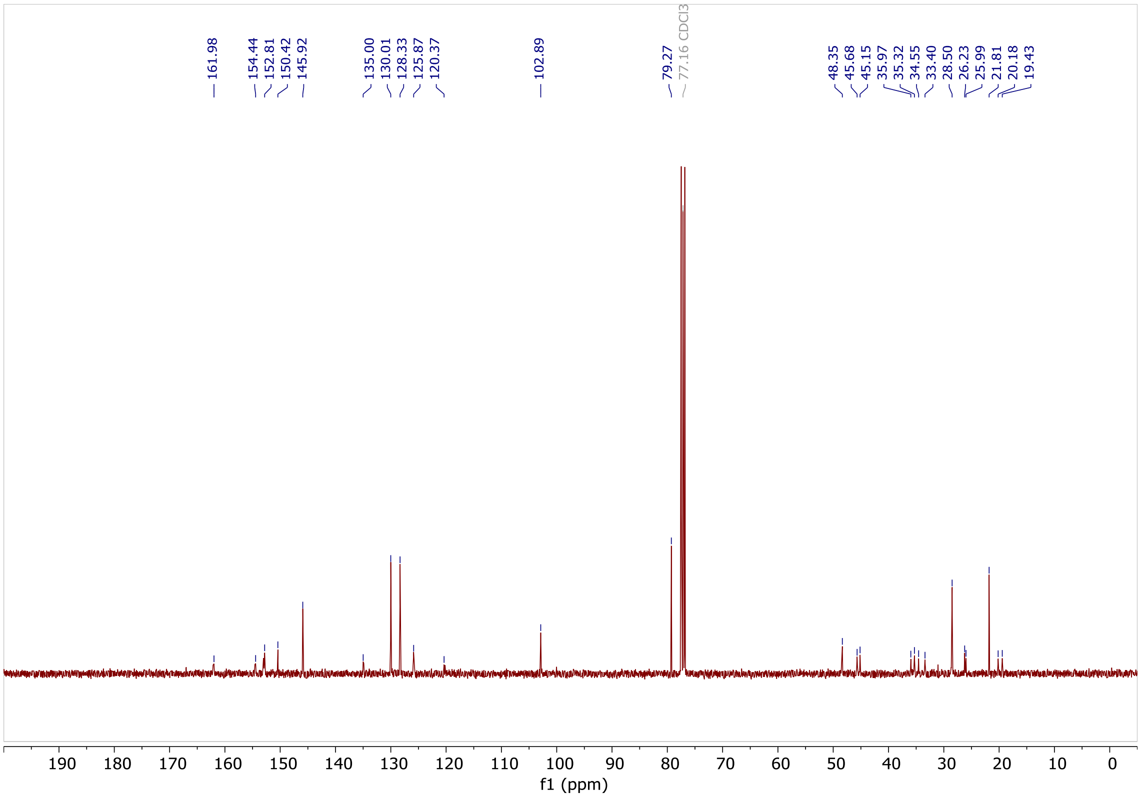

400 MHz ^{1}H NMR spectrum; 100.6 MHz ^{13}C NMR spectrum; $CDCl_3$ of **S55**

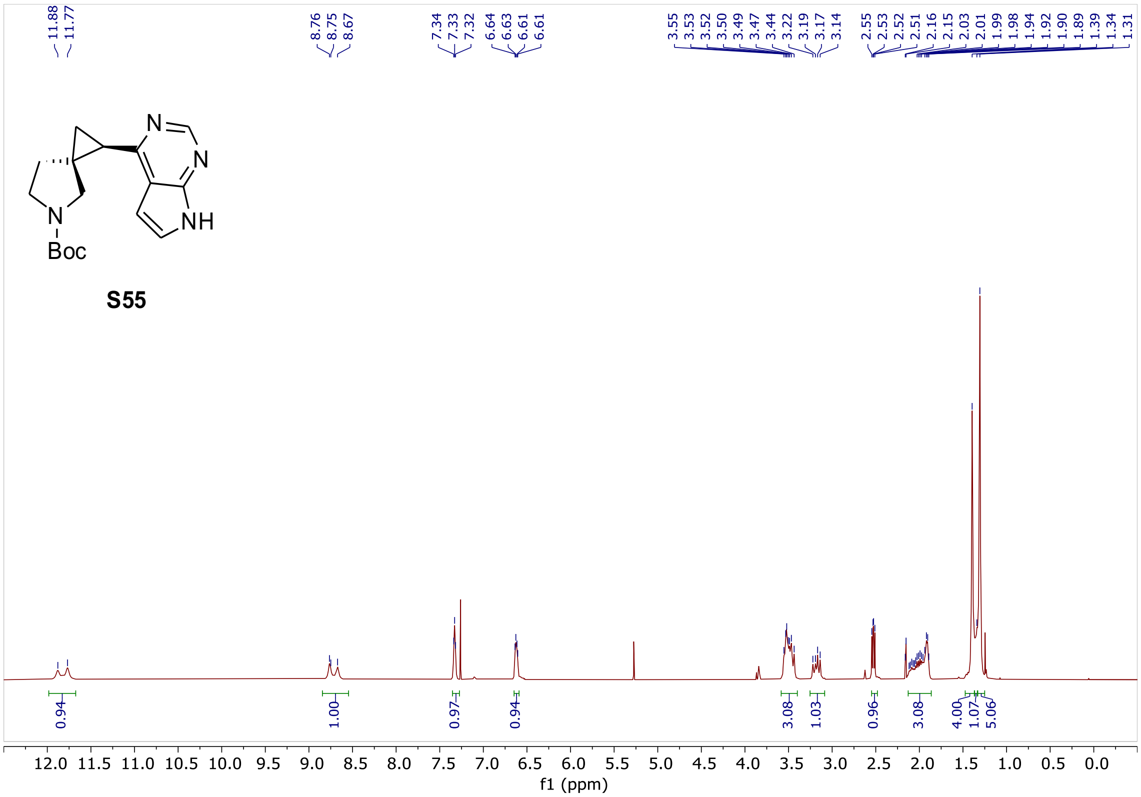

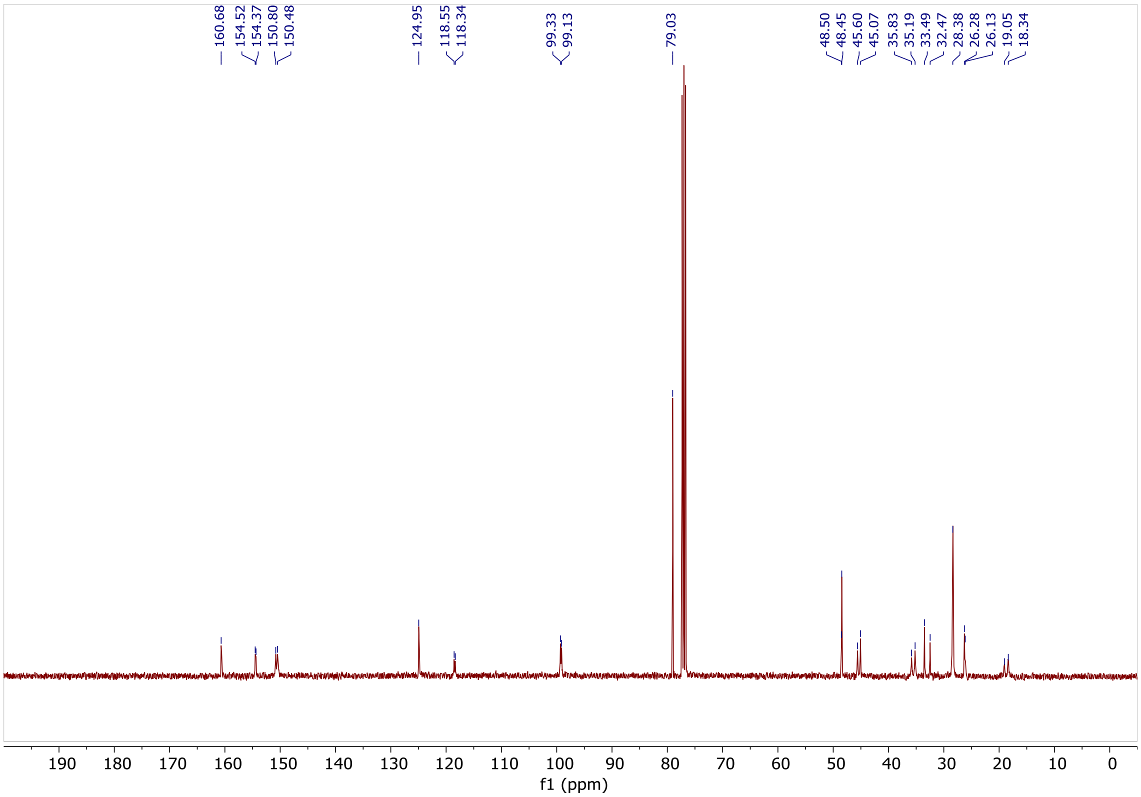

400 MHz ^{1}H NMR spectrum; 100.6 MHz ^{13}C NMR spectrum; $CDCl_3$ of **S56**

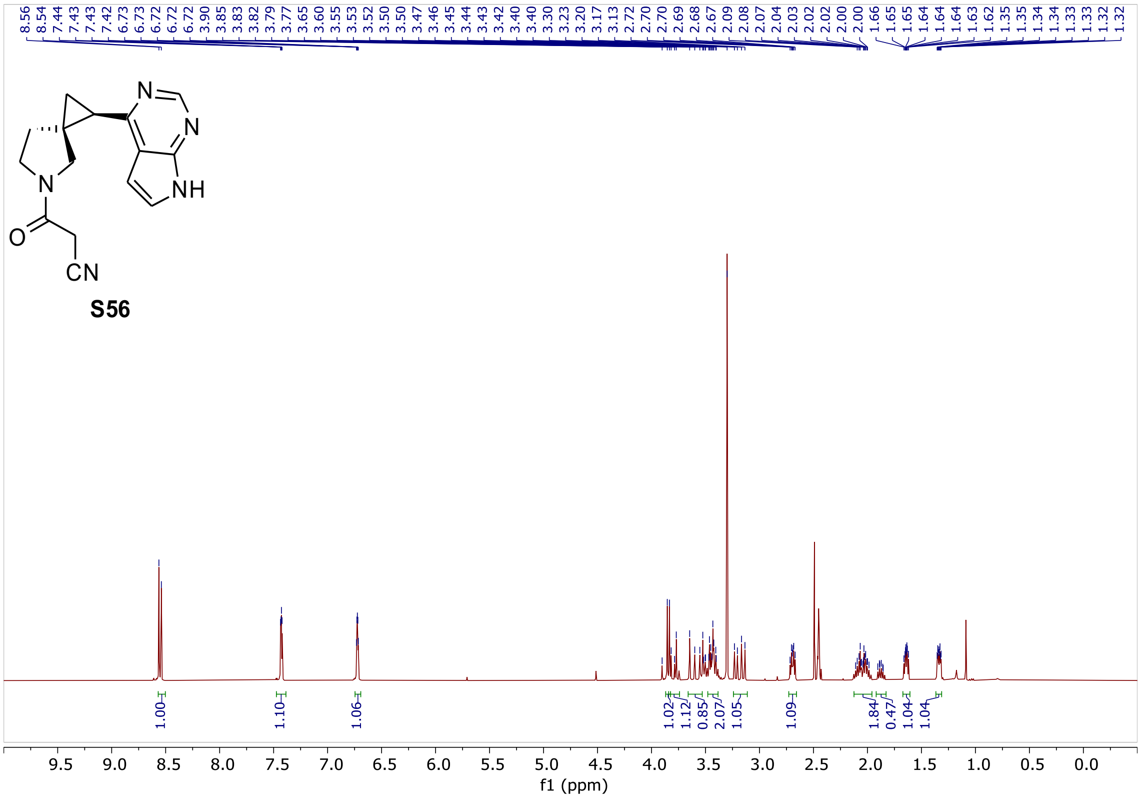

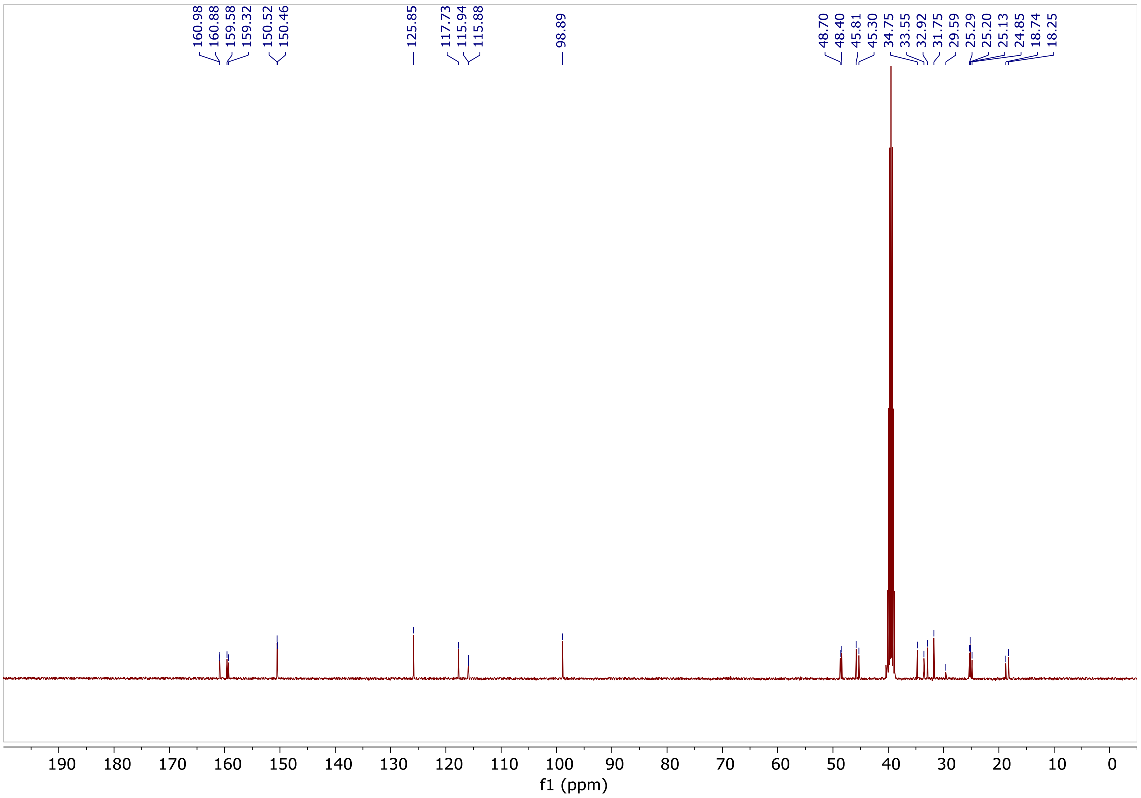

400 MHz ^1H NMR spectrum; 100.6 MHz ^{13}C NMR spectrum; $CDCl_3$ of **96**

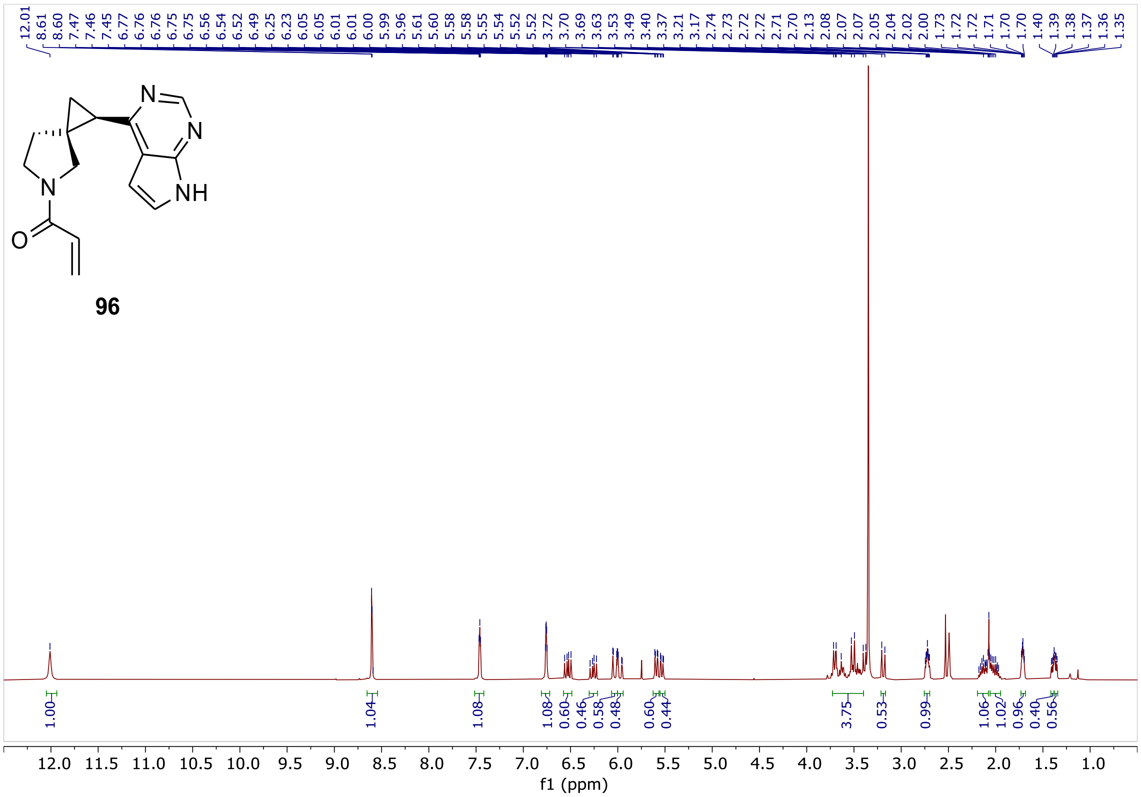

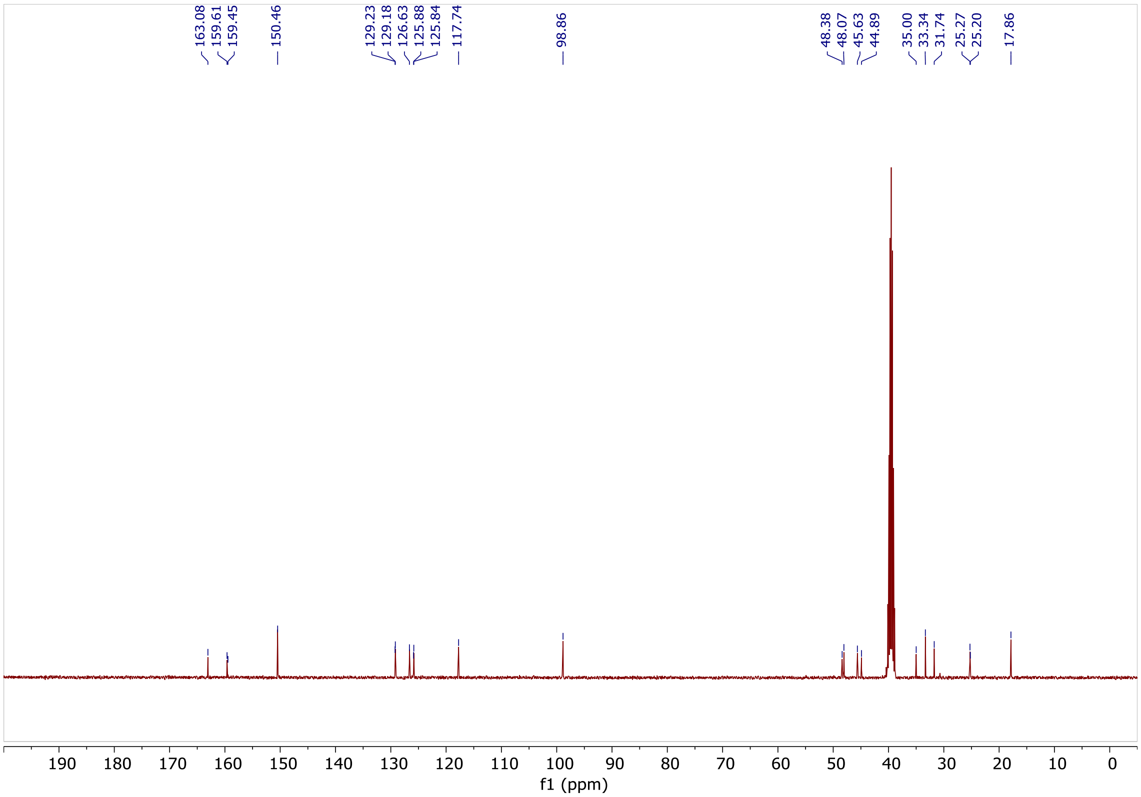

5. References

[1] P. J. Rayner, P. O'Brien, R. A. J. Horan, *J. Am. Chem. Soc.* **2013**, *135*, 8071–8077.

[2] J. Li, M. D. Burke, *J. Am. Chem. Soc.* **2011**, *133*, 13774–13777.

[3] S. Hanessian, T. Jennequin, N. Boyer, V. Babonneau, U. Soma, C. Mannoury la Cour, M. J. Millan, G. De Nanteuil, *ACS Med. Chem. Lett.* **2014**, *5*, 550–555.

[4] J. L. M. Matos, S. Vásquez-Céspedes, J. Gu, T. Oguma, R. A. Shenvi, *J. Am. Chem. Soc.* **2018**, *140*, 16976–16981.

[5] C. Dallanoce, M. Canovi, C. Matera, T. Mennini, M. De Amici, M. Gobbi, C. De Micheli, *Bioorg. Med. Chem.* **2012**, *20*, 6344–6355.

[6] G. Verniest, K. Piron, E. Van Hende, J. W. Thuring, G. MacDonald, F. Deroose, N. De Kimpe, *Org. Biomol. Chem.* **2010**, *8*, 2509–2512.

[7] M. Huang, W. Jiang, A. Jiang *Preparation Method of 3-Azabicyclo[3.1.0]Hexane Hydrochloride*, **2018**, CN108341766.

[8] O. A. Davis, R. A. Croft, J. A. Bull, *Chem. Comm.* **2015**, *51*, 15446–15449.

[9] T. Kawano, J. Kuwana, I. Ueda, *Bull. Chem. Soc. Jpn* **2003**, *76*, 789–797.

[10] Q. Zhou, A. F. Phoa, R. H. Abbassi, M. Hoque, T. A. Reekie, J. S. Font, R. M. Ryan, B. W. Stringer, B. W. Day, T. G. Johns, L. Munoz, M. Kassiou, *J. Med. Chem.* **2017**, *60*, 2052–2070.

[11] O. O. Grygorenko, P. Babenko, D. M. Volochnyuk, O. Raievskyi, I. v Komarov, *RSC Adv.* **2016**, *6*, 17595–17605.

[12] F. Lovering, J. Bikker, C. Humblet, *J. Med. Chem.* **2009**, *52*, 6752-6756.

[13] J. Wernevik, F. Bergström, A. Novén, J. Hulthe, L. Fredlund, D. Addison, J. Holmgren, P.-E. Strömstedt, E. Rehnström, T. Lundbäck, *Assay Drug Dev. Technol.* **2020**, *18*, 157-179.

[14] Schrödinger Release 2021-4: Protein Preparation Wizard; Epik, Schrödinger, LLC, New York, NY, 2021; Impact, Schrödinger, LLC, New York, NY; Prime, Schrödinger, LLC, New York, NY, **2021**.

[15] Lu,, C. Wu D. Ghoreishi, W. Chen, L. Wang, W. Damm, G. A. Ross, M. K. Dahlgren, E. Russell, C. D. Von Bargen, R. Abel, R. A. Friesner, E. D. Harder, *J. Chem. Theory. Comput.* **2021,** *17*, 4291-4300.

[16] Schrödinger Release 2021-4: LigPrep, Schrödinger, LLC, New York, NY, **2021**.

[17] R. A. Friesner, R. B. Murphy, M. P. Repasky, L. L. Frye, J. R. Greenwood, T. A. Halgren, P. C. Sanschagrin, D. T. Mainz, *J. Med. Chem.* **2006,** *49*, 6177-6196.

[18] X. Ouyang, S. Zhou, S. C. T. Su, Z. Ge, R. Li, C. K. Kwoh, *J. Comput. Chem.* **2013,** *34*, 326-336.

[19] R. A. Ward, M. J. Anderton, S. Ashton, P. A. Bethel, M. Box, S. Butterworth, N. Colclough, C. G. Chorley, C. Chuaqui, D. A. E. Cross, L. A. Dakin, J. É. Debreczeni, C. Eberlein, M. R. V. Finlay, G. B. Hill, M. Grist, T. C. M. Klinowska, C. Lane, S. Martin, J. P. Orme, P. Smith, F. Wang, M. J. Waring, M. J., *J. Med. Chem.* **2013**, *56*, 7025-7048.

[20] https://www.thermofisher.com/us/en/home/industrial/pharma-biopharma/drug-discovery-development/target-and-lead-identification-and-validation/kinasebiology/kinase-activity-assays/z-lyte.html
